# Proteomic Profiling and Functional Analysis of B Cell-Derived Exosomes upon *Pneumocystis* Infection

**DOI:** 10.1155/2022/5187166

**Published:** 2022-04-14

**Authors:** Dan Ma, Qian-Yu Zhang, Heng-Mo Rong, Kan Zhai, Zhao-Hui Tong

**Affiliations:** Department of Respiratory and Critical Care Medicine, Beijing Institute of Respiratory Medicine and Beijing Chao-Yang Hospital, Capital Medical University, Beijing 100020, China

## Abstract

*Pneumocystis* is a life-threatening fungal pathogen that frequently causes fatal pneumonia (PCP) in immunocompromised individuals. Recently, B cells have been reported to play a crucial role in the pathogenesis of PCP through producing antibodies and activating CD4^+^ T cell response. Exosomes are nanoscale small extracellular vesicles abundant with protein cargo and can mediate immune response during infectious disease. In this study, using tandem mass tag-based quantitative proteomics coupled with bioinformatic analysis, we attempted to characterize exosomes derived from B lymphocytes in response to PCP. Several proteins were verified by parallel reaction monitoring (PRM) analysis. Also, the effects of B cell exosomes on CD4^+^ T cell response and phagocytic function of macrophages were clarified. Briefly, 1701 proteins were identified from B cell exosomes, and the majority of them were reported in Vesiclepedia. A total of 51 differentially expressed proteins of B cell exosomes were found in response to PCP. They were mainly associated with immune response and transcription regulation. PRM analysis confirmed the significantly changed levels of histone H1.3, vimentin, and tyrosine-protein phosphatase nonreceptor type 6 (PTPN6). Moreover, a functional study revealed the proinflammatory profile of B cell exosomes on CD4^+^ T cell response in PCP. Taken together, our results suggest the involvement of exosomes derived from B cells in cell-to-cell communication, providing new information on the function of B cells in response to PCP.

## 1. Introduction

*Pneumocystis jirovecii* is an opportunistic fungal pathogen that causes life-threatening pneumonia in immunocompromised patients [1]. With the widespread use of highly active antiretroviral therapy in human immunodeficiency virus (HIV)-positive patients, the incidence of *Pneumocystis* pneumonia (PCP) in this population has decreased. At present, PCP is more common in HIV-negative patients [2, 3]. Importantly, HIV-negative patients often present with more severe symptoms requiring mechanical ventilation, and the mortality of these patients is higher (27%–50%) than that of patients with non-HIV PCP (4%–15%) [4, 5].

Vital roles of CD4^+^ T cells and macrophages in host defense against *Pneumocystis* have been well documented, considering that depletion of both cell populations results in the inability to control *Pneumocystis* infection [6, 7]. There is accumulating evidence indicating that B lymphocytes also participate in the immune response during PCP. Patients receiving anti-CD20 monoclonal antibody rituximab are at a high risk of developing PCP [8, 9]. Murine models of PCP have demonstrated that apart from generating *Pneumocystis-*specific antibodies, B cells could also prime CD4^+^ T cells through antigen presentation [10, 11]. Furthermore, IL-10-producing B regulatory cells exhibit an immunomodulatory function by regulating T helper (Th)1/Th17 cell responses during *Pneumocystis* infection [12]. However, the precise mechanism of the interaction between B cells and other immune cells in PCP remains to be fully elucidated.

Exosomes are small extracellular vesicles (EVs) (40–160 nm in diameter) that originate from multivesicular bodies and are released into extracellular space upon fusion with the plasma membrane. Exosomes carry biomolecules (including proteins, nucleic acids, lipids, and metabolites) and, thus, they can mediate intercellular communication under physiological and pathological conditions [13]. It is now well established that antigen-presenting cells (macrophages, dendritic cells, and B cells) can release exosomes to modulate the immune response in various types of infection [14, 15].

Previous studies have shown that B cells are capable of secreting antigen-presenting exosomes containing major histocompatibility complex (MHC) class I (MHC-I), class II (MHC-II), and costimulatory molecules, which could induce antigen-specific CD4^+^ T cell response or cytotoxic CD8^+^ T cell response [16–19]. Yet, there is also evidence that B cell-derived exosomes are immunosuppressive. For example, the Fas ligand expressed on B cell-derived exosomes could induce apoptosis in CD4^+^ T cells; therefore, it may be applied to restrain T cell-mediated responses in transplant recipients [20]. Another study has reported that CD39^+^ CD73^+^ EVs from B cells inhibit chemotherapeutic antitumor CD8^+^ T cell response [21]. Thus, the protein composition of B cell exosomes suggests their immune properties and their crucial role in mediating different immune responses. Here, we sought to purify exosomes released from lung B cells of mice and perform high-throughput tandem mass tag (TMT)-labeled quantitative proteomics [22] to characterize the exosomal protein components during PCP. Parallel reaction monitoring (PRM) targeted proteomics was carried out for validation. In addition, the effects of B cell exosomes on CD4^+^ T cell response and macrophage function were analyzed.

## 2. Materials and Methods

### 2.1. Animals

Healthy 6–8-week-old female C57BL/6 N mice and severe combined immunodeficient (SCID) mice were obtained from Vital River Lab Animal Co., Ltd (Beijing, China). The mice were housed in pathogen-free conditions and fed with autoclaved chow and water. All of the animal experiments were performed under deep anesthesia with 0.5% sodium pentobarbital (intraperitoneal injection, 50 mg/kg). All efforts were made to ameliorate animals' suffering. The animal procedures were approved by the Capital Medical University Animal Care and Use Committee.

### 2.2. Lung Infection with *Pneumocystis*

*Pneumocystis murina* (ATCC PRA-111™) was purchased from American Type Culture Collection (Manassas, VA, USA) and maintained in SCID mice as previously described [12]. Then, 1 × 10^6^*Pneumocystis* cysts diluted in 100 *μ*L phosphate buffered saline (PBS) were injected into C57BL/6 N mice trachea to generate the PCP model. Uninfected C57BL/6 N mice were inoculated with 100 *μ*L PBS.

### 2.3. Isolation of Lung B Cells

The mice were killed by exsanguination at 14 days after infection. After perfusion with 3 mL sterilized PBS through the right ventricle, lungs were extracted and minced into small pieces. Lung tissues were then digested in Roswell Park Memorial Institute (RPMI)-1640 media (Solarbio, Beijing, China) containing 10% fetal bovine serum (FBS, Hyclone, Logan, UT, USA), 50 U/mL DNase I (Sigma-Aldrich, St. Louis, MO, USA), and 1 mg/mL collagenase IV (Solarbio) for 60 min at 37°C. The digestions were filtered through 40-*μ*m cell strainer and further separated using Lymphocyte Separation Media (MP Biomedicals, Santa Ana, CA, USA) to obtain mononuclear cells. B cells from *Pneumocystis-*infected mice and uninfected control mice were isolated using CD19 MicroBeads (Miltenyi Biotec, Bergisch Gladbach, Germany) following the manufacturer's instructions. Flow cytometric analysis showed that the purity was more than 90%. In addition, as we used primary cells, the apoptosis of B cells was determined using Annexin V Apoptosis Detection Kit FITC (Invitrogen, Carlsbad, CA, USA).

### 2.4. Exosome Isolation and Characterization

Exosome-depleted FBS was prepared by ultracentrifugation at 120,000 × g for 16 h at 4°C, and the supernatant was passed through a 0.22 *μ*m polythersulfone syringe filter (Millipore, Billerica, MA, USA). Purified B cells of each group were cultured in RPMI-1640 medium supplemented with 10% exosome-depleted FBS at a density of 1.5 × 10^6^/mL. After 48 h, the cell culture supernatants were collected and centrifuged at 400 × g for 6 min to remove cells and further at 2000 × g for 30 min to remove cell debris. The supernatants were mixed with Ribo™ Exosome Isolation Reagent (RiboBio, Guangzhou, China) and incubated overnight at 4°C. The mixtures were centrifuged at 1500 × g for 30 min at 4°C, and the supernatants were aspirated. Eventually, the exosome-containing pellets were resuspended in PBS for the following experiments.

#### 2.4.1. Western Blot

Exosomal proteins were extracted with a radioimmunoprecipitation assay buffer (Solarbio) supplemented with 1 mM phenylmethylsulfonyl fluoride (Solarbio) and incubated on ice for 20 min. Protein concentration was determined using the BCA Protein Assay Kit (Pierce, Rockford, IL, USA). The protein lysates (30 *μ*g) were subjected to 10% SDS-PAGE gel for separation and transferred to polyvinylidene fluoride membranes (Millipore). The membranes were blocked with 5% nonfat powdered milk in 1 × Tris-buffered saline tween (TBST) buffer for 1 h at room temperature and then incubated with primary antibodies against ALG-2-interacting protein X (ALIX) (Proteintech, Rosemont, IL, USA, 12422-1-AP, 1 : 1000), CD9 (Abcam, Cambridge, UK, ab92726, 1 : 2000), and heat shock protein 90 (HSP90) (Cell Signaling Technology, Danvers, MA, USA, #4877, 1 : 1000) overnight at 4°C. After washing with 1 × TBST, the membranes were incubated with horseradish peroxidase-conjugated anti-rabbit antibody (Cell Signaling Technology, #7074S) for 1 h at room temperature. Bands were visualized using enhanced chemiluminescence reagent (Millipore) and detected by ChemiDoc system (Bio-Rad, Hercules, CA, USA).

#### 2.4.2. Transmission Electron Microscopy

A total of 20 *μ*L of isolated exosomes were loaded onto copper grids (Ted Pella Inc., Redding, CA, USA) and allowed to absorb for 2 min. The samples were further fixed with 2% phosphotungstic acid (Solarbio) for 10 min. After air drying at room temperature, the samples were observed under a JEM1230 transmission electron microscope (JEOL, Tokyo, Japan).

#### 2.4.3. Nanoparticle Tracking Analysis

Size distribution of exosomes was measured by ZetaView PMX 110 (Particle Metrix, Meerbusch, Germany) and its corresponding software ZetaView 8.04.02. The samples were appropriately diluted using 1 × PBS buffer (Solarbio). Brownian motion of particles was tracked and recorded by the NTA instrument at 11 different positions. Each sample was analyzed three times.

### 2.5. TMT-Based Quantitative Proteomic Analysis

#### 2.5.1. Sample Preparation

Three biological replicates of B cell exosomes isolated from *Pneumocystis-*infected and uninfected control mice were prepared. Each biological replicate was pooled from 10 mice. Quantitative proteomics was conducted by Applied Protein Technology (Shanghai, China). To extract protein, exosome samples were lysed with SDT buffer (4% (*w*/*v*) SDS, 100 mM Tris-HCl, and 0.1 M DTT, pH 7.6). The amount of protein was analyzed with the BCA method, as mentioned above. Protein digestion by trypsin was performed by employing the filter-aided sample preparation (FASP) procedure described by Wisniewski et al. [23]. The digested peptides of each sample were desalted on C18 Cartridges (Empore™ SPE Cartridges C18 (standard density), bed I.D. 7 mm, volume 3 mL, Sigma), concentrated by vacuum centrifugation, and further reconstituted in 40 *μ*L of 0.1% (*v*/*v*) formic acid.

#### 2.5.2. TMT Labeling and Peptide Fractionation

The peptide mixture (100 *μ*g) of each sample was labeled with TMT 6-Plex Isobaric Mass Tagging Kit (Thermo Scientific, Rockford, IL, USA) and then fractionated using High pH Reversed-Phase Peptide Fractionation Kit (Thermo Scientific) in accordance with the manufacturer's instructions.

#### 2.5.3. Liquid Chromatography and Tandem Mass Spectrometry (LC-MS/MS)

Each peptide fraction was subjected to Easy nLC, an HPLC liquid phase system (Proxeon Biosystems, now Thermo Fisher Scientific, Waltham, MA, USA). Eluent A was 0.1% formic acid, and eluent B was 84% acetonitrile supplemented with 0.1% formic acid. The chromatographic column was equilibrated with 95% eluent A. Then, the peptides were loaded onto a reverse phase trap column (Thermo Scientific Acclaim PepMap100, 100 *μ*m × 2 cm, nanoViper C18) and separated by the C18-reversed phase analytical column (Thermo Scientific Easy Column, 10 cm long, 75 *μ*m inner diameter, 3 *μ*m resin) at a flow rate of 300 nL/min controlled by IntelliFlow technology. The MS data were obtained using a Q Exactive mass spectrometer (Thermo Scientific) operated in the positive ion mode. The parameters were set as follows: the survey scan of precursor ions, 300–1,800 *m*/*z*; automatic gain control target, 1*e*6; maximum inject time, 50 ms; dynamic exclusion duration, 60.0 s; full scans acquired at a resolution of 70,000 at 200 *m/z*; resolution for MS/MS scans, 17,500 at 200 *m*/*z*; MS/MS activation type, high-energy collisional dissociation (HCD) fragmentation; isolation width, 2 *m/z*; normalized collision energy, 30 eV; the underfill ratio, 0.1%; the instrument was run with peptide recognition mode enabled.

#### 2.5.4. Protein Identification and Quantification

MS/MS raw data were searched by Proteome Discoverer 1.4 (Thermo Fisher Scientific) against the SwissProt database of *Mus musculus* (number of sequences: 76417, updated to January 6, 2021) using the algorithms of the MASCOT engine (Matrix Science, London, UK; version 2.2). The criteria for protein identification were as follows. Trypsin was selected as the digestive enzyme; two missed cleavages were allowed; peptide mass tolerances were set at 20 ppm for all MS spectra acquired; fragment mass tolerances were set at 0.1 Da for all MS/MS spectra acquired; fixed modifications were set as carbamidomethylation (C), TMT 6-plex (N-terminal), and TMT 6-plex (lysine, K); oxidation (methionine, M) and TMT 6-plex (tyrosine, Y) were defined as variable modifications; the peptide and protein false discovery rate (FDR) was set at ≤0.01. Proteins were quantified on the basis of the median of only unique peptides of each protein. To control experimental bias, the median protein ratio was normalized to 1. Differentially expressed proteins (DEPs) were defined as those with fold change > 1.2 (up- or downregulation) and *p* value < 0.05. The mass spectrometry proteomics data have been deposited in the ProteomeXchange Consortium via the PRIDE partner repository with the dataset identifier PXD030103.

### 2.6. Bioinformatic Analysis

Gene ontology (GO) annotation of the identified proteins and DEPs was performed using the Database for Annotation, Visualization and Integrated Discovery (DAVID, Version 6.8) [24, 25]. Protein Analysis Through Evolutionary Relationships (PANTHER) classification system was used to categorize the proteins [26]. Kyoto Encyclopedia of Genes and Genomes (KEGG) pathway enrichment analysis was conducted by Metascape [27]. All of the identified proteins were compared with the total and top 100 murine proteins recorded in Vesiclepedia (http://www.microvesicles.org, version 4.1, release date: August 15, 2018) [28, 29]. To further explore the properties of B cell exosomal proteins, the web-based tools SignalP 5.0 [30] and SecretomeP 2.0 [31] were used to predict classically and nonclassically secreted proteins, respectively.

### 2.7. PRM Analysis

To verify the results of TMT-labeled proteomics, 21 peptides of eight proteins were further quantified by LC-PRM/MS analysis. Sample preparation was the same as that for the TMT protocol. Tryptic peptides were separated using Easy nLC 1200 system (Thermo Scientific). Then, Q-Exactive HF mass spectrometry (Thermo Scientific) was applied for 60 min. Detection mode was set at positive ion mode. MS2 activation type was HCD. Peptide was quantified based on the ratio to the heavy isotope-labeled peptide DIPVPKPK. The raw data were analyzed by Skyline 3.5.0 (MacCoss Lab, University of Washington) [32].

### 2.8. CD4^+^ T Cell Proliferation Assay

CD4^+^ T cells were isolated from the spleens of two uninfected mice by CD4 (L3T4) MicroBeads (Miltenyi Biotec) in accordance with the manufacturer's instructions and then labeled with 1 *μ*M carboxyfluorescein diacetate succinimidyl ester (CFSE, BD Biosciences, San Jose, CA, USA) for 10 min at 37°C. After washing with PBS, the CFSE-labeled T cells and unlabeled control T cells were cultured in a 96-well plate at a density of 2 × 10^6^/mL and stimulated with plate-coated anti-CD3 monoclonal antibody (mAb) (5 *μ*g/mL, eBioscience, San Diego, CA, USA) and anti-CD28 mAb (2 *μ*g/mL, eBioscience). To evaluate the effect of exosomes on T cell proliferation, exosomes derived from uninfected and PCP B cells of equal quantity were added. After 72 h of culture, the cells were collected and the proliferation rate was determined by flow cytometry (FACSCanto II, BD Biosciences). The obtained data were analyzed with FlowJo software (TreeStar, Ashland, OR, USA).

### 2.9. *In Vitro* Differentiation of Th1 Cells

Naive CD4^+^ CD62L^+^ T cells from the spleens of two uninfected mice were purified with CD4^+^ CD62L^+^ T Cell Isolation Kit (Miltenyi Biotec). The isolated cells were cultured in a 96-well plate at a density of 2 × 10^6^/mL and stimulated with plate-coated anti-CD3 mAb and anti-CD28 mAb, as described above. Cytokines and mAb used for Th1 cell differentiation were IL-2 (10 ng/mL), IL-12 (10 ng/mL), and anti-IL-4 mAb (10 *μ*g/mL). Medium condition was defined as adding mere IL-2 (10 ng/mL). Coculture experiment was conducted to investigate the impact of B cell exosomes on Th1 cell differentiation rate. Naive CD4^+^ T cells were treated with exosomes released from uninfected and PCP B cells of equal quantity. The cells were cultured for 48 h and then restimulated for 4 h at 37°C with phorbol myristate acetate (PMA, 50 ng/mL, Sigma-Aldrich) and ionomycin (1 *μ*g/mL, Sigma-Aldrich) in the presence of brefeldin A (10 *μ*g/mL, Enzo Life Science, Farmingdale, NY, USA). After permeabilization, the cells were stained with anti-mouse IFN-*γ* antibody (eBioscience) and analyzed with FACSCanto II. The detailed methods are described in our previous publication [12].

### 2.10. Phagocytosis Assay

Bronchoalveolar lavage fluid (BALF) cells were collected from five uninfected mice by lavaging the lungs three times with sterile PBS using a polyethylene 18 G catheter. After centrifugation at 400 × g for 6 min, 2 × 10^5^ macrophages were treated with CON and PCP B cell exosomes (both from 1 × 10^6^ B cells) or left untreated for 2 h and then washed gently with PBS to remove nonadherent cells. Zymosan is a yeast extract, and its major component is *β*-glucan, which is the major component of the *Pneumocystis* cell wall. It can bind to several pattern recognition receptors present on macrophages and therefore can be used as a substitution for *Pneumocystis* to test the phagocytosis of this fungus. The isolated alveolar macrophages (AMs) from different groups were then incubated with pHrodo Red Zymosan A BioParticles Conjugate (Life Technologies, Carlsbad, CA, USA) for 1.5 h at 37°C, 5% CO_2_ (0.1 mg/mL, 200 *μ*L, and 20 *μ*g per well). The treated AMs were washed to remove noninternalized particles. For negative control, only AMs were added. The phagocytic function of AMs was assessed by analyzing the fluorescence intensity with flow cytometry.

### 2.11. Statistical Analysis

Statistical analyses were conducted using GraphPad Prism 7.0 (GraphPad Software, La Jolla, CA, USA). Data are presented as mean ± standard deviation. Two-tailed Student's *t* test was used for comparison between two groups. One-way ANOVA with Bonferroni post hoc test was applied for multiple comparisons. *p* values lower than 0.05 were considered statistically significant.

## 3. Results

### 3.1. Characterization of B Cell-Derived Exosomes

**Figure 1:**
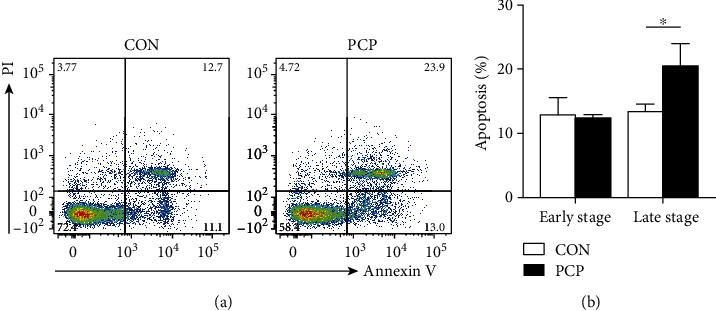
Apoptosis rate of primary B cells. B cells were extracted from the lung tissues of uninfected and *Pneumocystis-*infected mice (8–10 mice per group) using anti-CD19 MicroBeads. B cells were stained with Annexin V and PI. The proportion of apoptosis was assessed via flow cytometry. (a) Representative flow cytometric graph of B cell apoptosis. Annexin V^+^PI^−^ staining represents early apoptosis, while Annexin V^+^PI^+^ staining represents late apoptosis. (b) The comparison of early and late apoptotic percentage of B cells isolated from CON and PCP mice (*n* = 3). ^∗^*p* < 0.05 compared with the CON group by Student's *t* test. CON: uninfected control mice; PCP: *Pneumocystis* pneumonia mice.

To confirm the presence of B cell-derived exosomes, we isolated B cells from the lungs of uninfected control and *Pneumocystis-*infected mice, which were referred to as the CON group and the PCP group, respectively. The mice were killed at 2 weeks after infection, given that the histopathology exhibited the most severe inflammatory response during this period based on our previous study [12]. Interestingly, using Annexin V/Propidium Iodide (PI) staining method, we observed that pulmonary B cells from PCP mice showed increased apoptosis (late stage) (Figure 1), which might be the immunological impact of *Pneumocystis* infection.

**Figure 2:**
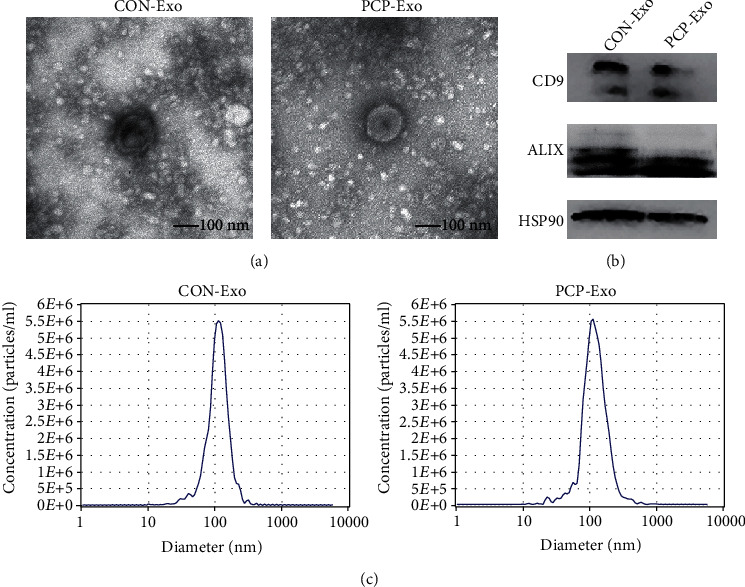
Exosome isolation and characterization. Exosomes were isolated from the culture medium of lung B cells from uninfected and *Pneumocystis-*infected mice (8–10 mice per group). (a) Electron microscopy images of exosomes. Scale bar represents 100 nm. (b) Immunoblot analysis of B cell exosomes for exosomal markers CD9, ALIX, and HSP90. (c) Size distribution of particles analyzed by nanoparticle tracking analysis. CON: uninfected control mice; PCP: *Pneumocystis* pneumonia mice; Exo: exosomes.

Exosomes were then extracted using a polyethylene glycol-based commercial reagent. The morphology of the isolated vesicles was visualized using electron microscopy and showed typical cup-shaped membrane structures with a size of approximately 100 nm (Figure 2(a)). The exosomal markers CD9, ALIX, and HSP90 were detected by western blot (Figure 2(b)). We then measured the size distribution of B cell exosomes. The diameter of the particles analyzed was slightly above 100 nm, consistent with the size range of exosomes (Figure 2(c)). These results indicated that B cells extracted from the lung tissue could generate exosomes *in vitro*.

### 3.2. Proteomic Analysis of B Cell Exosomes from Uninfected and *Pneumocystis-*Infected Mice

**Figure 3:**
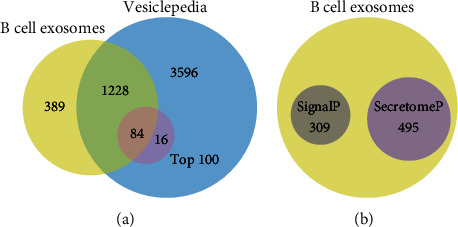
Venn diagrams of proteins identified in B cell exosomes. (a) Comparison of proteins identified in B cell-derived exosomes versus entire and top 100 murine proteins recorded in the Vesiclepedia database. (b) Identified proteins with prediction of classically (SignalP) and nonclassically secreted proteins (SecretomeP).

To unveil the protein contents of B cell exosomes comprehensively, we performed high-throughput TMT-labeled quantitative proteomics of exosomes derived from B cells of CON and PCP mice. Three independent biological replicates of each group were analyzed. The detailed information of each biological replicate is presented in Supplementary Table 1. In total, 1701 proteins were identified in B cell-derived exosomes (Supplementary Table 2). Next, we compared our mass spectrometry data with the previously published murine exosomal proteins deposited in Vesiclepedia (August 2018). The Venn diagram shows that 1312 proteins had been reported, and 84 of them were present in the top 100 ranked proteins (Figure 3(a)).

The protein secretion pathway of each identified protein was evaluated with two web-based tools SignalP and SecretomeP. SignalP analysis revealed that 309 proteins contained signal peptides needed for classical, endoplasmic reticulum- (ER-) Golgi secretion pathway (Figure 3(b), Supplementary Table 3). Meanwhile, SecretomeP analysis detected 495 proteins with neural network score (NN-score) above 0.5 and without prediction of signal peptides, which were considered as nonclassically secreted proteins (Figure 3(b), Supplementary Table 3). Notably, the remaining 897 (53%) exosomal proteins were predicted to be nonsecretory.

**Figure 4:**
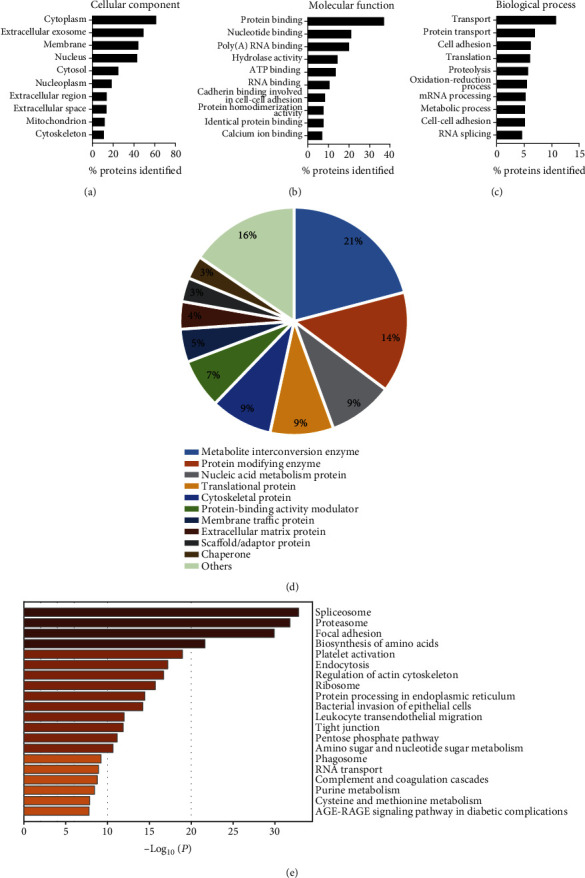
Bioinformatic analysis of the proteome of B cell exosomes. (a–c) Gene ontology annotation of the proteins identified in B cell exosomes based on (a) cellular component, (b) molecular function, and (c) biological process. (d) Pie chart showing Protein Analysis Through Evolutionary Relationships classification of the identified proteins from B cell exosomes. (e) The most enriched KEGG pathway of the proteome is visualized using Metascape.

To gain further insights into the functional profile of exosomes derived from B cells, we performed bioinformatic analysis of all of the identified proteins. GO classification based on cellular component showed that approximately 61% of the proteins were annotated with cytoplasm (cystol), followed by extracellular exosome (49%), membrane (44%), and nucleus (43%) (Figure 4(a)). When using molecular function ontology, the majority of the proteins possess protein binding activity (37%), while other functions include nucleotide binding (21%), poly (A) RNA binding (20%), hydrolase activity (14%), and ATP binding (14%) (Figure 4(b)). In terms of biological process, most proteins were involved in transport (11%), protein transport (7%), cell adhesion (6%), translation (6%), proteolysis (6%), and oxidation-reduction process (5%) (Figure 4(c)). PANTHER analysis was employed to classify the exosomal proteins. As shown in Figure 4(d), the exosomes of B cells carried different types of proteins, such as metabolic enzyme, translational protein, cytoskeletal protein, protein-binding activity modulator, and membrane traffic protein.

KEGG pathway enrichment analysis revealed that protein synthesis-related pathways were significantly enriched, including spliceosome, proteasome, ribosome, and protein processing in the ER. Immunological processes such as phagosome, endocytosis, leukocyte transendothelial migration, and complement and coagulation cascades were also overrepresented (Figure 4(e)). After retrieving the mass spectrometry data thoroughly, we found that B cell-specific markers and immunity-related molecules were abundant in exosomes (Table 1). Together, these data implied that exosomes derived from pulmonary B cells of both CON and PCP mice carried protein components suggestive of their formation and cellular origin, which probably exert functions on other immune cells.

### 3.3. *Pneumocystis* Infection Alters the Protein Composition of B Cell Exosomes

Using TMT-labeling quantitative proteomics, 51 proteins showed differential TMT signal intensity in PCP B cell exosomes with respect to the CON group. More specifically, 46 proteins were upregulated, while five proteins were downregulated, as listed in Table 2 (>1.2 for upregulated proteins and <0.83 for downregulated proteins). Based on the GO annotation, these DEPs are mainly associated with immune response, transcription regulation, and signal transduction (Supplementary Table 4). Of note, most upregulated proteins in B cell exosomes were classified as histones, which could function as damage-associated molecular pattern (DAMP) molecules in inflammatory response. Other DAMPs identified in the proteomics included protein S100-A8 and protein S100-A9, which were slightly increased in B cell exosomes after *Pneumocystis* infection, although not significantly (Table 1, Supplementary Table 2). Collectively, these data suggested that *Pneumocystis* infection stimulates pulmonary B cells to release exosomes abundant with DAMPs and other immune-relevant molecules, further impacting the communication between immune cells.

**Figure 5:**
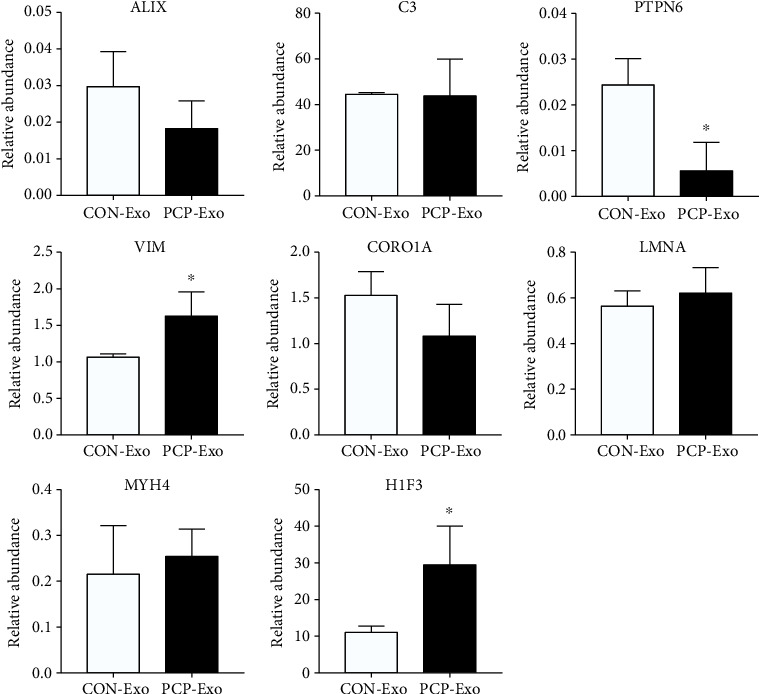
Validation of the selected proteins using PRM. Exosomes of CON and PCP B cells were isolated and subjected to LC-PRM/MS analysis. Relative protein abundance of eight proteins (ALIX, C3, PTPN6, VIM, CORO1A, LMNA, MYH4, and H1F3) was calculated based on the normalized peak area of peptide fragments. A two-tailed Student's *t* test was used to detect the difference between two groups (*n* = 3). ∗indicates *p* < 0.05.

To validate the mass spectrometry results, we utilized PRM targeted proteomics to quantify absolute protein abundance. Several proteins were selected, including exosomal marker protein ALIX, immunity-related molecules (complement C3, tyrosine-protein phosphatase nonreceptor type 6 (PTPN6)), and DEPs (coronin-1a (CORO1A), vimentin (VIM), prelamin-A/C (LMNA), myosin-4 (MYH4), and histone H1.3 (H1F3)). For each protein, between one and five peptides were chosen, and then, the targeted peptides and proteins were quantitatively analyzed (Figure 5). Detailed quantification information is shown in Supplementary Table 5. Surprisingly, the PRM analysis demonstrated that PTPN6 was significantly downregulated in *Pneumocystis-*infected B cell-derived exosomes, while the TMT quantitative proteomics of this protein did not show any prominent change (fold change 0.873, *p* value 0.011). VIM and H1F3, which were reported as DEPs in B cell exosomes, were also significantly enriched in the PCP group. In contrast, no significant differences were observed in terms of CORO1A, C3, LMNA, MYH4, and ALIX. Generally, the expression trend of these proteins using PRM analysis was in agreement with the LC-MS/MS results, although with different statistical significance.

### 3.4. Exosomes Derived from *Pneumocystis-*Infected B Cells Promote CD4^+^ T Cell Proliferation and Differentiation into Th1 Cells

**Figure 6:**
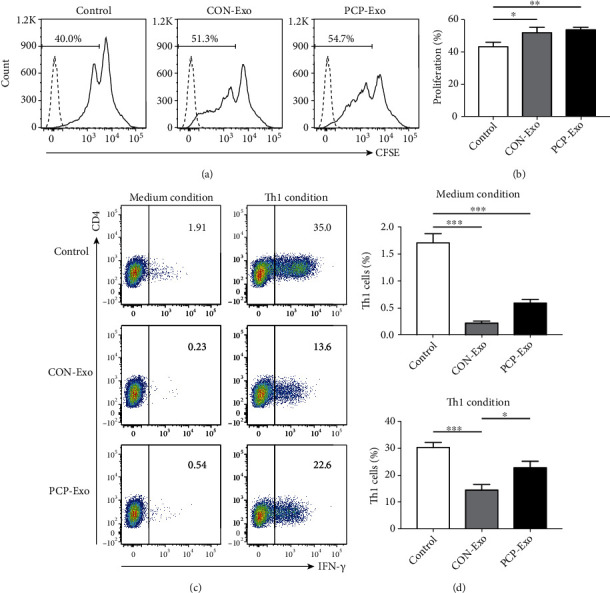
Effects of B cell-derived exosomes on CD4^+^ T cells. CD4^+^ T cells and naive CD4^+^ T cells were purified from the spleens of two wild-type mice and treated with CON and PCP B cell exosomes or left untreated (control). Proliferation and Th1 cell differentiation rate were analyzed. (a) Representative flow cytometric histograms for CFSE of CD4^+^ T cells receiving different treatments. Dotted lines represent CFSE unlabeled control. (b) The comparison result of (a). (c) Representative flow cytometric dot plots showing the differentiation of naive CD4^+^ T cells into Th1 subset and the corresponding comparison result (d). Comparisons were conducted by one-way ANOVA, followed by Bonferroni post hoc test (*n* = 3). ^∗^*p* < 0.05; ^∗∗^*p* < 0.01; ^∗∗∗^*p* < 0.001.

We next evaluated the effect of B cell-derived exosomes on major immune cells during PCP. First, CD4^+^ T cell proliferation and IFN-*γ*-expressing CD4^+^ T cell (Th1) differentiation rate were analyzed by flow cytometry. As shown in Figures 6(a) and 6(b), B cell exosomes of both groups promoted CD4^+^ T cells to proliferate significantly. The proliferation rate in the PCP group was slightly higher than that in the CON group, although not statistically significant. In contrast, B cell exosomes from both groups decreased Th1 cell expansion. However, exosomes from PCP B cells significantly enhanced the differentiation of naive CD4^+^ T cells into Th1 subset in comparison to CON B cells (Figures 6(c) and 6(d)).

**Figure 7:**
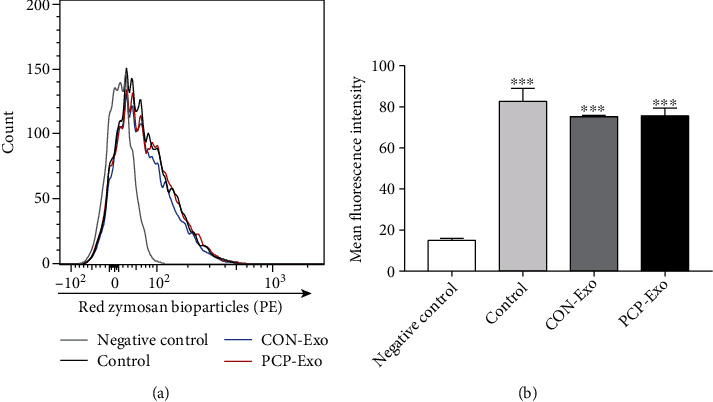
Effect of B cell-derived exosomes on phagocytic capacity of alveolar macrophages. AMs were isolated from five uninfected mice and treated with CON and PCP B cell exosomes or left untreated (control). AMs from different groups were incubated with dye-labeled zymosan particles to examine the phagocytic function. For negative control, only AMs were added. (a) Representative flow cytometric histogram showing the particle fluorescence within macrophages from different groups. (b) Comparison result of mean fluorescence intensity of four groups (*n* = 3). Comparison was conducted by one-way ANOVA, followed by Bonferroni post hoc test. ^∗∗∗^*p* < 0.001 compared with the negative control. AMs: alveolar macrophages.

AMs are innate immune cells residing in alveolar spaces and act as the first line of defense against exogenous pathogens. They are another indispensable effector cell population with their capacity to recognize and kill the *Pneumocystis* organism [7]. Previous studies have revealed that phagocytosis of *Pneumocystis* by AMs is the predominant mechanism in the clearance of this pathogen from lungs [33]. We therefore measured the effect of B cell exosomes derived from CON and PCP on the phagocytic function of AMs. A unique phagocytosis assay kit was applied based on the increasing fluorescence of pH-sensitive dye-labeled zymosan when these particles are ingested. Flow cytometry quantification analysis showed that while AMs internalized the particles with increasing fluorescence, exosomes derived from *Pneumocystis-*infected B cells had no significant influence on the phagocytic activity of AMs (Figures 7(a) and 7(b)). Overall, these findings suggested that B cell exosomes could potentiate CD4^+^ T cell immune response during PCP.

## 4. Discussion

B cells have been reported to play an increasingly important role in the immune defense against PCP. While immunological studies of B cells have mainly focused on their role as antibody-producing and antigen-presenting cells [10–12], no studies have examined the EVs of B cells and their function during *Pneumocystis* infection. Exosomes and other EVs produced by immune cells can mediate host response in bacterial, viral, and fungal respiratory infection. For instance, exosomes released from *Mycobacterium tuberculosis-*infected RAW264.7 cells could promote the recruitment of macrophages [34]. Exosomes derived from respiratory syncytial virus-infected cells induce the release of cytokines and chemokines from monocytes and airway epithelial cells, suggesting their roles in activating the innate immune response [35]. Also, fungal pathogens such as *Cryptococcus neoformans* [36], *Candida albicans* [37], and *Aspergillus fumigatus* [38] modify the EV protein components of infected immune cells, and these EVs released by the cells could facilitate the host antifungal response. In this study, we isolated exosomes released from lung B cells of uninfected and PCP mice. Exosomal proteome and its effects on other cells were characterized. As *Pneumocystis murina* cannot be cultured *in vitro* [1], an *in vivo* infection mouse model was generated. Also, the data obtained from primary cells can reflect the biological process more truly than those from cell lines.

Proteins are the ultimate performer of gene function, and the secreted proteins in the extracellular microenvironment have fundamental roles in intercellular communication. To obtain an overview of the proteome profile of B cell exosomes, mass spectrometry analysis was conducted. A total of 1701 distinct proteins were identified in exosomes from both *Pneumocystis-*infected and uninfected B cells. Comparison of these proteins with the Vesiclepedia database revealed that 77.1% of these proteins were considered exosomal, confirming the abundant levels of exosomes in our samples. Conventional ER-Golgi pathway and unconventional pathway are two types of protein secretion pathways, while the latter form is further categorized into vesicular and nonvesicular pathways [39]. Exosomes, a smaller subtype of EVs, are therefore speculated to carry proteins mainly of the unconventional pathway [40]. In our data, 495 (29.10%) proteins encapsulated in B cell exosomes were predicted to be unconventionally secreted, whereas a lower proportion (18.16%) of proteins were involved in the classical pathway based on the detection of signal peptides. The result is not surprising as previous studies have also reported the presence of signal peptides in exosomes [37, 41, 42], which indicates a more complex protein sorting mechanism during the biogenesis and secretion of exosomes. Due to the endosomal origin of exosomes, the remaining nonsecretory proteins were estimated to be cytoplasmic and membrane-associated proteins. The GO cellular component analysis also confirmed that a multitude of these identified proteins were annotated with cytoplasm and membrane. The proportion of predicted secretory and nonsecretory proteins was similar to that from a prior study that had investigated EV protein contents modulated by *Candida albicans* [37].

Exosomes have been implicated in cell-to-cell communication in inflammatory response [14]. We sought to determine how *Pneumocystis* infection impacts exosomes derived from B lymphocytes and modulates their protein cargo. We observed that most of the DEPs were involved in immune response, transcription regulation, and signal transduction. Notably, the proteome of PCP B cell exosomes contained more histones, both revealed by LC-MS/MS and by PRM validation analysis. The abundance may partly be attributed to the increased rate of apoptotic B cells during PCP [43]. Historically, histones in EVs have been recognized as specific contents in apoptotic vesicles released from dying cells. However, recent evidence suggests that histones are also present in exosomes and have other particular roles. For example, histones loaded into exosomes may be involved in a survival mechanism by eliminating harmful DNA to maintain cellular homeostasis without dying [44]. Another study has demonstrated that exosomal histones can mediate adhesion and interaction with other cells [45]. Importantly, we focused on exosomal histones for their roles as DAMPs, which could further activate immune cells and release inflammatory mediators [46]. Increased levels of histones in exosomes and EVs have been observed in some other infections, such as the key fungal component *β*-glucan infection model [47] and the Kaposi sarcoma-associated virus (KSHV) infection [48]. Hence, we hypothesized that the enrichment of histones in PCP B cell exosomes may be proinflammatory and could have effects on other immune cells.

PTPN6, also known as Src homology 2 domain-containing protein tyrosine phosphatase-1 (SHP-1), is a widely expressed cytoplasmic protein in all mature hematopoietic lineages. Intriguingly, PRM targeted analysis revealed that PTPN6 was significantly downregulated in PCP B cell exosomes. SHP-1 (PTPN6) exerts its regulatory role on T cells by inhibiting antigen-dependent activation and proliferation in *Leishmania* infection and other inflammatory conditions [49, 50]. Moreover, in a tumor model of melanoma, knockdown of SHP-1 (PTPN6) expands antitumor T cell repertoire and dampens tumor growth, suggesting the potential application of SHP-1 (PTPN6) as immune checkpoint in future immune therapy [51]. A previous study showed that when B cells were infected with KSHV, PTPN6 expression in B cell-derived exosomes was also significantly downregulated [48].

Another DEP validated by PRM analysis is VIM. It belongs to the intermediate filament cytoskeletal protein ubiquitously located in nonepithelial cells, including immune cells such as lymphocytes and macrophages [37, 47, 52–54]. Despite being a cytoskeleton component, VIM could be secreted by activated phagocytes to reinforce the bactericidal activity and augment the immune response [53]. VIM secretion via EVs has also been reported in fungal infection of macrophages with higher expression level compared with uninfected cells, which is consistent with our results [37, 47]. Besides, Epstein-Barr virus infection of B cell line induces dramatic increase in VIM mRNA and protein expression, further implying a possible role of VIM in infectious diseases [54].

Based on the enrichment of proinflammatory DAMP histones and immunity-related cytoskeleton VIM and the decreased level of the inhibitory molecule PTPN6, we inferred that exosomes released from B cells in PCP may have immune effects on other immune cells. While B cells have a critical role in activating CD4^+^ T cells in PCP, their significance in CD8^+^ T cells has not been reported. Furthermore, the role of CD8^+^ T cells in PCP is still questionable, with both protective and detrimental responses reported [55, 56]. Therefore, we first tested the proliferation of CD4^+^ T cells exposed to B cell exosomes. Significantly improved proliferation of CD4^+^ T cells by both groups of B cell exosomes was demonstrated. However, the expected immune stimulation role of *Pneumocystis-*infected B cell exosomes was not obvious. Subsequently, the differentiation of naive CD4^+^ T cells into Th1 cells was measured. We found that although B cell exosomes generally suppressed Th1 differentiation, exosomes derived from PCP mice promoted Th1 differentiation compared with those from uninfected mice. AMs are ultimately responsible for the innate immune response against PCP. The phagocytosis assay demonstrated that fluorescence in AMs increased significantly when incubated with zymosan, implying that AMs have a role in the elimination of *Pneumocystis*. In contrast, no effect of B cell exosomes was observed on the phagocytosis of macrophages. It is still unknown whether DAMPs and other immunological molecules identified in B cell exosomes could induce phagocytosis. In general, AMs have to be activated by host cytokines, such as IFN-*γ*, TNF-*α*, and granulocyte macrophage colony stimulating factor (GM-CSF), to maximize their phagocytic activity [7]. However, these cytokines were not identified by mass spectrometry. The relationship between B cells and macrophages during PCP was also not studied. Altogether, the alteration of the abovementioned immunological proteins in B cell exosomes in response to PCP could be responsible for the proinflammatory effect on CD4^+^ T cells. Further functional studies should be performed to elucidate the precise mechanism.

## 5. Conclusion

In conclusion, our findings suggest that B cells can secrete exosomes abundant in immune active protein contents, and these vesicles could modulate other immune cells such as CD4^+^ T cells in response to *Pneumocystis* infection. This work provides new insights into the pathogenesis of PCP for the possible role of exosomes derived from immune cells. Future investigations are warranted to unravel the involvement and function of exosomes from other immune effector cells during PCP.

## Figures and Tables

**(a):** 

Uniprot accession no.	Protein name	Gene symbol
P19437	B-lymphocyte antigen CD20	Ms4a1
P06800	Receptor-type tyrosine-protein phosphatase C	Ptprc
P35329	B cell receptor CD22	Cd22
P21855	B cell differentiation antigen CD72	Cd72
Q61470	Leukocyte antigen CD37	Cd37
P11911	B cell antigen receptor complex-associated protein alpha chain	Cd79a
P15530	B cell antigen receptor complex-associated protein beta chain	Cd79b

**(b):** 

Uniprot accession no.	Protein name	Gene symbol
P01899	H-2 class I histocompatibility antigen, D-B alpha chain	H2-D1
P01901	H-2 class I histocompatibility antigen, K-B alpha chain	H2-K1
P14438	H-2 class II histocompatibility antigen, A-U alpha chain (fragment)	H2-Aa
P14483	H-2 class II histocompatibility antigen, A beta chain	H2-Ab1
P01027	Complement C3	C3
P01029	Complement C4-B	C4b
Q9ES30	Complement C1q tumor necrosis factor-related protein 3	C1qtnf3
P06684	Complement C5	C5
P04186	Complement factor B	Cfb
Q8CFG9	Complement C1r-B subcomponent	C1rb
Q8BH35	Complement component C8 beta chain	C8b
Q64735	Complement component receptor 1-like protein	Cr1l
P01837	Immunoglobulin kappa constant	Igkc
Q9EQS9	Immunoglobulin superfamily DCC subclass member 4	Igdcc4
P01872	Immunoglobulin heavy constant mu	Ighm
P01749	Ig heavy chain V region 3	Ighv1-61
P04202	Transforming growth factor beta-1 proprotein	Tgfb1
P82198	Transforming growth factor-beta-induced protein ig-h3	Tgfbi
P27005	Protein S100-A8	S100a8
P31725	Protein S100-A9	S100a9
P51437	Cathelicidin antimicrobial peptide	Camp

**Table 2:** Differentially expressed proteins in B cell exosomes in response to *Pneumocystis* infection.

Uniprot accession no.	Protein name	Gene symbol	Fold change	*p* value
Immune response				
O35744	Chitinase-like protein 3	Chil3	2.076	7.515*E*-05
Q8C2K1	Differentially expressed in FDCP 6	Def6	1.305	1.464*E*-02
Q3SXB8	Collectin-11	Colec11	1.282	2.262*E*-02
P11911	B cell antigen receptor complex-associated protein alpha chain	Cd79a	1.260	3.765*E*-02
P01749	Ig heavy chain V region 3	Ighv1-61	1.236	1.096*E*-02
P19467	Mucin-13	Muc13	1.423	2.680*E*-04
Damage-associated molecular patterns				
P43277	Histone H1.3	H1f3	2.218	1.969*E*-02
P27661	Histone H2AX	H2ax	2.037	2.325*E*-02
P43274	Histone H1.4	H1f4	1.867	1.018*E*-02
P0C0S6	Histone H2A.Z	H2az1	1.701	7.582*E*-03
P43276	Histone H1.5	H1f5	1.660	4.329*E*-03
Q6GSS7	Histone H2A type 2-A	H2ac18	1.595	3.061*E*-02
P15864	Histone H1.2	H1f2	1.572	4.342*E*-02
P68433	Histone H3.1	H3c1	1.569	4.227*E*-03
Q8CGP7	Histone H2A type 1-K	H2ac15	1.564	4.561*E*-02
P62806	Histone H4	H4c1	1.516	3.010*E*-03
Q8CGP0	Histone H2B type 3-B	H2bu1-ps	1.513	3.093*E*-04
P10854	Histone H2B type 1-M	H2bc14	1.475	1.079*E*-03
P10922	Histone H1.0	H1f0	1.465	3.718*E*-02
P02301	Histone H3.3C	H3f3c	1.458	6.179*E*-03
Transcription regulation				
Q9CQE8	RNA transcription, translation and transport factor protein	RTRAF	2.014	4.639*E*-02
O54962	Barrier-to-autointegration factor	Banf1	1.746	2.677*E*-02
Q61116	Zinc finger protein 235	Znf235	1.365	2.023*E*-02
Q9Z277	Tyrosine-protein kinase BAZ1B	Baz1b	1.352	7.932*E*-06
P42586	Homeobox protein Nkx-2.2	Nkx2-2	1.272	4.253*E*-02
Q04750	DNA topoisomerase 1	Top1	1.237	1.698*E*-03
Q3TWW8	Serine/arginine-rich splicing factor 6	Srsf6	0.790	1.047*E*-03
Q9Z130	Heterogeneous nuclear ribonucleoprotein D-like	Hnrnpdl	0.639	9.385*E*-04
Signal transduction				
P70296	Phosphatidylethanolamine-binding protein 1	Pebp1	1.443	7.441*E*-03
P56716	Oxygen-regulated protein 1	Rp1	1.843	7.102*E*-03
Q7TSJ6	Serine/threonine-protein kinase LATS2	Lats2	1.245	4.905*E*-04
Q60870	Receptor expression-enhancing protein 5	Reep5	1.308	2.552*E*-02
Metabolism				
P16331	Phenylalanine-4-hydroxylase	Pah	1.390	3.699*E*-03
P00688	Pancreatic alpha-amylase	Amy2	1.229	4.990*E*-03
Vesicle trafficking				
Q8K012	Formin-binding protein 1-like	Fnbp1l	1.293	1.909*E*-02
Q3UQN2	F-BAR domain only protein 2	Fcho2	1.200	1.819*E*-04
P50396	Rab GDP dissociation inhibitor alpha	Gdi1	1.250	9.281*E*-03
Cytoskeletal components				
Q5SX39	Myosin-4	Myh4	2.690	2.488*E*-03
Q91Z83	Myosin-7	Myh7	1.643	4.466*E*-02
P20152	Vimentin	Vim	1.322	2.314*E*-02
Q62418	Drebrin-like protein	Dbnl	0.833	2.465*E*-02
O89053	Coronin-1A	Coro1a	0.829	2.040*E*-03
Q9QYB5	Gamma-adducin	Add3	0.801	4.797*E*-02
P48678	Prelamin-A/C	Lmna	1.264	3.074*E*-02
Ribosome components				
Q9CZT6	Protein CMSS1	Cmss1	1.362	4.707*E*-02
P47915	60S ribosomal protein L29	Rpl29	1.325	3.513*E*-03
Miscellaneous				
Q3UPH1	Protein PRRC1	Prrc1	1.542	1.967*E*-03
O55143	Sarcoplasmic/endoplasmic reticulum calcium ATPase 2	Atp2a2	1.224	4.780*E*-02
Q9QZR9	Collagen alpha-4(IV) chain	Col4a4	1.219	3.126*E*-03
Q921G7	Electron transfer flavoprotein-ubiquinone oxidoreductase, mitochondrial	Etfdh	1.204	1.111*E*-02
P62309	Small nuclear ribonucleoprotein G	Snrpg	1.202	2.930*E*-02

## Data Availability

The mass spectrometry proteomics data have been deposited in the ProteomeXchange Consortium via the PRIDE partner repository with the dataset identifier PXD030103. Other data supporting the findings are available from the corresponding author upon request.

## Supplementary Materials

Supplementary Materials Supplementary Table 1: initial culture cell number and protein amount of B cell exosomes used for mass spectrometry. Supplementary Table 2: all identified proteins of uninfected and *Pneumocystis*-infected B cell exosomes. Supplementary Table 3: SignalP and SecretomeP prediction results. Supplementary Table 4: differentially expressed proteins of B cell exosomes in response to PCP. Supplementary Table 5: quantitative information of peptides and proteins by PRM analysis.
