# Supplementary material for: Proteomic Profiling and Functional Analysis of B Cell-Derived Exosomes upon Pneumocystis Infection
Source: J Immunol Res. 2022 Apr 14;2022:5187166. doi: 10.1155/2022/5187166 (PMC9023222; doi:10.1155/2022/5187166)
Supplement: Supplementary Materials — Supplementary Table 1: initial culture cell number and protein amount of B cell exosomes used for mass spectrometry. Supplementary Table 2: all identified proteins of uninfected and Pneumocystis-infected B cell exosomes. Supplementary Table 3: SignalP and SecretomeP prediction results. Supplementary Table 4: differentially expressed proteins of B cell exosomes in response to PCP. Supplementary Table 5: quantitative information of peptides and proteins by PRM analysis. [file 5187166.f1.zip › Supplementary Table 1-initial culture cell number and protein amount of B-cell exosomes used for mass spectrometry.pdf]

**Supplementary Table 1: initial culture cell number and protein amount of B-cell exosomes used for mass spectrometry**

| Sample Name | CON-1 | CON-2 | CON-3 | PCP-1 | PCP-2 | PCP-3 |
|---|---|---|---|---|---|---|
| Initial Culture Cell Number | 1.25E+07 | 1.17E+07 | 1.28E+07 | 1.02E+07 | 1.04E+07 | 9.53E+06 |
| Protein Concentration (µg/µL) | 2.413 | 2.474 | 2.182 | 2.984 | 2.6 | 2.305 |
| Volume (µL) | 300 | 300 | 300 | 300 | 300 | 300 |
| Protein Amount (µg) | 723.9 | 742.2 | 654.6 | 895.2 | 780 | 691.5 |
