# Supplementary material for: Proteomic Profiling and Functional Analysis of B Cell-Derived Exosomes upon Pneumocystis Infection
Source: J Immunol Res. 2022 Apr 14;2022:5187166. doi: 10.1155/2022/5187166 (PMC9023222; doi:10.1155/2022/5187166)
Supplement: Supplementary Materials — Supplementary Table 1: initial culture cell number and protein amount of B cell exosomes used for mass spectrometry. Supplementary Table 2: all identified proteins of uninfected and Pneumocystis-infected B cell exosomes. Supplementary Table 3: SignalP and SecretomeP prediction results. Supplementary Table 4: differentially expressed proteins of B cell exosomes in response to PCP. Supplementary Table 5: quantitative information of peptides and proteins by PRM analysis. [file 5187166.f1.zip › Supplementary Table 2-all identified proteins of uninfected and pneumocystis infected B-cell exosomes.pdf]

Supplementary Table 2：all identified proteins of uninfected and pneumocystis infected B-cell exosomes

Accession	Protein Name	Gene Name	Function	Description	Coverage	Unique Peptides	Peptides	PSMs	AAs	MW [kDa]	calc. pI	CON-1/REF	CON-1/REF Count	CON-1/REF Variability [%]	CON-2/REF	CON-2/REF Count	CON-2/REF Variability [%]	CON-3/REF	CON-3/REF Count	CON-3/REF Variability [%]	PCP-1/REF	PCP-1/REF Count	PCP-1/REF Variability [%]	PCP-2/REF	PCP-2/REF Count	PCP-2/REF Variability [%]	PCP-3/REF	PCP-3/REF Count	PCP-3/REF Variability [%]	Identified or Quantified
P63260	Actin, cytoplasmic 2	Actg1	FUNCTION: Actins are highly conserved prot	Actin, cytoplasmic 2 OS=M	59.47	1	18	294	375	41.76579469	5.478027344	0.957405534	19	7.679037281	0.99451979	19	8.557022193	1.131286847	19	9.843387975	0.886560019	19	8.943186355	0.951980431	19	7.485347321	1.028211815	19	8.725875606	Quantified
P60710	Actin, cytoplasmic 1	Actb	FUNCTION: Actin is a highly conserved prote	Actin, cytoplasmic 1 OS=M	59.47	1	18	289	375	41.70973209	5.478027344	0.907531848	14	9.926821835	1.053811283	14	7.624010548	1.155750729	14	11.55789426	0.894227099	14	14.27497288	0.997552104	14	14.58441427	0.986568743	14	10.50280608	Quantified
P26039	Talin-1	Tln1	FUNCTION: Probably involved in connections	Talin-1 OS=Mus musculus	49.67	90	104	274	2541	269.6531256	6.176269531	1.012267029	246	5.168533626	1.026575585	246	5.084733639	1.012198141	246	5.376244482	0.993187261	246	5.739355902	0.958629939	246	7.259159109	0.984572819	245	5.379103547	Quantified
P62737	Actin, aortic smooth muscle	Acta2	FUNCTION: Actins are highly conserved prot	Actin, aortic smooth muscle	58.62	8	18	222	377	41.98180776	5.389160156	1.001029996	42	4.341925896	1.028434514	42	7.827071844	0.945916868	42	11.67799683	1.014615157	42	5.631671743	1.024629226	42	5.192533355	1.01917143	42	4.620634569	Quantified
P01027	Complement C3	C3	FUNCTION: C3 plays a central role in the act	Complement C3 OS=Mus m	9.8	20	20	326	1663	186.365641	6.727050781	0.978689864	326	5.964967565	1.00708185	326	6.186177264	1.006189613	326	6.75396225	0.9959963	326	9.408744368	0.944832326	326	10.72018808	1.012803262	326	5.753388153	Quantified
P13020	Gelsolin	Gsn	FUNCTION: Calcium-regulated, actin-modula	Gelsolin OS=Mus musculus	31.41	24	24	251	780	85.88808564	6.176269531	1.015889725	251	4.977875319	1.022005589	251	4.240300103	1.001821569	251	4.573132652	1.003311855	251	5.068345197	0.944464204	251	7.513620634	0.99697385	251	5.641536582	Quantified
P16546	Spectrin alpha chain, non-erythrocytic 1	Sptan1	FUNCTION: Fodrin, which seems to be involv	Spectrin alpha chain, non-e	45.15	102	102	246	2472	284.4222966	5.325683594	0.993428238	246	4.689283856	0.984665685	246	5.035796259	0.98032512	246	6.292298313	1.04206815	246	6.452722041	0.998087472	246	8.063880247	1.007479297	246	5.673150877	Quantified
P07901	Heat shock protein HSP 90-alpha	Hsp90aa1	FUNCTION: Molecular chaperone that promo	Heat shock protein HSP 90	51.3	20	40	225	733	84.73476896	5.008300781	1.020941097	94	3.743811431	1.023162813	94	6.461282771	1.0044046	94	6.094502809	0.965649529	94	7.520471924	0.960585365	94	7.405663975	0.994730802	94	6.533572028	Quantified
P11499	Heat shock protein HSP 90-beta	Hsp90ab1	FUNCTION: Molecular chaperone that promo	Heat shock protein HSP 90	52.62	19	41	239	724	83.22912123	5.033691406	1.026174115	98	5.73226262	1.03527967	98	5.02808016	1.030117412	98	7.588067572	0.962854833	98	7.588338703	0.948184883	98	10.26156385	0.976540041	98	5.559642861	Quantified
P62806	Histone H4	H4c1	FUNCTION: Core component of nucleosome.	Histone H4 OS=Mus muscu	53.4	8	8	194	103	11.3603823	11.35595703	0.805807901	194	13.82767308	0.80787076	194	11.58915197	0.852406665	194	12.24235643	1.26775815	194	10.45949588	1.345100988	194	14.58584402	1.125838089	194	10.46551366	Quantified
P11276	Fibronectin	Fn1	FUNCTION: Fibronectins bind cell surfaces a	Fibronectin OS=Mus muscu	16.92	34	34	193	2477	272.3678259	5.592285156	0.989862579	193	6.122959693	1.014745795	193	5.432188431	1.020403552	193	8.768597225	1.007203438	193	7.697845247	0.936337994	193	11.56150008	0.992205501	193	5.897619215	Quantified
P20152	Vimentin	Vim	FUNCTION: Vimentins are class-III intermedi	Vimentin OS=Mus musculu	71.03	28	34	181	466	53.65506578	5.122558594	0.936743826	145	6.413036477	0.864563301	145	11.33658796	0.830470547	145	10.73397382	1.231567807	145	13.29042691	1.233420532	145	12.54699433	1.015088769	145	5.368588959	Quantified
Q8CGP0	Histone H2B type 3-B	H2bu1-ps	FUNCTION: Core component of nucleosome.	Histone H2B type 3-B OS=	62.7	3	9	266	126	13.89953738	10.31591797	0.769579288	69	7.95675506	0.851285886	69	5.517311209	0.828888343	69	7.182052352	1.182769635	69	6.469446963	1.264111087	69	7.669829244	1.259604687	69	5.988708917	Quantified
Q9R0G6	Cartilage oligomeric matrix protein	Comp	FUNCTION: May play a role in the structural i	Cartilage oligomeric matrix	14.83	9	10	134	755	82.2894669	4.640136719	1.00339272	119	6.225972733	0.98431026	119	6.415245587	1.025030314	119	6.499694039	0.98936257	119	6.200112088	1.017016234	119	8.302081369	1.006819553	119	5.041221278	Quantified
Q02566	Myosin-6	Myh6	FUNCTION: Muscle contraction.	Myosin-6 OS=Mus musculu	38.8	24	70	154	1938	223.4264841	5.731933594	1.004672979	47	5.818870862	0.965820634	47	10.2564758	0.650980655	47	22.80966515	1.185367606	47	8.917987524	1.154455859	47	10.91655111	1.100215775	47	8.082953172	Quantified
P10854	Histone H2B type 1-M	H2bc14	FUNCTION: Core component of nucleosome.	Histone H2B type 1-M OS=	62.7	3	9	281	126	13.92756868	10.31591797	0.778920648	84	8.665192834	0.893519586	84	6.432213711	0.809725094	84	9.091114415	1.159025161	84	7.202767065	1.236874803	84	6.85296968	1.264072807	84	7.651144375	Quantified
Q8BTM8	Filamin-A	Flna	FUNCTION: Actin binding protein that promot	Filamin-A OS=Mus musculu	34.3	64	70	160	2647	281.0458908	6.036621094	1.017580606	150	5.891968226	1.025286177	150	5.636522343	1.011857916	150	6.684635784	0.982508916	150	6.682062447	0.951084779	150	9.976849409	0.98054288	150	7.045810224	Quantified
Q61703	Inter-alpha-trypsin inhibitor heavy chain H2	Itih2	FUNCTION: May act as a carrier of hyalurona	Inter-alpha-trypsin inhibitor	12.26	16	16	222	946	105.8614403	7.269042969	0.99621257	222	4.518228244	1.056082578	222	5.551438446	1.022029133	222	5.442647205	0.922519357	222	8.288805407	1.015039729	222	6.709197173	0.988556548	222	5.913957427	Quantified
Q64727	Vinculin	Vcl	FUNCTION: Actin filament (F-actin)-binding p	Vinculin OS=Mus musculus	65.95	62	62	168	1066	116.644313	5.998535156	0.960990766	168	4.348857269	1.062081327	168	7.118327703	0.995170623	168	5.384386413	0.943277994	168	9.904314561	1.06069119	168	7.113624223	0.991305742	168	5.493815999	Quantified
Q8VDD5	Myosin-9	Myh9	FUNCTION: During cell spreading, plays an i	Myosin-9 OS=Mus musculu	35.26	51	65	138	1960	226.2315611	5.655761719	0.977279019	110	5.669961776	0.974147259	110	6.854214697	0.974773627	110	7.43005053	1.037109504	110	6.546045283	1.007850937	110	7.895237772	1.03125979	110	6.558768084	Quantified
P57780	Alpha-actinin-4	Actn4	FUNCTION: F-actin cross-linking protein whic	Alpha-actinin-4 OS=Mus m	51.1	33	44	159	912	104.9114272	5.414550781	1.017699475	117	4.22285207	0.999148889	117	4.820243958	0.996859606	117	5.621270716	1.013264656	117	6.232440964	0.956530028	117	7.821044398	1.006961971	117	5.222402578	Quantified
Q8CGP7	Histone H2A type 1-K	H2ac15	FUNCTION: Core component of nucleosome.	Histone H2A type 1-K OS=	68.46	3	9	117	130	14.14096628	11.04833984	0.721248528	15	14.72649823	0.778707582	15	14.11929924	0.936763606	15	7.614351001	1.271885843	15	17.1990312	1.522107977	15	7.679491928	1.016035498	15	9.519613171	Quantified
P63017	Heat shock cognate 71 kDa protein	Hspa8	FUNCTION: Molecular chaperone implicated	Heat shock cognate 71 kDa	53.56	21	30	123	646	70.827216	5.516113281	0.993116229	68	3.776843864	1.011684682	68	5.425120574	1.026904758	68	6.404831803	1.000969556	68	6.490698505	0.976243817	68	9.118311523	0.979652073	68	6.208537913	Quantified
Q6GSS7	Histone H2A type 2-A	H2ac18	FUNCTION: Core component of nucleosome.	Histone H2A type 2-A OS=	68.46	4	9	117	130	14.08689035	10.90185547	0.757040766	18	16.72578619	0.796940707	18	11.83906916	0.872746573	18	7.251911789	1.315678666	18	14.84082124	1.524099728	18	10.1711541	1.030884659	18	4.726768327	Quantified
P58252	Elongation factor 2	Eef2	FUNCTION: Catalyzes the GTP-dependent ri	Elongation factor 2 OS=Mu	48.25	37	38	121	858	95.25289228	6.829589844	1.027437475	117	5.328304381	1.011512659	117	6.369377408	1.054159171	117	7.83900482	0.964597341	117	6.844253491	0.930043276	117	8.607035309	0.976832105	117	7.807633528	Quantified
P52480	Pyruvate kinase PKM	Pkm	FUNCTION: Glycolytic enzyme that catalyzes	Pyruvate kinase PKM OS=	58.95	32	33	108	531	57.80800753	7.474121094	1.029198707	95	4.693341312	1.051080462	95	5.347274719	1.036349684	95	5.887706761	0.956315889	95	8.398195712	0.943151798	95	8.646774565	0.971621647	95	5.725493508	Quantified
P17182	Alpha-enolase	Eno1	FUNCTION: Glycolytic enzyme the catalyzes	Alpha-enolase OS=Mus mu	66.82	22	25	83	434	47.11121751	6.800292969	1.042308667	74	4.408634899	1.073066058	74	6.26927625	1.076102315	74	6.648979042	0.914034888	74	7.192815847	0.915355269	74	7.813423401	0.936246008	74	7.107838297	Quantified
Q91Z83	Myosin-7	Myh7	FUNCTION: Myosins are actin-based motor n	Myosin-7 OS=Mus musculu	24.19	1	46	103	1935	222.7405309	5.757324219	0.987973884	1		0.894506916	1		0.464852138	1		1.409173078	1		1.265596922	1		1.181881395	1		Quantified
P35441	Thrombospondin-1	Thbs1	FUNCTION: Adhesive glycoprotein that medi	Thrombospondin-1 OS=Mu	20.34	23	25	122	1170	129.5637822	4.957519531	0.978807591	120	5.705882555	1.027429617	120	5.692037489	1.009812695	120	9.783125639	1.004478472	120	12.90503528	0.923398649	120	11.75439046	0.992253255	120	6.314197045	Quantified
Q62261	Spectrin beta chain, non-erythrocytic 1	Sptbn1	FUNCTION: Fodrin, which seems to be involv	Spectrin beta chain, non-er	39.61	84	84	163	2363	274.0524673	5.579589844	0.996588782	161	4.148701996	0.989857007	161	5.643613489	0.976048725	161	5.914398467	1.02727978	161	6.813279147	1.001076222	161	8.114989423	1.013060626	161	4.647513884	Quantified
Q9Z1T2	Thrombospondin-4	Thbs4	FUNCTION: Adhesive glycoprotein that medi	Thrombospondin-4 OS=Mu	13.19	8	10	62	963	106.2988711	4.665527344	0.987624787	44	5.302964821	0.978274996	44	5.931838401	1.093825336	44	14.56715412	0.939228938	44	11.28628008	0.933437603	44	12.64391346	0.993790139	44	4.901120609	Quantified
P50247	Adenosylhomocysteinase	Ahcy	FUNCTION: Adenosylhomocysteine is a com	Adenosylhomocysteinase O	43.06	16	18	91	432	47.65726283	6.536621094	0.989944091	82	4.327514342	1.013836195	82	4.447881385	1.003531871	82	5.763217834	1.013089403	82	4.726586467	0.967086298	82	9.052658505	0.990731089	82	5.378602456	Quantified
Q01853	Transitional endoplasmic reticulum ATPase	Vcp	FUNCTION: Necessary for the fragmentation	Transitional endoplasmic re	45.66	33	34	82	806	89.26572387	5.262207031	1.019250793	80	5.621968337	1.017277975	80	5.404311542	1.051347691	81	7.034232066	0.964558324	81	5.771522232	0.91474905	81	9.874355879	0.981305329	81	6.860229092	Quantified
Q08879	Fibulin-1	Fbln1	FUNCTION: Incorporated into fibronectin-con	Fibulin-1 OS=Mus musculu	9.65	7	7	124	705	77.98058055	5.160644531	1.005052374	119	7.326011603	1.004451893	120	6.681953331	0.998346893	119	5.395404674	1.006847768	120	6.814842347	0.963538251	115	10.39623968	1.012685882	119	5.98263784	Quantified
P68372	Tubulin beta-4B chain	Tubb4b	FUNCTION: Tubulin is the major constituent	Tubulin beta-4B chain OS=	51.01	1	17	78	445	49.79900427	4.894042969	0.993834014	4	4.043592892	1.004710644	4	3.45660253	1.004259928	4	10.84678837	1.003929938	4	7.228168223	0.9628213	4	9.084243054	1.01542793	4	13.16925174	Quantified
Q61704	Inter-alpha-trypsin inhibitor heavy chain H3	Itih3	FUNCTION: May act as a carrier of hyalurona	Inter-alpha-trypsin inhibitor	10.24	9	9	92	889	99.29568162	6.049316406	1.003739327	92	3.860979862	1.032522857	92	5.783306487	1.014867577	92	3.890199128	0.951103982	92	5.81403206	0.994879418	92	6.521849285	1.018483477	92	5.958163759	Quantified
P68369	Tubulin alpha-1A chain	Tuba1a	FUNCTION: Tubulin is the major constituent	Tubulin alpha-1A chain OS	53.22	5	18	60	451	50.10361194	5.059082031	1.00648857	23	6.313477933	1.010004775	23	5.121967988	1.061645091	23	6.959405305	0.990285026	23	4.501375918	0.940530057	23	6.911779843	0.971750551	23	4.484987921	Quantified
P19221	Prothrombin	F2	FUNCTION: Thrombin, which cleaves bonds	Prothrombin OS=Mus musc	7.93	6	7	76	618	70.22365081	6.430175781	0.994228855	73	3.504556833	1.041719218	73	4.490265593	0.985206135	73	5.767217596	0.969496549	73	6.002552158	0.974860881	73	7.308086314	1.013665254	73	5.167629642	Quantified
P43277	Histone H1.3	H1f3	FUNCTION: Histone H1 protein binds to linke	Histone H1.3 OS=Mus mus	52.04	2	18	83	221	22.08612715	11.03369141	0.611268356	4	9.388384533	0.615002113	4	19.21820007	0.753691078	4	21.05684388	1.151231366	4	6.647568123	1.858662882	4	7.75087725	1.381686795	4	27.89262086	Quantified
P15864	Histone H1.2	H1f2	FUNCTION: Histone H1 protein binds to linke	Histone H1.2 OS=Mus mus	50	4	15	79	212	21.25372322	11.00439453	0.78692104	12	16.82412658	0.754840758	12	35.80456481	0.87079175	12	11.68949356	1.138421536	12	32.29737119	1.570831821	12	24.57878506	1.084040701	12	21.98756109	Quantified
Q00623	Apolipoprotein A-I	Apoa1	FUNCTION: Participates in the reverse transp	Apolipoprotein A-I OS=Mus	7.2	4	5	96	264	30.59664433	5.731933594	0.870637278	84	7.963827573	1.09396936	84	8.695525818	0.883612138	84	5.684946889	0.863909597	84	10.6537324	1.264842701	84	12.30345204	1.122677206	84	10.24626229	Quantified
P14733	Lamin-B1	Lmnb1	FUNCTION: Lamins are components of the n	Lamin-B1 OS=Mus musculu	54.25	32	38	99	588	66.74476308	5.160644531	0.971712662	81	5.045550023	0.900999866	81	7.348696077	0.954316694	81	5.797432356	1.144837336	81	10.67526516	1.153105118	81	8.843766404	0.948195887	81	7.574383709	Quantified
P68368	Tubulin alpha-4A chain	Tuba4a	FUNCTION: Tubulin is the major constituent	Tubulin alpha-4A chain OS	53.57	5	18	59	448	49.89237157	5.059082031	1.028038666	22	7.983989183	1.006721197	22	3.488490847	0.986985356	22	9.832624866	0.982496599	22	6.574872853	0.986843986	22	12.30375369	1.002492392	22	3.927547931	Quantified
P09405	Nucleolin	Ncl	FUNCTION: Nucleolin is the major nucleolar	Nucleolin OS=Mus musculu	39.46	31	31	101	707	76.6767704	4.754394531	1.0518863	101	4.223085733	1.051525066	101	4.691976114	1.084832828	101	5.41434597	0.936010225	101	5.383272777	0.883528912	101	8.931435512	0.946599376	101	4.981591984	Quantified
P43274	Histone H1.4	H1f4	FUNCTION: Histone H1 protein binds to linke	Histone H1.4 OS=Mus mus	48.86	5	17	82	219	21.96401758	11.10693359	0.732364917	20	25.03014218	0.661970847	20	29.39224851	0.785782235	20	20.10475798	1.343234621	20	23.30925603	1.593107874	20	16.71620345	1.133766187	20	13.78655092	Quantified
P26041	Moesin	Msn	FUNCTION: Ezrin-radixin-moesin (ERM) fami	Moesin OS=Mus musculus	53.9	32	41	132	577	67.72488989	6.595214844	1.00701966	101	4.884131382	1.059115473	101	6.113349968	1.072158848	101	7.329851225	0.928337897	101	8.01520295	0.970624214	101	7.74386998	0.964193877	101	7.781133134	Quantified
P99024	Tubulin beta-5 chain	Tubb5	FUNCTION: Tubulin is the major constituent	Tubulin beta-5 chain OS=M	47.3	3	16	74	444	49.63897361	4.894042969	1.027464562	7	3.940003308	1.017758891	7	6.430157638	1.017547369	7	7.904391545	1.009606366	7	1.518420013	0.980530875	7	4.591791084	0.990276437	7	4.660277552	Quantified
P27661	Histone H2AX	H2ax	FUNCTION: Variant histone H2A which repla	Histone H2AX OS=Mus mu	69.93	6	11	91	143	15.13343658	10.74072266	0.688867445	15	15.19381962	0.662678394	15	21.67967864	0.772144514	15	8.068020117	1.540539119	15	13.01326931	1.733396455	15	11.09507105	1.052429851	15	4.878255668	Quantified
Q6GQT1	Alpha-2-macroglobulin-P	A2m	FUNCTION: Is able to inhibit all four classes	Alpha-2-macroglobulin-P O	3.26	3	5	154	1474	164.2482023	6.609863281	1.005746861	93	4.185469924	0.947806392	93	5.495763476	0.992254678	93	5.284559541	1.073991925	93	6.333059231	0.947719459	93	5.79240896	1.038179196	93	5.811879391	Quantified
P48678	Prelamin-A/C	Lmna	FUNCTION: Lamins are components of the n	Prelamin-A/C OS=Mus mus	41.2	29	31	91	665	74.19270157	6.976074219	0.944613072	80	7.27964991	0.855788875	80	12.87580844	0.887696112	80	6.905612414	1.205561623	80	12.25158031	1.193447543	80	11.57973932	0.997691291	80	6.519268339	Quantified
Q9D0F9	Phosphoglucomutase-1	Pgm1	FUNCTION: This enzyme participates in both	Phosphoglucomutase-1 OS	32.03	18	18	53	562	61.37959864	6.565917969	1.004626321	52	3.791825442	1.006150531	52	5.149523258	1.000325998	52	4.918264244	0.987289575	52	8.38693111	0.942706817	52	7.452972868	1.047045394	52	7.261686019	Quantified
Q61292	Laminin subunit beta-2	Lamb2	FUNCTION: Binding to cells via a high affinity	Laminin subunit beta-2 OS=	20.84	30	30	71	1799	196.4514774	6.668457031	0.994550182	71	5.397344078	0.9722228	71	5.037939415	0.978451218	71	8.311512362	1.029214389	71	8.156516942	1.024947232	71	7.150473207	1.004518501	71	4.632201072	Quantified
Q7TMM9	Tubulin beta-2A chain	Tubb2a	FUNCTION: Tubulin is the major constituent	Tubulin beta-2A chain OS=	38.88	1	14	66	445	49.87495877	4.894042969	1.018001464	1		1.013130417	1		1.074587509	1		1.023844197	1		0.962969306	1		0.900841168	1		Quantified
Q62009	Periostin	Postn	FUNCTION: Induces cell attachment and spre	Periostin OS=Mus musculu	18.02	16	16	80	838	93.08481958	7.532714844	1.021442806	80	6.046528676	1.036896096	80	4.481699366	1.01249238	80	6.604220639	0.96516294	80	5.995275956	0.929828419	80	6.600377383	0.998844282	80	5.648279909	Quantified
O35490	Betaine--homocysteine S-methyltransferase 1	Bhmt	FUNCTION: Involved in the regulation of hom	Betaine--homocysteine S-m	33.42	10	10	69	407	44.99188016	7.898925781	0.965113561	69	5.155174378	1.05860116	69	6.461829016	1.011651499	69	6.660441888	0.947436233	69	8.022269506	1.009737941	69	9.821463672	1.017320071	69	5.737929884	Quantified
Q9D6F9	Tubulin beta-4A chain	Tubb4a	FUNCTION: Tubulin is the major constituent	Tubulin beta-4A chain OS=	50.23	4	16	54	444	49.55390311	4.881347656	0.985941539	7	2.8384981	0.940549304	7	3.909827825	1.009057048	7	10.30991765	1.015742649	7	8.948233202	1.049396655	7	9.417855792	1.031238778	7	4.460379166	Quantified
P68433	Histone H3.1	H3c1	FUNCTION: Core component of nucleosome.	Histone H3.1 OS=Mus mus	31.62	2	7	134	136	15.39448411	11.12158203	0.777517557	11	17.71172773	0.791979762	11	20.19039819	0.848366576	11	10.07025808	1.214058082	11	18.19615382	1.412340244	11	3.729057778	1.167015057	11	8.070811965	Quantified
Q02053	Ubiquitin-like modifier-activating enzyme 1	Uba1	FUNCTION: Catalyzes the first step in ubiquit	Ubiquitin-like modifier-activ	24.29	23	23	54	1058	117.7340139	5.655761719	1.042578596	54	4.644859244	1.015690777	54	5.089700167	1.060645005	54	10.26675568	0.933117742	54	7.609086334	0.936951505	54	10.42207958	0.976922318	54	5.999979295	Quantified
P61161	Actin-related protein 2	Actr2	FUNCTION: ATP-binding component of the A	Actin-related protein 2 OS=	24.11	7	8	38	394	44.73223564	6.741699219	1.048613567	32	6.727932691	1.035420705	32	5.840925534	0.994420936	32	7.940708493	0.962702816	32	9.474514548	0.943268569	32	20.11252196	1.013515385	32	6.767015258	Quantified
P40124	Adenylyl cyclase-associated protein 1	Cap1	FUNCTION: Directly regulates filament dynan	Adenylyl cyclase-associate	43.67	20	20	60	474	51.53244297	7.518066406	1.021871703	54	5.250979339	1.019029698	54	5.052421857	1.047795629	54	7.403473089	0.979645511	54	5.592555842	0.933651743	54	9.419141856	0.980468994	54	5.842615661	Quantified
Q7TPR4	Alpha-actinin-1	Actn1	FUNCTION: F-actin cross-linking protein whic	Alpha-actinin-1 OS=Mus m	33.52	17	28	77	892	103.0035917	5.376464844	1.01732199	35	2.626402727	1.010787522	35	5.19997306	0.988756193	35	5.621723135	1.00827573	35	4.337291298	0.94437911	35	5.44096878	1.009731213	35	5.327795469	Quantified
P16858	Glyceraldehyde-3-phosphate dehydrogenase	Gapdh	FUNCTION: Has both glyceraldehyde-3-phos	Glyceraldehyde-3-phosphat	45.95	12	12	43	333	35.78721433	8.250488281	1.02543568	43	5.1699047	1.047700825	43	5.331013665	1.046251225	43	8.98605093	0.9581344	43	5.727179575	0.945297314	43	9.130732184	0.9730513	43	7.325802491	Quantified
Q61696	Heat shock 70 kDa protein 1A	Hspa1a	FUNCTION: Molecular chaperone implicated	Heat shock 70 kDa protein	25.43	5	14	61	641	70.03595415	5.719238281	1.008907446	20	6.324006649	1.013325824	20	6.702639742	0.964397516	20	7.236943962	1.035488935	20	8.349449699	1.022488238	20	8.238149188	1.01744754	20	7.310366157	Quantified
P02301	Histone H3.3C	H3f3c	FUNCTION: Core component of nucleosome.	Histone H3.3C OS=Mus mu	31.62	2	7	126	136	15.30549708	11.13623047	0.797609612	3	3.683407074	0.868115106	3	6.008514274	0.827130936	3	6.490516154	1.119923716	3	6.419585904	1.346806376	3	4.840056652	1.167216437	3	7.84405784	Quantified
Q9JKF1	Ras GTPase-activating-like protein IQGAP1	Iqgap1	FUNCTION: Plays a crucial role in regulating	Ras GTPase-activating-like	25.77	32	34	59	1657	188.6242524	6.480957031	1.04217719	55	3.51215579	1.031124897	55	4.950277681	1.041252414	55	6.225067224	0.965683648	55	5.291940179	0.965607776	55	6.878133986	0.967321568	55	5.333078008	Quantified
Q91ZX7	Prolow-density lipoprotein receptor-related protein 1	Lrp1	FUNCTION: Endocytic receptor involved in e	Prolow-density lipoprotein r	11.82	49	49	88	4545	504.4107055	5.363769531	0.986075811	88	4.784960601	0.984428227	88	5.256210977	0.996499695	88	7.668543306	1.012858241	88	7.727198468	1.022423604	88	9.266049376	1.007111094	88	5.07815278	Quantified
P17156	Heat shock-related 70 kDa protein 2	Hspa2	FUNCTION: Molecular chaperone implicated	Heat shock-related 70 kDa	21.01	4	13	58	633	69.59869094	5.668457031	0.973859375	4	3.065219949	0.964365532	4	2.571627909	0.96531934	4	7.382767339	1.020306304	4	4.801300083	1.055170318	4	3.495470114	1.06013161	4	6.005910948	Quantified
P40142	Transketolase	Tkt	FUNCTION: Catalyzes the transfer of a two-c	Transketolase OS=Mus mu	29.53	14	14	40	623	67.58758479	7.503417969	1.016862435	40	5.124471814	1.054484821	40	7.091441909	1.024918232	40	7.481249791	0.928125947	40	10.38229936	0.994012891	40	8.80932903	0.973744923	40	5.880311045	Quantified
P07724	Albumin	Alb	FUNCTION: Binds water, Ca(2+), Na(+), K(+)	Albumin OS=Mus musculus	13.32	8	8	99	608	68.64771403	6.074707031	0.956651191	98	10.6706886	0.99191139	98	11.4803843	0.980818795	98	13.88293716	0.955506208	97	10.41842832	1.074412254	94	17.79915158	1.029421077	98	11.12528552	Quantified
P63101	14-3-3 protein zeta/delta	Ywhaz	FUNCTION: Adapter protein implicated in the	14-3-3 protein zeta/delta O	48.98	8	11	41	245	27.7537453	4.792480469	0.97453929	25	6.495906465	0.934151473	25	5.576710386	1.071763702	25	9.019276082	1.010128815	25	7.541618815	1.066149135	25	6.055902503	0.971540492	25	4.258031559	Quantified
P61979	Heterogeneous nuclear ribonucleoprotein K	Hnrnpk	FUNCTION: One of the major pre-mRNA-bind	Heterogeneous nuclear ribo	36.93	12	12	35	463	50.94440521	5.541503906	1.039890903	35	7.259686075	1.056934171	35	4.735751607	1.119048765	35	4.792030988	0.92165457	35	4.694702868	0.91562888	35	10.6297732	0.936563164	35	6.186273987	Quantified
Q60605	Myosin light polypeptide 6	Myl6	FUNCTION: Regulatory light chain of myosin.	Myosin light polypeptide 6	31.79	5	5	18	151	16.91913397	4.652832031	0.989194088	18	4.67882754	0.941654564	18	6.178394381	1.118842183	18	12.4544422	1.002602488	18	7.039013966	1.053981895	18	11.13182343	0.952901612	18	7.13774401	Quantified
P11031	Activated RNA polymerase II transcriptional coactiva	Sub1	FUNCTION: General coactivator that function	Activated RNA polymerase	42.52	6	6	31	127	14.41841934	9.598144531	1.00099909	31	5.075001277	0.98013099	31	8.104423074	1.003294335	31	4.74644366	0.998415503	31	7.59715801	1.017101063	31	8.949656882	0.999902912	31	4.099899533	Quantified
O54724	Caveolae-associated protein 1	Cavin1	FUNCTION: Plays an important role in caveo	Caveolae-associated protei	31.89	11	11	34	392	43.92701734	5.516113281	1.027709534	34	4.553060287	1.022340545	34	7.118377608	0.998479917	34	5.366264715	0.969350063	34	8.46556116	0.992123337	34	11.40906461	0.98648233	34	9.507166104	Quantified
O88783	Coagulation factor V	F5	FUNCTION: Central regulator of hemostasis.	Coagulation factor V OS=M	3.39	10	10	55	2183	247.0762171	6.049316406	1.017769361	55	4.480706829	0.992580486	55	5.606456925	0.992651825	55	6.398856957	1.009621404	55	9.171060035	0.941688453	55	10.72038309	1.00712832	55	4.491126096	Quantified
P20029	Endoplasmic reticulum chaperone BiP	Hspa5	FUNCTION: Endoplasmic reticulum chaperon	Endoplasmic reticulum cha	45.04	23	26	60	655	72.37746644	5.160644531	1.00421869	42	3.995052376	1.001856453	42	5.21873485	0.981596264	42	5.804836861	0.994636253	42	7.960182252	1.009300717	42	8.403723684	1.00925879	42	4.793008311	Quantified
P06151	L-lactate dehydrogenase A chain	Ldha		L-lactate dehydrogenase A	32.53	9	11	36	332	36.47522314	7.737792969	1.023265604	27	4.533659836	1.021519921	27	3.593119419	1.072601246	27	7.757952421	0.943102426	27	9.448807475	0.928536667	27	9.257316448	0.985003543	27	6.517966565	Quantified
Q80X90	Filamin-B	Flnb	FUNCTION: Connects cell membrane constit	Filamin-B OS=Mus musculu	14.68	27	34	56	2602	277.6507614	5.706542969	1.024214677	47	4.15420862	1.008619829	47	4.028411225	0.982087448	47	8.820327695	1.004330308	47	7.5250292	0.97339538	47	10.58322946	1.004992405	47	6.245580743	Quantified
O08553	Dihydropyrimidinase-related protein 2	Dpysl2	FUNCTION: Plays a role in neuronal develop	Dihydropyrimidinase-relate	43.01	14	17	39	572	62.23859391	6.379394531	1.013365718	34	5.763640805	1.026976788	34	5.350322015	1.005031387	34	10.2711934	0.998902817	34	3.700017237	0.985750631	34	7.863705148	0.979876738	34	7.531708892	Quantified
P26043	Radixin	Rdx	FUNCTION: Probably plays a crucial role in t	Radixin OS=Mus musculus	38.42	12	22	57	583	68.50033179	6.201660156	0.987065921	24	3.335961208	1.025275587	24	6.901594447	0.996027185	24	6.494500209	0.985719243	24	6.642958784	1.028658654	24	5.788608268	1.008688067	24	3.04008635	Quantified
P82198	Transforming growth factor-beta-induced protein ig-h	Tgfbi	FUNCTION: Plays a role in cell adhesion (Pu	Transforming growth factor-	18.16	11	11	35	683	74.54966381	7.063964844	0.987676512	35	5.732889585	1.046506987	35	6.793752541	1.013793742	35	5.924563183	0.962557992	35	6.408703108	0.990984703	35	7.255397001	0.992330622	35	6.989371583	Quantified
O89053	Coronin-1A	Coro1a	FUNCTION: May be a crucial component of t	Coronin-1A OS=Mus muscu	37.53	18	18	48	461	50.95691701	6.480957031	1.054981378	48	6.623187201	1.068936915	48	7.411730455	1.138537291	48	12.40564444	0.90831628	48	7.819879151	0.896142041	48	12.82730612	0.899363753	48	6.56130895	Quantified
Q5SX39	Myosin-4	Myh4	FUNCTION: Muscle contraction.	Myosin-4 OS=Mus musculu	9.59	1	19	37	1939	222.719945	5.744628906	0.600384258	1		0.618227523	1		0.52331262	1		1.657100194	1		1.746573863	1		1.282663218	1		Quantified
Q9JLJ2	4-trimethylaminobutyraldehyde dehydrogenase	Aldh9a1	FUNCTION: Converts gamma-trimethylamino	4-trimethylaminobutyraldeh	17.61	8	8	38	494	53.48005778	6.976074219	1.016113134	37	4.838564423	0.997090241	37	5.106639063	0.997196059	37	4.260473523	0.993727561	37	4.283752552	0.976926208	37	6.28932235	0.999914362	37	6.322194078	Quantified
P08113	Endoplasmin	Hsp90b1	FUNCTION: Molecular chaperone that functio	Endoplasmin OS=Mus mus	21.7	17	19	53	802	92.41826033	4.817871094	0.982264264	43	5.528856877	0.994917569	43	6.368897182	0.996840035	43	6.277462552	0.993248079	43	5.632807772	1.040301308	43	7.025124002	1.000259307	43	5.761135033	Quantified
P16627	Heat shock 70 kDa protein 1-like	Hspa1l	FUNCTION: Molecular chaperone implicated	Heat shock 70 kDa protein	21.06	2	11	43	641	70.59315973	6.239746094	0.974185722	3	1.516356183	1.022998813	3	4.00216294	0.994188301	3	0.179569521	1.00153647	3	1.762927152	1.04049826	3	6.051631241	1.012347283	3	1.779222405	Quantified
Q99JY9	Actin-related protein 3	Actr3	FUNCTION: ATP-binding component of the A	Actin-related protein 3 OS=	46.17	15	15	46	418	47.32696787	5.884277344	1.01143404	46	5.329573528	1.006043611	46	7.365626425	1.047023535	46	9.297437763	0.959788791	46	8.420305216	0.967303058	46	15.22581414	0.973930809	46	5.675569686	Quantified
P12960	Contactin-1	Cntn1	FUNCTION: Contactins mediate cell surface i	Contactin-1 OS=Mus musc	20.78	20	20	56	1020	113.3169648	6.163574219	0.999074718	56	4.038051585	1.014281319	56	5.47559356	1.015999811	56	4.323106855	0.972075602	56	6.8687148	0.985168571	56	6.921870045	1.009226466	56	5.220611369	Quantified
P10126	Elongation factor 1-alpha 1	Eef1a1	FUNCTION: This protein promotes the GTP-d	Elongation factor 1-alpha 1	36.8	9	17	55	462	50.08209985	9.012207031	1.019799964	31	2.930588227	1.033229284	31	5.189989699	0.998054182	31	5.109491398	0.986138207	31	6.721138525	0.947795658	31	9.637703158	0.992706234	31	4.28476331	Quantified
Q64374	Regucalcin	Rgn	FUNCTION: Gluconolactonase with low activi	Regucalcin OS=Mus muscu	18.39	5	5	27	299	33.385489	5.338378906	0.979459469	27	4.673354667	1.01140494	27	4.493024745	1.06510182	27	8.407533969	0.973663189	27	5.412669704	1.010335675	27	6.535062869	0.978947027	27	4.790794836	Quantified
Q61879	Myosin-10	Myh10	FUNCTION: Involved with LARP6 in the stabi	Myosin-10 OS=Mus muscu	10.02	8	20	39	1976	228.8549648	5.541503906	1.003464646	12	6.011830233	0.988433623	12	11.71426182	0.954906613	12	3.271419854	1.057873526	12	14.51847295	1.050429039	12	18.55245403	0.971369694	12	10.00217403	Quantified
P05064	Fructose-bisphosphate aldolase A	Aldoa	FUNCTION: Plays a key role in glycolysis an	Fructose-bisphosphate aldo	43.96	13	16	46	364	39.33127641	8.089355469	0.992617921	38	4.359342929	1.030365773	38	6.029708109	1.002437779	38	12.80590727	0.996385644	38	9.250300042	1.052050232	38	9.832860398	0.975226602	38	5.098722554	Quantified
Q9Z1R3	Apolipoprotein M	Apom	FUNCTION: Probably involved in lipid transp	Apolipoprotein M OS=Mus	20	4	4	37	190	21.2594408	6.521972656	0.969525001	37	5.537355751	1.063360483	37	5.312816158	1.023086908	37	5.516335275	0.929537889	37	7.300543486	0.989506625	37	9.490700608	1.005238461	37	4.791144624	Quantified
P18760	Cofilin-1	Cfl1	FUNCTION: Binds to F-actin and exhibits pH-	Cofilin-1 OS=Mus musculus	53.01	7	8	35	166	18.54768013	8.089355469	0.966310922	25	4.829188236	1.049198726	25	7.162584557	1.07744654	25	13.45303295	0.952651431	25	11.95442069	0.941598367	25	8.895093037	0.942784818	25	6.018917859	Quantified
P31001	Desmin	Des	FUNCTION: Muscle-specific type III intermed	Desmin OS=Mus musculus	23.67	7	13	46	469	53.46503963	5.274902344	1.036277572	9	2.080954231	0.95820159	9	3.527198316	0.987944058	9	6.999546415	1.078477966	9	10.0104482	1.019776935	9	8.378132725	0.980577733	9	8.938964704	Quantified
Q9DCD0	6-phosphogluconate dehydrogenase, decarboxylating	Pgd	FUNCTION: Catalyzes the oxidative decarbo	6-phosphogluconate dehyd	28.36	11	12	36	483	53.21306504	7.225097656	1.012131655	35	5.099871167	1.028465533	35	4.399819239	1.000389802	35	8.01936752	0.971175392	35	7.324945658	0.969465876	35	6.767191376	0.990021257	35	5.317612624	Quantified
Q6ZQ38	Cullin-associated NEDD8-dissociated protein 1	Cand1	FUNCTION: Key assembly factor of SCF (SK	Cullin-associated NEDD8-d	17.48	22	22	51	1230	136.2446147	5.782714844	1.024565853	51	4.035723011	0.999156809	51	5.3038736	1.0313846	51	6.459452459	0.998876075	51	5.781976201	0.944713139	51	6.146334571	1.004631404	51	4.893148423	Quantified
Q60710	Deoxynucleoside triphosphate triphosphohydrolase S	Samhd1	FUNCTION: [Isoform 1]: Protein that acts both	Deoxynucleoside triphosph	26.44	15	15	29	658	75.84484801	7.928222656	1.038681017	29	4.2177925	1.047337226	29	4.265492184	1.037709353	29	7.677177201	0.961952884	29	5.983629239	0.917312961	29	10.81020934	0.943802667	29	4.888485031	Quantified
P11087	Collagen alpha-1(I) chain	Col1a1	FUNCTION: Type I collagen is a member of g	Collagen alpha-1(I) chain O	9.15	14	14	45	1453	137.94799	5.846191406	0.984632956	45	3.935798766	1.011987365	45	11.67974284	1.047254886	45	9.057645429	0.965332748	45	14.19599763	1.049710478	45	5.465770938	0.941593968	45	9.391727147	Quantified
P21619	Lamin-B2	Lmnb2	FUNCTION: Lamins are components of the n	Lamin-B2 OS=Mus musculu	45.3	22	28	57	596	67.27732022	5.503417969	1.000514282	39	5.665353215	0.967690602	39	6.275572942	0.913963488	39	8.102752448	1.071722529	39	8.661037247	1.100504782	39	7.855908625	1.019688043	39	5.613445881	Quantified
Q61838	Pregnancy zone protein	Pzp	FUNCTION: Is able to inhibit all four classes	Pregnancy zone protein OS	2.07	3	4	74	1495	165.7478009	6.683105469	1.008163645	17	4.38863934	0.995624674	17	4.969861733	0.962599916	17	9.19651091	1.007495778	17	7.337634728	0.928451227	17	9.626868311	1.056174816	17	5.513100962	Quantified
Q61316	Heat shock 70 kDa protein 4	Hspa4		Heat shock 70 kDa protein	26.99	22	22	52	841	94.07330722	5.236816406	1.040365756	52	4.107278407	0.999269797	52	6.512216351	1.030889927	52	8.99174612	0.976833332	52	5.944783896	0.965707056	52	11.87469666	0.988477055	52	6.998908395	Quantified
P80314	T-complex protein 1 subunit beta	Cct2	FUNCTION: Component of the chaperonin-co	T-complex protein 1 subuni	20.75	9	9	24	535	57.44111094	6.404785156	1.036978277	24	4.267038529	1.0379553	24	7.49025512	1.004284296	24	8.420118812	0.99642027	24	6.413114863	0.943605672	24	10.68199264	0.961162631	24	8.552543804	Quantified
Q07113	Cation-independent mannose-6-phosphate receptor	Igf2r	FUNCTION: Acts as a positive regulator of T-	Cation-independent manno	4.19	11	11	33	2483	273.6392104	5.706542969	1.013205944	33	5.179440404	1.017545034	33	3.109464328	1.014757586	33	7.294229791	1.011896851	33	6.082702301	0.922626255	33	7.319575853	0.989391889	33	3.545912968	Quantified
P62962	Profilin-1	Pfn1	FUNCTION: Binds to actin and affects the str	Profilin-1 OS=Mus musculu	61.43	7	7	20	140	14.9475114	8.279785156	0.995087938	20	5.090235377	0.991135013	20	3.354609242	1.028584075	20	6.580485075	1.009930267	20	7.28448909	0.965973372	20	7.879026321	1.014378021	20	5.291046303	Quantified
Q9WVK4	EH domain-containing protein 1	Ehd1	FUNCTION: ATP- and membrane-binding pro	EH domain-containing prote	38.2	10	17	32	534	60.56463207	6.829589844	1.004264739	17	2.875131403	1.051362327	17	5.121071217	0.992345635	17	4.613172457	0.976033467	17	6.036546259	0.971059974	17	5.292643021	0.980014482	17	5.147286582	Quantified
P26040	Ezrin	Ezr	FUNCTION: Probably involved in connections	Ezrin OS=Mus musculus O	35.84	14	23	49	586	69.36363205	6.100097656	1.018287675	28	3.657518463	1.062461233	28	5.729416705	1.015817427	28	5.031214996	0.968304847	28	8.950537585	0.948858937	28	10.7753901	0.974797324	28	8.773905415	Quantified
Q8K4G1	Latent-transforming growth factor beta-binding protei	Ltbp4	FUNCTION: Key regulator of transforming gro	Latent-transforming growth	7.26	11	11	26	1666	178.5229982	5.465332031	0.977046791	26	7.363309092	1.033543587	26	8.25828941	1.054112479	26	19.64900799	0.935678946	26	11.33608183	0.905169733	26	11.53012324	0.966522449	26	5.796470042	Quantified
P43276	Histone H1.5	H1f5	FUNCTION: Histone H1 protein binds to linke	Histone H1.5 OS=Mus mus	40.36	9	13	36	223	22.56243691	10.91650391	0.769251474	22	22.34250822	0.72923691	22	15.94284317	0.856699663	22	10.45229243	1.279192964	22	16.76545707	1.452945464	22	12.60521648	1.176883905	22	8.654314822	Quantified
P68254	14-3-3 protein theta	Ywhaq	FUNCTION: Adapter protein implicated in the	14-3-3 protein theta OS=Mu	43.67	5	9	41	245	27.76078364	4.779785156	0.943153013	20	5.651515843	0.926518016	20	11.01960334	0.998893996	20	10.38129413	1.022151063	20	8.488811349	1.055311126	20	12.69990751	1.030553799	20	4.432118016	Quantified
Q9QZQ8	Core histone macro-H2A.1	Macroh2a1	FUNCTION: Variant histone H2A which repla	Core histone macro-H2A.1	36.56	9	11	26	372	39.71048919	9.803222656	0.927490517	22	8.009860025	0.947412569	22	6.29441275	0.915871733	22	6.031807419	1.074494742	22	5.500047318	1.06912098	22	4.24878311	1.076876266	22	7.552165227	Quantified
P02468	Laminin subunit gamma-1	Lamc1	FUNCTION: Binding to cells via a high affinity	Laminin subunit gamma-1	18.29	28	28	57	1607	177.1847364	5.211425781	1.013835098	57	4.084775942	0.967125535	57	4.624535315	0.995796822	57	5.246892934	1.023197013	57	4.245811709	1.019103209	57	5.708695926	1.000320743	57	5.452417803	Quantified
Q6PDM2	Serine/arginine-rich splicing factor 1	Srsf1	FUNCTION: Plays a role in preventing exon s	Serine/arginine-rich splicing	43.15	12	12	38	248	27.72781623	10.35986328	1.004287451	38	4.745723117	1.072138876	38	6.177294731	1.027418966	38	11.91765135	0.963655359	38	5.976084474	0.946909843	38	16.30555723	1.001375758	38	6.650513225	Quantified
P42932	T-complex protein 1 subunit theta	Cct8	FUNCTION: Component of the chaperonin-co	T-complex protein 1 subuni	26.09	13	13	30	548	59.51751413	5.617675781	1.038492462	30	3.094087084	1.003506566	30	3.619491934	1.024257911	30	4.381221343	0.98561839	30	7.118158142	0.967263836	30	6.753110808	0.980434334	30	5.812023313	Quantified
P47753	F-actin-capping protein subunit alpha-1	Capza1	FUNCTION: F-actin-capping proteins bind in	F-actin-capping protein sub	41.26	7	8	19	286	32.91930822	5.554199219	1.031012501	18	4.12642227	1.038874136	18	2.52731666	1.03218992	18	5.624571772	0.959535856	18	7.241238806	0.945048317	18	7.142091033	0.976056204	18	6.804003109	Quantified

P16125	L-lactate dehydrogenase B chain	Ldhb		L-lactate dehydrogenase B	14.97	4	6	18	334	36.54903625	6.049316406	1.011162983	9	4.141803998	1.044244501	9	6.867809793	0.988845767	9	3.313346996	0.980824965	9	5.47726548	0.978564639	9	2.053204305	0.97958592	9	4.506371891	Quantified
P43275	Histone H1.1	H1f1	FUNCTION: Histone H1 protein binds to linke	Histone H1.1 OS=Mus mus	36.62	5	8	30	213	21.77190784	10.93115234	0.857367763	15	4.391293969	0.770662959	15	8.403935485	0.972270433	15	2.090716656	1.265223327	15	12.67090973	1.366192653	15	8.159337558	0.945440014	15	4.334298707	Quantified
Q9Z1Q5	Chloride intracellular channel protein 1	Clic1	FUNCTION: Can insert into membranes and f	Chloride intracellular chann	39.42	7	7	18	241	26.99573813	5.173339844	1.035307187	18	5.978566864	1.019739774	18	6.537985468	1.077044575	18	8.25228527	0.93113429	18	6.994169849	0.991682193	18	9.075347411	0.950643364	18	5.71189684	Quantified
P01029	Complement C4-B	C4b	FUNCTION: Non-enzymatic component of C3	Complement C4-B OS=Mus	4.32	7	7	38	1738	192.7936031	7.532714844	1.007439901	38	3.94329658	0.997882794	38	3.519581042	1.000777341	38	4.394689551	1.033709066	38	3.404045352	0.946908796	38	6.020333899	1.012181369	38	5.347115117	Quantified
P01942	Hemoglobin subunit alpha	Hba	FUNCTION: Involved in oxygen transport fron	Hemoglobin subunit alpha (	21.83	4	4	60	142	15.0757613	8.221191406	0.978473196	60	6.397144446	0.980346058	60	5.508103391	0.993088725	60	9.233598553	1.006492382	60	5.285149737	1.123527637	60	9.166297624	0.981505452	60	10.72639175	Quantified
Q60847	Collagen alpha-1(XII) chain	Col12a1	FUNCTION: Type XII collagen interacts with t	Collagen alpha-1(XII) chain	7.21	20	20	47	3120	340.0041585	5.643066406	1.000908716	47	5.108279879	1.008564804	47	5.046970823	0.979052812	47	10.08332944	0.999716743	47	7.0457146	0.971464729	47	11.59471439	1.002700466	47	5.264403696	Quantified
P0C0S6	Histone H2A.Z	H2az1	FUNCTION: Variant histone H2A which repla	Histone H2A.Z OS=Mus mu	31.25	3	5	33	128	13.54455269	10.57958984	0.78842783	18	11.39081248	0.714009205	18	15.21988682	0.791060859	18	10.41024668	1.463547518	18	10.68779422	1.332312846	18	10.23049149	1.105575606	18	6.767754343	Quantified
Q9EQP2	EH domain-containing protein 4	Ehd4	FUNCTION: ATP- and membrane-binding pro	EH domain-containing prote	24.4	9	11	23	541	61.44145019	6.756347656	1.02910469	19	4.091660887	1.015169102	19	4.353024973	1.104678355	19	5.431455164	0.96628771	19	5.646417964	0.967344363	19	7.491282147	0.922309509	19	4.580581125	Quantified
Q61233	Plastin-2	Lcp1	FUNCTION: Actin-binding protein. Plays a rol	Plastin-2 OS=Mus musculu	32.38	14	19	36	627	70.10478998	5.325683594	1.042236116	28	4.514698406	1.016695019	28	4.781028563	1.041657656	28	4.12437051	0.957444979	28	4.172661778	0.960945218	28	5.754608634	0.96314383	28	3.936705859	Quantified
P61982	14-3-3 protein gamma	Ywhag	FUNCTION: Adapter protein implicated in the	14-3-3 protein gamma OS=	53.44	7	12	42	247	28.28491393	4.894042969	0.984793466	18	7.976790704	0.983544173	18	5.96110298	0.933669812	18	10.6700099	1.015031122	18	10.76397058	1.088556023	18	11.72791889	1.08121543	18	8.012033701	Quantified
E9Q414	Apolipoprotein B-100	Apob	FUNCTION: Apolipoprotein B is a major prote	Apolipoprotein B-100 OS=M	1.73	10	10	38	4505	509.1129915	6.814941406	0.976214208	38	8.011212342	1.052619521	38	7.008402313	0.975928799	38	7.243638402	1.024279903	38	4.33625258	0.929052459	38	8.87011671	1.029480666	38	5.666027691	Quantified
P0CG49	Polyubiquitin-B	Ubb	FUNCTION: [Ubiquitin]: Exists either covalent	Polyubiquitin-B OS=Mus mu	61.64	5	5	27	305	34.34750008	7.532714844	0.977610989	27	6.838518356	0.996829709	27	5.154450863	0.926853536	27	8.277922878	1.059925191	27	6.075993741	1.023972573	27	5.743241583	1.037773184	27	5.979005742	Quantified
O88342	WD repeat-containing protein 1	Wdr1	FUNCTION: Induces disassembly of actin fila	WD repeat-containing prote	29.7	13	13	41	606	66.3649901	6.595214844	1.013472887	41	4.311330761	1.004632083	41	2.858942835	1.003578535	41	8.399160258	1.013702605	41	5.046749877	0.967065844	41	7.862869071	0.975708375	41	6.152337186	Quantified
Q9CQV8	14-3-3 protein beta/alpha	Ywhab	FUNCTION: Adapter protein implicated in the	14-3-3 protein beta/alpha O	51.22	5	10	38	246	28.06886316	4.830566406	0.983944189	16	5.592965969	0.941136397	16	5.852924853	1.021867825	16	16.04919168	1.005587448	16	9.975262144	1.080625992	16	4.885744588	1.001803993	16	5.901541062	Quantified
Q9EQH3	Vacuolar protein sorting-associated protein 35	Vps35	FUNCTION: Acts as component of the retrom	Vacuolar protein sorting-as	10.68	7	7	21	796	91.65481963	5.439941406	1.036460369	21	6.068992373	1.056577313	21	4.174155611	0.977266072	21	4.990960316	0.958455036	21	6.328256868	0.967342904	21	7.800699625	1.007074952	21	6.014939717	Quantified
Q9ET01	Glycogen phosphorylase, liver form	Pygl	FUNCTION: Phosphorylase is an important a	Glycogen phosphorylase, li	25.41	21	25	55	850	97.4009563	7.093261719	1.021392942	46	4.894973681	1.030872875	46	4.230837452	0.972719113	46	6.122164899	0.99576359	46	4.322876291	1.001363148	46	6.951805519	1.002192196	46	4.645696725	Quantified
P0DP26	Calmodulin-1	Calm1	FUNCTION: Calmodulin mediates the control	Calmodulin-1 OS=Mus mus	40.94	7	7	24	149	16.8268352	4.221191406	1.018590251	24	6.498978635	0.851085843	24	8.442423046	1.080658109	24	4.587192484	1.066754774	24	9.954452836	1.046347255	24	6.440393109	0.913983825	24	9.378635595	Quantified
P13541	Myosin-3	Myh3	FUNCTION: Muscle contraction.	Myosin-3 OS=Mus musculu	9.07	3	16	29	1940	223.6524144	5.808105469	1.000351745	4	1.085323332	0.999995266	4	8.248784375	0.67900862	4	37.88458431	1.202673591	4	14.12706542	1.076458566	4	13.28558989	1.113614091	4	8.729145276	Quantified
P07356	Annexin A2	Anxa2	FUNCTION: Calcium-regulated membrane-bi	Annexin A2 OS=Mus musc	32.15	11	11	22	339	38.65184495	7.693847656	0.994495589	22	5.13198411	1.058188938	22	4.441713188	1.014998948	22	6.648730417	0.964397241	22	6.919942942	0.959405276	22	8.463577368	0.977536214	22	7.347311304	Quantified
Q64487	Receptor-type tyrosine-protein phosphatase delta	Ptprd	FUNCTION: Can bidirectionally induce pre- a	Receptor-type tyrosine-prot	8.89	12	14	23	1912	214.27446	6.551269531	1.007197706	21	4.922802196	1.002012966	21	4.787153963	0.997004113	21	5.47592599	1.022157249	21	6.019185961	0.994692261	21	9.761212071	1.0225656	21	6.713467804	Quantified
Q921I1	Serotransferrin	Tf	FUNCTION: Transferrins are iron binding tran	Serotransferrin OS=Mus mu	4.59	3	4	68	697	76.67372603	7.181152344	0.966040159	54	3.957479897	1.041896511	54	7.20003246	0.998136961	54	4.917835848	0.953470549	54	5.967032403	1.09128481	54	6.843748624	1.01808373	54	7.124146848	Quantified
P08071	Lactotransferrin	Ltf	FUNCTION: Transferrins are iron binding tran	Lactotransferrin OS=Mus m	6.22	5	6	47	707	77.78797537	8.528808594	0.890513523	33	6.536907563	1.041835806	33	6.831576047	1.10798241	33	9.687333852	0.873749879	33	9.080825318	1.033782608	33	9.573654841	0.999310361	33	5.713877943	Quantified
P28481	Collagen alpha-1(II) chain	Col2a1	FUNCTION: Type II collagen is specific for ca	Collagen alpha-1(II) chain (	5.78	6	7	18	1487	141.8863005	6.917480469	0.993979623	17	4.598200814	1.005364568	17	4.408350626	1.014106683	17	3.600132515	1.011267643	17	5.941236934	0.959756805	17	7.763261153	0.971174794	17	6.750801057	Quantified
P47757	F-actin-capping protein subunit beta	Capzb	FUNCTION: F-actin-capping proteins bind in a	F-actin-capping protein sub	29.24	7	7	15	277	31.32575531	5.744628906	1.044798661	9	3.223723468	1.025069511	9	5.429784205	1.028825812	9	3.638667537	0.961586185	9	6.847734677	0.970149486	9	6.36881887	0.967045689	9	5.903307087	Quantified
Q60675	Laminin subunit alpha-2	Lama2	FUNCTION: Binding to cells via a high affinity	Laminin subunit alpha-2 OS	4.36	13	13	23	3118	343.593316	6.087402344	1.002023054	23	4.380676872	0.99191822	23	5.170404987	0.980125919	23	4.833289674	1.052347356	23	6.873595817	0.973602145	23	6.151718458	1.028248934	23	5.39812117	Quantified
P40936	Indolethylamine N-methyltransferase	Inmt	FUNCTION: Catalyzes the N-methylation of tr	Indolethylamine N-methyltra	39.02	9	9	20	264	29.44082069	6.392089844	1.074142694	20	6.454095032	1.094976559	20	5.310927056	1.061112051	20	6.304770996	0.899278366	20	6.97799352	0.928992349	20	5.559191787	0.914410428	20	5.970068311	Quantified
Q6A028	Switch-associated protein 70	Swap70	FUNCTION: Phosphatidylinositol 3,4,5-trisph	Switch-associated protein 7	17.26	10	10	32	585	68.95253316	6.049316406	1.026822724	32	3.624631032	1.024538106	32	8.002954023	1.047385357	32	6.78144209	0.957572387	32	6.852120592	0.959680073	32	9.081611242	0.971816476	32	5.346465567	Quantified
Q8K0E8	Fibrinogen beta chain	Fgb	FUNCTION: Cleaved by the protease thrombi	Fibrinogen beta chain OS=I	16.63	8	8	34	481	54.71769782	7.078613281	1.030743902	34	4.877058268	1.031494289	34	9.060140298	1.017336646	34	8.546443667	0.964847901	34	6.035175975	0.970759233	34	15.54382343	0.994307674	34	7.304710088	Quantified
P09411	Phosphoglycerate kinase 1	Pgk1	FUNCTION: Catalyzes one of the two ATP pr	Phosphoglycerate kinase 1	41.01	10	16	45	417	44.52198868	7.898925781	1.018357509	21	3.46447455	1.043137501	21	2.680060058	1.027683465	21	3.52376286	0.961690002	21	5.59893769	0.928991196	21	6.066527839	0.97626062	21	6.938288608	Quantified
P10630	Eukaryotic initiation factor 4A-II	Eif4a2	FUNCTION: ATP-dependent RNA helicase w	Eukaryotic initiation factor 4	34.15	3	11	22	407	46.37278034	5.478027344	1.016655726	5	3.798439134	1.03787101	5	2.720222794	0.937370193	5	2.186728345	1.026736334	5	3.700139488	0.966701602	5	1.834459416	1.039907681	5	0.821476374	Quantified
Q91V92	ATP-citrate synthase	Acly	FUNCTION: Catalyzes the cleavage of citrate	ATP-citrate synthase OS=M	20.16	18	18	26	1091	119.6512985	7.444824219	1.002514236	26	7.401541694	1.022152422	26	3.4994625	1.002376131	26	8.700342435	0.97732334	26	6.901955411	0.998260496	26	8.194435134	0.986252992	26	8.927524471	Quantified
Q8R0Y6	Cytosolic 10-formyltetrahydrofolate dehydrogenase	Aldh1l1		Cytosolic 10-formyltetrahyd	14.52	14	14	25	902	98.64653023	5.909667969	1.016815371	25	5.154417268	1.016438892	25	2.691911035	0.990566646	25	5.410978049	0.98924634	25	5.048219141	0.949127818	25	5.415395085	1.014775979	25	4.205059339	Quantified
Q71LX4	Talin-2	Tln2	FUNCTION: As a major component of focal a	Talin-2 OS=Mus musculus	5.35	1	16	32	2375	253.4623072	5.795410156	0.944065859	3	1.685848578	0.950799197	3	0.300419366	0.889642603	3	3.550355173	1.118268133	3	0.705584078	1.188622019	3	13.06567287	0.991548612	3	2.920776982	Quantified
P17742	Peptidyl-prolyl cis-trans isomerase A	Ppia	FUNCTION: PPIase that catalyzes the cis-tra	Peptidyl-prolyl cis-trans isor	56.71	9	10	34	164	17.95985222	7.898925781	0.979413359	33	4.037930497	1.054704552	33	4.684022772	1.030461248	33	6.217524975	0.983767204	33	4.84314796	0.986434483	33	10.0545942	0.975591166	33	6.955507278	Quantified
P11983	T-complex protein 1 subunit alpha	Tcp1	FUNCTION: Component of the chaperonin-co	T-complex protein 1 subuni	21.04	12	12	22	556	60.41067273	6.163574219	1.009237234	19	2.415980392	1.016654497	19	5.274092153	1.04441796	19	8.074343262	0.974659367	19	7.536010215	0.985748381	19	7.807018578	0.985996367	19	3.564492798	Quantified
O88207	Collagen alpha-1(V) chain	Col5a1	FUNCTION: Type V collagen is a member of	Collagen alpha-1(V) chain (	3.05	6	6	22	1838	183.5639557	4.982910156	0.977932607	22	6.269771145	1.002849683	22	3.638756128	0.972238237	22	6.935324603	1.017337763	22	5.432735316	0.98844624	22	6.177110305	1.026748164	22	5.95191575	Quantified
P58771	Tropomyosin alpha-1 chain	Tpm1	FUNCTION: Binds to actin filaments in muscl	Tropomyosin alpha-1 chain	52.82	7	18	34	284	32.66067714	4.741699219	1.058005887	12	5.114205928	0.961465533	12	7.100632105	0.816986649	12	11.10375401	1.123757956	12	8.458466185	1.214715582	12	5.986149935	0.986150429	12	5.795520974	Quantified
P60843	Eukaryotic initiation factor 4A-I	Eif4a1	FUNCTION: ATP-dependent RNA helicase w	Eukaryotic initiation factor 4	30.54	4	11	22	406	46.12455781	5.478027344	1.039578699	6	3.979792269	1.034132245	6	3.58321905	0.989203709	6	5.32210615	0.999212759	6	4.359756784	0.965181957	6	5.492957247	0.973671392	6	6.011232046	Quantified
O08638	Myosin-11	Myh11	FUNCTION: Muscle contraction.	Myosin-11 OS=Mus muscu	11.97	10	21	30	1972	226.8876902	5.452636719	0.956610001	11	3.61138467	0.950409656	11	4.989863894	0.959553963	11	4.719660419	1.06847809	11	4.942026516	1.055004521	11	15.21353417	1.019666567	11	7.683197678	Quantified
P32261	Antithrombin-III	Serpinc1	FUNCTION: Most important serine protease i	Antithrombin-III OS=Mus m	18.28	8	8	40	465	51.9705881	6.455566406	1.011584563	40	3.522335688	1.022086771	40	4.779296842	1.003801148	40	6.160472528	0.967315989	40	4.754839546	0.977500728	40	4.944779595	0.999722933	40	5.899400932	Quantified
P61205	ADP-ribosylation factor 3	Arf3	FUNCTION: GTP-binding protein that functior	ADP-ribosylation factor 3 O	33.7	3	5	15	181	20.58773094	7.430175781	1.048980392	11	6.684690693	0.976045142	11	9.695908439	0.999028026	11	3.976536048	1.000585963	11	4.469955144	0.990161796	11	6.326778202	0.985254278	11	4.113841964	Quantified
Q01149	Collagen alpha-2(I) chain	Col1a2	FUNCTION: Type I collagen is a member of g	Collagen alpha-2(I) chain O	4.88	6	6	27	1372	129.4783276	9.187988281	1.021936585	27	3.279598907	1.011921024	27	7.004795396	0.971319967	27	6.857373533	0.985576218	27	8.832807499	1.052599316	27	10.76770063	0.983052284	27	7.576044089	Quantified
Q9ERK4	Exportin-2	Cse1l	FUNCTION: Export receptor for importin-alph	Exportin-2 OS=Mus muscul	9.58	9	9	14	971	110.3842869	5.770019531	1.017803375	14	2.021290882	0.990790215	14	5.412856627	1.047512984	14	3.151037121	0.986810512	14	3.838043968	0.944505506	14	12.27359918	1.004588418	14	3.589420577	Quantified
P24549	Retinal dehydrogenase 1	Aldh1a1	FUNCTION: Can convert/oxidize retinaldehyc	Retinal dehydrogenase 1 O	49.5	11	20	31	501	54.43284429	7.796386719	1.024246944	17	8.873920766	1.013987425	17	6.88664257	1.018846086	17	6.619252141	0.983053919	17	10.53574357	0.994292112	17	9.199325267	0.925920084	17	9.797100342	Quantified
Q9CVB6	Actin-related protein 2/3 complex subunit 2	Arpc2	FUNCTION: Actin-binding component of the A	Actin-related protein 2/3 co	34.33	13	13	41	300	34.3355231	7.356933594	1.041108162	41	2.72544085	1.004751478	41	8.949499468	1.004158086	41	8.070758904	0.973364763	41	8.24909259	0.935242479	41	12.22015776	1.022199016	41	5.453332634	Quantified
Q9WU78	Programmed cell death 6-interacting protein	Pdcd6ip	FUNCTION: Multifunctional protein involved i	Programmed cell death 6-ir	11.97	11	11	20	869	95.96421467	6.521972656	1.002767649	20	3.449905596	1.01170401	20	7.441242975	1.037388201	20	5.109914742	0.961708676	20	6.191259357	0.991359476	20	6.677671486	0.996324768	20	5.603479973	Quantified
Q91Y97	Fructose-bisphosphate aldolase B	Aldob		Fructose-bisphosphate aldo	18.68	7	8	24	364	39.48210908	8.265136719	0.970138116	21	3.861298039	1.045349384	21	5.93094213	0.995164064	21	5.573527166	0.942402823	21	13.02401136	1.097626664	21	6.558320613	0.991515463	21	4.117133485	Quantified
Q61753	D-3-phosphoglycerate dehydrogenase	Phgdh	FUNCTION: Catalyzes the reversible oxidatio	D-3-phosphoglycerate dehy	18.95	7	7	18	533	56.54911991	6.536621094	0.99352815	18	5.52572892	0.993907769	18	3.685216558	0.999372173	18	4.640312006	1.008504462	18	4.849300052	0.981996689	18	8.223432919	1.017333572	18	3.914659966	Quantified
P29351	Tyrosine-protein phosphatase non-receptor type 6	Ptpn6	FUNCTION: Modulates signaling by tyrosine	Tyrosine-protein phosphata	35.13	19	19	43	595	67.51718733	7.811035156	1.023600157	43	7.90038846	1.042967077	43	5.701496795	1.113651171	43	6.082529776	0.927523446	43	7.62746727	0.904422766	43	14.71572005	0.947234957	43	7.395659422	Quantified
P80313	T-complex protein 1 subunit eta	Cct7	FUNCTION: Component of the chaperonin-co	T-complex protein 1 subuni	19.3	9	9	20	544	59.61411078	7.840332031	1.042730763	20	5.329989665	1.028921547	20	2.913995031	1.026691296	20	5.56237986	0.96438436	20	4.479402572	0.925352868	20	5.10896178	0.974427315	20	5.963319958	Quantified
Q61001	Laminin subunit alpha-5	Lama5	FUNCTION: Binding to cells via a high affinity	Laminin subunit alpha-5 OS	5.19	16	16	32	3718	403.7915676	6.727050781	1.014781272	32	5.125581386	0.981010231	32	4.126143462	0.973087707	32	9.580202113	1.041518412	32	8.762063095	1.029675315	32	6.674767434	1.006630991	32	9.102287482	Quantified
P62631	Elongation factor 1-alpha 2	Eef1a2	FUNCTION: This protein promotes the GTP-d	Elongation factor 1-alpha 2	21.6	1	9	25	463	50.42231472	9.026855469	0.950719148	1		0.989478739	1		0.982300749	1		1.069358978	1		1.096241206	1		0.975645674	1		Quantified
Q68FD5	Clathrin heavy chain 1	Cltc	FUNCTION: Clathrin is the major protein of th	Clathrin heavy chain 1 OS=	16.9	25	25	39	1675	191.4345158	5.693847656	1.00910635	39	4.378993631	0.996884982	39	2.960786107	0.976410889	39	8.039443512	0.999960332	39	6.842467271	0.991697121	39	7.847807415	1.007159737	39	5.038333564	Quantified
Q9QUI0	Transforming protein RhoA	Rhoa	FUNCTION: Small GTPase which cycles betv	Transforming protein RhoA	37.31	5	5	15	193	21.76808894	6.100097656	1.034993728	15	6.924739915	1.050075928	15	3.765113524	1.013090684	15	10.40970113	0.965469533	15	4.107316088	0.993513375	15	7.769539678	1.01664227	15	6.008496347	Quantified
Q9WVA4	Transgelin-2	Tagln2		Transgelin-2 OS=Mus musc	43.22	7	7	16	199	22.38115775	8.235839844	1.025294539	16	5.858016811	0.95782153	16	6.075589888	1.086782914	16	5.557046439	1.014912748	16	3.623738649	1.101675095	16	10.14061715	0.87184542	16	9.264543856	Quantified
P14824	Annexin A6	Anxa6	FUNCTION: May associate with CD21. May r	Annexin A6 OS=Mus musc	32.1	18	20	30	673	75.83740127	5.503417969	1.018694107	28	5.262552541	1.035929928	28	4.391870575	1.054763494	28	8.358371213	0.939362249	28	6.439701751	0.90191941	28	8.098191628	0.961255452	28	7.930995849	Quantified
P02469	Laminin subunit beta-1	Lamb1	FUNCTION: Binding to cells via a high affinity	Laminin subunit beta-1 OS=	10.47	18	18	39	1786	196.9614502	4.944824219	1.007715004	39	4.252604183	0.986022624	39	4.335456145	0.990459355	39	4.18781536	1.021213181	39	5.775278984	1.006765567	39	9.002317637	0.986447983	39	4.971671106	Quantified
Q9Z1Q9	Valine--tRNA ligase	Vars1		Valine--tRNA ligase OS=Mu	12.27	13	13	21	1263	140.1270933	7.767089844	1.022088821	21	6.744673332	1.011298591	21	5.034512445	1.068314612	21	8.849213332	0.974553304	21	6.022103011	0.945413169	21	10.49320798	0.992770798	21	8.659269721	Quantified
Q62465	Synaptic vesicle membrane protein VAT-1 homolog	Vat1	FUNCTION: Plays a part in calcium-regulated	Synaptic vesicle membrane	16.26	5	5	9	406	43.06896216	6.366699219	1.030142707	9	2.911746905	1.022985358	9	4.111644174	0.993044299	9	3.57266515	0.977822526	9	3.454692459	0.982236385	9	6.034278608	0.981120611	9	3.118247377	Quantified
O70435	Proteasome subunit alpha type-3	Psma3	FUNCTION: Component of the 20S core prote	Proteasome subunit alpha t	35.69	10	10	27	255	28.38708781	5.439941406	1.018208547	26	3.953460649	1.020247368	26	3.0382491	1.023876186	26	7.404885228	0.991340065	26	6.433763136	0.908152922	26	12.3912405	1.013326032	26	3.69750597	Quantified
B0V2N1	Receptor-type tyrosine-protein phosphatase S	Ptprs	FUNCTION: Cell surface receptor that binds t	Receptor-type tyrosine-prot	6.55	8	8	17	1907	211.7709001	7.225097656	1.021027983	17	3.694811245	1.012282019	17	3.471814672	1.01310535	17	7.379266477	0.990058368	17	3.382361978	0.972146833	17	10.00353361	1.032518644	17	5.526427149	Quantified
Q9R1P4	Proteasome subunit alpha type-1	Psma1	FUNCTION: Component of the 20S core prote	Proteasome subunit alpha t	31.56	8	8	20	263	29.52782531	6.455566406	1.019264974	20	4.524890327	1.014594982	20	5.811360896	0.995131829	20	6.429296766	0.990739869	20	7.669450121	0.973218665	20	5.985845382	1.027870471	20	5.018684448	Quantified
Q6URW6	Myosin-14	Myh14	FUNCTION: Cellular myosin that appears to p	Myosin-14 OS=Mus muscu	6.25	5	11	16	2000	228.4463478	5.554199219	0.978194363	6	9.623307091	0.921185231	6	8.147589104	0.953381361	6	21.80535849	1.015622099	6	11.37508396	1.050159295	6	11.95739176	1.055836544	6	8.305008333	Quantified
Q99JY3	GTPase IMAP family member 4	Gimap4	FUNCTION: During thymocyte development,	GTPase IMAP family memb	35.98	11	11	22	328	38.02035691	6.227050781	1.043672909	22	4.763087964	1.029032765	22	7.211435044	0.969974173	22	7.105867632	1.024937602	22	7.086229794	0.976952906	22	12.5235048	0.98448288	22	6.056465811	Quantified
P68510	14-3-3 protein eta	Ywhah	FUNCTION: Adapter protein implicated in the	14-3-3 protein eta OS=Mus	42.28	5	9	41	246	28.19402538	4.894042969	0.934717532	15	10.63772998	0.909972458	15	10.24337128	1.048167127	15	8.348342632	1.049670796	15	19.42822771	1.031299758	15	8.568786226	0.994683829	15	7.141126776	Quantified
Q05793	Basement membrane-specific heparan sulfate proteo	Hspg2	FUNCTION: Integral component of basement	Basement membrane-speci	6.45	20	20	31	3707	398.0394811	6.315917969	0.989197553	31	7.757562217	1.018295328	31	7.172747332	1.014415353	31	12.39008901	1.002733925	31	6.375924627	0.978313339	31	10.87734287	0.988467649	31	7.305882639	Quantified
Q9Z2U1	Proteasome subunit alpha type-5	Psma5	FUNCTION: Component of the 20S core prote	Proteasome subunit alpha t	29.46	5	5	16	241	26.39420625	4.792480469	0.981718063	16	7.69053739	0.964500113	16	8.411586017	0.992466064	16	16.3177786	1.043780395	16	3.812467851	1.073225125	16	5.702828861	0.974864182	16	4.429640625	Quantified
Q61937	Nucleophosmin	Npm1	FUNCTION: Involved in diverse cellular proce	Nucleophosmin OS=Mus m	21.92	5	5	17	292	32.53981467	4.767089844	0.998017261	17	5.590078396	0.963060232	17	7.415890905	1.068035885	17	4.480308226	1.012648295	17	6.601994711	1.003751148	17	7.328183509	0.973437525	17	6.660576806	Quantified
Q9CWJ9	Bifunctional purine biosynthesis protein ATIC	Atic	FUNCTION: Bifunctional enzyme that catalyz	Bifunctional purine biosynth	17.06	6	6	16	592	64.17712588	6.756347656	1.017640355	16	6.744753361	1.037156735	16	3.640891074	0.984444975	16	7.103726991	0.983171491	16	9.585231556	0.928606648	16	7.407719228	1.000676203	16	6.675629972	Quantified
A2A8L5	Receptor-type tyrosine-protein phosphatase F	Ptprf	FUNCTION: Possible cell adhesion receptor.	Receptor-type tyrosine-prot	9.64	12	14	21	1898	211.356056	6.653808594	1.001220033	19	6.775341557	0.999922912	19	3.333406887	0.990904902	19	5.770931751	0.992922262	19	5.744026469	0.970196495	19	4.023228847	1.006529225	19	4.873901352	Quantified
P21981	Protein-glutamine gamma-glutamyltransferase 2	Tgm2	FUNCTION: Catalyzes the cross-linking of pr	Protein-glutamine gamma-g	23.91	13	13	18	686	77.01226805	5.097167969	1.056282153	15	4.788618347	1.044783623	15	4.130036588	1.016862995	15	3.188197808	0.992433605	15	7.03236696	0.96783869	15	12.25995723	0.961496673	15	8.026326378	Quantified
Q8VDN2	Sodium/potassium-transporting ATPase subunit alph	Atp1a1	FUNCTION: This is the catalytic component c	Sodium/potassium-transpor	10.65	9	9	12	1023	112.9098102	5.452636719	0.988837045	12	4.965076488	0.985091967	12	9.105801584	1.041666202	12	5.941738965	0.980074295	12	5.141336227	0.929164945	12	10.80071867	0.972571114	12	7.033089126	Quantified
P70168	Importin subunit beta-1	Kpnb1	FUNCTION: Functions in nuclear protein impo	Importin subunit beta-1 OS=	18.38	13	13	22	876	97.1220527	4.779785156	1.004457232	22	5.293601936	1.023215621	22	4.430374801	1.032480377	22	7.298510922	0.977205848	22	3.809561654	0.949090712	22	8.283213785	0.996132315	22	5.421773437	Quantified
P09041	Phosphoglycerate kinase 2	Pgk2	FUNCTION: Essential for sperm motility and m	Phosphoglycerate kinase 2	16.31	1	7	31	417	44.82421595	6.800292969	1.018557515	7	1.456152745	0.992338399	7	1.419843728	0.96657121	7	2.467949767	1.027408822	7	1.501141421	0.989977391	7	5.814064744	0.994050471	7	1.833313944	Quantified
P28352	DNA-(apurinic or apyrimidinic site) endonuclease	Apex1	FUNCTION: Multifunctional protein that plays	DNA-(apurinic or apyrimidir	19.56	4	4	15	317	35.46811427	7.913574219	1.01857689	15	4.604384037	1.04048821	15	7.182183968	1.046879213	15	5.552441743	0.944608264	15	3.983379625	0.984426714	15	11.39601554	0.966354317	15	5.110022535	Quantified
Q9Z204	Heterogeneous nuclear ribonucleoproteins C1/C2	Hnrnpc	FUNCTION: Binds pre-mRNA and nucleates t	Heterogeneous nuclear ribc	17.89	6	6	14	313	34.36386304	5.046386719	1.026299487	14	4.221952748	0.939400748	14	10.35763884	1.122963742	14	7.777529545	0.981463949	14	3.708145855	0.983290869	14	7.262126881	0.925957671	14	10.05528749	Quantified
Q8BK62	Olfactomedin-like protein 3	Olfml3	FUNCTION: Secreted scaffold protein that pla	Olfactomedin-like protein 3	19.21	9	9	20	406	45.71717973	6.227050781	1.008690474	20	6.083341546	1.004140136	20	5.026633503	0.989579397	20	5.996986527	1.004276532	20	7.362089389	0.947290651	20	4.568105579	1.00652765	20	4.977225601	Quantified
P62827	GTP-binding nuclear protein Ran	Ran	FUNCTION: GTPase involved in nucleocytop	GTP-binding nuclear protei	29.63	6	6	17	216	24.4076189	7.488769531	1.006123348	17	4.204649853	1.028579766	17	4.870343568	1.004349122	17	8.75024641	1.015484285	17	8.497415593	0.996827736	17	4.897229431	0.973184434	17	4.739087873	Quantified
Q60972	Histone-binding protein RBBP4	Rbbp4	FUNCTION: Core histone-binding subunit tha	Histone-binding protein RBI	19.06	4	7	21	425	47.62607453	4.894042969	1.033411131	14	6.213849784	0.967421769	14	7.700751718	1.061758956	14	10.70002628	0.934030451	14	5.616568804	0.950040364	14	9.774412045	0.994958201	14	8.261355179	Quantified
Q64449	C-type mannose receptor 2	Mrc2	FUNCTION: May play a role as endocytotic le	C-type mannose receptor 2	5.61	8	8	20	1479	166.9677111	5.985839844	0.976678103	20	4.56962238	0.988378608	20	3.122835151	1.014034124	20	3.800458746	1.02391889	20	5.380734666	0.959017087	20	8.227015577	1.003311654	20	4.121130012	Quantified
A2AQ07	Tubulin beta-1 chain	Tubb1	FUNCTION: Tubulin is the major constituent c	Tubulin beta-1 chain OS=M	13.53	2	6	26	451	50.40854408	5.071777344	1.008501163	7	6.169442829	0.990030123	7	1.416196906	0.960268919	7	4.237469617	1.024478566	7	3.840563563	0.928669059	7	10.1668092	1.044552798	7	2.7566421	Quantified
P21550	Beta-enolase	Eno3	FUNCTION: Appears to have a function in str	Beta-enolase OS=Mus mus	17.51	2	5	12	434	46.99530586	7.181152344	0.963185458	3	6.748693414	1.012837764	3	1.359170519	0.918879104	3	7.621750686	0.992267629	3	6.861603632	1.098666169	3	9.447716754	1.049896905	3	2.663120997	Quantified
P14152	Malate dehydrogenase, cytoplasmic	Mdh1		Malate dehydrogenase, cyt	39.52	13	13	23	334	36.48808123	6.580566406	1.007947035	23	4.193615026	1.055976389	23	7.053075002	1.071170675	23	5.837245371	0.928960139	23	6.167375203	0.948468064	23	9.718508681	0.973804448	23	7.694292165	Quantified
P62960	Y-box-binding protein 1	Ybx1	FUNCTION: DNA- and RNA-binding protein ir	Y-box-binding protein 1 OS	20.19	3	3	5	322	35.70857168	9.876464844	1.074068035	5	0.94846698	0.97929282	5	1.489168843	1.008143408	5	4.318436677	1.020010266	5	1.367730901	0.951267325	5	5.79153707	1.004005075	5	2.841582487	Quantified
Q9ES30	Complement C1q tumor necrosis factor-related protei	C1qtnf3		Complement C1q tumor ne	13.01	4	4	25	246	26.81078282	6.741699219	1.014773953	25	4.845455444	1.033622151	25	4.89267353	1.007675502	25	7.030699604	1.023397705	25	7.519544191	0.936363816	25	5.820440616	1.007412164	25	5.261892935	Quantified
Q9Z2U0	Proteasome subunit alpha type-7	Psma7	FUNCTION: Component of the 20S core prote	Proteasome subunit alpha t	31.85	9	9	22	248	27.83761489	8.455566406	0.99444903	22	4.204822033	1.010405169	22	2.709686344	1.02594183	22	6.154527761	1.001327085	22	4.499378449	0.927542165	22	8.921738928	1.007009284	22	3.877477325	Quantified
Q60668	Heterogeneous nuclear ribonucleoprotein D0	Hnrnpd	FUNCTION: Binds with high affinity to RNA m	Heterogeneous nuclear ribc	20.85	6	6	13	355	38.33029334	7.811035156	1.031099574	13	5.723865018	1.068779631	13	4.677779656	1.077537042	13	5.430149509	0.931857141	13	4.414568603	0.915186057	13	3.402906543	0.953875509	13	4.50179885	Quantified
Q9R098	Hepatocyte growth factor activator	Hgfac	FUNCTION: Activates hepatocyte growth fact	Hepatocyte growth factor ac	5.36	3	3	13	653	70.52186119	7.034667969	1.022221073	13	3.351408341	1.004168115	13	5.030981469	1.000160439	13	4.176013594	1.007016618	13	5.151248395	0.979463949	13	4.545861008	1.032985244	13	4.648870616	Quantified
Q8BGQ7	Alanine--tRNA ligase, cytoplasmic	Aars1	FUNCTION: Catalyzes the attachment of alar	Alanine--tRNA ligase, cytop	12.5	12	12	20	968	106.8412314	5.668457031	1.012011913	20	4.359458069	1.017743646	20	3.623173697	1.015725315	20	8.497268337	0.996611146	20	7.106729183	0.984767104	20	9.699340588	0.972705928	20	5.425715704	Quantified
P62835	Ras-related protein Rap-1A	Rap1a	FUNCTION: Induces morphological reversion	Ras-related protein Rap-1A	30.43	1	6	21	184	20.97370894	6.668457031	0.98725305	1		0.933490651	1		0.796996037	1		1.180589901	1		1.22774676	1		1.017660121	1		Quantified
P29391	Ferritin light chain 1	Ftl1	FUNCTION: Stores iron in a soluble, non-toxi	Ferritin light chain 1 OS=Mu	48.63	6	6	13	183	20.78949658	5.998535156	0.989216493	13	2.277378171	0.96464654	13	5.86190364	1.09797359	13	9.058611307	1.013166428	13	3.433760435	1.013155145	13	3.139268264	0.938954914	13	6.245211111	Quantified
Q04857	Collagen alpha-1(VI) chain	Col6a1	FUNCTION: Collagen VI acts as a cell-bindin	Collagen alpha-1(VI) chain	11.22	10	10	16	1025	108.4219373	5.363769531	1.006047148	16	3.547596011	1.015919768	16	7.401877335	0.962390302	16	6.013716811	1.042074143	16	10.42054768	1.022788363	16	5.423826925	0.998938987	16	6.84236466	Quantified
Q8BH64	EH domain-containing protein 2	Ehd2	FUNCTION: ATP- and membrane-binding pro	EH domain-containing prote	16.39	6	7	14	543	61.13584158	6.507324219	0.97330916	9	6.438332955	1.013221429	9	2.356819447	1.100494344	9	8.126782237	0.951928895	9	5.78978986	0.889865888	9	16.380613	0.957176769	9	6.936260828	Quantified
P47754	F-actin-capping protein subunit alpha-2	Capza2	FUNCTION: F-actin-capping proteins bind in a	F-actin-capping protein sub	26.22	5	6	13	286	32.94661672	5.846191406	1.051663847	12	9.077777406	1.016267596	12	11.9587407	1.028390959	12	3.910044534	0.982454156	12	10.01831705	1.012223534	12	4.54079708	0.953263359	12	9.350072399	Quantified
Q63918	Caveolae-associated protein 2	Cavin2	FUNCTION: Plays an important role in caveol	Caveolae-associated protei	29.67	13	13	31	418	46.73538139	5.211425781	1.004736402	31	4.180435033	1.037518492	31	4.96573837	0.977799713	31	7.256102137	0.982984766	31	5.150730648	0.972282796	31	9.039209592	0.997546749	31	7.380070184	Quantified
O88844	Isocitrate dehydrogenase [NADP] cytoplasmic	Idh1		Isocitrate dehydrogenase [N	24.64	12	12	27	414	46.64446883	7.166503906	0.979479937	27	5.44758578	0.995533787	27	2.702761119	1.052750999	27	4.920682344	1.001495577	27	4.193920552	0.968098489	27	6.749534987	1.003531473	27	7.907097605	Quantified
Q2PZL6	Protocadherin Fat 4	Fat4	FUNCTION: Cadherins are cell-cell interactio	Protocadherin Fat 4 OS=Mu	3.09	14	14	27	4981	539.9816612	4.906738281	1.012884896	27	5.739235164	0.987202654	27	2.545267033	0.99922009	27	2.427032176	1.0257812	27	4.922684663	0.933925671	27	7.075016493	1.012568478	27	3.895152231	Quantified
P45591	Cofilin-2	Cfl2	FUNCTION: Controls reversibly actin polymer	Cofilin-2 OS=Mus musculus	18.67	2	3	12	166	18.6978392	7.884277344	1.020751033	2	13.32617508	1.010991894	2	3.305452515	0.961779409	2	5.724474218	0.899033406	2	9.431493547	0.894895571	2	11.60664009	1.179263879	2	18.50215772	Quantified
P26638	Serine--tRNA ligase, cytoplasmic	Sars1	FUNCTION: Catalyzes the attachment of seri	Serine--tRNA ligase, cytopl	22.66	10	10	23	512	58.3517451	6.303222656	0.996550359	23	7.530490541	0.995782493	23	8.682871059	1.009160297	23	10.34342175	0.978059754	23	7.750003945	0.972272678	23	13.29644684	1.003261282	23	7.265217986	Quantified
Q99JI6	Ras-related protein Rap-1b	Rap1b	FUNCTION: GTP-binding protein that posses	Ras-related protein Rap-1b	41.3	3	8	26	184	20.8115937	5.782714844	0.999253763	6	5.154015734	1.055744236	6	1.596473824	1.073411472	6	5.527311457	0.92568523	6	2.677742619	0.981379948	6	6.006504381	0.950432468	6	2.01505901	Quantified
P28665	Murinoglobulin-1	Mug1	FUNCTION: A proteinase activates the inhibit	Murinoglobulin-1 OS=Mus r	0.95	1	2	36	1476	165.1925494	6.417480469	0.98741641	32	4.933907123	0.951470586	32	4.438972134	0.968590704	32	3.875210921	1.096887562	32	4.674672625	0.918695187	32	4.588087032	1.053239861	32	3.232538336	Quantified
P20918	Plasminogen	Plg	FUNCTION: Plasmin dissolves the fibrin of bl	Plasminogen OS=Mus mus	4.19	3	4	37	812	90.74891761	6.595214844	0.904953086	34	7.251066296	1.075821323	34	5.206200102	1.150218729	34	7.936687731	0.891616312	34	14.63633026	0.982049472	34	9.823182053	0.968993746	34	4.461286633	Quantified
Q9D0E1	Heterogeneous nuclear ribonucleoprotein M	Hnrnpm	FUNCTION: Pre-mRNA binding protein in viv	Heterogeneous nuclear ribc	10.56	6	6	11	729	77.59738112	8.631347656	1.010041153	11	3.796333581	0.990706807	11	5.592729385	0.930572641	11	8.012514793	1.045988721	11	6.544576716	1.042711961	11	6.416272682	1.016168472	11	3.908289872	Quantified
P62141	Serine/threonine-protein phosphatase PP1-beta catal	Ppp1cb	FUNCTION: Protein phosphatase that associ	Serine/threonine-protein ph	19.57	2	6	12	327	37.16262773	6.188964844	1.01066745	1		0.99634736	1		0.953354221	1		1.026610252	1		1.055115707	1		1.006830158	1		Quantified
P62821	Ras-related protein Rab-1A	Rab1A	FUNCTION: The small GTPases Rab are key	Ras-related protein Rab-1A	60.98	6	12	20	205	22.66338034	6.214355469	1.031602029	9	2.761512788	1.014826279	9	2.518018465	1.009937412	9	3.516970234	1.007309486	9	4.284135704	0.938163325	9	4.166598402	0.970621206	9	7.008118256	Quantified
P07742	Ribonucleoside-diphosphate reductase large subunit	Rrm1	FUNCTION: Provides the precursors necessa	Ribonucleoside-diphosphat	16.16	12	12	16	792	90.15251002	6.697753906	1.025240251	16	3.351019685	0.965208857	16	5.102613824	1	16	5.646586171	1.004122499	16	3.429586048	1.005666218	16	5.855932773	1.027954658	16	7.21072994	Quantified
Q8BPB5	EGF-containing fibulin-like extracellular matrix protein	Efemp1	FUNCTION: Binds EGFR, the EGF receptor, i	EGF-containing fibulin-like	9.53	4	4	18	493	54.91627772	5.135253906	0.977635246	18	5.216556968	1.02227284	18	5.604421599	1.05961523	18	7.668815967	0.979375692	18	6.065968884	0.937739072	18	9.474377848	0.97667811	18	6.633484199	Quantified
P10922	Histone H1.0	H1f0	FUNCTION: Histones H1 are necessary for th	Histone H1.0 OS=Mus mus	27.32	5	5	14	194	20.84819184	10.90185547	0.81788547	14	16.86059472	0.814430015	14	19.35050451	0.867136735	14	16.26379662	1.277831095	14	16.49441186	1.401730165	14	20.87351785	0.981099788	14	6.42186846	Quantified
P28656	Nucleosome assembly protein 1-like 1	Nap1l1	FUNCTION: Plays a key role in the regulation	Nucleosome assembly prot	11.51	2	3	13	391	45.31690931	4.462402344	1.03376518	6	2.825589617	1.035262466	6	2.85195009	1.071313374	6	4.362618254	0.966934143	6	3.87701293	0.965448078	6	7.046518634	0.940977683	6	4.044236951	Quantified
P30681	High mobility group protein B2	Hmgb2	FUNCTION: Multifunctional protein with vario	High mobility group protein	33.81	7	8	28	210	24.14686491	7.312988281	0.986328917	27	3.303008278	1.064249666	27	6.588084421	1.08816301	27	8.464450926	0.94058678	27	8.886584227	0.91375671	27	11.77890354	0.937369422	27	6.673826671	Quantified
Q9CPW4	Actin-related protein 2/3 complex subunit 5	Arpc5	FUNCTION: Component of the Arp2/3 comple	Actin-related protein 2/3 co	38.41	5	5	11	151	16.27832796	5.668457031	1.011630124	11	2.865891267	0.994680423	11	5.291658072	1.044356231	11	5.185003501	1.016674244	11	5.428986949	0.983618151	11	2.833166005	0.935727245	11	3.768516758	Quantified
O09173	Homogentisate 1,2-dioxygenase	Hgd		Homogentisate 1,2-dioxyge	11.69	5	5	12	445	49.92754142	7.239746094	1.005649853	12	3.771102923	1.016426863	12	3.601326275	1.025468957	12	7.130080535	0.99296595	12	2.791537573	0.92160593	12	7.016948348	1.0322107	12	7.454777681	Quantified
Q8VHX6	Filamin-C	Flnc	FUNCTION: Muscle-specific filamin, which pl	Filamin-C OS=Mus musculu	7.89	11	20	26	2726	290.9374835	5.947753906	1.007680193	13	6.055860223	0.995391596	13	4.172571242	0.998894938	13	4.427195855	1.007818882	13	10.16596968	0.915373746	13	9.020737661	1.009077652	13	7.387770514	Quantified
Q8VCM7	Fibrinogen gamma chain	Fgg	FUNCTION: Together with fibrinogen alpha (F	Fibrinogen gamma chain O	4.36	4	4	15	436	49.35983954	5.858886719	0.919768938	15	10.78897388	0.92702572	15	6.382058532	1.387661121	15	49.01863036	0.931514267	15	15.31216404	0.856386971	15	15.46351193	0.924447003	15	12.16400483	Quantified
P14869	60S acidic ribosomal protein P0	Rplp0	FUNCTION: Ribosomal protein P0 is the func	60S acidic ribosomal protei	25.87	6	6	14	317	34.19475276	6.252441406	1.034905924	14	5.605038361	0.975430036	14	4.751818193	1.009662327	14	4.387629914	1.016514061	14	4.761887308	0.97016186	14	9.607434708	0.987296087	14	2.199131988	Quantified
P27773	Protein disulfide-isomerase A3	Pdia3		Protein disulfide-isomerase	27.13	15	15	25	505	56.64268632	6.214355469	0.940510103	25	5.347202525	0.963704265	25	5.484103574	0.961019353	25	8.734156515	1.061998376	25	7.442601311	1.101196736	25	5.622361338	1.054225046	25	6.642168341	Quantified
Q9Z1Z0	General vesicular transport factor p115	Uso1	FUNCTION: General vesicular transport facto	General vesicular transport	13.87	12	12	20	959	106.9166014	4.932128906	1.006663385	20	3.836027566	0.987276592	20	7.010232033	0.982002075	20	5.585124144	1.036083669	20	5.631343311	0.966114779	20	8.250465918	1.008546179	20	3.743250588	Quantified
Q62093	Serine/arginine-rich splicing factor 2	Srsf2	FUNCTION: Necessary for the splicing of pre	Serine/arginine-rich splicing	19.46	4	4	19	221	25.461185	11.85400391	1.01313231	19	5.306644988	0.995412364	19	6.316330385	1.042568522	19	8.614863275	0.96135495	19	13.46477975	0.942853815	19	10.01042869	1.038909531	19	8.228856315	Quantified
P08074	Carbonyl reductase [NADPH] 2	Cbr2	FUNCTION: May function in the pulmonary m	Carbonyl reductase [NADPH	43.85	8	9	15	244	25.94161562	9.012207031	1.007079471	13	4.256240288	1.044099431	13	3.764482359	1.047070488	13	6.394476654	0.977054163	13	5.83481622	0.985735312	13	7.557239749	0.945948774	13	4.443793003	Quantified

P60766	Cell division control protein 42 homolog	Cdc42	FUNCTION: Plasma membrane-associated s	Cell division control protein	35.6	5	6	22	191	21.24501818	6.551269531	1.003870888	20	4.133163217	1.023421236	20	5.899929048	0.997686388	20	3.859276707	0.984031478	20	7.922921679	0.977425994	20	6.743600637	1.023890967	20	5.099479841	Quantified
P10493	Nidogen-1	Nid1	FUNCTION: Sulfated glycoprotein widely dist	Nidogen-1 OS=Mus muscul	9.8	10	10	16	1245	136.4504957	5.439941406	0.969163872	16	6.691942449	1.01465031	16	4.483458657	1.010438507	16	8.66367649	1.000715135	16	8.226217512	0.957461771	16	9.727130651	1.003276399	16	10.84333898	Quantified
Q3TXS7	26S proteasome non-ATPase regulatory subunit 1	Psmd1	FUNCTION: Component of the 26S proteasor	26S proteasome non-ATPa	9.97	8	8	13	953	105.6626576	5.389160156	1.010936135	13	5.107043566	1.030176174	13	4.040121031	0.951030318	13	9.75835444	0.976888529	13	9.412160415	1.066372702	13	13.90561158	0.997480013	13	8.935918262	Quantified
P16460	Argininosuccinate synthase	Ass1	FUNCTION: One of the enzymes of the urea (	Argininosuccinate synthase	25.49	11	11	27	412	46.55501256	8.221191406	1.020581475	27	4.865151902	1.029562376	27	5.124580515	0.99845753	27	4.738426271	0.964002282	27	4.875809895	0.95354726	27	11.49750248	1.026168605	27	5.287993577	Quantified
Q9QUM9	Proteasome subunit alpha type-6	Psma6	FUNCTION: Component of the 20S core prot	Proteasome subunit alpha t	39.84	9	9	18	246	27.35479932	6.756347656	0.996621975	18	3.456919186	0.996336425	18	4.600277142	0.994580429	18	8.294987191	1.039529148	18	4.48154419	0.965708845	18	7.382808081	1.019494602	18	7.590041014	Quantified
Q9D8N0	Elongation factor 1-gamma	Eef1g	FUNCTION: Probably plays a role in anchorin	Elongation factor 1-gamma	27.46	10	10	16	437	50.02916022	6.741699219	1.017119305	15	3.246240546	1.047603271	15	5.168979761	1.047548938	15	3.853557084	0.960517699	15	2.267328053	0.928224099	15	7.027847531	0.971663703	15	2.796343353	Quantified
P02535	Keratin, type I cytoskeletal 10	Krt10	FUNCTION: Plays a role in the establishment	Keratin, type I cytoskeletal	10.7	4	6	16	570	57.73505521	5.109863281	1.01695061	13	19.46857279	1.03439607	13	11.13136676	0.80614407	13	22.94717703	1.171229504	13	18.96508477	0.881960777	13	22.78745468	0.89665555	13	13.88936683	Quantified
P19157	Glutathione S-transferase P 1	Gstp1	FUNCTION: Conjugation of reduced glutathio	Glutathione S-transferase F	15.24	2	2	9	210	23.5941143	7.869628906	1.025003823	9	12.09069416	1.025164326	9	3.944884362	1.03252722	9	9.828038988	0.888849428	9	14.46909209	1.057920467	9	24.66329474	1.00720993	9	9.185562757	Quantified
Q9QXS1	Plectin	Plec	FUNCTION: Interlinks intermediate filaments	Plectin OS=Mus musculus	6.46	27	28	32	4691	533.8610869	5.960449219	0.978332377	30	5.412165691	0.97142685	30	11.76926459	0.987912778	30	8.112446096	1.057098927	30	6.405472169	1.03140169	30	4.696754125	1.003578545	30	4.843537335	Quantified
O08710	Thyroglobulin	Tg	FUNCTION: Acts as a substrate for the produ	Thyroglobulin OS=Mus mus	1.41	4	4	22	2766	304.2773322	5.490722656	1.023841063	22	3.299477111	0.968807552	22	8.054129079	1.077450978	22	11.68227915	0.985531908	22	6.481022601	1.024095884	22	14.84616278	0.953308276	22	4.002725003	Quantified
Q9CW03	Structural maintenance of chromosomes protein 3	Smc3	FUNCTION: Central component of cohesin, a	Structural maintenance of c	10.76	13	13	22	1217	141.4678582	7.181152344	1.046263406	22	4.795983401	0.9905729	22	4.452768945	1.022084567	22	6.723099303	0.986113921	22	3.105634464	0.946624116	22	6.888357762	0.99493396	22	4.412512839	Quantified
Q922U2	Keratin, type II cytoskeletal 5	Krt5		Keratin, type II cytoskeletal	6.21	3	5	10	580	61.72858805	7.752441406	1.012630468	4	2.877797091	0.960412491	4	1.590838497	0.74650578	4	9.583128028	1.283313607	4	19.95242132	0.934037884	4	9.764757526	0.932530858	4	8.520541752	Quantified
P50446	Keratin, type II cytoskeletal 6A	Krt6a	FUNCTION: Epidermis-specific type I keratin	Keratin, type II cytoskeletal	4.88	1	3	7	553	59.29862033	7.942871094	1.023442308	1		1.089847133	1		0.995649453	1		0.967656897	1		0.900687109	1		0.995112681	1		Quantified
P62259	14-3-3 protein epsilon	Ywhae	FUNCTION: Adapter protein implicated in the	14-3-3 protein epsilon OS=I	44.71	9	11	30	255	29.15541736	4.741699219	0.967439256	17	3.324473113	0.937397398	17	3.279968455	1.026294947	17	8.844641197	1.0018019	17	6.052556577	1.101521979	17	7.227796201	1.056458495	17	4.071076846	Quantified
Q8VEK3	Heterogeneous nuclear ribonucleoprotein U	Hnrnpu	FUNCTION: DNA- and RNA-binding protein ir	Heterogeneous nuclear ribo	9.38	9	9	18	800	87.86272924	6.239746094	1.009864164	18	4.770214558	1.012460984	18	7.921013867	1.043773246	18	6.57724622	1.002300085	18	8.634980578	0.975366127	18	5.911467185	0.954753224	18	9.555410014	Quantified
Q61598	Rab GDP dissociation inhibitor beta	Gdi2	FUNCTION: Regulates the GDP/GTP exchan	Rab GDP dissociation inhib	28.09	6	10	22	445	50.50469224	6.252441406	0.972026023	15	6.557154394	1.015713561	15	4.329555468	1.020192149	15	5.773700642	1.027369505	15	8.08608004	0.963739993	15	10.83827695	0.958382554	15	4.501160169	Quantified
Q61176	Arginase-1	Arg1	FUNCTION: Key element of the urea cycle cc	Arginase-1 OS=Mus muscu	12.07	4	4	13	323	34.78617365	7.005371094	0.981444394	13	3.307782284	1.036797994	13	2.772538851	0.971089503	13	3.151259151	0.984030473	13	3.39663838	1.040134973	13	1.882262439	1.001714681	13	6.030454655	Quantified
P06684	Complement C5	C5	FUNCTION: Activation of C5 by a C5 convert	Complement C5 OS=Mus n	2.38	5	5	24	1680	188.7587577	6.814941406	1.012498141	24	3.977831594	0.993892779	24	4.404193552	0.993995525	24	6.016733738	1.030314801	24	4.775120005	0.961331054	24	4.979843953	0.968109384	24	4.772774501	Quantified
O35945	Aldehyde dehydrogenase, cytosolic 1	Aldh1a7	FUNCTION: Can oxidize benzaldehyde, prop	Aldehyde dehydrogenase, c	21.76	1	10	18	501	54.55297753	7.708496094	1.019785156	4	5.894750661	0.992669197	4	2.312150849	0.9866296	4	3.799397698	1.027377097	4	3.13410352	1.02736651	4	7.641504175	0.996177535	4	3.926334772	Quantified
P97333	Neuropilin-1	Nrp1	FUNCTION: Receptor involved in the develop	Neuropilin-1 OS=Mus musc	7.91	5	5	9	923	102.9345669	5.896972656	1.011723713	9	5.900425373	0.997779255	9	3.52531924	0.99409839	9	1.533047481	0.993326317	9	3.39408526	1.015054178	9	6.279414045	1.029832148	9	6.58874306	Quantified
P53657	Pyruvate kinase PKLR	Pklr	FUNCTION: Plays a key role in glycolysis. {E	Pyruvate kinase PKLR OS=	8.36	4	5	24	574	62.26961145	7.093261719	1.01752797	11	7.408263987	1.041340924	11	4.36801379	1.002358069	11	5.276726069	0.947786476	11	1.897920738	0.981186829	11	4.01653447	1.002193702	11	4.381177637	Quantified
O35286	Pre-mRNA-splicing factor ATP-dependent RNA helic	Dhx15	FUNCTION: Pre-mRNA processing factor inv	Pre-mRNA-splicing factor A	4.4	3	3	10	795	90.9492065	7.459472656	1.054603268	10	5.301220639	0.989558454	10	5.494971311	1.001136111	10	6.416639732	0.994985354	10	6.232931793	0.991847795	10	3.495806228	0.995706881	10	5.662485597	Quantified
Q8K1B8	Fermitin family homolog 3	Fermt3	FUNCTION: Plays a central role in cell adhes	Fermitin family homolog 3 (	19.55	11	11	18	665	75.58684965	7.049316406	1.00877073	18	5.696733399	1.027857308	18	5.683455371	1.011563062	18	6.116993633	0.987726629	18	6.110604102	0.933532996	18	6.87373411	1.000116626	18	7.170246757	Quantified
Q91ZW3	SWI/SNF-related matrix-associated actin-dependent	Smarca5	FUNCTION: Helicase that possesses intrinsic	SWI/SNF-related matrix-as	5.52	6	6	10	1051	121.5501534	8.147949219	0.981037217	10	4.457412313	0.930946887	10	3.166810238	0.957735296	10	4.336079946	1.063687222	10	5.464480062	1.101123594	10	11.76859775	1.069807129	10	3.521635023	Quantified
Q8CCK0	Core histone macro-H2A.2	Macroh2a2	FUNCTION: Variant histone H2A which repla	Core histone macro-H2A.2	19.35	5	7	12	372	40.06737352	9.686035156	0.936385571	8	7.146937512	0.971704745	8	4.084729892	0.905086356	8	6.427799642	1.090005135	8	3.111243902	1.048894828	8	5.45141663	1.083798529	8	6.713238206	Quantified
Q06806	Tyrosine-protein kinase receptor Tie-1	Tie1	FUNCTION: Transmembrane tyrosine-protein	Tyrosine-protein kinase rec	4.67	6	6	12	1134	124.503058	6.917480469	1.037418353	12	7.603715283	1.032833059	12	3.390849302	1.002731615	12	7.778322842	0.972955445	12	4.899469157	0.916087383	12	7.306900186	0.967903284	12	8.154606979	Quantified
P47856	Glutamine--fructose-6-phosphate aminotransferase [i	Gfpt1	FUNCTION: Controls the flux of glucose into t	Glutamine--fructose-6-phos	20.37	12	12	22	697	78.48915957	6.844238281	1.006757786	22	5.706605659	1.009010721	22	9.310748871	0.963679908	22	5.534933326	0.999813023	22	6.794505464	1.019263559	22	19.85456187	1.03315058	22	8.990600009	Quantified
P80317	T-complex protein 1 subunit zeta	Cct6a	FUNCTION: Component of the chaperonin-co	T-complex protein 1 subuni	18.08	8	8	11	531	57.96767193	7.078613281	1.061825913	11	4.783474565	1.055889874	11	2.066600095	0.957316602	11	14.30778695	0.944140398	11	4.364463508	0.9273036	11	1.870460476	0.984970321	11	6.970511791	Quantified
Q8R555	Cartilage acidic protein 1	Crtac1		Cartilage acidic protein 1 O	13.16	7	7	10	646	70.28038351	5.135253906	0.981831088	10	7.082088818	1.017305107	10	6.455594582	0.971886941	10	6.712567773	0.990298092	10	5.18905706	1.046821457	10	7.23693499	1.000564083	10	3.252786335	Quantified
Q91ZJ5	UTP--glucose-1-phosphate uridylyltransferase	Ugp2	FUNCTION: Plays a central role as a glucosy	UTP--glucose-1-phosphate	20.87	9	9	18	508	56.94374728	7.605957031	0.998020151	18	2.861939296	1.01085275	18	3.521871144	0.993636307	18	3.772926551	1.014505812	18	4.013483558	0.964721046	18	6.891095702	1.029317509	18	4.262639148	Quantified
Q60864	Stress-induced-phosphoprotein 1	Stip1	FUNCTION: Acts as a co-chaperone for HSP	Stress-induced-phosphopro	24.49	15	15	22	543	62.54240209	6.800292969	0.988855644	22	3.617175602	1.055365388	22	5.928410885	1.008577463	22	10.49015977	0.939146199	22	7.813914502	0.977662365	22	8.622623238	0.998837647	22	3.728135681	Quantified
Q99K51	Plastin-3	Pls3	FUNCTION: Actin-bundling protein. {ECO:00(	Plastin-3 OS=Mus musculu	16.19	4	9	13	630	70.69719067	5.617675781	0.973669313	5	2.764869079	1.046550104	5	1.899585053	1.017471847	5	4.58602189	0.986993401	5	2.506399754	1.084914966	5	3.534847805	0.976509392	5	1.281655094	Quantified
P49429	4-hydroxyphenylpyruvate dioxygenase	Hpd	FUNCTION: Key enzyme in the degradation c	4-hydroxyphenylpyruvate d	22.14	8	8	21	393	45.02588832	7.063964844	1.004393047	21	3.827677243	0.976524771	21	3.652856457	0.949829696	21	5.296461866	1.065581637	21	5.513452059	1.030776458	21	9.333894371	1.01207294	21	5.056520882	Quantified
Q9Z2X1	Heterogeneous nuclear ribonucleoprotein F	Hnrnpf	FUNCTION: Component of the heterogeneou	Heterogeneous nuclear ribo	27.47	7	8	11	415	45.70091694	5.490722656	1.059874705	10	5.723401732	1.02913835	10	7.542983319	0.955666703	10	10.04679946	0.964533431	10	5.645163477	0.978182913	10	10.35663669	1.008566117	10	10.47777711	Quantified
Q9QYB1	Chloride intracellular channel protein 4	Clic4	FUNCTION: Can insert into membranes and f	Chloride intracellular chann	30.83	5	5	11	253	28.71071482	5.592285156	1.005818027	11	4.455341778	1.004274609	11	3.623531854	0.938516088	11	7.543335938	0.987589215	11	9.572260745	0.980403656	11	10.35498257	1.001177435	11	3.90017237	Quantified
P17225	Polypyrimidine tract-binding protein 1	Ptbp1	FUNCTION: Plays a role in pre-mRNA splicin	Polypyrimidine tract-binding	7.21	4	4	9	527	56.44296193	8.338378906	1.073626511	8	5.071302145	1.016832901	8	5.60795588	1.026364205	8	2.972555467	0.983898069	8	4.565076664	0.972765976	8	11.16923685	0.928539095	8	3.896869118	Quantified
Q7TQI3	Ubiquitin thioesterase OTUB1	Otub1	FUNCTION: Hydrolase that can specifically re	Ubiquitin thioesterase OTUI	20.3	4	4	10	271	31.25040299	4.944824219	0.967339974	10	6.156249087	0.963548657	10	4.400126226	1.036690572	10	4.845252199	0.998566096	10	5.508795101	1.052433632	10	8.822686202	1.023255982	10	2.977838433	Quantified
P97298	Pigment epithelium-derived factor	Serpinf1	FUNCTION: Neurotrophic protein; induces ex	Pigment epithelium-derived	7.67	3	3	9	417	46.20523666	6.976074219	0.871644948	9	14.54705956	1.022891756	9	1.656802381	1.056404263	9	17.60701075	0.890025194	9	15.42185258	1.056403464	9	10.22443875	1.008874211	9	8.075579497	Quantified
P62137	Serine/threonine-protein phosphatase PP1-alpha cat	Ppp1ca	FUNCTION: Protein phosphatase that associ	Serine/threonine-protein ph	15.45	1	5	10	330	37.51581201	6.328613281	0.94708044	1		1.039173761	1		1.185078169	1		0.915279403	1		0.929776921	1		0.951138231	1		Quantified
Q9D8U8	Sorting nexin-5	Snx5	FUNCTION: Involved in several stages of intr	Sorting nexin-5 OS=Mus m	27.23	11	11	21	404	46.76806005	6.624511719	0.992582369	20	5.944702106	1.041295969	20	7.098938473	1.01689486	20	8.591167226	0.985018796	20	6.018598983	1.037523412	20	10.71505777	0.967259718	20	7.938835828	Quantified
Q921M7	CYFIP-related Rac1 interactor B	Cyrib	FUNCTION: Negatively regulates RAC1 signa	CYFIP-related Rac1 interac	16.67	5	5	11	324	36.75265567	6.062011719	0.990798691	11	2.88906282	1.017274627	11	6.459811838	1.005663286	11	5.387947837	1.016308276	11	6.404248375	1.005372184	11	6.321504174	0.997381293	11	5.225014023	Quantified
Q9Z1N5	Spliceosome RNA helicase Ddx39b	Ddx39b	FUNCTION: Involved in nuclear export of spli	Spliceosome RNA helicase	23.36	9	9	13	428	49.00396998	5.668457031	1.048403713	13	6.633751293	1.055505216	13	8.424674491	1.062977596	13	5.226209618	0.940087267	13	6.278933356	0.917926036	13	7.836294452	1.006226892	13	6.55601734	Quantified
P98064	Mannan-binding lectin serine protease 1	Masp1	FUNCTION: Functions in the lectin pathway c	Mannan-binding lectin serir	9.09	5	5	14	704	79.91638337	5.554199219	0.987324385	14	5.298623949	1.016638637	14	5.191485927	1.052947412	14	8.039868686	0.9929846	14	2.705503591	0.958178038	14	8.170837902	0.985299533	14	5.183255935	Quantified
P24270	Catalase	Cat	FUNCTION: Occurs in almost all aerobically r	Catalase OS=Mus musculu	17.65	9	9	18	527	59.75765202	7.884277344	1.00037922	18	4.148332378	0.999871132	18	5.759300775	0.972577955	18	5.732059819	0.986527085	18	4.527610645	0.968516033	18	6.996790808	1.019576082	18	4.805461809	Quantified
P63087	Serine/threonine-protein phosphatase PP1-gamma c	Ppp1cc	FUNCTION: Protein phosphatase that associ	Serine/threonine-protein ph	17.03	1	5	10	323	36.95972555	6.536621094	0.954928883	1		1.014470137	1		0.934523255	1		1.077244396	1		1.106748974	1		0.983667072	1		Quantified
P62874	Guanine nucleotide-binding protein G(I)/G(S)/G(T) su	Gnb1	FUNCTION: Guanine nucleotide-binding prote	Guanine nucleotide-binding	15.88	3	6	16	340	37.35301477	5.998535156	1.001324613	7	8.516855819	1.022697809	7	1.458619235	1.060047747	7	10.88240287	0.950957824	7	7.314968865	0.969851926	7	4.569327471	0.961105704	7	1.36453817	Quantified
P01899	H-2 class I histocompatibility antigen, D-B alpha chai	H2-D1	FUNCTION: Involved in the presentation of fo	H-2 class I histocompatibilit	18.78	5	7	11	362	40.81026009	6.727050781	1.021465442	7	1.99670357	0.983741297	7	1.430988169	0.939079682	7	3.985457208	1.053403414	7	2.875870153	1.003388355	7	6.930296559	1.028971515	7	4.025858566	Quantified
P24452	Macrophage-capping protein	Capg	FUNCTION: Calcium-sensitive protein which	Macrophage-capping protei	20.45	6	6	8	352	39.21591208	7.195800781	1.035066344	8	2.724478798	1.01498113	8	5.511055539	1.022671045	8	7.473911572	0.958611455	8	9.111060478	0.964734462	8	5.80317102	0.956855151	8	6.566513064	Quantified
P46471	26S proteasome regulatory subunit 7	Psmc2	FUNCTION: Component of the 26S proteasor	26S proteasome regulatory	20.09	7	7	10	433	48.61713668	5.947753906	1.000563018	10	3.590797207	1.039712482	10	9.047957535	1.020935711	10	5.444157101	0.938649494	10	6.873412156	0.987807858	10	8.736491479	0.9754655	10	5.982110739	Quantified
P14685	26S proteasome non-ATPase regulatory subunit 3	Psmd3	FUNCTION: Component of the 26S proteasor	26S proteasome non-ATPa	21.7	11	11	16	530	60.6803487	8.440917969	0.987548143	16	6.057357458	1.002017582	16	7.546494187	0.982113281	16	6.05013031	0.982761071	16	6.128583745	1.025362091	16	7.222212213	1.011480577	16	6.846394454	Quantified
Q9R1P0	Proteasome subunit alpha type-4	Psma4	FUNCTION: Component of the 20S core prot	Proteasome subunit alpha t	16.86	6	6	14	261	29.45216098	7.723144531	0.989626398	14	2.843377031	1.018789018	14	4.519287713	1.004346758	14	6.118316703	0.999977418	14	4.340104836	0.939890747	14	4.59313256	1.003022443	14	6.529382098	Quantified
P49722	Proteasome subunit alpha type-2	Psma2	FUNCTION: Component of the 20S core prot	Proteasome subunit alpha t	32.05	5	5	6	234	25.91027729	7.430175781	1.010870373	6	4.420185275	0.949531578	6	4.101942135	1.049557235	6	1.702925636	0.984403492	6	10.12154791	0.950646053	6	2.817516675	1.0069899	6	8.319743929	Quantified
P08003	Protein disulfide-isomerase A4	Pdia4		Protein disulfide-isomerase	15.2	9	9	12	638	71.9375129	5.312988281	0.967928697	12	2.407471895	0.963063131	12	7.025031542	0.943081154	12	5.555858783	1.029125839	12	4.796302766	1.093331245	12	7.934560989	1.045449751	12	3.193279949	Quantified
Q8C1B7	Septin-11	Septin11	FUNCTION: Filament-forming cytoskeletal G1	Septin-11 OS=Mus muscul	21.35	4	9	17	431	49.66331563	6.683105469	1.025958538	6	5.344792021	1.016231772	6	6.245935215	1.056962708	6	4.630909206	1.000216068	6	8.104687671	0.94599652	6	8.279854454	0.935339962	6	5.564166521	Quantified
Q922B2	Aspartate--tRNA ligase, cytoplasmic	Dars1	FUNCTION: Catalyzes the specific attachmer	Aspartate--tRNA ligase, cyt	23.15	10	10	16	501	57.11118557	6.493652344	1.025797766	16	5.45428725	0.996660662	16	3.867766261	0.998045161	16	7.502492647	1.01295884	16	13.30840197	0.979379525	16	8.985870554	0.963564657	16	11.95461713	Quantified
P14206	40S ribosomal protein SA	Rpsa	FUNCTION: Required for the assembly and/o	40S ribosomal protein SA C	20.68	4	4	13	295	32.81743973	4.868652344	0.990539613	13	3.313892555	1.008495572	13	5.146333671	1.090165694	13	6.573820629	0.966103159	13	4.912960684	0.993951961	13	4.796277092	0.955515189	13	1.213305552	Quantified
P58774	Tropomyosin beta chain	Tpm2	FUNCTION: Binds to actin filaments in muscl	Tropomyosin beta chain OS	22.18	2	10	18	284	32.81655297	4.703613281	0.947298382	2	0.059333336	0.876967681	2	2.451836092	0.975995823	2	0.68516621	1.106876769	2	0.177683734	1.227417643	2	0.503667389	0.993234651	2	2.260266993	Quantified
Q76MZ3	Serine/threonine-protein phosphatase 2A 65 kDa reg	Ppp2r1a	FUNCTION: The PR65 subunit of protein pho	Serine/threonine-protein ph	21.05	11	11	20	589	65.2809321	5.109863281	0.985124848	20	7.873054471	1.034575805	20	6.629563806	1.013764303	20	12.11849663	0.976476164	20	9.482347626	1.011124087	20	10.70910434	0.966276532	20	7.433295631	Quantified
Q9CU62	Structural maintenance of chromosomes protein 1A	Smc1a	FUNCTION: Involved in chromosome cohesic	Structural maintenance of c	15.49	19	19	21	1233	143.1461032	7.635253906	0.983757608	21	6.187208475	0.983645653	21	6.05908967	1.013776315	21	5.546760868	1.006732588	21	9.676671819	0.973741753	21	7.513074382	0.981476504	21	8.190842708	Quantified
P04186	Complement factor B	Cfb	FUNCTION: Factor B which is part of the alte	Complement factor B OS=M	3.94	3	3	10	761	84.95053889	7.371582031	0.900307513	10	7.17951183	1.077664801	10	4.717155556	1.141306157	10	16.67599847	0.849133536	10	19.83990276	1.07781333	10	10.44507776	0.99935957	10	4.35533504	Quantified
P97798	Neogenin	Neo1	FUNCTION: Multi-functional cell surface rece	Neogenin OS=Mus muscul	5.89	9	9	19	1493	163.0582118	6.468261719	1.016097609	19	5.420038613	1.025047172	19	3.64692825	1.02146511	19	3.4018129	0.950199337	19	4.845619047	0.976228019	19	7.897784523	1.015680929	19	5.03961013	Quantified
Q78ZA7	Nucleosome assembly protein 1-like 4	Nap1l4	FUNCTION: Acts as histone chaperone in nu	Nucleosome assembly prot	16.8	4	5	15	375	42.65286051	4.665527344	1.015084089	8	2.51444642	1.020482413	8	3.491411691	1.042971541	8	12.27378738	0.962600268	8	10.92106161	0.93739492	8	8.88917078	0.949912112	8	3.93157279	Quantified
Q8VIJ6	Splicing factor, proline- and glutamine-rich	Sfpq	FUNCTION: DNA- and RNA binding protein, i	Splicing factor, proline- and	21.03	11	12	20	699	75.39427771	9.437011719	1.004022919	16	3.569412667	1.039899161	16	5.024097825	1.017796141	16	8.194614067	0.950623859	16	7.804141699	0.982269148	16	6.211300428	1.016999738	16	6.972876211	Quantified
P62192	26S proteasome regulatory subunit 4	Psmc1	FUNCTION: Component of the 26S proteasor	26S proteasome regulatory	15.68	4	5	10	440	49.15369606	6.214355469	0.985614386	6	3.031430415	1.047531304	6	5.466687822	1.016804072	6	10.58726926	0.962756509	6	6.315432691	0.972475048	6	3.37404431	0.991540264	6	1.788467988	Quantified
Q91VC3	Eukaryotic initiation factor 4A-III	Eif4a3	FUNCTION: ATP-dependent RNA helicase. Ir	Eukaryotic initiation factor 4	17.52	5	6	12	411	46.81015338	6.727050781	1.018065427	10	4.052220046	1.001387245	10	3.343728833	1.002851354	10	4.876390242	1.013442468	10	2.370394123	1.013372259	10	3.773110315	0.971238233	10	4.56100959	Quantified
Q60841	Reelin	Reln	FUNCTION: Extracellular matrix serine protea	Reelin OS=Mus musculus (	3.18	8	8	15	3461	387.2459873	5.731933594	1.014295657	15	4.674967001	0.960417138	15	6.691987023	0.975633893	15	5.563420706	1.023863921	15	6.625671845	1.019916777	15	6.984809787	1.028114602	15	4.589106696	Quantified
P80318	T-complex protein 1 subunit gamma	Cct3	FUNCTION: Component of the chaperonin-co	T-complex protein 1 subuni	22.39	11	11	20	545	60.59136434	6.697753906	1.036029631	20	4.212790489	1.001643884	20	5.016182433	1.030830644	20	4.919627358	0.958983718	20	4.992971805	0.957157548	20	11.47423726	0.95844319	20	5.452146585	Quantified
P21107	Tropomyosin alpha-3 chain	Tpm3	FUNCTION: Binds to actin filaments in muscl	Tropomyosin alpha-3 chain	36.49	6	14	30	285	32.97380542	4.716308594	1.01066471	12	7.973590282	0.884775444	12	5.963596532	1.006264037	12	5.465375745	1.014651365	12	10.2055112	1.193075746	12	14.57879573	0.917547561	12	5.169692527	Quantified
Q9D554	Splicing factor 3A subunit 3	Sf3a3	FUNCTION: Involved in pre-mRNA splicing a	Splicing factor 3A subunit 3	16.77	7	7	9	501	58.8052381	5.338378906	1.013195672	7	5.671010377	1.018603276	7	4.894490992	1.04360028	7	9.170722937	0.981252579	7	4.459729217	0.973225108	7	9.667034277	0.999693905	7	1.110559843	Quantified
Q93092	Transaldolase	Taldo1	FUNCTION: Transaldolase is important for th	Transaldolase OS=Mus mu	21.96	8	8	22	337	37.36337324	7.034667969	1.005098587	22	3.777312144	1.064896458	22	4.745676651	1.05303461	22	6.402851906	1.000676134	22	5.157987504	0.962809322	22	7.085031704	0.964262633	22	5.886146964	Quantified
P56395	Cytochrome b5	Cyb5a	FUNCTION: Cytochrome b5 is a membrane-b	Cytochrome b5 OS=Mus m	34.33	3	3	4	134	15.23153491	5.071777344	1.009409459	4	0.42152319	0.985500274	4	1.866979502	0.879326131	4	5.133158853	1.036561316	4	2.289742163	1.07303568	4	4.657872689	1.066606764	4	2.022934918	Quantified
P45376	Aldo-keto reductase family 1 member B1	Akr1b1	FUNCTION: Catalyzes the NADPH-depender	Aldo-keto reductase family	26.9	9	9	14	316	35.70945307	7.181152344	0.983925915	14	4.571492759	1.0155639	14	3.398474615	1.017119725	14	4.505101843	0.96902591	14	4.971982075	1.03093172	14	5.561888064	0.973330631	14	4.759753654	Quantified
P61750	ADP-ribosylation factor 4	Arf4	FUNCTION: GTP-binding protein that functior	ADP-ribosylation factor 4 O	29.44	2	4	6	180	20.38361473	7.137207031	1.01079105	2	0.697774508	1.036155757	2	1.225475541	0.969698563	2	7.373818428	1.005809416	2	6.040082113	0.970973435	2	0.062715211	1.013537509	2	2.527703991	Quantified
Q8BK67	Protein RCC2	Rcc2	FUNCTION: Multifunctional protein that may (	Protein RCC2 OS=Mus mu	21.35	11	11	15	520	55.94809652	8.719238281	1.005109277	15	6.09635912	1.029056385	15	3.844914276	1.123880239	15	11.0298519	0.935777376	15	7.34085897	0.938677266	15	6.996245363	0.926005058	15	5.837789518	Quantified
Q9CQ65	S-methyl-5'-thioadenosine phosphorylase	Mtap	FUNCTION: Catalyzes the reversible phosph	S-methyl-5'-thioadenosine p	14.13	3	3	6	283	31.04174725	7.137207031	1.001754824	6	5.928277202	1.003500282	6	4.625002648	0.992670291	6	4.659014741	0.991417025	6	8.222750953	0.961613849	6	5.97273464	0.997966949	6	2.124343242	Quantified
O55222	Integrin-linked protein kinase	Ilk	FUNCTION: Receptor-proximal protein kinase	Integrin-linked protein kinas	15.93	8	8	15	452	51.33992197	8.074707031	1.011239562	15	6.181339339	1.034696875	15	6.59347728	1.004736718	15	4.299841469	0.958554004	15	9.828336373	0.982179389	15	10.21683773	1.013332397	15	7.517447346	Quantified
Q99L47	Hsc70-interacting protein	St13	FUNCTION: One HIP oligomer binds the ATP	Hsc70-interacting protein O	19.95	7	7	13	371	41.62950173	5.262207031	0.994495649	13	3.649950603	1.018272247	13	2.120513105	0.992145076	13	7.686074809	0.978359664	13	7.088048313	0.993197964	13	11.85829202	0.975351024	13	3.85481298	Quantified
P68040	Receptor of activated protein C kinase 1	Rack1	FUNCTION: Scaffolding protein involved in th	Receptor of activated protei	24.92	7	7	12	317	35.0545595	7.693847656	1.043882274	12	5.695143359	1.033756642	12	3.823876337	1.016997966	12	7.025657649	0.983043511	12	3.57061038	0.918788169	12	12.510352	0.983946096	12	6.017051501	Quantified
O35375	Neuropilin-2	Nrp2	FUNCTION: High affinity receptor for semaph	Neuropilin-2 OS=Mus musc	8.27	8	8	26	931	104.5648429	5.236816406	0.997846417	26	3.315302917	0.970512763	26	3.332665074	0.97162505	26	5.515785735	1.032525246	26	5.096609979	1.021140054	26	7.622399448	1.018529893	26	4.466548602	Quantified
P50580	Proliferation-associated protein 2G4	Pa2g4	FUNCTION: May play a role in a ERBB3-regu	Proliferation-associated pro	35.28	12	12	15	394	43.67120906	6.858886719	1.016780695	15	2.487716009	1.034807348	15	4.2572447	1.047436301	15	5.325745118	0.950981136	15	4.006359047	0.97195251	15	4.844615548	0.973397945	15	5.027537119	Quantified
P39749	Flap endonuclease 1	Fen1	FUNCTION: Structure-specific nuclease with	Flap endonuclease 1 OS=M	11.9	4	4	16	378	42.28789374	8.338378906	1.045027799	16	3.471265906	1.039281518	16	4.174726944	0.91672674	16	10.03367908	0.989048337	16	6.174190176	1.008467577	16	6.99597263	1.016359965	16	7.27385946	Quantified
Q08024	Core-binding factor subunit beta	Cbfb	FUNCTION: Forms the heterodimeric comple	Core-binding factor subunit	12.3	2	2	6	187	22.01677898	5.770019531	1.03105905	6	5.20157795	0.85788419	6	8.568914103	1.05373127	6	6.285528033	0.963774936	6	4.661936851	1.195353406	6	7.381160434	1.01132581	6	9.714420869	Quantified
P21836	Acetylcholinesterase	Ache	FUNCTION: Terminates signal transduction a	Acetylcholinesterase OS=M	2.12	1	1	5	614	68.12546355	6.328613281	0.971405883	5	17.665145	0.913791157	5	6.407459356	0.972070312	5	5.535420714	1.084881567	5	13.27826588	1.195455273	5	23.50456812	0.941579113	5	8.13043507	Quantified
Q8BH61	Coagulation factor XIII A chain	F13a1	FUNCTION: Factor XIII is activated by thromt	Coagulation factor XIII A ch	2.87	2	2	11	732	83.15467966	5.922363281	1.054998341	11	3.444031552	0.98596024	11	13.68000065	0.977729436	11	8.968087305	1.036099111	11	6.174146512	1.034756732	11	14.24358581	0.976164359	11	6.621344208	Quantified
Q91YR9	Prostaglandin reductase 1	Ptgr1	FUNCTION: Functions as 15-oxo-prostagland	Prostaglandin reductase 1 (	3.04	1	1	10	329	35.53721083	7.957519531	0.96701974	10	6.453684709	0.986304499	10	4.202223045	1.023518164	10	4.750634819	1.031652583	10	5.999387884	1.043336552	10	4.641519599	0.980961689	10	4.39855207	Quantified
P09055	Integrin beta-1	Itgb1	FUNCTION: Integrins alpha-1/beta-1, alpha-2	Integrin beta-1 OS=Mus mu	11.78	7	8	12	798	88.17332437	5.935058594	1.014384599	11	2.317167141	1.015359349	11	2.91933046	1.042667224	11	3.03064221	0.998708851	11	7.8963376	0.940895135	11	3.832278921	0.983711714	11	4.173481056	Quantified
P62196	26S proteasome regulatory subunit 8	Psmc5	FUNCTION: Component of the 26S proteasor	26S proteasome regulatory	9.61	2	3	8	406	45.59705542	7.547363281	1.074635747	4	0.893648798	0.999351026	4	5.325282513	1.017231614	4	12.51660002	0.964123921	4	2.713653104	0.958148088	4	11.00800687	0.993020624	4	5.492538648	Quantified
Q9R0N0	Galactokinase	Galk1	FUNCTION: Major enzyme for galactose meta	Galactokinase OS=Mus mu	3.32	1	1	6	392	42.26845143	5.262207031	1.000509848	6	2.591313073	1.028376636	6	5.275027284	0.977940763	6	8.185846102	1.041555762	6	13.24982449	0.990884349	6	6.383417866	0.98053202	6	2.8851997	Quantified
Q922D8	C-1-tetrahydrofolate synthase, cytoplasmic	Mthfd1		C-1-tetrahydrofolate syntha	10.7	10	10	13	935	101.136042	7.137207031	1.018038396	13	5.255977024	1.009061284	13	6.546762963	1.058262815	13	7.728077178	1.034422093	13	4.434508606	0.962988356	13	7.252504652	0.969806019	13	6.610608642	Quantified
P63330	Serine/threonine-protein phosphatase 2A catalytic su	Ppp2ca	FUNCTION: PP2A is the major phosphatase	Serine/threonine-protein ph	21.68	1	5	12	309	35.58541378	5.541503906	1.012114288	3	6.248537293	1.017901872	3	4.104393534	1.059216153	3	2.167432884	0.956963564	3	2.144124569	0.908270174	3	0.195039554	1.01739732	3	11.22588799	Quantified
Q61470	Leukocyte antigen CD37	Cd37		Leukocyte antigen CD37 O	13.17	3	3	5	281	31.83151476	8.353027344	1.036577929	5	6.844861646	0.911188545	5	11.15665741	1.004602926	5	6.627117747	1.067710465	5	7.981028156	1.082214556	5	7.325703355	0.965166558	5	3.214086926	Quantified
O88543	COP9 signalosome complex subunit 3	Cops3	FUNCTION: Component of the COP9 signalo	COP9 signalosome comple	8.98	4	4	6	423	47.80098942	6.653808594	0.993342705	4	6.716057741	0.975251343	4	9.712383204	0.912323307	4	11.93210784	1.075112957	4	4.593313791	1.034171384	4	9.744616497	1.071588143	4	14.14067698	Quantified
P15530	B-cell antigen receptor complex-associated protein b	Cd79b	FUNCTION: Required in cooperation with CD	B-cell antigen receptor com	6.14	1	1	6	228	25.7099644	5.973144531	1.013566381	6	2.724353834	0.907870358	6	9.814819676	1.075574277	6	8.289818309	1.014343825	6	3.126846261	1.148000939	6	12.37106217	0.960641286	6	4.715721316	Quantified
O88662	Epithelial membrane protein 2	Emp2	FUNCTION: Functions as a key regulator of c	Epithelial membrane protei	5.23	2	2	7	172	19.73419772	7.723144531	0.936147967	7	4.082076547	0.932828867	7	5.078078994	1.158480223	7	8.732978889	0.957782135	7	4.538699777	0.979375163	7	6.660202669	0.972580873	7	5.745158259	Quantified
Q9QXY6	EH domain-containing protein 3	Ehd3	FUNCTION: ATP- and membrane-binding pro	EH domain-containing prote	16.07	2	9	15	535	60.78272504	6.455566406	0.967557803	3	0.774516373	1.027302288	3	0.499174803	0.971103131	3	6.821629251	0.991279623	3	3.431982089	1.007194004	3	2.270072081	1.03895752	3	0.783503088	Quantified
Q9D1A2	Cytosolic non-specific dipeptidase	Cndp2	FUNCTION: Hydrolyzes a variety of dipeptide	Cytosolic non-specific dipe	13.05	5	5	9	475	52.73361243	5.655761719	1.009009194	9	2.571455932	1.022240089	9	4.417458609	0.963570661	9	4.985151882	1.000872382	9	3.631132274	1.02376321	9	8.732435634	0.984410708	9	5.000334628	Quantified
Q9CZ44	NSFL1 cofactor p47	Nsfl1c	FUNCTION: Reduces the ATPase activity of	NSFL1 cofactor p47 OS=M	18.92	5	5	12	370	40.68539213	5.147949219	1.031588164	12	6.737142162	1.0009764	12	4.963355532	0.97858355	12	4.103887398	0.995445945	12	6.646375457	0.996200889	12	6.963006812	1.006805331	12	5.753338759	Quantified
P97321	Prolyl endopeptidase FAP	Fap	FUNCTION: Cell surface glycoprotein serine	Prolyl endopeptidase FAP (	11.04	6	6	8	761	87.88879371	7.078613281	0.988378439	8	3.687204395	0.977349738	8	4.402233602	0.986074506	8	4.056478466	1.007863833	8	6.430126224	1.046797281	8	7.577202275	1.012249101	8	3.206069958	Quantified
P04104	Keratin, type II cytoskeletal 1	Krt1	FUNCTION: May regulate the activity of kinas	Keratin, type II cytoskeletal	4.55	3	3	5	637	65.56531789	8.147949219	1.135984661	5	2.474116408	1.162862986	5	14.1470121	0.761396939	5	18.61299074	1.128382936	5	1.500054612	0.867451377	5	8.947790536	0.955032721	5	5.730146348	Quantified
Q9Z2D6	Methyl-CpG-binding protein 2	Mecp2	FUNCTION: Chromosomal protein that binds	Methyl-CpG-binding protein	9.3	3	3	4	484	52.27547114	9.964355469	0.967836113	4	5.45993491	0.955856486	4	1.703861637	0.884511487	4	6.959922829	1.165587523	4	3.96025407	1.200064295	4	6.190877892	0.951659966	4	2.363074929	Quantified
Q62188	Dihydropyrimidinase-related protein 3	Dpysl3	FUNCTION: Necessary for signaling by class	Dihydropyrimidinase-relatec	13.51	4	7	9	570	61.89708719	6.493652344	1.009228634	4	1.899123647	1.009928406	4	1.675175061	0.993096393	4	3.167170296	0.997856207	4	2.935209171	0.97691489	4	3.725043477	1.02554227	4	7.006238653	Quantified
P63158	High mobility group protein B1	Hmgb1	FUNCTION: Multifunctional redox sensitive pı	High mobility group protein	31.16	8	8	22	215	24.87816252	5.744628906	0.951695591	22	4.015485681	0.989315971	22	3.317209665	1.092189811	22	7.14069836	0.967797348	22	7.381680919	1.037384901	22	4.325251996	0.993851701	22	6.451625393	Quantified
Q99020	Heterogeneous nuclear ribonucleoprotein A/B	Hnrnpab	FUNCTION: Transcriptional repressor. Binds	Heterogeneous nuclear ribo	18.6	4	4	8	285	30.81225416	7.913574219	0.988906857	8	6.273592766	1.028735667	8	4.228494139	1.081408896	8	9.879925044	0.892411107	8	12.64094329	1.045738327	8	9.622591623	0.946448869	8	12.17354783	Quantified
Q9R182	Angiopoietin-related protein 3	Angptl3	FUNCTION: Acts in part as a hepatokine that	Angiopoietin-related protein	5.93	3	3	7	455	52.51046224	6.653808594	1.020982206	7	1.629086482	0.946367499	7	2.742534743	0.971193908	7	6.45224832	1.09365807	7	4.424918271	0.985410854	7	4.802251605	0.968686386	7	5.68009254	Quantified
Q99JX4	Eukaryotic translation initiation factor 3 subunit M	Eif3m	FUNCTION: Component of the eukaryotic tra	Eukaryotic translation initiat	4.81	1	1	2	374	42.48985903	5.744628906																			Identified
Q9D1G1	Ras-related protein Rab-1B	Rab1b	FUNCTION: The small GTPases Rab are key	Ras-related protein Rab-1B	59.7	4	10	16	201	22.17322474	5.731933594	0.981538174	5	13.49474216	1.035420738	5	1.070828421	0.987971092	5	5.045782839	0.989389812	5	5.975264684	0.931282445	5	3.639040905	1.039579482	5	6.143989006	Quantified
P62317	Small nuclear ribonucleoprotein Sm D2	Snrpd2	FUNCTION: Plays role in pre-mRNA splicing	Small nuclear ribonucleopro	47.46	5	5	14	118	13.51822746	9.905761719	0.963497308	14	4.720730676	0.975926146	14	4.388715768	1.028836343	14	4.865480373	1.007189226	14	6.580448197	1.074428726	14	3.58756049	1.007651415	14	3.977759705	Quantified
P02798	Metallothionein-2	Mt2	FUNCTION: Metallothioneins have a high cor	Metallothionein-2 OS=Mus	21.31	2	2	4	61	6.110212775	7.825683594	0.733734064	4	7.235218586	0.902760369	4	4.301521347	1.754382829	4	18.94271374	0.828484276	4	15.31601506	1.070248266	4	9.443377152	0.647072794	4	22.4485218	Quantified
P08226	Apolipoprotein E	Apoe	FUNCTION: APOE is an apolipoprotein, a prc	Apolipoprotein E OS=Mus r	15.11	5	5	11	311	35.84435086	5.681152344	0.971025651	11	3.487557793	1.009828804	11	8.127366448	1.045238255	11	6.968075162	0.96733639	11	7.709421464	0.989088492	11	3.777813372	1.024537651	11	5.101056917	Quantified
Q61035	Histidine--tRNA ligase, cytoplasmic	Hars1	FUNCTION: Catalyzes the ATP-dependent lig	Histidine--tRNA ligase, cytc	8.84	5	5	10	509	57.39614894	5.998535156	1.001571235	10	2.270173852	0.915920367	10	17.08416968	1.028345849	10	6.469260538	0.964749668	10	13.77350665	1.157568265	10	23.2868183	0.956315749	10	7.719523876	Quantified
Q9D6Z1	Nucleolar protein 56	Nop56	FUNCTION: Involved in the early to middle st	Nucleolar protein 56 OS=M	8.28	4	4	6	580	64.42410762	9.144042969	0.993458925	6	2.276578224	0.994160053	6	4.624222877	0.950777259	6	4.294365226	1.065206561	6	5.619113199	1.069010039	6	10.24410811	0.994216825	6	2.852391825	Quantified
P46638	Ras-related protein Rab-11B	Rab11b	FUNCTION: The small GTPases Rab are key	Ras-related protein Rab-11I	29.82	8	8	16	218	24.47448177	5.935058594	0.995827249	16	4.894207118	1.000379664	16	7.473803505	0.934246178	16	6.08389279	1.03850966	16	9.669769079	1.029458363	16	6.544705797	1.022799488	16	4.827788116	Quantified
P02088	Hemoglobin subunit beta-1	Hbb-b1	FUNCTION: Involved in oxygen transport fron	Hemoglobin subunit beta-1	12.93	1	2	27	147	15.83014835	7.649902344	0.924811358	25	6.327836452	0.944526773	25	6.239373492	0.998108682	25	3.823550231	0.98321546	25	5.194704474	1.070325327	25	6.195986671	1.078245684	25	8.043836344	Quantified
P62715	Serine/threonine-protein phosphatase 2A catalytic su	Ppp2cb	FUNCTION: PP2A can modulate the activity (	Serine/threonine-protein ph	21.68	1	5	10	309	35.55233078	5.427246094	1.04722425	1		1.086709728	1		0.990876431	1		0.966431357	1		0.874302277	1		0.996305161	1		Quantified
O09061	Proteasome subunit beta type-1	Psmb1	FUNCTION: Component of the 20S core prot	Proteasome subunit beta ty	30.42	6	6	11	240	26.35530979	7.811035156	0.982904329	11	3.825844951	1.003661532	11	2.086748156	1.058423078	11	4.021112519	1.014683087	11	3.684004928	0.968630569	11	9.26210279	0.967289993	11	3.792143938	Quantified
P28063	Proteasome subunit beta type-8	Psmb8	FUNCTION: The proteasome is a multicatalyt	Proteasome subunit beta ty	17.03	5	5	13	276	30.24055451	6.683105469	1.034815395	13	3.863724333	0.947333021	13	11.84749921	0.982220633	13	9.515444972	0.998728528	13	5.47594567	0.987143853	13	6.156822487	0.989651982	13	5.323162287	Quantified
P50516	V-type proton ATPase catalytic subunit A	Atp6v1a	FUNCTION: Catalytic subunit of the periphera	V-type proton ATPase catal	10.05	6	6	10	617	68.28255638	5.579589844	0.997882533	10	3.476329261	0.997253937	10	1.944156875	0.960937123	10	11.39313065	1.05549097	10	4.665217967	0.967267243	10	8.709535592	1.058339079	10	5.015778276	Quantified
Q01405	Protein transport protein Sec23A	Sec23a	FUNCTION: Component of the coat protein cc	Protein transport protein Se	6.93	4	4	5	765	86.10637775	7.078613281	0.983848479	4	10.33375203	1.027982486	4	5.000312052	1.046206123	4	3.462078068	0.974578387	4	0.503911651	0.921478377	4	11.61824366	1.031161097	4	5.793654001	Quantified
Q9D2M8	Ubiquitin-conjugating enzyme E2 variant 2	Ube2v2	FUNCTION: Has no ubiquitin ligase activity o	Ubiquitin-conjugating enzyr	6.9	1	1	5	145	16.35622858	8.089355469	1.026793266	5	0.690606179	0.971134646	5	4.577227403	1.08234403	5	2.795082695	1.012365138	5	5.358582258	0.931475249	5	2.628308827	0.948389811	5	3.88619537	Quantified

P60335	Poly(rC)-binding protein 1	Pcbp1	FUNCTION: Single-stranded nucleic acid bin	Poly(rC)-binding protein 1 C	19.38	3	6	12	356	37.47394517	7.093261719	0.972343689	6	3.775742474	1.032832477	6	4.162336669	0.979061939	6	6.054262556	1.004491305	6	5.350988294	0.997014923	6	4.956140953	0.978959613	6	2.488817766	Quantified
Q62418	Drebrin-like protein	Dbnl	FUNCTION: Adapter protein that binds F-actin	Drebrin-like protein OS=Mu	7.57	2	2	4	436	48.66961686	4.919433594	1.032198018	4	10.27954319	1.044784598	4	1.261054151	1.165216595	4	1.69740909	0.849201767	4	3.44559696	0.949381786	4	7.109079781	0.902871326	4	7.102637577	Quantified
Q8BG05	Heterogeneous nuclear ribonucleoprotein A3	Hnrnpa3	FUNCTION: Plays a role in cytoplasmic traffic	Heterogeneous nuclear ribo	25.59	8	8	24	379	39.62760668	9.012207031	1.07500503	24	4.118933834	1.044409017	24	3.905860179	1.047321995	24	6.023822756	0.938161792	24	5.551384436	0.89465164	24	10.92839227	0.964996496	24	6.618053269	Quantified
Q501J6	Probable ATP-dependent RNA helicase DDX17	Ddx17	FUNCTION: As an RNA helicase, unwinds RN	Probable ATP-dependent R	11.69	4	7	8	650	72.35399759	8.587402344	0.995903603	4	1.877253175	1.041986775	4	3.742250875	0.99951644	4	3.761557863	0.962070134	4	5.067340192	0.984064009	4	1.333541158	0.992897046	4	3.956019217	Quantified
Q05895	Thrombospondin-3	Thbs3	FUNCTION: Adhesive glycoprotein that media	Thrombospondin-3 OS=Mu	3.56	1	3	5	956	104.0525369	4.640136719	1.002657003	1		1.016742348	1		1.020418939	1		1.047040268	1		0.968069097	1		0.947776382	1		Quantified
O88544	COP9 signalosome complex subunit 4	Cops4	FUNCTION: Component of the COP9 signalo	COP9 signalosome comple	17.73	6	6	12	406	46.25566774	5.833496094	0.994831569	12	2.202628793	0.948072787	12	4.239582619	0.993910528	12	6.352739352	1.038514658	12	5.580522588	1.092581227	12	9.526919749	1.020891297	12	4.304813674	Quantified
Q61982	Neurogenic locus notch homolog protein 3	Notch3	FUNCTION: Functions as a receptor for mem	Neurogenic locus notch hor	1.98	4	4	6	2318	244.0844272	5.325683594	0.950664408	6	2.928951706	1.019017581	6	4.954589488	1.146659219	6	6.222374087	0.999822405	6	6.775267215	0.958758033	6	12.71282345	0.955317737	6	2.538041804	Quantified
P80315	T-complex protein 1 subunit delta	Cct4	FUNCTION: Component of the chaperonin-co	T-complex protein 1 subuni	19.85	8	8	16	539	58.02973643	8.016113281	1.022405353	16	3.969753651	0.997019338	16	4.157384981	1.03786389	16	6.451785526	1.008179555	16	9.403854538	0.976730959	16	10.43995585	0.961801678	16	3.06552242	Quantified
Q08857	Platelet glycoprotein 4	Cd36	FUNCTION: Multifunctional glycoprotein that	Platelet glycoprotein 4 OS=	8.9	4	4	10	472	52.66415408	8.353027344	0.993283323	10	3.73766543	0.962784968	10	3.945105968	1.09476066	10	4.870504404	1.02080762	10	3.618363274	1.054417626	10	15.99554123	0.938490267	10	8.562013271	Quantified
P19253	60S ribosomal protein L13a	Rpl13a	FUNCTION: Associated with ribosomes but is	60S ribosomal protein L13a	10.84	2	2	4	203	23.44933414	11.01904297	1.059530705	1		0.964772491	1		0.952460224	1		1.052332474	1		0.931634441	1		1.039691317	1		Quantified
Q05144	Ras-related C3 botulinum toxin substrate 2	Rac2	FUNCTION: Plasma membrane-associated s	Ras-related C3 botulinum to	29.17	2	6	11	192	21.42707811	7.605957031	0.995574727	3	2.932340316	1.024148532	3	12.57051639	0.996241478	3	11.95100739	0.994718676	3	19.02460364	1.044307253	3	23.76963325	0.982117075	3	0.325425084	Quantified
Q91WN1	DnaJ homolog subfamily C member 9	Dnajc9	FUNCTION: May play a role as co-chaperone	DnaJ homolog subfamily C	8.49	2	2	4	259	30.04030593	5.935058594	1.040392551	4	2.301103382	1.10233592	4	5.988601073	1.058525821	4	2.335965655	0.882511698	4	4.874179055	0.913322895	4	12.09674602	0.953113033	4	1.694567325	Quantified
Q9R1P1	Proteasome subunit beta type-3	Psmb3	FUNCTION: Component of the 20S core prote	Proteasome subunit beta ty	7.8	1	1	3	205	22.94944719	6.551269531	1.025559569	3	0.763356491	1.044911743	3	1.812962413	1.043180313	3	4.501518807	0.978242627	3	0.075846296	0.895423122	3	4.656460917	0.994594893	3	7.917421815	Quantified
P46460	Vesicle-fusing ATPase	Nsf	FUNCTION: Required for vesicle-mediated tr	Vesicle-fusing ATPase OS=	8.74	7	7	10	744	82.56095983	6.946777344	1.016055103	10	2.154425798	0.996716351	10	6.043939305	1.008140687	10	3.999147407	1.010694578	10	3.496647811	0.931442844	10	5.493178663	0.993724747	10	3.668375391	Quantified
P26231	Catenin alpha-1	Ctnna1	FUNCTION: Associates with the cytoplasmic	Catenin alpha-1 OS=Mus m	8.5	4	8	12	906	100.043556	6.227050781	0.949876386	6	2.788134134	1.023682581	6	5.896012172	0.995639151	6	2.223944446	0.997740599	6	4.499752542	1.040992752	6	4.004072346	1.026082553	6	3.359121197	Quantified
Q9JHU4	Cytoplasmic dynein 1 heavy chain 1	Dync1h1	FUNCTION: Cytoplasmic dynein 1 acts as a r	Cytoplasmic dynein 1 heav	4.65	25	25	31	4644	531.7098361	6.417480469	1.014494736	31	4.975434461	0.99642986	31	4.892428485	1.015139633	31	4.284875469	1.008972343	31	5.398363012	0.976854197	31	6.67860508	0.998645485	31	5.391412869	Quantified
P28271	Cytoplasmic aconitate hydratase	Aco1	FUNCTION: Iron sensor. Binds a 4Fe-4S clus	Cytoplasmic aconitate hydr	9.9	10	10	23	889	98.06346271	7.503417969	1.006622046	23	5.3377412	1.007418515	23	2.475778471	1.026673882	23	4.360972061	0.993451932	23	5.689455541	0.975906079	23	5.388735116	1.009261742	23	5.431818362	Quantified
Q61990	Poly(rC)-binding protein 2	Pcbp2	FUNCTION: Single-stranded nucleic acid bin	Poly(rC)-binding protein 2 C	20.44	3	6	11	362	38.19740813	6.785644531	1.046686053	5	6.701003143	0.97907739	5	10.87199407	1.059426357	5	1.750488065	1.004205048	5	4.516786537	0.958557204	5	6.622224763	0.923544775	5	4.009569717	Quantified
Q9WTI7	Unconventional myosin-Ic	Myo1c	FUNCTION: Myosins are actin-based motor n	Unconventional myosin-Ic C	4.99	5	5	7	1063	121.8678752	9.349121094	0.955046957	7	2.766324715	0.96579358	7	2.038372137	0.967038354	7	6.910320257	1.033287692	7	8.211750334	1.059406176	7	8.783059491	1.040544088	7	7.411681175	Quantified
P17710	Hexokinase-1	Hk1	FUNCTION: Catalyzes the phosphorylation of	Hexokinase-1 OS=Mus mu	9.14	9	9	10	974	108.2338074	6.800292969	1.016899186	10	4.74811847	1.008966669	10	4.12376235	0.974450875	10	3.746709247	1.014616702	10	4.233803555	0.99913538	10	3.804329132	0.988141709	10	6.564215193	Quantified
P62880	Guanine nucleotide-binding protein G(I)/G(S)/G(T) su	Gnb2	FUNCTION: Guanine nucleotide-binding prot	Guanine nucleotide-binding	21.76	4	7	13	340	37.30706097	5.998535156	1.028852818	3	0.803487859	1.046885361	3	3.020760979	1.054491792	3	1.977894141	0.948526197	3	0.356034695	0.89057063	3	11.76206391	0.994619435	3	1.777649309	Quantified
Q78PY7	Staphylococcal nuclease domain-containing protein 1	Snd1	FUNCTION: Endonuclease that mediates miR	Staphylococcal nuclease d	11.1	9	9	13	910	102.0245936	7.430175781	1.028093423	13	3.917846052	1.024314151	13	2.716109314	1.023289647	13	4.644097837	0.982461271	13	3.754543005	1.009689384	13	8.840874161	0.979851235	13	4.883105647	Quantified
P10639	Thioredoxin	Txn	FUNCTION: Participates in various redox rea	Thioredoxin OS=Mus musc	29.52	3	3	5	105	11.66764002	4.919433594	1.009170147	5	2.358306148	0.998207097	5	2.044625004	0.982869612	5	6.954851062	1.017574356	5	9.755030953	1.006310262	5	7.076575269	0.989633877	5	3.459970756	Quantified
Q58A65	C-Jun-amino-terminal kinase-interacting protein 4	Spag9	FUNCTION: The JNK-interacting protein (JIP)	C-Jun-amino-terminal kinas	2.57	2	2	4	1321	146.1288859	5.147949219	1.035860097	4	2.579605105	0.998848037	4	1.819812064	1.054190724	4	11.57869882	0.905694444	4	6.713803673	0.937408885	4	7.40118826	1.041691966	4	2.067302301	Quantified
Q8VC12	Urocanate hydratase	Uroc1		Urocanate hydratase OS=M	4.88	3	3	8	676	74.54255057	7.532714844	0.981256104	8	2.527756758	0.97826297	8	6.519708639	0.96515809	8	3.023878625	1.022465887	8	7.956050119	0.978313456	8	5.613607191	1.046262828	8	3.870886978	Quantified
Q91V41	Ras-related protein Rab-14	Rab14	FUNCTION: Regulates, together with its guar	Ras-related protein Rab-14	19.53	3	4	11	215	23.88192868	6.214355469	0.985498863	6	8.111058641	0.970185758	6	1.837283161	0.979421931	6	3.323260781	1.045909971	6	4.03595823	1.017133271	6	3.084085732	0.989029064	6	6.310829194	Quantified
P11152	Lipoprotein lipase	Lpl	FUNCTION: Key enzyme in triglyceride metal	Lipoprotein lipase OS=Mus	12.45	4	4	5	474	53.07579762	7.869628906	1.00398806	5	5.877862105	1.030849955	5	7.849788745	0.983430115	5	6.001191016	1.007796955	5	3.05117226	0.990727842	5	6.033222914	0.958690023	5	1.921078402	Quantified
Q9QZE5	Coatomer subunit gamma-1	Copg1	FUNCTION: The coatomer is a cytosolic prot	Coatomer subunit gamma-1	4	3	3	5	874	97.45037032	5.351074219	1.06089214	5	4.148848244	1.008631469	5	1.467738727	1.068925185	5	6.467146193	0.965870842	5	3.347004841	0.929703811	5	10.39940481	0.972111098	5	6.095861485	Quantified
Q99NB9	Splicing factor 3B subunit 1	Sf3b1	FUNCTION: Involved in pre-mRNA splicing as	Splicing factor 3B subunit 1	7.67	9	9	14	1304	145.7240846	7.093261719	1.00694203	14	3.244079117	1.001173541	14	2.531549731	0.98929366	14	4.158244546	1.041778445	14	4.042509019	0.980778785	14	4.891481396	0.997719325	14	3.344548487	Quantified
Q9EQU5	Protein SET	Set	FUNCTION: Multitasking protein, involved in a	Protein SET OS=Mus musc	17.65	5	5	9	289	33.35767498	4.322753906	0.999982454	9	4.965972898	1.015972628	9	3.656669704	1.114230965	9	8.571066373	0.867923626	9	7.196986006	1.074537677	9	5.466170514	0.894941924	9	7.181981377	Quantified
P00493	Hypoxanthine-guanine phosphoribosyltransferase	Hprt1	FUNCTION: Converts guanine to guanosine r	Hypoxanthine-guanine pho	11.93	2	2	3	218	24.55458896	6.683105469	0.962483722	3	0.397153659	1.050399072	3	0.946334582	0.96613623	3	4.39219654	0.98977951	3	7.384690493	1.087566171	3	7.777602077	0.98419092	3	2.092918383	Quantified
P42208	Septin-2	Septin2	FUNCTION: Filament-forming cytoskeletal G1	Septin-2 OS=Mus musculus	16.62	5	5	7	361	41.4992778	6.551269531	1.037656698	7	2.637298034	0.9822925	7	9.075133656	1.048839269	7	9.46177394	0.978307142	7	4.543824052	0.964918506	7	7.427960927	0.935483039	7	4.023989742	Quantified
Q91YI0	Argininosuccinate lyase	Asl		Argininosuccinate lyase OS	15.95	6	6	12	464	51.70657802	6.961425781	1.004158446	12	2.889520149	1.042203594	12	4.681911014	0.943607877	12	5.853295308	0.965111988	12	5.41051094	1.010814327	12	7.485570615	1.033062789	12	6.130696431	Quantified
Q8CFG9	Complement C1r-B subcomponent	C1rb	FUNCTION: C1r B chain is a serine protease	Complement C1r-B subcom	5.1	4	4	8	706	79.8845765	5.808105469	0.983156943	8	4.166090188	0.993293834	8	4.026095472	1.038785784	8	6.899965571	1.015856338	8	3.54742227	0.966687124	8	9.051194575	0.980758598	8	4.954970221	Quantified
Q91WQ3	Tyrosine--tRNA ligase, cytoplasmic	Yars1	FUNCTION: Catalyzes the attachment of tyro	Tyrosine--tRNA ligase, cyto	15.91	9	9	12	528	59.06808256	7.005371094	1.035042029	12	3.422785087	1.003964246	12	2.561591837	1.015738201	12	3.224035021	0.990858233	12	4.84309258	0.992975922	12	4.231912889	0.972637364	12	2.879582848	Quantified
Q8BKG3	Inactive tyrosine-protein kinase 7	Ptk7	FUNCTION: Inactive tyrosine kinase involved	Inactive tyrosine-protein kin	1.04	1	1	4	1062	117.4573965	6.844238281	1.052401866	4	5.657928978	1.044969208	4	5.623974339	1.019055908	4	0.816969946	0.949457708	4	4.913480976	0.932700041	4	8.866785985	0.980415591	4	1.089514615	Quantified
O70475	UDP-glucose 6-dehydrogenase	Ugdh	FUNCTION: Catalyzes the formation of UDP-	UDP-glucose 6-dehydroger	21.1	7	7	10	493	54.79726962	7.562011719	1.048876519	10	4.877112481	1.04604127	10	5.370213835	1.011628411	10	6.081618472	0.961045708	10	7.932479706	0.964569754	10	5.661973916	0.994697175	10	8.020641211	Quantified
Q9CQI3	Glia maturation factor beta	Gmfb	FUNCTION: This protein causes differentiatio	Glia maturation factor beta	32.39	3	4	6	142	16.7124345	5.160644531	0.959660946	5	7.551131632	0.860582972	5	19.40338422	1.114459808	5	22.30583631	1.002982975	5	7.074887348	1.151312146	5	3.203283228	1.005137608	5	10.61704658	Quantified
P09103	Protein disulfide-isomerase	P4hb	FUNCTION: This multifunctional protein catal	Protein disulfide-isomerase	16.9	9	9	16	509	57.02277114	4.881347656	0.980273046	16	3.664676692	1.018644436	16	10.45278606	0.997838079	16	4.296711443	0.982224313	16	12.74581485	1.029883945	16	10.14776465	1.046104034	16	7.40822151	Quantified
Q6P1B1	Xaa-Pro aminopeptidase 1	Xpnpep1	FUNCTION: Contributes to the degradation of	Xaa-Pro aminopeptidase 1	13	6	6	9	623	69.54701368	5.541503906	1.029595155	9	9.033679714	1.022286395	9	10.07747147	0.923358703	9	12.04821098	1.010589951	9	7.89745658	0.966028897	9	7.851390801	1.052214425	9	5.909481425	Quantified
Q00519	Xanthine dehydrogenase/oxidase	Xdh	FUNCTION: Key enzyme in purine degradatio	Xanthine dehydrogenase/o	5.92	7	8	11	1335	146.4680042	7.562011719	1.047317419	10	4.006312631	1.029702381	10	4.618006513	1.015192506	10	3.272019716	0.996653535	10	1.804594956	0.979538239	10	6.196345211	0.965419329	10	6.346116838	Quantified
Q7TNV0	Protein DEK	Dek	FUNCTION: Involved in chromatin organizatio	Protein DEK OS=Mus musc	7.37	3	3	12	380	43.13204338	6.858886719	0.99979886	12	7.475983105	0.956684057	12	8.148572676	1.059054665	12	12.98665782	0.99967709	12	5.722853716	1.037404738	12	11.40943923	0.945399259	12	12.80200159	Quantified
P84084	ADP-ribosylation factor 5	Arf5	FUNCTION: GTP-binding protein involved in p	ADP-ribosylation factor 5 O	27.22	2	4	8	180	20.5165808	6.785644531	1.066536698	4	4.098902368	0.962609293	4	8.885729916	0.981951158	4	2.514502099	1.006388253	4	4.074759038	0.916891691	4	4.749566722	1.042349299	4	2.964464809	Quantified
Q9WV32	Actin-related protein 2/3 complex subunit 1B	Arpc1b	FUNCTION: Component of the Arp2/3 comple	Actin-related protein 2/3 co	23.92	7	7	15	372	41.03745967	8.353027344	0.947958677	15	5.108428303	1.025941313	15	2.527724575	1.053374636	15	4.518596837	1.013916579	15	3.634684845	1.02645915	15	7.778310847	0.957530041	15	2.904898097	Quantified
P28828	Receptor-type tyrosine-protein phosphatase mu	Ptprm	FUNCTION: Involved in cell-cell adhesion thr	Receptor-type tyrosine-prot	3.1	4	4	6	1452	163.5493347	6.683105469	0.969274236	6	6.031854061	1.077490647	6	2.827975826	1.0005786	6	9.085275237	0.914587109	6	10.38753997	1.08594503	6	7.961995812	0.987444321	6	4.566998708	Quantified
Q9WUA3	ATP-dependent 6-phosphofructokinase, platelet type	Pfkp	FUNCTION: Catalyzes the phosphorylation of	ATP-dependent 6-phosphot	5.99	2	4	5	784	85.40032356	7.107910156	1.011681041	2	9.573160011	0.938028676	2	8.828406859	1.041483694	2	6.940772157	0.967632692	2	0.695833933	0.920084487	2	12.08082585	1.099708781	2	13.33452116	Quantified
Q60973	Histone-binding protein RBBP7	Rbbp7	FUNCTION: Core histone-binding subunit tha	Histone-binding protein RBI	15.76	2	5	10	425	47.76017102	5.046386719	1.003114025	3	0.521770689	0.913041634	3	7.942309344	0.950600264	3	2.768611609	1.064403715	3	5.26898469	1.114147746	3	18.26311716	1.000760581	3	2.59277989	Quantified
Q8BH35	Complement component C8 beta chain	C8b	FUNCTION: Constituent of the membrane att	Complement component C8	5.09	2	2	4	589	66.18618036	7.767089844	0.974846225	4	1.277057148	0.92812385	4	7.423549299	1.048215203	4	0.73414523	1.012195096	4	1.246496068	1.010467684	4	12.83736061	0.974045398	4	1.957624249	Quantified
O55103	Periaxin	Prx	FUNCTION: Scaffolding protein that functions	Periaxin OS=Mus musculus	4.53	6	6	7	1391	147.5950666	8.104003906	0.925480191	7	9.183574106	0.905798723	7	5.089986002	1.06026597	7	4.926491522	1.038010198	7	6.999011972	1.159961762	7	9.165087675	0.995330769	7	4.728143757	Quantified
P62814	V-type proton ATPase subunit B, brain isoform	Atp6v1b2	FUNCTION: Non-catalytic subunit of the perip	V-type proton ATPase subu	9.2	5	5	7	511	56.51485043	5.808105469	0.945791715	7	2.170995313	0.979510052	7	1.199003351	0.956015336	7	4.473692369	1.108216691	7	7.267460532	1.048085262	7	8.117420459	1.02580068	7	5.906426883	Quantified
P63085	Mitogen-activated protein kinase 1	Mapk1	FUNCTION: Serine/threonine kinase which ac	Mitogen-activated protein k	18.99	6	8	11	358	41.24918206	6.976074219	0.996727661	8	1.514193438	1.037292936	8	2.211413497	0.988272299	8	7.576621758	0.977244781	8	4.837645525	0.953241917	8	3.481746637	0.997971402	8	4.237727217	Quantified
O08810	116 kDa U5 small nuclear ribonucleoprotein compone	Eftud2	FUNCTION: Required for pre-mRNA splicing	116 kDa U5 small nuclear r	11.64	8	9	13	971	109.2913082	4.995605469	0.966717321	9	1.195283181	1.00405702	9	6.743528405	0.968438137	9	6.005948026	1.051738065	9	7.838481554	1.02024761	9	12.02337505	1.010565206	9	7.224143523	Quantified
Q04750	DNA topoisomerase 1	Top1	FUNCTION: Releases the supercoiling and to	DNA topoisomerase 1 OS=	12.39	9	9	12	767	90.81890078	9.334472656	0.894275182	12	4.993148078	0.906491961	12	3.472727696	0.905922954	12	5.564282357	1.161415818	12	9.26220871	1.064191513	12	7.883260125	1.121425913	12	3.562071295	Quantified
P29341	Polyadenylate-binding protein 1	Pabpc1	FUNCTION: Binds the poly(A) tail of mRNA, i	Polyadenylate-binding prote	13.05	8	8	11	636	70.62587282	9.495605469	1.007303823	11	5.851766259	0.984814387	11	10.33358784	0.995971716	11	3.951660731	1.002765985	11	5.992810154	1.014172783	11	10.79988817	0.991634916	11	8.931466343	Quantified
Q3TTY5	Keratin, type II cytoskeletal 2 epidermal	Krt2	FUNCTION: Probably contributes to terminal	Keratin, type II cytoskeletal	4.53	3	3	7	707	70.87984225	8.060058594	1.126724678	7	5.103086418	1.158710052	7	2.648073608	0.78516766	7	12.88827368	1.140993064	7	15.04096531	0.933804651	7	23.24406628	0.872234879	7	6.376977038	Quantified
Q8R001	Microtubule-associated protein RP/EB family membe	Mapre2	FUNCTION: May be involved in microtubule p	Microtubule-associated pro	28.22	9	10	13	326	36.92313156	5.376464844	1.011239641	11	1.777587598	0.965879933	11	5.334771018	0.988141202	11	4.613705391	1.00630089	11	7.056240834	0.992167669	11	5.478816532	0.975519831	11	2.548191167	Quantified
P63323	40S ribosomal protein S12	Rps12		40S ribosomal protein S12	30.3	3	3	5	132	14.51555719	7.239746094	1	5	3.533609708	1.005243973	5	6.359261374	1.028459884	5	2.153478694	0.962322882	5	3.267429042	1.046441042	5	11.58994793	0.987131023	5	7.289346741	Quantified
P05063	Fructose-bisphosphate aldolase C	Aldoc		Fructose-bisphosphate aldo	18.18	3	6	13	363	39.37023706	7.122558594	0.94558829	5	3.581952991	1.00811306	5	4.753655629	0.97468493	5	5.393413131	0.987756034	5	3.453887441	1.029721815	5	1.031326601	1.095492552	5	5.984222923	Quantified
Q61699	Heat shock protein 105 kDa	Hsph1	FUNCTION: Acts as a nucleotide-exchange fa	Heat shock protein 105 kDa	4.2	3	3	4	858	96.34635802	5.528808594	1.024486558	4	3.968986373	1.023818971	4	7.540238545	0.99034816	4	3.143265314	1.015402056	4	2.355481714	0.951870233	4	7.35565181	1.006076855	4	8.605806901	Quantified
P63005	Platelet-activating factor acetylhydrolase IB subunit b	Pafah1b1	FUNCTION: Positively regulates the activity c	Platelet-activating factor ac	28.54	9	10	17	410	46.64013185	7.371582031	1.008067014	16	2.704726289	1.044217914	16	3.642723799	0.99897767	16	7.185623427	0.976216533	16	5.914877429	0.968410338	16	7.72244646	0.997174697	16	4.522262495	Quantified
P30416	Peptidyl-prolyl cis-trans isomerase FKBP4	Fkbp4	FUNCTION: Immunophilin protein with PPIas	Peptidyl-prolyl cis-trans iso	19.21	8	8	12	458	51.53993824	5.719238281	0.994152614	12	3.252487281	1.022654779	12	3.85613396	1.059490747	12	4.342654755	0.972235904	12	4.925573338	0.992279673	12	11.05353181	0.975127602	12	3.401793622	Quantified
Q9R0P3	S-formylglutathione hydrolase	Esd	FUNCTION: Serine hydrolase involved in the	S-formylglutathione hydrola	12.41	4	4	9	282	31.29931772	7.122558594	1.000599028	9	8.327406035	1.017331733	9	3.146153055	1.057201562	9	13.24083132	0.936371755	9	11.26412084	0.963168382	9	6.293968456	0.982253343	9	5.037508281	Quantified
P47738	Aldehyde dehydrogenase, mitochondrial	Aldh2	FUNCTION: Is capable of converting retinald	Aldehyde dehydrogenase, r	15.61	6	7	10	519	56.5017545	7.620605469	1.009204854	8	1.440417934	1.028666821	8	5.571412275	1.012277602	8	2.663911065	0.99782368	8	6.777787973	0.938356828	8	3.744130176	1.002919645	8	4.724637382	Quantified
Q6P8X1	Sorting nexin-6	Snx6	FUNCTION: Involved in several stages of intr	Sorting nexin-6 OS=Mus m	15.76	6	6	7	406	46.61975815	6.163574219	0.998646773	6	4.299179719	1.021522625	6	4.634157622	1.03835101	6	3.325976123	0.966341176	6	3.686630761	0.981883404	6	4.645909513	0.969007182	6	8.875774766	Quantified
Q8VED5	Keratin, type II cytoskeletal 79	Krt79		Keratin, type II cytoskeletal	3.58	1	2	5	531	57.51682141	7.693847656	1.155960253	4	1.123685789	1.263989665	4	4.468729565	0.74010513	4	9.513117059	1.163938087	4	3.726112514	0.786298073	4	7.632970391	0.805709051	4	0.450527169	Quantified
D3YXG0	Hemicentin-1	Hmcn1	FUNCTION: Promotes cleavage furrow matur	Hemicentin-1 OS=Mus mus	1.14	6	6	8	5634	611.1744146	6.639160156	0.949705967	8	8.73751783	0.983253298	8	7.188566962	1.010027959	8	10.00228735	0.981826791	8	6.735440868	0.985065616	8	2.91943088	1.03112282	8	8.600518435	Quantified
P54822	Adenylosuccinate lyase	Adsl	FUNCTION: Catalyzes two non-sequential ste	Adenylosuccinate lyase OS	7.64	4	4	8	484	54.83094382	7.269042969	1.01507693	8	2.432983984	1.073552891	8	2.870784675	1.001221083	8	3.730683795	0.958670357	8	3.493516036	0.936824777	8	5.660858594	0.963771927	8	9.708754962	Quantified
Q6IRU2	Tropomyosin alpha-4 chain	Tpm4	FUNCTION: Binds to actin filaments in muscl	Tropomyosin alpha-4 chain	33.06	9	10	21	248	28.4504343	4.678222656	0.949986747	18	6.81025116	0.909281058	18	7.71563569	1.006192227	18	2.915636712	1.116568475	18	8.644814048	1.212616344	18	10.48564949	0.928889449	18	4.075060571	Quantified
Q8R016	Bleomycin hydrolase	Blmh	FUNCTION: The normal physiological role of	Bleomycin hydrolase OS=M	9.67	4	4	4	455	52.4769074	6.480957031	1.017480184	4	4.673199807	1.055222023	4	1.801394625	1.060331575	4	10.57498219	0.920518942	4	6.983476026	0.931586241	4	2.664580143	0.997507216	4	4.403873322	Quantified
P35700	Peroxiredoxin-1	Prdx1	FUNCTION: Thiol-specific peroxidase that ca	Peroxiredoxin-1 OS=Mus m	39.2	6	7	12	199	22.16235198	8.118652344	1.027075347	11	2.568285251	1.039483465	11	3.59731287	1.062340698	11	5.949324092	0.96077096	11	4.878411219	0.931344109	11	9.242516792	0.980868709	11	3.364437094	Quantified
P26350	Prothymosin alpha	Ptma	FUNCTION: Prothymosin alpha may mediate	Prothymosin alpha OS=Mu	26.13	3	3	5	111	12.24705328	3.789550781	1.038797971	5	4.132920472	0.701621704	5	7.222363393	1.343602222	5	6.521466899	1.094886146	5	2.026064957	0.880193623	5	3.005046945	0.836189978	5	4.703607674	Quantified
P14602	Heat shock protein beta-1	Hspb1	FUNCTION: Small heat shock protein which f	Heat shock protein beta-1 C	20.57	4	4	8	209	22.99972169	6.551269531	0.984458705	8	2.697148643	0.969557035	8	2.989092349	0.993143321	8	4.412370417	1.049875195	8	6.44249804	0.999027991	8	5.770565255	0.998787234	8	4.60502937	Quantified
Q6WVG3	BTB/POZ domain-containing protein KCTD12	Kctd12	FUNCTION: Auxiliary subunit of GABA-B rec	BTB/POZ domain-containin	19.27	5	5	15	327	35.86977772	5.808105469	1.040861936	15	7.121678741	1.021258432	15	8.276815723	1.042950315	15	8.535743086	0.933324466	15	8.846293863	0.903574962	15	15.66286785	1.056993438	15	11.34675357	Quantified
P42669	Transcriptional activator protein Pur-alpha	Pura	FUNCTION: This is a probable transcription a	Transcriptional activator pro	13.08	2	3	5	321	34.86238816	6.442871094	1.001410984	4	3.071432342	1.033394019	4	2.681599823	1.015989112	4	1.562990495	1.008034651	4	4.82524838	1.002184022	4	8.615503322	0.96663265	4	0.598722464	Quantified
O88569	Heterogeneous nuclear ribonucleoproteins A2/B1	Hnrnpa2b1	FUNCTION: Heterogeneous nuclear ribonucle	Heterogeneous nuclear ribo	28.61	8	9	19	353	37.37971756	8.953613281	0.97279005	16	6.937202489	0.982679133	16	4.889348109	0.992583346	16	6.494159468	1.004541254	16	3.985795769	0.974479246	16	7.614130951	1.033015824	16	10.24515637	Quantified
Q9D0R2	Threonine--tRNA ligase 1, cytoplasmic	Tars1	FUNCTION: Catalyzes the attachment of thre	Threonine--tRNA ligase 1, c	12.19	7	8	9	722	83.30285296	7.356933594	1.016712692	8	3.318526772	1.030140079	8	2.439440166	0.964043164	8	4.116466954	1.008736755	8	7.244757126	0.970236882	8	4.727995785	0.992354493	8	4.366060506	Quantified
O55131	Septin-7	Septin7	FUNCTION: Filament-forming cytoskeletal G1	Septin-7 OS=Mus musculus	27.52	12	14	21	436	50.5179045	8.572753906	1.012859436	16	4.036568371	1.011410049	16	5.900613431	1.038195911	16	5.775520371	0.972311352	16	8.381054862	0.96579327	16	8.491866867	0.996288818	16	6.125084969	Quantified
P29699	Alpha-2-HS-glycoprotein	Ahsg	FUNCTION: Probably involved in differentiati	Alpha-2-HS-glycoprotein O	2.03	1	1	18	345	37.30168266	6.507324219	0.948462136	18	4.787656461	0.967135771	18	4.896374626	1.160843847	18	6.110616877	0.959369235	18	3.57921919	0.961449349	18	5.759868584	0.96147937	18	5.381878747	Quantified
Q8VCC9	Spondin-1	Spon1	FUNCTION: Cell adhesion protein that promo	Spondin-1 OS=Mus muscul	8.18	7	7	10	807	90.7614256	6.023925781	1.026794614	10	5.266932955	1.02248224	10	2.12479098	0.986845741	10	7.452448819	1.001098576	10	5.544162241	0.949040543	10	10.84336587	0.996092495	10	2.319384404	Quantified
Q9CZU6	Citrate synthase, mitochondrial	Cs		Citrate synthase, mitochon	13.15	6	6	9	464	51.70338534	8.572753906	0.967510983	9	6.965901741	1.025240716	9	7.079944072	0.999453791	9	8.366173156	0.998863998	9	4.121093532	1.016778338	9	17.20641611	0.986095599	9	5.230408907	Quantified
Q6PDN3	Myosin light chain kinase, smooth muscle	Mylk	FUNCTION: Calcium/calmodulin-dependent m	Myosin light chain kinase, s	2.83	6	6	9	1941	212.7920666	6.252441406	1.009145271	9	7.304797138	0.997756674	9	6.918348446	1.027703944	9	5.614976634	0.986287048	9	2.777927821	0.963556528	9	5.499002662	1.004720988	9	5.624049321	Quantified
Q61555	Fibrillin-2	Fbn2	FUNCTION: [Fibrillin-2]: Fibrillins are structur	Fibrillin-2 OS=Mus muscul	2.3	6	6	8	2907	313.6014295	4.843261719	0.965494939	8	2.359063816	1.015490922	8	5.992652363	1.142069597	8	6.656393907	0.940551311	8	5.656379707	0.951016092	8	12.03292365	1.014568097	8	1.729745297	Quantified
Q61655	ATP-dependent RNA helicase DDX19A	Ddx19a	FUNCTION: ATP-dependent RNA helicase in	ATP-dependent RNA helica	4.18	2	2	7	478	53.89879758	6.668457031	1.031668191	7	2.761070453	0.936300529	7	5.760425402	1.098574055	7	6.126250826	0.982657726	7	3.795036006	0.977740334	7	8.907525999	0.943044322	7	5.055460311	Quantified
Q99KP6	Pre-mRNA-processing factor 19	Prpf19	FUNCTION: [Isoform 1]: Ubiquitin-protein liga	Pre-mRNA-processing facto	14.68	5	5	5	504	55.2043885	6.609863281	0.974923003	5	1.972913735	0.985538482	5	2.758591851	1.005178206	5	3.767838136	1.050733836	5	4.39761408	0.959993271	5	6.075084979	0.983010692	5	4.479872293	Quantified
P11103	Poly [ADP-ribose] polymerase 1	Parp1	FUNCTION: Poly-ADP-ribosyltransferase that	Poly [ADP-ribose] polymera	14.91	12	12	21	1013	113.028375	8.953613281	1.012470284	21	3.429413198	0.995817223	21	3.353301298	1.055477626	21	6.977406006	0.981639952	21	6.382409584	0.956334849	21	6.929234262	0.97362498	21	6.642682064	Quantified
P55264	Adenosine kinase	Adk	FUNCTION: ATP dependent phosphorylation	Adenosine kinase OS=Mus	11.36	4	4	7	361	40.12349061	6.214355469	1.017922398	7	6.480365877	1.02611592	7	2.067206757	1.043534949	7	11.6946338	0.969583965	7	9.432189146	0.935481847	7	6.170359575	1.008935234	7	5.846315174	Quantified
Q9JHH6	Carboxypeptidase B2	Cpb2	FUNCTION: Cleaves C-terminal arginine or ly	Carboxypeptidase B2 OS=M	9.72	3	3	10	422	48.83963474	7.972167969	1.060549082	10	4.999177433	1.071730982	10	1.808511089	0.976801324	10	3.532557937	0.994495496	10	4.388375086	0.913662704	10	4.003812275	0.979730056	10	4.315272024	Quantified
Q922R8	Protein disulfide-isomerase A6	Pdia6	FUNCTION: May function as a chaperone tha	Protein disulfide-isomerase	14.55	6	6	8	440	48.0702932	5.135253906	0.935629347	8	8.329042156	0.913296363	8	6.95380078	0.936521711	8	9.533406841	1.100826393	8	7.617564759	1.160953243	8	6.562116694	1.029670929	8	5.888995295	Quantified
Q11011	Puromycin-sensitive aminopeptidase	Npepps	FUNCTION: Aminopeptidase with broad subs	Puromycin-sensitive amino	7.07	7	7	8	920	103.2598415	5.896972656	1.031377731	8	2.19754452	1.018188181	8	2.000018341	0.992424514	8	4.163545	1.007581899	8	1.690515361	0.985587054	8	4.751383255	1.001473584	8	4.302565248	Quantified
Q9JLV5	Cullin-3	Cul3	FUNCTION: Core component of multiple culli	Cullin-3 OS=Mus musculus	4.95	4	4	5	768	88.89141609	8.455566406	0.990842871	5	1.052438868	0.983441445	5	5.059314422	1.004936318	5	5.04355602	0.981755766	5	1.996054758	0.992466408	5	3.071357286	1.039377181	5	6.888022512	Quantified
P53994	Ras-related protein Rab-2A	Rab2a	FUNCTION: Required for protein transport fro	Ras-related protein Rab-2A	16.51	3	3	5	212	23.53275101	6.536621094	0.988699475	5	1.730413029	0.990104103	5	4.006583689	0.991936288	5	5.665907853	0.99774111	5	5.500609267	1.067970909	5	3.319484387	0.984713792	5	2.135984804	Quantified
P17751	Triosephosphate isomerase	Tpi1	FUNCTION: Triosephosphate isomerase is ar	Triosephosphate isomerase	13.25	3	3	4	249	26.6957674	7.298339844	1.041900711	4	1.357472834	1.068620157	4	9.425572747	1.035773201	4	21.34378957	0.968282439	4	14.97375648	0.903735275	4	19.79945802	0.886236545	4	5.716510922	Quantified
P50396	Rab GDP dissociation inhibitor alpha	Gdi1	FUNCTION: Regulates the GDP/GTP exchan	Rab GDP dissociation inhib	17.23	2	6	10	447	50.48910992	5.084472656	0.890181539	3	20.77449279	0.828414114	3	13.18617234	0.898844075	3	7.348751336	1.160275181	3	19.52221928	1.019120728	3	14.84121495	1.092885305	3	7.0261148	Quantified
Q9CS84	Neurexin-1	Nrxn1	FUNCTION: Cell surface protein involved in c	Neurexin-1 OS=Mus muscu	4.16	5	5	7	1514	166.0633446	6.087402344	1.004646414	7	5.592464386	0.919261349	7	5.84140675	0.931143022	7	10.78767809	1.072071166	7	6.185178304	1.14105777	7	14.95882098	0.995632538	7	6.2533453	Quantified
Q8VED9	Galectin-related protein	Lgalsl	FUNCTION: Does not bind lactose, and may	Galectin-related protein OS	6.98	1	1	2	172	18.94364224	5.351074219	1.020087348	2	1.176870206	0.974861986	2	0.700920152	0.871727598	2	1.950262691	1.053373834	2	4.792715792	1.100754329	2	3.323430063	1.057614028	2	4.154499103	Quantified
P12382	ATP-dependent 6-phosphofructokinase, liver type	Pfkl	FUNCTION: Catalyzes the phosphorylation of	ATP-dependent 6-phosphot	6.03	2	4	6	780	85.30541439	7.166503906	0.982840087	3	3.77346831	0.969127109	3	0.643276859	1.004992348	3	2.943992628	1.009437206	3	2.305058233	1.070124622	3	0.369812857	0.989095517	3	3.918706734	Quantified
P14211	Calreticulin	Calr	FUNCTION: Calcium-binding chaperone that	Calreticulin OS=Mus musc	26.44	10	10	17	416	47.96486891	4.487792969	0.943786841	17	6.798411607	1.0024679	17	7.807086706	0.936826076	17	5.187916708	1.058224739	17	6.666732487	1.044151546	17	4.565066088	1.063001448	17	6.826630617	Quantified
Q99KI0	Aconitate hydratase, mitochondrial	Aco2	FUNCTION: Catalyzes the isomerization of ci	Aconitate hydratase, mitoch	10.26	8	8	9	780	85.41002386	7.928222656	1.010536784	9	2.367699385	1.008608644	9	4.221608315	0.952664459	9	2.926501833	1.012760759	9	3.21597532	1.010429355	9	7.244809479	1.002326862	9	3.489038816	Quantified
Q8R1B4	Eukaryotic translation initiation factor 3 subunit C	Eif3c	FUNCTION: Component of the eukaryotic tra	Eukaryotic translation initia	5.93	4	4	5	911	105.4647663	5.782714844	0.988734118	5	6.93373483	1.011653505	5	2.176343312	1.054691558	5	4.124351069	1.033206901	5	5.204643658	0.903519408	5	3.113024637	0.970236417	5	0.967189957	Quantified
Q8CI94	Glycogen phosphorylase, brain form	Pygb	FUNCTION: Glycogen phosphorylase that reg	Glycogen phosphorylase, b	10.08	5	9	17	843	96.6684762	6.727050781	1.015690726	8	1.269453558	0.991270229	8	7.234684433	0.962934153	8	4.687358895	1.039480376	8	9.565526507	0.975957818	8	11.03753287	1.002953576	8	2.001815917	Quantified
Q9D662	Protein transport protein Sec23B	Sec23b	FUNCTION: Component of the coat protein c	Protein transport protein Se	4.69	3	3	5	767	86.38154841	6.961425781	1.085436982	3	0.865037559	0.966571522	3	0.399433082	1.004347903	3	0.236547843	1.00147304	3	4.080032141	1.005328192	3	6.163003002	0.976748441	3	3.217988549	Quantified
Q9WUM4	Coronin-1C	Coro1c	FUNCTION: Plays a role in directed cell migr	Coronin-1C OS=Mus musc	19.2	8	8	14	474	53.08696956	7.078613281	1.026561469	14	2.773279438	1.024935697	14	2.970192758	1.018313046	14	3.595774614	0.989589189	14	7.247598271	0.930593136	14	8.375882962	0.9738907	14	4.374486926	Quantified
P14438	H-2 class II histocompatibility antigen, A-U alpha cha	H2-Aa		H-2 class II histocompatibi	11.89	2	2	4	227	25.1196795	4.932128906	1.003548961	4	1.965538931	0.94012997	4	7.115746799	1.030816497	4	1.0967237	1.010881873	4	3.874834472	1.064495858	4	6.455706476	0.995507742	4	2.281753997	Quantified
Q8BXK9	Chloride intracellular channel protein 5	Clic5	FUNCTION: Required for normal hearing (By	Chloride intracellular chann	22.71	3	3	3	251	28.26941635	5.935058594	0.978470884	3	2.240667115	0.995418574	3	6.7894651	0.977293574	3	23.51182416	0.986975017	3	6.347296006	1.005857596	3	1.443474514	0.988945442	3	5.270111296	Quantified
G3X987	GTPase IMAP family member 9	Gimap9		GTPase IMAP family memb	8.59	1	2	2	291	33.2771059	6.565917969	1.029030685	1		1.043219357	1		1.007400821	1		0.940169595	1		1.014718672	1		0.986930269	1		Quantified
O08539	Myc box-dependent-interacting protein 1	Bin1	FUNCTION: Is a key player in the control of p	Myc box-dependent-interac	14.8	9	9	14	588	64.43024657	5.033691406	0.9761143	14	7.234175842	1.052404538	14	4.714623983	1.078665525	14	9.978336066	0.953544842	14	3.687941786	0.945087804	14	7.083177093	0.956882783	14	5.40682703	Quantified
Q8BFY9	Transportin-1	Tnpo1	FUNCTION: Functions in nuclear protein impo	Transportin-1 OS=Mus mus	5.23	3	4	7	898	102.2908019	4.982910156	1.020167565	6	2.978739403	0.93936394	6	2.199666044	1.013917273	6	3.327457229	0.987949475	6	4.788541951	1.001901497	6	8.313171192	1.035293703	6	15.50684088	Quantified
P61164	Alpha-centractin	Actr1a	FUNCTION: Component of a multi-subunit co	Alpha-centractin OS=Mus r	12.23	3	4	12	376	42.58690748	6.639160156	1.014603828	6	4.406198137	0.984249882	6	3.735352916	1.021092472	6	1.532129066	1.032274063	6	5.655094508	1.001494115	6	10.13512543	0.965036439	6	3.826510865	Quantified
A6X935	Inter alpha-trypsin inhibitor, heavy chain 4	Itih4	FUNCTION: Type II acute-phase protein (APF	Inter alpha-trypsin inhibitor,	2.76	3	3	12	942	104.5939263	6.404785156	0.949209703	12	7.311451455	1.093816242	12	3.317942098	1.058584352	12	4.166302367	0.873413195	12	6.390871694	1.039494139	12	5.796297901	0.96026303	12	9.572149579	Quantified
Q8CIH5	1-phosphatidylinositol 4,5-bisphosphate phosphodies	Plcg2	FUNCTION: The production of the second me	1-phosphatidylinositol 4,5-b	4.58	4	4	5	1265	147.4981396	6.770996094	1.001513	5	2.951938033	1.062088845	5	1.753745848	1.019100117	5	3.107514072	0.961577346	5	5.743509629	0.971617154	5	4.554297481	1.003665721	5	11.2296123	Quantified
P62320	Small nuclear ribonucleoprotein Sm D3	Snrpd3	FUNCTION: Plays role in pre-mRNA splicing	Small nuclear ribonucleopro	24.6	2	2	3	126	13.9073049	10.31591797	0.979988135	3	4.064296841	0.956957816	3	3.93904146	1.020039118	3	7.760569051	1.031545263	3	2.517981778	1.024743446	3	2.121766503	1.054084737	3	2.153484853	Quantified
P61202	COP9 signalosome complex subunit 2	Cops2	FUNCTION: Essential component of the COP	COP9 signalosome comple	11.74	5	5	8	443	51.5640572	5.528808594	0.996118136	8	5.899025513	0.993655155	8	6.070129625	1.002767477	8	4.558621215	0.972020091	8	8.866126643	0.985070385	8	8.955042788	1.027708249	8	3.378562307	Quantified
Q8CHH9	Septin-8	Septin8	FUNCTION: Filament-forming cytoskeletal G1	Septin-8 OS=Mus musculus	7.46	1	4	9	429	49.78116277	5.998535156	1.02597952	1		1.072462303	1		1.111529001	1		0.933127463	1		0.817094163	1		0.965266134	1		Quantified
O35344	Importin subunit alpha-4	Kpna3	FUNCTION: Functions in nuclear protein imp	Importin subunit alpha-4 OS	3.45	1	1	2	521	57.73682619	4.944824219	1.046779969	2	3.757674465	0.914278479	2	5.364093935	0.997018583	2	0.135287478	1.085639988	2	12.78933536	1.024329104	2	1.45363278	0.962869473	2	3.711809979	Quantified
Q9EPL8	Importin-7	Ipo7	FUNCTION: Functions in nuclear protein imp	Importin-7 OS=Mus muscul	7.23	6	6	6	1038	119.409865	4.817871094	0.999592791	5	2.934041029	0.978838451	5	7.242046944	1.008774804	5	2.34452556	0.963702091	5	7.161663287	0.968734635	5	11.70414953	1.021706568	5	4.350598141	Quantified
Q7TMK9	Heterogeneous nuclear ribonucleoprotein Q	Syncrip	FUNCTION: Heterogeneous nuclear ribonucle	Heterogeneous nuclear ribo	10.11	6	6	7	623	69.58961107	8.587402344	1.002570008	7	3.88787014	1.034812856	7	1.221037527	1.040684001	7	3.484244701	0.904539802	7	6.374067453	1.016386423	7	13.5310079	0.93579857	7	5.457869735	Quantified
Q80YX1	Tenascin	Tnc	FUNCTION: Extracellular matrix protein implic	Tenascin OS=Mus musculu	2.18	5	5	10	2110	231.6591224	4.894042969	1.008013234	10	3.513808975	1.036004478	10	3.637169988	1.01143282	10	5.173852936	0.999593996	10	8.288568376	0.918852904	10	6.976608554	0.988963431	10	3.278941098	Quantified
P06800	Receptor-type tyrosine-protein phosphatase C	Ptprc	FUNCTION: Protein tyrosine-protein phospha	Receptor-type tyrosine-prot	5.57	9	9	16	1293	144.745665	5.935058594	0.989321978	16	6.318035059	1.000128602	16	5.765374017	0.982498584	16	5.292585507	1.029348979	16	6.978569627	1.004040841	16	13.72509803	1.027976527	16	8.514403503	Quantified
Q3TBD2	Rho GTPase-activating protein 45	Arhgap45	FUNCTION: Contains a GTPase activator for	Rho GTPase-activating pro	4.3	4	4	4	1116	122.8252234	5.935058594	1.040882859	4	2.529714779	1.066643638	4	4.096969929	1.100055828	4	9.04556449	0.908366436	4	4.694114185	0.878816854	4	5.297429891	0.947545866	4	12.17256098	Quantified

Q8R081	Heterogeneous nuclear ribonucleoprotein L	Hnrnpl	FUNCTION: Splicing factor binding to exonic	Heterogeneous nuclear ribc	6.48	3	3	5	586	63.92325246	8.104003906	1.051785914	5	1.788420205	1.03730079	5	3.56374094	1.004393265	5	0.494569978	0.960153855	5	14.98309115	1.01590355	5	1.91193908	0.903947159	5	6.551757208	Quantified
Q8BJY1	26S proteasome non-ATPase regulatory subunit 5	Psmd5	FUNCTION: Acts as a chaperone during the ε	26S proteasome non-ATPa	10.71	5	5	9	504	55.93690586	5.211425781	1.021309953	9	2.284984398	1.049630777	9	4.684086082	1.003849614	9	5.85776419	0.947251041	9	13.23978498	0.905504505	9	6.345015835	1.058880012	9	7.220854557	Quantified
P01901	H-2 class I histocompatibility antigen, K-B alpha chai	H2-K1	FUNCTION: Involved in the presentation of fo	H-2 class I histocompatibilit	15.18	4	6	10	369	41.27561138	6.392089844	0.966426292	6	2.626733874	1.011860092	6	7.920534634	1.0500388	6	6.883876142	0.975691785	6	2.40103128	0.941426179	6	6.919821208	1.000108543	6	6.855648021	Quantified
P48758	Carbonyl reductase [NADPH] 1	Cbr1	FUNCTION: NADPH-dependent reductase wi	Carbonyl reductase [NADPI	12.64	3	3	6	277	30.62196493	8.309082031	0.979419064	6	3.475218808	0.969425225	6	5.689312494	0.962395029	6	4.678625321	1.041939347	6	19.05415887	1.035745415	6	11.43311371	0.979768645	6	5.785983522	Quantified
P54775	26S proteasome regulatory subunit 6B	Psmc4	FUNCTION: Component of the 26S proteasor	26S proteasome regulatory	8.37	2	3	4	418	47.37858804	5.211425781	0.971443862	3	1.922505225	0.965816444	3	1.253560094	1.069231133	3	11.19736679	0.958233808	3	1.34341767	0.98668084	3	2.914522749	0.980863235	3	1.012002793	Quantified
Q9CWK8	Sorting nexin-2	Snx2	FUNCTION: Involved in several stages of intr	Sorting nexin-2 OS=Mus m	16.57	9	9	10	519	58.43475166	5.122558594	0.981750448	10	2.369304988	1.027776679	10	5.746769934	1.021500896	10	3.34833945	1.011762559	10	7.384653039	0.992958797	10	7.902975982	0.98720292	10	3.914740136	Quantified
P35278	Ras-related protein Rab-5C	Rab5c	FUNCTION: Protein transport. Probably invol	Ras-related protein Rab-5C	26.39	4	4	7	216	23.39777003	8.411621094	0.967324996	7	5.315816805	1.017574644	7	5.698420054	1.045756898	7	8.369522705	0.993388029	7	4.30318984	0.940715764	7	12.76045368	1.038023352	7	7.669538336	Quantified
P35235	Tyrosine-protein phosphatase non-receptor type 11	Ptpn11	FUNCTION: Acts downstream of various rece	Tyrosine-protein phosphata	9.95	6	6	7	593	67.99212699	7.298339844	1.047043199	7	7.055384129	0.994336993	7	2.483558424	0.977440704	7	6.35487342	0.982229015	7	3.234361788	0.997635289	7	2.284186275	0.998096228	7	8.495038791	Quantified
Q08943	FACT complex subunit SSRP1	Ssrp1	FUNCTION: Component of the FACT comple	FACT complex subunit SSF	10.45	6	6	8	708	80.81025968	6.756347656	1.014016264	8	4.819651977	0.988258602	8	6.195061095	1.027822862	8	6.029746787	1.042520725	8	2.026883815	0.919927989	8	12.48956735	0.962879299	8	3.266738513	Quantified
Q923D2	Flavin reductase (NADPH)	Blvrb	FUNCTION: Broad specificity oxidoreductase	Flavin reductase (NADPH)	16.02	3	3	10	206	22.18337513	7.005371094	0.977082459	10	3.06104516	0.997161228	10	9.252377083	0.994287607	10	4.824668315	1.021580713	10	4.943750031	1.093732432	10	8.281111466	0.967246169	10	6.277415999	Quantified
P55258	Ras-related protein Rab-8A	Rab8a	FUNCTION: The small GTPases Rab are key	Ras-related protein Rab-8A	23.19	2	5	11	207	23.65318972	9.070800781	1.029353995	3	0.549936829	1.034069748	3	1.738873687	0.955826528	3	3.346574757	1.031110102	3	4.006998151	0.989801294	3	7.695264075	1.020328902	3	2.11227184	Quantified
Q9CQC6	Basic leucine zipper and W2 domain-containing prot	Bzw1	FUNCTION: Enhances histone H4 gene trans	Basic leucine zipper and W	15.27	6	7	11	419	48.01274847	5.922363281	0.994571891	10	2.723600485	1.059234971	10	3.184818611	0.987850952	10	3.995384892	0.989723739	10	3.413558891	0.977370024	10	10.8181897	1.013205621	10	1.914154203	Quantified
Q9WU60	Attractin	Atrn	FUNCTION: Involved in the initial immune cel	Attractin OS=Mus musculus	1.33	2	2	4	1428	157.9540306	7.269042969	0.890389714	4	2.938103389	0.975952917	4	3.883648762	0.932529845	4	4.772215217	1.137846742	4	15.9722457	1.007530503	4	5.694195904	1.006852056	4	7.041357686	Quantified
Q99MN1	Lysine--tRNA ligase	Kars1	FUNCTION: Catalyzes the specific attachmer	Lysine--tRNA ligase OS=M	11.6	6	6	8	595	67.79649821	5.935058594	0.991970316	8	5.389266719	1.03479551	8	4.799227096	1.04478952	8	6.761319742	0.965328484	8	11.47585667	0.967414071	8	6.656371729	0.952998633	8	3.902608507	Quantified
P09528	Ferritin heavy chain	Fth1	FUNCTION: Stores iron in a soluble, non-toxi	Ferritin heavy chain OS=M	28.02	5	5	8	182	21.05325796	5.884277344	1.035724849	8	6.956431214	0.9280546	8	8.416513084	1.025431469	8	11.55415773	1.035412288	8	5.898178297	1.056129025	8	10.474286	0.927809939	8	7.65648935	Quantified
Q3UV17	Keratin, type II cytoskeletal 2 oral	Krt76	FUNCTION: Probably contributes to terminal	Keratin, type II cytoskeletal	2.69	1	2	5	594	62.80596402	8.426269531	1.202379518	4	1.094762865	1.238555698	4	4.027699405	0.718386312	4	4.599342301	1.112566491	4	2.885664694	0.804109393	4	6.585686361	0.874486428	4	4.144326766	Quantified
P28474	Alcohol dehydrogenase class-3	Adh5	FUNCTION: Catalyzes the oxidation of long-c	Alcohol dehydrogenase cla	14.44	6	6	10	374	39.52206812	7.254394531	0.980296991	10	5.972790507	1.022584467	10	4.889098732	1.041618051	10	5.751194108	1.004072943	10	6.355075552	0.9383262	10	10.43879255	1.014844557	10	7.558645626	Quantified
O54941	SWI/SNF-related matrix-associated actin-dependent	Smarce1	FUNCTION: Involved in transcriptional activat	SWI/SNF-related matrix-ass	6.81	2	2	4	411	46.60955582	4.881347656	1.042787223	4	5.280887224	0.903852978	4	14.40507456	0.98610442	4	5.745015083	1.032785926	4	5.295344391	1.121457726	4	4.257101796	1.018310117	4	6.52830397	Quantified
Q810U3	Neurofascin	Nfasc	FUNCTION: Cell adhesion, ankyrin-binding p	Neurofascin OS=Mus musc	5.73	7	7	12	1240	137.8892348	6.188964844	1.027668032	12	7.912296954	0.993376468	12	4.056719692	1.040912843	12	4.601346556	1.004111768	12	2.557820089	1.028788465	12	6.629697224	0.974257599	12	3.700690408	Quantified
O35350	Calpain-1 catalytic subunit	Capn1	FUNCTION: Calcium-regulated non-lysosoma	Calpain-1 catalytic subunit	7.29	4	5	8	713	82.05429699	5.871582031	1.024426395	7	3.823315271	1.029026954	7	2.070626616	1.027997052	7	8.677723572	0.976080469	7	1.888655974	0.919048328	7	2.793574937	0.995361869	7	8.672149176	Quantified
Q9Z2N8	Actin-like protein 6A	Actl6a	FUNCTION: Involved in transcriptional activat	Actin-like protein 6A OS=M	8.86	4	4	7	429	47.41734915	5.604980469	0.979379994	7	4.064278192	0.996583243	7	4.509967206	1.030984001	7	7.284145168	1.020416206	7	5.476987281	0.947197881	7	14.12818066	0.997155801	7	3.240014027	Quantified
P18242	Cathepsin D	Ctsd	FUNCTION: Acid protease active in intracellu	Cathepsin D OS=Mus musc	15.12	6	6	7	410	44.92499017	7.151855469	1.033184758	7	2.311118937	1.040243174	7	3.222166632	0.96280591	7	2.379650169	1.038478509	7	0.78468797	0.953996642	7	6.704776271	0.95691309	7	4.205341603	Quantified
Q8C650	Septin-10	Septin10	FUNCTION: Filament-forming cytoskeletal G1	Septin-10 OS=Mus muscul	5.97	1	3	8	452	52.38816167	6.595214844	1.002931624	1		0.968934867	1		1.048076889	1		0.999501649	1		0.984624205	1		1.007410354	1		Quantified
Q99KJ8	Dynactin subunit 2	Dctn2	FUNCTION: Modulates cytoplasmic dynein bi	Dynactin subunit 2 OS=Mu	16.42	7	7	8	402	44.0897471	5.262207031	0.987362289	8	6.196173444	0.99650629	8	7.175120778	0.971355832	8	2.434120688	1.018405022	8	1.669841438	1.021429195	8	8.567376419	0.990190277	8	4.642223244	Quantified
O55029	Coatomer subunit beta'	Copb2	FUNCTION: The coatomer is a cytosolic prot	Coatomer subunit beta' OS	5.08	4	4	5	905	102.3844442	5.300292969	0.931371734	5	10.94178282	1.049838064	5	28.92817486	0.960078669	5	9.394034771	0.932749082	5	12.20779298	0.866769199	5	5.539029354	0.990515702	5	13.60858286	Quantified
Q8R550	SH3 domain-containing kinase-binding protein 1	Sh3kbp1	FUNCTION: Adapter protein involved in regul	SH3 domain-containing kin	4.23	2	2	2	709	78.12225165	7.547363281	0.924842099	2	13.76771597	0.941784765	2	2.170788375	0.971271146	2	10.89749345	1.113642049	2	0.780642889	0.923784014	2	11.52659437	1.109066397	2	18.36844502	Quantified
Q921M3	Splicing factor 3B subunit 3	Sf3b3	FUNCTION: Involved in pre-mRNA splicing a	Splicing factor 3B subunit 3	9.29	8	8	11	1217	135.464736	5.262207031	1.010259735	11	3.209853894	1.00134616	11	2.315544686	0.948317091	11	7.797332141	1.033372025	11	4.412845238	0.981643017	11	2.125338725	1.031425932	11	6.994094124	Quantified
Q6P1F6	Serine/threonine-protein phosphatase 2A 55 kDa reg	Ppp2r2a	FUNCTION: The B regulatory subunit might n	Serine/threonine-protein ph	9.84	5	5	8	447	51.65948894	6.201660156	1.00376812	8	3.507485991	1.02488736	8	5.034706276	1.013072725	8	6.461867392	0.942101208	8	6.861668339	0.942705745	8	3.389547017	0.986863201	8	7.505081266	Quantified
P28843	Dipeptidyl peptidase 4	Dpp4	FUNCTION: Cell surface glycoprotein recept	Dipeptidyl peptidase 4 OS=	3.55	3	3	5	760	87.38118917	6.417480469	0.987511492	5	1.407196163	0.968722382	5	3.002095161	1.129855713	5	6.231442887	0.972756679	5	2.264751581	1.019178172	5	0.437009586	0.972117349	5	5.529816769	Quantified
Q63844	Mitogen-activated protein kinase 3	Mapk3	FUNCTION: Serine/threonine kinase which a	Mitogen-activated protein k	13.16	2	4	5	380	43.03911698	6.609863281	1.015283748	2	0.078530049	1.000673203	2	0.398115964	0.996338675	2	1.231327036	0.987229152	2	1.988195794	0.978152933	2	3.573630109	1.034252755	2	3.125606224	Quantified
P09535	Insulin-like growth factor II	Igf2	FUNCTION: The insulin-like growth factors pc	Insulin-like growth factor II	13.89	2	2	3	180	20.01718589	9.158691406	0.857000084	3	1.975531193	1.015508831	3	5.697228353	1.210095193	3	9.617226157	1.019505603	3	0.340680551	0.974340524	3	1.920859663	0.981513652	3	1.527452728	Quantified
P84091	AP-2 complex subunit mu	Ap2m1	FUNCTION: Component of the adaptor prot	AP-2 complex subunit mu (	7.59	3	3	6	435	49.62302771	9.539550781	1.045656811	6	2.474378943	1.074847806	6	6.121513719	1.018983631	6	6.645490337	0.975118775	6	3.61353033	0.934846496	6	9.855131611	0.951850369	6	2.164229762	Quantified
P08752	Guanine nucleotide-binding protein G(i) subunit alpha	Gnai2	FUNCTION: Guanine nucleotide-binding prot	Guanine nucleotide-binding	26.2	6	7	7	355	40.46303879	5.452636719	1.00341067	6	4.865607821	0.996600163	6	5.251212116	1.0225545	6	5.939095611	0.973633209	6	2.42312612	0.947911183	6	3.584594689	1.030803187	6	4.894014191	Quantified
P61028	Ras-related protein Rab-8B	Rab8b	FUNCTION: The small GTPases Rab are key	Ras-related protein Rab-8B	17.39	1	4	9	207	23.5880713	9.070800781	0.9373643	1		1.036778866	1		0.970970643	1		1.045570613	1		1.112792902	1		0.964578733	1		Quantified
Q9DAK9	14 kDa phosphohistidine phosphatase	Phpt1	FUNCTION: Exhibits phosphohistidine phosp	14 kDa phosphohistidine pl	49.19	5	5	9	124	13.98771025	5.528808594	0.996078698	9	2.718500571	1	9	5.791231742	1.025658411	9	7.884127169	0.970184762	9	10.03855283	0.98842139	9	12.50399726	0.979599442	9	5.471548012	Quantified
Q9QUR6	Prolyl endopeptidase	Prep	FUNCTION: Cleaves peptide bonds on the C-	Prolyl endopeptidase OS=M	10.56	6	6	14	710	80.69989667	5.731933594	1.003442929	14	7.313124738	1.011330539	14	5.076347652	1.022785909	14	11.4322781	1.021635414	14	11.03917801	0.969669075	14	3.562603685	0.972498752	14	3.957645712	Quantified
Q8JZK9	Hydroxymethylglutaryl-CoA synthase, cytoplasmic	Hmgcs1	FUNCTION: Catalyzes the condensation of a	Hydroxymethylglutaryl-CoA	6.35	3	3	4	520	57.53246104	5.985839844	0.981186953	4	0.922819989	0.99147974	4	5.889099895	0.968876195	4	1.122066749	1.128264567	4	3.45462492	0.993420468	4	9.794047144	0.981490588	4	1.071282321	Quantified
P51150	Ras-related protein Rab-7a	Rab7a	FUNCTION: Key regulator in endo-lysosomal	Ras-related protein Rab-7a	23.19	4	4	5	207	23.47484232	6.697753906	0.986373442	5	3.609482745	0.998140758	5	4.967096527	0.960678647	5	4.43881015	1.018713686	5	6.328196907	1.011498436	5	4.60205518	1.002269558	5	3.412899261	Quantified
P97384	Annexin A11	Anxa11	FUNCTION: Required for midbody formation ;	Annexin A11 OS=Mus mus	4.17	1	2	4	503	54.04481798	7.664550781	0.963885294	2	0.203089066	0.979335645	2	4.79162555	1.076486816	2	0.887829364	0.941867315	2	1.431803684	1.067149847	2	11.0058237	1.011190281	2	0.39234832	Quantified
Q9EQF5	Dihydropyrimidinase	Dpys	FUNCTION: Catalyzes the second step of the	Dihydropyrimidinase OS=M	5.97	3	3	5	519	56.68861582	7.195800781	0.996902781	5	1.040169506	1.065711835	5	9.874327931	1.00393745	5	3.523990038	0.931478027	5	7.188796768	1.018173494	5	5.291567974	0.953702183	5	2.97033502	Quantified
O35841	Apoptosis inhibitor 5	Api5	FUNCTION: Antiapoptotic factor that may hav	Apoptosis inhibitor 5 OS=M	11.9	6	6	8	504	56.74977995	5.922363281	0.957972174	8	8.548076267	1.005912212	8	5.18497448	1.022669234	8	4.683167112	1.038572486	8	12.610494	1.064150953	8	14.24153851	0.98378024	8	7.867864161	Quantified
Q99MR6	Serrate RNA effector molecule homolog	Srrt	FUNCTION: Acts as a mediator between the	Serrate RNA effector molec	6.17	5	5	11	875	100.3903006	5.973144531	1.008823888	11	4.809276525	1.046205588	11	9.598074363	1.061892805	11	4.126528306	0.947255856	11	4.861169097	1.006371268	11	9.831247099	0.991921675	11	6.100648598	Quantified
E9Q7G0	Nuclear mitotic apparatus protein 1	Numa1	FUNCTION: Microtubule (MT)-binding protein	Nuclear mitotic apparatus p	8.36	15	15	17	2094	235.48742	5.871582031	0.951540301	17	3.840577004	0.931243114	17	6.385818505	0.960165214	17	4.994866781	1.09538543	17	5.787956652	1.074817196	17	7.596507478	1.040658266	17	4.048949844	Quantified
Q5SUR0	Phosphoribosylformylglycinamidine synthase	Pfas	FUNCTION: Phosphoribosylformylglycinamid	Phosphoribosylformylglycin	5.01	5	5	7	1337	144.5378363	5.668457031	1.078066081	7	6.432187587	1.00633225	7	3.83323122	1.030205222	7	4.032458752	0.97479316	7	3.972076457	1.029456905	7	12.0658155	0.98798784	7	6.375560848	Quantified
P19973	Lymphocyte-specific protein 1	Lsp1	FUNCTION: May play a role in mediating neu	Lymphocyte-specific protei	15.76	4	4	9	330	36.69183259	4.817871094	1.013915875	9	7.103929802	1.005077733	9	4.456107883	0.990142701	9	6.908492505	1.021207018	9	10.28008444	0.954578313	9	13.61668623	1.00364411	9	3.4251945	Quantified
P23198	Chromobox protein homolog 3	Cbx3	FUNCTION: Component of heterochromatin.	Chromobox protein homolo	17.49	4	4	8	183	20.84234021	5.224121094	0.954650166	8	7.182724645	0.993917667	8	3.635541843	1.051343003	8	6.722879848	0.978834231	8	10.31207387	1.094326426	8	6.268338435	0.956082149	8	4.277085864	Quantified
P45700	Mannosyl-oligosaccharide 1,2-alpha-mannosidase IA	Man1a1	FUNCTION: Involved in the maturation of Asr	Mannosyl-oligosaccharide	7.48	5	5	8	655	73.22956274	6.814941406	1.020582613	8	4.638731463	1.020092138	8	5.619639994	1.006434381	8	5.163994999	1.013764583	8	5.128030394	0.899869827	8	10.88269321	1.01540159	8	6.269040096	Quantified
P10107	Annexin A1	Anxa1	FUNCTION: Plays important roles in the innat	Annexin A1 OS=Mus musc	10.69	3	3	4	346	38.70997548	7.371582031	0.932572076	4	4.748296556	1.000886929	4	7.6555478	0.946629831	4	3.595351009	0.977086844	4	7.67089103	1.049155022	4	5.802935626	1.096920836	4	2.93543116	Quantified
Q3UIA2	Rho GTPase-activating protein 17	Arhgap17	FUNCTION: Rho GTPase-activating protein ir	Rho GTPase-activating pro	2.6	2	2	4	846	92.14476762	7.562011719	0.998551867	4	1.19197843	1.074156508	4	3.511040026	0.994366346	4	10.59483116	0.986971654	4	14.29663233	1.072627855	4	15.85072566	0.996254992	4	7.774928942	Quantified
P12023	Amyloid-beta A4 protein	App	FUNCTION: Functions as a cell surface recep	Amyloid-beta A4 protein OS	4.29	3	3	4	770	86.66703554	4.792480469	0.987178806	4	3.759588758	0.931719757	4	2.634944187	1.045395563	4	3.600459928	1.077181436	4	2.807280358	0.979295379	4	0.611213888	0.991797859	4	2.175191667	Quantified
P70428	Exostosin-2	Ext2	FUNCTION: Glycosyltransferase required for	Exostosin-2 OS=Mus musc	8.64	5	5	10	718	82.01186838	6.653808594	1.018715574	10	4.772842629	1.04523538	10	5.229800102	0.974759101	10	9.509614628	0.991947556	10	8.055030639	0.974531245	10	6.506452265	0.974889137	10	2.258161226	Quantified
Q80SW1	S-adenosylhomocysteine hydrolase-like protein 1	Ahcyl1	FUNCTION: Multifaceted cellular regulator w	S-adenosylhomocysteine h	3.96	1	3	10	530	58.91344487	6.888183594	0.966519116	1		1.047767003	1		1.023537606	1		0.984579982	1		1.032995707	1		0.972431859	1		Quantified
O35643	AP-1 complex subunit beta-1	Ap1b1	FUNCTION: Subunit of clathrin-associated ac	AP-1 complex subunit beta	5.73	1	7	12	943	103.8688926	5.173339844	1.021472394	1		0.98805324	1		0.971477849	1		1.059836393	1		1.022164566	1		0.969957134	1		Quantified
Q9DBG3	AP-2 complex subunit beta	Ap2b1	FUNCTION: Component of the adaptor protei	AP-2 complex subunit beta	5.98	1	7	12	937	104.5160572	5.376464844	0.976287426	1		1.064432389	1		1.02977579	1		0.905911425	1		1.037622069	1		1.01429143	1		Quantified
Q8K4Z5	Splicing factor 3A subunit 1	Sf3a1	FUNCTION: Involved in pre-mRNA splicing a	Splicing factor 3A subunit 1	9.86	7	7	9	791	88.48935634	5.224121094	0.995040375	9	6.094981542	0.961746577	9	5.439747279	0.944505962	9	5.875213714	1.016050386	9	14.11811419	1.065750918	9	7.299795244	0.993959698	9	6.343683523	Quantified
P70695	Fructose-1,6-bisphosphatase isozyme 2	Fbp2	FUNCTION: Catalyzes the hydrolysis of fructo	Fructose-1,6-bisphosphatas	3.24	1	1	7	339	36.92386948	6.163574219	0.957043839	7	3.092450345	1.006080574	7	3.230491854	0.953762143	7	6.099077554	1.02891589	7	2.225643568	0.967562406	7	10.48561091	1.010070623	7	4.040114838	Quantified
Q9JK38	Glucosamine 6-phosphate N-acetyltransferase	Gnpnat1		Glucosamine 6-phosphate I	7.07	2	2	5	184	20.77766535	7.693847656	0.981783989	5	3.265281142	0.963253123	5	3.928637221	0.964910607	5	6.987959598	1.043420243	5	2.610216467	1.014696021	5	3.675595396	1.056240786	5	1.440455239	Quantified
P63038	60 kDa heat shock protein, mitochondrial	Hspd1	FUNCTION: Chaperonin implicated in mitoch	60 kDa heat shock protein,	10.65	5	5	6	573	60.91739324	6.176269531	1.010806092	6	2.429015089	0.995263502	6	2.79170485	0.936986164	6	4.718301005	1.073775722	6	7.093106296	1.045463599	6	10.18625436	1.008463635	6	3.870001064	Quantified
Q03265	ATP synthase subunit alpha, mitochondrial	Atp5f1a	FUNCTION: Mitochondrial membrane ATP sy	ATP synthase subunit alph	7.96	4	4	7	553	59.71559681	9.187988281	1.028327283	7	7.275901839	0.918114474	7	16.64701627	0.935150453	7	11.0393396	1.024568665	7	7.024624194	1.103659154	7	16.00199078	1.074432887	7	4.000125364	Quantified
P97447	Four and a half LIM domains protein 1	Fhl1	FUNCTION: May have an involvement in mus	Four and a half LIM domain	8.21	2	2	2	280	31.86682517	8.367675781	1.05917602	2	6.957119318	1.075931211	2	0.353437464	1.100443948	2	14.80345605	0.908246654	2	8.423028115	0.890312824	2	13.76496885	0.906349668	2	12.64885574	Quantified
Q9R062	Glycogenin-1	Gyg1	FUNCTION: Self-glucosylates, via an inter-su	Glycogenin-1 OS=Mus mus	11.71	3	3	4	333	37.37830536	5.287597656	1.048099172	4	2.383394234	1.009897469	4	4.647886238	1.030282333	4	1.860466684	0.978365922	4	2.51571389	0.962682657	4	7.077108784	0.960337759	4	2.457270803	Quantified
O70194	Eukaryotic translation initiation factor 3 subunit D	Eif3d	FUNCTION: mRNA cap-binding component o	Eukaryotic translation initiat	4.38	2	2	3	548	63.94840429	6.049316406	1.063967923	3	2.040773294	1.007681229	3	2.109890648	1.058392434	3	5.603691012	1.046062398	3	5.529183081	0.99208268	3	11.01566015	0.948104328	3	5.48231537	Quantified
Q62000	Mimecan	Ogn	FUNCTION: Induces bone formation in conju	Mimecan OS=Mus musculu	6.38	1	1	2	298	33.99063477	5.744628906	0.919359486	2	8.37721931	1.038524863	2	5.989448784	0.817104661	2	15.94135222	1.056021037	2	2.496105473	1.167145102	2	7.231207953	1.100710599	2	12.34112954	Quantified
Q61554	Fibrillin-1	Fbn1	FUNCTION: [Fibrillin-1]: Structural componen	Fibrillin-1 OS=Mus musculu	4.46	13	13	19	2873	312.0833685	4.919433594	0.93340842	19	5.840346997	0.997510299	19	3.87970888	1.088456904	19	5.071833168	0.988828799	19	3.866406348	1.023685047	19	6.109344484	1.007456197	19	7.243392237	Quantified
Q02819	Nucleobindin-1	Nucb1	FUNCTION: Major calcium-binding protein of	Nucleobindin-1 OS=Mus m	15.25	6	6	8	459	53.37586319	5.071777344	1.006589553	8	2.743763336	0.9934297	8	7.122951521	1.010936278	8	7.675750353	0.988676835	8	3.040162982	1.046495533	8	9.000490684	0.950960542	8	8.549269146	Quantified
Q9CQI6	Coactosin-like protein	Cotl1	FUNCTION: Binds to F-actin in a calcium-ind	Coactosin-like protein OS=I	30.99	6	6	9	142	15.9339898	5.401855469	1.014122878	9	4.908707289	1.035600009	9	5.425350401	1.022417277	9	5.242922936	0.975836035	9	7.025371305	0.932462987	9	9.586704153	0.971123644	9	3.238416477	Quantified
Q91YM2	Rho GTPase-activating protein 35	Arhgap35	FUNCTION: Rho GTPase-activating protein (r	Rho GTPase-activating pro	1.53	2	2	3	1499	170.2853904	6.609863281	1.0785334	3	4.003691718	0.975951139	3	0.771951835	0.866339242	3	0.440065181	1.131331414	3	0.484714197	0.860454676	3	2.950138543	1.097638884	3	5.98216463	Quantified
Q61656	Probable ATP-dependent RNA helicase DDX5	Ddx5	FUNCTION: Involved in the alternative regula	Probable ATP-dependent R	12.54	5	8	10	614	69.24681118	8.924316406	1.01971309	6	2.050256187	1.062687228	6	4.092759526	1.027363601	6	6.196790449	0.970040567	6	4.602809782	0.946604338	6	3.973926893	0.979010123	6	3.815250323	Quantified
Q61301	Catenin alpha-2	Ctnna2	FUNCTION: May function as a linker betweer	Catenin alpha-2 OS=Mus m	4.41	1	5	7	953	105.2198251	5.706542969	1.0416255	1		1.070658869	1		0.9413584	1		0.989605451	1		0.816151109	1		1.022274199	1		Quantified
Q812C9	Retina-specific copper amine oxidase	Aoc2	FUNCTION: Has a monoamine oxidase activi	Retina-specific copper amir	2.38	2	2	5	757	83.53083368	6.785644531	1.020299464	5	1.832088733	0.982492725	5	1.834252389	1.023117299	5	1.713024632	1.004146036	5	1.944665307	0.966033157	5	8.228929384	1.045613194	5	12.02251699	Quantified
E9PV24	Fibrinogen alpha chain	Fga	FUNCTION: Cleaved by the protease thrombi	Fibrinogen alpha chain OS=	3.17	4	4	12	789	87.37532693	6.112792969	0.993162518	12	6.352591322	0.904372592	12	17.90369336	1.01821065	12	3.133638798	1.106859984	12	11.98736034	1.121452023	12	18.25656015	0.922174619	12	9.913950196	Quantified
Q61702	Inter-alpha-trypsin inhibitor heavy chain H1	Itih1	FUNCTION: May act as a carrier of hyaluronε	Inter-alpha-trypsin inhibitor	1.54	2	2	11	907	101.0040323	6.961425781	0.988133495	11	2.21939715	1.086565527	11	3.248560968	1.010640951	11	5.236787416	0.893144673	11	7.772851619	1.02439552	11	5.292635791	1.005371319	11	4.932526986	Quantified
Q9WTM5	RuvB-like 2	Ruvbl2	FUNCTION: Possesses single-stranded DNA	RuvB-like 2 OS=Mus musc	13.17	6	6	10	463	51.08053062	5.643066406	1.003030727	10	2.822550167	0.963519437	10	3.353642647	0.936051705	10	4.469315076	1.074450852	10	2.565685573	1.046254206	10	4.537446866	1.036277765	10	3.879304171	Quantified
Q62469	Integrin alpha-2	Itga2	FUNCTION: Integrin alpha-2/beta-1 is a colla	Integrin alpha-2 OS=Mus m	1.27	1	1	3	1178	128.8737444	5.211425781	0.952885636	3	0.401373593	0.949107678	3	2.412395852	0.939425134	3	0.118255362	1.084411796	3	1.779581735	1.055804747	3	7.555129619	1.01892114	3	2.528973985	Quantified
Q7TSV4	Phosphoglucomutase-2	Pgm2	FUNCTION: Catalyzes the conversion of the	Phosphoglucomutase-2 OS	1.61	1	1	5	620	68.70428036	6.138183594	0.993249195	5	1.669471837	0.955791864	5	0.852156517	0.958634506	5	9.033594915	1.071142534	5	4.761121225	0.984791445	5	2.194575873	1.02191261	5	5.952095776	Quantified
P61027	Ras-related protein Rab-10	Rab10	FUNCTION: The small GTPases Rab are key	Ras-related protein Rab-10	25	4	6	11	200	22.52659012	8.382324219	0.998409062	5	1.966817147	0.988147925	5	6.675194011	0.989809694	5	5.121707908	1.060426369	5	10.25483375	1.026878916	5	4.881918745	1.009881801	5	1.954179165	Quantified
P32233	Developmentally-regulated GTP-binding protein 1	Drg1	FUNCTION: Catalyzes the conversion of GTF	Developmentally-regulated	4.36	1	1	2	367	40.48681477	8.895019531	1.027785031	2	4.635752132	0.925483133	2	3.172926653	1.057490424	2	2.110443551	0.964053257	2	2.088905371	1.001148983	2	14.60733427	1.040577216	2	4.943978384	Quantified
Q7M6Y3	Phosphatidylinositol-binding clathrin assembly protei	Picalm	FUNCTION: Cytoplasmic adapter protein that	Phosphatidylinositol-binding	4.55	2	2	2	660	71.49829152	7.913574219	1.015010426	2	5.837548756	1.042171722	2	9.932199101	1.015147183	2	1.604217843	0.953547343	2	0.739072879	0.944634463	2	6.539386558	1.018323028	2	1.031306411	Quantified
P63001	Ras-related C3 botulinum toxin substrate 1	Rac1	FUNCTION: Plasma membrane-associated s	Ras-related C3 botulinum tc	33.85	3	7	13	192	21.43624265	8.499511719	1.007802628	5	3.232492379	1.040875299	5	5.072161312	0.957200576	5	4.14572718	1.038575358	5	15.4083931	0.997944336	5	11.24943062	0.97840369	5	14.21038292	Quantified
Q8BVE3	V-type proton ATPase subunit H	Atp6v1h	FUNCTION: Subunit of the peripheral V1 corr	V-type proton ATPase subu	4.76	1	1	1	483	55.81914446	6.609863281	1.046598087	1		0.961382222	1		0.909409368	1		1.036433095	1		1.032958019	1		1.061270474	1		Quantified
P17563	Methanethiol oxidase	Selenbp1	FUNCTION: Catalyzes the oxidation of metha	Methanethiol oxidase OS=	16.1	6	6	9	472	52.48036472	6.290527344	1.015366077	9	2.798656797	1.007094837	9	3.866512407	1.000568309	9	5.95377574	0.961887065	9	5.801881898	0.979515471	9	9.418465573	0.971620442	9	6.728909565	Quantified
Q3TWW8	Serine/arginine-rich splicing factor 6	Srsf6	FUNCTION: Plays a role in constitutive splici	Serine/arginine-rich splicing	10.32	2	4	5	339	39.00210141	11.45849609	1.089370554	2	3.223535463	1.151990898	2	3.032931769	1.072950991	2	1.888253943	0.870346946	2	2.05130443	0.851991667	2	11.27882459	0.896083685	2	1.423954084	Quantified
P59999	Actin-related protein 2/3 complex subunit 4	Arpc4	FUNCTION: Actin-binding component of the A	Actin-related protein 2/3 co	20.83	4	4	13	168	19.6543047	8.426269531	1.02384342	13	3.574774765	1.021650453	13	4.859226699	1.024313439	13	2.556592514	0.987323531	13	4.047436572	0.966345813	13	5.114152823	0.989239875	13	1.845969919	Quantified
P25785	Metalloproteinase inhibitor 2	Timp2	FUNCTION: Complexes with metalloproteina	Metalloproteinase inhibitor	13.64	4	4	6	220	24.31209057	7.488769531	0.956951996	6	3.911624606	1.082711792	6	4.536418206	0.991849347	6	3.883354913	0.904310017	6	4.696638214	1.085175336	6	7.840211361	1.00143479	6	7.064956066	Quantified
Q9WVA3	Mitotic checkpoint protein BUB3	Bub3	FUNCTION: Has a dual function in spindle-as	Mitotic checkpoint protein E	14.42	3	3	3	326	36.93111917	6.844238281	1.019069945	3	3.992539703	0.998135847	3	2.108195736	0.980444576	3	6.267002148	1.006146866	3	8.683016009	0.991702286	3	7.302248393	0.995247948	3	9.110987743	Quantified
P26443	Glutamate dehydrogenase 1, mitochondrial	Glud1	FUNCTION: Mitochondrial glutamate dehydrc	Glutamate dehydrogenase	8.24	4	4	6	558	61.29817574	8.001464844	1.011755473	6	3.890795817	1.021466606	6	6.811887488	1.018248712	6	2.985665538	0.975929223	6	9.809769815	0.996356905	6	2.692739718	0.972503661	6	3.860476631	Quantified
O35522	Proteasome subunit beta type-9	Psmb9	FUNCTION: The proteasome is a multicatalyt	Proteasome subunit beta ty	8.68	2	2	6	219	23.41173353	5.224121094	1.031003474	6	1.64036135	0.958017841	6	2.992411335	1.030244911	6	2.392150284	1.040877643	6	5.436828657	1.014256906	6	6.540960605	0.961268552	6	4.946934581	Quantified
P19324	Serpin H1	Serpinh1	FUNCTION: Binds specifically to collagen. Co	Serpin H1 OS=Mus muscul	13.67	5	5	5	417	46.50418561	8.821777344	0.951090467	5	4.800998305	0.976842952	5	7.788650506	0.969152279	5	10.50116092	0.986063734	5	11.73477537	1.010295101	5	4.464394334	1.046754156	5	15.31319363	Quantified
P99026	Proteasome subunit beta type-4	Psmb4	FUNCTION: Component of the 20S core prot	Proteasome subunit beta ty	17.05	3	3	5	264	29.09733627	5.643066406	1.041049194	5	3.165568962	1.055415606	5	5.960796938	0.966858254	5	2.275654454	0.983124234	5	1.565708984	0.844250881	5	11.07854574	1.059035558	5	3.650445755	Quantified
Q61550	Double-strand-break repair protein rad21 homolog	Rad21	FUNCTION: [Double-strand-break repair prot	Double-strand-break repair	2.05	1	1	4	635	72.03791872	4.640136719	1.059728107	4	1.025678691	0.894186979	4	3.112625827	1.044841971	4	9.65324847	1.014118075	4	6.884282441	1.088609873	4	21.30519026	0.968936119	4	1.306830469	Quantified
Q80XI4	Phosphatidylinositol 5-phosphate 4-kinase type-2 bet	Pip4k2b	FUNCTION: Participates in the biosynthesis c	Phosphatidylinositol 5-phos	7.21	1	3	3	416	47.28876832	7.327636719	1.021800129	1		1.153016012	1		1.049172785	1		0.867042148	1		0.937049369	1		0.947016924	1		Quantified
Q9JJN5	Carboxypeptidase N catalytic chain	Cpn1	FUNCTION: Protects the body from potent va	Carboxypeptidase N cataly	2.84	1	1	3	457	51.81300936	8.279785156	0.995879942	3	6.488167152	1.073925488	3	2.233694466	0.951558453	3	7.308179545	0.987135152	3	1.030125105	1.045598227	3	2.746198751	0.966645894	3	13.82296345	Quantified
P02463	Collagen alpha-1(IV) chain	Col4a1	FUNCTION: Type IV collagen is the major str	Collagen alpha-1(IV) chain	1.44	1	2	6	1669	160.5785242	8.235839844	1.000700638	3	2.154397787	1.058038099	3	3.490945288	1.024294107	3	14.12990555	0.90509855	3	4.086388639	1.140139497	3	13.00044469	0.983358633	3	12.19823551	Quantified
P62908	40S ribosomal protein S3	Rps3	FUNCTION: Involved in translation as a comp	40S ribosomal protein S3 C	17.7	4	4	7	243	26.657416	9.656738281	0.985013397	7	2.594140985	0.995016861	7	4.347828991	0.974772194	7	7.470757271	1.016921536	7	3.882867896	1.044787127	7	8.893448284	1.012606026	7	4.209521436	Quantified
Q920B9	FACT complex subunit SPT16	Supt16h	FUNCTION: Component of the FACT comple	FACT complex subunit SPT	3.82	3	3	7	1047	119.7492967	5.655761719	1.054114808	7	6.061706013	0.978328356	7	4.68058847	0.990344861	7	9.010352855	1.037491136	7	4.18142546	0.923780846	7	8.00190639	1.006756872	7	4.979206333	Quantified
P12367	cAMP-dependent protein kinase type II-alpha regulat	Prkar2a	FUNCTION: Regulatory subunit of the cAMP-	cAMP-dependent protein ki	4.74	2	2	4	401	45.36054646	4.894042969	1.031374684	4	1.557145058	1.042287036	4	1.519603693	0.967519231	4	4.010222608	1.044470342	4	0.988519945	0.952310863	4	6.929467333	0.967842623	4	2.281874054	Quantified
Q8BV49	Pyrin and HIN domain-containing protein 1	Pyhin1		Pyrin and HIN domain-cont	5.71	2	2	3	420	46.85731027	9.451660156	1.065594822	3	9.056949154	1.043387846	3	1.478249354	1.025164415	3	7.546498111	0.982589477	3	0.302383082	0.924669464	3	2.241651307	0.93639162	3	4.964693776	Quantified
O88456	Calpain small subunit 1	Capns1	FUNCTION: Regulatory subunit of the calciur	Calpain small subunit 1 OS	4.83	1	1	4	269	28.44494788	5.630371094	0.948673864	4	6.476746549	0.990610516	4	0.882576424	0.951636853	4	6.532728569	0.995518759	4	1.885378317	1.161025182	4	14.82811145	1.004298727	4	3.307856747	Quantified
P18654	Ribosomal protein S6 kinase alpha-3	Rps6ka3	FUNCTION: Serine/threonine-protein kinase t	Ribosomal protein S6 kinas	12.7	7	7	9	740	83.64090271	6.888183594	1.028829103	9	2.295310021	0.971820123	9	4.447963942	0.978616262	9	5.060899945	0.986581392	9	2.822143741	1.010195262	9	5.957704225	0.980994514	9	5.129434286	Quantified
Q9R1T4	Septin-6	Septin6	FUNCTION: Filament-forming cytoskeletal G1	Septin-6 OS=Mus musculu	16.36	2	6	8	434	49.58817133	6.430175781	1.000721264	2	0.447303155	1.028909544	2	3.485695053	1.064376882	2	0.010927063	0.958627931	2	1.785669939	0.974369916	2	6.475395759	0.971846469	2	1.156231626	Quantified
Q61207	Prosaposin	Psap	FUNCTION: [Prosaposin]: Behaves as a mye	Prosaposin OS=Mus musc	9.34	5	5	6	557	61.3813568	5.186035156	0.991080437	6	5.16552458	0.988508967	6	3.886455922	1.093683886	6	6.231100217	0.977471468	6	4.481057711	0.997820157	6	5.676224808	0.945276006	6	5.189840821	Quantified
Q8CF98	Collectin-10	Colec10	FUNCTION: Lectin that binds to various suga	Collectin-10 OS=Mus musc	13.36	4	4	5	277	30.50432144	7.327636719	0.997581702	5	1.610215186	1.010234469	5	1.661253371	0.887816909	5	3.132405547	1.056290354	5	6.436557894	1.029733475	5	5.479225312	0.996044389	5	10.3281023	Quantified
Q9R1P3	Proteasome subunit beta type-2	Psmb2	FUNCTION: Component of the 20S core prot	Proteasome subunit beta ty	17.91	3	3	4	201	22.89173294	7.020019531	0.940898912	3	3.203053734	0.990475618	3	0.402826215	1.036788332	3	4.695907286	1.076230433	3	0.275867732	1.094705222	3	17.18767961	0.951400077	3	6.185229608	Quantified
P35396	Peroxisome proliferator-activated receptor delta	Ppard	FUNCTION: Ligand-activated transcription fa	Peroxisome proliferator-acti	1.59	1	1	6	440	49.68293557	7.020019531	0.954415655	6	3.979319901	1.011775015	6	2.029412151	0.927881297	6	1.872518554	1.026406668	6	3.185734534	1.100059615	6	4.385216249	1.048438928	6	4.339093629	Quantified
O35639	Annexin A3	Anxa3	FUNCTION: Inhibitor of phospholipase A2, al	Annexin A3 OS=Mus musc	7.43	1	2	3	323	36.36156923	5.757324219	1.023824139	1		0.960645859	1		0.938220747	1		1.063753863	1		1.021727004	1		1.031900514	1		Quantified
Q7TNC4	Putative RNA-binding protein Luc7-like 2	Luc7l2	FUNCTION: May bind to RNA via its Arg/Ser-	Putative RNA-binding prote	7.65	2	2	2	392	46.55468044	10.09619141	0.996488367	2	2.667580249	1.035718441	2	1.721707265	1.040435066	2	12.23084929	0.995015882	2	6.106844718	1.045795481	2	3.834238591	0.911524681	2	1.20024647	Quantified
Q8VE97	Serine/arginine-rich splicing factor 4	Srsf4	FUNCTION: Plays a role in alternative splice	Serine/arginine-rich splicing	7.77	2	4	5	489	55.94676574	11.39990234	1.024842854	2	3.507534364	1.087442233	2	0.935049484	1.062432678	2	6.338071767	0.903291843	2	10.09983211	0.958413781	2	4.184362447	0.94041614	2	18.37411148	Quantified
Q9R1T2	SUMO-activating enzyme subunit 1	Sae1	FUNCTION: The heterodimer acts as an E1 li	SUMO-activating enzyme s	12.57	3	3	3	350	38.59560758	5.363769531	1.044501055	3	2.593572982	1.030453141	3	2.419790294	1.009372524	3	0.705536189	0.929455099	3	1.975960455	1.014289791	3	9.240211135	1.012813281	3	0.834091442	Quantified
P35979	60S ribosomal protein L12	Rpl12	FUNCTION: Binds directly to 26S ribosomal F	60S ribosomal protein L12 (	29.09	4	4	8	165	17.79352569	9.422363281	0.971419908	8	2.442897762	1.058928589	8	2.406804887	1.02965689	8	4.466706803	0.974700039	8	2.607790764	1.016463968	8	3.880037957	0.978551372	8	4.043978419	Quantified
Q6V4S5	Protein sidekick-2	Sdk2	FUNCTION: Adhesion molecule that promote	Protein sidekick-2 OS=Mus	1.84	4	4	5	2176	239.7531326	7.356933594	0.952117535	5	6.386413812	1.010377714	5	7.560019979	0.974798552	5	8.996691366	0.972096726	5	14.49090304	1.023911844	5	13.65394863	0.982046957	5	2.833195246	Quantified
Q9ES46	Beta-parvin	Parvb	FUNCTION: Adapter protein that plays a role	Beta-parvin OS=Mus musc	10.68	3	3	3	365	41.64309635	6.328613281	1.040345832	3	2.510989244	1.037196043	3	0.759651109	1.003424297	3	4.715693358	0.959590241	3	1.735837946	0.981157995	3	6.414580974	1.039221245	3	8.600432664	Quantified
Q7TNG5	Echinoderm microtubule-associated protein-like 2	Eml2	FUNCTION: Tubulin binding protein that inhib	Echinoderm microtubule-as	3.08	2	2	5	649	70.68920746	6.277832031	1.00528404	5	0.872758975	1.026970677	5	2.475836599	0.994323717	5	7.94545573	1.010634746	5	2.663948456	0.980837469	5	9.582631186	0.939439316	5	6.74284741	Quantified
Q64737	Trifunctional purine biosynthetic protein adenosine-3	Gart		Trifunctional purine biosynt	1.88	2	2	3	1010	107.4355942	6.683105469	1.035579568	3	8.835200224	0.945578893	3	3.942061003	0.939678777	3	3.335054893	1.016039201	3	1.573983861	1.119777835	3	7.557190339	0.992317813	3	6.591614036	Quantified
Q8C0C7	Phenylalanine--tRNA ligase alpha subunit	Farsa		Phenylalanine--tRNA ligase	3.94	2	2	3	508	57.56275189	8.279785156	1.047476228	3	3.898303793	1.023847574	3	0.003483506	0.989545104	3	10.04959	0.956359025	3	8.815941491	0.932350691	3	11.18705547	1.042184917	3	3.848407394	Quantified
P97351	40S ribosomal protein S3a	Rps3a	FUNCTION: May play a role during erythropo	40S ribosomal protein S3a	10.98	3	3	4	264	29.86571964	9.729980469	0.991421675	4	6.159588635	0.992943414	4	6.63549343	1.011333328	4	1.963483767	1.003986366	4	5.061481917	1.022736332	4	4.98697728	0.971252571	4	4.53511954	Quantified
O88685	26S proteasome regulatory subunit 6A	Psmc3	FUNCTION: Component of the 26S proteasor	26S proteasome regulatory	23.3	9	9	11	442	49.51755833	5.186035156	0.99420591	11	2.960373686	1.035536894	11	6.516800302	1.028634999	11	10.24635819	0.953356728	11	2.813229074	0.971829584	11	6.5959331	1.031096758	11	7.445146744	Quantified
Q99K48	Non-POU domain-containing octamer-binding protein	Nono	FUNCTION: DNA- and RNA binding protein, i	Non-POU domain-containin	16.49	7	8	13	473	54.5064274	8.953613281	0.996887647	9	4.540168871	1.053719705	9	4.495453061	1.037090875	9	10.04392094	0.974918114	9	4.569582987	0.922360288	9	4.993499844	0.963439484	9	4.051767582	Quantified
P84089	Enhancer of rudimentary homolog	Erh	FUNCTION: May have a role in the cell cycle	Enhancer of rudimentary hc	18.27	2	2	2	104	12.25100303	5.922363281	0.921987513	2	13.51794109	0.8298212	2	14.22261265	1.203426686	2	9.738812832	1.084905165	2	10.84357537	1.200432159	2	6.547128705	0.836553522	2	4.872481293	Quantified
Q6NZB0	DnaJ homolog subfamily C member 8	Dnajc8	FUNCTION: Suppresses polyglutamine (polyQ	DnaJ homolog subfamily C	16.21	4	4	5	253	29.79437445	9.056152344	0.950666104	5	3.313501164	1.03244868	5	3.557319802	0.976014377	5	0.830455105	1.062613024	5	7.662247853	1.074179353	5	4.587480598	0.957745664	5	5.653242917	Quantified

Q8VHY0	Chondroitin sulfate proteoglycan 4	Cspg4	FUNCTION: Proteoglycan playing a role in ce	Chondroitin sulfate proteogl	2.19	3	3	4	2327	252.1531613	5.439941406	0.940626662	4	14.48973689	0.971921986	4	0.913998338	0.879334831	4	13.15796021	1.127446773	4	4.048165737	1.206463619	4	26.78251103	0.944026208	4	6.278924047	Quantified
O35864	COP9 signalosome complex subunit 5	Cops5	FUNCTION: Probable protease subunit of the	COP9 signalosome comple	16.17	3	3	3	334	37.52470945	6.536621094	0.977984846	3	2.084865222	1.006504012	3	1.258484236	1.032228697	3	3.70776322	1.051342284	3	5.430078705	1.063523433	3	0.412748338	0.979600993	3	0.074629006	Quantified
Q60692	Proteasome subunit beta type-6	Psmb6	FUNCTION: Component of the 20S core prot	Proteasome subunit beta ty	12.18	3	3	4	238	25.36248176	5.109863281	0.992203637	4	3.793052743	1.011343134	4	0.992363063	1.039453759	4	5.080576976	0.988011013	4	2.222374076	0.974947776	4	6.529347445	1.022377023	4	5.127124656	Quantified
Q91ZR1	Ras-related protein Rab-4B	Rab4b	FUNCTION: Protein transport. Probably invol	Ras-related protein Rab-4B	21.13	3	4	8	213	23.6139531	6.062011719	1.014274541	3	3.410767636	1.002947019	3	0.669003795	0.97727073	3	0.393924072	0.980706963	3	2.25568737	0.937300088	3	5.199620639	1.104330631	3	5.680286116	Quantified
P62334	26S proteasome regulatory subunit 10B	Psmc6	FUNCTION: Component of the 26S proteasor	26S proteasome regulatory	7.97	3	3	4	389	44.14510118	7.488769531	0.994762302	4	3.850988703	1.011166533	4	2.127845298	0.957074391	4	5.148542045	1.005136482	4	1.681918998	1.006049768	4	6.966829736	1.021610571	4	2.587920174	Quantified
P13864	DNA (cytosine-5)-methyltransferase 1	Dnmt1	FUNCTION: Methylates CpG residues. Prefer	DNA (cytosine-5)-methyltra	1.91	3	3	4	1620	183.0735711	7.737792969	0.977521169	4	6.177448903	0.976881056	4	4.362137301	0.992259351	4	13.56984652	1.06951422	4	7.888922845	0.968010418	4	3.346261629	0.980494883	4	6.05180221	Quantified
Q00612	Glucose-6-phosphate 1-dehydrogenase X	G6pdx	FUNCTION: Cytosolic glucose-6-phosphate d	Glucose-6-phosphate 1-deh	11.26	6	6	7	515	59.2247698	6.493652344	1.007588826	6	5.795160472	1.05951621	6	5.885792803	1.02627486	6	3.336277907	0.979491902	6	3.5075532	0.976740827	6	1.714388207	0.974758999	6	3.230241127	Quantified
O35516	Neurogenic locus notch homolog protein 2	Notch2	FUNCTION: Functions as a receptor for mem	Neurogenic locus notch hor	1.7	4	4	5	2473	265.4543921	5.224121094	0.922465711	5	9.218344652	0.991652603	5	4.493334282	1.012751506	5	12.5107809	1.003419755	5	4.138649372	1.004494215	5	2.318033283	0.911004243	5	8.239476692	Quantified
Q9DCG6	Phenazine biosynthesis-like domain-containing prote	Pbld1		Phenazine biosynthesis-like	3.82	1	1	2	288	32.02757649	7.034667969	1.020106267	2	1.92787146	0.995900851	2	1.857647178	1.020435866	2	10.53357752	0.962117546	2	8.734237606	1.018795499	2	0.355655065	1.00400244	2	0.668886553	Quantified
Q03350	Thrombospondin-2	Thbs2	FUNCTION: Adhesive glycoprotein that medi	Thrombospondin-2 OS=Mu	5.2	3	5	7	1172	129.7984489	4.817871094	1.008575266	5	1.062821786	1.010167314	5	4.833190911	0.921909308	5	3.681173199	1.048109188	5	0.704402259	1.068320346	5	3.794734824	1.013474489	5	5.424050148	Quantified
P48774	Glutathione S-transferase Mu 5	Gstm5	FUNCTION: Conjugation of reduced glutathio	Glutathione S-transferase M	8.93	2	2	2	224	26.61724595	7.210449219	0.912800687	2	6.644072316	0.995378443	2	0.354636488	0.940564445	2	6.349165438	1.000872039	2	4.069926735	1.073610041	2	7.561093488	1.136559567	2	1.385872588	Quantified
P16301	Phosphatidylcholine-sterol acyltransferase	Lcat	FUNCTION: Central enzyme in the extracellu	Phosphatidylcholine-sterol a	2.28	1	1	5	438	49.71517934	6.430175781	0.983975413	5	8.711170446	1.019958613	5	7.47529502	0.996029256	5	9.59282957	0.993719464	5	2.923589343	1.043242828	5	5.284856721	0.992715402	5	5.876437173	Quantified
P61021	Ras-related protein Rab-5B	Rab5b	FUNCTION: Protein transport. Probably invol	Ras-related protein Rab-5B	5.58	1	1	3	215	23.69190144	8.133300781	0.999198091	3	6.797518405	0.973734076	3	4.333425011	1.027715054	3	0.231676168	1.071853962	3	1.595811807	0.982693499	3	6.614723341	0.967979456	3	4.443336379	Quantified
P99027	60S acidic ribosomal protein P2	Rplp2	FUNCTION: Plays an important role in the elc	60S acidic ribosomal protei	49.57	3	3	4	115	11.64383232	4.538574219	1.043629501	4	2.474316892	0.917482963	4	3.155867845	0.989189737	4	7.300490882	1.083391044	4	4.035577288	1.13261461	4	8.170896098	0.928110496	4	1.129690727	Quantified
P56959	RNA-binding protein FUS	Fus	FUNCTION: DNA/RNA-binding protein that pl	RNA-binding protein FUS C	10.81	4	4	7	518	52.64151096	9.363769531	1.034967636	7	1.074820002	1.021196951	7	8.938313388	1.009878403	7	6.302231053	1.00044801	7	8.242581219	0.965343548	7	16.41362685	0.903588405	7	15.97893059	Quantified
P70349	Histidine triad nucleotide-binding protein 1	Hint1	FUNCTION: Hydrolyzes purine nucleotide ph	Histidine triad nucleotide-bi	16.67	2	2	2	126	13.7680993	6.873535156	1.050426572	2	1.662329711	1.005984922	2	2.365338908	1.030809754	2	4.482446715	1.015612822	2	6.170041692	0.956053776	2	0.845068558	0.935643147	2	0.164754238	Quantified
P51437	Cathelicidin antimicrobial peptide	Camp	FUNCTION: Acts as a potent antimicrobial pe	Cathelicidin antimicrobial p	19.77	2	2	5	172	19.44115659	8.675292969	1.046394256	5	2.979162361	0.97096024	5	5.41876259	0.94345305	5	6.211057094	1.07568945	5	6.477126403	1.043980698	5	13.50830094	0.981716125	5	0.903855587	Quantified
P56399	Ubiquitin carboxyl-terminal hydrolase 5	Usp5	FUNCTION: Cleaves linear and branched mu	Ubiquitin carboxyl-terminal	7.11	5	5	7	858	95.77235949	5.008300781	1.025066523	6	6.239551866	1.008179785	6	7.117995111	1.062030083	6	5.320175108	0.962937081	6	1.211316983	1.006187561	6	4.103127349	0.9775521	6	1.790039431	Quantified
O08784	Treacle protein	Tcof1	FUNCTION: Nucleolar protein that acts as a r	Treacle protein OS=Mus mu	2.35	3	3	3	1320	134.9208578	9.349121094	0.966302736	3	2.088939961	0.912353976	3	17.980052	1.080654059	3	4.960251406	1.194115749	3	1.472366994	0.926514193	3	8.825295832	0.912406316	3	1.709242969	Quantified
Q9CZ30	Obg-like ATPase 1	Ola1	FUNCTION: Hydrolyzes ATP, and can also h	Obg-like ATPase 1 OS=Mu	12.37	5	5	8	396	44.7012851	7.811035156	0.999418665	8	1.973996631	1.018339773	8	5.198902879	1.019063212	8	4.761016253	0.987420811	8	10.13785781	0.993073318	8	13.78461882	1.023341313	8	6.538284184	Quantified
Q02013	Aquaporin-1	Aqp1	FUNCTION: Forms a water-specific channel t	Aquaporin-1 OS=Mus musc	10.78	2	2	2	269	28.77521427	7.430175781	1.031586042	2	9.894543573	0.872557023	2	22.24021455	1.075458775	2	4.303007629	1.000919216	2	7.13392901	1.009039432	2	1.646209434	1.020658783	2	7.456057651	Quantified
P49312	Heterogeneous nuclear ribonucleoprotein A1	Hnrnpa1	FUNCTION: Involved in the packaging of pre-	Heterogeneous nuclear ribc	15	3	4	10	320	34.17525569	9.231933594	1.014349499	7	3.077245152	1.034051474	7	2.701909276	0.998124399	7	4.193013572	1.024415323	7	2.436073258	0.935785527	7	4.710137745	0.977839938	7	3.659524346	Quantified
Q8QZY1	Eukaryotic translation initiation factor 3 subunit L	Eif3l	FUNCTION: Component of the eukaryotic trai	Eukaryotic translation initiat	6.21	3	3	4	564	66.57032328	6.442871094	0.980183053	4	7.913173637	0.957564348	4	10.63301314	1.029931047	4	5.628604025	1.015700429	4	8.101181181	1.105388594	4	8.711792165	0.932364036	4	3.453053713	Quantified
Q9CZU3	Exosome RNA helicase MTR4	Mtrex	FUNCTION: Catalyzes the ATP-dependent ur	Exosome RNA helicase MT	2.02	2	2	3	1040	117.5612694	6.404785156	0.937699654	3	2.405841637	0.981257245	3	9.626354361	1.016238226	3	3.518514283	1.02659708	3	6.322117685	1.016463281	3	0.89608812	0.964344654	3	3.003364025	Quantified
Q99LG2	Transportin-2	Tnpo2	FUNCTION: Probably functions in nuclear prc	Transportin-2 OS=Mus mus	4.28	2	3	4	887	100.3907108	4.982910156	1.010355222	3	4.278104154	0.998169814	3	2.666423681	0.951155012	3	0.256603566	0.945664551	3	0.615241538	0.957153924	3	0.753883834	1.057305143	3	4.825023034	Quantified
Q9D1D6	Collagen triple helix repeat-containing protein 1	Cthrc1	FUNCTION: May act as a negative regulator	Collagen triple helix repeat-	11.43	3	3	3	245	26.44336698	7.503417969	0.993757294	3	3.139640127	1.039226126	3	4.425788362	1.010658424	3	8.871241862	0.973868189	3	0.11911078	0.985463761	3	4.995164807	0.990972186	3	1.293689225	Quantified
Q9DBT5	AMP deaminase 2	Ampd2	FUNCTION: AMP deaminase plays a critical	AMP deaminase 2 OS=Mus	5.76	4	4	6	798	91.9653807	6.227050781	0.945797331	6	9.842674281	0.995365005	6	2.726972908	0.975103394	6	5.46911088	1.005498227	6	3.546668095	1.059910778	6	7.81185074	1.008650791	6	5.735981352	Quantified
Q9CPP0	Nucleoplasmin-3	Npm3	FUNCTION: Plays a role in the regulation of c	Nucleoplasmin-3 OS=Mus	9.14	1	1	1	175	19.01131394	4.817871094																			Identified
Q64511	DNA topoisomerase 2-beta	Top2b	FUNCTION: Control of topological states of D	DNA topoisomerase 2-beta	4.16	5	7	10	1612	181.7950175	8.294433594	0.954716383	6	2.902512139	0.922990457	6	4.867984307	0.959912688	6	3.350374991	1.134247691	6	3.698690999	1.027118262	6	9.510604761	1.002249112	6	5.569612231	Quantified
Q3U1J4	DNA damage-binding protein 1	Ddb1	FUNCTION: Protein, which is both involved ir	DNA damage-binding prote	5.96	8	8	14	1140	126.7722653	5.262207031	1.031859957	14	4.772375437	1.007604773	14	3.556913033	1.024687738	14	6.90196111	0.986018113	14	3.30177388	0.980293257	14	10.83095278	0.968372558	14	4.063489559	Quantified
Q91WP0	Mannan-binding lectin serine protease 2	Masp2	FUNCTION: Serum protease that plays an im	Mannan-binding lectin serir	1.9	1	1	2	685	75.46787881	6.074707031	0.99079293	2	6.513371473	0.98581612	2	2.096629021	1.115644338	2	8.846424249	0.974361667	2	2.503561043	0.907023046	2	3.910509982	0.992999817	2	2.585135381	Quantified
P61327	Protein mago nashi homolog	Magoh	FUNCTION: Required for pre-mRNA splicing	Protein mago nashi homolo	28.77	4	4	4	146	17.15278709	6.112792969	0.947661776	3	5.400946562	0.992243485	3	2.785527579	1.025108313	3	1.637094776	0.984274457	3	7.299313992	1.069779545	3	1.424735293	1.013984725	3	5.119959498	Quantified
P70195	Proteasome subunit beta type-7	Psmb7	FUNCTION: Component of the 20S core prot	Proteasome subunit beta ty	13.72	3	3	4	277	29.87225325	7.986816406	1.011420985	4	4.02521181	0.997041275	4	1.414438472	0.961248279	4	5.580377104	1.039637772	4	4.146098831	1.026750381	4	2.104200007	0.987356585	4	2.416198418	Quantified
P97310	DNA replication licensing factor MCM2	Mcm2	FUNCTION: Acts as component of the MCM2	DNA replication licensing fa	1.44	1	1	1	904	102.013354	5.719238281	1.059930538	1		0.98385262	1		1.125583353	1		0.986782563	1		0.957570375	1		0.872464865	1		Quantified
Q9Z2L7	Cytokine receptor-like factor 3	Crlf3	FUNCTION: May play a role in the negative r	Cytokine receptor-like facto	9.95	4	4	4	442	49.52779102	5.033691406	1.015390222	4	4.489158966	1.031646792	4	1.807506781	1.066443369	4	5.440041084	0.973369674	4	3.979992419	0.914673067	4	6.0999161	0.995257181	4	5.339177919	Quantified
P62315	Small nuclear ribonucleoprotein Sm D1	Snrpd1	FUNCTION: Plays role in pre-mRNA splicing	Small nuclear ribonucleopro	20.17	2	2	2	119	13.27336469	11.56103516	0.983323393	2	0.082833911	0.996344091	2	4.270999848	0.957814158	2	6.00130706	1.005538871	2	4.680724909	1.081528404	2	6.023913946	1.033524858	2	0.836551828	Quantified
P60670	Nuclear protein localization protein 4 homolog	Nploc4	FUNCTION: The ternary complex containing	Nuclear protein localization	2.3	2	2	2	608	67.97397365	6.455566406	1.014321923	1		1.016524189	1		0.977013435	1		1.066462168	1		0.923149301	1		0.99242543	1		Quantified
Q8CIB5	Fermitin family homolog 2	Fermt2	FUNCTION: Scaffolding protein that enhance	Fermitin family homolog 2 C	14.12	8	8	10	680	77.75050889	6.697753906	1.015549704	10	3.359605781	0.998736439	10	6.635428525	1.018664697	10	6.755361756	0.980741884	10	7.262706483	0.966470101	10	4.558316524	0.970788891	10	2.865639918	Quantified
Q9DBC7	cAMP-dependent protein kinase type I-alpha regulato	Prkar1a	FUNCTION: Regulatory subunit of the cAMP-	cAMP-dependent protein ki	12.34	3	3	3	381	43.15803339	5.351074219	1.097046291	3	8.582627981	0.912784438	3	19.08148606	1.060892777	3	26.15082134	0.985581812	3	10.7852011	1.048995403	3	11.73363199	0.90954928	3	4.18016387	Quantified
Q2L4X1	Basic leucine zipper and W2 domain-containing prote	Bzw2	FUNCTION: May be involved in neuronal diffe	Basic leucine zipper and W	8.35	2	3	4	419	48.03290676	6.683105469	1.029799411	3	0.489131468	1.012680583	3	0.608507972	0.980353587	3	0.2661905	1.023777307	3	1.549995124	0.979145923	3	0.058079798	1.014000858	3	1.13826922	Quantified
Q99JW4	LIM and senescent cell antigen-like-containing doma	Lims1	FUNCTION: Adapter protein in a cytoplasmic	LIM and senescent cell anti	6.15	2	2	5	325	37.21482836	8.045410156	0.987586629	5	4.415379528	1.009346368	5	4.24427796	1.00018511	5	5.259287334	1.00220561	5	6.036185812	1.041183367	5	9.689841952	0.989439746	5	3.822169032	Quantified
O88796	Ribonuclease P protein subunit p30	Rpp30	FUNCTION: Component of ribonuclease P, a	Ribonuclease P protein sub	3.36	1	1	1	268	29.45481573	9.056152344																			Identified
Q6PEB6	MOB-like protein phocein	Mob4	FUNCTION: May play a role in membrane tra	MOB-like protein phocein C	13.78	2	2	3	225	26.01558685	5.782714844	1.006782611	3	4.626753615	0.925883175	3	7.652310662	0.896216792	3	3.843432002	0.984129834	3	6.700136812	1.047989404	3	8.534790274	1.048801132	3	9.285246357	Quantified
Q769J6	A disintegrin and metalloproteinase with thrombospo	Adamts13	FUNCTION: Cleaves the vWF multimers in pl	A disintegrin and metallopro	1.26	2	2	3	1426	155.2559328	6.565917969	1.007982845	3	10.84984184	1.111482148	3	3.383704932	0.977527949	3	1.040509084	0.862457653	3	4.171776681	0.818291208	3	10.86224849	1.052467761	3	8.716164289	Quantified
O54865	Guanylate cyclase soluble subunit beta-1	Gucy1b1	FUNCTION: Mediates responses to nitric oxic	Guanylate cyclase soluble s	8.39	4	4	4	620	70.55262136	5.312988281	1.02268604	4	6.932437382	1.013186553	4	5.121561285	0.955750265	4	9.184968208	1.004718111	4	3.285721076	0.97802855	4	4.347795392	1.052662424	4	3.571889606	Quantified
P17427	AP-2 complex subunit alpha-2	Ap2a2	FUNCTION: Component of the adaptor protei	AP-2 complex subunit alpha	4.16	4	4	6	938	103.9510639	6.932128906	1.008683735	6	3.077741391	0.990166346	6	4.823092315	0.975053741	6	1.967200381	1.012446929	6	2.973471823	1.018253645	6	3.98227744	0.991175	6	2.82445999	Quantified
Q8BG32	26S proteasome non-ATPase regulatory subunit 11	Psmd11	FUNCTION: Component of the 26S proteasor	26S proteasome non-ATPa	13.98	5	5	7	422	47.40703188	6.480957031	0.995739145	6	5.692291449	1.044790632	6	4.471570857	0.988557811	6	10.42438578	0.983598852	6	1.89346776	0.995437289	6	9.697043155	0.990190648	6	2.784322055	Quantified
Q9WU84	Copper chaperone for superoxide dismutase	Ccs	FUNCTION: Delivers copper to copper zinc s	Copper chaperone for supe	4.74	1	1	1	274	28.89340326	6.100097656	1.009586188	1		0.993040929	1		0.906310314	1		1.045767011	1		0.991595716	1		1.083787825	1		Quantified
P02104	Hemoglobin subunit epsilon-Y2	Hbb-y	FUNCTION: Hemoglobin epsilon chain is a b	Hemoglobin subunit epsilor	12.93	1	2	4	147	16.12643766	8.177246094	0.943793549	2	6.22135073	0.915908228	2	4.941075815	0.998657776	2	2.267880241	0.999450415	2	0.453417255	1.069037667	2	9.896724832	1.129328194	2	6.101936636	Quantified
O70318	Band 4.1-like protein 2	Epb41l2	FUNCTION: Required for dynein-dynactin cor	Band 4.1-like protein 2 OS=	6.78	5	5	5	988	109.8725909	5.427246094	0.988590777	5	1.453262324	1.006820694	5	4.426427313	0.915540847	5	1.149063313	1.112908747	5	5.489626632	1.028729655	5	4.467580491	1.001902706	5	1.730393768	Quantified
Q6PDG5	SWI/SNF complex subunit SMARCC2	Smarcc2	FUNCTION: Involved in transcriptional activat	SWI/SNF complex subunit S	4.78	5	5	8	1213	132.5215507	5.592285156	0.99734461	8	9.062160498	0.945620223	8	0.630316026	0.963094681	8	7.106210878	1.022679283	8	3.473845761	1.034784881	8	8.7834534	1.027063229	8	3.842099747	Quantified
P31938	Dual specificity mitogen-activated protein kinase kina	Map2k1	FUNCTION: Dual specificity protein kinase wl	Dual specificity mitogen-act	10.18	4	4	6	393	43.44635592	6.697753906	1.041266745	6	5.434268851	1.022609234	6	4.372539657	0.963627567	6	7.209066556	0.991806054	6	7.898955832	0.931961599	6	8.160809528	0.983517485	6	1.84987095	Quantified
Q8VDM4	26S proteasome non-ATPase regulatory subunit 2	Psmd2	FUNCTION: Component of the 26S proteasor	26S proteasome non-ATPa	10.79	10	10	17	908	100.138677	5.173339844	1.015770686	17	5.902529178	0.983542803	17	7.172066742	0.991047317	17	7.735578192	0.987283559	17	7.638021172	1.053631434	17	7.317115504	0.98128221	17	2.716669782	Quantified
Q62356	Follistatin-related protein 1	Fstl1	FUNCTION: May modulate the action of some	Follistatin-related protein 1	8.5	3	3	4	306	34.53159396	5.846191406	0.957457726	4	2.068674823	1.011688701	4	2.510815784	1.023223184	4	3.285941099	0.972321807	4	8.985065119	0.988786837	4	5.131735776	1.020773576	4	3.641235942	Quantified
P31230	Aminoacyl tRNA synthase complex-interacting multifu	Aimp1	FUNCTION: Non-catalytic component of the r	Aminoacyl tRNA synthase c	3.87	1	1	1	310	33.97587002	8.353027344	1.02822721	1		1.022924668	1		1.036153176	1		0.962163685	1		0.980564985	1		0.975522971	1		Quantified
Q02788	Collagen alpha-2(VI) chain	Col6a2	FUNCTION: Collagen VI acts as a cell-bindin	Collagen alpha-2(VI) chain	4.45	4	4	5	1034	110.265699	6.417480469	0.985922753	5	1.143901827	0.950993413	5	5.447324802	0.945505402	5	5.858031194	1.10086083	5	6.509150825	1.007024711	5	4.62274973	1.004096072	5	7.963175233	Quantified
P34022	Ran-specific GTPase-activating protein	Ranbp1	FUNCTION: Plays a role in RAN-dependent n	Ran-specific GTPase-activa	5.42	1	1	3	203	23.58171052	5.224121094	0.954896894	3	4.209209694	0.78788131	3	6.184181581	1.115013124	3	11.40628628	1.126915588	3	1.501839216	1.209666019	3	3.415239128	0.949406543	3	2.703751208	Quantified
Q8BTZ7	Mannose-1-phosphate guanyltransferase beta	Gmppb	FUNCTION: Catalyzes the formation of GDP-	Mannose-1-phosphate guar	9.44	3	3	4	360	39.8906107	6.741699219	0.97565834	4	4.847513278	1.004212587	4	1.41179972	0.993664897	4	9.606773549	1.026670415	4	5.075164138	1.006464248	4	1.436205569	0.976147312	4	4.830439942	Quantified
Q9R0Q7	Prostaglandin E synthase 3	Ptges3	FUNCTION: Cytosolic prostaglandin synthase	Prostaglandin E synthase 3	20	3	3	3	160	18.70941938	4.551269531	0.946608439	3	2.596145136	1.05762117	3	1.764208866	1.188829271	3	3.123554219	0.900458746	3	0.244541685	0.955899799	3	5.728255538	0.922377707	3	0.890009696	Quantified
Q9CR57	60S ribosomal protein L14	Rpl14	FUNCTION: Component of the large ribosom	60S ribosomal protein L14 (	5.53	1	1	2	217	23.54920416	11.01904297	0.939419998	2	9.802260795	0.940438194	2	9.256331059	0.896144155	2	12.18136805	0.985086047	2	9.309948625	1.175197234	2	36.36210292	1.143739089	2	13.87173466	Quantified
Q9JKK7	Tropomodulin-2	Tmod2	FUNCTION: Blocks the elongation and depol	Tropomodulin-2 OS=Mus m	1.99	1	1	7	351	39.48662245	5.351074219	0.930915812	7	3.253362666	0.985008024	7	1.562556654	1.030000199	7	1.749077007	0.967906916	7	5.903597089	1.120398144	7	5.725701161	0.937769345	7	5.729448998	Quantified
P08249	Malate dehydrogenase, mitochondrial	Mdh2		Malate dehydrogenase, mit	12.13	4	4	7	338	35.58876027	8.675292969	1.033337039	7	2.914531691	1.029273878	7	1.937894881	1.013039052	7	4.106492058	0.977844577	7	4.708331824	0.975367639	7	4.408613958	1.01300073	7	2.745021441	Quantified
Q8VE37	Regulator of chromosome condensation	Rcc1	FUNCTION: Guanine-nucleotide releasing fac	Regulator of chromosome c	6.89	2	2	2	421	44.9027473	8.104003906	0.982259045	2	5.527696369	0.945042863	2	2.083528044	1.010022513	2	1.752110776	1.06808402	2	4.993599182	0.931660154	2	9.075142929	1.057928745	2	0.630894324	Quantified
P70389	Insulin-like growth factor-binding protein complex aci	Igfals	FUNCTION: May have an important role in re	Insulin-like growth factor-bir	2.32	1	1	1	603	66.91790958	6.595214844	0.954681028	1		1.039109919	1		1.011995688	1		0.905598524	1		1.157571443	1		1.013264789	1		Quantified
O70172	Phosphatidylinositol 5-phosphate 4-kinase type-2 alp	Pip4k2a	FUNCTION: Catalyzes the phosphorylation o	Phosphatidylinositol 5-phos	8.15	1	3	3	405	46.12246888	6.990722656	0.951846377	1		0.93020403	1		0.874757155	1		1.243662925	1		1.089401306	1		0.988467249	1		Quantified
P27612	Phospholipase A-2-activating protein	Plaa	FUNCTION: Plays a role in protein ubiquitinat	Phospholipase A-2-activatir	7.43	5	5	6	794	87.16556685	6.138183594	0.997795468	6	2.023463198	0.979940961	6	1.943951851	1.013877095	6	3.755425916	0.996829651	6	6.882655071	1.047830095	6	5.888996594	1.002999884	6	7.958373655	Quantified
P15306	Thrombomodulin	Thbd	FUNCTION: Thrombomodulin is a specific en	Thrombomodulin OS=Mus r	4.33	3	3	4	577	61.826515	4.602050781	0.915523542	4	6.220803365	0.920529558	4	10.8863267	1.04555981	4	8.381208321	1.089205289	4	8.677346223	0.904415544	4	1.233605188	0.977569868	4	3.492040566	Quantified
Q9WUM3	Coronin-1B	Coro1b	FUNCTION: Regulates leading edge dynamic	Coronin-1B OS=Mus muscu	7.85	5	5	6	484	53.87800221	5.782714844	1.00419817	6	3.799668517	1.010415876	6	6.123710786	1.015856145	6	3.927771212	1.020712676	6	6.000820749	0.964308492	6	5.957431851	1.000319236	6	4.054358786	Quantified
Q9ERL7	Glia maturation factor gamma	Gmfg		Glia maturation factor gamr	21.13	2	3	6	142	16.73754228	5.706542969	0.972236917	5	2.53469199	0.87149674	5	5.519903316	1.147985851	5	6.315568228	1.051736281	5	8.98708971	1.068840197	5	2.859819886	0.941819967	5	8.374105879	Quantified
Q3U962	Collagen alpha-2(V) chain	Col5a2	FUNCTION: Type V collagen is a member of	Collagen alpha-2(V) chain (	1.94	2	3	10	1497	144.9289142	6.697753906	0.992836365	9	3.097178544	1.019395311	9	3.310554884	0.976444991	9	4.732768591	1.003916399	9	4.796134847	1.003207089	9	7.825682746	0.998860606	9	1.526096181	Quantified
Q3THS6	S-adenosylmethionine synthase isoform type-2	Mat2a	FUNCTION: Catalyzes the formation of S-ade	S-adenosylmethionine synt	14.43	5	5	10	395	43.66136784	6.480957031	1.019783726	10	2.008901439	1.028145727	10	5.772431221	0.990862671	10	5.026874897	1.009325851	10	4.699783399	0.949935607	10	8.597189539	1.027744353	10	2.040093954	Quantified
O35381	Acidic leucine-rich nuclear phosphoprotein 32 family	Anp32a	FUNCTION: Implicated in a number of cellula	Acidic leucine-rich nuclear p	14.57	1	5	10	247	28.52024376	4.068847656	0.987770408	2	4.813862662	1.034615236	2	5.841661975	1.03312173	2	11.48263143	0.87129011	2	4.637474448	1.121568692	2	15.35616281	1.00667902	2	5.542110279	Quantified
Q9WVE8	Protein kinase C and casein kinase substrate in neur	Pacsin2	FUNCTION: Regulates the morphogenesis ar	Protein kinase C and casein	11.11	6	6	6	486	55.79821987	5.198730469	1.024474153	6	5.313974299	1.008270213	6	6.585810673	0.96887958	6	3.504728572	1.041416022	6	4.023206589	0.941038381	6	8.170051432	0.974761567	6	8.150171926	Quantified
P62307	Small nuclear ribonucleoprotein F	Snrpf	FUNCTION: Plays role in pre-mRNA splicing	Small nuclear ribonucleopro	15.12	1	1	2	86	9.718777215	4.665527344	1.014784261	2	0.651409738	0.941590273	2	0.220280711	1.032331303	2	9.43827247	1.03666761	2	0.10945975	1.042647565	2	0.14772537	0.96553355	2	10.51463259	Quantified
Q91Y47	Coagulation factor XI	F11	FUNCTION: Factor XI triggers the middle pha	Coagulation factor XI OS=M	4.33	3	3	10	624	69.74309957	8.250488281	1.001434056	10	4.941317594	1.033832579	10	7.393192959	1.007466143	10	13.62874235	0.987626019	10	17.14675773	0.952217509	10	10.79767923	0.960162692	10	3.222397167	Quantified
Q80TY0	Formin-binding protein 1	Fnbp1	FUNCTION: Required to coordinate membran	Formin-binding protein 1 OS	4.38	3	3	3	616	71.29948375	5.668457031	1.043893545	3	0.204780115	1.021651905	3	6.906263965	1.040699185	3	0.966695074	0.976263489	3	0.219899793	0.985464367	3	5.680728607	0.983381114	3	1.836309149	Quantified
P10649	Glutathione S-transferase Mu 1	Gstm1	FUNCTION: Conjugation of reduced glutathio	Glutathione S-transferase M	14.22	3	3	3	218	25.95305836	7.942871094	1.013592008	3	11.18047313	1.046024259	3	7.118691444	0.9828737	3	2.954080899	1.004114454	3	1.711182559	0.957244257	3	23.83351096	1.061994938	3	2.899302322	Quantified
Q9CXW3	Calcyclin-binding protein	Cacybp	FUNCTION: May be involved in calcium-depe	Calcyclin-binding protein O	19.65	4	5	17	229	26.49375286	7.869628906	0.992585573	5	3.903809424	1.03741019	5	8.081936146	1.016584267	5	3.853812941	0.979967203	5	5.117063814	1.034199111	5	4.286027216	0.987413259	5	0.841033252	Quantified
P48036	Annexin A5	Anxa5	FUNCTION: This protein is an anticoagulant p	Annexin A5 OS=Mus musc	23.82	8	9	14	319	35.73020868	4.957519531	0.989798179	13	3.57437458	0.972488766	13	4.951664642	1.134885125	13	8.606537517	0.969107372	13	2.039883848	0.930634147	13	11.8782567	0.95171244	13	5.081502644	Quantified
Q8VBW6	NEDD8-activating enzyme E1 regulatory subunit	Nae1	FUNCTION: Regulatory subunit of the dimeric	NEDD8-activating enzyme	5.62	2	2	2	534	60.23568835	5.516113281	1.006976327	2	1.113796273	1.013249543	2	4.033957521	0.949288819	2	0.507810628	1.017735527	2	1.863703798	1.062073872	2	5.818100096	1.000427889	2	1.756936335	Quantified
P08121	Collagen alpha-1(III) chain	Col3a1	FUNCTION: Collagen type III occurs in most	Collagen alpha-1(III) chain	3.01	4	4	12	1464	138.8576276	6.521972656	1.002235625	12	4.766196754	0.998674978	12	4.224776166	0.987903167	12	5.637221852	0.986917318	12	4.934006065	1.029530196	12	9.712016632	1.020459154	12	4.814790624	Quantified
P61226	Ras-related protein Rap-2b	Rap2b	FUNCTION: Small GTP-binding protein which	Ras-related protein Rap-2b	11.48	2	2	3	183	20.49120867	4.805175781	0.994168005	3	2.433185951	0.96704776	3	3.102639089	0.961574134	3	1.4620922	1.059295647	3	2.150535778	1.064518111	3	6.495115412	1.028001896	3	1.066652473	Quantified
O54774	AP-3 complex subunit delta-1	Ap3d1	FUNCTION: Part of the AP-3 complex, an ada	AP-3 complex subunit delta	2.34	2	2	2	1199	134.9959896	7.371582031	0.944559841	2	8.131402205	0.986049428	2	0.090460762	1.155271818	2	3.542141697	0.898941378	2	6.790010154	1.074834698	2	4.076574746	0.972807974	2	0.152185532	Quantified
P62242	40S ribosomal protein S8	Rps8		40S ribosomal protein S8 C	12.98	3	3	7	208	24.19016371	10.31591797	1.025029102	7	2.104252831	1.022363075	7	2.854137218	0.998794045	7	3.471426257	1.010845263	7	4.52316682	1.020527803	7	12.02208586	0.992509284	7	2.937886405	Quantified
Q9JKX3	Transferrin receptor protein 2	Tfr2	FUNCTION: Mediates cellular uptake of trans	Transferrin receptor protein	1.13	1	1	2	798	88.3469007	6.125488281	0.953263743	2	4.313471489	0.986955645	2	0.26864441	0.961206339	2	4.139141104	1.007252396	2	2.047490872	1.041857305	2	0.07098791	1.093936729	2	6.448498413	Quantified
Q9D2V7	Coronin-7	Coro7	FUNCTION: F-actin regulator involved in ante	Coronin-7 OS=Mus muscul	4.56	4	4	5	922	100.7493538	5.770019531	0.992325155	5	1.28929344	0.998433771	5	5.536562667	1.037482101	5	2.175101442	0.993895405	5	5.626997559	0.934365257	5	7.290176562	0.991125119	5	2.764852085	Quantified
Q61543	Golgi apparatus protein 1	Glg1	FUNCTION: Binds fibroblast growth factor an	Golgi apparatus protein 1 C	3.57	4	4	4	1175	133.6461537	6.844238281	0.942389574	4	8.121330951	0.979958906	4	1.93671066	1.01231233	4	8.479194164	1.093298578	4	11.28963385	0.947678784	4	2.659589813	1.017135411	4	7.782115471	Quantified
Q99J77	Sialic acid synthase	Nans	FUNCTION: Produces N-acetylneuraminate-9	Sialic acid synthase OS=M	6.96	2	2	3	359	39.99836292	7.063964844	1.003413844	3	7.34699961	0.984855698	3	4.146111949	0.852876766	3	27.17354669	1.128191389	3	4.20577886	1.102325107	3	27.97062728	1.059916659	3	3.56482129	Quantified
Q3TIR3	Synembryn-A	Ric8a	FUNCTION: Guanine nucleotide exchange fa	Synembryn-A OS=Mus mus	2.08	1	1	2	530	59.80861093	5.681152344	1.046112155	2	1.710936536	1.021503501	2	1.580123639	0.993958973	2	0.523032014	0.951148075	2	1.396166428	0.886882165	2	3.82250505	1.073398755	2	3.318463004	Quantified
Q922D4	Serine/threonine-protein phosphatase 6 regulatory su	Ppp6r3	FUNCTION: Regulatory subunit of protein pho	Serine/threonine-protein ph	2.13	1	1	1	844	94.59331091	4.576660156	1.137777719	1		0.939307497	1		0.99140549	1		0.981116526	1		1.088801205	1		0.920072884	1		Quantified
Q6PHN9	Ras-related protein Rab-35	Rab35	FUNCTION: The small GTPases Rab are key	Ras-related protein Rab-35	14.43	1	3	7	201	23.01076773	8.294433594	1.028257045	1		0.961896047	1		1.034829839	1		1.034952112	1		0.934733931	1		0.997623159	1		Quantified
Q9EST5	Acidic leucine-rich nuclear phosphoprotein 32 family	Anp32b	FUNCTION: Multifunctional protein working a	Acidic leucine-rich nuclear p	11.76	2	6	12	272	31.06007519	4.005371094	0.96711664	4	5.088560975	1.024902981	4	1.8959053	1.083581058	4	6.338213565	0.881416346	4	8.775804574	0.945232824	4	5.351386578	1.022249484	4	11.20954791	Quantified
P06745	Glucose-6-phosphate isomerase	Gpi	FUNCTION: In the cytoplasm, catalyzes the c	Glucose-6-phosphate isom	6.27	3	3	3	558	62.72718006	8.133300781	0.993920841	3	0.903247472	1.04137607	3	2.507008606	1.033488049	3	1.162251552	0.916053485	3	4.606930866	1.016560146	3	3.859935875	1.010141462	3	2.241376626	Quantified
Q99J45	Nuclear receptor-binding protein	Nrbp1	FUNCTION: May play a role in subcellular tra	Nuclear receptor-binding pr	2.24	1	1	2	535	59.82814208	5.084472656	1.031460367	2	1.026577009	0.939396734	2	4.339746482	0.989655927	2	0.997231958	1.052031679	2	5.417987555	1.023312152	2	2.279993279	0.997562522	2	6.710791473	Quantified
Q9QXZ0	Microtubule-actin cross-linking factor 1	Macf1	FUNCTION: [Isoform 2]: F-actin-binding prote	Microtubule-actin cross-link	1.02	6	6	6	7354	831.3616886	5.427246094	1.009004093	6	4.748287238	0.970020225	6	1.571052571	0.998369617	6	1.145391625	0.994721145	6	6.427653107	0.981685219	6	7.308648588	1.026594514	6	6.296464814	Quantified
P13707	Glycerol-3-phosphate dehydrogenase [NAD(+)], cyto	Gpd1	FUNCTION: Has glycerol-3-phosphate dehyd	Glycerol-3-phosphate dehy	8.31	2	3	5	349	37.54842767	7.166503906	0.983438046	4	2.81408849	1.019271051	4	2.988173224	0.961723528	4	1.276528821	0.986544188	4	3.545501387	1.033754247	4	8.065177816	0.986781494	4	4.288232935	Quantified
Q6DFW4	Nucleolar protein 58	Nop58	FUNCTION: Required for 60S ribosomal subu	Nucleolar protein 58 OS=M	9.33	3	3	4	536	60.3047192	8.338378906	1.016976274	4	2.403233177	0.970757738	4	7.107570299	0.966232799	4	7.926356129	1.083321131	4	4.651906029	0.99533191	4	10.41570426	1.008921601	4	1.1945612	Quantified
P13609	Serglycin	Srgn	FUNCTION: Plays a role in formation of mast	Serglycin OS=Mus musculu	10.53	1	1	2	152	16.70082708	4.411621094	1.009280382	2	7.738506152	0.883966554	2	4.580173804	1.061936487	2	3.714333588	1.080929196	2	4.641371742	0.91523695	2	3.619836501	1.029291901	2	14.89705366	Quantified
Q9Z0Y1	Dynactin subunit 3	Dctn3	FUNCTION: Together with dynein may be inv	Dynactin subunit 3 OS=Mus	13.44	3	3	3	186	20.96529738	6.062011719	1.065983347	2	6.518081458	0.937479664	2	4.833906557	0.967895617	2	3.177795876	1.020754621	2	8.476387854	1.053193943	2	2.321218437	1.001001357	2	1.544877031	Quantified
Q6PER3	Microtubule-associated protein RP/EB family membe	Mapre3	FUNCTION: Plus-end tracking protein (+TIP)	Microtubule-associated pro	9.25	1	2	3	281	31.94596999	5.541503906																			Identified
P32921	Tryptophan--tRNA ligase, cytoplasmic	Wars1	FUNCTION: T1-TrpRS has aminoacylation ac	Tryptophan--tRNA ligase, c	20.79	11	11	15	481	54.32305455	6.888183594	1.000618249	15	4.537685082	0.976922606	15	4.755513124	1.003840343	15	6.571777744	1.007672	15	4.235422558	0.946241369	15	9.518736171	0.992120807	15	2.231743306	Quantified
P50608	Fibromodulin	Fmod	FUNCTION: Affects the rate of fibrils formatio	Fibromodulin OS=Mus mus	2.93	1	1	1	376	43.02749541	6.036621094	0.980311243	1		1.020126935	1		0.984514392	1		0.956900379	1		1.160363162	1		0.984696081	1		Quantified
Q8BPB0	MOB kinase activator 1B	Mob1b	FUNCTION: Activator of LATS1/2 in the Hipp	MOB kinase activator 1B O	10.65	2	2	3	216	25.07463932	6.727050781	0.948963267	3	0.461479495	1.034494932	3	2.477957036	1.01904838	3	2.289356472	1.00551888	3	1.303723701	0.948208514	3	7.055943333	0.982355582	3	3.420797593	Quantified
Q9D8W5	26S proteasome non-ATPase regulatory subunit 12	Psmd12	FUNCTION: Component of the 26S proteasor	26S proteasome non-ATPa	7.68	4	4	5	456	52.86148378	7.063964844	0.988926054	5	4.245338877	1.058763441	5	1.216128872	0.995751774	5	4.994155202	0.939739343	5	7.59316043	0.999066276	5	7.972859638	1.002427221	5	4.172498569	Quantified
Q6Q899	Antiviral innate immune response receptor RIG-I	Ddx58	FUNCTION: Innate immune receptor that sen	Antiviral innate immune res	4.21	3	3	4	926	105.9080382	6.653808594	1.012388661	4	6.998311728	0.9693169	4	9.437469721	1.130068488	4	5.423392778	0.976506023	4	4.612933761	0.994000005	4	16.7311403	0.968896133	4	6.353867518	Quantified
Q64442	Sorbitol dehydrogenase	Sord	FUNCTION: Polyol dehydrogenase that catal	Sorbitol dehydrogenase OS	6.44	2	2	3	357	38.22471201	7.020019531	0.969514824	3	2.253586324	1.083557534	3	1.967342104	0.990441658	3	2.848657479	0.947029993	3	9.548415908	0.947469338	3	0.454901639	0.979347547	3	1.626865999	Quantified
Q3B7Z2	Oxysterol-binding protein 1	Osbp	FUNCTION: Lipid transporter involved in lipid	Oxysterol-binding protein 1	4.1	3	3	4	805	88.74108582	7.195800781	1.007354698	4	4.3294301	0.995451406	4	1.989500251	0.976061898	4	2.25536194	1.047087979	4	1.927763185	0.982265832	4	2.555076073	1.010945704	4	2.352015919	Quantified
Q9DB05	Alpha-soluble NSF attachment protein	Napa	FUNCTION: Required for vesicular transport b	Alpha-soluble NSF attachm	6.1	1	1	2	295	33.16824999	5.452636719	0.983716727	2	11.80629542	0.931588021	2	10.37582577	1.005751576	2	7.968269409	0.980372896	2	0.100745447	1.079661854	2	3.367932834	1.071085554	2	5.225504042	Quantified
Q3ULJ0	Glycerol-3-phosphate dehydrogenase 1-like protein	Gpd1l	FUNCTION: Plays a role in regulating cardiac	Glycerol-3-phosphate dehy	11.97	3	4	5	351	38.20091757	6.770996094	0.990817293	4	1.023929045	1.051477371	4	0.534636427	1.05322308	4	3.760437506	0.964063595	4	3.025285703	1.029845687	4	4.810753688	0.935467342	4	3.856392587	Quantified
P56371	Ras-related protein Rab-4A	Rab4a	FUNCTION: Protein transport. Plays a role in	Ras-related protein Rab-4A	9.63	1	2	6	218	24.39319321	6.074707031	1.060718278	1		0.997411177	1		1.056115553	1		0.887659876	1		1.019095476	1		1	1		Quantified
P35279	Ras-related protein Rab-6A	Rab6a	FUNCTION: Protein transport. Regulator of m	Ras-related protein Rab-6A	10.1	1	2	6	208	23.57491911	5.541503906	0.933376573	1		0.998821543	1		1.007630875	1		0.995727405	1		1.090942506	1		1.032975677	1		Quantified
Q8CAA7	Glucose 1,6-bisphosphate synthase	Pgm2l1	FUNCTION: Glucose 1,6-bisphosphate synth	Glucose 1,6-bisphosphate s	1.77	1	1	1	621	70.23433634	6.493652344	0.968500847	1		1.009035277	1		1.361588018	1		0.850216804	1		0.780327115	1		0.917423602	1		Quantified
Q9WTQ5	A-kinase anchor protein 12	Akap12	FUNCTION: Anchoring protein that mediates	A-kinase anchor protein 12	1.78	2	2	2	1684	180.5861113	4.437011719	1.06203867	2	1.623012583	0.997661026	2	7.652929104	1.131863815	2	8.390581609	0.8757501	2	2.103302433	0.850897039	2	12.16724555	1.01832662	2	8.518216146	Quantified
Q91WG8	Bifunctional UDP-N-acetylglucosamine 2-epimerase/	Gne	FUNCTION: Regulates and initiates biosynthe	Bifunctional UDP-N-acetylg	11.63	8	8	10	722	79.1479398	6.814941406	0.987185812	10	4.560275817	1.0247209	10	7.941712204	0.966557513	10	5.721695376	0.97904222	10	8.84720024	0.982122242	10	11.58243429	1.048856804	10	3.761538777	Quantified
Q8BVI4	Dihydropteridine reductase	Qdpr	FUNCTION: The product of this enzyme, tetra	Dihydropteridine reductase	6.64	1	1	1	241	25.55378425	7.811035156	1.034685557	1		1.044126048	1		0.99812071	1		0.955866811	1		0.992393853	1		0.987980623	1		Quantified
P97429	Annexin A4	Anxa4	FUNCTION: Calcium/phospholipid-binding pr	Annexin A4 OS=Mus musc	7.21	1	2	2	319	35.89312153	5.566894531	0.913595938	1		0.999333503	1		1.023257	1		1.015526713	1		0.940003268	1		1.105782542	1		Quantified
Q61220	Protein kinase C-binding protein NELL2	Nell2	FUNCTION: Required for neuron survival thro	Protein kinase C-binding pr	4.64	3	3	6	819	91.36944411	5.820800781	0.963928267	6	8.029575467	1.034544632	6	9.18715216	0.993322899	6	2.308847145	0.951930699	6	4.72852086	1.076631251	6	12.79544587	1.002490216	6	7.170282195	Quantified
Q61171	Peroxiredoxin-2	Prdx2	FUNCTION: Thiol-specific peroxidase that ca	Peroxiredoxin-2 OS=Mus m	13.13	3	3	10	198	21.76505951	5.414550781	1.022565947	10	4.931269763	1.025228321	10	6.168381329	1.01229669	10	4.681427409	1.019579082	10	8.932124464	0.996653319	10	10.95698919	0.968789096	10	5.159576604	Quantified
P39447	Tight junction protein ZO-1	Tjp1	FUNCTION: Tjp1, TjpP2, and Tjp3 are closely	Tight junction protein ZO-1	1.66	3	3	6	1745	194.6224866	6.639160156	1.0092641	6	2.259083732	0.985753366	6	3.005696016	1.029714108	6	3.168155734	1.026357231	6	2.005369336	1.021377851	6	6.541150052	0.979104576	6	4.155423926	Quantified

O08692	Neutrophilic granule protein	Ngp	FUNCTION: Acts as an inhibitor of cathepsin	Neutrophilic granule protein	22.75	4	4	4	167	19.31959368	5.312988281	1.015156934	4	7.315732966	1.027794788	4	7.328703281	0.948422926	4	11.46897967	0.997750853	4	7.973037662	1.025239297	4	1.413477204	1.035732167	4	3.265453871	Quantified
P70694	Estradiol 17 beta-dehydrogenase 5	Akr1c6	FUNCTION: Active toward androgens, estrog	Estradiol 17 beta-dehydrog	7.12	4	4	10	323	37.02396194	8.279785156	0.984855949	10	6.601319037	1.02156055	10	5.445833495	1.004980631	10	4.075531398	0.996970623	10	5.60498925	0.967058706	10	7.49480553	1.017487003	10	7.922396309	Quantified
Q9CQR2	40S ribosomal protein S21	Rps21		40S ribosomal protein S21	18.07	1	1	1	83	9.135554365	8.514160156																			Identified
Q62151	Advanced glycosylation end product-specific recepto	Ager	FUNCTION: Mediates interactions of advance	Advanced glycosylation en	8.46	2	2	3	402	42.62723323	6.074707031	0.918956909	3	7.023202559	0.907142675	3	12.88823722	1.032961117	3	17.24058864	0.988573148	3	26.590317	0.905615393	3	5.893386968	0.95820205	3	3.37042948	Quantified
Q99KD5	Protein unc-45 homolog A	Unc45a	FUNCTION: May act as co-chaperone for HS	Protein unc-45 homolog A	1.06	1	1	1	944	103.3818028	6.341308594	0.969788151	1		0.957087211	1		0.990427912	1		1.039853938	1		0.965952481	1		1.090731343	1		Quantified
P70296	Phosphatidylethanolamine-binding protein 1	Pebp1	FUNCTION: Binds ATP, opioids and phospha	Phosphatidylethanolamine-	7.49	1	1	1	187	20.81733033	5.401855469	0.85272141	1		0.950961745	1		0.719388931	1		1.244968017	1		1.247482717	1		1.149500846	1		Quantified
Q9JMA1	Ubiquitin carboxyl-terminal hydrolase 14	Usp14	FUNCTION: Proteasome-associated deubiqu	Ubiquitin carboxyl-terminal	10.14	5	5	5	493	55.96595047	5.236816406	1.019685691	5	5.56169649	1.007936331	5	2.716232601	1.012716425	5	8.215395782	0.953554846	5	2.863962727	0.976049807	5	7.614182655	1.01477434	5	3.438251936	Quantified
Q99PW4	EKC/KEOPS complex subunit Tp53rk	Tp53rk	FUNCTION: Component of the EKC/KEOPS	EKC/KEOPS complex subu	6.56	1	1	1	244	27.37629131	9.231933594																			Identified
P84096	Rho-related GTP-binding protein RhoG	Rhog	FUNCTION: Required for the formation of me	Rho-related GTP-binding pr	9.95	1	2	3	191	21.29493252	8.118652344	1.027711551	1		1.021050972	1		0.99910365	1		0.979797602	1		0.955144718	1		1.017580978	1		Quantified
P61082	NEDD8-conjugating enzyme Ubc12	Ube2m	FUNCTION: Accepts the ubiquitin-like protein	NEDD8-conjugating enzym	20.77	3	3	3	183	20.88664964	7.693847656	0.978101014	3	1.12388919	1.015470235	3	1.279195721	0.970965405	3	15.60569761	0.928702962	3	0.438955174	1.011721597	3	0.354498129	1.029603052	3	1.745328737	Quantified
Q99L45	Eukaryotic translation initiation factor 2 subunit 2	Eif2s2	FUNCTION: eIF-2 functions in the early steps	Eukaryotic translation initiat	7.55	2	2	2	331	38.06822293	5.795410156	1.019328544	2	2.164377407	1.026092023	2	2.750917141	1.044145807	2	8.255829454	0.982651588	2	5.399262216	0.941107213	2	2.646997227	0.971786671	2	3.812388655	Quantified
Q9ES52	Phosphatidylinositol 3,4,5-trisphosphate 5-phosphata	Inpp5d	FUNCTION: Phosphatidylinositol (PtdIns) pho	Phosphatidylinositol 3,4,5-t	2.6	3	3	3	1191	133.4583463	7.796386719	1.050952766	2	6.359983255	1.023059151	2	3.935620427	0.983174591	2	16.8878493	0.961642122	2	6.880553698	0.937981558	2	0.732337986	1.01708302	2	22.4365152	Quantified
P68500	Contactin-5	Cntn5	FUNCTION: Contactins mediate cell surface i	Contactin-5 OS=Mus musc	1.55	1	1	1	1098	120.6709935	6.609863281	0.985984042	1		0.918960148	1		1.174192282	1		0.936777168	1		0.919348897	1		1.040032451	1		Quantified
Q9Z0E6	Guanylate-binding protein 2	Gbp2	FUNCTION: Hydrolyzes GTP to GMP in 2 cor	Guanylate-binding protein 2	8.32	5	5	5	589	66.69722992	5.706542969	1.031111416	5	4.907910136	0.993191838	5	2.420743945	1.051465671	5	2.983609689	1.020323245	5	9.734953572	1.001237354	5	9.439991591	0.951100011	5	4.49124562	Quantified
D3Z7H8	Cartilage intermediate layer protein 2	Cilp2	FUNCTION: May play a role in cartilage scaff	Cartilage intermediate layer	1.03	1	1	1	1162	125.8545966	7.767089844	0.899474865	1		0.992569884	1		1.03037804	1		1.161161341	1		1.101237996	1		0.874215805	1		Quantified
P97326	Cadherin-6	Cdh6	FUNCTION: Cadherins are calcium-depender	Cadherin-6 OS=Mus muscu	2.66	2	2	2	790	88.30004165	4.995605469	1.102466588	2	18.62897409	1.032063457	2	4.037710242	1.049241034	2	5.900155455	0.891789824	2	6.652402129	1.01974006	2	9.57462854	0.90874989	2	2.479447057	Quantified
Q60817	Nascent polypeptide-associated complex subunit alp	Naca	FUNCTION: Prevents inappropriate targeting	Nascent polypeptide-assoc	6.98	1	1	2	215	23.3697129	4.563964844	1.026592565	2	5.019019998	0.987919595	2	2.966449883	0.91688763	2	1.823797595	1.021410456	2	1.082580895	1.124278731	2	0.994808977	1.003811839	2	0.221370128	Quantified
P25206	DNA replication licensing factor MCM3	Mcm3	FUNCTION: Acts as component of the MCM2	DNA replication licensing fa	1.48	1	1	2	812	91.48916752	5.554199219																			Identified
Q61398	Procollagen C-endopeptidase enhancer 1	Pcolce	FUNCTION: Binds to the C-terminal propeptic	Procollagen C-endopeptida	2.78	1	1	3	468	50.13610303	8.411621094	0.94189168	3	3.048700171	0.960358615	3	6.102551368	1.081508895	3	9.932121702	0.945721883	3	6.38828594	0.968101278	3	7.375270618	1.079767317	3	8.474844472	Quantified
P58281	Dynamin-like 120 kDa protein, mitochondrial	Opa1	FUNCTION: Dynamin-related GTPase that is	Dynamin-like 120 kDa prote	2.6	3	3	5	960	111.2695618	7.547363281	0.879861155	5	11.59714122	0.897847328	5	6.543159049	0.967653774	5	5.075255861	1.032409068	5	8.173389837	1.134829556	5	12.82156628	0.964876672	5	11.25516725	Quantified
Q99PV0	Pre-mRNA-processing-splicing factor 8	Prpf8	FUNCTION: Plays role in pre-mRNA splicing	Pre-mRNA-processing-splic	2.53	6	6	6	2335	273.4425669	8.836425781	0.996282279	6	1.88558287	0.991681343	6	1.139462358	0.9905637	6	3.484063293	1.034589689	6	3.339528374	0.987656661	6	5.919975028	1.023923872	6	2.418398164	Quantified
O09110	Dual specificity mitogen-activated protein kinase kina	Map2k3	FUNCTION: Dual specificity kinase. Is activat	Dual specificity mitogen-act	4.32	1	2	2	347	39.27105982	7.430175781	1.095992407	1		0.892261742	1		0.970743778	1		1.013157496	1		1.17806609	1		0.952785167	1		Quantified
P62270	40S ribosomal protein S18	Rps18	FUNCTION: Located at the top of the head of	40S ribosomal protein S18	13.16	2	2	2	152	17.70786103	10.98974609	0.961113287	2	1.114389537	0.97772047	2	1.55950401	1.036280091	2	2.553506443	1.058377708	2	4.671488586	0.921266927	2	13.74222062	1.029343107	2	3.182967569	Quantified
Q9DBH5	Vesicular integral-membrane protein VIP36	Lman2	FUNCTION: Plays a role as an intracellular le	Vesicular integral-membran	3.07	1	1	2	358	40.40421495	6.946777344	0.937381171	2	7.955963997	0.855770867	2	11.18781201	0.965258563	2	0.901134499	1.114244466	2	1.882207081	1.378128079	2	32.08207244	0.918099357	2	5.082689364	Quantified
Q91X52	L-xylulose reductase	Dcxr	FUNCTION: Catalyzes the NADPH-depender	L-xylulose reductase OS=M	9.84	1	2	4	244	25.72943735	7.312988281	0.935488155	2	5.389002585	1.059436849	2	2.385673328	1.073999735	2	19.43053389	0.931942669	2	10.89426031	0.931355388	2	21.0633017	1.030112294	2	0.888697101	Quantified
Q9DBE0	Cysteine sulfinic acid decarboxylase	Csad	FUNCTION: Catalyzes the decarboxylation of	Cysteine sulfinic acid decar	7.1	4	4	5	493	55.1093645	6.609863281	0.992210364	5	2.373710947	1.018412273	5	5.313400784	1.007527415	5	4.961336489	1.005454463	5	8.068618804	1.060530086	5	13.47251385	0.974891705	5	3.30048091	Quantified
O08788	Dynactin subunit 1	Dctn1	FUNCTION: Plays a key role in dynein-media	Dynactin subunit 1 OS=Mus	6.01	7	7	7	1281	141.5880679	5.896972656	1.024736781	7	8.206166811	1.028153905	7	3.453599081	0.975398698	7	10.42028392	0.944660076	7	5.091932307	0.965770759	7	14.08126602	0.979055834	7	8.750597695	Quantified
Q9QYC0	Alpha-adducin	Add1	FUNCTION: Membrane-cytoskeleton-associa	Alpha-adducin OS=Mus mu	7.89	6	6	8	735	80.59634048	5.896972656	0.966405355	8	11.78577155	0.958665958	8	12.04251915	0.984559701	8	5.582234918	1.013985668	8	7.471845066	0.987810494	8	7.093857909	1.021240032	8	4.427375554	Quantified
Q91WJ8	Far upstream element-binding protein 1	Fubp1	FUNCTION: Regulates MYC expression by b	Far upstream element-bindi	7.53	3	6	7	651	68.49702009	7.928222656	1.03995818	3	3.226795469	1.037957562	3	3.239493593	1.091595578	3	3.136946275	0.958635142	3	4.407380499	0.901499143	3	4.794145609	0.912913244	3	3.757559408	Quantified
Q9CYL5	Golgi-associated plant pathogenesis-related protein 1	Glipr2		Golgi-associated plant path	12.34	2	2	3	154	17.07951784	9.510253906	0.991138558	3	1.220373504	1.013113389	3	4.649354291	0.990731896	3	1.796438229	0.974472336	3	4.268399555	1.013966872	3	4.864248717	1.021299402	3	0.175898807	Quantified
Q3UCV8	Ubiquitin thioesterase otulin	Otulin	FUNCTION: Deubiquitinase that specifically r	Ubiquitin thioesterase otulin	4.55	2	2	2	352	40.29431939	5.681152344	0.985167687	2	6.941382785	0.999256718	2	5.173179793	0.986375467	2	2.812348541	0.966502685	2	0.588593078	0.99540252	2	2.206974217	1.083690343	2	13.0444413	Quantified
P63154	Crooked neck-like protein 1	Crnkl1	FUNCTION: Involved in pre-mRNA splicing p	Crooked neck-like protein 1	1.74	1	1	1	690	83.36344107	6.932128906	0.951372167	1		0.999705125	1		0.94820617	1		1.031177337	1		1.104634645	1		1.036432293	1		Quantified
Q91VR5	ATP-dependent RNA helicase DDX1	Ddx1	FUNCTION: Acts as an ATP-dependent RNA	ATP-dependent RNA helica	1.76	1	1	1	740	82.44777154	7.210449219	0.99256773	1		1.050460649	1		1.108101969	1		0.902534029	1		0.804821717	1		1.066965134	1		Quantified
Q01705	Neurogenic locus notch homolog protein 1	Notch1	FUNCTION: Functions as a receptor for mem	Neurogenic locus notch hor	0.79	2	2	4	2531	270.6541794	5.097167969	0.921642181	4	2.108755984	1.114759566	4	0.719213482	1.272290544	4	1.947000501	0.824244314	4	0.418771152	0.819556134	4	6.574016851	0.956072501	4	1.656337793	Quantified
P62082	40S ribosomal protein S7	Rps7	FUNCTION: Required for rRNA maturation. {E	40S ribosomal protein S7 C	4.64	1	1	1	194	22.11325902	10.09619141	1.053762241	1		1.029110954	1		1.119420517	1		0.965840832	1		0.92676412	1		0.875832946	1		Quantified
Q9D4H8	Cullin-2	Cul2	FUNCTION: Core component of multiple culli	Cullin-2 OS=Mus musculus	3.76	2	3	3	745	86.82077573	7.005371094	1.081894535	2	1.023708571	0.944057147	2	0.549683484	1.058058408	2	6.836138772	0.897705999	2	7.336629041	1.016184624	2	0.21056458	1.023680678	2	0.090980264	Quantified
P61967	AP-1 complex subunit sigma-1A	Ap1s1	FUNCTION: Subunit of clathrin-associated ad	AP-1 complex subunit sigm	15.82	2	2	3	158	18.72060462	5.731933594	1.026112982	3	2.252038367	0.938423107	3	6.313586459	0.958079338	3	8.516054594	0.991789623	3	6.55175805	1.023867579	3	1.592643014	1.053869036	3	8.711378285	Quantified
Q91VC4	Plasmalemma vesicle-associated protein	Plvap	FUNCTION: Endothelial cell-specific membra	Plasmalemma vesicle-asso	3.88	2	2	5	438	49.90209267	8.396972656	1.010595087	5	1.684631039	0.93803238	5	2.517591066	1.063000111	5	0.696742117	1.017916006	5	4.037564116	1.066998195	5	7.101851979	0.955657256	5	9.307603379	Quantified
Q8BRK9	Alpha-mannosidase 2x	Man2a2	FUNCTION: Catalyzes the first committed ste	Alpha-mannosidase 2x OS=	1.48	2	2	2	1152	130.5664967	6.873535156	0.899250537	2	10.01910751	0.967362709	2	7.347545293	1.017195874	2	3.751392505	1.016327192	2	0.611368849	0.988346331	2	1.641204987	1.118353659	2	20.8855323	Quantified
Q8K1I3	Secreted phosphoprotein 24	Spp2	FUNCTION: Could coordinate an aspect of bc	Secreted phosphoprotein 2	5.91	1	1	1	203	23.12125125	8.367675781	0.98520095	1		1.040208821	1		1.052195936	1		0.976015667	1		0.923396241	1		1.003291874	1		Quantified
P62754	40S ribosomal protein S6	Rps6	FUNCTION: May play an important role in con	40S ribosomal protein S6 C	14.86	3	3	3	249	28.66298801	10.84326172	0.993787809	3	0.005187454	1.017790542	3	0.035444599	1.039709419	3	0.595204248	0.97551239	3	3.964390712	0.986358251	3	4.554385816	1.013901588	3	0.170866195	Quantified
P08228	Superoxide dismutase [Cu-Zn]	Sod1	FUNCTION: Destroys radicals which are norn	Superoxide dismutase [Cu-	14.94	2	2	4	154	15.93280596	6.507324219	1.012667361	4	1.55772385	0.929721898	4	3.56018455	1.058150266	4	6.35561385	0.996448942	4	2.189320932	0.998852176	4	3.309108368	1.011469499	4	3.906219535	Quantified
Q3TEA8	Heterochromatin protein 1-binding protein 3	Hp1bp3	FUNCTION: Component of heterochromatin tl	Heterochromatin protein 1-b	8.66	5	5	8	554	60.8290885	9.700683594	0.922560128	8	6.168080186	0.934539276	8	3.774005241	0.925908053	8	10.79793961	1.098373555	8	4.494405117	1.085013863	8	6.953029969	1.034339826	8	1.515813468	Quantified
P24369	Peptidyl-prolyl cis-trans isomerase B	Ppib	FUNCTION: PPIase that catalyzes the cis-tra	Peptidyl-prolyl cis-trans iso	9.26	2	2	3	216	23.69856434	9.554199219	1.003395693	3	2.667224891	0.979766375	3	3.216613855	0.925229742	3	4.61326449	1.046161454	3	3.073439576	1.001558974	3	5.693322959	1.038809526	3	9.024104317	Quantified
Q9QVP4	Myosin regulatory light chain 2, atrial isoform	Myl7		Myosin regulatory light chai	17.14	2	2	2	175	19.43756777	4.805175781	1.106114642	2	13.49521901	1.121015605	2	3.354304966	0.783932839	2	21.43986119	1.009220241	2	0.219510375	1.094804373	2	1.072384419	0.942063085	2	0.24771091	Quantified
Q7TMY8	E3 ubiquitin-protein ligase HUWE1	Huwe1	FUNCTION: E3 ubiquitin-protein ligase which	E3 ubiquitin-protein ligase	0.53	2	2	3	4377	482.3324176	5.224121094	1.018980848	3	1.130513575	0.952370276	3	7.027463659	1.006371119	3	4.290370211	1.062423935	3	7.814100803	0.923911482	3	15.38852482	0.992996945	3	1.150566026	Quantified
P34960	Macrophage metalloelastase	Mmp12	FUNCTION: May be involved in tissue injury	Macrophage metalloelastas	2.54	1	1	1	473	54.93565766	9.012207031	0.99266488	1		1.044768397	1		1.003877111	1		0.945758737	1		1.038864072	1		1.00696043	1		Quantified
O88487	Cytoplasmic dynein 1 intermediate chain 2	Dync1i2	FUNCTION: Acts as one of several non-catal	Cytoplasmic dynein 1 interr	9.97	4	4	5	612	68.35166286	5.287597656	0.983661102	5	6.053331696	0.98929269	5	7.891174297	1.034653041	5	6.738248549	1.032709837	5	7.297468653	0.973953867	5	9.36087813	1.002075587	5	7.134395415	Quantified
P08030	Adenine phosphoribosyltransferase	Aprt	FUNCTION: Catalyzes a salvage reaction res	Adenine phosphoribosyltrar	5.56	1	1	1	180	19.71151693	6.785644531	0.977640484	1		1.066746039	1		0.954887075	1		1.023957374	1		1.015461483	1		0.988338041	1		Quantified
Q99K85	Phosphoserine aminotransferase	Psat1	FUNCTION: Catalyzes the reversible convers	Phosphoserine aminotransf	5.14	2	2	6	370	40.44674901	8.030761719	0.97327254	6	5.183229725	0.998405579	6	3.329424794	0.994095353	6	9.339372167	1.014772904	6	6.786936119	1.056194068	6	8.022023108	1.00339826	6	2.673232184	Quantified
Q3UPL0	Protein transport protein Sec31A	Sec31a	FUNCTION: Component of the coat protein co	Protein transport protein Se	2.6	3	3	4	1230	133.4855656	6.756347656	1.032234502	4	1.906475729	1.055365684	4	3.863485498	1.055350508	4	4.724061946	0.905305289	4	4.452896314	0.996332418	4	10.24408055	0.959960243	4	5.679176344	Quantified
P61079	Ubiquitin-conjugating enzyme E2 D3	Ube2d3	FUNCTION: Accepts ubiquitin from the E1 co	Ubiquitin-conjugating enzyr	7.48	1	1	1	147	16.67637578	7.796386719	0.975602955	1		0.996363338	1		1.015805295	1		1.027692472	1		1.054171487	1		0.971773817	1		Quantified
P14483	H-2 class II histocompatibility antigen, A beta chain	H2-Ab1		H-2 class II histocompatibili	19.62	4	4	7	265	30.1091928	7.239746094	0.975694083	7	2.842936968	0.97003508	7	1.80491164	1.018857172	7	11.07765913	1.029545194	7	5.09629014	1.114865085	7	6.7001211	0.991828909	7	7.38872944	Quantified
F6ZDS4	Nucleoprotein TPR	Tpr	FUNCTION: Component of the nuclear pore c	Nucleoprotein TPR OS=Mu	2.1	5	5	5	2431	273.8241095	5.033691406	0.927243187	5	3.106343003	0.946303485	5	1.640070294	0.952006289	5	5.755940508	1.120491998	5	5.602217903	1.044498764	5	7.55928883	1.04628229	5	0.165477627	Quantified
P50431	Serine hydroxymethyltransferase, cytosolic	Shmt1	FUNCTION: Interconversion of serine and gly	Serine hydroxymethyltransf	4.6	2	2	3	478	52.56747771	6.946777344	1.025467259	3	1.649525728	0.964660786	3	0.523171895	0.949613402	3	4.168470212	1.010761549	3	1.10290194	1.000518895	3	0.951869872	0.956312544	3	0.842755997	Quantified
P51174	Long-chain specific acyl-CoA dehydrogenase, mitoch	Acadl	FUNCTION: Long-chain specific acyl-CoA de	Long-chain specific acyl-Co	8.84	3	3	3	430	47.87749565	8.309082031	1.043555882	3	1.362063908	0.982275349	3	5.139650595	0.909013622	3	13.96729346	1.045408335	3	7.230751716	1.120950728	3	10.68732302	1.003627561	3	5.901380075	Quantified
Q9QVP9	Protein-tyrosine kinase 2-beta	Ptk2b	FUNCTION: Non-receptor protein-tyrosine kin	Protein-tyrosine kinase 2-be	1.88	2	2	4	1009	115.7200926	6.354003906	1.061531455	4	6.785777424	0.989270928	4	2.463323431	0.874480832	4	10.40875381	1.011839753	4	9.328906319	1.012041738	4	10.91270528	1.059104774	4	7.836467883	Quantified
Q9Z2W0	Aspartyl aminopeptidase	Dnpep	FUNCTION: Aminopeptidase with specificity t	Aspartyl aminopeptidase O	8.88	2	2	6	473	52.17380499	7.254394531	1.055383622	6	4.32168917	0.954292958	6	6.673826595	0.980934281	6	3.362851245	1.028624771	6	7.641148758	0.846986834	6	7.092700002	1.059830673	6	2.20519718	Quantified
Q61595	Kinectin	Ktn1	FUNCTION: Receptor for kinesin thus involve	Kinectin OS=Mus musculus	1.73	2	2	2	1327	152.4979757	5.858886719	0.937243888	2	2.411497077	0.97887161	2	7.116688758	0.970232955	2	5.439521176	1.107769929	2	3.396164494	1.084026584	2	14.33711581	0.977651917	2	2.967185651	Quantified
Q8C0E2	Vacuolar protein sorting-associated protein 26B	Vps26b	FUNCTION: Acts as component of the retrom	Vacuolar protein sorting-as	5.36	1	1	1	336	39.10017405	7.371582031	0.976292534	1		0.970468765	1		1.060263388	1		0.985885126	1		1.008652231	1		1.019108739	1		Quantified
Q62422	Osteoclast-stimulating factor 1	Ostf1	FUNCTION: Induces bone resorption, acting	Osteoclast-stimulating facto	3.72	1	1	1	215	23.76789955	5.681152344	0.995931165	1		1.050657659	1		1.013325685	1		0.994670932	1		1.049842175	1		0.929734663	1		Quantified
O55234	Proteasome subunit beta type-5	Psmb5	FUNCTION: Component of the 20S core prote	Proteasome subunit beta ty	15.91	3	4	8	264	28.51421329	7.020019531	0.987849409	6	1.498568405	0.971108938	6	4.994240812	1.001211824	6	10.1126647	0.973167048	6	3.474881042	1.011836992	6	9.116194822	1.017378473	6	1.91788091	Quantified
P01837	Immunoglobulin kappa constant	Igkc		Immunoglobulin kappa con	35.51	3	3	3	107	11.92665419	5.896972656	0.933668779	3	3.785765674	0.980656709	3	0.353712449	0.977704864	3	0.633197092	1.040856962	3	1.23538787	1.002220045	3	13.92781925	1.028690859	3	0.105324203	Quantified
P42567	Epidermal growth factor receptor substrate 15	Eps15	FUNCTION: Involved in cell growth regulation	Epidermal growth factor rec	5.35	3	3	5	897	98.41041775	4.602050781	1.062315449	5	3.069906947	0.962204442	5	14.6343864	1.033612348	5	10.75990162	0.958093437	5	2.407949788	1.000910702	5	10.13187866	0.986050628	5	5.585850029	Quantified
P62071	Ras-related protein R-Ras2	Rras2	FUNCTION: GTP-binding protein with GTPas	Ras-related protein R-Ras2	7.84	1	1	1	204	23.38472465	6.011230469	0.988269096	1		0.93519308	1		0.977968206	1		1.017626859	1		1.078567004	1		1.064814315	1		Quantified
Q9WV85	Nucleoside diphosphate kinase 3	Nme3	FUNCTION: Major role in the synthesis of nuc	Nucleoside diphosphate kin	14.2	3	3	3	169	19.08681972	6.683105469	1.022864331	3	10.92227747	0.939568171	3	1.348172359	1.015739194	3	0.583159159	1.031326676	3	2.754680346	1.053707543	3	8.212152426	1.053258833	3	5.631945805	Quantified
P54823	Probable ATP-dependent RNA helicase DDX6	Ddx6	FUNCTION: Essential for the formation of P-b	Probable ATP-dependent R	6.21	3	3	3	483	54.15726759	8.660644531	0.997385851	3	5.266173241	1.042341158	3	1.157073845	0.970684851	3	9.611338875	1.002831909	3	13.48928035	0.983104505	3	15.71756852	1.059601312	3	4.545487658	Quantified
P70460	Vasodilator-stimulated phosphoprotein	Vasp	FUNCTION: Ena/VASP proteins are actin-ass	Vasodilator-stimulated phos	5.87	2	2	2	375	39.64192918	8.528808594	1.036298818	2	6.853597277	1.036526474	2	1.885088414	0.989165278	2	5.650325795	1.000789087	2	0.067509182	0.956690789	2	4.977819189	0.978081944	2	0.280850304	Quantified
Q04519	Sphingomyelin phosphodiesterase	Smpd1	FUNCTION: Converts sphingomyelin to cerar	Sphingomyelin phosphodie	3.83	2	2	4	627	69.882161	7.195800781	1.03841063	4	2.561220544	1.029403873	4	2.009397155	1.022002099	4	3.468993086	0.929752158	4	0.878655564	0.972737936	4	2.068930377	1.000877285	4	4.383623693	Quantified
Q6ZWN5	40S ribosomal protein S9	Rps9		40S ribosomal protein S9 C	31.96	7	7	9	194	22.57755693	10.65283203	0.995357239	9	2.917493711	1.024879639	9	1.12692416	0.968578603	9	4.960506195	1.020399601	9	6.620085738	1.016775936	9	5.712999191	0.981359053	9	2.967521851	Quantified
P05132	cAMP-dependent protein kinase catalytic subunit alp	Prkaca	FUNCTION: Phosphorylates a large number c	cAMP-dependent protein ki	13.96	5	5	7	351	40.54497853	8.792480469	1.008935971	7	4.435490175	1.018592575	7	8.814688437	0.963517296	7	10.9884743	1.015863862	7	8.535703346	0.953457553	7	8.597111193	0.96516438	7	4.39837179	Quantified
Q01730	Ras suppressor protein 1	Rsu1	FUNCTION: Potentially plays a role in the Ra	Ras suppressor protein 1 O	6.5	2	2	3	277	31.53074293	8.880371094	0.972777994	3	1.69834856	0.964340085	3	3.717073749	1.052420835	3	3.19161528	0.960730815	3	0.457244963	1.073475995	3	4.924605931	1.008591311	3	1.085589028	Quantified
Q03249	Galactose-1-phosphate uridylyltransferase	Galt	FUNCTION: Plays an important role in galact	Galactose-1-phosphate uric	2.11	1	1	3	379	43.20450631	6.770996094	0.921084855	3	3.653171872	1.003585079	3	7.657749383	0.987281377	3	1.834851921	1.042020103	3	0.28136581	1.119242095	3	0.839370742	1.0104359	3	3.529422556	Quantified
Q8BYW1	Rho GTPase-activating protein 25	Arhgap25	FUNCTION: GTPase activator for the Rho-typ	Rho GTPase-activating pro	4.01	3	3	3	648	73.33701153	6.379394531	1.024606293	3	0.110810715	0.999307402	3	4.611404179	1.014274346	3	1.667583597	0.97218047	3	0.615042292	0.99068399	3	3.718361877	0.992043435	3	2.242445058	Quantified
P10518	Delta-aminolevulinic acid dehydratase	Alad	FUNCTION: Catalyzes an early step in the bi	Delta-aminolevulinic acid d	6.67	3	3	4	330	36.00030563	6.785644531	0.980069519	4	0.811031657	1.042281412	4	3.972755135	1.03327851	4	2.498526183	1.003729807	4	3.274392404	0.99185858	4	7.547452691	0.988424947	4	3.90674697	Quantified
P15532	Nucleoside diphosphate kinase A	Nme1	FUNCTION: Major role in the synthesis of nuc	Nucleoside diphosphate kin	36.84	2	5	7	152	17.19685384	7.371582031	0.932151361	3	8.467922469	1.053116043	3	0.721834147	0.995977605	3	0.079108122	0.987880187	3	3.729095969	1.091466929	3	8.109171093	0.934927741	3	2.909815395	Quantified
Q8BHN3	Neutral alpha-glucosidase AB	Ganab	FUNCTION: Catalytic subunit of glucosidase	Neutral alpha-glucosidase	4.87	5	5	7	944	106.8435122	6.062011719	0.98292788	7	2.101122837	1.01926621	7	2.141230136	0.987192172	7	1.479584945	1.008564238	7	5.18644303	1.026286786	7	7.092476059	0.994798001	7	4.264179767	Quantified
Q9QZH3	Peptidyl-prolyl cis-trans isomerase E	Ppie	FUNCTION: Involved in pre-mRNA splicing a	Peptidyl-prolyl cis-trans iso	2.99	1	1	2	301	33.4275182	5.604980469	1.001006806	2	1.396752633	0.9807162	2	0.98551811	0.972849849	2	2.933526414	1.058857048	2	1.578691565	0.92148079	2	4.549148942	1.058605795	2	2.671809261	Quantified
Q91X83	S-adenosylmethionine synthase isoform type-1	Mat1a	FUNCTION: Catalyzes the formation of S-ade	S-adenosylmethionine synt	11.87	4	4	5	396	43.48097159	5.820800781	1.011046684	5	6.244583643	1.074376048	5	5.983737751	0.99252321	5	6.975766964	0.97558247	5	4.290816213	0.909650126	5	6.606996402	1.025389782	5	9.701479292	Quantified
Q80UG5	Septin-9	Septin9	FUNCTION: Filament-forming cytoskeletal GT	Septin-9 OS=Mus musculus	6.52	4	4	9	583	65.53393073	8.895019531	0.982895258	9	1.393925459	1.011646615	9	5.037090405	1.00957026	9	5.281703021	0.990369736	9	6.30808436	0.987815746	9	6.73104656	1.028498866	9	4.841221736	Quantified
Q07076	Annexin A7	Anxa7	FUNCTION: Calcium/phospholipid-binding pr	Annexin A7 OS=Mus musc	4.1	2	2	2	463	49.89330265	6.176269531	0.958643525	2	2.378481596	1.000459657	2	5.76278707	1.067025253	2	14.4911779	1.022323395	2	8.642649381	1.003925261	2	14.8448586	0.950548101	2	3.774803983	Quantified
Q6P5F9	Exportin-1	Xpo1	FUNCTION: Mediates the nuclear export of ce	Exportin-1 OS=Mus muscul	6.91	6	6	9	1071	123.0130466	6.074707031	0.990426254	9	6.543342636	1.004896323	9	4.389082011	0.951297425	9	9.857148499	0.973857215	9	6.082358459	0.966491535	9	6.458794507	1.043961354	9	6.779722673	Quantified
Q80XU3	Nuclear ubiquitous casein and cyclin-dependent kina	Nucks1		Nuclear ubiquitous casein a	6.41	1	1	2	234	26.29762688	5.135253906	0.888037343	2	7.679389663	1.143851728	2	3.000038782	1.132067875	2	2.129740445	0.948120568	2	2.169647009	1.007731117	2	6.040090032	0.874718147	2	2.437006232	Quantified
Q9WTR5	Cadherin-13	Cdh13	FUNCTION: Cadherins are calcium-depender	Cadherin-13 OS=Mus musc	5.46	3	3	3	714	78.13696098	5.122558594	0.98215279	3	4.651586678	1.040750589	3	4.539164175	1.021800501	3	1.948146343	1.01187738	3	7.395796805	1.306782181	3	6.921110954	0.956070023	3	7.846309232	Quantified
P21855	B-cell differentiation antigen CD72	Cd72	FUNCTION: Plays a role in B-cell proliferation	B-cell differentiation antiger	2.82	1	1	1	354	40.32194536	6.430175781																			Identified
P27601	Guanine nucleotide-binding protein subunit alpha-13	Gna13	FUNCTION: Guanine nucleotide-binding prote	Guanine nucleotide-binding	7.96	2	3	4	377	44.02677425	8.206542969	1.032921454	3	9.40735679	0.998435597	3	0.840530097	1.060907703	3	3.169766579	0.974992653	3	6.694686199	1.035277019	3	7.47162598	0.975469407	3	0.186537867	Quantified
Q91VH2	Sorting nexin-9	Snx9	FUNCTION: Involved in endocytosis and intra	Sorting nexin-9 OS=Mus m	2.02	1	1	1	595	66.50357255	5.516113281	1.04279484	1		1.023093334	1		1.096333238	1		0.977536917	1		0.899460648	1		0.924677955	1		Quantified
Q922J3	CAP-Gly domain-containing linker protein 1	Clip1	FUNCTION: Binds to the plus end of microtut	CAP-Gly domain-containing	1.73	2	2	2	1391	155.7180787	5.236816406	1.041549899	2	5.512463492	0.953118894	2	5.582945095	0.867837582	2	26.37514373	1.020022256	2	11.85511025	1.108214044	2	9.936406063	1.072112231	2	6.261921907	Quantified
A6H5Y3	Methionine synthase	Mtr	FUNCTION: Catalyzes the transfer of a methy	Methionine synthase OS=M	1.6	2	2	2	1253	138.9806406	5.566894531	1.057888917	2	2.111188396	0.871905583	2	22.48105989	1.019636715	2	9.594772164	1.024933207	2	0.192592094	1.103283681	2	22.73815565	0.970138435	2	7.361237505	Quantified
P24527	Leukotriene A-4 hydrolase	Lta4h	FUNCTION: Epoxide hydrolase that catalyzes	Leukotriene A-4 hydrolase	6.71	4	4	5	611	69.00714516	6.417480469	1.014853485	5	2.832097001	1.000787979	5	3.41105032	1.013504267	5	5.822804654	1.000145048	5	3.377629874	1.049739499	5	6.792117783	0.958008546	5	1.070649112	Quantified
O88545	COP9 signalosome complex subunit 6	Cops6	FUNCTION: Component of the COP9 signalo	COP9 signalosome comple	2.47	1	1	2	324	35.8570025	5.731933594	1.013056049	2	4.595242433	1.032389363	2	4.371984185	1.039145381	2	2.153439966	0.983073792	2	1.233370101	0.970602654	2	0.258188614	0.960925795	2	1.338134103	Quantified
Q9D1Q6	Endoplasmic reticulum resident protein 44	Erp44	FUNCTION: Mediates thiol-dependent retenti	Endoplasmic reticulum resi	9.11	2	3	3	406	46.82330689	5.274902344	1.017654389	2	5.905594498	0.961130854	2	1.756006867	0.950124757	2	14.56984541	1.0482915	2	3.391469886	1.145823872	2	0.270530574	0.957608438	2	8.210898267	Quantified
Q6PDQ2	Chromodomain-helicase-DNA-binding protein 4	Chd4	FUNCTION: Component of the histone deace	Chromodomain-helicase-DN	0.84	2	2	2	1915	217.6137991	5.808105469	1.081549973	2	10.73243338	0.994848049	2	1.293616249	0.979129826	2	15.551182	0.97949115	2	3.119707068	0.931280019	2	19.67796353	1.008945136	2	14.33999827	Quantified
Q9QUR8	Semaphorin-7A	Sema7a	FUNCTION: Plays an important role in integri	Semaphorin-7A OS=Mus m	1.96	1	1	3	664	74.94614623	7.737792969	0.967576698	3	4.111540275	1.035112742	3	7.071798121	0.999495367	3	0.708259752	0.933649353	3	3.183186803	0.977932156	3	3.445641216	1.040855528	3	5.140598003	Quantified
Q61249	Immunoglobulin-binding protein 1	Igbp1	FUNCTION: Associated to surface IgM-recep	Immunoglobulin-binding pro	3.24	1	1	1	340	38.94658791	6.176269531																			Identified
Q6PE01	U5 small nuclear ribonucleoprotein 40 kDa protein	Snrnp40	FUNCTION: Required for pre-mRNA splicing	U5 small nuclear ribonucle	4.47	1	1	1	358	39.25070946	8.104003906	0.987454428	1		0.955132613	1		0.979364093	1		1.135787788	1		1.050137766	1		0.938459546	1		Quantified
Q61081	Hsp90 co-chaperone Cdc37	Cdc37	FUNCTION: Co-chaperone that binds to num	Hsp90 co-chaperone Cdc37	16.89	4	4	9	379	44.56494914	5.338378906	1.022704952	9	4.853072518	1.000651909	9	2.581328195	1.002973711	9	5.407052956	1.002123542	9	2.323486683	0.980485707	9	2.913857112	0.987070189	9	3.283924083	Quantified
Q9DAW9	Calponin-3	Cnn3	FUNCTION: Thin filament-associated protein	Calponin-3 OS=Mus muscu	8.79	2	3	4	330	36.40583966	5.719238281	0.54223902	3	3.167902375	0.597972491	3	22.28007967	0.644372107	3	27.44255147	0.578049219	3	1.718877955	0.840056738	3	12.57671559	2.881734554	3	12.81761608	Quantified
Q8BGB5	LIM domain-containing protein 2	Limd2	FUNCTION: Acts as an activator of the protei	LIM domain-containing prot	14.84	2	2	3	128	14.22785742	9.041503906	1.020593194	3	2.661770315	1.0314522	3	2.499939064	1.086792742	3	9.082795695	0.984793818	3	1.986811878	0.903398289	3	1.450272536	0.885426706	3	0.849084412	Quantified
Q9QZS0	Collagen alpha-3(IV) chain	Col4a3	FUNCTION: Type IV collagen is the major str	Collagen alpha-3(IV) chain	3.54	4	4	7	1669	161.6242658	9.480957031	1.002596917	6	2.788648942	0.979189431	7	3.750033836	0.956198484	6	2.345792441	1.057748333	7	8.502196973	1.020102475	6	3.085816481	0.996699014	6	6.215623691	Quantified
Q9D1H7	Golgi to ER traffic protein 4 homolog	Get4	FUNCTION: As part of a cytosolic protein qua	Golgi to ER traffic protein 4	4.59	1	1	1	327	36.50205845	5.414550781	1.019161675	1		1.023350931	1		1.039627804	1		0.928753734	1		0.984603425	1		1.012252477	1		Quantified
O55226	Chondroadherin	Chad	FUNCTION: Promotes attachment of chondro	Chondroadherin OS=Mus m	11.17	4	4	4	358	40.32323491	9.393066406	0.970510628	3	0.607033264	1.025517713	3	2.336278898	0.94761452	3	0.191847939	1.049345488	3	0.121527199	0.999269059	3	2.229219399	1.024567873	3	5.182681123	Quantified
Q9JKR6	Hypoxia up-regulated protein 1	Hyou1	FUNCTION: Has a pivotal role in cytoprotecti	Hypoxia up-regulated prote	6.11	4	4	4	999	111.1121768	5.186035156	1.054979611	4	1.984293315	1.026154254	4	7.562475023	0.933628705	4	7.528240432	0.9328604	4	1.852562668	0.97932248	4	7.145639034	1.084750555	4	4.119418893	Quantified
O88587	Catechol O-methyltransferase	Comt	FUNCTION: Catalyzes the O-methylation, anc	Catechol O-methyltransfera	2.64	1	1	3	265	29.46729486	5.833496094	0.978927121	3	0.569869515	1.00560978	3	0.993607687	0.982202021	3	0.404213562	1.030062376	3	3.397455715	0.975673866	3	0.231494886	1.028671	3	1.368066582	Quantified
Q9JM99	Proteoglycan 4	Prg4	FUNCTION: Plays a role in boundary lubricati	Proteoglycan 4 OS=Mus mu	0.76	1	1	3	1054	115.9244092	8.411621094	1.009434753	3	6.54330791	1.029572965	3	1.904030747	0.972055446	3	0.186474437	1.036776207	3	3.666531443	1.034734494	3	8.572081898	0.95338549	3	1.37903654	Quantified
O35295	Transcriptional activator protein Pur-beta	Purb	FUNCTION: Has capacity to bind repeated el	Transcriptional activator pro	4.94	1	2	3	324	33.88077052	5.427246094	0.962718445	2	1.544565802	1.010483345	2	2.081794253	1.01692943	2	4.55188043	1.028386323	2	4.384840024	1.019706388	2	2.842774955	0.987023295	2	1.488430141	Quantified
Q6P4T2	U5 small nuclear ribonucleoprotein 200 kDa helicase	Snrnp200	FUNCTION: Plays role in pre-mRNA splicing	U5 small nuclear ribonucle	4.87	8	8	8	2136	244.3916145	6.062011719	0.978347762	8	6.557380535	0.945008947	8	6.900945285	0.979902709	8	3.680692528	1.069849774	8	2.489753491	1.079515041	8	7.781979797	1.011336042	8	1.331469734	Quantified
Q3THK3	General transcription factor IIF subunit 1	Gtf2f1	FUNCTION: TFIIF is a general transcription ir	General transcription factor	2.56	1	1	1	508	57.20649933	7.005371094	0.94306574	1		1.068959328	1		1.053727913	1		1.019619805	1		0.88287229	1		0.993591501	1		Quantified
Q9Z1F9	SUMO-activating enzyme subunit 2	Uba2	FUNCTION: The heterodimer acts as an E1 li	SUMO-activating enzyme s	7.99	5	5	7	638	70.52469059	5.236816406	1.019614846	7	3.418329745	1.082677817	7	4.161039131	1.094088604	7	7.59335299	0.890687283	7	3.422412052	0.944275327	7	5.236378487	0.921382158	7	6.101209688	Quantified
Q61147	Ceruloplasmin	Cp	FUNCTION: Ceruloplasmin is a blue, copper-	Ceruloplasmin OS=Mus mu	2.92	2	2	2	1061	121.074203	5.846191406	1.001772782	2	11.79714801	1.067595354	2	3.897344955	1.058861178	2	13.05190931	0.917715128	2	3.894487076	0.953222978	2	0.687176409	0.980593015	2	4.817482079	Quantified
Q8BRF7	Sec1 family domain-containing protein 1	Scfd1	FUNCTION: Plays a role in SNARE-pin asser	Sec1 family domain-contair	11.89	7	7	8	639	72.27709061	6.379394531	0.988337995	8	7.161874767	0.978927992	8	5.04243908	1.000462425	8	8.279246712	1.047145637	8	4.835936263	1.036631328	8	8.994672964	1.031947324	8	10.28300667	Quantified
Q8CEF9	Transmembrane protein 132C	Tmem132c		Transmembrane protein 13	1.91	1	1	1	1099	121.3020398	6.049316406	1.027514318	1		1.067514699	1		0.938908619	1		0.94046315	1		1.057804439	1		1.013448011	1		Quantified
Q8VC30	Triokinase/FMN cyclase	Tkfc	FUNCTION: Catalyzes both the phosphorylat	Triokinase/FMN cyclase OS	1.73	1	1	2	578	59.65335094	6.917480469	0.986540175	2	3.589298423	0.989412581	2	2.997108508	0.940206582	2	4.830927897	1.024263239	2	2.507225333	1.011798773	2	5.717711981	1.07436402	2	17.06002207	Quantified
P05202	Aspartate aminotransferase, mitochondrial	Got2	FUNCTION: Catalyzes the irreversible transa	Aspartate aminotransferase	4.88	2	2	21	430	47.38121061	8.997558594	0.962480874	21	2.820220737	1.051511947	21	3.00620539	1.012880526	21	3.245242177	0.929876878	21	4.431886406	1.036002543	21	4.341140842	1.018666286	21	3.067925395	Quantified
B2RXS4	Plexin-B2	Plxnb2	FUNCTION: Cell surface receptor for SEMA4	Plexin-B2 OS=Mus muscul	0.54	1	1	1	1842	206.0992494	5.871582031	1.00659674	1		1.006043034	1		0.983557127	1		0.966346476	1		1.039267519	1		1.037060855	1		Quantified
P35293	Ras-related protein Rab-18	Rab18	FUNCTION: Required for the localization of Z	Ras-related protein Rab-18	17.96	3	3	3	206	23.02069197	5.363769531	0.956280611	3	3.752299287	0.960917348	3	1.117094204	0.993196055	3	4.605498047	0.979902186	3	5.762727253	1.012368692	3	8.892529515	1.124041245	3	6.163485581	Quantified
Q9D154	Leukocyte elastase inhibitor A	Serpinb1a	FUNCTION: Neutrophil serine protease inhibi	Leukocyte elastase inhibito	8.18	4	4	4	379	42.54776404	6.214355469	1.02858394	4	3.104624919	1.029615027	4	2.279808997	1.058492358	4	1.786259269	0.915638979	4	4.572483529	0.978092549	4	4.473008658	1.000228721	4	4.399662108	Quantified
Q61490	CD166 antigen	Alcam	FUNCTION: Cell adhesion molecule that med	CD166 antigen OS=Mus mu	3.77	3	3	5	583	65.05098426	6.150878906	0.974554282	5	11.54190616	1.08360481	5	4.521335455	0.947976603	5	8.190510424	0.976744654	5	10.94047652	1.085306359	5	25.3331216	1.045118789	5	11.7849447	Quantified
P63328	Serine/threonine-protein phosphatase 2B catalytic su	Ppp3ca	FUNCTION: Calcium-dependent, calmodulin-	Serine/threonine-protein ph	9.98	5	5	10	521	58.60628009	5.858886719	0.978748411	10	5.061923844	1.047164694	10	7.880545678	1.062344238	10	4.045566998	0.947281914	10	7.044267192	0.961421341	10	2.528614182	0.993243704	10	7.724005296	Quantified

P07310	Creatine kinase M-type	Ckm	FUNCTION: Reversibly catalyzes the transfer	Creatine kinase M-type OS	10.76	4	4	4	381	43.01780101	7.063964844	0.974142387	4	5.860507012	1.005629246	4	0.505527237	0.965375563	4	2.790452186	1.015510642	4	4.044295631	1.041717538	4	15.21393679	1.044516182	4	4.837088905	Quantified
Q6DIB5	Multiple epidermal growth factor-like domains protein	Megf10	FUNCTION: Membrane receptor involved in p	Multiple epidermal growth fa	1.83	2	2	2	1147	122.8875231	7.049316406	0.982570231	2	4.073027393	1.033845385	2	1.219492896	1.075708158	2	4.015062973	0.928969063	2	2.38302087	0.964706517	2	7.296035611	1.007817591	2	0.164703494	Quantified
P25444	40S ribosomal protein S2	Rps2		40S ribosomal protein S2 C	15.36	5	5	5	293	31.21166248	10.24267578	0.980526442	5	4.733747748	1.005967118	5	1.705990247	0.966290744	5	5.072906005	1.024131203	5	6.250063202	1.019120011	5	0.714097663	1.031184492	5	1.256241215	Quantified
Q61792	LIM and SH3 domain protein 1	Lasp1	FUNCTION: Plays an important role in the reg	LIM and SH3 domain protei	18.25	5	5	8	263	29.97531204	7.049316406	1.004382474	8	3.073106228	1.013374924	8	3.755112181	0.937821662	8	4.068411268	1.021409709	8	9.639166704	0.995187204	8	4.236371436	0.965158661	8	5.120197149	Quantified
Q8R059	UDP-glucose 4-epimerase	Gale	FUNCTION: Catalyzes two distinct but analog	UDP-glucose 4-epimerase (	6.92	2	2	3	347	38.20025433	6.741699219	0.974129732	3	2.62871935	0.95398086	3	0.478511606	1.02237372	3	0.788956405	1.141339869	3	1.751655297	1.050225352	3	3.338909185	0.996707576	3	5.727750687	Quantified
Q9WVJ2	26S proteasome non-ATPase regulatory subunit 13	Psmd13	FUNCTION: Component of the 26S proteasor	26S proteasome non-ATPa	6.91	3	3	5	376	42.78208872	5.706542969	0.98309655	5	8.148419179	1.03468114	5	3.240977445	1.030452991	5	3.84268394	0.961902725	5	6.026080622	0.867663505	5	2.570297108	1.023475592	5	2.342346597	Quantified
Q9Z0N1	Eukaryotic translation initiation factor 2 subunit 3, X-li	Eif2s3x	FUNCTION: As a subunit of eukaryotic initiati	Eukaryotic translation initiat	9.96	4	4	5	472	51.03319441	8.396972656	1.011699879	4	2.724306993	1.008056909	4	0.648445239	0.998993023	4	3.118719354	1.018546937	4	7.452279127	1.022902667	4	3.322185187	0.940054823	4	1.092431186	Quantified
P60882	Multiple epidermal growth factor-like domains protein	Megf8	FUNCTION: Acts as a negative regulator of h	Multiple epidermal growth fa	0.43	1	1	1	2789	297.2920444	6.800292969	1.093112287	1		1.055837388	1		0.937580515	1		0.988921248	1		0.945893344	1		0.977182495	1		Quantified
Q9QYB5	Gamma-adducin	Add3	FUNCTION: Membrane-cytoskeleton-associa	Gamma-adducin OS=Mus r	1.42	1	1	1	706	78.72769892	5.947753906	1.023375314	1		1.085281164	1		1.160303876	1		0.915704268	1		0.744102342	1		0.959971858	1		Quantified
P99029	Peroxiredoxin-5, mitochondrial	Prdx5	FUNCTION: Thiol-specific peroxidase that ca	Peroxiredoxin-5, mitochond	3.81	1	1	1	210	21.88354166	8.851074219	0.913513529	1		0.94755492	1		0.960246808	1		1.056769785	1		1.043973644	1		1.13017144	1		Quantified
Q9JM76	Actin-related protein 2/3 complex subunit 3	Arpc3	FUNCTION: Component of the Arp2/3 comple	Actin-related protein 2/3 co	17.42	3	3	3	178	20.51132571	8.587402344	1.002563513	3	6.481893889	0.871734988	3	2.24565446	1.103772429	3	4.688251655	1.089439585	3	4.361695242	1.099215765	3	0.915514277	0.889496422	3	12.32286054	Quantified
Q62443	Neuronal pentraxin-1	Nptx1	FUNCTION: May be involved in mediating up	Neuronal pentraxin-1 OS=M	9.26	4	4	6	432	47.08778395	6.442871094	0.990447455	6	4.564888914	0.995712426	6	3.099875452	0.990999137	6	5.66290377	1.050828248	6	6.844655034	0.980592277	6	8.319098115	0.996112677	6	7.718221922	Quantified
Q61508	Extracellular matrix protein 1	Ecm1	FUNCTION: Involved in endochondral bone f	Extracellular matrix protein	2.86	2	2	3	559	62.79185788	6.800292969	1.009200981	3	1.398849346	0.934052265	3	0.765160001	0.942606681	3	2.59305899	1.078318372	3	3.15891775	0.97084114	3	1.362199087	1.071057085	3	13.54682273	Quantified
Q9Z2D0	Myotubularin-related protein 9	Mtmr9	FUNCTION: Acts as an adapter for myotubula	Myotubularin-related protei	2.02	1	1	1	545	62.86789683	6.624511719	1.105217534	1		0.826129136	1		1.083062239	1		1.052653684	1		1.00751926	1		0.95052982	1		Quantified
Q8JZQ5	Amiloride-sensitive amine oxidase [copper-containing	Aoc1	FUNCTION: Catalyzes the degradation of cor	Amiloride-sensitive amine c	1.2	1	1	2	751	85.37001544	6.639160156	1.03434998	2	1.109975824	0.982246955	2	6.138427753	0.966700796	2	6.796591969	1.035206724	2	1.301266044	0.991044605	2	0.979300617	1.010066209	2	0.056327055	Quantified
Q9CPY7	Cytosol aminopeptidase	Lap3	FUNCTION: Presumably involved in the proc	Cytosol aminopeptidase OS	2.31	1	1	1	519	56.10575085	7.723144531	0.973785789	1		1.082180702	1		0.9802797	1		0.934543406	1		1.155319341	1		0.95364725	1		Quantified
Q60737	Casein kinase II subunit alpha	Csnk2a1	FUNCTION: Catalytic subunit of a constitutive	Casein kinase II subunit alp	3.84	1	1	1	391	45.10483429	7.737792969	1.050354507	1		1.00235516	1		1.023689464	1		0.972739334	1		0.937204618	1		1.004605753	1		Quantified
O08583	THO complex subunit 4	Alyref	FUNCTION: Export adapter involved in nucle	THO complex subunit 4 OS	2.75	1	1	2	255	26.92360855	11.15087891	1.0851159	2	4.571609493	0.892333466	2	9.086626961	1.01985573	2	5.792177727	1.037442854	2	5.59708674	1.050377163	2	15.09211123	0.951309495	2	8.442543272	Quantified
Q9CQD1	Ras-related protein Rab-5A	Rab5a	FUNCTION: Small GTPase which cycles betw	Ras-related protein Rab-5A	8.84	1	1	1	215	23.58379769	8.147949219	1.0108862	1		0.971121281	1		0.906809019	1		1.070760466	1		0.986231866	1		1.083774338	1		Quantified
Q9QUJ7	Long-chain-fatty-acid--CoA ligase 4	Acsl4	FUNCTION: Catalyzes the conversion of long	Long-chain-fatty-acid--CoA	3.66	2	2	2	711	79.02565778	8.279785156	1.011946593	2	5.514630388	1.039072954	2	0.709470288	0.916452282	2	4.71010029	0.990949638	2	1.806454045	1.036155343	2	3.441269817	1.046574665	2	1.345562737	Quantified
Q8C052	Microtubule-associated protein 1S	Map1s	FUNCTION: Microtubule-associated protein tl	Microtubule-associated pro	1.34	1	1	1	973	102.8753052	7.020019531	0.944048569	1		1.050828034	1		1.08355757	1		0.899024497	1		0.998046485	1		1.032563651	1		Quantified
P62281	40S ribosomal protein S11	Rps11		40S ribosomal protein S11	14.56	3	3	3	158	18.41899389	10.30126953	0.977478507	3	8.221252667	1.036984368	3	0.914848207	1.004658848	3	3.112933125	1.016242352	3	2.504187066	0.995560772	3	0.501964575	1.040477868	3	5.209408794	Quantified
Q99M28	RNA-binding protein with serine-rich domain 1	Rnps1	FUNCTION: Part of pre- and post-splicing mu	RNA-binding protein with se	4.92	1	1	1	305	34.18770302	11.83935547	1.056639277	1		0.997312562	1		0.993337727	1		1.014911585	1		0.941539356	1		0.992492678	1		Quantified
Q9DCW4	Electron transfer flavoprotein subunit beta	Etfb	FUNCTION: Heterodimeric electron transfer fl	Electron transfer flavoprotei	3.92	1	1	1	255	27.6059856	8.104003906	0.859690562	1		0.825807334	1		0.834569867	1		0.956368216	1		0.993944679	1		1.596522184	1		Quantified
Q9JLI8	Squamous cell carcinoma antigen recognized by T-c	Sart3	FUNCTION: U6 snRNP-binding protein that fu	Squamous cell carcinoma a	4.37	3	4	7	962	109.5498318	5.236816406	1.047256215	2	5.961536487	1.039256499	2	2.638013198	1.02469538	2	4.633885309	0.939370219	2	2.900506692	0.918641226	2	10.19057841	1.007090943	2	6.502159364	Quantified
P35585	AP-1 complex subunit mu-1	Ap1m1	FUNCTION: Subunit of clathrin-associated ac	AP-1 complex subunit mu-1	11.35	4	4	7	423	48.51203251	7.298339844	1.037143683	7	3.440192561	1.043146449	7	1.795750423	1.005081354	7	4.319703092	0.941105987	7	2.589848349	0.938833044	7	3.862490445	1.071197655	7	7.880836626	Quantified
Q9JM14	5'(3')-deoxyribonucleotidase, cytosolic type	Nt5c	FUNCTION: Dephosphorylates the 5' and 2'(3	5'(3')-deoxyribonucleotidase	15.5	2	2	2	200	23.06177439	5.490722656	1.009299012	2	2.137018871	0.993591491	2	3.056609447	0.972704118	2	0.349510364	1.05806625	2	5.103362065	1.003068545	2	6.516266151	0.986869416	2	0.096791124	Quantified
P26262	Plasma kallikrein	Klkb1	FUNCTION: The enzyme cleaves Lys-Arg an	Plasma kallikrein OS=Mus	4.23	3	3	3	638	71.33591095	8.016113281	0.952281223	3	2.647114936	1.065866526	3	2.089248871	1.277369412	3	2.369298801	0.862377179	3	5.383928688	0.894047905	3	1.484829955	0.91805523	3	9.783370853	Quantified
Q60631	Growth factor receptor-bound protein 2	Grb2	FUNCTION: Adapter protein that provides a c	Growth factor receptor-bour	13.82	3	3	3	217	25.22240707	6.315917969	0.995892927	3	6.186029218	1.030656731	3	6.296443192	0.965188437	3	7.582715905	1.014183891	3	2.353029533	1.051235266	3	1.350102454	1.013366525	3	0.060047392	Quantified
O54988	STE20-like serine/threonine-protein kinase	Slk	FUNCTION: Mediates apoptosis and actin str	STE20-like serine/threonine	8.19	9	10	12	1233	141.3703359	5.135253906	1.010439915	11	6.380863895	1.060967995	11	6.002092489	1.018274952	11	19.98681706	0.992859184	11	7.140199822	0.971740131	11	13.29276732	0.934314493	11	18.9877998	Quantified
P47955	60S acidic ribosomal protein P1	Rplp1	FUNCTION: Plays an important role in the elo	60S acidic ribosomal protei	14.91	1	1	1	114	11.46767752	4.322753906	1.056081223	1		0.778831009	1		1.169563686	1		0.989444995	1		1.297995715	1		0.845514884	1		Quantified
P19096	Fatty acid synthase	Fasn	FUNCTION: Fatty acid synthetase catalyzes t	Fatty acid synthase OS=Mu	1.8	5	5	6	2504	272.256792	6.580566406	1.004269878	6	2.991765227	1.007121382	6	4.467673904	1.030022217	6	5.556530251	1.004216023	6	3.544067431	0.978136607	6	9.009029148	0.96715852	6	3.372269912	Quantified
P51885	Lumican	Lum		Lumican OS=Mus musculus	14.79	4	4	6	338	38.24061284	6.430175781	0.965335779	6	0.889159167	0.937990842	6	2.271650494	1.011086701	6	5.282336263	1.042674197	6	1.382347893	1.036330052	6	13.58506462	1.010226987	6	4.381500625	Quantified
Q9CRA5	Golgi phosphoprotein 3	Golph3	FUNCTION: Phosphatidylinositol-4-phosphate	Golgi phosphoprotein 3 OS	5.03	1	1	1	298	33.73148796	6.442871094	0.949708666	1		0.975151817	1		1.048095994	1		0.980452091	1		1.021860307	1		1.053140611	1		Quantified
Q8BYA0	Tubulin-specific chaperone D	Tbcd	FUNCTION: Tubulin-folding protein implicated	Tubulin-specific chaperone	1.84	1	1	1	1196	133.2357572	6.507324219	1.109556529	1		0.782334406	1		0.989304514	1		1.084547145	1		1.185791649	1		0.961370536	1		Quantified
Q8CAY6	Acetyl-CoA acetyltransferase, cytosolic	Acat2	FUNCTION: Involved in the biosynthetic path	Acetyl-CoA acetyltransferas	2.02	1	1	2	397	41.27120593	7.503417969	0.965537466	2	1.490210068	1.002938698	2	3.78410237	0.957999543	2	3.208732794	1.045307326	2	0.328034742	1.042884977	2	6.067137362	1.028177284	2	3.251288953	Quantified
P97927	Laminin subunit alpha-4	Lama4	FUNCTION: Binding to cells via a high affinity	Laminin subunit alpha-4 OS	2.37	4	4	4	1816	201.6921198	6.214355469	0.947832514	4	8.988732528	0.958364419	4	1.718667602	1.017157053	4	13.06926054	1.033752628	4	9.272479924	1.040652886	4	10.41837362	0.955438119	4	4.782490191	Quantified
P17095	High mobility group protein HMG-I/HMG-Y	Hmga1	FUNCTION: HMG-I/Y bind preferentially to the	High mobility group protein	7.48	1	1	1	107	11.60714234	10.31591797	0.94853461	1		0.725287327	1		1.186593622	1		1.451841708	1		0.964373602	1		0.725818135	1		Quantified
Q6ZWX6	Eukaryotic translation initiation factor 2 subunit 1	Eif2s1	FUNCTION: Functions in the early steps of pr	Eukaryotic translation initiat	9.52	3	3	3	315	36.08543629	5.084472656	1.054219554	3	5.393378518	0.961970441	3	7.403127874	1.031812154	3	10.95799524	1.089150264	3	2.382566002	1.037649582	3	6.179912075	0.973961313	3	0.542049427	Quantified
Q8CFI7	DNA-directed RNA polymerase II subunit RPB2	Polr2b	FUNCTION: DNA-dependent RNA polymeras	DNA-directed RNA polymer	1.45	2	2	3	1174	133.8247078	6.873535156	0.997916913	3	6.291078332	0.960076884	3	2.70495508	0.989164645	3	5.301592148	0.952984802	3	0.620082524	1.039274569	3	0.878559102	1.099843266	3	9.290299987	Quantified
P25911	Tyrosine-protein kinase Lyn	Lyn	FUNCTION: Non-receptor tyrosine-protein kin	Tyrosine-protein kinase Lyr	10.16	3	6	8	512	58.77472637	7.151855469	0.961415333	5	4.265415812	0.98504623	5	2.54448334	1.004929592	5	5.252082972	1.030357445	5	0.29453472	0.981182037	5	7.347328851	1.029906964	5	6.780949162	Quantified
P70441	Na(+)/H(+) exchange regulatory cofactor NHE-RF1	Slc9a3r1	FUNCTION: Scaffold protein that connects pl	Na(+)/H(+) exchange regula	3.94	1	1	5	355	38.57654766	5.896972656	0.991904234	5	3.356032498	0.893985779	5	6.314038665	0.940098869	5	10.48195152	1.130966741	5	2.256068927	1.113369293	5	11.19101426	1.017509997	5	2.356659864	Quantified
Q9R118	Serine protease HTRA1	Htra1	FUNCTION: Serine protease with a variety of	Serine protease HTRA1 OS	4.58	2	2	4	480	51.1816288	7.649902344	1.00586903	4	5.670757561	1.039306452	4	5.000622086	1.057723743	4	1.598824051	0.953815294	4	7.52465647	0.935755584	4	4.582301917	0.957117608	4	3.579170202	Quantified
Q99MI6	GTPase IMAP family member 3	Gimap3	FUNCTION: During thymocyte development,	GTPase IMAP family memb	4.98	1	2	3	301	34.15745815	7.093261719																			Identified
Q3V3R4	Integrin alpha-1	Itga1	FUNCTION: Integrin alpha-1/beta-1 is a recep	Integrin alpha-1 OS=Mus m	0.76	1	1	1	1179	130.7269852	6.201660156	0.955586598	1		0.981882658	1		1.080078686	1		0.966798238	1		0.952374043	1		1.058878484	1		Quantified
Q61087	Laminin subunit beta-3	Lamb3	FUNCTION: Binding to cells via a high affinity	Laminin subunit beta-3 OS=	1.37	1	1	1	1168	128.8162592	7.474121094	1.023254215	1		1.016419992	1		0.808488884	1		1.153687737	1		0.98953	1		1.04608072	1		Quantified
Q9JIY5	Serine protease HTRA2, mitochondrial	Htra2	FUNCTION: Serine protease that shows prote	Serine protease HTRA2, mi	2.84	1	1	2	458	49.31805631	9.598144531	1.027356424	2	2.106023955	0.85453848	2	21.1614559	1.027145044	2	6.885481998	1.011973884	2	3.611877114	1.175353836	2	25.23341801	0.980426379	2	12.11702632	Quantified
Q8K2Q9	Shootin-1	Shtn1	FUNCTION: Involved in the generation of inte	Shootin-1 OS=Mus muscul	4.44	3	3	3	631	71.29852935	5.439941406	0.961852941	3	0.066849824	0.97036356	3	10.8952304	0.995782203	3	14.34163927	1.006135902	3	9.559703517	1.154258825	3	2.379365792	1.026182863	3	0.158866298	Quantified
P61255	60S ribosomal protein L26	Rpl26	FUNCTION: Component of the large ribosom	60S ribosomal protein L26 (	5.52	1	1	1	145	17.24753305	10.55029297	1.028188586	1		0.979480408	1		1.080329771	1		1.041987852	1		0.857609425	1		0.96565053	1		Quantified
Q8K1M6	Dynamin-1-like protein	Dnm1l	FUNCTION: Functions in mitochondrial and p	Dynamin-1-like protein OS=	5.66	5	5	6	742	82.6064984	7.049316406	1.000352199	6	4.162105342	1.010204163	6	5.19556663	0.986242207	6	5.434811315	0.998146152	6	5.13209468	1.019520761	6	7.888126596	1.005161542	6	4.372852516	Quantified
O54754	Aldehyde oxidase 1	Aox1	FUNCTION: Oxidase with broad substrate sp	Aldehyde oxidase 1 OS=Mu	3.53	4	4	4	1333	146.5833803	7.020019531	1.014259989	4	2.709979495	1.043440407	4	2.760601839	0.928602427	4	6.779713383	1.05907037	4	5.457566403	1.028372865	4	2.732134686	0.986794934	4	6.293754402	Quantified
P42225	Signal transducer and activator of transcription 1	Stat1	FUNCTION: Signal transducer and transcripti	Signal transducer and activ	6.14	4	4	4	749	87.14211412	5.579589844	1.030905633	4	0.960873445	1.002137961	4	1.753569357	0.987853059	4	9.266073816	0.984481556	4	9.918524357	0.912363566	4	7.737288247	0.991268729	4	1.234806402	Quantified
Q8R4Y4	Stabilin-1	Stab1	FUNCTION: Acts as a scavenger receptor for	Stabilin-1 OS=Mus muscul	0.7	2	2	2	2571	276.0738739	6.653808594	0.93493373	2	12.53858689	1.030274306	2	7.814286928	1.170875559	2	32.3639555	0.912354406	2	19.85520039	0.921832928	2	23.59872719	0.945726169	2	8.19434943	Quantified
Q8C196	Carbamoyl-phosphate synthase [ammonia], mitochor	Cps1	FUNCTION: Involved in the urea cycle of ure	Carbamoyl-phosphate synth	1.27	2	2	2	1500	164.513672	6.917480469	1.006567233	2	4.863837885	0.943183579	2	4.855668462	0.914143781	2	2.128156515	1.184301797	2	16.54343772	0.977381416	2	7.758143403	0.987319133	2	12.27815576	Quantified
Q60854	Serpin B6	Serpinb6	FUNCTION: Inhibitor of cathepsin G, kallikrei	Serpin B6 OS=Mus muscul	2.38	1	1	1	378	42.57117354	5.744628906	0.955478608	1		0.996715567	1		0.98428439	1		1.111176259	1		1.009108473	1		0.970098098	1		Quantified
Q99LX0	Parkinson disease protein 7 homolog	Park7	FUNCTION: Multifunctional protein with contr	Parkinson disease protein 7	16.93	3	3	3	189	20.00838202	6.770996094	1.0090094	3	0.541233704	1.004621138	3	0.345426025	0.98229152	3	3.18663177	1.018077418	3	3.091402694	0.966297865	3	0.211566177	1.012560273	3	0.907114821	Quantified
P47911	60S ribosomal protein L6	Rpl6	FUNCTION: Component of the large ribosom	60S ribosomal protein L6 O	8.11	3	3	3	296	33.48892678	10.69677734	0.983502	3	8.175002249	1.046755309	3	0.374341604	1.127778181	3	2.315519744	0.948989924	3	3.020513064	0.902436675	3	0.877837739	1.020178919	3	2.70895879	Quantified
Q8R0W0	Epiplakin	Eppk1	FUNCTION: Cytoskeletal linker protein that c	Epiplakin OS=Mus muscul	2.61	1	1	1	6548	724.1826371	6.074707031	1.053653926	1		0.953438546	1		0.894200522	1		1.032618615	1		1.058296764	1		1.068658724	1		Quantified
Q9Z0X1	Apoptosis-inducing factor 1, mitochondrial	Aifm1	FUNCTION: Functions both as NADH oxidore	Apoptosis-inducing factor 1	7.03	4	4	4	612	66.72384694	9.173339844	0.956549935	4	13.42728164	0.926885897	4	10.38401804	0.976737971	4	14.01393659	0.98930234	4	5.639757194	1.140400262	4	13.06821081	0.982283312	4	5.302613862	Quantified
Q64739	Collagen alpha-2(XI) chain	Col11a2	FUNCTION: May play an important role in fib	Collagen alpha-2(XI) chain	0.52	1	1	1	1736	171.4306766	6.480957031	0.853654448	1		1.065874503	1		1.333258951	1		0.818609851	1		1.17414964	1		0.802278246	1		Quantified
Q99LF4	RNA-splicing ligase RtcB homolog	Rtcb	FUNCTION: Catalytic subunit of the tRNA-spl	RNA-splicing ligase RtcB h	12.48	6	6	8	505	55.21386972	7.225097656	1.033006288	8	5.557381586	1.035366071	8	3.626184729	0.945780467	8	7.065984471	0.982245346	8	8.05527196	1.039858203	8	17.0485196	1.007296273	8	9.366137516	Quantified
Q6IFX2	Keratin, type I cytoskeletal 42	Krt42		Keratin, type I cytoskeletal	6.19	1	3	3	452	50.10189421	5.160644531	1.16184666	1		1.257028238	1		0.815790918	1		0.944118906	1		0.764630582	1		0.976582866	1		Quantified
Q9QZB7	Actin-related protein 10	Actr10		Actin-related protein 10 OS	3.6	1	1	1	417	46.17826656	7.605957031	1.014663485	1		0.881091647	1		0.98585061	1		0.863040641	1		1.03548139	1		1.271840084	1		Quantified
Q64433	10 kDa heat shock protein, mitochondrial	Hspe1	FUNCTION: Co-chaperonin implicated in mitc	10 kDa heat shock protein,	7.84	1	1	1	102	10.95586631	8.353027344	0.962729022	1		0.947955473	1		1.06324254	1		1.051564359	1		1.030479033	1		0.974540153	1		Quantified
P26928	Hepatocyte growth factor-like protein	Mst1		Hepatocyte growth factor-lik	2.37	2	2	2	716	80.56686259	7.562011719	0.975810313	2	2.242734877	1.019166498	2	6.232874663	0.982289877	2	9.161588072	0.961596404	2	9.270527797	1.074445322	2	16.32623589	1.030823652	2	0.059976651	Quantified
A2ASQ1	Agrin	Agrn	FUNCTION: [Isoform 1]: heparan sulfate basa	Agrin OS=Mus musculus O	3.13	6	6	7	1950	207.4032748	6.315917969	0.950941187	7	2.026290426	1.033256961	7	2.752753833	1.029792588	7	10.64022786	0.99355579	7	7.424570629	1.033382228	7	14.8744967	1.004190278	7	3.038382934	Quantified
Q3THE2	Myosin regulatory light chain 12B	Myl12b	FUNCTION: Myosin regulatory subunit that pl	Myosin regulatory light chai	18.6	3	3	4	172	19.76651689	4.843261719	0.955622626	4	4.330731841	0.94518606	4	3.376831241	0.974375734	4	2.423334782	1.122328789	4	10.86154221	1.074128459	4	5.217218819	1.010601775	4	1.243423355	Quantified
P80316	T-complex protein 1 subunit epsilon	Cct5	FUNCTION: Component of the chaperonin-co	T-complex protein 1 subuni	9.8	7	7	8	541	59.58592691	6.023925781	1.017274213	8	4.905695309	0.993911283	8	4.975206894	1.030387021	8	3.587652226	0.984585606	8	8.992260452	0.950068416	8	11.16601201	0.976171476	8	5.133900516	Quantified
P28798	Progranulin	Grn	FUNCTION: Secreted protein that acts as a k	Progranulin OS=Mus muscu	2.21	1	1	1	589	63.4130741	6.800292969	0.825365448	1		1.099301228	1		1.286561573	1		0.822851294	1		1.040560625	1		0.924016955	1		Quantified
P70122	Ribosome maturation protein SBDS	Sbds	FUNCTION: Required for the assembly of ma	Ribosome maturation protei	6.8	2	2	3	250	28.76225933	8.763183594	1.020788004	3	1.314981715	1.010466344	3	0.794690103	0.987740184	3	2.604805897	1.015483619	3	0.06062514	0.931661093	3	0.691656974	1.01532783	3	0.33361339	Quantified
Q8CHR6	Dihydropyrimidine dehydrogenase [NADP(+)]	Dpyd	FUNCTION: Involved in pyrimidine base degr	Dihydropyrimidine dehydrog	4	4	4	4	1025	111.1813071	7.269042969	1.021890734	4	3.292183922	0.995525399	4	5.563368594	0.967583141	4	13.38009386	0.994266015	4	0.817642435	1.010975542	4	14.57973876	0.967015102	4	4.870116124	Quantified
Q8R1F1	Protein Niban 2	Niban2	FUNCTION: May play a role in apoptosis sup	Protein Niban 2 OS=Mus m	4.54	4	4	4	749	84.76496994	5.935058594	0.984540759	4	3.930736404	1.009302289	4	1.368016751	0.95706439	4	4.685200927	1.031632131	4	1.085608897	0.954962596	4	4.039128773	1.004256005	4	5.60130697	Quantified
Q9CPR4	60S ribosomal protein L17	Rpl17	FUNCTION: Component of the large ribosom	60S ribosomal protein L17 (	21.74	3	4	5	184	21.40938569	10.18408203	0.96410119	4	1.058293839	1.01049449	4	3.308365879	0.987585668	4	4.8437029	1.04351348	4	4.692059873	1.001653764	4	4.604061754	0.994180489	4	4.098252638	Quantified
P97352	Protein S100-A13	S100a13	FUNCTION: Plays a role in the export of prote	Protein S100-A13 OS=Mus	19.39	2	2	2	98	11.15090151	6.125488281	0.98495254	2	0.69597402	0.858209273	2	2.446939225	1.153236496	2	1.269207656	0.911015631	2	7.753530633	1.369222635	2	1.900680509	0.886266785	2	2.676719167	Quantified
O70133	ATP-dependent RNA helicase A	Dhx9	FUNCTION: Multifunctional ATP-dependent n	ATP-dependent RNA helica	4.42	6	6	7	1380	149.3805264	6.829589844	1.021930747	7	1.910048459	0.94925329	7	4.60520937	0.968417972	7	3.2536017	0.967526668	7	3.860183632	1.021120218	7	5.891164765	1.060460931	7	6.478944012	Quantified
Q8BWT1	3-ketoacyl-CoA thiolase, mitochondrial	Acaa2	FUNCTION: In the production of energy from	3-ketoacyl-CoA thiolase, mi	3.53	1	1	2	397	41.8034394	8.089355469	1.029960888	2	0.259225215	1.005933236	2	4.610829573	0.838832411	2	12.90850654	1.116588235	2	12.6376443	1.096079601	2	16.40514338	0.976441808	2	7.627328028	Quantified
Q8BZM1	Glomulin	Glmn	FUNCTION: Regulatory component of cullin-F	Glomulin OS=Mus musculu	1.68	1	1	1	596	67.7122711	5.059082031	0.753226031	1		1.913407743	1		0.992930385	1		0.692824567	1		0.788672142	1		0.718530159	1		Quantified
Q99PL5	Ribosome-binding protein 1	Rrbp1	FUNCTION: Acts as a ribosome receptor and	Ribosome-binding protein 1	28.04	16	16	20	1605	172.7758447	9.334472656	0.989467575	20	4.106234607	1.025740143	20	14.22631015	0.930838638	20	6.941975957	1.071702058	20	3.406036599	1.045901507	20	5.307471596	0.906340205	20	12.636947	Quantified
Q9CXY6	Interleukin enhancer-binding factor 2	Ilf2	FUNCTION: Appears to function predominant	Interleukin enhancer-bindin	10	3	3	5	390	43.03518913	5.262207031	0.983000534	5	5.617733162	1.002046036	5	5.012302052	1.033630739	5	10.57648084	0.959126067	5	4.275565636	0.991890258	5	6.802239598	1.02543574	5	2.109477768	Quantified
P00375	Dihydrofolate reductase	Dhfr	FUNCTION: Key enzyme in folate metabolism	Dihydrofolate reductase OS	3.74	1	1	1	187	21.59223914	8.528808594	1.005783257	1		1.034507238	1		1.042381089	1		0.982004004	1		0.91061637	1		1.000849386	1		Quantified
Q8CIE6	Coatomer subunit alpha	Copa	FUNCTION: The coatomer is a cytosolic prot	Coatomer subunit alpha OS	2.61	3	3	3	1224	138.3438611	7.649902344	1.063949163	3	4.415301412	0.998703382	3	3.29441404	1.021950539	3	6.278720543	1.003607591	3	0.812607002	0.902074668	3	7.948709384	0.986023348	3	5.298071838	Quantified
P97807	Fumarate hydratase, mitochondrial	Fh	FUNCTION: Catalyzes the reversible stereos	Fumarate hydratase, mitoch	14	6	6	8	507	54.32207593	9.041503906	0.973299427	8	8.152286815	1.047213388	8	5.223517988	1.018335982	8	4.82410352	0.928121328	8	6.549913196	1.031311853	8	4.269135529	1.012262285	8	5.833601273	Quantified
Q9D379	Epoxide hydrolase 1	Ephx1	FUNCTION: Biotransformation enzyme that c	Epoxide hydrolase 1 OS=M	6.81	3	3	3	455	52.54299978	8.353027344	0.959511916	3	1.766832028	0.943702168	3	5.533843315	0.994843237	3	7.727561651	1.083105432	3	2.889899391	1.028821035	3	0.551438496	1.068450029	3	3.339830992	Quantified
Q6P9R2	Serine/threonine-protein kinase OSR1	Oxsr1	FUNCTION: Phosphorylates RELL1, RELL2,	Serine/threonine-protein kir	3.61	1	1	1	527	58.17710984	6.430175781	0.997529381	1		0.883008268	1		0.983731699	1		1.108028947	1		0.976827575	1		1.075064874	1		Quantified
P22892	AP-1 complex subunit gamma-1	Ap1g1	FUNCTION: Subunit of clathrin-associated ac	AP-1 complex subunit gam	3.65	3	3	4	822	91.29213758	6.800292969	1.048254783	4	7.686400541	1.02980258	4	0.628982723	1.012349648	4	0.838392781	0.979915371	4	9.5738274	0.877407409	4	3.250190499	0.994317128	4	2.774274497	Quantified
O70251	Elongation factor 1-beta	Eef1b	FUNCTION: EF-1-beta and EF-1-delta stimula	Elongation factor 1-beta OS	5.78	1	1	1	225	24.6782467	4.690917969	1.056241849	1		1.052199578	1		1.046812947	1		0.918069404	1		0.923564274	1		0.98111349	1		Quantified
P53026	60S ribosomal protein L10a	Rpl10a	FUNCTION: Component of the large ribosom	60S ribosomal protein L10a	10.14	2	2	3	217	24.90060022	9.979003906	0.986827905	3	3.288396575	1.00614959	3	0.670277285	0.973916149	3	0.663644706	0.997522754	3	0.655052649	1.080240169	3	2.306902941	0.994919068	3	6.540135795	Quantified
Q3U0S6	Ras-interacting protein 1	Rasip1	FUNCTION: Required for the proper formation	Ras-interacting protein 1 OS	1.14	1	1	1	961	103.4943657	7.825683594	1.145459155	1		0.896954563	1		0.936185044	1		1.023580289	1		1.119735507	1		0.959738951	1		Quantified
P35329	B-cell receptor CD22	Cd22	FUNCTION: Mediates B-cell B-cell interaction	B-cell receptor CD22 OS=M	3.25	2	2	3	862	96.52123214	7.928222656	1.002799521	3	26.29521552	0.958480587	3	14.24583561	1.058928436	3	13.58810404	0.975652591	3	16.13159947	0.934199521	3	12.81364908	0.921805273	3	4.056058988	Quantified
P63242	Eukaryotic translation initiation factor 5A-1	Eif5a	FUNCTION: mRNA-binding protein involved i	Eukaryotic translation initiat	27.92	4	4	7	154	16.82140541	5.236816406	0.993970661	7	4.380125672	0.948494661	7	2.996431111	0.975023813	7	10.70152121	1.058665744	7	13.86368869	0.958862695	7	1.446357873	1.01286932	7	3.787178983	Quantified
P61290	Proteasome activator complex subunit 3	Psme3	FUNCTION: Subunit of the 11S REG-gamma	Proteasome activator comp	5.91	1	1	1	254	29.48756039	5.947753906	1.036232732	1		0.952830179	1		0.956724359	1		0.982744899	1		1.078965499	1		1.054845385	1		Quantified
Q9EQS9	Immunoglobulin superfamily DCC subclass member	Igdcc4		Immunoglobulin superfamily	2.32	3	3	3	1252	134.6814294	6.176269531	0.974551089	3	1.721916606	0.994390255	3	5.255626914	1.011708092	3	14.21706192	1.064832383	3	10.85640584	1.030183374	3	2.283436579	0.90198118	3	0.689600862	Quantified
Q6A0D4	Raftlin	Rftn1	FUNCTION: Involved in protein trafficking via	Raftlin OS=Mus musculus (	3.61	2	2	2	554	61.49922633	7.679199219	0.984522822	2	3.784311114	0.999465231	2	3.197682685	1.019745148	2	2.496563028	1.056502312	2	6.051847174	1.001913047	2	4.319517505	0.952935319	2	8.873479064	Quantified
P62748	Hippocalcin-like protein 1	Hpcal1	FUNCTION: May be involved in the calcium-c	Hippocalcin-like protein 1 C	4.15	1	1	1	193	22.32400726	5.503417969	0.9727988	1		1.021442721	1		1.056183671	1		0.979061821	1		0.920829136	1		1.03085728	1		Quantified
Q8BWY3	Eukaryotic peptide chain release factor subunit 1	Etf1	FUNCTION: Directs the termination of nascer	Eukaryotic peptide chain re	17.62	7	7	7	437	49.00015206	5.706542969	1.034637356	7	1.964977011	1.000031648	7	2.069446957	1.028506375	7	4.872092458	0.990223042	7	3.067061796	0.918914102	7	7.25671047	1.035317303	7	3.940071746	Quantified
P26645	Myristoylated alanine-rich C-kinase substrate	Marcks	FUNCTION: MARCKS is the most prominent	Myristoylated alanine-rich C	6.15	1	1	1	309	29.64381765	4.335449219	0.975206438	1		0.853934699	1		1.237755625	1		1.09154228	1		1.101287271	1		0.783306422	1		Quantified
Q8R4V5	Oncoprotein-induced transcript 3 protein	Oit3	FUNCTION: May be involved in hepatocellula	Oncoprotein-induced transc	4.76	3	3	4	546	60.27663584	5.389160156	0.972262654	4	2.915583137	1.015966778	4	3.086132956	1.070470886	4	3.561193255	0.990152962	4	4.625133512	1.002370809	4	9.968806923	0.956824239	4	3.083327872	Quantified
P47791	Glutathione reductase, mitochondrial	Gsr	FUNCTION: Maintains high levels of reduced	Glutathione reductase, mitc	2.2	1	1	1	500	53.62853395	7.986816406	0.988636291	1		1.077666861	1		0.982686965	1		1.017800389	1		0.899295821	1		1.009273263	1		Quantified
Q9CQJ6	Density-regulated protein	Denr	FUNCTION: May be involved in the translatio	Density-regulated protein O	4.04	1	1	2	198	22.15213331	5.300292969	0.928890198	2	2.867219075	1.000888918	2	3.198115398	1.003919328	2	7.145702152	0.972374272	2	4.516387479	1.087763388	2	2.970926864	1.061620837	2	7.588282274	Quantified
Q8BPU7	Engulfment and cell motility protein 1	Elmo1	FUNCTION: Involved in cytoskeletal rearrang	Engulfment and cell motility	2.48	2	2	2	727	83.88165273	6.277832031	1.014179745	2	2.235510196	1.067014778	2	0.571795617	0.964464554	2	6.644721141	0.941444577	2	7.579282238	1.004866984	2	8.957819009	1.024824344	2	7.300407617	Quantified
Q6ZPJ3	(E3-independent) E2 ubiquitin-conjugating enzyme U	Ube2o	FUNCTION: E2/E3 hybrid ubiquitin-protein lig	(E3-independent) E2 ubiqui	0.78	1	1	1	1288	140.745572	5.059082031	1.127817717	1		0.99000688	1		0.924349484	1		1.003796428	1		1.000307619	1		0.980838203	1		Quantified
Q9EQG9	Ceramide transfer protein	Cert1	FUNCTION: Shelters ceramides and diacylgly	Ceramide transfer protein C	5.45	3	3	5	624	71.06681124	5.439941406	0.991251873	5	5.503446444	1.023676617	5	1.478709567	0.998544974	5	4.281205183	1.036172733	5	2.922555678	1.082096195	5	24.7323498	0.978163347	5	1.934239688	Quantified
O88998	Noelin	Olfm1	FUNCTION: Contributes to the regulation of a	Noelin OS=Mus musculus (	3.71	2	2	3	485	55.36250314	6.946777344	0.992465091	3	0.521582726	1.009782955	3	6.916599194	1.020310348	3	1.718431983	0.93823053	3	0.06384019	1.031157861	3	1.594919475	0.999733179	3	1.667717963	Quantified
P11911	B-cell antigen receptor complex-associated protein al	Cd79a	FUNCTION: Required in cooperation with CD	B-cell antigen receptor com	17.73	2	2	2	220	24.56732233	5.109863281	0.987291699	2	3.015359426	0.893151581	2	10.97517239	0.828609477	2	16.77208591	1.161035376	2	1.007028272	1.230270422	2	27.6687201	1.021792602	2	8.095774769	Quantified
O88322	Nidogen-2	Nid2	FUNCTION: Cell adhesion glycoprotein. Migh	Nidogen-2 OS=Mus muscul	3.28	4	4	5	1403	153.8160599	5.376464844	1.025081427	5	9.643379995	1.019107397	5	5.131703315	1.003928486	5	5.368497968	1.022593822	5	5.641323627	1.058047809	5	9.688110525	0.943313835	5	2.929478138	Quantified
Q9D0I9	Arginine--tRNA ligase, cytoplasmic	Rars1	FUNCTION: Forms part of a macromolecular	Arginine--tRNA ligase, cyto	5.3	4	4	6	660	75.62514988	7.547363281	0.994259864	6	5.834884097	0.993516904	6	2.146143911	0.991490712	6	2.826819593	1.019457945	6	3.667764179	1.009259188	6	7.220539774	1.015200829	6	6.343253936	Quantified
Q569Z6	Thyroid hormone receptor-associated protein 3	Thrap3	FUNCTION: Involved in pre-mRNA splicing. F	Thyroid hormone receptor-a	5.57	5	5	6	951	108.1137005	10.16943359	0.970608194	6	4.704714375	0.981556185	6	7.470583093	1.037648043	6	5.837443808	1.057032365	6	2.012876144	0.974170694	6	5.74346319	0.963244473	6	5.665096322	Quantified
Q62010	Oviduct-specific glycoprotein	Ovgp1	FUNCTION: Binds to oocyte zona pellucida ir	Oviduct-specific glycoprotei	0.97	1	1	9	721	78.75802982	9.187988281	1.011357896	3	5.786072229	1.030251563	3	0.889974828	0.994687735	3	3.657278761	0.971342227	3	2.327071618	0.947557902	3	5.311727658	0.983907649	3	7.170030521	Quantified
Q8BWY9	Protein CIP2A	Cip2a	FUNCTION: Promotes anchorage-independe	Protein CIP2A OS=Mus mu	2.32	2	3	4	907	101.9783093	6.277832031	0.983594921	1		1.034274062	1		1.015123394	1		0.973728942	1		1.064751082	1		0.971086503	1		Quantified
Q8VH51	RNA-binding protein 39	Rbm39	FUNCTION: RNA-binding protein that acts as	RNA-binding protein 39 OS	3.21	1	1	1	530	59.36970784	10.09619141	0.910664983	1		0.866520684	1		1.058282712	1		0.930727729	1		1.143628944	1		1.179870334	1		Quantified
Q8CJG0	Protein argonaute-2	Ago2	FUNCTION: Required for RNA-mediated gen	Protein argonaute-2 OS=Mu	4.07	3	3	4	860	97.24192641	9.187988281	0.961415522	4	1.141675709	1.053991947	4	5.835156232	1.028337402	4	4.205232655	0.931189264	4	3.502706277	1.02160573	4	5.184963387	0.954090252	4	0.758128971	Quantified
Q9JHK4	Geranylgeranyl transferase type-2 subunit alpha	Rabggta	FUNCTION: Catalyzes the transfer of a geran	Geranylgeranyl transferase	4.76	3	3	4	567	64.94808029	5.795410156	0.991347504	4	3.045758837	1.087506091	4	5.334922725	0.968229461	4	2.108761924	0.970269266	4	5.214381379	0.860834981	4	9.153491276	1.062720232	4	7.691277679	Quantified
Q91VI7	Ribonuclease inhibitor	Rnh1	FUNCTION: Ribonuclease inhibitor which inh	Ribonuclease inhibitor OS=	7.68	3	3	5	456	49.78411499	4.779785156	0.998261595	5	4.289490631	0.962916222	5	3.767535516	1.053411653	5	1.331869916	1.023932766	5	5.825255718	0.974314707	5	7.102126261	0.955074341	5	6.161760971	Quantified
O08808	Protein diaphanous homolog 1	Diaph1	FUNCTION: Actin nucleation and elongation f	Protein diaphanous homolo	5.82	8	8	11	1255	139.2547353	5.566894531	0.994402086	11	1.965102315	1.097596128	11	3.947718822	0.992493578	11	7.656681803	0.958618542	11	5.102415139	0.977927868	11	6.385187026	0.967340091	11	4.491703676	Quantified
Q62348	Translin	Tsn	FUNCTION: DNA-binding protein that specific	Translin OS=Mus musculus	6.58	1	1	2	228	26.18475322	6.442871094	0.970937634	2	1.13587886	1.097402087	2	3.332397496	1.033083483	2	5.375300401	0.852503481	2	4.291030551	1.078168092	2	5.245596242	1.007675623	2	3.675048283	Quantified
Q9QXX0	Protein jagged-1	Jag1	FUNCTION: Ligand for multiple Notch recepto	Protein jagged-1 OS=Mus r	1.07	2	2	3	1218	134.0739589	6.252441406	0.936865155	3	4.31968118	1.029269193	3	3.799519902	1.089514026	3	5.867446311	0.95122261	3	8.880734008	1.08282946	3	5.293913268	0.972371772	3	3.014170635	Quantified
Q9ES89	Exostosin-like 2	Extl2	FUNCTION: Glycosyltransferase required for	Exostosin-like 2 OS=Mus m	2.12	1	1	1	330	37.36633796	9.041503906	0.982297108	1		0.91386181	1		0.904618346	1		0.985535946	1		1.017206447	1		1.247776809	1		Quantified
Q8VCT3	Aminopeptidase B	Rnpep	FUNCTION: Exopeptidase which selectively r	Aminopeptidase B OS=Mus	5.38	3	3	4	650	72.37011915	5.351074219	1.033630364	4	1.183962175	1.004668576	4	2.708863759	1.014584829	4	5.646418237	0.994570258	4	0.583086295	0.938761294	4	0.70376092	0.992614199	4	0.378188462	Quantified
Q8BME9	Cerebellin-4	Cbln4	FUNCTION: Acts as a synaptic organizer in s	Cerebellin-4 OS=Mus musc	6.06	1	1	1	198	21.59534233	8.733886719	0.991227736	1		1.0484463	1		0.926435478	1		1.000772727	1		1.068525422	1		1.018541156	1		Quantified

Q61553	Fascin	Fscn1	FUNCTION: Actin-binding protein that contair	Fascin OS=Mus musculus (	3.45	2	2	5	493	54.47393547	6.888183594	0.986961533	5	9.36019815	1.022205504	5	1.847898092	0.974872818	5	2.251249132	0.944915114	5	9.216015946	1.083945862	5	7.061595637	1.031694701	5	2.913375319	Quantified
P62702	40S ribosomal protein S4, X isoform	Rps4x		40S ribosomal protein S4, X	14.07	3	3	4	263	29.57905527	10.15478516	0.990742711	4	1.397168937	1.013604884	4	2.64080301	1.015170558	4	1.868895907	1.02871859	4	2.378882004	0.966288559	4	8.683609868	0.99226128	4	3.556249919	Quantified
P70236	Dual specificity mitogen-activated protein kinase kina	Map2k6	FUNCTION: Dual specificity protein kinase wl	Dual specificity mitogen-act	5.99	1	2	2	334	37.40821466	7.386230469	0.991048719	1		0.974603118	1		1.058621273	1		1.053944071	1		1.015463384	1		0.927405914	1		Quantified
Q9Z130	Heterogeneous nuclear ribonucleoprotein D-like	Hnrnpdl	FUNCTION: Acts as a transcriptional regulato	Heterogeneous nuclear ribo	2.33	1	1	2	301	33.53809266	7.312988281	1.130367691	2	0.490896754	1.17136568	2	3.671991378	1.283068518	2	3.420430079	0.77974398	2	2.310259175	0.784808321	2	2.657833226	0.726856797	2	1.265684914	Quantified
Q8R5A3	Amyloid beta A4 precursor protein-binding family B m	Apbb1ip	FUNCTION: Appears to function in the signal	Amyloid beta A4 precursor	1.34	1	1	1	670	74.2723501	5.351074219	1.035791061	1		1.072679531	1		1.005224678	1		0.925661402	1		0.947340841	1		1.005132406	1		Quantified
Q9DC11	Plexin domain-containing protein 2	Plxdc2	FUNCTION: May play a role in tumor angioge	Plexin domain-containing p	1.32	1	1	2	530	59.57839164	6.595214844	0.738005325	2	10.72465742	0.723314933	2	17.5126354	0.806536928	2	15.96614207	0.754090766	2	7.645645251	0.892255545	2	22.78843295	2.083923746	2	26.59284481	Quantified
Q01768	Nucleoside diphosphate kinase B	Nme2	FUNCTION: Major role in the synthesis of nuc	Nucleoside diphosphate kir	28.95	1	4	5	152	17.35193207	7.503417969	0.994395466	1		1.093493123	1		0.937956228	1		0.98282681	1		0.967163229	1		1.031623601	1		Quantified
P55284	Cadherin-5	Cdh5	FUNCTION: Cadherins are calcium-depender	Cadherin-5 OS=Mus muscu	2.55	2	2	9	784	87.84825818	5.300292969	1.00832756	9	3.043025667	0.994658515	9	3.679693152	1.010870677	9	1.11260585	0.994988382	9	3.064141263	0.983744034	9	3.540066213	0.991691355	9	4.759411813	Quantified
P84104	Serine/arginine-rich splicing factor 3	Srsf3	FUNCTION: Splicing factor that specifically p	Serine/arginine-rich splicing	4.88	1	1	1	164	19.31790433	11.64892578	0.928471914	1		1.105275885	1		1.352900363	1		0.826158885	1		0.897450547	1		0.816941572	1		Quantified
Q3UQ44	Ras GTPase-activating-like protein IQGAP2	Iqgap2	FUNCTION: Binds to activated CDC42 and R	Ras GTPase-activating-like	2.29	2	4	4	1575	180.4155465	5.643066406	0.953098902	2	3.402603468	0.949610528	2	5.958286701	0.919392901	2	2.915933617	1.014166304	2	7.472857414	1.110014918	2	9.072908082	1.13301598	2	0.968146606	Quantified
Q91WC0	Actin-histidine N-methyltransferase	Setd3	FUNCTION: Protein-histidine N-methyltransfe	Actin-histidine N-methyltran	2.86	1	1	1	594	67.13412083	5.604980469	0.880157626	1		0.78575999	1		1.183901374	1		1.075655676	1		1.145552935	1		1.008735994	1		Quantified
Q9R269	Periplakin	Ppl	FUNCTION: Component of the cornified enve	Periplakin OS=Mus muscul	0.34	1	1	1	1755	203.8803084	5.541503906	0.907862824	1		0.971316757	1		0.996508869	1		1.025483531	1		1.135602123	1		1.045408926	1		Quantified
Q9DBJ1	Phosphoglycerate mutase 1	Pgam1	FUNCTION: Interconversion of 3- and 2-phos	Phosphoglycerate mutase 1	19.29	4	4	8	254	28.8138678	7.181152344	0.97581974	8	4.596880844	1.007564876	8	4.970314413	1.055687892	8	6.768420822	1.012828124	8	5.845978269	1.008640353	8	10.4473378	0.978243095	8	1.074493362	Quantified
Q9CZJ2	Heat shock 70 kDa protein 12B	Hspa12b		Heat shock 70 kDa protein	1.17	1	1	1	685	76.07110812	8.470214844	1.020532531	1		1.007103047	1		0.963828815	1		0.989364945	1		1.005411493	1		1.040648725	1		Quantified
O35737	Heterogeneous nuclear ribonucleoprotein H	Hnrnph1	FUNCTION: This protein is a component of th	Heterogeneous nuclear ribo	16.04	4	5	7	449	49.16840061	6.303222656	0.972048475	6	24.80255721	1.070592927	6	9.420267179	0.972596685	6	25.11330881	0.901493026	6	3.915757859	0.980160003	6	22.06284499	1.004790133	6	5.392263505	Quantified
P49817	Caveolin-1	Cav1	FUNCTION: May act as a scaffolding protein	Caveolin-1 OS=Mus muscu	45.51	7	7	9	178	20.52547669	6.023925781	1.023339249	9	4.032033574	0.988718409	9	4.786882498	1.006084818	9	6.8383849	1.03871666	9	6.710047479	0.95834048	9	8.696679839	1.005964012	9	1.515608379	Quantified
Q8BUK6	Protein Hook homolog 3	Hook3	FUNCTION: Component of the FTS/Hook/FHI	Protein Hook homolog 3 OS	1.25	1	1	1	718	83.16645273	5.186035156	1.032900966	1		0.963647611	1		0.970575894	1		0.993891144	1		1.059632272	1		1.030842858	1		Quantified
Q60716	Prolyl 4-hydroxylase subunit alpha-2	P4ha2	FUNCTION: Catalyzes the post-translational	Prolyl 4-hydroxylase subun	3.72	1	2	2	537	60.96381759	5.795410156	1.007937762	1		0.93951783	1		0.8980428	1		1.143724982	1		1.031927589	1		1.029750011	1		Quantified
Q9JIF7	Coatomer subunit beta	Copb1	FUNCTION: The coatomer is a cytosolic prote	Coatomer subunit beta OS=	10.49	8	8	9	953	106.997675	5.998535156	1.041715962	9	3.369343445	1.004422412	9	5.629552428	1.011142992	9	6.917917595	0.969162301	9	5.005677328	0.977710538	9	10.5898464	1.023039193	9	5.319935043	Quantified
Q3UQU0	Bromodomain-containing protein 9	Brd9	FUNCTION: Plays a role in chromatin remode	Bromodomain-containing pr	1.17	1	1	5	596	66.79833195	6.265136719	1.024302773	5	6.450313728	1.065081029	5	3.309241142	1.020456689	5	0.984915414	0.970684091	5	2.986755974	0.93816283	5	0.535292587	0.998550936	5	5.066244713	Quantified
P16110	Galectin-3	Lgals3	FUNCTION: Galactose-specific lectin which b	Galectin-3 OS=Mus muscul	9.47	2	2	2	264	27.4975067	8.382324219	0.971214534	2	3.466712442	1.02098169	2	2.241973284	1.045287284	2	18.4625414	0.885609235	2	10.22082867	1.151098492	2	3.329152919	0.984813646	2	16.22071623	Quantified
P39054	Dynamin-2	Dnm2	FUNCTION: Microtubule-associated force-pro	Dynamin-2 OS=Mus muscu	7.82	6	6	7	870	98.08407505	7.430175781	1.026414138	7	0.667975797	0.973760478	7	5.091398181	1.032476239	7	4.626777043	0.955160813	7	4.417141668	1.003552889	7	6.994568715	0.956627608	7	8.548440854	Quantified
O35955	Proteasome subunit beta type-10	Psmb10	FUNCTION: The proteasome is a multicatalyt	Proteasome subunit beta ty	10.26	3	3	5	273	29.04498246	6.873535156	1.051100336	5	1.176614413	1.008318248	5	3.860938226	1.037522332	5	5.689459921	0.969514902	5	10.68203967	0.905639194	5	3.380966114	1.033457121	5	1.016869006	Quantified
A3KGB4	TBC1 domain family member 8B	Tbc1d8b	FUNCTION: Involved in vesicular recycling, p	TBC1 domain family memb	0.72	1	1	1	1114	127.8116297	5.490722656	1.017457718	1		1.007663268	1		0.989404589	1		1.000130566	1		1.030490582	1		0.98805084	1		Quantified
P70336	Rho-associated protein kinase 2	Rock2	FUNCTION: Protein kinase which is a key reg	Rho-associated protein kina	3.67	5	5	5	1388	160.484635	5.985839844	1.005419802	5	4.122096887	1.003811424	5	4.544965779	1.001120673	5	7.086060176	1.003632212	5	7.672046045	1.010541614	5	16.85916845	0.972709823	5	7.600512347	Quantified
Q9DCN1	NAD-capped RNA hydrolase NUDT12	Nudt12	FUNCTION: Hydrolyzes NAD(P)H to NMNH a	NAD-capped RNA hydrolas	5.84	3	3	3	462	51.47807064	7.122558594	0.975290504	3	6.967370272	1.038822776	3	0.420098247	0.964821982	3	5.872078445	0.94804234	3	1.739606666	1.000361089	3	1.881550895	1.060637232	3	0.70511817	Quantified
Q8VD75	Huntingtin-interacting protein 1	Hip1	FUNCTION: Plays a role in clathrin-mediated	Huntingtin-interacting protei	1.55	1	1	1	1029	115.1302286	5.427246094	1.002269381	1		1.003268772	1		0.963995152	1		1.034635711	1		0.938992228	1		1.057447817	1		Quantified
Q61107	Guanylate-binding protein 4	Gbp4	FUNCTION: Binds GTP, GDP and GMP. Hyd	Guanylate-binding protein 4	3.23	2	2	2	620	70.75671098	6.639160156	1.017225755	2	0.581974151	0.938127536	2	1.170954361	0.991770669	2	0.583118746	1.026053351	2	2.701029442	0.955044838	2	19.87924333	1.074015094	2	9.024711052	Quantified
O08807	Peroxiredoxin-4	Prdx4	FUNCTION: Thiol-specific peroxidase that ca	Peroxiredoxin-4 OS=Mus m	9.12	1	2	2	274	31.03314207	7.151855469	0.945313332	1		1.002650155	1		1.115932914	1		0.952919989	1		1.055276958	1		0.958155225	1		Quantified
P35282	Ras-related protein Rab-21	Rab21	FUNCTION: Regulates integrin internalization	Ras-related protein Rab-21	4.5	1	1	2	222	24.09115037	7.942871094	1.175399232	2	21.05176908	0.96548886	2	0.483894167	0.966500924	2	3.344472103	1.064850853	2	3.857115466	0.874646796	2	20.44776313	0.900147351	2	16.69497226	Quantified
Q8R1K1	Ubiquitin-associated domain-containing protein 2	Ubac2	FUNCTION: Restricts trafficking of FAF2 from	Ubiquitin-associated domai	2.32	1	1	7	345	39.03145401	9.437011719	1.002470022	7	5.434506765	0.997048512	7	1.815564123	0.998021657	7	5.28739532	1.076419012	7	4.367356019	0.876412614	7	4.283576283	0.962554745	7	2.74048654	Quantified
P97461	40S ribosomal protein S5	Rps5		40S ribosomal protein S5 C	8.82	2	2	2	204	22.87504872	9.715332031	1.006083586	2	1.362229558	1.034914612	2	2.42442891	1.028546507	2	2.457635086	0.996829292	2	0.79267099	0.946863512	2	6.243286156	0.977855349	2	2.782617472	Quantified
Q91WG2	Rab GTPase-binding effector protein 2	Rabep2	FUNCTION: Plays a role in membrane traffick	Rab GTPase-binding effecto	1.62	1	1	1	554	62.09481977	4.894042969	0.999500056	1		1.04311196	1		1.070242096	1		1.002164449	1		0.946150008	1		0.924232176	1		Quantified
Q3U0V1	Far upstream element-binding protein 2	Khsrp	FUNCTION: Binds to the dendritic targeting e	Far upstream element-bindi	6.42	2	5	7	748	76.7277136	7.327636719	0.979493273	3	0.789701941	1.019147885	3	0.915393261	1.133262408	3	3.746939163	0.921790889	3	2.299325128	0.965641582	3	1.552500672	0.951243182	3	0.157660587	Quantified
Q8R326	Paraspeckle component 1	Pspc1	FUNCTION: Together with NONO, required fo	Paraspeckle component 1 (	2.87	2	2	2	523	58.72128057	6.668457031	1.003718451	1		0.993707985	1		0.997405605	1		1.105867398	1		1.021641523	1		0.906024578	1		Quantified
Q61781	Keratin, type I cytoskeletal 14	Krt14	FUNCTION: The nonhelical tail domain is inv	Keratin, type I cytoskeletal	7.85	0	4	5	484	52.83388545	5.173339844																			Identified
P33434	72 kDa type IV collagenase	Mmp2	FUNCTION: Ubiquitinous metalloproteinase t	72 kDa type IV collagenase	8.16	5	5	7	662	74.0548623	5.528808594	0.978572511	7	3.572604892	1.037484912	7	7.60314662	0.9781219	7	6.464616929	1.008336055	7	10.01461045	1.077248805	7	8.577497734	0.934691212	7	10.23012397	Quantified
Q6P9S7	Polypeptide N-acetylgalactosaminyltransferase 10	Galnt10	FUNCTION: Catalyzes the initial reaction in C	Polypeptide N-acetylgalacto	1	1	1	1	603	69.07185693	8.426269531	0.93140873	1		1.054925624	1		1.023614834	1		1.014857965	1		0.965369924	1		1.010809953	1		Quantified
Q8BH43	Wiskott-Aldrich syndrome protein family member 2	Wasf2	FUNCTION: Downstream effector molecule in	Wiskott-Aldrich syndrome p	5.43	2	2	2	497	54.03996167	5.528808594	1.010974013	2	3.75523802	1.044887684	2	0.803504647	1.129471915	2	7.544211082	0.885773694	2	14.36902557	0.936122831	2	6.462428394	0.958672949	2	10.37728127	Quantified
Q99KN9	Clathrin interactor 1	Clint1	FUNCTION: Binds to membranes enriched in	Clathrin interactor 1 OS=Mu	6.34	3	3	3	631	68.46877091	6.252441406	0.976979141	3	0.779491537	1.025310833	3	0.747678146	0.984383565	3	2.245140204	0.964736814	3	3.905373591	1.033669634	3	3.617701234	1.049267151	3	4.496775682	Quantified
Q9R0P5	Destrin	Dstn	FUNCTION: Actin-depolymerizing protein. Se	Destrin OS=Mus musculus	14.55	2	2	3	165	18.50948631	7.972167969	0.993639086	3	1.163332248	0.98672829	3	0.063775913	0.980448498	3	1.295042609	1.064091847	3	2.880533889	1.077023461	3	0.726734033	0.977661886	3	4.022344121	Quantified
Q8C5L6	Inositol polyphosphate 5-phosphatase K	Inpp5k	FUNCTION: Inositol 5-phosphatase which act	Inositol polyphosphate 5-ph	1.28	1	1	1	468	54.12422973	5.884277344	0.942026105	1		0.958297364	1		0.970439636	1		1.1357927	1		1.018997548	1		1.011250462	1		Quantified
P08556	GTPase NRas	Nras	FUNCTION: Ras proteins bind GDP/GTP and	GTPase NRas OS=Mus mu	9.52	2	2	2	189	21.18552269	5.173339844	1.053107224	2	5.798975843	0.987190922	2	4.731341601	1.059413332	2	1.061922888	1.038447742	2	3.814272321	0.907966991	2	3.527532152	0.92549558	2	6.38067633	Quantified
Q8BFQ4	WD repeat-containing protein 82	Wdr82	FUNCTION: Regulatory component of the SE	WD repeat-containing prote	4.47	1	1	1	313	35.05646656	7.693847656	0.928808172	1		1.00711	1		1.012817118	1		1.119835556	1		0.982469088	1		0.961139012	1		Quantified
Q9D0T1	NHP2-like protein 1	Snu13	FUNCTION: Involved in pre-mRNA splicing a	NHP2-like protein 1 OS=Mu	15.63	2	2	2	128	14.16455536	8.455566406	0.869118021	2	4.679990755	0.930409728	2	1.193423574	1.026247978	2	5.182563408	1.188413102	2	3.409218709	1.333532212	2	3.412680982	0.809917567	2	2.712378394	Quantified
Q8R3C0	Mini-chromosome maintenance complex-binding prot	Mcmbp	FUNCTION: Associated component of the MC	Mini-chromosome maintena	1.56	1	1	1	642	72.84476718	5.655761719	1.072506675	1		0.933216381	1		1.047170625	1		0.985446992	1		1.020709359	1		0.967948449	1		Quantified
Q3TDD9	Protein phosphatase 1 regulatory subunit 21	Ppp1r21	FUNCTION: utative regulator of protein phosp	Protein phosphatase 1 regu	0.9	1	1	1	780	88.28239628	6.902832031	0.982491212	1		0.95488049	1		0.959393651	1		0.88503442	1		1.320274276	1		1.060357871	1		Quantified
Q9Z315	U4/U6.U5 tri-snRNP-associated protein 1	Sart1	FUNCTION: Plays a role in mRNA splicing as	U4/U6.U5 tri-snRNP-associ	1.49	1	2	10	806	90.83037619	5.820800781																			Identified
Q8K2H4	Arf-GAP with coiled-coil, ANK repeat and PH domain	Acap1	FUNCTION: GTPase-activating protein (GAP)	Arf-GAP with coiled-coil, AN	3.92	2	2	2	740	81.65266583	7.518066406	1.069978406	2	11.91813431	0.96208827	2	12.4690992	0.989636454	2	4.527691221	0.975262409	2	2.572663859	1.048078657	2	2.848662971	0.989590984	2	4.439621233	Quantified
P70398	Probable ubiquitin carboxyl-terminal hydrolase FAF-X	Usp9x	FUNCTION: Deubiquitinase involved both in t	Probable ubiquitin carboxyl	0.98	3	3	4	2559	290.5257656	5.871582031	0.950620813	4	5.236422423	0.958451955	4	2.033913586	1.005741529	4	2.194324106	1.084452181	4	2.949955108	1.031562477	4	2.20729591	0.98293211	4	6.088008036	Quantified
P54923	[Protein ADP-ribosylarginine] hydrolase	Adprh	FUNCTION: Specifically acts as a arginine m	[Protein ADP-ribosylarginine	1.93	1	1	1	362	40.042411	5.757324219	1.046862989	1		0.895505214	1		0.992125614	1		1.084331438	1		1.126977652	1		0.934155491	1		Quantified
Q9EQQ9	Protein O-GlcNAcase	Oga	FUNCTION: Cleaves GlcNAc but not GalNAc	Protein O-GlcNAcase OS=M	1.31	1	1	1	916	103.0959472	4.919433594	1.063251576	1		1.092476908	1		1.01311853	1		0.988625807	1		0.840420155	1		0.945587361	1		Quantified
Q68FH4	N-acetylgalactosamine kinase	Galk2	FUNCTION: Acts on GalNAc. Also acts as a g	N-acetylgalactosamine kina	3.49	2	2	3	458	50.47049017	6.902832031	0.997678038	3	0.926888342	0.980436574	3	1.035501963	1.006788233	3	2.34927795	1.006961672	3	0.250681827	1.014390972	3	15.36356837	1.003377072	3	4.499186081	Quantified
Q9QWL7	Keratin, type I cytoskeletal 17	Krt17	FUNCTION: Type I keratin involved in the for	Keratin, type I cytoskeletal	8.31	1	4	5	433	48.1320687	5.059082031	1.117672942	2	8.008057167	1.254291015	2	2.500251086	0.86836783	2	21.43036045	0.928511956	2	26.7038498	0.65279139	2	16.41128866	1.019286008	2	4.458520729	Quantified
Q8C115	Pleckstrin homology domain-containing family H mer	Plekhh2	FUNCTION: In the kidney glomerulus may pla	Pleckstrin homology domai	0.4	1	1	2	1491	167.627278	7.474121094	1.002935008	2	1.464675163	0.973436621	2	0.495431357	1.00884019	2	0.106719339	1.017630445	2	8.558066912	0.942182214	2	1.271708134	1.050743815	2	8.080358118	Quantified
Q9CQF9	Prenylcysteine oxidase	Pcyox1	FUNCTION: Involved in the degradation of pr	Prenylcysteine oxidase OS	4.75	2	2	3	505	56.45867543	6.917480469	0.999712974	3	0.088338528	0.999013371	3	1.858390133	0.943453328	3	4.713286435	1.044029143	3	7.956556381	1.049055664	3	1.874751438	1.030661684	3	4.306349048	Quantified
Q9D3E6	Cohesin subunit SA-1	Stag1	FUNCTION: Component of cohesin complex,	Cohesin subunit SA-1 OS=I	0.64	1	1	1	1258	144.3487134	5.592285156	0.961851868	1		0.998445818	1		0.975009014	1		1.034274629	1		1.032718044	1		1.036443151	1		Quantified
Q91YR7	Pre-mRNA-processing factor 6	Prpf6	FUNCTION: Involved in pre-mRNA splicing a	Pre-mRNA-processing facto	1.28	1	1	1	941	106.6547413	8.089355469	1.01360138	1		0.96066574	1		0.864080064	1		1.118653855	1		1.077332006	1		1.037453306	1		Quantified
Q3V4B5	COMM domain-containing protein 6	Commd6	FUNCTION: May modulate activity of cullin-R	COMM domain-containing p	10.34	1	1	1	87	9.788906215	5.376464844	0.999968811	1		0.8358195	1		0.963227108	1		1.031385451	1		1.13062743	1		1.133807071	1		Quantified
Q9JII6	Aldo-keto reductase family 1 member A1	Akr1a1	FUNCTION: Catalyzes the NADPH-depender	Aldo-keto reductase family	15.38	5	5	5	325	36.56392832	7.386230469	0.97550522	5	3.282790403	0.994821389	5	1.354705877	1.000789507	5	3.407139404	1.023802495	5	3.658311544	1.072753759	5	6.188825686	0.964807554	5	2.660971489	Quantified
P40237	CD82 antigen	Cd82	FUNCTION: Associates with CD4 or CD8 and	CD82 antigen OS=Mus mus	4.14	1	1	1	266	29.60888622	5.020996094	1.056957938	1		0.994288157	1		1.04569418	1		0.966130988	1		0.921747294	1		0.997615797	1		Quantified
P63168	Dynein light chain 1, cytoplasmic	Dynll1	FUNCTION: Acts as one of several non-catal	Dynein light chain 1, cytopla	7.87	1	1	2	89	10.35910842	7.400878906	0.957378918	2	0.890608764	0.964344912	2	6.972252988	0.939122828	2	9.189965914	1.046899182	2	6.214011741	1.206367326	2	8.031618405	0.997100547	2	0.680610089	Quantified
Q8K4K6	Pantothenate kinase 1	Pank1	FUNCTION: Plays a role in the physiological	Pantothenate kinase 1 OS=	4.38	2	2	3	548	60.05302381	8.030761719	1.083665787	3	2.336374836	0.96328936	3	0.220773789	1.055785532	3	1.8294939	0.924193876	3	0.716977481	0.95331489	3	2.318835081	1.00396307	3	2.334403452	Quantified
Q99LD4	COP9 signalosome complex subunit 1	Gps1	FUNCTION: Essential component of the COP	COP9 signalosome comple	2.55	1	1	1	471	53.4081289	6.844238281	1.033263675	1		0.945252795	1		0.945980336	1		1.046698702	1		1.138261996	1		0.977581037	1		Quantified
Q9Z0P5	Twinfilin-2	Twf2	FUNCTION: Actin-binding protein involved in	Twinfilin-2 OS=Mus muscul	7.74	2	2	2	349	39.44639312	6.800292969	0.931186479	2	1.037090441	0.924731788	2	13.59698761	0.963349085	2	9.627997947	0.980819089	2	7.002325405	1.168820583	2	1.572282775	1.108256138	2	28.6354332	Quantified
Q8K012	Formin-binding protein 1-like	Fnbp1l	FUNCTION: Required to coordinate membran	Formin-binding protein 1-lik	1.65	1	1	2	605	69.8415642	6.639160156	0.885499748	2	4.698293935	1.00004707	2	5.874885216	0.785950911	2	19.32892733	1.104250382	2	2.860089088	1.205900813	2	5.995281106	1.143294255	2	6.53829086	Quantified
P28659	CUGBP Elav-like family member 1	Celf1	FUNCTION: RNA-binding protein implicated i	CUGBP Elav-like family me	1.65	1	1	1	486	52.0738936	8.455566406	0.987932954	1		0.979280286	1		1.072638117	1		1.047119956	1		0.974089284	1		0.941376179	1		Quantified
Q80X19	Collagen alpha-1(XIV) chain	Col14a1	FUNCTION: Plays an adhesive role by integr	Collagen alpha-1(XIV) chai	1.34	3	3	6	1797	192.891752	5.097167969	0.969173847	6	0.957405574	0.934939878	6	4.568655576	0.954918659	6	6.350061437	1.092563086	6	5.341493695	1.035953774	6	9.204070745	1.039587621	6	5.297466926	Quantified
Q8BYC6	Serine/threonine-protein kinase TAO3	Taok3	FUNCTION: Serine/threonine-protein kinase t	Serine/threonine-protein kir	1.67	1	1	1	898	105.2697877	7.356933594	0.967093939	1		0.96156766	1		0.966572759	1		1.094742025	1		1.112695719	1		0.97147627	1		Quantified
P97464	Exostosin-1	Ext1	FUNCTION: Glycosyltransferase required for	Exostosin-1 OS=Mus musc	4.69	4	4	4	746	86.25281973	9.041503906	1.012045065	4	2.549064461	0.988451898	4	3.054296943	1.003455694	4	8.000553203	1.026602838	4	6.329308071	1.021624924	4	12.15513178	0.991290643	4	5.035540992	Quantified
Q61103	Zinc finger protein ubi-d4	Dpf2	FUNCTION: Plays an active role in transcripti	Zinc finger protein ubi-d4 O	2.3	1	1	1	391	44.20122598	6.468261719	0.792417218	1		0.707864254	1		0.724671335	1		1.999387892	1		1.11062538	1		0.786719956	1		Quantified
Q91YE3	Egl nine homolog 1	Egln1	FUNCTION: Cellular oxygen sensor that cata	Egl nine homolog 1 OS=Mu	2.75	1	1	1	400	43.08332439	8.294433594	1.013055466	1		1.003435062	1		0.945365472	1		1.029591124	1		0.967263499	1		1.055372323	1		Quantified
Q01320	DNA topoisomerase 2-alpha	Top2a	FUNCTION: Control of topological states of D	DNA topoisomerase 2-alpha	1.9	1	3	6	1528	172.6821044	8.602050781	1.023215669	2	0.522136106	0.969085781	2	11.23492943	0.999614073	2	1.54199848	0.998001494	2	3.522033924	1.011525557	2	14.16480887	1.020423127	2	1.004199869	Quantified
Q9CS42	Ribose-phosphate pyrophosphokinase 2	Prps2	FUNCTION: Catalyzes the synthesis of phosp	Ribose-phosphate pyropho	2.2	1	1	1	318	34.76396901	6.609863281																			Identified
P62869	Elongin-B	Elob	FUNCTION: SIII, also known as elongin, is a	Elongin-B OS=Mus musculu	17.8	2	2	2	118	13.16164886	5.008300781	1.031956293	2	0.74023032	0.939349264	2	2.929326212	1.021805684	2	5.831139805	1.023030472	2	5.914578698	1.061895825	2	2.10367285	0.967164544	2	1.553130259	Quantified
Q6ZWV7	60S ribosomal protein L35	Rpl35	FUNCTION: Component of the large ribosoma	60S ribosomal protein L35 (	4.88	1	1	8	123	14.54361773	11.04833984																			Identified
Q9D0W5	Peptidyl-prolyl cis-trans isomerase-like 1	Ppil1	FUNCTION: Involved in pre-mRNA splicing a	Peptidyl-prolyl cis-trans isor	6.63	1	1	2	166	18.22511925	7.986816406	0.998657718	2	4.0537939	0.970990176	2	3.0009057	1.03806397	2	0.927112874	1.008729489	2	2.253524414	1.027068717	2	6.714929404	0.984677594	2	0.119620212	Quantified
Q8BTU1	Cilia- and flagella-associated protein 20	Cfap20	FUNCTION: Cilium- and flagellum-specific pro	Cilia- and flagella-associate	4.66	1	1	1	193	22.73394139	9.759277344	1.052762087	1		1.040001492	1		0.962762265	1		0.999758412	1		0.985733533	1		0.973100509	1		Quantified
Q01339	Beta-2-glycoprotein 1	Apoh	FUNCTION: Binds to various kinds of negativ	Beta-2-glycoprotein 1 OS=M	2.61	1	1	1	345	38.59291033	8.221191406	0.998434127	1		1.042912567	1		1.003666245	1		0.951032602	1		1.118397931	1		0.950030249	1		Quantified
Q9QYJ0	DnaJ homolog subfamily A member 2	Dnaja2	FUNCTION: Co-chaperone of Hsc70. Stimula	DnaJ homolog subfamily A	1.94	1	1	1	412	45.71659277	6.480957031	1.007434578	1		1.09348558	1		1.05371809	1		0.938898418	1		0.968423098	1		0.930344374	1		Quantified
P56480	ATP synthase subunit beta, mitochondrial	Atp5f1b	FUNCTION: Mitochondrial membrane ATP sy	ATP synthase subunit beta,	4.91	2	2	2	529	56.26546922	5.338378906	0.987082612	2	8.341841822	0.955563889	2	6.22979965	0.91371021	2	9.258731037	1.085510734	2	12.76910939	1.030079884	2	3.811375697	1.06619243	2	7.788063948	Quantified
Q60963	Platelet-activating factor acetylhydrolase	Pla2g7	FUNCTION: Lipoprotein-associated calcium-i	Platelet-activating factor ac	1.36	1	1	1	440	49.22699648	7.122558594	0.994211011	1		1.033697966	1		0.990780612	1		0.997708983	1		0.987641594	1		1.01021983	1		Quantified
Q923G2	DNA-directed RNA polymerases I, II, and III subunit R	Polr2h	FUNCTION: DNA-dependent RNA polymeras	DNA-directed RNA polymer	8.67	1	1	1	150	17.13244875	4.678222656	1.039547027	1		0.798821364	1		0.967839791	1		1.225235783	1		1.052153147	1		0.977310195	1		Quantified
P61971	Nuclear transport factor 2	Nutf2	FUNCTION: Mediates the import of GDP-bou	Nuclear transport factor 2 C	4.72	1	1	3	127	14.46904904	5.376464844	0.957096649	3	2.019664029	1.018787409	3	2.249973612	1.044469819	3	0.146986602	1.009264368	3	1.318337581	0.941465576	3	4.522343466	1.013526715	3	1.523552321	Quantified
Q60715	Prolyl 4-hydroxylase subunit alpha-1	P4ha1	FUNCTION: Catalyzes the post-translational	Prolyl 4-hydroxylase subun	5.81	3	3	4	534	60.87179673	5.896972656	0.981904668	4	2.113940033	1.052107788	4	6.017735068	0.962903808	4	3.627081446	0.967577217	4	7.852337824	1.03885227	4	7.837155636	1.017716513	4	3.022972235	Quantified
Q9DBD0	Inhibitor of carbonic anhydrase	Ica	FUNCTION: Inhibitor for carbonic anhydrase 2	Inhibitor of carbonic anhydr	2.57	2	2	4	700	76.71546626	7.254394531	1.011329573	4	2.417672984	1.021033119	4	0.37263638	1.001882413	4	3.255149894	0.967049991	4	3.128026015	1.026844418	4	6.928754642	1.018546441	4	3.007340831	Quantified
Q9WVJ9	EGF-containing fibulin-like extracellular matrix protein	Efemp2		EGF-containing fibulin-like e	7.45	3	3	3	443	49.39225753	4.894042969	0.939448422	3	1.504972701	0.91241718	3	0.329095344	1.061359869	3	0.62174435	1.059353932	3	1.366093327	0.989417374	3	2.599887106	0.949931826	3	2.636971429	Quantified
Q9CQI7	U2 small nuclear ribonucleoprotein B''	Snrpb2	FUNCTION: Involved in pre-mRNA splicing a	U2 small nuclear ribonucleo	7.56	1	2	2	225	25.30704353	9.715332031	0.922090733	1		0.928364006	1		0.979509236	1		1.098284531	1		1.071218785	1		1.061710688	1		Quantified
P62858	40S ribosomal protein S28	Rps28		40S ribosomal protein S28	17.39	1	1	2	69	7.836201805	10.69677734	0.938321681	2	0.123136112	0.849255217	2	12.59837228	1.024315452	2	16.23715336	1.113879152	2	2.754855779	1.200809162	2	15.48398221	0.961027261	2	23.65409312	Quantified
Q9Z2T6	Keratin, type II cuticular Hb5	Krt85		Keratin, type II cuticular Hb	5.13	2	3	3	507	55.72329956	6.417480469	1.02537596	2	11.7909009	1.022192308	2	26.80371465	0.829067785	2	11.18467151	1.168534209	2	11.83225702	1.024192827	2	23.20008184	0.942695805	2	3.938162161	Quantified
Q9CR16	Peptidyl-prolyl cis-trans isomerase D	Ppid	FUNCTION: PPIase that catalyzes the cis-tra	Peptidyl-prolyl cis-trans isor	11.35	4	5	5	370	40.71674608	7.430175781	1.010859867	4	1.753648212	1.058984256	4	3.819927751	1.034009753	4	0.179362119	0.942918608	4	1.809209116	0.972676607	4	0.716090109	0.980484929	4	4.398735041	Quantified
Q9R1C7	Pre-mRNA-processing factor 40 homolog A	Prpf40a	FUNCTION: Binds to WASL/N-WASP and su	Pre-mRNA-processing facto	1.36	1	1	2	953	108.4124122	7.693847656	1.051728618	2	2.313225402	0.879283396	2	16.01688202	1.00658069	2	8.534563872	1.025665455	2	4.397802591	1.132503392	2	13.15059928	0.973891568	2	10.64846712	Quantified
Q8BY87	Ubiquitin carboxyl-terminal hydrolase 47	Usp47	FUNCTION: Ubiquitin-specific protease that s	Ubiquitin carboxyl-terminal	0.87	1	1	1	1376	157.3563658	5.109863281	1.004031929	1		1.046051707	1		0.972161685	1		0.965523385	1		1.039368828	1		1.009295836	1		Quantified
O55142	60S ribosomal protein L35a	Rpl35a	FUNCTION: Required for the proliferation and	60S ribosomal protein L35a	8.18	1	1	3	110	12.54574823	10.88720703	1.00092308	3	4.068565801	1.025204844	3	0.518238479	1.009226366	3	8.116612721	0.986316661	3	3.326852428	0.996569948	3	2.265286783	1.002982692	3	2.843599382	Quantified
O55128	Histone deacetylase complex subunit SAP18	Sap18	FUNCTION: Component of the SIN3-repressi	Histone deacetylase comple	13.07	2	2	2	153	17.5839565	9.349121094	0.963888526	2	3.592874964	0.961309957	2	2.24980749	0.924246673	2	9.745586251	1.101198267	2	8.483666212	1.019416045	2	3.912356511	1.066784183	2	7.216507808	Quantified
P27046	Alpha-mannosidase 2	Man2a1	FUNCTION: Catalyzes the first committed ste	Alpha-mannosidase 2 OS=I	0.52	1	1	1	1150	131.5476682	8.030761719	0.983383641	1		0.942879216	1		0.991067709	1		0.908220586	1		1.094053761	1		1.148939277	1		Quantified
Q8K2I3	Dimethylaniline monooxygenase [N-oxide-forming] 2	Fmo2	FUNCTION: This protein is involved in the ox	Dimethylaniline monooxyge	6.54	4	4	6	535	60.93537281	8.470214844	1.073649399	6	8.211246875	1.099171148	6	10.29171649	0.944603363	6	2.755082533	0.957209171	6	8.453127205	0.885496363	6	5.014771929	0.979123866	6	7.589111387	Quantified
P01872	Immunoglobulin heavy constant mu	Ighm	FUNCTION: IgM antibodies play an important	Immunoglobulin heavy cons	6.83	3	3	3	454	49.94028842	7.005371094	0.954099425	3	1.169947913	1.00882393	3	0.409884042	1.085603989	3	3.479413767	0.963029483	3	0.483175934	0.960186057	3	6.219698977	0.967439599	3	4.517083515	Quantified
Q63810	Calcineurin subunit B type 1	Ppp3r1	FUNCTION: Regulatory subunit of calcineurin	Calcineurin subunit B type 1	17.65	3	3	3	170	19.2875765	4.805175781	1.041251928	3	2.700982079	1.062544295	3	1.66799985	0.9847362	3	4.382142349	0.982131411	3	0.52067506	0.935343778	3	1.04798265	1.01889492	3	0.990889582	Quantified
P62305	Small nuclear ribonucleoprotein E	Snrpe	FUNCTION: Plays role in pre-mRNA splicing	Small nuclear ribonucleopro	13.04	1	1	1	92	10.7966374	9.437011719	1.017570246	1		0.953381364	1		1.061283199	1		0.975360726	1		1.047309211	1		0.981252839	1		Quantified
Q80XA6	RalBP1-associated Eps domain-containing protein 2	Reps2	FUNCTION: Involved in growth factor signalin	RalBP1-associated Eps dor	1.54	1	1	1	521	57.71390743	7.532714844	0.953971556	1		0.965086441	1		1.008940169	1		1.032153998	1		1.056261499	1		1.0309011	1		Quantified
Q8VDM6	Heterogeneous nuclear ribonucleoprotein U-like prote	Hnrnpul1	FUNCTION: Acts as a basic transcriptional re	Heterogeneous nuclear ribo	1.63	2	2	2	859	95.94249422	6.580566406	0.983786114	2	10.90093908	0.970206858	2	2.14108569	1.03512166	2	6.909517454	0.930355253	2	0.922571181	0.932375355	2	0.02398721	1.130000695	2	18.43419486	Quantified
Q04592	Proprotein convertase subtilisin/kexin type 5	Pcsk5	FUNCTION: Serine endoprotease that proces	Proprotein convertase subti	0.85	2	2	2	1877	209.1153697	6.087402344	1.028324895	2	0.566333252	0.989734343	2	1.458892013	0.985010751	2	1.662288563	1.061201435	2	2.891720687	0.948216345	2	2.958847931	0.988123889	2	1.121880183	Quantified
Q91W10	Metal cation symporter ZIP8	Slc39a8	FUNCTION: Electroneutral transporter of the p	Metal cation symporter ZIP8	1.52	1	1	4	462	50.04958895	5.858886719	0.963458866	4	4.648473454	1.043994688	4	3.669956969	0.972732587	4	11.33465359	0.948480712	4	6.010681516	0.986417529	4	3.294121291	1.023160583	4	1.469396695	Quantified
P62309	Small nuclear ribonucleoprotein G	Snrpg	FUNCTION: Plays role in pre-mRNA splicing	Small nuclear ribonucleopro	15.79	1	1	1	76	8.490427635	8.880371094	0.90747649	1		0.908442799	1		0.960983519	1		1.13322825	1		1.192652819	1		1.011368094	1		Quantified
O88947	Coagulation factor X	F10	FUNCTION: Factor Xa is a vitamin K-depende	Coagulation factor X OS=M	1.87	1	1	1	481	53.98318146	5.655761719	1.019606668	1		1.03141437	1		1.032178092	1		0.969874064	1		1.053886813	1		0.927714153	1		Quantified
Q9QZR9	Collagen alpha-4(IV) chain	Col4a4	FUNCTION: Type IV collagen is the major str	Collagen alpha-4(IV) chain	0.59	1	1	1	1682	163.9928233	8.543457031	0.902045758	1		0.947357049	1		0.898553811	1		1.146904545	1		1.141134771	1		1.062125115	1		Quantified
Q9WTL7	Acyl-protein thioesterase 2	Lypla2	FUNCTION: Acts as a acyl-protein thioestera	Acyl-protein thioesterase 2	3.46	1	1	1	231	24.77771373	7.225097656	0.905296834	1		0.935943184	1		1.039950982	1		1.01642178	1		1.177746866	1		1.021624642	1		Quantified
Q61161	Mitogen-activated protein kinase kinase kinase kinas	Map4k2	FUNCTION: Serine/threonine-protein kinase w	Mitogen-activated protein ki	1.95	1	1	1	821	91.20797592	6.455566406	1.210928756	1		0.602567323	1		1.052640883	1		1.036146092	1		1.438190858	1		0.879743635	1		Quantified
O89026	Roundabout homolog 1	Robo1	FUNCTION: Receptor for SLIT1 and SLIT2 th	Roundabout homolog 1 OS	2.98	5	5	5	1612	176.2973662	6.176269531	0.96189496	5	4.084208872	1.007740841	5	2.651897895	0.984356106	5	3.555551651	1.020868947	5	3.922658534	1.067270464	5	5.675004212	0.96151626	5	0.802074975	Quantified
Q8R574	Phosphoribosyl pyrophosphate synthase-associated	Prpsap2	FUNCTION: Seems to play a negative regula	Phosphoribosyl pyrophosph	5.96	2	2	2	369	40.85436181	7.166503906	0.972854029	2	2.272603942	0.983438725	2	5.181226032	0.94631071	2	10.50096931	1.10021604	2	13.01634293	1.114364798	2	13.25746842	0.946927744	2	4.451153031	Quantified
Q6IQX7	Chondroitin sulfate synthase 2	Chpf	FUNCTION: Has both beta-1,3-glucuronic aci	Chondroitin sulfate synthas	1.03	1	1	1	774	85.48089476	6.844238281	1.017744954	1		1.004596739	1		0.895832003	1		1.035968612	1		1.040428978	1		1.055087542	1		Quantified
P60122	RuvB-like 1	Ruvbl1	FUNCTION: Possesses single-stranded DNA	RuvB-like 1 OS=Mus musc	8.11	3	3	3	456	50.18230083	6.417480469	1.002124586	3	5.777034266	0.93107846	3	2.956716089	0.952996853	3	0.27920162	1.072641183	3	6.46632297	0.998381039	3	1.673873276	1.042479492	3	5.739708058	Quantified
Q61545	RNA-binding protein EWS	Ewsr1	FUNCTION: Might function as a transcriptiona	RNA-binding protein EWS (	2.14	1	1	3	655	68.41976533	9.334472656	0.981864074	3	1.508862213	0.994874075	3	0.193985073	1.076796613	3	10.0675631	1.067088821	3	1.582743226	0.938991185	3	0.44586451	1.039032982	3	4.976008725	Quantified
Q9WUA2	Phenylalanine--tRNA ligase beta subunit	Farsb		Phenylalanine--tRNA ligase	4.92	3	3	4	589	65.6553238	7.122558594	1.052822076	4	1.385138841	1.077338424	4	0.775558499	0.994984306	4	2.911750519	0.938804882	4	3.782927864	0.923997014	4	0.76028083	0.959917414	4	6.084555664	Quantified
Q9JMG1	Endothelial differentiation-related factor 1	Edf1	FUNCTION: Transcriptional coactivator stimu	Endothelial differentiation-re	8.11	1	1	1	148	16.358916	9.993652344																			Identified
Q3UVV9	von Willebrand factor A domain-containing protein 3A	Vwa3a		von Willebrand factor A dor	0.7	1	1	1	1148	130.0240602	8.382324219	0.997326752	1		0.90474271	1		1.013788497	1		0.986288416	1		1.284420048	1		0.956474034	1		Quantified
Q9QUN9	Dickkopf-related protein 3	Dkk3	FUNCTION: Antagonizes canonical Wnt signa	Dickkopf-related protein 3 (	4.01	1	1	1	349	38.36281549	4.538574219	1.084589539	1		1.00218809	1		0.891765215	1		1.065739542	1		1.141529538	1		0.901431468	1		Quantified
Q91WT9	Cystathionine beta-synthase	Cbs	FUNCTION: Hydro-lyase catalyzing the first s	Cystathionine beta-synthas	1.43	1	1	1	561	61.50423926	6.480957031	0.960751321	1		0.982586802	1		0.925608011	1		0.967478156	1		0.916865983	1		1.249216556	1		Quantified

A2AQ19	RNA polymerase-associated protein RTF1 homolog	Rtf1	FUNCTION: Component of the PAF1 comple	RNA polymerase-associate	0.98	1	1	1	715	80.74790514	8.162597656	0.960441125	1		1.010798622	1		1.096711256	1		1.014273218	1		0.984879586	1		0.934907205	1		Quantified
P35991	Tyrosine-protein kinase BTK	Btk	FUNCTION: Non-receptor tyrosine kinase ind	Tyrosine-protein kinase BTI	2.12	1	1	1	659	76.38849968	7.898925781	1.074958589	1		1.026351585	1		1.083002119	1		0.900007671	1		0.954082626	1		0.949033423	1		Quantified
P21279	Guanine nucleotide-binding protein G(q) subunit alph	Gnaq	FUNCTION: Guanine nucleotide-binding prot	Guanine nucleotide-binding	8.36	3	3	4	359	42.13131112	5.681152344	1.036083592	4	4.111937875	0.997729699	4	3.909564548	0.979858236	4	1.770866749	0.982300718	4	5.797912273	1.059535017	4	2.309944173	1.013988333	4	3.911112708	Quantified
Q99P58	Ras-related protein Rab-27B	Rab27b	FUNCTION: Small GTPase which cycles betv	Ras-related protein Rab-27	4.59	1	1	1	218	24.54421947	5.541503906	1.154532251	1		0.815160931	1		1.059147489	1		0.972173683	1		1.152641801	1		0.933357305	1		Quantified
Q9EPK7	Exportin-7	Xpo7	FUNCTION: Mediates the nuclear export of p	Exportin-7 OS=Mus muscul	3.22	3	3	3	1087	123.7309111	6.379394531	1.009683773	3	5.099207952	0.989982372	3	3.092016817	0.965748754	3	5.959037374	1.003731399	3	0.861271023	1.048620471	3	4.970515562	0.999298846	3	0.844691187	Quantified
Q78PG9	Coiled-coil domain-containing protein 25	Ccdc25		Coiled-coil domain-containi	13.94	3	3	5	208	24.46420336	6.946777344	0.940216608	5	5.916788174	1.010747453	5	4.965414717	1.036876503	5	3.986161045	0.935389047	5	5.836269923	0.906820666	5	6.818896128	1.115267919	5	30.04648512	Quantified
P25799	Nuclear factor NF-kappa-B p105 subunit	Nfkb1	FUNCTION: NF-kappa-B is a pleiotropic trans	Nuclear factor NF-kappa-B	1.44	1	1	1	971	105.5491204	5.389160156	0.993744979	1		1.016919112	1		0.91887998	1		1.074312482	1		1.033604568	1		1.005226138	1		Quantified
Q02248	Catenin beta-1	Ctnnb1	FUNCTION: Key downstream component of t	Catenin beta-1 OS=Mus mu	4.1	3	3	4	781	85.41620078	5.858886719	0.986545012	4	7.640212855	1.057609948	4	3.466052107	1.032407059	4	2.170900758	0.958586501	4	0.633721471	0.987312344	4	4.291288774	0.983989916	4	4.573752652	Quantified
O35593	26S proteasome non-ATPase regulatory subunit 14	Psmd14	FUNCTION: Component of the 26S proteasor	26S proteasome non-ATPa	9.68	3	3	4	310	34.55455753	6.521972656	1.00625479	4	4.383209819	1.007052473	4	3.425328532	0.968295245	4	7.554340454	1.023465237	4	4.203993804	0.962491942	4	5.746151794	1.009943053	4	7.309777865	Quantified
P54265	Myotonin-protein kinase	Dmpk	FUNCTION: Non-receptor serine/threonine pr	Myotonin-protein kinase OS	0.95	1	1	1	631	69.55716403	4.906738281	1.001424057	1		0.919507027	1		1.050079962	1		1.118627107	1		0.960864494	1		0.953922059	1		Quantified
P86048	60S ribosomal protein L10-like	Rpl10l		60S ribosomal protein L10-l	3.74	1	1	1	214	24.54191744	10.11083984	0.88483833	1		0.935262662	1		0.94851686	1		1.026434739	1		0.987396253	1		1.252024731	1		Quantified
Q8C854	Myelin expression factor 2	Myef2	FUNCTION: Transcriptional repressor of the r	Myelin expression factor 2 (	1.35	1	1	1	591	63.25425929	8.865722656	1.252695774	1		0.952718574	1		0.956837914	1		0.824566678	1		0.818057401	1		1.14732604	1		Quantified
Q8BGC0	HIV Tat-specific factor 1 homolog	Htatsf1	FUNCTION: Functions as a general transcript	HIV Tat-specific factor 1 ho	1.98	2	2	2	757	86.18755235	4.398925781	1.005745881	2	2.39276501	0.997786686	2	21.10517719	0.930358844	2	0.776593714	0.972848315	2	2.191439519	1.139360955	2	21.60821014	1.022034987	2	4.439979876	Quantified
Q61136	Serine/threonine-protein kinase PRP4 homolog	Prpf4b	FUNCTION: Has a role in pre-mRNA splicing.	Serine/threonine-protein kir	0.79	1	1	1	1007	116.9044823	10.22802734	0.958332464	1		1.010100672	1		0.935311226	1		1.063088516	1		1.028242554	1		1.045697351	1		Quantified
A2AVA0	Sushi, von Willebrand factor type A, EGF and pentra	Svep1	FUNCTION: May play a role in the cell attach	Sushi, von Willebrand facto	0.45	2	2	4	3567	387.2022643	5.566894531	1.024556347	4	2.913750405	1.025037782	4	1.597426211	0.983998081	4	3.516204997	1.003941473	4	4.536669445	0.985728513	4	0.3128091	0.986885743	4	2.990198141	Quantified
P97372	Proteasome activator complex subunit 2	Psme2	FUNCTION: Implicated in immunoproteasom	Proteasome activator comp	14.64	4	4	6	239	27.04013548	5.757324219	1.02219907	6	3.201708985	0.997811344	6	4.060863828	1.012430542	6	2.423729314	0.97123415	6	2.982568405	1.057974933	6	7.6927484	0.990363528	6	2.564977958	Quantified
Q810U4	Neuronal cell adhesion molecule	Nrcam	FUNCTION: Cell adhesion protein that is requ	Neuronal cell adhesion mol	1.04	1	1	1	1256	138.4349204	5.909667969	0.939802133	1		0.936560243	1		1.043177966	1		0.978113745	1		1.135663297	1		1.045681699	1		Quantified
Q9CYG7	Mitochondrial import receptor subunit TOM34	Tomm34	FUNCTION: Plays a role in the import of cyto	Mitochondrial import recept	4.85	1	1	2	309	34.25675308	9.144042969	0.996259203	2	2.538580853	1.037433165	2	2.191859969	0.95417702	2	0.054273371	0.968438517	2	0.270674468	0.953073175	2	6.739111689	1.095154857	2	3.341786229	Quantified
Q9DCL9	Multifunctional protein ADE2	Paics		Multifunctional protein ADE	10.35	5	5	6	425	46.97606111	7.225097656	1.005393484	6	6.430411052	0.994874703	6	3.627781173	1.004922735	6	9.250573477	1.031039861	6	8.054702261	1.041242134	6	8.747978997	0.980940945	6	4.039689005	Quantified
P19437	B-lymphocyte antigen CD20	Ms4a1	FUNCTION: B-lymphocyte-specific membran	B-lymphocyte antigen CD2(	3.78	1	1	1	291	31.937481	5.224121094	0.958767744	1		0.948387207	1		0.976225648	1		1.174130538	1		0.973666217	1		0.986234019	1		Quantified
Q3UQN2	F-BAR domain only protein 2	Fcho2	FUNCTION: Functions in an early step of clat	F-BAR domain only protein	1.98	2	2	2	809	88.6790863	6.888183594	0.899650036	2	21.17731988	0.926905529	2	3.384485926	0.925137817	2	5.170185424	1.112591555	2	17.00624908	1.079737377	2	5.47918126	1.110092889	2	5.277771873	Quantified
P35564	Calnexin	Canx	FUNCTION: Calcium-binding protein that inte	Calnexin OS=Mus musculu	2.71	2	2	2	591	67.23574461	4.640136719	0.963777239	2	7.156007936	0.918068435	2	1.415345864	0.951185475	2	0.602470869	1.046136512	2	3.88961435	1.033734938	2	7.270688351	1.135269121	2	0.611790505	Quantified
Q9CZD3	Glycine--tRNA ligase	Gars1	FUNCTION: Catalyzes the ATP-dependent lig	Glycine--tRNA ligase OS=M	7.27	6	6	8	729	81.82573659	6.653808594	0.993228975	8	4.386565755	0.991668539	8	1.763256283	0.990008129	8	5.911395323	1.020027127	8	2.320587601	0.979421943	8	6.676911337	1.002099411	8	4.555421915	Quantified
Q62376	U1 small nuclear ribonucleoprotein 70 kDa	Snrnp70	FUNCTION: Component of the spliceosomal	U1 small nuclear ribonucle	2.01	1	1	1	448	51.96091697	9.935058594	1.014131898	1		1.042994031	1		1.067881347	1		0.978931108	1		0.952067349	1		0.931950907	1		Quantified
Q8BND5	Sulfhydryl oxidase 1	Qsox1	FUNCTION: Catalyzes the oxidation of sulfhy	Sulfhydryl oxidase 1 OS=M	1.07	1	1	1	748	82.73301286	7.166503906	0.996611586	1		0.996121196	1		0.920446057	1		1.074393874	1		1.011698425	1		1.036511384	1		Quantified
O08759	Ubiquitin-protein ligase E3A	Ube3a	FUNCTION: E3 ubiquitin-protein ligase which	Ubiquitin-protein ligase E3	1.15	1	1	1	870	99.75571831	5.084472656	1.008646248	1		0.88231817	1		0.966395681	1		1.06509476	1		1.12185321	1		1.04062667	1		Quantified
Q9D1J3	SAP domain-containing ribonucleoprotein	Sarnp	FUNCTION: Binds both single-stranded and c	SAP domain-containing ribc	4.29	1	1	1	210	23.51833366	6.653808594	1.042770906	1		1.109170313	1		0.881599765	1		0.850839666	1		0.863445831	1		1.2251298	1		Quantified
P63037	DnaJ homolog subfamily A member 1	Dnaja1	FUNCTION: Co-chaperone for HSPA8/Hsc70	DnaJ homolog subfamily A	2.02	1	1	1	397	44.83945466	7.078613281	1.019596299	1		1.019791238	1		0.951797506	1		0.989254257	1		0.989150085	1		1.051195797	1		Quantified
P00688	Pancreatic alpha-amylase	Amy2		Pancreatic alpha-amylase (	2.36	1	1	1	508	57.28180448	7.283691406	0.965791863	1		0.846259031	1		0.922117246	1		1.142971319	1		1.117127449	1		1.09889526	1		Quantified
P70232	Neural cell adhesion molecule L1-like protein	Chl1	FUNCTION: Extracellular matrix and cell adh	Neural cell adhesion molec	0.58	1	1	1	1209	134.9898091	5.566894531	0.926809996	1		1.114626963	1		1.022983236	1		0.859923884	1		1.234410217	1		0.946882267	1		Quantified
Q8BY89	Choline transporter-like protein 2	Slc44a2	FUNCTION: Choline transporter. {ECO:00002	Choline transporter-like pro	2.41	1	1	1	706	80.05674459	8.792480469	0.974695232	1		1.026819473	1		1.250988839	1		0.863911398	1		0.879759973	1		0.943461668	1		Quantified
Q5XJY5	Coatomer subunit delta	Arcn1	FUNCTION: The coatomer is a cytosolic prot	Coatomer subunit delta OS	8.02	4	4	4	511	57.19320317	6.214355469	1.034286319	4	1.539838166	0.977544503	4	1.362553617	1.011602523	4	6.831109336	1.003722719	4	5.908169262	0.975913807	4	2.964864667	0.963698024	4	1.775707359	Quantified
P59708	Splicing factor 3B subunit 6	Sf3b6	FUNCTION: Involved in pre-mRNA splicing a	Splicing factor 3B subunit 6	11.2	1	1	1	125	14.57555229	9.378417969	0.952699193	1		0.919919451	1		0.948600802	1		1.108448907	1		1.146451502	1		1.018672932	1		Quantified
P63028	Translationally-controlled tumor protein	Tpt1	FUNCTION: Involved in calcium binding and	Translationally-controlled tu	7.56	1	1	1	172	19.44955017	4.855957031	1.058904113	1		1.093548354	1		1.087746343	1		0.836533725	1		0.895302717	1		0.986324614	1		Quantified
Q8BWG8	Beta-arrestin-1	Arrb1	FUNCTION: Functions in regulating agonist-n	Beta-arrestin-1 OS=Mus mu	4.55	1	1	1	418	46.94318953	6.277832031	1.009865358	1		1.048856312	1		1.014687827	1		0.993035692	1		0.937264032	1		0.984979138	1		Quantified
P97311	DNA replication licensing factor MCM6	Mcm6	FUNCTION: Acts as component of the MCM2	DNA replication licensing fa	2.44	2	2	2	821	92.80899999	5.452636719	0.996292058	2	5.663053808	1.003792343	2	13.5858083	1.00910448	2	2.552771576	1.070774106	2	20.06671968	1.026968418	2	13.1981416	0.903219212	2	6.741515222	Quantified
Q5SW19	Clustered mitochondria protein homolog	Cluh	FUNCTION: mRNA-binding protein involved i	Clustered mitochondria prot	0.61	1	1	1	1315	147.9747897	6.023925781	1.02085267	1		1.019491917	1		1.066360146	1		0.932856897	1		0.994002193	1		0.974476603	1		Quantified
Q61768	Kinesin-1 heavy chain	Kif5b	FUNCTION: Microtubule-dependent motor re	Kinesin-1 heavy chain OS=	2.8	3	3	3	963	109.4836801	6.442871094	1.033273404	3	8.386741243	0.971193984	3	1.256525173	1.018166274	3	0.457986263	1.097283214	3	7.490822425	1.034505549	3	2.187590996	0.923608017	3	0.048935493	Quantified
Q60870	Receptor expression-enhancing protein 5	Reep5	FUNCTION: May promote functional cell surf	Receptor expression-enhar	5.41	2	2	2	185	21.03688027	8.382324219	0.931608459	2	7.775212728	0.985842335	2	4.587978136	0.729723016	2	6.25409334	1.155324864	2	11.680851	1.141602746	2	6.607066497	1.165164134	2	0.466204887	Quantified
Q08481	Platelet endothelial cell adhesion molecule	Pecam1	FUNCTION: Cell adhesion molecule which is	Platelet endothelial cell adh	1.79	1	1	1	727	81.21109954	6.712402344	0.946933131	1		1.05860728	1		1.158051957	1		0.93530164	1		0.968356211	1		0.916951122	1		Quantified
P63280	SUMO-conjugating enzyme UBC9	Ube2i	FUNCTION: Accepts the ubiquitin-like protein	SUMO-conjugating enzyme	4.43	1	1	1	158	17.99519966	8.660644531																			Identified
P62918	60S ribosomal protein L8	Rpl8	FUNCTION: Component of the large ribosom	60S ribosomal protein L8 O	9.34	2	2	2	257	28.00729016	11.03369141	0.911100743	2	10.98127197	0.956280948	2	4.648535858	1.020841414	2	5.639735532	1.009594424	2	7.05425704	1.167160457	2	20.99357343	1.014019311	2	9.704796812	Quantified
Q9R0E1	Multifunctional procollagen lysine hydroxylase and gl	Plod3	FUNCTION: Multifunctional enzyme that cata	Multifunctional procollagen	1.35	1	1	1	741	84.8686397	6.227050781	1.012541437	1		0.987522632	1		0.957777187	1		0.988778869	1		1.07148904	1		1.03838224	1		Quantified
Q9WUU7	Cathepsin Z	Ctsz	FUNCTION: Exhibits carboxy-monopeptidase	Cathepsin Z OS=Mus musc	4.58	1	1	1	306	33.97418147	6.595214844	1.028979101	1		0.909619829	1		1.138116491	1		1.002526788	1		0.95566173	1		0.957566037	1		Quantified
Q9DCT8	Cysteine-rich protein 2	Crip2		Cysteine-rich protein 2 OS=	3.37	1	1	1	208	22.71205944	8.631347656	1.02876375	1		0.968497605	1		1.024078277	1		1.055454205	1		1.117145719	1		0.871443563	1		Quantified
Q8BRK8	5'-AMP-activated protein kinase catalytic subunit alp	Prkaa2	FUNCTION: Catalytic subunit of AMP-activat	5'-AMP-activated protein ki	1.81	1	1	1	552	61.98243757	7.869628906	1.074254962	1		0.986894111	1		0.938347897	1		0.952921802	1		1.034877437	1		1.055419003	1		Quantified
Q8R2Y2	Cell surface glycoprotein MUC18	Mcam	FUNCTION: Plays a role in cell adhesion, an	Cell surface glycoprotein M	2.78	1	1	1	648	71.5005073	5.833496094	1.009701432	1		0.93816981	1		0.926562377	1		1.152393773	1		1.01937933	1		0.995620679	1		Quantified
Q8CB27	Ubiquitin thioesterase OTU1	Yod1	FUNCTION: Hydrolase that can remove conj	Ubiquitin thioesterase OTU	2.62	1	1	1	343	37.46186751	5.757324219	1.002980541	1		1.008078434	1		1.052078258	1		0.981795248	1		1.006166541	1		0.964700758	1		Quantified
Q9JMH9	Unconventional myosin-XVIIIa	Myo18a	FUNCTION: May link Golgi membranes to the	Unconventional myosin-XV	0.88	2	2	2	2050	232.610836	6.277832031	0.970260095	2	9.827564473	0.928834531	2	11.27613604	0.964648329	2	10.8569747	0.957424298	2	4.686271038	0.902356011	2	7.388815484	1.231864646	2	34.75764497	Quantified
Q8K2V1	Serine/threonine-protein phosphatase 4 regulatory su	Ppp4r1	FUNCTION: Regulatory subunit of serine/thre	Serine/threonine-protein ph	1.05	1	1	1	951	106.2303512	4.805175781	1.066744754	1		1.123365286	1		0.952320105	1		0.965980522	1		0.87104037	1		0.981757054	1		Quantified
P36993	Protein phosphatase 1B	Ppm1b	FUNCTION: Enzyme with a broad specificity.	Protein phosphatase 1B OS	2.05	1	1	1	390	42.76764378	5.186035156	1.035962255	1		0.939207163	1		1.072587235	1		0.960835331	1		1.012330205	1		1.000709395	1		Quantified
Q8VCT4	Carboxylesterase 1D	Ces1d	FUNCTION: Major lipase in white adipose tis	Carboxylesterase 1D OS=M	6.19	3	3	4	565	61.74870096	6.609863281	1.020110733	4	7.108723014	1.020002793	4	3.728187241	1.018027989	4	5.730822759	0.914251355	4	4.839055414	0.998749453	4	5.632447316	1.008316574	4	2.29596737	Quantified
Q8CIN4	Serine/threonine-protein kinase PAK 2	Pak2	FUNCTION: Serine/threonine protein kinase t	Serine/threonine-protein kir	3.63	2	2	2	524	57.89378832	5.770019531	0.985042456	2	3.856236342	0.971730688	2	2.581988796	1.050209695	2	6.041822948	1.042116767	2	4.820245386	1.036452048	2	5.675551708	0.943198642	2	2.794962052	Quantified
Q9ESX5	H/ACA ribonucleoprotein complex subunit DKC1	Dkc1	FUNCTION: Catalytic subunit of H/ACA small	H/ACA ribonucleoprotein cc	2.75	2	2	2	509	57.36531312	9.275878906	0.961585599	2	0.655323869	1.029000903	2	0.905050177	1.01645211	2	3.380741512	1.020638635	2	1.65794604	0.962276516	2	5.736861007	1.010071087	2	7.120900159	Quantified
Q9QXK3	Coatomer subunit gamma-2	Copg2	FUNCTION: The coatomer is a cytosolic prot	Coatomer subunit gamma-2	1.95	1	1	1	871	97.61749204	5.795410156	1.049825609	1		0.850692369	1		1.010439899	1		1.0563796	1		0.937017895	1		1.10245988	1		Quantified
Q91WE2	PSME3-interacting protein	Psme3ip1	FUNCTION: Promotes the association of the	PSME3-interacting protein (	3.54	1	1	1	254	28.68429871	5.135253906	0.880058357	1		0.891207601	1		1.072949616	1		1.189078443	1		1.274917017	1		0.825060383	1		Quantified
Q8VDF3	Death-associated protein kinase 2	Dapk2	FUNCTION: Calcium/calmodulin-dependent s	Death-associated protein ki	5.41	1	1	1	370	42.7513269	7.283691406	0.928862841	1		0.998778092	1		1.016189991	1		1.014884661	1		0.994779469	1		1.066234308	1		Quantified
Q9Z1G3	V-type proton ATPase subunit C 1	Atp6v1c1	FUNCTION: Subunit of the peripheral V1 con	V-type proton ATPase subu	5.24	2	2	2	382	43.85993468	7.459472656	0.933045821	2	2.652695116	1.011545264	2	2.572431381	1.019143241	2	14.4480564	1.017802846	2	8.385673966	1.051924867	2	2.595554709	0.999516381	2	0.898700778	Quantified
P36536	GTP-binding protein SAR1a	Sar1a	FUNCTION: Involved in transport from the en	GTP-binding protein SAR1a	4.04	1	1	3	198	22.35652898	6.932128906	0.986928883	3	6.049192667	0.979082478	3	0.937248309	0.9784544	3	3.837661245	1.00553059	3	0.202273092	1.142076507	3	5.607205814	1.000545282	3	0.060806758	Quantified
Q9CSU0	Regulation of nuclear pre-mRNA domain-containing p	Rprd1b	FUNCTION: Interacts with phosphorylated C-	Regulation of nuclear pre-m	4.91	1	1	1	326	36.86119228	5.973144531	1.015883395	1		0.898320648	1		0.799775245	1		1.189354299	1		1.250453828	1		1.000863566	1		Quantified
Q60790	Ras GTPase-activating protein 3	Rasa3	FUNCTION: Inhibitory regulator of the Ras-cy	Ras GTPase-activating prot	1.56	2	2	2	834	95.92643529	7.342285156	1.216396614	2	40.29476573	0.928496218	2	15.84993583	0.902176359	2	32.53608069	1.006757626	2	8.587842438	0.986116139	2	0.942291156	0.909686315	2	11.20293648	Quantified
O54962	Barrier-to-autointegration factor	Banf1	FUNCTION: Plays fundamental roles in nucle	Barrier-to-autointegration fa	16.85	3	3	3	89	10.09602513	6.087402344	0.721193022	3	2.344638361	0.652517106	3	0.146702274	0.892188587	3	13.8232791	1.304927899	3	14.8159135	1.582616834	3	0.171989354	1.068660436	3	0.217863898	Quantified
P31725	Protein S100-A9	S100a9	FUNCTION: S100A9 is a calcium- and zinc-b	Protein S100-A9 OS=Mus r	15.04	3	3	6	113	13.04028311	7.166503906	0.906189088	6	7.918516395	0.906215427	6	8.253901302	0.930776206	6	8.617803492	1.157017241	6	5.785510663	1.260058987	6	10.30040546	0.926034611	6	2.359836714	Quantified
Q9D0F3	Protein ERGIC-53	Lman1	FUNCTION: Mannose-specific lectin. May rec	Protein ERGIC-53 OS=Mus	1.16	1	1	2	517	57.75286317	6.341308594	0.956813962	2	9.032292978	0.991530761	2	8.649170809	1.134052871	2	11.36166314	0.928367264	2	7.832024338	1.080320133	2	6.763233249	0.93722075	2	7.327696778	Quantified
Q8QZY9	Splicing factor 3B subunit 4	Sf3b4	FUNCTION: Involved in pre-mRNA splicing a	Splicing factor 3B subunit 4	3.07	2	2	2	424	44.327109	8.558105469	0.937885139	2	9.324167733	1.013618327	2	4.499061731	1.041641925	2	0.029366481	0.9169351	2	0.344243302	1.041706132	2	24.78554548	1.072304839	2	0.796555956	Quantified
Q8BKC5	Importin-5	Ipo5	FUNCTION: Functions in nuclear protein imp	Importin-5 OS=Mus muscul	2.73	3	3	3	1097	123.5108617	4.932128906	0.985117576	3	5.446341438	0.980039179	3	8.519874334	1.020700902	3	12.50069171	0.978207657	3	4.339744074	0.949337432	3	26.61323919	0.947612249	3	0.781370655	Quantified
P48193	Protein 4.1	Epb41	FUNCTION: Protein 4.1 is a major structural	Protein 4.1 OS=Mus muscu	0.7	1	1	1	858	95.85254208	5.604980469	1.062980172	1		1.037402318	1		0.972108678	1		1.017453919	1		0.966158763	1		0.948361929	1		Quantified
O35744	Chitinase-like protein 3	Chil3	FUNCTION: Lectin that binds saccharides wit	Chitinase-like protein 3 OS	2.01	1	1	2	398	44.43014748	5.693847656	0.687836014	2	17.83587448	0.680807395	2	17.26412766	0.672621687	2	12.44332367	1.341700016	2	4.061082563	1.492073521	2	18.29254685	1.404494042	2	9.467319875	Quantified
Q9QZ88	Vacuolar protein sorting-associated protein 29	Vps29	FUNCTION: Acts as component of the retrom	Vacuolar protein sorting-as	12.09	2	2	2	182	20.48265919	6.785644531	1.005879851	2	2.34022342	1.050536169	2	0.478826442	0.950239661	2	1.745802492	1.001594616	2	5.932766807	0.954620453	2	5.818187355	1.039317442	2	5.403286725	Quantified
P46467	Vacuolar protein sorting-associated protein 4B	Vps4b	FUNCTION: Involved in late steps of the endc	Vacuolar protein sorting-as	4.28	2	2	3	444	49.38854146	7.107910156	1.007358578	3	1.114445097	1.015597832	3	0.9298245	0.936472014	3	2.323763341	0.971429307	3	6.571980045	1.038648341	3	0.943882539	1.046233035	3	7.386046891	Quantified
P54116	Stomatin	Stom	FUNCTION: Regulates ion channel activity ar	Stomatin OS=Mus musculu	3.52	1	1	2	284	31.35547984	6.932128906	0.992610808	2	2.181358229	0.869730369	2	3.624925039	1.025991339	2	2.142069655	1.126448594	2	1.501425534	1.075176971	2	0.559342071	0.967344285	2	6.366550621	Quantified
P26516	26S proteasome non-ATPase regulatory subunit 7	Psmd7	FUNCTION: Component of the 26S proteasor	26S proteasome non-ATPa	6.85	2	2	2	321	36.51721417	6.770996094	0.990061947	2	3.330448873	0.982303811	2	2.988241541	0.981171029	2	3.315504036	1.003044414	2	1.977175155	1.070243632	2	2.493412349	1.02520028	2	6.594803157	Quantified
Q9Z1R2	Large proline-rich protein BAG6	Bag6	FUNCTION: ATP-independent molecular cha	Large proline-rich protein B.	1.65	2	2	2	1154	120.9617175	5.706542969	0.97865374	2	7.249857938	0.941117343	2	1.462654065	0.948077738	2	2.102687865	0.984981009	2	5.201477585	1.203515831	2	21.83223696	1.050578078	2	3.60649983	Quantified
P97315	Cysteine and glycine-rich protein 1	Csrp1	FUNCTION: Could play a role in neuronal dev	Cysteine and glycine-rich p	36.79	4	4	6	193	20.56975862	8.572753906	0.995907526	6	4.026732578	1.017104732	6	9.413025149	1.08903641	6	13.89330342	0.965013367	6	11.79152809	0.947509991	6	9.538894075	0.968412762	6	6.834874421	Quantified
P56528	ADP-ribosyl cyclase/cyclic ADP-ribose hydrolase 1	Cd38	FUNCTION: Synthesizes the second messen	ADP-ribosyl cyclase/cyclic	9.21	3	3	4	304	34.38532032	8.294433594	1.012467447	4	0.290969834	0.953412531	4	6.710020804	1.090219168	4	3.408361776	0.983816093	4	7.435637863	0.902522609	4	3.626469698	1.042910817	4	2.768325037	Quantified
Q3TCH7	Cullin-4A	Cul4a	FUNCTION: Core component of multiple culli	Cullin-4A OS=Mus musculu	1.58	1	1	1	759	87.69745752	8.353027344	0.950840206	1		0.906921545	1		1.019828217	1		1.108011382	1		0.957557144	1		1.067886359	1		Quantified
Q9CZX8	40S ribosomal protein S19	Rps19	FUNCTION: Required for pre-rRNA processin	40S ribosomal protein S19	6.21	1	1	1	145	16.07559645	10.40380859	1.008466148	1		0.909798898	1		1.076095796	1		1.089462114	1		1.056524674	1		0.899958731	1		Quantified
Q6PD03	Serine/threonine-protein phosphatase 2A 56 kDa reg	Ppp2r5a	FUNCTION: The B regulatory subunit might n	Serine/threonine-protein ph	5.14	4	4	4	486	56.31124184	7.034667969	0.989046492	4	3.724558763	1.027302649	4	3.188370411	0.968775839	4	1.134488893	0.983713491	4	3.72316436	0.976620415	4	6.754157279	1.021453473	4	2.34338705	Quantified
P50518	V-type proton ATPase subunit E 1	Atp6v1e1	FUNCTION: Subunit of the peripheral V1 con	V-type proton ATPase subu	9.29	2	2	2	226	26.14083412	8.426269531	0.978643109	2	3.175112011	0.93572672	2	2.581677955	0.934477981	2	1.188707453	1.117073772	2	1.848458135	1.015557312	2	12.67821561	1.057212327	2	2.509116971	Quantified
P59325	Eukaryotic translation initiation factor 5	Eif5	FUNCTION: Catalyzes the hydrolysis of GTP	Eukaryotic translation initiat	9.79	4	4	5	429	48.93772371	5.516113281	1.030782703	5	8.141760861	0.961253054	5	6.964709585	1.055409723	5	6.574744853	1.032058657	5	6.788413296	1.02464104	5	11.86697465	0.964512411	5	5.875631139	Quantified
Q9JHJ0	Tropomodulin-3	Tmod3	FUNCTION: Blocks the elongation and depol	Tropomodulin-3 OS=Mus m	3.41	1	1	1	352	39.4783345	5.135253906	1.007548142	1		0.982163473	1		0.994941825	1		1.011273716	1		1.101616541	1		0.966117858	1		Quantified
P49443	Protein phosphatase 1A	Ppm1a	FUNCTION: Enzyme with a broad specificity.	Protein phosphatase 1A OS	4.97	2	2	2	382	42.40573918	5.363769531	1.064275025	2	3.38151421	0.995877572	2	7.591827893	1.002262517	2	2.04656585	0.98391198	2	0.724826278	1.014249595	2	8.28581743	0.961070192	2	1.195023825	Quantified
P16330	2',3'-cyclic-nucleotide 3'-phosphodiesterase	Cnp	FUNCTION: May participate in RNA metaboli	2',3'-cyclic-nucleotide 3'-ph	6.43	2	2	3	420	47.09353846	8.968261719	1.069374091	3	8.364589604	0.91135158	3	12.32533056	1.142659897	3	0.578850935	0.941855022	3	4.641380319	1.044153403	3	7.877320654	0.999589898	3	0.197676289	Quantified
Q3SXB8	Collectin-11	Colec11	FUNCTION: Lectin that plays a role in innate	Collectin-11 OS=Mus musc	2.94	1	1	1	272	28.9701297	5.122558594	0.847584215	1		0.930324351	1		0.873165577	1		1.181933319	1		1.005021874	1		1.212731931	1		Quantified
Q9WTZ2	Membrane-bound transcription factor site-1 protease	Mbtps1	FUNCTION: Serine protease that catalyzes th	Membrane-bound transcript	1.33	2	2	2	1052	117.3828738	8.763183594	1.004407703	2	1.163344258	0.968476629	2	0.897682822	0.989946637	2	4.509359358	1.049914646	2	3.199304041	1.008098678	2	7.24390391	1.005404504	2	0.553087073	Quantified
O35685	Nuclear migration protein nudC	Nudc	FUNCTION: Plays a role in neurogenesis and	Nuclear migration protein n	5.12	3	3	3	332	38.33429108	5.262207031	1.018882947	3	2.119436893	1.061620384	3	1.541985959	0.988352957	3	2.140641436	0.951336917	3	0.996921429	0.976211174	3	17.09098731	1.045466512	3	19.64680227	Quantified
Q9CPX6	Ubiquitin-like-conjugating enzyme ATG3	Atg3	FUNCTION: E2 conjugating enzyme required	Ubiquitin-like-conjugating e	1.91	1	1	1	314	35.77340419	4.716308594	1.019133761	1		0.967047361	1		1.077407333	1		0.945152925	1		0.972363783	1		1.022075465	1		Quantified
Q9Z1X4	Interleukin enhancer-binding factor 3	Ilf3	FUNCTION: RNA-binding protein that plays a	Interleukin enhancer-bindin	2.23	2	2	3	898	95.96135106	8.763183594	1.052830829	3	1.386295284	1.041386688	3	2.193174448	1.035343375	3	0.568616143	0.945378922	3	4.187671594	1.019885896	3	0.219545567	0.989516797	3	2.66412092	Quantified
O89020	Afamin	Afm	FUNCTION: Functions as carrier for hydrophc	Afamin OS=Mus musculus	2.3	2	2	2	608	69.33376465	5.782714844	0.950915047	2	5.368235163	0.995548127	2	0.806638519	1.047091153	2	6.303566507	1.007292694	2	2.895410023	1.012855171	2	0.075977674	1.005743508	2	5.972509575	Quantified
Q99KB8	Hydroxyacylglutathione hydrolase, mitochondrial	Hagh	FUNCTION: Thiolesterase that catalyzes the	Hydroxyacylglutathione hyc	7.44	2	2	2	309	34.06245512	7.752441406	0.955737976	2	1.658853001	1.012973561	2	2.26561037	0.958670154	2	2.361711212	1.002411892	2	7.153813758	1.079597234	2	3.949000692	1.047783618	2	2.919233293	Quantified
P27048	Small nuclear ribonucleoprotein-associated protein B	Snrpb	FUNCTION: Plays role in pre-mRNA splicing	Small nuclear ribonucleopro	9.96	2	2	2	231	23.64022285	10.90185547	1.003127878	2	0.84852204	0.929715954	2	5.062531433	1.008227223	2	5.236002717	1.025748284	2	2.746718059	1.091833309	2	0.528057273	1.002901011	2	3.137043126	Quantified
P55302	Alpha-2-macroglobulin receptor-associated protein	Lrpap1	FUNCTION: Molecular chaperone for LDL rec	Alpha-2-macroglobulin rece	4.72	2	2	2	360	42.18916481	7.869628906	0.942675158	2	2.52520519	0.985077073	2	4.366333077	0.914406718	2	5.890940601	1.097631741	2	6.396255162	1.044347365	2	1.618302509	1.066002467	2	0.199714626	Quantified
Q9CXW4	60S ribosomal protein L11	Rpl11	FUNCTION: Component of the ribosome, a la	60S ribosomal protein L11 (	6.74	1	1	1	178	20.23964104	9.598144531	0.974175854	1		0.872270041	1		1.17631545	1		1.016919346	1		1.081322531	1		0.922290225	1		Quantified
Q60865	Caprin-1	Caprin1	FUNCTION: May regulate the transport and tr	Caprin-1 OS=Mus musculu	2.4	2	2	2	707	78.12080542	5.249511719	0.970886482	2	9.605364767	1.004238414	2	2.182345816	1.189661259	2	6.450950105	0.959098458	2	8.000859921	0.990200607	2	1.374629146	0.870972782	2	11.6011258	Quantified
Q3UDE2	Tubulin--tyrosine ligase-like protein 12	Ttll12	FUNCTION: Negatively regulates post-transla	Tubulin--tyrosine ligase-like	1.56	1	1	1	639	73.9957909	5.630371094	1.030279727	1		1.01505543	1		0.878171707	1		1.098607313	1		0.979874718	1		1.022986118	1		Quantified
Q61686	Chromobox protein homolog 5	Cbx5	FUNCTION: Component of heterochromatin t	Chromobox protein homolo	6.81	1	1	1	191	22.17199017	5.858886719	0.927391813	1		1.008541547	1		1.041090638	1		1.048270119	1		1.086461775	1		0.93942352	1		Quantified
Q9JJW0	Peroxisomal membrane protein 4	Pxmp4		Peroxisomal membrane prc	2.83	1	1	1	212	24.18076976	9.832519531																			Identified
O08529	Calpain-2 catalytic subunit	Capn2	FUNCTION: Calcium-regulated non-lysosoma	Calpain-2 catalytic subunit	5.43	3	4	5	700	79.82177162	4.957519531	0.981768207	4	0.964958852	0.999858368	4	1.920658482	1.012317064	4	6.225992036	0.9533495	4	4.246154165	1.01031906	4	2.641336407	1.030092402	4	2.574686438	Quantified
P30115	Glutathione S-transferase A3	Gsta3	FUNCTION: Conjugation of reduced glutathio	Glutathione S-transferase A	4.07	2	2	2	221	25.3442663	8.733886719	0.938904344	2	2.055614628	0.990632067	2	10.32506324	0.971060971	2	10.3985789	1.109728236	2	16.21933684	1.102093248	2	2.446757099	0.942502039	2	0.866657266	Quantified
P97812	Indian hedgehog protein	Ihh	FUNCTION: Intercellular signal essential for a	Indian hedgehog protein OS	3.16	2	2	2	411	45.45639181	8.602050781	0.961065345	2	6.336713357	0.958950491	2	1.622460851	0.978345515	2	0.266233253	1.042001878	2	7.875988734	1.06521098	2	7.398664016	1.045776754	2	5.101544867	Quantified
Q9CQC9	GTP-binding protein SAR1b	Sar1b	FUNCTION: Involved in transport from the en	GTP-binding protein SAR1b	5.56	1	1	2	198	22.36747623	6.112792969	0.990287028	2	4.497442179	0.998334279	2	0.834553569	1.04290055	2	3.822850684	0.981306815	2	3.256992416	0.95942711	2	2.804089037	1.026391395	2	2.394975796	Quantified
Q9EPX2	Papilin	Papln		Papilin OS=Mus musculus	0.63	1	1	2	1280	138.8156389	7.752441406	1.049232483	2	2.210416263	0.990892795	2	1.876185656	1.008615097	2	2.206744646	1.001897765	2	0.799191159	0.970735758	2	2.017753485	0.985256125	2	0.055912171	Quantified
Q8BU30	Isoleucine--tRNA ligase, cytoplasmic	Iars1	FUNCTION: Catalyzes the specific attachmer	Isoleucine--tRNA ligase, cy	1.35	2	2	2	1262	144.1789483	6.551269531	1.069659983	2	1.38477338	0.990624998	2	8.790254623	1.019603593	2	2.18115027	1.014582	2	12.71243449	0.962945261	2	5.795716423	0.934624373	2	11.75538264	Quantified
Q8BP47	Asparagine--tRNA ligase, cytoplasmic	NARS1	FUNCTION: Catalyzes the attachment of asp	Asparagine--tRNA ligase, c	3.76	2	2	2	559	64.23801299	5.858886719	0.954112392	2	4.727071647	0.989020013	2	2.244006996	0.97072071	2	5.492333922	0.986388601	2	3.351496823	0.956422482	2	15.02534878	1.13738585	2	22.34979567	Quantified
P52431	DNA polymerase delta catalytic subunit	Pold1	FUNCTION: As the catalytic component of th	DNA polymerase delta cata	1.81	2	2	2	1105	123.7119334	7.605957031	1.051931442	2	4.67078626	0.993844573	2	4.671651077	1.03294888	2	0.601769303	0.94397938	2	4.195419091	0.962432464	2	6.684632528	1.013895919	2	1.614408579	Quantified
Q3TC72	Fumarylacetoacetate hydrolase domain-containing pr	Fahd2	FUNCTION: May have hydrolase activity. {EC	Fumarylacetoacetate hydro	8.31	2	2	2	313	34.66809037	8.162597656	0.998020968	2	6.651070245	0.99086258	2	1.688034127	1.027864648	2	0.171323846	0.995723853	2	0.993813577	0.95456522	2	6.241521815	1.031257187	2	3.859872925	Quantified
Q9CY64	Biliverdin reductase A	Blvra	FUNCTION: Reduces the gamma-methene b	Biliverdin reductase A OS=	3.05	1	1	1	295	33.50352457	7.020019531	1.036994314	1		1.037344939	1		0.962765893	1		0.943406757	1		1.092458833	1		0.985881149	1		Quantified
Q640N1	Adipocyte enhancer-binding protein 1	Aebp1	FUNCTION: [Isoform 1]: As a positive regulat	Adipocyte enhancer-binding	3.63	3	3	3	1128	128.2844921	5.084472656	1.014127152	3	5.72412643	0.995066689	3	0.152584605	0.970975338	3	0.460429108	0.942080871	3	6.869816066	1.026160315	3	7.401480733	1.038607344	3	7.706099488	Quantified
Q61830	Macrophage mannose receptor 1	Mrc1	FUNCTION: Mediates the endocytosis of glyc	Macrophage mannose rece	0.48	1	1	1	1456	164.8744111	6.829589844	1.069639432	1		1.021165831	1		1.079074248	1		0.96748142	1		0.91282886	1		0.920672247	1		Quantified
Q8VDG3	Poly(A)-specific ribonuclease PARN	Parn	FUNCTION: 3'-exoribonuclease that has a pr	Poly(A)-specific ribonucleas	2.08	1	1	1	624	71.51389171	5.973144531	1.038009315	1		0.897776181	1		0.992303772	1		1.022870166	1		1.099477055	1		1.019988623	1		Quantified
Q80UM3	N-alpha-acetyltransferase 15, NatA auxiliary subunit	Naa15	FUNCTION: Auxillary subunit of the N-termin	N-alpha-acetyltransferase 1	1.85	2	2	3	865	100.8965639	7.620605469	1.06477642	3	0.045705907	1.063377559	3	1.295907041	0.998066676	3	2.698780305	0.947864853	3	6.461847637	0.952318616	3	8.939052098	1.017081143	3	3.634554285	Quantified
Q9CQT1	Methylthioribose-1-phosphate isomerase	Mri1	FUNCTION: Catalyzes the interconversion of	Methylthioribose-1-phospha	6.23	2	2	3	369	39.38635164	5.909667969	0.992904965	3	3.940891369	1.014898244	3	0.270203502	0.977930507	3	10.6960211	1.018438481	3	10.24783856	0.992970146	3	19.17100996	0.997093207	3	4.35029947	Quantified
Q9DB34	Charged multivesicular body protein 2a	Chmp2a	FUNCTION: Probable core component of the	Charged multivesicular bod	8.11	1	1	1	222	25.11786746	5.973144531	1.076910393	1		0.867091443	1		1.027772177	1		0.945868317	1		1.061950977	1		1.074109199	1		Quantified
Q8CCJ3	E3 UFM1-protein ligase 1	Ufl1	FUNCTION: E3 protein ligase that mediates u	E3 UFM1-protein ligase 1 C	1.13	1	1	1	793	89.46445351	6.668457031																			Identified
O88712	C-terminal-binding protein 1	Ctbp1	FUNCTION: Corepressor targeting diverse tra	C-terminal-binding protein 1	3.63	2	2	2	441	47.71453312	6.770996094	0.972108937	2	5.91186632	1.013160928	2	1.835019803	1.0276671	2	4.71249871	1.040525269	2	4.293174718	0.994065576	2	3.109585627	0.964113547	2	3.146338468	Quantified
Q8QZT1	Acetyl-CoA acetyltransferase, mitochondrial	Acat1	FUNCTION: This is one of the enzymes that c	Acetyl-CoA acetyltransferas	2.12	1	1	1	424	44.78734241	8.514160156	0.986095542	1		1.060832501	1		1.028191561	1		1.009900692	1		0.966769493	1		0.946222167	1		Quantified
P56716	Oxygen-regulated protein 1	Rp1	FUNCTION: Microtubule-associated protein r	Oxygen-regulated protein 1	0.38	1	1	4	2095	234.2414343	7.532714844	0.702471116	4	4.329883605	0.682232897	4	10.88439022	0.812862022	4	1.302227494	1.507320481	4	5.122004045	1.416880164	4	1.770692637	1.126857909	4	2.972072931	Quantified
Q8CIZ8	von Willebrand factor	Vwf	FUNCTION: Important in the maintenance of	von Willebrand factor OS=M	0.36	1	1	2	2813	309.0626996	5.503417969	0.960029461	2	2.036253061	0.966914156	2	0.797275928	0.979776947	2	0.698967608	0.939348476	2	6.075201381	0.882644954	2	1.573005472	1.252563961	2	7.008646461	Quantified
P13595	Neural cell adhesion molecule 1	Ncam1	FUNCTION: This protein is a cell adhesion m	Neural cell adhesion molec	1.79	2	2	2	1115	119.3534587	4.830566406	0.992954967	2	0.501738722	0.983674659	2	0.938119155	0.951230832	2	4.148678923	0.970043363	2	6.469291876	1.061296043	2	1.116408172	1.094368638	2	0.641200734	Quantified

Q9DC48	Pre-mRNA-processing factor 17	Cdc40	FUNCTION: Required for pre-mRNA splicing	Pre-mRNA-processing facto	1.73	1	1	1	579	65.41952669	7.063964844	0.993641863	1		0.927475088	1		1.027394272	1		0.985531126	1		0.975020248	1		1.106492878	1		Quantified
P49182	Heparin cofactor 2	Serpind1	FUNCTION: Thrombin inhibitor activated by th	Heparin cofactor 2 OS=Mus	2.51	1	1	2	478	54.46198944	7.342285156	1.010838029	2	3.049351251	0.964007538	2	4.059842319	0.909662759	2	2.723880448	1.059054725	2	4.142679716	1.054968554	2	1.926704986	1.05671019	2	6.725338717	Quantified
P97290	Plasma protease C1 inhibitor	Serping1	FUNCTION: Activation of the C1 complex is u	Plasma protease C1 inhibito	1.39	1	1	1	504	55.54915748	6.290527344	1.026694937	1		1.008770824	1		1.008169258	1		0.837466923	1		0.930952011	1		1.181796082	1		Quantified
Q9D1R9	60S ribosomal protein L34	Rpl34	FUNCTION: Component of the large ribosoma	60S ribosomal protein L34 (	20.51	3	3	3	117	13.28443648	11.47314453	0.97085663	3	13.30129302	0.984983216	3	0.033840405	0.938012846	3	30.11376011	0.982719972	3	7.864692592	0.97745095	3	24.95987808	1.009342335	3	1.772666464	Quantified
Q91YR1	Twinfilin-1	Twf1	FUNCTION: Actin-binding protein involved in	Twinfilin-1 OS=Mus muscul	7.43	3	3	3	350	40.05447261	6.668457031	0.988326552	3	1.347172096	0.989341275	3	7.903491522	1.017222362	3	0.620933459	0.987560223	3	3.032978798	1.033939525	3	4.515025514	1.014112704	3	3.58916116	Quantified
Q8BM72	Heat shock 70 kDa protein 13	Hspa13	FUNCTION: Has peptide-independent ATPas	Heat shock 70 kDa protein	9.13	4	4	5	471	51.67704786	5.630371094	0.991508085	5	4.659742415	0.95696296	5	4.288466027	0.965279705	5	5.559147652	1.004959141	5	6.357693774	1.072550412	5	8.47084083	1.057073103	5	5.490072104	Quantified
P70335	Rho-associated protein kinase 1	Rock1	FUNCTION: Protein kinase which is a key reg	Rho-associated protein kina	2.66	4	4	6	1354	158.071831	5.858886719	1.063247878	6	2.389118061	1.02354519	6	3.365676555	0.972412164	6	2.902712086	0.96447458	6	1.920855233	0.943675325	6	1.953844532	1.015898602	6	3.534403825	Quantified
P97823	Acyl-protein thioesterase 1	Lypla1	FUNCTION: Acts as a acyl-protein thioestera	Acyl-protein thioesterase 1	6.52	2	2	3	230	24.67136906	6.624511719	1.047568239	3	0.213339857	1.020450295	3	1.995217736	1.079484222	3	0.978052884	0.962095325	3	1.348032694	0.941986256	3	4.129899407	0.950865184	3	0.500238896	Quantified
P28661	Septin-4	Septin4	FUNCTION: Filament-forming cytoskeletal GT	Septin-4 OS=Mus musculus	5.44	2	3	3	478	54.90148702	5.871582031	1.000625554	2	1.101832946	1.112034109	2	7.448366697	0.991876565	2	6.424672501	0.919367813	2	6.390314417	0.909572458	2	13.25370007	1.037013476	2	0.471097379	Quantified
P35980	60S ribosomal protein L18	Rpl18	FUNCTION: Component of the large ribosoma	60S ribosomal protein L18 (	15.43	3	3	3	188	21.63118015	11.78076172	0.992604281	3	0.093131129	1.075322754	3	0.714552042	1.019177313	3	5.639329953	0.945832306	3	5.430996828	0.996926989	3	2.801163447	1.013950932	3	2.248294361	Quantified
Q9DBF1	Alpha-aminoadipic semialdehyde dehydrogenase	Aldh7a1	FUNCTION: Multifunctional enzyme mediatin	Alpha-aminoadipic semiald	3.34	2	2	3	539	58.82429871	7.474121094	0.985608747	3	4.667417176	1.055329217	3	6.287907554	0.966227232	3	10.68624479	0.940366976	3	0.039995409	0.997814616	3	2.155967722	1.002652849	3	3.906068359	Quantified
Q8CDN6	Thioredoxin-like protein 1	Txnl1	FUNCTION: Active thioredoxin with a redox p	Thioredoxin-like protein 1 C	3.11	1	1	2	289	32.21669632	4.957519531	1.047501931	2	1.16652161	1.012918721	2	1.613568067	1.016503786	2	15.5925659	0.970895005	2	11.65747324	0.988385024	2	18.34503459	0.961214443	2	1.373980363	Quantified
P55288	Cadherin-11	Cdh11	FUNCTION: Cadherins are calcium-dependen	Cadherin-11 OS=Mus musc	2.39	2	2	2	796	88.05785974	4.894042969	0.991656235	2	0.995669123	1.057989175	2	4.238052018	1.048375497	2	0.412026265	0.895140659	2	7.710133556	1.00459281	2	5.510853066	1.012361934	2	4.820253665	Quantified
P62331	ADP-ribosylation factor 6	Arf6	FUNCTION: GTP-binding protein involved in p	ADP-ribosylation factor 6 O	8.57	1	1	1	175	20.06943569	8.953613281	1.04205328	1		0.982069664	1		1.048860429	1		1.043894661	1		0.990387524	1		0.902377407	1		Quantified
Q05722	Collagen alpha-1(IX) chain	Col9a1	FUNCTION: Structural component of hyaline	Collagen alpha-1(IX) chain	0.87	1	1	1	921	92.03470509	8.953613281	0.995268537	1		0.998211536	1		0.930625429	1		1.056310052	1		1.06764874	1		1.008849741	1		Quantified
Q9CQM5	Thioredoxin domain-containing protein 17	Txndc17	FUNCTION: Disulfide reductase. May particip	Thioredoxin domain-contair	7.32	1	1	1	123	14.00574443	4.767089844	1.033364944	1		0.939747256	1		1.036129477	1		1.052146283	1		0.946933856	1		0.990383781	1		Quantified
Q9R1Q7	Proteolipid protein 2	Plp2	FUNCTION: May play a role in cell differentia	Proteolipid protein 2 OS=Mu	7.89	1	1	1	152	16.59669633	7.137207031	0.93090557	1		0.880395344	1		0.983760067	1		1.127260152	1		1.084124755	1		1.063306867	1		Quantified
P62996	Transformer-2 protein homolog beta	Tra2b	FUNCTION: Sequence-specific RNA-binding	Transformer-2 protein homo	7.29	2	2	2	288	33.64556011	11.25341797	1.013898608	2	1.418797044	1.073096599	2	3.744460872	0.94759839	2	0.350676216	0.999126827	2	3.118574008	0.885020112	2	1.178561612	1.054879278	2	0.468564026	Quantified
Q3UW53	Protein Niban 1	Niban1	FUNCTION: Regulates phosphorylation of a r	Protein Niban 1 OS=Mus m	1.94	2	2	2	926	102.5849309	4.779785156	0.968842583	2	8.136873755	0.953379706	2	3.593541866	0.945397688	2	12.74091697	1.067222941	2	3.642240326	1.002664727	2	8.049960715	1.082519728	2	17.6331943	Quantified
Q60520	Paired amphipathic helix protein Sin3a	Sin3a	FUNCTION: Acts as a transcriptional repress	Paired amphipathic helix pr	1.33	2	2	2	1274	144.9975117	7.254394531	0.905665955	2	0.622343914	0.90061947	2	2.760160948	0.97270805	2	3.922167851	1.108465804	2	2.134703657	1.194763391	2	4.651556968	1.031322255	2	3.01404624	Quantified
Q9JJQ0	GPI mannosyltransferase 3	Pigb	FUNCTION: Mannosyltransferase involved in	GPI mannosyltransferase 3	1.11	1	1	1	542	63.07858936	9.231933594																			Identified
Q9JII5	DAZ-associated protein 1	Dazap1	FUNCTION: RNA-binding protein, which may	DAZ-associated protein 1 C	2.71	1	1	1	406	43.18697274	8.558105469	1.001854479	1		1.022517495	1		1.240492134	1		0.843670182	1		0.881211473	1		0.951618882	1		Quantified
P52840	Sulfotransferase 1A1	Sult1a1	FUNCTION: Sulfotransferase that utilizes 3'-p	Sulfotransferase 1A1 OS=M	2.41	1	1	1	291	33.95223003	8.104003906	1.049355843	1		1.046682524	1		0.997250855	1		0.882054353	1		0.809542776	1		1.156146023	1		Quantified
Q9Z2M7	Phosphomannomutase 2	Pmm2	FUNCTION: Involved in the synthesis of the (	Phosphomannomutase 2 O	10.33	2	2	4	242	27.6389655	6.417480469	0.977440382	4	3.184836205	1.007385156	4	1.679586512	0.90067426	4	3.017628593	1.075229242	4	1.652777049	1.090680823	4	11.03085084	1.014836476	4	1.991211273	Quantified
Q9WTX5	S-phase kinase-associated protein 1	Skp1	FUNCTION: Essential component of the SCF	S-phase kinase-associated	15.95	2	2	2	163	18.66026136	4.538574219	0.945318456	2	0.372544864	0.968863143	2	2.058790993	0.991678443	2	8.20004687	1.035932177	2	12.22735971	1.016847075	2	9.195619696	1.068674369	2	0.112850685	Quantified
Q8VDP4	Cell cycle and apoptosis regulator protein 2	Ccar2	FUNCTION: Core component of the DBIRD c	Cell cycle and apoptosis re	0.98	1	1	1	922	102.9383917	5.249511719	0.985917639	1		0.915480598	1		0.97525109	1		1.113303907	1		1.051407004	1		1.010746659	1		Quantified
P70288	Histone deacetylase 2	Hdac2	FUNCTION: Responsible for the deacetylatio	Histone deacetylase 2 OS=	4.1	2	2	2	488	55.26675413	5.909667969	1.032040668	2	5.357464097	1.0036988	2	6.42135051	0.959279769	2	16.29791667	0.999445819	2	8.989226815	1.043446276	2	12.61556567	0.991941233	2	2.025638036	Quantified
Q8BHL4	Retinoic acid-induced protein 3	Gprc5a	FUNCTION: Orphan receptor. Could be involv	Retinoic acid-induced prote	2.25	1	1	1	356	40.07475781	7.620605469	0.942280301	1		0.988008143	1		1.179604485	1		0.927231981	1		0.860076332	1		1.048591948	1		Quantified
Q8CC86	Nicotinate phosphoribosyltransferase	Naprt	FUNCTION: Catalyzes the first step in the bio	Nicotinate phosphoribosyltr	4.46	2	2	2	538	58.22837153	6.455566406	0.472390217	2	154.292114	0.479419307	2	154.8360149	0.484274261	2	141.3833355	0.677484348	2	70.11143623	0.593916808	2	88.29398732	2.12550647	2	156.934539	Quantified
Q8CI51	PDZ and LIM domain protein 5	Pdlim5	FUNCTION: May play an important role in the	PDZ and LIM domain protei	3.21	2	2	3	591	63.25910118	8.250488281	0.983683191	3	7.813826588	0.979070761	3	1.026395053	1.021619847	3	6.888808882	1.003410236	3	3.408846211	1.022802986	3	1.380374568	1.041857347	3	4.994924835	Quantified
Q922S4	cGMP-dependent 3',5'-cyclic phosphodiesterase	Pde2a	FUNCTION: Cyclic nucleotide phosphodieste	cGMP-dependent 3',5'-cycli	0.85	1	1	1	939	105.5507193	5.414550781	1.061256196	1		1.028581884	1		1.042118326	1		1.022439293	1		0.898075228	1		0.916040768	1		Quantified
Q9DB16	Calcium-binding protein 39-like	Cab39l	FUNCTION: Component of a complex that bir	Calcium-binding protein 39-	2.67	1	1	1	337	39.08073054	8.309082031	0.9808137	1		0.997672044	1		0.979422133	1		0.949721537	1		1.083462755	1		1.068334644	1		Quantified
Q60953	Protein PML	Pml	FUNCTION: Functions via its association with	Protein PML OS=Mus musc	2.6	2	2	2	885	98.1796381	5.630371094	0.96647961	2	6.845465835	0.965193854	2	7.949208151	0.884922575	2	18.01983638	1.090233147	2	15.41104921	1.049384656	2	12.14346188	1.084163421	2	8.021329362	Quantified
Q8BLY2	Threonine--tRNA ligase 2, cytoplasmic	Tars3	FUNCTION: Catalyzes the attachment of thre	Threonine--tRNA ligase 2, c	2.28	1	2	2	790	91.26032176	7.532714844	0.94769773	1		1.062394413	1		1.117425994	1		0.936474406	1		0.982496434	1		0.948521344	1		Quantified
Q61210	Rho guanine nucleotide exchange factor 1	Arhgef1	FUNCTION: Seems to play a role in the regul	Rho guanine nucleotide exc	1.52	2	2	3	920	102.7406876	5.604980469	0.997524404	3	3.89624518	0.993693143	3	7.348924874	0.987500763	3	0.286381435	0.970400239	3	0.204205935	1.030876726	3	7.775117418	1.06008087	3	1.366109281	Quantified
Q7TSK7	ADAMTS-like protein 2	Adamtsl2		ADAMTS-like protein 2 OS=	1.67	2	2	2	957	105.5785646	6.829589844	0.988969619	2	4.855520872	1.05900874	2	3.314015899	1.098446434	2	3.952257567	0.942048307	2	10.15041757	0.899169814	2	6.300862537	0.969126582	2	9.658098663	Quantified
Q9EQK5	Major vault protein	Mvp	FUNCTION: Required for normal vault structu	Major vault protein OS=Mus	4.99	4	4	5	861	95.86517877	5.592285156	0.97181249	5	7.036437036	0.978443086	5	5.081366911	1.004994846	5	6.987016127	1.04951141	5	3.079204951	1.027234714	5	6.572854235	1.024999724	5	4.336679627	Quantified
Q71KU9	Fibrinogen-like protein 1	Fgl1	FUNCTION: Immune suppressive molecule th	Fibrinogen-like protein 1 OS	4.46	1	1	2	314	36.41641806	5.706542969	1.003052485	2	2.626316083	1.025390657	2	2.881137012	0.980243618	2	6.349956965	0.975440773	2	2.748234604	1.037568969	2	3.586733754	1.012985722	2	2.587544248	Quantified
P54071	Isocitrate dehydrogenase [NADP], mitochondrial	Idh2	FUNCTION: Plays a role in intermediary meta	Isocitrate dehydrogenase [N	1.77	1	1	1	452	50.87394857	8.689941406	1.083869409	1		1.037451266	1		0.964124059	1		0.984914418	1		0.907221195	1		1.004261116	1		Quantified
Q9D358	Low molecular weight phosphotyrosine protein phosp	Acp1	FUNCTION: Acts on tyrosine phosphorylated	Low molecular weight phos	6.96	1	1	1	158	18.17987761	6.741699219	1.000225353	1		0.958415482	1		1.02658348	1		1.05792023	1		1.030270364	1		0.959220383	1		Quantified
P15327	Bisphosphoglycerate mutase	Bpgm	FUNCTION: Plays a major role in regulating h	Bisphosphoglycerate mutas	10.81	3	3	3	259	29.95965805	7.063964844	0.977706624	3	5.875772867	1.042370509	3	1.519369918	0.995727015	3	2.115361776	0.986535941	3	7.541211558	0.997181481	3	3.518394668	0.988381399	3	0.663941044	Quantified
Q8K0T7	Protein unc-13 homolog C	Unc13c	FUNCTION: May play a role in vesicle matura	Protein unc-13 homolog C (	0.27	1	1	1	2210	249.6866093	6.074707031	1.046465911	1		0.925030576	1		1.029344974	1		1.121864416	1		0.889568396	1		0.963971977	1		Quantified
P52432	DNA-directed RNA polymerases I and III subunit RPA	Polr1c	FUNCTION: DNA-dependent RNA polymeras	DNA-directed RNA polymer	2.89	1	1	1	346	39.08194188	5.211425781																			Identified
Q9R1W5	Calcitonin gene-related peptide type 1 receptor	Calcrl	FUNCTION: Receptor for calcitonin-gene-rela	Calcitonin gene-related pep	2.16	1	1	1	463	53.22967319	6.712402344	0.960676221	1		0.984891299	1		0.967994258	1		1.018764665	1		0.92790702	1		1.13947777	1		Quantified
O89001	Carboxypeptidase D	Cpd		Carboxypeptidase D OS=M	0.58	1	1	1	1377	152.3098779	6.176269531	1.041136949	1		0.973478541	1		1.017003436	1		0.950325156	1		1.17586915	1		0.933151055	1		Quantified
P47199	Quinone oxidoreductase	Cryz	FUNCTION: Does not have alcohol dehydrog	Quinone oxidoreductase OS	2.11	1	1	1	331	35.24638299	8.074707031	1.009895987	1		1.047796993	1		1.024193353	1		0.996056315	1		0.995453052	1		0.93722982	1		Quantified
Q9D0L8	mRNA cap guanine-N7 methyltransferase	Rnmt	FUNCTION: Catalytic subunit of the mRNA-ca	mRNA cap guanine-N7 met	2.58	1	1	1	465	53.25765314	6.480957031	1.102004171	1		0.955975165	1		1.031646632	1		0.954028536	1		0.943679211	1		1.008092474	1		Quantified
Q9JHL1	Na(+)/H(+) exchange regulatory cofactor NHE-RF2	Slc9a3r2	FUNCTION: Scaffold protein that connects pl	Na(+)/H(+) exchange regula	14.24	4	4	4	337	37.37986249	7.591308594	0.98117539	4	2.958112477	1.029675051	4	2.256604997	0.993116177	4	6.514340843	1.001358376	4	5.057047138	1.028449437	4	4.544021939	1.012803442	4	9.823118491	Quantified
O54818	Tumor protein D53	Tpd52l1		Tumor protein D53 OS=Mus	5.88	2	2	2	204	22.5010613	6.214355469	0.977345237	2	1.19882221	1.01381897	2	6.896940554	1.012686305	2	0.336261683	0.995556945	2	3.885100198	0.969939471	2	6.318059898	1.035540935	2	0.89016689	Quantified
Q9D020	Cytosolic 5'-nucleotidase 3A	Nt5c3a	FUNCTION: Nucleotidase which shows speci	Cytosolic 5'-nucleotidase 3A	6.95	2	2	3	331	37.22837856	6.653808594	0.972238421	3	5.744999155	1.012365255	3	0.974267355	1.031935425	3	9.812416158	1.020386636	3	1.921844682	0.952606798	3	1.66280513	1.00055541	3	2.156694471	Quantified
Q62283	Tetraspanin-7	Tspan7	FUNCTION: May be involved in cell proliferati	Tetraspanin-7 OS=Mus mus	2.81	1	1	5	249	27.52572312	7.195800781	1.023994849	5	1.705836956	0.985799746	5	2.554395281	1.071776965	5	2.508466246	0.96825668	5	4.319447875	0.868500295	5	6.675322294	0.989763994	5	2.114790015	Quantified
Q8BGE6	Cysteine protease ATG4B	Atg4b	FUNCTION: Cysteine protease required for th	Cysteine protease ATG4B (	3.05	1	1	1	393	44.34671501	5.071777344																			Identified
P42232	Signal transducer and activator of transcription 5B	Stat5b	FUNCTION: Carries out a dual function: signa	Signal transducer and activ	4.07	4	4	5	786	89.94552649	6.049316406	1.008106657	5	3.691744972	0.973854023	5	4.515876994	0.999888728	5	4.138794074	0.987035199	5	3.730928565	1.00014069	5	5.093736113	0.985300984	5	3.671925602	Quantified
Q8R5H1	Ubiquitin carboxyl-terminal hydrolase 15	Usp15	FUNCTION: Hydrolase that removes conjuga	Ubiquitin carboxyl-terminal	0.71	1	1	1	981	112.2536916	5.173339844	1.070893758	1		1.051404508	1		0.988629725	1		0.967267009	1		0.902722147	1		0.995220551	1		Quantified
P20060	Beta-hexosaminidase subunit beta	Hexb	FUNCTION: Responsible for the degradation	Beta-hexosaminidase subu	4.29	2	2	2	536	61.07712133	8.118652344	0.908718815	2	6.856584957	1.044546403	2	2.546073971	1.092999128	2	11.82950636	0.950403519	2	1.581713855	0.994575894	2	0.902933029	1.009646314	2	4.214323411	Quantified
Q810B6	Rabankyrin-5	Ankfy1	FUNCTION: Proposed effector of Rab5. Binds	Rabankyrin-5 OS=Mus mus	1.8	2	2	3	1169	128.5711463	5.909667969	1.065251083	3	4.744038119	0.952295111	3	3.579374044	0.919667356	3	7.668631377	1	3	4.077238705	0.993701341	3	2.071354381	1.044585658	3	3.136676065	Quantified
Q03311	Cholinesterase	Bche	FUNCTION: Esterase with broad substrate sp	Cholinesterase OS=Mus mu	1.16	1	1	2	603	68.41818668	7.254394531	0.987830695	2	1.219820574	1.009746164	2	1.855407655	1.00987237	2	1.932158829	1.025711072	2	0.484777374	1.002650371	2	2.014700294	0.983687241	2	0.714595739	Quantified
Q99KK7	Dipeptidyl peptidase 3	Dpp3	FUNCTION: Cleaves and degrades bioactive	Dipeptidyl peptidase 3 OS=	12.2	8	8	9	738	82.84610912	5.376464844	1.019331926	9	3.792752625	0.992331246	9	5.273402045	1.04126301	9	11.61616142	0.999503496	9	6.609946352	1.006644281	9	6.862931581	0.994193679	9	4.605235903	Quantified
Q9JKY5	Huntingtin-interacting protein 1-related protein	Hip1r	FUNCTION: Component of clathrin-coated pit	Huntingtin-interacting protei	4.87	5	5	5	1068	119.352543	6.521972656	1.048545828	5	7.958399439	0.975374013	5	10.31018643	1.03569442	5	6.318196649	1.009453592	5	7.651012761	0.965929061	5	2.974031648	0.97459144	5	2.236103574	Quantified
P12246	Serum amyloid P-component	Apcs		Serum amyloid P-compone	2.68	1	1	1	224	26.23026788	6.354003906	1.033322763	1		1.057356973	1		1.064447084	1		0.947686626	1		0.927131609	1		0.946867517	1		Quantified
Q99KQ4	Nicotinamide phosphoribosyltransferase	Nampt	FUNCTION: The secreted form behaves both	Nicotinamide phosphoribos	1.43	1	1	1	491	55.41251189	7.151855469	0.921762668	1		0.99459856	1		1.021569937	1		1.041158025	1		0.990966817	1		1.047594908	1		Quantified
Q9DBA8	Probable imidazolonepropionase	Amdhd1		Probable imidazolonepropic	3.76	2	2	3	426	46.45855727	6.946777344	0.877299782	3	5.076859139	0.956485948	3	7.092468346	0.914992701	3	13.27347326	1.01993425	3	8.876962231	1.18326875	3	3.14879251	1.066131766	3	15.82421996	Quantified
O55143	Sarcoplasmic/endoplasmic reticulum calcium ATPas	Atp2a2	FUNCTION: This magnesium-dependent enzy	Sarcoplasmic/endoplasmic	0.67	1	1	1	1044	114.7836842	5.338378906	1.019811511	1		0.910697743	1		0.81051349	1		1.056581587	1		1.104881743	1		1.193983886	1		Quantified
P01749	Ig heavy chain V region 3	Ighv1-61		Ig heavy chain V region 3 O	5.98	1	1	1	117	13.0073327	7.869628906	0.947110049	1		0.913331334	1		0.869911839	1		1.185522834	1		1.145357322	1		1.043305685	1		Quantified
O54931	A-kinase anchor protein 2	Akap2	FUNCTION: Binds to regulatory subunit (RII)	A-kinase anchor protein 2 O	0.9	1	1	1	893	98.51859514	5.211425781	1.019473855	1		1.033293768	1		0.99036397	1		0.994398121	1		0.903401978	1		1.038798081	1		Quantified
P06801	NADP-dependent malic enzyme	Me1		NADP-dependent malic enz	4.37	3	3	4	572	63.91308761	7.444824219	0.99574305	4	1.269956043	1.010465653	4	3.405119361	0.994717085	4	1.577134636	1.028307073	4	2.207269533	1.00398666	4	10.33230204	0.999138229	4	4.224876059	Quantified
Q99PT1	Rho GDP-dissociation inhibitor 1	Arhgdia	FUNCTION: Controls Rho proteins homeosta	Rho GDP-dissociation inhib	19.12	4	4	6	204	23.39281598	5.198730469	1.014235028	6	2.663954996	1.014565227	6	3.006014757	1.01390954	6	7.490372351	0.979767746	6	6.365119373	0.954783806	6	8.200612841	0.999825045	6	7.323051024	Quantified
Q3UL36	Arginine and glutamate-rich protein 1	Arglu1		Arginine and glutamate-rich	8.49	3	3	3	271	32.86765884	10.35986328	0.982488562	3	5.53265837	1.001179017	3	1.815646502	1.029069592	3	4.436559066	0.975822788	3	2.334774287	1.07556818	3	3.48355641	0.979292231	3	11.27492956	Quantified
P70697	Uroporphyrinogen decarboxylase	Urod	FUNCTION: Catalyzes the decarboxylation of	Uroporphyrinogen decarbox	5.99	2	2	2	367	40.66589814	6.653808594	0.983143054	2	3.271406445	1.007803962	2	1.452964269	0.957602518	2	2.677975631	1.072576353	2	2.616342937	0.99780624	2	10.45738328	1.003323245	2	2.207559859	Quantified
Q02257	Junction plakoglobin	Jup	FUNCTION: Common junctional plaque prote	Junction plakoglobin OS=M	4.83	3	3	3	745	81.74878919	6.138183594	0.992059358	3	2.349360675	0.966794367	3	1.07508957	0.98797879	3	6.616393349	1.058300123	3	6.835796376	1.064610362	3	1.230957947	0.960727263	3	0.297200188	Quantified
Q64514	Tripeptidyl-peptidase 2	Tpp2	FUNCTION: Component of the proteolytic cas	Tripeptidyl-peptidase 2 OS=	1.19	2	2	2	1262	139.7905074	6.580566406	1.019449944	2	2.35212905	0.898710365	2	20.33176461	0.989999761	2	1.026187461	1.010125119	2	0.504876693	1.105656027	2	28.04572957	1.02944185	2	0.213768401	Quantified
P28650	Adenylosuccinate synthetase isozyme 1	Adss1	FUNCTION: Component of the purine nucleot	Adenylosuccinate syntheta	3.06	1	1	1	457	50.22299841	8.426269531	1.022025748	1		0.996865656	1		1.017686754	1		0.964130037	1		0.99254682	1		1.02259199	1		Quantified
Q61282	Aggrecan core protein	Acan	FUNCTION: This proteoglycan is a major con	Aggrecan core protein OS=	0.33	1	1	1	2132	221.8052921	4.297363281	1.10613866	1		1.060712644	1		0.951824741	1		0.954407215	1		0.868799387	1		1.023582117	1		Quantified
Q8VCA8	Secernin-2	Scrn2		Secernin-2 OS=Mus muscu	5.18	2	2	2	425	46.57122721	5.706542969	0.952341148	2	2.16394863	1.00640596	2	7.354634009	1.040910304	2	7.132354494	1.011134774	2	13.20593374	1.055463926	2	15.14570253	0.961873968	2	4.581492551	Quantified
Q9JIX8	Apoptotic chromatin condensation inducer in the nucl	Acin1	FUNCTION: Auxiliary component of the splici	Apoptotic chromatin conder	3.29	4	4	5	1338	150.6289296	5.909667969	1.025092582	5	3.111792218	1.096391153	5	4.270595405	0.919225366	5	8.898442296	0.965747097	5	5.211169941	0.878705189	5	4.332046616	1.066653452	5	2.392640592	Quantified
P38647	Stress-70 protein, mitochondrial	Hspa9	FUNCTION: Chaperone protein which plays a	Stress-70 protein, mitochon	1.33	1	1	1	679	73.41563193	6.074707031	0.974943037	1		0.985633757	1		0.969991614	1		0.985423667	1		0.979669217	1		1.124428652	1		Quantified
P14131	40S ribosomal protein S16	Rps16		40S ribosomal protein S16 (	4.79	1	1	1	146	16.43504168	10.21337891	0.963138485	1		0.96598555	1		0.935114892	1		1.091611156	1		1.079386842	1		1.02975864	1		Quantified
P31324	cAMP-dependent protein kinase type II-beta regulato	Prkar2b	FUNCTION: Regulatory subunit of the cAMP-	cAMP-dependent protein ki	1.44	1	1	1	416	46.13800376	4.982910156	0.993862182	1		0.940295248	1		1.010629922	1		1.065198346	1		0.998368658	1		1.015784193	1		Quantified
P51830	Adenylate cyclase type 9	Adcy9	FUNCTION: Adenylyl cyclase that catalyzes t	Adenylate cyclase type 9 O	0.89	1	1	1	1353	150.857178	7.210449219	0.88488101	1		1.170672828	1		1.281215044	1		0.784165041	1		0.906260241	1		0.909398781	1		Quantified
Q8C7R4	Ubiquitin-like modifier-activating enzyme 6	Uba6	FUNCTION: Activates ubiquitin by first adeny	Ubiquitin-like modifier-activa	1.61	2	2	2	1053	117.8908176	6.112792969	0.98335918	2	0.04752424	1.017616768	2	1.330418876	1.084444716	2	2.266462983	0.977195897	2	4.174018165	0.96568286	2	0.164299938	0.966245057	2	0.015944574	Quantified
Q9DCH4	Eukaryotic translation initiation factor 3 subunit F	Eif3f	FUNCTION: Component of the eukaryotic tran	Eukaryotic translation initiat	6.93	2	2	2	361	37.96032898	5.579589844	1.036980345	2	1.146547029	0.945067026	2	1.273630832	0.998273946	2	5.7999183	1.032578909	2	2.273561134	0.939993991	2	5.320920004	1.046253726	2	3.58994329	Quantified
P28660	Nck-associated protein 1	Nckap1	FUNCTION: Part of the WAVE complex that r	Nck-associated protein 1 O	0.89	1	1	1	1128	128.7004102	6.624511719	1.087581895	1		0.966317466	1		0.918456479	1		1.04345651	1		0.942614749	1		1.049884155	1		Quantified
Q9DB29	Isoamyl acetate-hydrolyzing esterase 1 homolog	Iah1	FUNCTION: Probable lipase. {ECO:0000250}	Isoamyl acetate-hydrolyzing	10.04	2	2	2	249	27.95619791	5.516113281	1.052619034	2	4.213115357	1.001764589	2	2.257749519	0.991735948	2	5.155928759	0.994832531	2	6.193385097	1.018220776	2	0.008163554	0.965799882	2	1.722739197	Quantified
Q61092	Laminin subunit gamma-2	Lamc2	FUNCTION: Binding to cells via a high affinity	Laminin subunit gamma-2 (	1.26	2	2	2	1191	130.0773932	6.303222656	0.96585401	2	4.032578139	0.957964632	2	2.189754743	0.978638825	2	1.253763364	1.034587064	2	2.809283554	1.096523256	2	11.96555353	1.031117485	2	2.966648351	Quantified
Q9DBS1	Transmembrane protein 43	Tmem43	FUNCTION: May have an important role in ma	Transmembrane protein 43	2.5	1	1	1	400	44.75506805	7.356933594	0.986394917	1		0.934902372	1		0.900591647	1		1.056127594	1		1.067367492	1		1.122590328	1		Quantified
Q64373	Bcl-2-like protein 1	Bcl2l1	FUNCTION: Potent inhibitor of cell death. Inhi	Bcl-2-like protein 1 OS=Mu	3.86	1	1	1	233	26.11576952	4.944824219	0.97647683	1		0.972538126	1		0.968387818	1		1.053539679	1		0.91439139	1		1.10879338	1		Quantified
Q61205	Platelet-activating factor acetylhydrolase IB subunit a	Pafah1b3	FUNCTION: Inactivates paf by removing the a	Platelet-activating factor ac	9.48	1	1	1	232	25.8373057	6.932128906	1.080557842	1		1.033915527	1		0.828321337	1		0.963225315	1		0.905377011	1		1.18948539	1		Quantified
Q8C1A5	Thimet oligopeptidase	Thop1	FUNCTION: Involved in the metabolism of ne	Thimet oligopeptidase OS=	3.64	2	2	2	687	77.9761971	6.062011719	1.00255262	2	2.719624519	1.039897336	2	4.801753438	0.982646968	2	3.790398328	0.997485771	2	1.949336466	1.007705989	2	14.72431396	0.987154841	2	5.774621544	Quantified
Q8BK63	Casein kinase I isoform alpha	Csnk1a1	FUNCTION: Casein kinases are operationally	Casein kinase I isoform alp	2.08	1	1	1	337	38.88988999	9.568847656	1.026210981	1		1.005366331	1		0.94467297	1		1.02842667	1		1.062951204	1		0.983962944	1		Quantified
Q3UKJ7	WD40 repeat-containing protein SMU1	Smu1	FUNCTION: Involved in pre-mRNA splicing a	WD40 repeat-containing pro	5.85	4	4	5	513	57.50723211	7.181152344	0.972841546	5	2.540822205	1.014677194	5	0.746045279	1.050547284	5	5.680806368	1.007421393	5	0.811163067	1.009716517	5	9.772444189	0.919907769	5	5.170414838	Quantified
P61222	ATP-binding cassette sub-family E member 1	Abce1	FUNCTION: Antagonizes the binding of 2-5A	ATP-binding cassette sub-fa	2	1	1	1	599	67.2710207	8.338378906	1.053271602	1		1.004487444	1		0.984951539	1		0.988842553	1		0.979074591	1		1.001689139	1		Quantified
O35900	U6 snRNA-associated Sm-like protein LSm2	Lsm2	FUNCTION: Plays role in pre-mRNA splicing	U6 snRNA-associated Sm-l	20	1	1	1	95	10.82762656	6.521972656	0.975274978	1		1.057559592	1		1.075982039	1		0.981062523	1		0.892024906	1		0.981111054	1		Quantified
P61358	60S ribosomal protein L27	Rpl27	FUNCTION: Component of the large ribosoma	60S ribosomal protein L27 (	22.06	2	2	2	136	15.78774833	10.56494141	0.97578782	2	1.137911227	1.011788247	2	7.441547936	0.947194437	2	7.517582256	1.057883837	2	3.649133582	1.086074064	2	3.85765699	0.97747651	2	9.003937897	Quantified
P62900	60S ribosomal protein L31	Rpl31		60S ribosomal protein L31 (	6.4	1	1	1	125	14.45393054	10.53564453	1.03155837	1		1.198427611	1		1.003865899	1		0.914368976	1		0.864340937	1		0.933622292	1		Quantified
O08599	Syntaxin-binding protein 1	Stxbp1	FUNCTION: Participates in the regulation of s	Syntaxin-binding protein 1 (	5.39	3	3	3	594	67.52586454	6.961425781	0.945747834	3	0.585862751	0.994787679	3	3.086585215	1.008414461	3	7.189385897	0.977464854	3	19.31466072	1.031105149	3	2.093292482	1.021639156	3	4.43593164	Quantified
P62264	40S ribosomal protein S14	Rps14		40S ribosomal protein S14 (	8.61	1	1	1	151	16.26253368	10.05224609	0.961410333	1		1.020189669	1		1.178012278	1		0.931182323	1		0.877819553	1		0.980189801	1		Quantified
P67871	Casein kinase II subunit beta	Csnk2b	FUNCTION: Plays a complex role in regulatin	Casein kinase II subunit be	3.72	1	1	1	215	24.92600328	5.554199219	0.982159591	1		0.915835978	1		0.947291125	1		1.223564358	1		0.89773384	1		1.025877439	1		Quantified
P62301	40S ribosomal protein S13	Rps13		40S ribosomal protein S13 (	5.96	1	1	1	151	17.21166734	10.53564453	0.979552571	1		1.049844778	1		1.038084081	1		1.000990712	1		0.926626826	1		0.987158368	1		Quantified
Q9D0M5	Dynein light chain 2, cytoplasmic	Dynll2	FUNCTION: Acts as one of several non-catal	Dynein light chain 2, cytopla	7.87	1	1	1	89	10.34306589	7.371582031	0.928050401	1		1.0545734	1		1.014116505	1		0.903210893	1		1.131964794	1		1.039394612	1		Quantified
Q9Z0H3	SWI/SNF-related matrix-associated actin-dependent	Smarcb1	FUNCTION: Core component of the BAF (SW	SWI/SNF-related matrix-ass	4.94	1	1	1	385	44.1131801	6.227050781	0.990859632	1		1.069872206	1		1.337395371	1		0.760551615	1		0.729139458	1		0.976778044	1		Quantified
P60867	40S ribosomal protein S20	Rps20		40S ribosomal protein S20 (	30.25	4	4	4	119	13.36430914	9.935058594	0.989911297	4	7.334557669	1.006166394	4	1.084241595	0.954522077	4	5.470700321	1.036617455	4	1.012074089	1.011898835	4	2.311197483	1.019704065	4	2.524923978	Quantified
P62751	60S ribosomal protein L23a	Rpl23a	FUNCTION: Component of the ribosome, a la	60S ribosomal protein L23a	8.33	1	1	1	156	17.68413502	10.44775391	0.996251429	1		0.988132189	1		0.928596561	1		1.062382965	1		1.082256392	1		1.006283546	1		Quantified
P62743	AP-2 complex subunit sigma	Ap2s1	FUNCTION: Component of the adaptor protei	AP-2 complex subunit sigm	16.2	2	2	2	142	17.00670103	6.176269531	1.000845385	2	4.260935111	0.939047927	2	5.00700562	0.98478742	2	2.65239555	1.045442958	2	6.729503433	0.97377265	2	16.25889496	1.068073029	2	4.095113397	Quantified
Q6ZWU9	40S ribosomal protein S27	Rps27	FUNCTION: Component of the small ribosom	40S ribosomal protein S27 (	22.62	2	2	2	84	9.454836185	9.451660156	0.874703123	2	4.274556325	0.959143631	2	1.54329345	1.093519322	2	2.887253411	1.143125436	2	6.133956082	1.032360355	2	8.933515584	0.917282748	2	15.3479142	Quantified
P62889	60S ribosomal protein L30	Rpl30		60S ribosomal protein L30 (	6.96	1	1	1	115	12.77574215	9.627441406	0.947087897	1		1.026027577	1		0.829865696	1		1.036345179	1		1.14538612	1		1.11677351	1		Quantified
P62878	E3 ubiquitin-protein ligase RBX1	Rbx1	FUNCTION: E3 ubiquitin ligase component of	E3 ubiquitin-protein ligase R	16.67	1	1	1	108	12.26573859	6.961425781	0.923870146	1		0.989932435	1		1.053354972	1		0.999506736	1		0.980959698	1		1.062920072	1		Quantified
P83940	Elongin-C	Eloc	FUNCTION: SIII, also known as elongin, is a	Elongin-C OS=Mus muscul	8.04	1	1	1	112	12.4649994	4.779785156	1.004418135	1		1.044128663	1		1.062306384	1		0.944866838	1		0.960739254	1		0.976759611	1		Quantified
Q9CZM2	60S ribosomal protein L15	Rpl15		60S ribosomal protein L15 (	4.41	1	1	2	204	24.13108494	11.61962891	0.962098193	2	1.217493225	0.94445555	2	10.66846935	1.045899361	2	1.674315017	1.049181395	2	5.214250308	0.963846797	2	11.62078523	1.035982102	2	1.951566708	Quantified
P62911	60S ribosomal protein L32	Rpl32		60S ribosomal protein L32 (	9.63	1	1	1	135	15.84980494	11.32666016	0.926177641	1		0.980524892	1		1.020485975	1		1.044607356	1		1.121078263	1		0.97802627	1		Quantified
P62313	U6 snRNA-associated Sm-like protein LSm6	Lsm6	FUNCTION: Plays role in pre-mRNA splicing	U6 snRNA-associated Sm-l	10	1	1	1	80	9.121767515	9.583496094	0.981316824	1		0.953963348	1		1.006621675	1		1.148886245	1		0.976905823	1		0.945772299	1		Quantified
P10852	4F2 cell-surface antigen heavy chain	Slc3a2	FUNCTION: Component of several heterodim	4F2 cell-surface antigen he	1.71	1	1	1	526	58.3003234	5.909667969	0.96313846	1		1.003742232	1		0.921567723	1		1.237855618	1		1.018123432	1		0.890940333	1		Quantified
O54950	5'-AMP-activated protein kinase subunit gamma-1	Prkag1	FUNCTION: AMP/ATP-binding subunit of AM	5'-AMP-activated protein ki	2.42	1	1	1	330	37.49676169	7.137207031	0.944245999	1		1.053008275	1		0.916064882	1		0.981515269	1		1.136378285	1		1.053218447	1		Quantified
Q3THG9	Alanyl-tRNA editing protein Aarsd1	Aarsd1	FUNCTION: Functions in trans to edit the ami	Alanyl-tRNA editing protein	3.16	1	1	2	412	44.94321048	6.417480469	0.98424236	2	1.246101225	0.929856249	2	0.107102556	1.059675301	2	6.513385267	0.933254121	2	1.859716444	1.105488432	2	11.38746382	1.048431688	2	3.094028216	Quantified
Q6P542	ATP-binding cassette sub-family F member 1	Abcf1	FUNCTION: Required for efficient Cap- and IF	ATP-binding cassette sub-fa	1.91	1	2	2	837	94.88689851	6.507324219	0.976059665	1		0.957290041	1		0.98957608	1		1.03087463	1		1.097189231	1		1.015207911	1		Quantified
Q8CBW3	Abl interactor 1	Abi1	FUNCTION: May act in negative regulation of	Abl interactor 1 OS=Mus mu	3.12	1	1	1	481	52.25526549	7.635253906	0.922635405	1		0.95626343	1		0.943083778	1		1.067740402	1		0.955955029	1		1.17227959	1		Quantified
Q9CQR4	Acyl-coenzyme A thioesterase 13	Acot13	FUNCTION: Acyl-CoA thioesterases are a gro	Acyl-coenzyme A thioestera	5.71	1	1	1	140	15.17290133	8.821777344	0.954259221	1		1.072597707	1		1.075699207	1		0.917581406	1		0.961335339	1		1.009922897	1		Quantified
P00329	Alcohol dehydrogenase 1	Adh1		Alcohol dehydrogenase 1 (	2.4	1	1	1	375	39.7455614	8.104003906	1.095812245	1		1.074469826	1		1.016216152	1		0.932199326	1		0.904616587	1		0.947007134	1		Quantified
Q60994	Adiponectin	Adipoq	FUNCTION: Important adipokine involved in t	Adiponectin OS=Mus musc	4.05	2	2	3	247	26.79223255	5.566894531	0.996605112	3	1.963356598	0.934578702	3	2.299492737	0.986132087	3	1.731256687	1.051935218	3	4.375913526	0.975207571	3	5.647024326	1.001726461	3	1.616985647	Quantified
Q9JKV1	Proteasomal ubiquitin receptor ADRM1	Adrm1	FUNCTION: Component of the 26S proteasor	Proteasomal ubiquitin recep	2.21	1	1	1	407	42.03371124	5.071777344	1.010901061	1		0.998051	1		1.022993942	1		0.971962553	1		0.979542191	1		1.026669455	1		Quantified

Q8BK64	Activator of 90 kDa heat shock protein ATPase homo	Ahsa1	FUNCTION: Acts as a co-chaperone of HSP9	Activator of 90 kDa heat sh	2.66	1	1	1	338	38.0932202	5.528808594	1.016584469	1		0.997585057	1		1.062449615	1		0.927183364	1		1.029307614	1		0.992152049	1		Quantified
D3YVF0	A-kinase anchor protein 5	Akap5	FUNCTION: Multivalent scaffold protein that a	A-kinase anchor protein 5 C	0.81	1	1	1	745	79.34952454	4.754394531	0.988665375	1		0.978793446	1		1.170790011	1		0.939384324	1		1.052426127	1		0.892350297	1		Quantified
Q07456	Protein AMBP	Ambp	FUNCTION: Inter-alpha-trypsin inhibitor inhibi	Protein AMBP OS=Mus mu	2.58	1	1	1	349	39.00403942	6.315917969	0.97557193	1		0.966228135	1		0.915892278	1		0.967562383	1		1.019413252	1		1.201146176	1		Quantified
P97467	Peptidyl-glycine alpha-amidating monooxygenase	Pam	FUNCTION: Bifunctional enzyme that catalyz	Peptidyl-glycine alpha-amic	0.82	1	1	1	979	108.8935907	6.551269531	0.992832652	1		1.016494307	1		0.975805776	1		0.977638202	1		1.022119633	1		1.047481648	1		Quantified
O08739	AMP deaminase 3	Ampd3	FUNCTION: AMP deaminase plays a critical	AMP deaminase 3 OS=Mus	1.17	1	1	1	766	88.59560721	7.327636719	1.081091776	1		1.09342153	1		0.911456463	1		0.914679645	1		0.896386057	1		1.083538756	1		Quantified
P16406	Glutamyl aminopeptidase	Enpep	FUNCTION: Regulates central hypertension t	Glutamyl aminopeptidase C	1.06	1	1	1	945	107.8880548	5.439941406	0.940867548	1		1.036693839	1		0.792440728	1		1.05680985	1		1.266401599	1		1.06043793	1		Quantified
Q9JIF0	Protein arginine N-methyltransferase 1	Prmt1	FUNCTION: Arginine methyltransferase that r	Protein arginine N-methyltra	9.16	3	3	3	371	42.40793749	5.427246094	1.020321051	3	3.234871122	1.017442778	3	0.592391425	0.998206078	3	1.411284324	0.943221382	3	1.497192671	0.938967047	3	10.48285445	0.941535925	3	1.482565895	Quantified
B2RXR6	Serine/threonine-protein phosphatase 6 regulatory ar	Ankrd44	FUNCTION: Putative regulatory subunit of pro	Serine/threonine-protein ph	1.01	1	1	1	993	107.3173025	6.417480469	1.00189122	1		1.043515608	1		1.038891593	1		1.017905565	1		0.942107692	1		0.943334443	1		Quantified
Q9CZ52	Anthrax toxin receptor 1	Antxr1	FUNCTION: Plays a role in cell attachment ar	Anthrax toxin receptor 1 OS	1.42	1	1	1	562	62.26876413	7.605957031	0.96203878	1		0.999562122	1		1.02508247	1		0.961952711	1		1.042195126	1		1.046176734	1		Quantified
Q9JME5	AP-3 complex subunit beta-2	Ap3b2	FUNCTION: Subunit of non-clathrin- and clath	AP-3 complex subunit beta-	0.74	1	1	1	1082	119.1180314	5.630371094	0.97489391	1		0.965723214	1		1.075334935	1		0.980731325	1		1.03294279	1		0.999313666	1		Quantified
Q8R146	Acylamino-acid-releasing enzyme	Apeh	FUNCTION: This enzyme catalyzes the hydro	Acylamino-acid-releasing e	2.73	2	2	2	732	81.5286466	5.592285156	1.01172892	2	2.657846145	1.017680522	2	1.797423133	1.082393576	2	8.003785621	1.000552263	2	0.100155875	0.925554438	2	1.612544657	0.937245962	2	4.51500416	Quantified
Q99MK8	Beta-adrenergic receptor kinase 1	Grk2	FUNCTION: Specifically phosphorylates the a	Beta-adrenergic receptor ki	2.9	2	2	2	689	79.58752038	7.283691406	0.965736343	2	16.95700033	0.985878057	2	12.34308789	0.971160329	2	7.521958404	1.030716365	2	8.040781046	1.036015714	2	20.10411855	1.026444119	2	17.46352878	Quantified
Q9R0Q6	Actin-related protein 2/3 complex subunit 1A	Arpc1a	FUNCTION: Probably functions as componen	Actin-related protein 2/3 co	6.49	2	2	2	370	41.5996876	8.177246094	1.01360506	2	3.44569958	1.020128071	2	4.357002578	0.984884969	2	4.573115218	1.02396476	2	2.081037295	0.978698494	2	6.775121714	0.987736957	2	4.641231645	Quantified
Q9D8S3	ADP-ribosylation factor GTPase-activating protein 3	Arfgap3	FUNCTION: GTPase-activating protein (GAP	ADP-ribosylation factor GTF	1.34	1	1	1	523	57.42021784	8.470214844	1.050944546	1		1.025495607	1		1.032892684	1		1.060015039	1		0.874842894	1		0.916303855	1		Quantified
Q8CG72	ADP-ribose glycohydrolase ARH3	Adprs	FUNCTION: ADP-ribose glycohydrolase that	ADP-ribose glycohydrolase	2.97	1	1	1	370	39.3893693	4.957519531	0.993114764	1		1.092454506	1		1.06934772	1		0.798788343	1		1.081105949	1		1.003992346	1		Quantified
A2BH40	AT-rich interactive domain-containing protein 1A	Arid1a	FUNCTION: Involved in transcriptional activa	AT-rich interactive domain-	0.48	1	1	1	2283	241.9391769	6.683105469	0.990451644	1		1.067860352	1		1.012613478	1		0.958735645	1		0.921881781	1		1.030888364	1		Quantified
Q8CG76	Aflatoxin B1 aldehyde reductase member 2	Akr7a2	FUNCTION: Catalyzes the NADPH-depender	Aflatoxin B1 aldehyde redu	2.72	1	1	3	367	40.58621032	8.118652344	0.954334795	3	2.626692355	1.067480454	3	1.370357322	1.092721714	3	7.663123627	0.947147119	3	4.602589509	0.951848361	3	0.135708275	0.998026589	3	4.003142463	Quantified
Q8VEH3	ADP-ribosylation factor-like protein 8A	Arl8a	FUNCTION: Plays a role in lysosomes motilit	ADP-ribosylation factor-like	4.84	1	1	1	186	21.37602909	7.767089844	0.981246757	1		1.01833767	1		1.018296271	1		0.975591456	1		1.020903528	1		1.011986049	1		Quantified
P50428	Arylsulfatase A	Arsa	FUNCTION: Hydrolyzes cerebroside sulfate.	Arylsulfatase A OS=Mus mu	1.38	1	1	1	506	53.71370657	5.871582031	1.028861174	1		1.114665218	1		0.996283769	1		0.957425299	1		0.968504845	1		0.931000814	1		Quantified
Q3TYD4	Arylsulfatase G	Arsg	FUNCTION: Displays arylsulfatase activity wi	Arylsulfatase G OS=Mus m	3.62	2	2	2	525	57.39739231	6.392089844	0.96161502	2	6.725845681	0.99851036	2	2.509563595	0.975365134	2	5.955204607	1.015308094	2	13.62239032	0.989341755	2	1.705374168	1.075176374	2	0.954348225	Quantified
Q91WU5	Arsenite methyltransferase	As3mt	FUNCTION: Catalyzes the transfer of a methy	Arsenite methyltransferase	2.39	1	1	1	376	41.76711726	5.858886719	0.974540188	1		0.931850265	1		0.907296165	1		1.175892501	1		1.072103176	1		1.005535461	1		Quantified
Q9DCQ2	Putative L-aspartate dehydrogenase	Aspdh	FUNCTION: Specifically catalyzes the NAD o	Putative L-aspartate dehydr	4.18	1	1	1	287	30.25069568	6.946777344	0.937726937	1		0.984741161	1		1.04253419	1		1.048266095	1		1.018513057	1		0.994136594	1		Quantified
Q99MQ4	Asporin	Aspn	FUNCTION: Binds calcium and plays a role ir	Asporin OS=Mus musculus	2.14	1	1	1	373	42.54518323	8.572753906	0.896778538	1		1.046126284	1		1.059216053	1		1.06289162	1		0.947236443	1		0.97798551	1		Quantified
Q80T21	ADAMTS-like protein 4	Adamtsl4	FUNCTION: Positive regulation of apoptosis.	ADAMTS-like protein 4 OS=	1.83	1	1	1	1036	113.1521783	8.147949219	1.034719774	1		1.061566186	1		0.846725417	1		1.02152479	1		1.085919163	1		1.017751296	1		Quantified
Q91YH5	Atlastin-3	Atl3	FUNCTION: GTPase tethering membranes th	Atlastin-3 OS=Mus musculu	2.03	1	1	1	541	60.5365658	6.100097656	0.99198375	1		1.03528007	1		0.98913338	1		1.015360312	1		0.969280794	1		1.005682028	1		Quantified
Q9R001	A disintegrin and metalloproteinase with thrombospo	Adamts5	FUNCTION: Metalloproteinase that plays an i	A disintegrin and metallopro	0.86	1	1	1	930	101.778648	8.719238281	1.060554628	1		0.89068468	1		1.06115803	1		1.008360787	1		1.143945072	1		0.913992887	1		Quantified
P59017	Bcl-2-like protein 13	Bcl2l13	FUNCTION: May promote the activation of ca	Bcl-2-like protein 13 OS=Mu	2.53	1	1	1	434	46.6905463	4.589355469	1.058139386	1		0.955301321	1		1.224903672	1		0.866776452	1		0.93702349	1		0.927720544	1		Quantified
Q09200	Beta-1,4 N-acetylgalactosaminyltransferase 1	B4galnt1	FUNCTION: Involved in the biosynthesis of g	Beta-1,4 N-acetylgalactosa	2.06	1	1	1	533	59.17509707	8.587402344	0.45610279	1		0.448652073	1		0.691203565	1		2.783948705	1		1.47376224	1		0.442552494	1		Quantified
P15535	Beta-1,4-galactosyltransferase 1	B4galt1	FUNCTION: [Beta-1,4-galactosyltransferase 1	Beta-1,4-galactosyltransfera	2.01	1	1	1	399	44.382864	9.378417969	0.997890832	1		1.03977111	1		0.9879381	1		1.099130525	1		0.92668658	1		0.935966265	1		Quantified
P18572	Basigin	Bsg	FUNCTION: Plays an important role in targeti	Basigin OS=Mus musculus	2.06	1	1	1	389	42.41831579	5.846191406	0.997598358	1		0.998335651	1		1.099460248	1		0.997214899	1		0.870928871	1		0.992690168	1		Quantified
Q9Z277	Tyrosine-protein kinase BAZ1B	Baz1b	FUNCTION: Atypical tyrosine-protein kinase t	Tyrosine-protein kinase BA	0.47	1	1	1	1479	170.5437714	8.602050781	0.864605677	1		0.883930466	1		0.857611411	1		1.163912788	1		1.187210388	1		1.172802373	1		Quantified
Q07813	Apoptosis regulator BAX	Bax	FUNCTION: Accelerates programmed cell de	Apoptosis regulator BAX OS	6.77	1	1	1	192	21.38081559	4.982910156	1.106193544	1		0.785836529	1		1.146352537	1		1.184510045	1		0.977562829	1		0.80530798	1		Quantified
Q9R069	Basal cell adhesion molecule	Bcam	FUNCTION: Laminin alpha-5 receptor. May m	Basal cell adhesion molecu	3.38	2	2	2	622	67.62756708	6.252441406	1.033446129	2	11.00284142	0.81336023	2	10.01454204	1.047808409	2	2.222287415	1.008615947	2	1.364718712	1.116017151	2	13.59371201	1.045899331	2	15.36787709	Quantified
P98063	Bone morphogenetic protein 1	Bmp1	FUNCTION: Cleaves the C-terminal propeptic	Bone morphogenetic protei	2.62	3	3	3	991	111.5952457	6.946777344	0.96744346	3	1.801033277	1.005185958	3	8.292350212	1.01393122	3	5.006268638	1.01818517	3	6.324781474	0.98140922	3	7.477320147	0.994696701	3	12.94673935	Quantified
P97452	Ribosome biogenesis protein BOP1	Bop1	FUNCTION: Component of the PeBoW comp	Ribosome biogenesis prote	0.96	1	1	1	732	82.49355628	6.277832031	0.978279838	1		0.965019093	1		1.013223899	1		1.00475065	1		1.053470713	1		1.030394728	1		Quantified
P46737	Lys-63-specific deubiquitinase BRCC36	Brcc3	FUNCTION: Metalloprotease that specifically	Lys-63-specific deubiquitina	6.53	1	1	1	291	33.31876222	5.833496094	0.918189143	1		1.035864367	1		1.002368839	1		0.972638438	1		1.058367064	1		1.05706277	1		Quantified
Q8BRN9	Coiled-coil and C2 domain-containing protein 1B	Cc2d1b	FUNCTION: Transcription factor that binds sp	Coiled-coil and C2 domain-	0.83	1	1	1	848	93.03387938	5.414550781	0.969196982	1		1.037727206	1		1.129666019	1		0.942617223	1		0.907396372	1		0.978270554	1		Quantified
Q8BHG2	CXXC motif containing zinc binding protein	Czib		CXXC motif containing zinc	10.63	1	1	1	160	18.00885151	5.122558594	0.945753785	1		1.00105303	1		1.006606438	1		1.018198961	1		1.03943985	1		1.026770111	1		Quantified
Q64444	Carbonic anhydrase 4	Ca4	FUNCTION: Reversible hydration of carbon d	Carbonic anhydrase 4 OS=	4.26	1	1	1	305	34.32950504	8.206542969	0.929821687	1		0.946262499	1		1.007501387	1		1.129158853	1		1.072181704	1		0.969892371	1		Quantified
O35887	Calumenin	Calu	FUNCTION: Involved in regulation of vitamin	Calumenin OS=Mus muscu	6.67	2	2	2	315	37.0405248	4.665527344	1.013253354	2	3.31320769	1.005812995	2	3.321660923	0.972889057	2	2.258110967	0.996721732	2	0.75400842	1.000840214	2	3.885575157	1.033687741	2	0.720091503	Quantified
P10605	Cathepsin B	Ctsb	FUNCTION: Thiol protease which is believed	Cathepsin B OS=Mus musc	2.36	1	1	1	339	37.25568316	5.909667969	1.020828354	1		1.066638436	1		0.986038116	1		0.917707744	1		1.036422753	1		1.003834623	1		Quantified
P49935	Pro-cathepsin H	Ctsh	FUNCTION: Important for the overall degrada	Pro-cathepsin H OS=Mus m	2.1	1	1	1	333	37.14609038	8.396972656	0.98930099	1		0.874773385	1		0.920828568	1		1.2372659	1		1.143155664	1		0.932376594	1		Quantified
O70370	Cathepsin S	Ctss	FUNCTION: Thiol protease. Key protease res	Cathepsin S OS=Mus musc	2.06	1	1	1	340	38.44945752	6.961425781	0.922393973	1		0.895370452	1		0.971429444	1		0.999824975	1		1.01027208	1		1.244236101	1		Quantified
Q8K354	Carbonyl reductase [NADPH] 3	Cbr3	FUNCTION: Has low NADPH-dependent oxid	Carbonyl reductase [NADPH	5.05	1	1	1	277	30.93391921	6.565917969	0.993865175	1		0.9815275	1		0.927116135	1		1.078476475	1		1.020880201	1		1.03821538	1		Quantified
Q8CH18	Cell division cycle and apoptosis regulator protein 1	Ccar1	FUNCTION: Associates with components of t	Cell division cycle and apop	0.7	1	1	1	1146	131.9788488	5.757324219	0.964840045	1		0.942504416	1		0.865782063	1		1.065621327	1		1.096472704	1		1.149124007	1		Quantified
Q8BVC4	Coiled-coil domain-containing protein 68	Ccdc68	FUNCTION: Centriolar protein required for ce	Coiled-coil domain-containi	3.9	1	1	1	333	37.96859659	7.327636719	0.98238604	1		1.090807947	1		1.046650906	1		0.893707446	1		1.007711742	1		0.990025572	1		Quantified
D3YZP9	Coiled-coil domain-containing protein 6	Ccdc6		Coiled-coil domain-containi	1.49	1	1	1	469	52.90582111	7.342285156	0.953859442	1		1.019494165	1		1.031672222	1		1.000692978	1		1.019458515	1		0.999295748	1		Quantified
Q64314	Hematopoietic progenitor cell antigen CD34	Cd34	FUNCTION: Possible adhesion molecule with	Hematopoietic progenitor ce	2.09	1	1	1	382	40.95710476	5.300292969	0.972779383	1		0.979627407	1		1.091823785	1		1.022653969	1		0.978775266	1		0.957136992	1		Quantified
P40240	CD9 antigen	Cd9	FUNCTION: Integral membrane protein assoc	CD9 antigen OS=Mus musc	5.31	1	1	1	226	25.24090384	7.225097656	1.022512716	1		0.932455674	1		0.925999277	1		1.074475068	1		1.167574042	1		0.979694385	1		Quantified
Q91WE6	Threonylcarbamoyladenosine tRNA methylthiotransfe	Cdkal1	FUNCTION: Catalyzes the methylthiolation of	Threonylcarbamoyladenosi	1.38	1	1	1	578	65.24733528	7.649902344	2.209900437	1		0.676513609	1		0.70104899	1		0.802489381	1		0.740765246	1		0.813646986	1		Quantified
P31809	Carcinoembryonic antigen-related cell adhesion mole	Ceacam1	FUNCTION: [Isoform 1]: Cell adhesion proteir	Carcinoembryonic antigen-r	3.26	1	1	2	521	56.98058633	5.579589844	1.048474679	2	7.628043937	0.904486711	2	9.966783892	1.037409936	2	1.365078195	0.978273407	2	5.469369153	0.948592596	2	12.38575636	1.080107298	2	1.474334773	Quantified
Q9WUD1	STIP1 homology and U box-containing protein 1	Stub1	FUNCTION: E3 ubiquitin-protein ligase which	STIP1 homology and U box	2.63	1	1	1	304	34.88731271	6.011230469	0.571633788	1		0.538016785	1		0.539306347	1		0.639278936	1		0.632307572	1		3.092446098	1		Quantified
Q9CR86	Calcium-regulated heat stable protein 1	Carhsp1	FUNCTION: Binds mRNA and regulates the s	Calcium-regulated heat stal	5.41	1	1	1	148	16.05213495	8.206542969	0.966092618	1		1.027536546	1		1.006308736	1		1.024819309	1		1.027887132	1		0.97681151	1		Quantified
A2AGT5	Cytoskeleton-associated protein 5	Ckap5	FUNCTION: Binds to the plus end of microtut	Cytoskeleton-associated pr	0.64	1	1	1	2032	225.4918816	7.957519531	0.921473416	1		1.012972014	1		1.071305152	1		0.969835584	1		0.979422224	1		1.050764524	1		Quantified
Q61419	Cytidine monophosphate-N-acetylneuraminic acid hy	Cmah	FUNCTION: Sialic acids are components of c	Cytidine monophosphate-N	1.39	1	1	1	577	66.89251345	6.379394531	0.968067361	1		0.887848206	1		1.005019976	1		1.077487456	1		1.085599467	1		1.042034761	1		Quantified
Q3U5Q7	UMP-CMP kinase 2, mitochondrial	Cmpk2	FUNCTION: May participate in dUTP and dC	UMP-CMP kinase 2, mitoch	5.59	2	2	2	447	50.00472843	7.269042969	1.059823785	2	3.524133967	1.01098295	2	1.937233345	1.002481169	2	3.214498316	0.966299261	2	1.269433323	0.994598685	2	1.339234216	0.980106336	2	5.052467419	Quantified
Q9CZT6	Protein CMSS1	Cmss1		Protein CMSS1 OS=Mus m	7.25	1	1	1	276	31.57354649	8.455566406	0.941033901	1		0.944648247	1		0.726974723	1		1.265751076	1		1.276804224	1		1.016817792	1		Quantified
Q9DBC3	Cap-specific mRNA (nucleoside-2'-O-)-methyltransfe	Cmtr1	FUNCTION: S-adenosyl-L-methionine-depen	Cap-specific mRNA (nuclec	2.87	3	3	3	837	95.61506599	7.269042969	0.97464562	3	6.350906246	0.954416123	3	0.472468396	0.999542297	3	2.508070472	0.975996958	3	10.93946706	1.04734817	3	11.25143854	1.037511301	3	3.913585314	Quantified
Q9CQE8	RNA transcription, translation and transport factor pro	RTRAF	FUNCTION: RNA-binding protein involved in	RNA transcription, translatio	3.28	1	1	1	244	28.13477719	6.888183594	0.701375017	1		0.778935649	1		0.666488553	1		1.284563198	1		1.935076175	1		1.103906807	1		Quantified
P53996	Cellular nucleic acid-binding protein	Cnbp	FUNCTION: Single-stranded DNA-binding pro	Cellular nucleic acid-binding	14.61	3	3	4	178	19.57860709	7.576660156	1.039359861	4	1.642382749	1.009759767	4	6.912818151	1.063851424	4	7.468391435	0.951905032	4	5.448972166	0.922651152	4	14.68815468	0.979361646	4	4.014753342	Quantified
Q9JJZ8	Cyclic nucleotide-gated cation channel alpha-3	Cnga3	FUNCTION: Visual signal transduction is med	Cyclic nucleotide-gated cati	2.38	1	1	1	631	72.65562469	6.990722656	1.052478054	1		0.580944754	1		1.12339073	1		1.094728097	1		1.425838723	1		0.936046939	1		Quantified
Q08093	Calponin-2	Cnn2	FUNCTION: Thin filament-associated protein	Calponin-2 OS=Mus muscu	5.57	1	2	2	305	33.13404155	7.620605469	1.033500182	1		0.984549515	1		0.971164706	1		1.065000854	1		0.99482553	1		0.972791647	1		Quantified
Q69Z26	Contactin-4	Cntn4	FUNCTION: Contactins mediate cell surface i	Contactin-4 OS=Mus muscu	0.88	1	1	1	1026	113.4186753	7.356933594	0.961165849	1		1.050572369	1		0.957597216	1		1.074794868	1		0.922594911	1		1.02421865	1		Quantified
Q9DBL7	Bifunctional coenzyme A synthase	Coasy	FUNCTION: Bifunctional enzyme that catalyz	Bifunctional coenzyme A sy	1.95	1	1	1	563	61.98463691	7.107910156	0.988951217	1		0.992344733	1		1.057208105	1		0.925088694	1		0.941259769	1		1.087817767	1		Quantified
Q61245	Collagen alpha-1(XI) chain	Col11a1	FUNCTION: May play an important role in fibr	Collagen alpha-1(XI) chain	1.5	3	3	4	1804	180.9209228	5.211425781	0.837507145	4	18.24495373	0.848445397	4	13.25620546	0.855099364	4	11.01917978	1.236693912	4	11.33029051	1.683250205	4	18.51585851	0.917945794	4	18.18285725	Quantified
Q8C166	Copine-1	Cpne1	FUNCTION: Calcium-dependent phospholipic	Copine-1 OS=Mus musculu	1.31	1	1	1	536	58.84936568	5.655761719	1.002400805	1		1.043918789	1		0.988485727	1		1.034774624	1		0.975925716	1		0.962181968	1		Quantified
Q8BFS6	Serine/threonine-protein phosphatase CPPED1	Cpped1	FUNCTION: Protein phosphatase that dephos	Serine/threonine-protein ph	2.88	1	1	1	312	35.22543681	5.338378906	1.028878845	1		1.210666263	1		0.967685264	1		0.678662716	1		0.831426585	1		1.224608688	1		Quantified
Q9EPU4	Cleavage and polyadenylation specificity factor subu	Cpsf1	FUNCTION: Component of the cleavage and	Cleavage and polyadenylat	0.76	1	1	1	1441	160.7159713	6.392089844	1.07240137	1		1.041484029	1		0.899851313	1		0.960945175	1		0.909010556	1		1.108349999	1		Quantified
Q64735	Complement component receptor 1-like protein	Cr1l	FUNCTION: Acts as a cofactor for compleme	Complement component re	2.28	1	1	1	483	53.72762283	6.653808594	0.993365749	1		0.94182395	1		0.986285094	1		0.999092408	1		1.134354665	1		1.028094597	1		Quantified
P23927	Alpha-crystallin B chain	Cryab	FUNCTION: May contribute to the transparen	Alpha-crystallin B chain OS	5.14	1	1	1	175	20.05640968	7.327636719	1.028608953	1		1.052763188	1		0.916116791	1		1.000599993	1		0.957105745	1		1.053664362	1		Quantified
P41241	Tyrosine-protein kinase CSK	Csk	FUNCTION: Non-receptor tyrosine-protein kin	Tyrosine-protein kinase CS	1.56	1	1	1	450	50.68378825	7.063964844	0.96875282	1		0.972703323	1		0.986678719	1		0.908996675	1		0.962284116	1		1.214683363	1		Quantified
Q9CZ04	COP9 signalosome complex subunit 7a	Cops7a	FUNCTION: Component of the COP9 signalo	COP9 signalosome comple	7.64	2	2	2	275	30.20595139	7.869628906	0.981806084	2	4.842769698	0.96515267	2	2.389652287	0.921332113	2	5.075133829	1.069158321	2	6.465672159	1.109444844	2	2.426735598	1.029305267	2	0.913159712	Quantified
Q62059	Versican core protein	Vcan	FUNCTION: May play a role in intercellular si	Versican core protein OS=M	0.21	1	1	1	3357	366.5597917	4.640136719	0.914079458	1		1.104776781	1		0.965444484	1		0.875866653	1		1.170015499	1		1.058982206	1		Quantified
Q99LI7	Cleavage stimulation factor subunit 3	Cstf3	FUNCTION: One of the multiple factors requir	Cleavage stimulation factor	1.26	1	1	1	717	82.82372157	8.118652344	1.059599147	1		1.027014643	1		1.011397073	1		0.979093269	1		0.9229917	1		0.983766994	1		Quantified
Q9EPL2	Calsyntenin-1	Clstn1	FUNCTION: Induces KLC1 association with v	Calsyntenin-1 OS=Mus mu	1.02	1	1	1	979	108.8314554	4.919433594	0.961965246	1		1.011620951	1		1.173872279	1		0.901176101	1		0.969270768	1		0.969458049	1		Quantified
Q7TSG2	RNA polymerase II subunit A C-terminal domain phos	Ctdp1	FUNCTION: Processively dephosphorylates '	RNA polymerase II subunit	1.56	1	1	1	960	104.4895227	5.389160156	1.027511775	1		0.995189049	1		0.935990545	1		1.161501811	1		0.922089617	1		0.9524994	1		Quantified
P30999	Catenin delta-1	Ctnnd1	FUNCTION: Key regulator of cell-cell adhesio	Catenin delta-1 OS=Mus m	3.2	2	2	2	938	104.8599677	6.873535156	0.967677854	2	1.628739411	0.962660974	2	4.128887797	1.022098683	2	2.724994122	1.016088009	2	13.13611933	1.002087275	2	3.412236555	1.04571991	2	10.64549453	Quantified
Q9D5V5	Cullin-5	Cul5	FUNCTION: Core component of multiple SCF	Cullin-5 OS=Mus musculus	1.54	1	1	1	780	90.91628953	7.811035156	1.065503282	1		0.884976936	1		1.058562899	1		0.705858201	1		1.05558618	1		1.280159264	1		Quantified
Q9WTX6	Cullin-1	Cul1	FUNCTION: Core component of multiple culli	Cullin-1 OS=Mus musculus	1.03	1	1	3	776	89.63497753	8.001464844	1.075342536	3	3.800531792	1.001404272	3	3.016969295	0.971747205	3	0.937893579	1.010389208	3	1.235130703	0.8178152	3	1.036397766	1.007545178	3	3.498169847	Quantified
Q7TMB8	Cytoplasmic FMR1-interacting protein 1	Cyfip1	FUNCTION: Component of the CYFIP1-EIF4E	Cytoplasmic FMR1-interact	4.23	5	5	8	1253	145.147763	6.902832031	1.046651754	8	4.278912614	1.031110739	8	12.63268787	0.985003305	8	7.488839609	0.96396739	8	10.36206505	0.921527571	8	13.13346049	1.000946019	8	8.671581827	Quantified
O08967	Cytohesin-3	Cyth3	FUNCTION: Promotes guanine-nucleotide ex	Cytohesin-3 OS=Mus musc	2.01	1	1	1	399	46.25040728	5.541503906	1.044182176	1		0.900131131	1		1.006519169	1		0.987188708	1		1.084135382	1		1.040426918	1		Quantified
Q8CBY8	Dynactin subunit 4	Dctn4	FUNCTION: Could have a dual role in dynein	Dynactin subunit 4 OS=Mus	5.35	2	2	2	467	53.02305784	7.723144531	0.776590745	1		0.979555874	1		0.816641829	1		1.182095176	1		0.963516795	1		1.321576924	1		Quantified
Q99LD8	N(G),N(G)-dimethylarginine dimethylaminohydrolase	Ddah2	FUNCTION: Hydrolyzes N(G),N(G)-dimethyl-l	N(G),N(G)-dimethylarginine	3.51	1	1	1	285	29.62731292	6.011230469	1.055916588	1		1.066266501	1		0.83788557	1		1.013227497	1		1	1		1.060626225	1		Quantified
Q62095	ATP-dependent RNA helicase DDX3Y	Ddx3y	FUNCTION: Probable ATP-dependent RNA h	ATP-dependent RNA helica	3.04	2	2	2	658	73.3822483	7.532714844	1.121567159	2	2.801447546	0.942572154	2	1.804281422	0.899674829	2	23.90795243	1.036699408	2	12.14105131	1.100941569	2	7.60207599	0.956053328	2	1.300773874	Quantified
Q810A7	ATP-dependent RNA helicase DDX42	Ddx42	FUNCTION: ATP-dependent RNA helicase. B	ATP-dependent RNA helica	1.51	2	2	2	929	101.9023365	6.976074219	1.019713872	2	9.531474184	1.06686864	2	9.021147298	0.999770393	2	5.941333061	0.88335702	2	1.992939185	1.17686331	2	50.11082681	0.897473997	2	5.304584233	Quantified
Q8C2K1	Differentially expressed in FDCP 6	Def6	FUNCTION: Phosphatidylinositol 3,4,5-trisph	Differentially expressed in F	1.43	1	1	1	630	73.40842273	6.265136719	0.906759762	1		0.877344448	1		0.891550455	1		1.075378871	1		1.292071496	1		1.124829159	1		Quantified
O54786	DNA fragmentation factor subunit alpha	Dffa	FUNCTION: Inhibitor of the caspase-activate	DNA fragmentation factor su	9.06	2	2	2	331	36.54970311	4.906738281	1.021972714	2	7.968146289	1.001797124	2	0.922805043	0.991096025	2	4.06681966	1.028618695	2	0.413668209	0.999766493	2	15.54194092	0.971890084	2	2.385984405	Quantified
P49183	Deoxyribonuclease-1	Dnase1	FUNCTION: Serum endocuclease secreted ir	Deoxyribonuclease-1 OS=M	3.17	1	1	1	284	32.00712323	4.919433594	0.877597578	1		1.011284792	1		0.882932293	1		0.952782603	1		1.336389331	1		1.115690658	1		Quantified
Q9QYI3	DnaJ homolog subfamily C member 7	Dnajc7	FUNCTION: Acts as co-chaperone regulating	DnaJ homolog subfamily C	1.42	1	1	1	494	56.43960686	6.493652344	0.963335163	1		1.046809945	1		1.113611271	1		0.926965121	1		0.965562719	1		0.973650986	1		Quantified
Q8BUR4	Dedicator of cytokinesis protein 1	Dock1	FUNCTION: Involved in cytoskeletal rearrang	Dedicator of cytokinesis pro	0.38	1	1	1	1865	214.9486753	7.620605469	0.915015913	1		0.999554032	1		0.973544766	1		1.008102	1		1.066971505	1		1.091905472	1		Quantified
Q8C147	Dedicator of cytokinesis protein 8	Dock8	FUNCTION: Guanine nucleotide exchange fa	Dedicator of cytokinesis pro	0.33	1	1	1	2100	238.8264217	6.961425781	0.966628644	1		1.017334015	1		1.059911583	1		0.9529031	1		0.979977161	1		1.028750397	1		Quantified
O35098	Dihydropyrimidinase-related protein 4	Dpysl4	FUNCTION: Necessary for signaling by class	Dihydropyrimidinase-relatec	2.8	1	1	1	572	61.92247857	6.976074219	1.081909513	1		1.071172434	1		0.822648601	1		0.949155187	1		1.030797815	1		1.092349805	1		Quantified
Q6NT99	Dual specificity protein phosphatase 23	Dusp23	FUNCTION: Protein phosphatase that mediat	Dual specificity protein phos	5.33	1	1	1	150	16.62656575	8.206542969	0.982171737	1		0.846454145	1		0.85777889	1		0.896632627	1		1.308709366	1		1.291249821	1		Quantified
Q8BL66	Early endosome antigen 1	Eea1	FUNCTION: Binds phospholipid vesicles con	Early endosome antigen 1 (	2.27	4	4	4	1411	160.8170795	5.770019531	0.983965408	4	3.236174274	0.985994607	4	4.350278531	1.012621813	4	6.690835451	1.006535753	4	3.859245907	1.005272874	4	8.447469769	1.040556092	4	3.065444093	Quantified
P57776	Elongation factor 1-delta	Eef1d	FUNCTION: [Isoform 1]: EF-1-beta and EF-1-	Elongation factor 1-delta OS	4.27	1	1	2	281	31.27388418	5.020996094	1.0538463	2	5.134923011	0.981636696	2	2.483055323	0.979809115	2	10.37235361	0.944280665	2	1.079138887	1.073805682	2	12.21361726	1.012761436	2	9.924699828	Quantified
Q3UGC7	Eukaryotic translation initiation factor 3 subunit J-A	Eif3j1	FUNCTION: Component of the eukaryotic trar	Eukaryotic translation initiat	8.05	2	2	2	261	29.32557656	4.805175781	0.978739516	2	5.937518142	1.054596561	2	4.400155934	0.982728821	2	17.23234536	0.950963292	2	0.284728955	0.979673297	2	0.560513537	1.054594512	2	8.16604034	Quantified
P23116	Eukaryotic translation initiation factor 3 subunit A	Eif3a	FUNCTION: RNA-binding component of the e	Eukaryotic translation initiat	3.2	6	6	7	1344	161.8382246	6.770996094	1.022047677	6	6.026959887	1.072135509	6	9.956261879	1.030799925	6	4.627954316	0.930828723	6	5.044290015	0.906394335	6	8.064374171	0.970559807	6	13.14747111	Quantified
Q8JZQ9	Eukaryotic translation initiation factor 3 subunit B	Eif3b	FUNCTION: RNA-binding component of the e	Eukaryotic translation initiat	2.74	2	2	2	803	91.31314281	5.020996094	0.946497635	2	0.99608459	1.108534211	2	0.067613763	1.08272631	2	10.46346432	0.922821571	2	6.471320952	0.938891197	2	9.650489714	0.973302688	2	0.280688291	Quantified
Q9Z1D1	Eukaryotic translation initiation factor 3 subunit G	Eif3g	FUNCTION: RNA-binding component of the e	Eukaryotic translation initiat	4.06	1	1	1	320	35.61592168	5.896972656	1.058908391	1		0.971673154	1		0.934220791	1		1.028695614	1		1.085876893	1		0.984898669	1		Quantified
Q9QZD9	Eukaryotic translation initiation factor 3 subunit I	Eif3i	FUNCTION: Component of the eukaryotic trar	Eukaryotic translation initiat	3.38	1	1	1	325	36.43758861	5.643066406	1.032380973	1		0.984350646	1		0.922752214	1		1.074648388	1		1.036434309	1		0.99442681	1		Quantified
Q9DBZ5	Eukaryotic translation initiation factor 3 subunit K	Eif3k	FUNCTION: Component of the eukaryotic trar	Eukaryotic translation initiat	3.21	1	1	2	218	25.07045202	4.932128906	1.019453614	2	2.927263834	1.03601949	2	1.484268992	1.073724827	2	6.31514865	0.968415127	2	2.145750722	0.92530276	2	7.987980402	0.951997107	2	0.275209596	Quantified
Q05BC3	Echinoderm microtubule-associated protein-like 1	Eml1	FUNCTION: Modulates the assembly and org	Echinoderm microtubule-as	2.58	2	2	2	814	89.62358927	7.020019531	1.087265526	2	1.240833666	1.025749866	2	1.221238711	0.965609126	2	4.617284602	0.967588025	2	7.564481725	0.889570132	2	3.598705907	1.038203336	2	0.429414494	Quantified
P59900	EMILIN-3	Emilin3		EMILIN-3 OS=Mus musculu	1.19	1	1	1	758	82.34532043	6.932128906	0.881468724	1		1.11667201	1		1.142261462	1		0.867660453	1		0.958063692	1		1.014381452	1		Quantified
Q9R1E6	Ectonucleotide pyrophosphatase/phosphodiesterase	Enpp2	FUNCTION: Hydrolyzes lysophospholipids to	Ectonucleotide pyrophosph	0.81	1	1	1	862	98.82148955	7.269042969	1.009218199	1		0.983673553	1		0.946729547	1		1.052333266	1		1.077935951	1		0.989893933	1		Quantified
Q149F3	Eukaryotic peptide chain release factor GTP-binding	Gspt2	FUNCTION: Involved in translation terminatio	Eukaryotic peptide chain re	1.11	1	1	2	632	69.10280447	5.198730469	0.819170438	2	29.69260857	0.803324251	2	27.12325038	1.038489973	2	5.880845014	1.401895469	2	48.63311509	1.030449998	2	8.902365015	0.826358187	2	38.06917859	Quantified
Q9D172	Glutamine amidotransferase-like class 1 domain-con	Gatd3a		Glutamine amidotransferas	6.02	2	2	2	266	28.07278118	8.777832031	1.00513273	2	1.824857269	1.007870603	2	0.561993345	0.996220538	2	0.878999597	1.017197495	2	1.953248478	0.986228917	2	4.533524172	1.001596912	2	1.177369046	Quantified
Q8K0G8	Epithelial splicing regulatory protein 2	Esrp2	FUNCTION: mRNA splicing factor that regula	Epithelial splicing regulator	1.12	1	1	1	717	77.3115222	6.379394531	1.004051244	1		0.9528175	1		0.959242494	1		1.487741874	1		0.731511046	1		0.77868922	1		Quantified
Q3U7R1	Extended synaptotagmin-1	Esyt1	FUNCTION: Binds glycerophospholipids in a	Extended synaptotagmin-1	1.1	1	1	1	1092	121.4784332	5.947753906	1.00403796	1		1.010638795	1		0.982550957	1		1.013076611	1		0.95312322	1		1.039860252	1		Quantified
Q921G7	Electron transfer flavoprotein-ubiquinone oxidoreduct	Etfdh	FUNCTION: Accepts electrons from ETF and	Electron transfer flavoprotei	0.97	1	1	1	616	68.04761266	7.576660156	0.979598677	1		0.922110094	1		0.856272405	1		1.097639446	1		1.073406855	1		1.148240585	1		Quantified
Q8VBV3	Exosome complex component RRP4	Exosc2	FUNCTION: Non-catalytic component of the F	Exosome complex compon	3.07	1	1	1	293	32.61103561	7.503417969	1.064933687	1		1.001228621	1		0.976803379	1		1.014879386	1		0.965253969	1		0.984110174	1		Quantified
Q3TCJ1	BRISC complex subunit Abraxas 2	Abraxas2	FUNCTION: Component of the BRISC comple	BRISC complex subunit Ab	1.93	1	1	1	415	46.91391591	6.176269531	1.030584217	1		0.959240149	1		1.050029515	1		0.955548011	1		1.089115863	1		0.969113178	1		Quantified
Q6DTY7	6-phosphofructo-2-kinase/fructose-2,6-bisphosphatas	Pfkfb4	FUNCTION: Synthesis and degradation of fru	6-phosphofructo-2-kinase/fr	2.13	1	1	1	469	54.03228544	6.480957031	1.054433692	1		0.930653812	1		1.051559675	1		0.985319373	1		0.974486908	1		1.012562022	1		Quantified
Q9DCL2	Cytosolic iron-sulfur assembly component 2A	Ciao2a	FUNCTION: Component of the cytosolic iron-	Cytosolic iron-sulfur assem	6.88	1	1	1	160	18.40639537	4.817871094	0.996845432	1		0.943510548	1		1.065075233	1		0.895859523	1		1.086993629	1		1.066808826	1		Quantified
P23591	GDP-L-fucose synthase	Gfus	FUNCTION: Catalyzes the two-step NADP-de	GDP-L-fucose synthase OS	2.18	1	1	1	321	35.85501596	6.741699219	0.939889631	1		0.999164062	1		0.975604738	1		1.127866256	1		0.991799264	1		0.98690389	1		Quantified
Q91WF7	Polyphosphoinositide phosphatase	Fig4	FUNCTION: The PI(3,5)P2 regulatory comple	Polyphosphoinositide phos	0.77	1	1	1	907	103.3811996	6.976074219	0.983643131	1		0.969231849	1		0.956928958	1		1.115521179	1		0.967748712	1		1.022631809	1		Quantified
Q62446	Peptidyl-prolyl cis-trans isomerase FKBP3	Fkbp3	FUNCTION: FK506- and rapamycin-binding p	Peptidyl-prolyl cis-trans isor	4.91	1	1	2	224	25.13229158	9.275878906	0.981526267	2	2.717383165	0.956281686	2	0.290694186	0.983109669	2	2.073517633	1.060516575	2	8.543702121	1.084694844	2	0.852978043	0.992687459	2	4.42141784	Quantified
Q9JL26	Formin-like protein 1	Fmnl1	FUNCTION: Plays a role in the regulation of c	Formin-like protein 1 OS=M	0.64	1	1	1	1094	121.9828724	5.820800781	1.020676358	1		1.012233226	1		0.920304327	1		1.181994421	1		0.974515607	1		0.906217363	1		Quantified
P50285	Dimethylaniline monooxygenase [N-oxide-forming] 1	Fmo1	FUNCTION: This protein is involved in the ox	Dimethylaniline monooxyge	3.2	2	2	3	532	59.87595639	8.470214844	0.964726984	3	2.286409368	0.990344035	3	1.675667281	1.04908248	3	4.938560617	0.9873974	3	6.175829594	1.065007648	3	4.852019644	0.933771029	3	1.04743141	Quantified
Q9WV91	Prostaglandin F2 receptor negative regulator	Ptgfrn	FUNCTION: Inhibits the binding of prostaglan	Prostaglandin F2 receptor r	0.8	1	1	1	879	98.65972501	6.609863281	0.954927895	1		1.157224799	1		0.914258487	1		0.916295886	1		1.181014501	1		0.968783382	1		Quantified

Q91XD4	Formimidoyltransferase-cyclodeaminase	Ftcd	FUNCTION: Folate-dependent enzyme, that c	Formimidoyltransferase-cyc	1.85	1	1	1	541	58.90153798	6.112792969	0.808955654	1		0.965069283	1		1.127158334	1		0.912167975	1		1.045053334	1		1.176966247	1		Quantified
Q99LJ1	Tissue alpha-L-fucosidase	Fuca1	FUNCTION: Alpha-L-fucosidase is responsibl	Tissue alpha-L-fucosidase (	1.33	1	1	1	452	52.24719261	6.946777344	0.943903321	1		0.938312663	1		0.956137246	1		0.938717238	1		1.400393339	1		1.019646526	1		Quantified
P97855	Ras GTPase-activating protein-binding protein 1	G3bp1	FUNCTION: ATP- and magnesium-dependen	Ras GTPase-activating prot	3.44	1	1	1	465	51.79689111	5.592285156	0.997846445	1		0.9989569	1		0.981098066	1		1.055025947	1		0.97344612	1		1.005818859	1		Quantified
Q8CFX1	GDH/6PGL endoplasmic bifunctional protein	H6pd	FUNCTION: Bifunctional enzyme localized in	GDH/6PGL endoplasmic bi	1.01	1	1	1	789	88.87174202	6.932128906	0.995276529	1		1.099528025	1		1.045515548	1		0.965295962	1		0.964249165	1		0.921245334	1		Quantified
Q8BMP6	Golgi resident protein GCP60	Acbd3	FUNCTION: Involved in the maintenance of G	Golgi resident protein GCP6	5.9	2	2	3	525	60.14381773	5.109863281	1.018905008	3	1.70591731	0.943523306	3	3.713594715	1.056396552	3	1.659567037	1.012428119	3	0.671910709	1.134379129	3	12.65134809	0.93992678	3	4.3658589	Quantified
Q61599	Rho GDP-dissociation inhibitor 2	Arhgdib	FUNCTION: Regulates the GDP/GTP exchan	Rho GDP-dissociation inhib	21	3	3	3	200	22.83649276	5.109863281	0.947252944	3	8.094211881	1.001786324	3	2.676085328	1.016837179	3	8.326901274	1.032885215	3	2.969154149	0.919747527	3	0.940982167	0.995921584	3	2.732684316	Quantified
Q6P5E6	ADP-ribosylation factor-binding protein GGA2	Gga2	FUNCTION: Plays a role in protein sorting an	ADP-ribosylation factor-binc	1.99	1	1	1	603	66.00737517	7.298339844	0.997224385	1		0.969255016	1		0.987766886	1		1.021579962	1		0.943663386	1		1.083748961	1		Quantified
Q9CQM9	Glutaredoxin-3	Glrx3	FUNCTION: Together with BOLA2, acts as a	Glutaredoxin-3 OS=Mus mu	3.56	1	1	1	337	37.75437384	5.592285156	1.142243181	1		0.910696591	1		0.894404074	1		1.110838368	1		0.930682106	1		1.020013269	1		Quantified
O08795	Glucosidase 2 subunit beta	Prkcsh	FUNCTION: Regulatory subunit of glucosidas	Glucosidase 2 subunit beta	3.65	2	2	2	521	58.75575461	4.462402344	1.009813185	2	3.516651355	0.939633815	2	1.843262948	0.990693067	2	4.924632863	1.029148217	2	8.088261609	1.078254966	2	4.905387326	1.008441024	2	0.495752426	Quantified
Q8K0C9	GDP-mannose 4,6 dehydratase	Gmds	FUNCTION: Catalyzes the conversion of GDF	GDP-mannose 4,6 dehydra	2.42	1	1	1	372	41.95822567	7.034667969	0.996923689	1		0.937199922	1		0.914448918	1		1.102098265	1		0.960959431	1		1.110305928	1		Quantified
Q922H4	Mannose-1-phosphate guanyltransferase alpha	Gmppa	FUNCTION: May serve as a regulatory subun	Mannose-1-phosphate guar	1.67	1	1	1	420	46.21513331	7.620605469	0.980840378	1		0.989623721	1		0.969123674	1		0.965577559	1		1.112887417	1		1.055038082	1		Quantified
Q99L27	GMP reductase 2	Gmpr2	FUNCTION: Catalyzes the irreversible NADP	GMP reductase 2 OS=Mus	2.3	1	1	1	348	37.99405542	7.444824219	0.949614348	1		0.982102276	1		1.042668364	1		0.934604375	1		1.023460407	1		1.097603942	1		Quantified
O70325	Phospholipid hydroperoxide glutathione peroxidase	Gpx4	FUNCTION: Essential antioxidant peroxidase	Phospholipid hydroperoxide	4.06	1	1	1	197	22.21485116	8.455566406	1.028475492	1		1.033021041	1		0.989591794	1		0.949825271	1		1.028849417	1		1.000692891	1		Quantified
Q8VD04	GRIP1-associated protein 1	Gripap1	FUNCTION: Regulates the endosomal recycli	GRIP1-associated protein 1	2.98	2	2	2	806	92.65837426	5.249511719	0.981902874	2	11.40025387	0.957569684	2	6.216488826	0.961481373	2	6.531793534	1.045758154	2	2.855352866	1.012639365	2	8.647531277	1.068506652	2	8.315937375	Quantified
Q91Z53	Glyoxylate reductase/hydroxypyruvate reductase	Grhpr	FUNCTION: Enzyme with hydroxy-pyruvate r	Glyoxylate reductase/hydro	3.05	1	1	1	328	35.30647719	7.649902344	0.943448379	1		1.078081186	1		1.054984304	1		0.943578833	1		0.975295976	1		1.00378658	1		Quantified
Q9D8T2	Gasdermin-D	Gsdmd	FUNCTION: [Gasdermin-D, N-terminal]: Prom	Gasdermin-D OS=Mus mus	1.64	1	1	1	487	53.20428296	5.097167969	1.018251409	1		0.967892715	1		1.064497091	1		0.954029176	1		1.02879481	1		0.993770206	1		Quantified
P97494	Glutamate--cysteine ligase catalytic subunit	Gclc		Glutamate--cysteine ligase	1.41	1	1	1	637	72.5250727	5.896972656	0.971970055	1		0.99968014	1		1.052261959	1		0.987521947	1		0.921275485	1		1.05131737	1		Quantified
P51855	Glutathione synthetase	Gss		Glutathione synthetase OS	1.9	1	1	1	474	52.21395341	5.795410156	0.973067618	1		0.993181543	1		1.027983116	1		0.981719999	1		1.001369092	1		1.0427666	1		Quantified
Q64471	Glutathione S-transferase theta-1	Gstt1	FUNCTION: Conjugation of reduced glutathio	Glutathione S-transferase th	2.92	1	1	1	240	27.35633673	7.269042969	0.977820596	1		0.98395598	1		0.982980565	1		1.048959945	1		1.040286855	1		1.007159435	1		Quantified
Q61133	Glutathione S-transferase theta-2	Gstt2	FUNCTION: Conjugation of reduced glutathio	Glutathione S-transferase th	6.15	1	1	1	244	27.61659104	7.547363281	0.952987195	1		1.049180383	1		0.987648649	1		1.00098577	1		1.036018311	1		1.007277694	1		Quantified
P51859	Hepatoma-derived growth factor	Hdgf	FUNCTION: Acts as a transcriptional repress	Hepatoma-derived growth fa	3.8	1	1	1	237	26.25264293	4.830566406	1.031272591	1		0.815725837	1		1.036874021	1		1.099685496	1		1.190752709	1		0.933588903	1		Quantified
Q9R257	Heme-binding protein 1	Hebp1	FUNCTION: May bind free porphyrinogens th	Heme-binding protein 1 OS	8.42	1	1	1	190	21.0533555	5.262207031	0.938709951	1		1.054119644	1		1.049940389	1		0.92270981	1		1.055851726	1		1.013829454	1		Quantified
P36552	Oxygen-dependent coproporphyrinogen-III oxidase, r	Cpox	FUNCTION: Involved in the heme biosynthes	Oxygen-dependent coproporc	2.03	1	1	1	443	49.68284184	8.528808594	1.041220728	1		0.990176936	1		1.026975262	1		1.006243402	1		0.898701032	1		1.012778307	1		Quantified
O54879	High mobility group protein B3	Hmgb3	FUNCTION: Multifunctional protein with vario	High mobility group protein	10	1	2	2	200	22.99545351	8.367675781	0.964384948	1		0.979557687	1		1.074708038	1		1.007250538	1		1.005486495	1		0.985134161	1		Quantified
Q9QUP5	Hyaluronan and proteoglycan link protein 1	Hapln1	FUNCTION: Stabilizes the aggregates of prot	Hyaluronan and proteoglyca	2.25	1	1	1	356	40.45216468	7.796386719	1.042641889	1		1.040414462	1		1.0270249	1		0.955762683	1		0.988134365	1		0.953538382	1		Quantified
P35492	Histidine ammonia-lyase	Hal		Histidine ammonia-lyase O	7	4	4	5	657	72.21240189	6.341308594	1.009662172	5	3.252793312	1.040857054	5	6.457177182	0.948681203	5	4.446771316	0.988132167	5	2.369673816	0.984266972	5	2.311428676	1.01705108	5	5.086336368	Quantified
P34914	Bifunctional epoxide hydrolase 2	Ephx2	FUNCTION: Bifunctional enzyme. The C-term	Bifunctional epoxide hydrol	2.17	1	1	1	554	62.47460301	6.188964844	1.011293041	1		0.978678548	1		0.99706026	1		1.019538117	1		0.95667835	1		1.042338075	1		Quantified
P51125	Calpastatin	Cast	FUNCTION: Specific inhibition of calpain (cal	Calpastatin OS=Mus muscu	1.65	1	1	2	788	84.87064884	5.516113281	1.029813351	2	3.845897769	1.010650115	2	1.139024805	1.039451557	2	3.549418257	0.994060622	2	1.362228668	0.9597859	2	6.845263965	0.962152411	2	1.412245499	Quantified
P13597	Intercellular adhesion molecule 1	Icam1	FUNCTION: ICAM proteins are ligands for the	Intercellular adhesion mole	1.3	1	1	1	537	58.80719037	6.125488281	0.933209804	1		0.889135857	1		1.097744977	1		1.019708031	1		1.098796514	1		1.022508639	1		Quantified
Q9JHR7	Insulin-degrading enzyme	Ide	FUNCTION: Plays a role in the cellular break	Insulin-degrading enzyme (	1.77	2	2	2	1019	117.6971444	6.536621094	0.948087806	2	8.243865167	0.991663705	2	2.060057842	1.046065097	2	17.79842625	0.988033261	2	17.14701746	1.034456778	2	1.627171786	1.006113896	2	5.496674213	Quantified
Q60872	Eukaryotic translation initiation factor 1A	Eif1a	FUNCTION: Seems to be required for maxima	Eukaryotic translation initiat	15.28	2	2	2	144	16.49224745	5.236816406	1.000540374	2	0.417312992	1.035844485	2	6.814537224	1.052321176	2	0.251205799	0.958605206	2	0.628950621	1.056154662	2	2.953966978	0.928076556	2	4.919002766	Quantified
Q05D44	Eukaryotic translation initiation factor 5B	Eif5b	FUNCTION: Plays a role in translation initiatic	Eukaryotic translation initiat	0.66	1	1	1	1216	137.5319566	5.592285156	0.992029325	1		1.123326659	1		1.00055907	1		0.973998268	1		0.920357306	1		0.967023947	1		Quantified
P63073	Eukaryotic translation initiation factor 4E	Eif4e	FUNCTION: Recognizes and binds the 7-met	Eukaryotic translation initiat	3.69	1	1	1	217	25.03748677	6.150878906	0.947316367	1		0.940092662	1		0.984449471	1		1.101090121	1		0.901788516	1		1.115793654	1		Quantified
Q62448	Eukaryotic translation initiation factor 4 gamma 2	Eif4g2	FUNCTION: Appears to play a role in the swit	Eukaryotic translation initiat	1.99	2	2	2	906	102.0407185	7.137207031	1.018481215	2	2.951821999	1.034546344	2	2.931844349	0.988206279	2	0.181050156	0.962367194	2	2.318506852	0.938819314	2	3.644084432	1.051199122	2	4.116207606	Quantified
Q9WUK2	Eukaryotic translation initiation factor 4H	Eif4h	FUNCTION: Stimulates the RNA helicase act	Eukaryotic translation initiat	3.63	1	1	1	248	27.32440075	7.225097656	1.020786687	1		1.055642621	1		1.00088511	1		1.036399149	1		1.015610279	1		0.890411113	1		Quantified
Q61730	Interleukin-1 receptor accessory protein	Il1rap	FUNCTION: Coreceptor for IL1RL2 in the IL-3	Interleukin-1 receptor acces	2.11	1	1	2	570	65.69856227	7.767089844	0.939858003	2	2.847525258	0.979262082	2	0.211267451	1.030764611	2	10.90566595	1.024334053	2	5.336628189	0.929835909	2	1.857529831	1.084382581	2	5.22640553	Quantified
P24547	Inosine-5'-monophosphate dehydrogenase 2	Impdh2	FUNCTION: Catalyzes the conversion of inos	Inosine-5'-monophosphate	1.56	1	1	1	514	55.77977208	7.283691406	1.063645462	1		1.046422655	1		0.977420904	1		0.961024836	1		0.884953615	1		1.038105902	1		Quantified
Q80V26	Golgi-resident adenosine 3',5'-bisphosphate 3'-phosp	Bpnt2	FUNCTION: Exhibits 3'-nucleotidase activity t	Golgi-resident adenosine 3'	4.49	2	2	2	356	38.59195043	6.468261719	1.022498972	2	2.238889376	1.043889166	2	4.841816495	0.995321312	2	3.79959025	0.874341109	2	6.630078249	1.067872265	2	9.739495356	1.038614032	2	1.385055377	Quantified
Q9JHU9	Inositol-3-phosphate synthase 1	Isyna1	FUNCTION: Key enzyme in myo-inositol bios	Inositol-3-phosphate syntha	1.44	1	1	1	557	60.89313133	6.417480469	1.020843835	1		0.997484843	1		0.978013942	1		1.045188082	1		0.931613457	1		1.022150515	1		Quantified
Q91YE6	Importin-9	Ipo9	FUNCTION: Functions in nuclear protein impo	Importin-9 OS=Mus muscul	0.86	1	1	1	1041	115.9777366	4.805175781	1.027375559	1		1.033409412	1		1.13278704	1		0.882832228	1		1.043485636	1		0.898016752	1		Quantified
Q64287	Interferon regulatory factor 4	Irf4	FUNCTION: Transcriptional activator. Binds t	Interferon regulatory factor	2.22	1	1	1	450	51.54442408	6.990722656	1.025062462	1		0.957597874	1		1.113561585	1		0.893343361	1		0.987102049	1		1.029221247	1		Quantified
Q9CX00	IST1 homolog	Ist1	FUNCTION: ESCRT-III-like protein involved ir	IST1 homolog OS=Mus mu	2.49	1	1	1	362	39.44347137	5.439941406	1.015785795	1		1.069697573	1		0.888251286	1		0.972997294	1		1.052638921	1		1.050824596	1		Quantified
P11881	Inositol 1,4,5-trisphosphate receptor type 1	Itpr1	FUNCTION: Intracellular channel that mediate	Inositol 1,4,5-trisphosphate	0.29	1	1	1	2749	312.9681491	6.036621094	1.001201468	1		0.925419829	1		0.956635342	1		1.072240391	1		1.095002468	1		1.020740529	1		Quantified
O88792	Junctional adhesion molecule A	F11r	FUNCTION: Seems to play a role in epithelial	Junctional adhesion molecu	3.67	1	1	1	300	32.40309671	6.770996094	1.000190657	1		1.077211524	1		1.002720907	1		0.968348021	1		1.017804702	1		0.954174043	1		Quantified
Q9D3R6	Katanin p60 ATPase-containing subunit A-like 2	Katnal2	FUNCTION: Severs microtubules in vitro in a	Katanin p60 ATPase-contai	3.53	1	2	3	539	61.11462588	7.518066406	1.029813615	2	2.288234265	0.973305552	2	4.372329384	0.993924572	2	2.764292419	0.957403584	2	2.402958879	1.111981707	2	2.678460163	1.001056498	2	4.805306711	Quantified
Q9DBP5	UMP-CMP kinase	Cmpk1	FUNCTION: Catalyzes the phosphorylation o	UMP-CMP kinase OS=Mus	5.1	1	1	1	196	22.15129618	5.833496094	0.989848186	1		0.933240477	1		0.978148745	1		1.065975949	1		1.182442179	1		0.953291594	1		Quantified
B1AVY7	Kinesin-like protein KIF16B	Kif16b	FUNCTION: Plus end-directed microtubule-de	Kinesin-like protein KIF16B	0.76	1	1	1	1312	149.9651892	6.442871094	0.977435958	1		1.011099411	1		1.126120937	1		0.903637608	1		0.977104578	1		1.001026313	1		Quantified
Q8C078	Calcium/calmodulin-dependent protein kinase kinase	Camkk2	FUNCTION: Calcium/calmodulin-dependent p	Calcium/calmodulin-depenc	2.38	1	1	1	588	64.57649697	5.896972656	1.044758379	1		1.10483416	1		0.996931388	1		0.886633288	1		1.070863752	1		0.935299296	1		Quantified
P68404	Protein kinase C beta type	Prkcb	FUNCTION: Calcium-activated, phospholipid-	Protein kinase C beta type	1.04	1	1	1	671	76.70104358	7.005371094	0.951510871	1		0.943075164	1		1.006061723	1		0.985191717	1		0.903248853	1		1.201009554	1		Quantified
Q61789	Laminin subunit alpha-3	Lama3	FUNCTION: Binding to cells via a high affinity	Laminin subunit alpha-3 OS	1.98	7	7	7	3330	365.9916266	6.653808594	0.993706653	7	10.64836508	0.977646763	7	10.95763557	0.952043773	7	4.185284996	1.072128038	7	12.52296655	0.995710667	7	6.157415569	0.986437804	7	4.81484335	Quantified
O89112	Glutathione S-transferase LANCL1	Lancl1	FUNCTION: Functions as glutathione transfer	Glutathione S-transferase L	2.01	1	1	1	399	45.31164682	7.767089844	0.975143949	1		1.036949126	1		1.080516121	1		0.943590744	1		0.953117058	1		1.000397421	1		Quantified
Q7TSJ6	Serine/threonine-protein kinase LATS2	Lats2	FUNCTION: Negative regulator of YAP1 in th	Serine/threonine-protein kir	0.67	1	1	1	1042	115.399856	8.221191406	0.899522784	1		0.943351359	1		0.874125	1		1.113448715	1		1.13426852	1		1.13529059	1		Quantified
P32067	Lupus La protein homolog	Ssb	FUNCTION: Binds to the 3' poly(U) terminus c	Lupus La protein homolog (	3.86	2	2	2	415	47.72696109	9.773925781	0.929643388	2	0.928946792	0.991944937	2	10.26623848	0.968283306	2	18.72312378	1.039645465	2	8.144519243	1.026907738	2	16.10695786	1.063187143	2	11.60072891	Quantified
Q5SUF2	Luc7-like protein 3	Luc7l3	FUNCTION: Binds cAMP regulatory element	Luc7-like protein 3 OS=Mus	1.85	1	1	1	432	51.41912836	9.773925781	1.009992771	1		1.029222574	1		1.051032407	1		0.94743752	1		1.019159221	1		0.962496461	1		Quantified
Q07797	Galectin-3-binding protein	Lgals3bp	FUNCTION: Promotes integrin-mediated cell	Galectin-3-binding protein (	3.12	1	1	2	577	64.4496494	5.135253906	0.964166258	2	9.844934581	1.031212145	2	0.26871003	1.092149244	2	17.54566249	0.914842574	2	1.505295502	0.95512795	2	11.68161974	1.020125512	2	3.939810175	Quantified
O89017	Legumain	Lgmn	FUNCTION: Has a strict specificity for hydrol	Legumain OS=Mus muscul	2.76	1	1	1	435	49.34130468	6.392089844	1.000081594	1		0.987305926	1		0.89229064	1		1.019594578	1		1.066597485	1		1.097645143	1		Quantified
Q8BFW7	Lipoma-preferred partner homolog	Lpp	FUNCTION: May play a structural role at sites	Lipoma-preferred partner hc	1.47	1	1	1	613	65.84791936	7.371582031	1.000789104	1		1.062802315	1		0.961150048	1		1.021550802	1		1.007938055	1		0.968385888	1		Quantified
Q99N69	Leupaxin	Lpxn	FUNCTION: Transcriptional coactivator for an	Leupaxin OS=Mus musculu	4.4	1	1	1	386	43.44954621	6.430175781	0.96122536	1		1.228564311	1		0.953464603	1		0.966068938	1		0.895804293	1		0.95939747	1		Quantified
Q9ESE1	Lipopolysaccharide-responsive and beige-like ancho	Lrba	FUNCTION: May be involved in coupling sign	Lipopolysaccharide-respons	0.74	3	3	3	2856	316.8627944	5.693847656	1.027204979	3	3.350513855	0.989339475	3	1.918013659	0.953362126	3	1.114327696	0.994472628	3	2.158786828	1.049984895	3	8.107954401	1.001945173	3	7.310735476	Quantified
Q922Q8	Leucine-rich repeat-containing protein 59	Lrrc59	FUNCTION: Required for nuclear import of F(	Leucine-rich repeat-contain	2.61	1	1	1	307	34.85577234	9.524902344	0.928812358	1		1.089717685	1		0.799835644	1		1.069427038	1		1.088149548	1		1.100301675	1		Quantified
Q3UZ39	Leucine-rich repeat flightless-interacting protein 1	Lrrfip1	FUNCTION: Transcriptional repressor which p	Leucine-rich repeat flightles	1.65	1	1	1	729	79.20122221	4.817871094	1.03300167	1		0.969058892	1		1.052790127	1		1.020618603	1		1.077184138	1		0.893316038	1		Quantified
Q9QXA5	U6 snRNA-associated Sm-like protein LSm4	Lsm4	FUNCTION: Plays role in pre-mRNA splicing	U6 snRNA-associated Sm-l	5.11	1	1	1	137	15.06670183	10.05224609	0.956430966	1		1.010610824	1		1.14993289	1		0.879601664	1		1.066635032	1		0.967105891	1		Quantified
Q8CG19	Latent-transforming growth factor beta-binding protein	Ltbp1	FUNCTION: Key regulator of transforming grc	Latent-transforming growth	0.47	1	1	1	1712	186.5931451	6.023925781	1.034247808	1		1.031922378	1		1.002424414	1		0.991473022	1		0.984528696	1		0.965381149	1		Quantified
Q9CYI4	Putative RNA-binding protein Luc7-like 1	Luc7l	FUNCTION: May bind to RNA via its Arg/Ser-	Putative RNA-binding prote	4.04	1	1	1	371	43.9074131	9.876464844	0.989049336	1		1.000683792	1		0.949087658	1		1.00044728	1		1.031878873	1		1.07014915	1		Quantified
A2AHG0	Leucine zipper putative tumor suppressor 3	Lzts3	FUNCTION: May be involved in promoting the	Leucine zipper putative tum	1.14	1	1	1	700	74.93973285	6.990722656	1.055122593	1		0.990226398	1		0.990030547	1		1.005888917	1		0.997652693	1		0.981149954	1		Quantified
O08663	Methionine aminopeptidase 2	Metap2	FUNCTION: Cotranslationally removes the N-	Methionine aminopeptidase	2.93	1	1	1	478	52.88825695	5.820800781	1.002878195	1		0.841116983	1		0.957632306	1		0.883389518	1		1.594104943	1		1.003814983	1		Quantified
P27546	Microtubule-associated protein 4	Map4	FUNCTION: Non-neuronal microtubule-assoc	Microtubule-associated pro	1.51	1	1	1	1125	117.3569972	4.982910156	1.008157734	1		0.994316409	1		0.973962183	1		1.039192551	1		0.9916791	1		1.013517195	1		Quantified
Q61166	Microtubule-associated protein RP/EB family membe	Mapre1	FUNCTION: Plus-end tracking protein (+TIP)	Microtubule-associated pro	3.73	1	1	3	268	29.9972252	5.224121094	1.015115156	3	1.907340339	0.98899907	3	0.539034564	1.045602598	3	1.052408484	1.00770036	3	1.788149542	0.969732512	3	5.175233706	0.966104081	3	2.832008825	Quantified
Q99LB6	Methionine adenosyltransferase 2 subunit beta	Mat2b	FUNCTION: Regulatory subunit of S-adenosy	Methionine adenosyltransfe	2.99	1	1	1	334	37.36898765	6.946777344	1.006632985	1		1.028269422	1		0.92710153	1		1.0263628	1		1.088272793	1		0.985985761	1		Quantified
O89029	Matrilin-4	Matn4	FUNCTION: Major component of the extracell	Matrilin-4 OS=Mus musculu	1.44	1	1	2	624	68.87387861	5.757324219	1.080793368	2	3.473067711	1.095439202	2	1.155244363	0.925373213	2	0.204417722	0.86853294	2	3.438618653	0.88947549	2	9.545936446	1.115234538	2	2.345930961	Quantified
O35701	Matrilin-3	Matn3	FUNCTION: Major component of the extracell	Matrilin-3 OS=Mus musculu	4.16	1	1	1	481	51.81159508	7.474121094	0.983752513	1		0.947863734	1		0.888660935	1		1.04074567	1		1.025557066	1		1.165457805	1		Quantified
Q7TPV4	Myb-binding protein 1A	Mybbp1a	FUNCTION: May activate or repress transcrip	Myb-binding protein 1A OS	0.45	1	1	1	1344	151.9415314	8.953613281	1.045286956	1		1.077993603	1		0.923460236	1		1.01329411	1		0.996899333	1		0.963140911	1		Quantified
P49717	DNA replication licensing factor MCM4	Mcm4	FUNCTION: Acts as component of the MCM2	DNA replication licensing fa	0.81	1	1	1	862	96.67561699	7.195800781	1.039565678	1		0.922054884	1		1.044478176	1		0.978739743	1		0.967757896	1		1.056404675	1		Quantified
Q61881	DNA replication licensing factor MCM7	Mcm7	FUNCTION: Acts as component of the MCM2	DNA replication licensing fa	1.11	1	1	1	719	81.1595054	6.366699219	1.057679963	1		1.06733449	1		1.01760507	1		0.961665477	1		0.837774044	1		1.003193496	1		Quantified
Q91VH6	Protein MEMO1	Memo1	FUNCTION: May control cell migration by rela	Protein MEMO1 OS=Mus m	4.38	1	1	1	297	33.67046604	7.137207031	1.016544467	1		1.005181898	1		1.007278504	1		1.01025978	1		0.953197685	1		1.007517069	1		Quantified
O88559	Menin	Men1	FUNCTION: Essential component of a MLL/S	Menin OS=Mus musculus (	1.96	1	1	1	611	67.45848239	6.392089844	0.896646351	1		0.979639431	1		1.006501633	1		1.092541178	1		1.119712661	1		0.977610013	1		Quantified
Q8VE43	Meteorin-like protein	Metrnl	FUNCTION: Hormone induced following exer	Meteorin-like protein OS=M	2.57	1	1	1	311	34.50743525	7.854980469	1.027217736	1		1.029299759	1		0.989847833	1		0.946139572	1		0.898867046	1		1.087729525	1		Quantified
P55002	Microfibrillar-associated protein 2	Mfap2	FUNCTION: Component of the elastin-associ	Microfibrillar-associated pro	5.46	1	1	1	183	20.56467184	4.894042969	1.02460754	1		1.028138105	1		0.948558194	1		0.977212986	1		0.990091326	1		1.052165605	1		Quantified
P34884	Macrophage migration inhibitory factor	Mif	FUNCTION: Pro-inflammatory cytokine. Invol	Macrophage migration inhib	6.09	1	1	1	115	12.49617837	7.342285156	1.056366323	1		0.878249795	1		0.9778029	1		1.060704574	1		1.086105569	1		1.008304933	1		Quantified
Q8VI63	MOB kinase activator 2	Mob2	FUNCTION: Stimulates the autophosphorylat	MOB kinase activator 2 OS	2.98	1	1	1	235	26.8342621	6.521972656	0.979963519	1		0.995936686	1		1.051402653	1		1.030801832	1		0.985965859	1		0.964996001	1		Quantified
A2BDX3	Adenylyltransferase and sulfurtransferase MOCS3	Mocs3	FUNCTION: Plays a central role in 2-thiolatio	Adenylyltransferase and su	2.17	1	1	2	460	49.34377008	7.488769531	1.016912202	2	2.504344191	0.989864159	2	0.284970589	0.985211278	2	5.779195301	1.011907501	2	2.556440074	0.943173924	2	0.777258379	1.05231615	2	1.242939824	Quantified
P47809	Dual specificity mitogen-activated protein kinase kina	Map2k4	FUNCTION: Dual specificity protein kinase w	Dual specificity mitogen-act	2.27	1	1	1	397	44.08534617	8.074707031	0.961005917	1		0.929668697	1		1.046321488	1		0.954059635	1		1.131034053	1		1.054936116	1		Quantified
Q8VEM8	Phosphate carrier protein, mitochondrial	Slc25a3	FUNCTION: Transport of phosphate groups fr	Phosphate carrier protein, r	2.8	1	1	1	357	39.60645902	9.261230469	1.006503505	1		0.977009565	1		0.914898909	1		1.067533329	1		1.02469874	1		1.052805356	1		Quantified
Q3KNA1	Mas-related G-protein coupled receptor member B2	Mrgprb2	FUNCTION: Mast cell-specific receptor for ba	Mas-related G-protein coup	5.62	1	1	1	338	38.80615915	7.854980469	0.97681787	1		1.001858593	1		0.941523075	1		1.033412904	1		1.025739985	1		1.060250477	1		Quantified
Q99J25	rRNA methyltransferase 1, mitochondrial	Mrm1	FUNCTION: S-adenosyl-L-methionine-depend	rRNA methyltransferase 1,	2.19	1	1	1	320	34.82137805	8.807128906	0.901690235	1		0.945340521	1		0.987438764	1		1.046091216	1		1.044287719	1		1.124718265	1		Quantified
Q9D6Y7	Mitochondrial peptide methionine sulfoxide reductase	Msra	FUNCTION: Has an important function as a re	Mitochondrial peptide methi	3.43	1	1	2	233	25.97182754	8.411621094	0.977562033	2	4.988735683	0.962531669	2	3.193493873	0.978611082	2	8.211381685	1.017405524	2	9.973266946	1.100591138	2	14.7782711	1.023787586	2	2.409521457	Quantified
Q9R190	Metastasis-associated protein MTA2	Mta2	FUNCTION: May be involved in the regulatior	Metastasis-associated prote	1.5	1	1	1	668	74.98265594	9.671386719	1.029312738	1		1.021885762	1		0.987417901	1		1.000273269	1		1.098861612	1		0.920981641	1		Quantified
P62774	Myotrophin	Mtpn	FUNCTION: Promotes dimerization of NF-kap	Myotrophin OS=Mus muscu	14.41	1	1	2	118	12.85262167	5.516113281	1.03756249	2	6.951044967	1.124108122	2	7.632222367	0.888568539	2	2.635312578	0.897923047	2	18.16247465	0.929382304	2	11.22463171	1.106840734	2	2.860821953	Quantified
P19467	Mucin-13	Muc13	FUNCTION: Epithelial and hemopoietic transı	Mucin-13 OS=Mus musculu	1.92	1	1	1	573	58.66473147	6.521972656	0.84736789	1		0.839272246	1		0.853444656	1		1.230600309	1		1.237722951	1		1.146390785	1		Quantified
Q9DBV4	Matrix remodeling-associated protein 8	Mxra8	FUNCTION: Transmembrane protein which ca	Matrix remodeling-associate	1.58	1	1	1	442	49.71930292	7.151855469	1.081853218	1		0.950616617	1		0.942225556	1		1.073569334	1		0.97222425	1		0.997591822	1		Quantified
Q5SYD0	Unconventional myosin-Id	Myo1d	FUNCTION: Unconventional myosin that func	Unconventional myosin-Id (	0.99	1	1	1	1006	116.0073576	9.407714844	1.123201334	1		0.966333476	1		0.987323899	1		1.004535803	1		0.940034137	1		0.97559322	1		Quantified
O70624	Myocilin	Myoc	FUNCTION: Secreted glycoprotein regulating	Myocilin OS=Mus musculus	1.84	1	1	1	490	55.27958222	5.604980469	0.939982145	1		0.963506124	1		1.042562665	1		0.954314998	1		1.036943936	1		1.100081948	1		Quantified
Q9DBR7	Protein phosphatase 1 regulatory subunit 12A	Ppp1r12a	FUNCTION: Key regulator of protein phospha	Protein phosphatase 1 regu	1.17	1	1	1	1029	114.9270551	5.490722656	1.038800112	1		1.039492094	1		0.99092125	1		0.947114635	1		0.990402416	1		1.00713731	1		Quantified
Q9QY36	N-alpha-acetyltransferase 10	Naa10	FUNCTION: Catalytic subunit of the N-termina	N-alpha-acetyltransferase 1	4.68	1	1	1	235	26.50291283	5.643066406	0.962018441	1		0.932330391	1		0.918091128	1		1.165469597	1		1.103860747	1		0.99733789	1		Quantified
Q6PGB6	N-alpha-acetyltransferase 50	Naa50	FUNCTION: N-alpha-acetyltransferase that a	N-alpha-acetyltransferase 5	4.73	1	1	1	169	19.40203387	8.807128906	0.988056044	1		1.01651564	1		1.026535658	1		0.967877622	1		1.029918008	1		0.999971333	1		Quantified
Q6ZQA0	Neurobeachin-like protein 2	Nbeal2	FUNCTION: Probably involved in thrombopoi	Neurobeachin-like protein 2	0.44	1	1	1	2742	301.7343883	6.442871094	0.979020269	1		0.903540704	1		1.045388705	1		0.958341928	1		1.02967612	1		1.122617783	1		Quantified
Q91W39	Nuclear receptor coactivator 5	Ncoa5	FUNCTION: Nuclear receptor coregulator that	Nuclear receptor coactivato	1.38	1	1	2	579	65.27971079	9.817871094	0.978493044	2	0.295446077	0.996987193	2	0.61490463	0.98442503	2	0.522195624	0.991073207	2	4.061415607	1.042186692	2	6.850259307	1.047331469	2	0.205424348	Quantified
Q9QYG0	Protein NDRG2	Ndrg2	FUNCTION: Contributes to the regulation of t	Protein NDRG2 OS=Mus m	6.74	2	2	3	371	40.76320053	5.401855469	0.995077585	3	3.763552051	1.064907424	3	1.091492912	0.940539296	3	10.60090902	0.978597121	3	18.67398208	0.970128389	3	2.665975709	1.035394309	3	2.135441934	Quantified
Q3UHN9	Bifunctional heparan sulfate N-deacetylase/N-sulfotra	Ndst1	FUNCTION: Essential bifunctional enzyme th	Bifunctional heparan sulfate	2.15	2	2	2	882	100.6616067	7.767089844	0.9885714	2	2.1664003	1.039985044	2	3.481516941	0.958052253	2	9.093083306	0.982670098	2	1.744033146	1.056632218	2	0.250586201	1.017667684	2	2.042716141	Quantified
P29595	NEDD8	Nedd8	FUNCTION: Ubiquitin-like protein which plays	NEDD8 OS=Mus musculus	17.28	1	1	1	81	8.966873795	7.254394531	1.021390503	1		0.884021134	1		1.015682249	1		1.037437689	1		1.063085636	1		1.032911251	1		Quantified
P70255	Nuclear factor 1 C-type	Nfic	FUNCTION: Recognizes and binds the palind	Nuclear factor 1 C-type OS	2.51	1	1	1	439	48.7378125	8.323730469	0.9509046	1		0.918874029	1		0.915003935	1		1.054132797	1		1.057048784	1		1.169168442	1		Quantified
Q8BZW8	NHL repeat-containing protein 2	Nhlrc2	FUNCTION: Required for normal embryonic d	NHL repeat-containing prote	0.97	1	1	1	725	78.3809485	5.541503906	0.988000414	1		1.037390502	1		0.983931478	1		1.006698965	1		0.970158806	1		1.021748273	1		Quantified
Q9CRB2	H/ACA ribonucleoprotein complex subunit 2	Nhp2	FUNCTION: Required for ribosome biogenesi	H/ACA ribonucleoprotein cc	6.54	1	1	1	153	17.23603612	8.411621094	0.793876787	1		1.037700221	1		1.434263444	1		0.817638129	1		0.942333973	1		0.921152133	1		Quantified
P42586	Homeobox protein Nkx-2.2	Nkx2-2	FUNCTION: Transcriptional activator involvec	Homeobox protein Nkx-2.2	4.03	1	1	1	273	30.10707129	8.089355469	0.978017384	1		0.787504609	1		0.924094019	1		1.23774929	1		1.156021257	1		1.028606348	1		Quantified
Q8K2T1	NmrA-like family domain-containing protein 1	Nmral1	FUNCTION: Redox sensor protein. Undergoe	NmrA-like family domain-cc	2.59	1	1	1	309	34.35456608	6.858886719	1.049377899	1		0.899216763	1		0.874855787	1		1.0966114	1		1.204443847	1		1.001823044	1		Quantified
Q3UFM5	Nucleolar MIF4G domain-containing protein 1	Nom1	FUNCTION: Plays a role in targeting PPP1CA	Nucleolar MIF4G domain-co	0.82	1	1	4	854	95.89980374	8.865722656	1.000243767	4	4.400367684	0.991182162	4	0.919693731	0.985140159	4	1.601048241	0.997105809	4	1.18549611	0.971438898	4	3.946404894	1.038985145	4	0.708259178	Quantified
Q7TNH6	Nephrocystin-3	Nphp3	FUNCTION: Required for normal ciliary devel	Nephrocystin-3 OS=Mus mu	0.45	1	1	1	1325	150.1948505	6.507324219	0.992034793	1		0.902949302	1		0.964295224	1		0.991617078	1		0.961392795	1		1.209072401	1		Quantified
Q91V88	Nephronectin	Npnt	FUNCTION: Functional ligand of integrin alph	Nephronectin OS=Mus mus	1.6	1	1	2	561	61.44989868	8.353027344	1.026018807	2	4.959829888	1.007797177	2	1.083997533	1.047161009	2	1.642588317	0.97429173	2	3.508403098	0.952498651	2	3.360017749	0.985357487	2	0.585789904	Quantified
Q8BHG1	Nardilysin	Nrdc	FUNCTION: Cleaves peptide substrates on th	Nardilysin OS=Mus muscul	0.78	1	1	1	1161	132.8076896	4.868652344	1.01028446	1		1.072179049	1		1.03065278	1		0.960230033	1		0.94096421	1		0.971884765	1		Quantified
Q8R480	Nuclear pore complex protein Nup85	Nup85	FUNCTION: Essential component of the nucl	Nuclear pore complex prote	1.22	1	1	2	656	74.72773664	5.566894531	1.020480746	1		1.007867378	1		0.978537531	1		1.005874148	1		0.937134549	1		1.047297869	1		Quantified
O35103	Osteomodulin	Omd	FUNCTION: May be implicated in biominerali	Osteomodulin OS=Mus mu	1.89	1	1	2	423	49.71321581	5.160644531	1.007369184	2	10.50118649	1.034288719	2	2.994337608	1.059665078	2	9.032129341	0.943095291	2	3.08819877	1.039607657	2	7.151658393	0.935511165	2	4.44611963	Quantified
P47713	Cytosolic phospholipase A2	Pla2g4a	FUNCTION: Has primarily calcium-dependen	Cytosolic phospholipase A2	0.94	1	1	1	748	85.16756547	5.427246094	1.044740836	1		0.965371572	1		0.950246218	1		1.001626864	1		1.056034311	1		1.033987998	1		Quantified
Q9CY58	Plasminogen activator inhibitor 1 RNA-binding protein	Serbp1	FUNCTION: May play a role in the regulation	Plasminogen activator inhib	2.7	1	1	1	407	44.68730202	8.543457031	0.976401666	1		1.035269353	1		1.029436669	1		1.01967853	1		1.02513509	1		0.938085826	1		Quantified
Q9D0B6	Protein PBDC1	Pbdc1		Protein PBDC1 OS=Mus m	5.05	1	1	1	198	22.20975018	4.551269531	0.985984478	1		1.051198085	1		0.973689351	1		0.990996821	1		1.03474233	1		0.997471824	1		Quantified
O55134	Protocadherin-12	Pcdh12	FUNCTION: Cellular adhesion molecule that	Protocadherin-12 OS=Mus	1.61	2	2	5	1180	128.593577	5.084472656	0.973089823	5	1.047627152	0.970575841	5	4.510737572	1.014556571	5	8.634542446	0.975107922	5	6.997391184	1.012342711	5	9.092943554	1.013828139	5	3.797360457	Quantified
Q3UA06	Pachytene checkpoint protein 2 homolog	Trip13	FUNCTION: Plays a key role in chromosome	Pachytene checkpoint prote	2.08	1	1	1	432	48.34638432	5.858886719	1.054550701	1		0.844794631	1		1.061586598	1		1.017207021	1		1.148016909	1		0.958979484	1		Quantified
Q9QYX7	Protein piccolo	Pclo	FUNCTION: Scaffold protein of the presynapt	Protein piccolo OS=Mus mu	0.36	2	2	2	5068	550.4961714	6.507324219	1.028379643	2	1.389787727	1.003933876	2	3.886595678	0.992152452	2	2.726681556	1.014823179	2	1.077317084	1.00541903	2	0.741473636	0.976788222	2	3.470865358	Quantified
P17918	Proliferating cell nuclear antigen	Pcna	FUNCTION: Auxiliary protein of DNA polymer	Proliferating cell nuclear an	8.81	2	2	2	261	28.76635231	4.767089844	0.992925124	2	4.457018765	0.983459236	2	1.748448081	1.020183765	2	2.38369836	0.999113489	2	1.778218476	1.01458806	2	3.608366084	1.014997904	2	0.429897945	Quantified
Q9D5U0	Lysophosphatidylcholine acyltransferase 2B	Lpcat2b	FUNCTION: Probable acetyltransferase. {EC	Lysophosphatidylcholine ac	3.29	1	1	1	516	58.34379742	8.909667969	0.907012017	1		1.04283587	1		0.781208944	1		1.075953538	1		1.135566059	1		1.160874964	1		Quantified

Q922E4	Ethanolamine-phosphate cytidylyltransferase	Pcyt2	FUNCTION: Plays an important role in the bic	Ethanolamine-phosphate cy	3.71	2	2	2	404	45.20657124	6.580566406	1.040935806	2	3.037701761	0.981086123	2	4.123846455	0.972698368	2	7.043708172	1.021263881	2	2.764890381	0.961607655	2	12.1720269	1.025556513	2	9.696086684	Quantified
Q8C7K6	Prenylcysteine oxidase-like	Pcyox1l	FUNCTION: Probable oxidoreductase. {ECO:	Prenylcysteine oxidase-like	3.23	2	2	2	495	54.84019172	7.649902344	0.979983995	2	1.911062149	0.98457651	2	0.427110533	0.980846051	2	3.775549554	1.032768966	2	2.054965721	1.000910319	2	2.751166582	1.046162123	2	1.227040511	Quantified
Q61823	Programmed cell death protein 4	Pdcd4	FUNCTION: Inhibits translation initiation and	Programmed cell death pro	1.92	1	1	1	469	51.66999085	5.160644531	1.030196827	1		1.063262584	1		1.032665556	1		0.946564059	1		0.975928667	1		0.951599101	1		Quantified
Q9Z0X4	cGMP-inhibited 3',5'-cyclic phosphodiesterase A	Pde3a	FUNCTION: Cyclic nucleotide phosphodieste	cGMP-inhibited 3',5'-cyclic	0.96	1	1	1	1141	124.4342866	6.303222656	1.000176481	1		0.811156891	1		1.170563821	1		0.885894823	1		1.167237562	1		1.051379135	1		Quantified
Q8CG03	cGMP-specific 3',5'-cyclic phosphodiesterase	Pde5a	FUNCTION: Plays a role in signal transductio	cGMP-specific 3',5'-cyclic p	2.2	2	2	2	865	98.34429874	6.049316406	1.014092564	2	2.501033903	1.021473828	2	1.067068611	1.043699701	2	1.64735506	0.984955037	2	1.083708252	0.959643848	2	5.713173571	0.97184457	2	1.230743458	Quantified
P23440	Rod cGMP-specific 3',5'-cyclic phosphodiesterase su	Pde6b	FUNCTION: This protein participates in proce	Rod cGMP-specific 3',5'-cy	1.52	1	1	1	856	98.47124974	5.312988281	0.932034813	1		1.102120014	1		1.102806175	1		0.897867755	1		0.996666465	1		0.968541408	1		Quantified
Q8CI19	Platelet-derived growth factor C	Pdgfc	FUNCTION: Growth factor that plays an esse	Platelet-derived growth fact	2.32	1	1	1	345	38.71660145	7.166503906	1.018218381	1		1.050477528	1		0.942446297	1		0.958853039	1		0.967365917	1		1.073528199	1		Quantified
O70400	PDZ and LIM domain protein 1	Pdlim1	FUNCTION: Cytoskeletal protein that may ac	PDZ and LIM domain protei	6.42	2	2	2	327	35.75188904	6.844238281	0.960322179	2	3.444183141	1.005978058	2	12.95570367	1.076245049	2	4.010009631	1.007014328	2	18.85984684	1.062348093	2	0.507808124	0.911132444	2	3.445934131	Quantified
Q4VA53	Sister chromatid cohesion protein PDS5 homolog B	Pds5b	FUNCTION: Regulator of sister chromatid co	Sister chromatid cohesion p	1.24	2	2	2	1446	164.3151899	8.499511719	1.047546484	2	2.671370757	1.040927319	2	0.464043752	0.894365534	2	4.195621431	1.010332021	2	8.867407084	0.972146388	2	10.10355552	1.04872234	2	3.255836236	Quantified
Q99K01	Pyridoxal-dependent decarboxylase domain-containi	Pdxdc1		Pyridoxal-dependent decarl	1.91	2	2	3	787	87.28074896	5.478027344	0.983944532	3	2.199030567	0.951337035	3	1.675308771	1.052883369	3	2.123604808	0.991038091	3	1.575473376	1.066832582	3	4.618661468	0.902041787	3	5.949443218	Quantified
Q8K183	Pyridoxal kinase	Pdxk	FUNCTION: Catalyzes the phosphorylation o	Pyridoxal kinase OS=Mus r	3.21	1	1	1	312	34.99265624	6.290527344	0.992989718	1		1.055318069	1		0.995754063	1		1.000707489	1		1.011060278	1		0.964809563	1		Quantified
Q9QY39	PDZ domain-containing protein 4	Pdzd4		PDZ domain-containing pro	1.68	1	1	1	772	86.76453694	5.985839844	1.032253756	1		0.965475985	1		0.982390938	1		1.011442806	1		1.015154034	1		1.02486072	1		Quantified
Q11136	Xaa-Pro dipeptidase	Pepd	FUNCTION: Splits dipeptides with a prolyl or	Xaa-Pro dipeptidase OS=M	3.85	1	1	1	493	54.99296723	5.782714844	1.179478151	1		0.652575162	1		1.146888961	1		0.989162544	1		1.172057472	1		0.958007397	1		Quantified
P61759	Prefoldin subunit 3	Vbp1	FUNCTION: Binds specifically to cytosolic ch	Prefoldin subunit 3 OS=Mu	4.08	1	1	1	196	22.42139596	6.277832031	0.986149491	1		0.894908646	1		1.007149464	1		1.215756425	1		1.199065963	1		0.803510336	1		Quantified
P47857	ATP-dependent 6-phosphofructokinase, muscle type	Pfkm	FUNCTION: Catalyzes the phosphorylation o	ATP-dependent 6-phosphof	1.03	1	1	1	780	85.21456508	8.001464844	1.17563939	1		0.973760407	1		0.905765837	1		1.033883982	1		0.982117654	1		0.950237172	1		Quantified
P28654	Decorin	Dcn	FUNCTION: May affect the rate of fibrils form	Decorin OS=Mus musculus	4.52	2	2	2	354	39.78390249	8.675292969	1.100009274	2	7.052184817	1.086847111	2	4.794590025	0.921111422	2	6.340679859	0.96261793	2	8.155712165	0.907590923	2	2.318492524	0.999937831	2	0.294292652	Quantified
P16331	Phenylalanine-4-hydroxylase	Pah	FUNCTION: Catalyzes the hydroxylation of L-	Phenylalanine-4-hydroxylas	3.75	2	2	2	453	51.86739369	6.303222656	0.826249811	2	25.23739539	0.895696984	2	20.40598045	0.826865756	2	23.69208251	1.085634777	2	8.016849256	1.250901431	2	27.07506259	1.207244638	2	29.13805232	Quantified
P61458	Pterin-4-alpha-carbinolamine dehydratase	Pcbd1	FUNCTION: Involved in tetrahydrobiopterin bi	Pterin-4-alpha-carbinolamir	6.73	1	1	1	104	11.9780007	6.800292969	0.986956417	1		0.966907638	1		0.902721485	1		1.106387894	1		1.102049426	1		1.012044272	1		Quantified
Q9DCE5	p21-activated protein kinase-interacting protein 1	Pak1ip1	FUNCTION: Negatively regulates the PAK1 k	p21-activated protein kinas	2.09	1	1	1	382	42.0893706	8.470214844	1.033050636	1		0.96916706	1		1.044324531	1		1.004662621	1		0.993205965	1		0.969563752	1		Quantified
O08852	Polycystin-1	Pkd1	FUNCTION: Component of a heteromeric calc	Polycystin-1 OS=Mus musc	0.21	1	1	1	4293	466.2807299	5.871582031	0.915225634	1		0.933919041	1		0.94740256	1		1.01723218	1		1.083911174	1		1.174441223	1		Quantified
Q62077	1-phosphatidylinositol 4,5-bisphosphate phosphodies	Plcg1	FUNCTION: Mediates the production of the s	1-phosphatidylinositol 4,5-b	0.69	1	1	1	1302	149.5732534	5.795410156	0.838456259	1		0.87917151	1		0.792971462	1		0.86318259	1		0.924094875	1		1.744257179	1		Quantified
Q9JHK5	Pleckstrin	Plek	FUNCTION: Major protein kinase C substrate	Pleckstrin OS=Mus muscul	6	2	2	2	350	39.87551082	8.338378906	0.98230389	2	2.492421755	1.040891843	2	1.138534808	0.945989089	2	7.529169017	1.002621285	2	5.08705952	0.972897835	2	10.39555072	1.06330847	2	10.46144758	Quantified
Q9R0E2	Procollagen-lysine,2-oxoglutarate 5-dioxygenase 1	Plod1	FUNCTION: Part of a complex composed of F	Procollagen-lysine,2-oxoglu	1.51	1	1	2	728	83.54229345	6.536621094	0.950932442	2	6.844669747	1.105426412	2	8.276657095	0.999752124	2	5.890864652	0.868940463	2	14.84073242	1.008171428	2	10.82339154	1.07500798	2	3.194403777	Quantified
P70208	Plexin-A3	Plxna3	FUNCTION: Coreceptor for SEMA3A and SEI	Plexin-A3 OS=Mus muscul	0.37	1	1	1	1872	207.825228	7.093261719	0.986128596	1		1.020735086	1		0.962480348	1		1.038169267	1		1.022275349	1		1.003048459	1		Quantified
Q9R0M4	Podocalyxin	Podxl	FUNCTION: Involved in the regulation of both	Podocalyxin OS=Mus musc	2.98	1	1	2	503	53.35647088	4.970214844	1.061742663	2	2.393935774	1.048072175	2	1.782294574	0.958531263	2	5.978000514	0.97387599	2	3.539102792	0.938297626	2	5.198373332	1.012708933	2	2.362270718	Quantified
P97814	Proline-serine-threonine phosphatase-interacting pro	Pstpip1	FUNCTION: Involved in regulation of the actir	Proline-serine-threonine ph	1.69	1	1	1	415	47.56062678	5.528808594	1.081306727	1		0.929142133	1		0.973324486	1		1.014336031	1		1.003207652	1		1.028349791	1		Quantified
Q60676	Serine/threonine-protein phosphatase 5	Ppp5c	FUNCTION: Serine/threonine-protein phospha	Serine/threonine-protein ph	2.2	1	1	1	499	56.84020209	6.201660156	0.919381794	1		1.031984773	1		1.00532905	1		0.894350638	1		1.236342491	1		1.029698723	1		Quantified
O08709	Peroxiredoxin-6	Prdx6	FUNCTION: Thiol-specific peroxidase that ca	Peroxiredoxin-6 OS=Mus m	9.38	2	2	2	224	24.85497617	6.011230469	1.00338403	2	2.821204945	1.036377293	2	10.89801041	0.906740466	2	20.36213848	1.017392051	2	4.433324934	1.034689351	2	8.149784963	1.030364395	2	4.080076253	Quantified
Q08761	Vitamin K-dependent protein S	Pros1	FUNCTION: Anticoagulant plasma protein; it i	Vitamin K-dependent protei	1.19	1	1	2	675	74.88578639	5.820800781	1.026890685	2	1.591597274	1.003212029	2	0.351277681	0.981839	2	0.273457037	0.978826749	2	4.034448581	1.034541776	2	2.826370671	1.010148744	2	3.961255556	Quantified
Q9DAW6	U4/U6 small nuclear ribonucleoprotein Prp4	Prpf4	FUNCTION: Plays role in pre-mRNA splicing	U4/U6 small nuclear ribonu	1.73	1	1	1	521	58.33334655	7.283691406	0.953378417	1		0.924023662	1		0.95775723	1		1.165757981	1		1.148402726	1		0.943302235	1		Quantified
Q3UPH1	Protein PRRC1	Prrc1		Protein PRRC1 OS=Mus m	2.93	1	1	1	443	46.26845501	5.947753906	0.833226894	1		0.827170556	1		0.786721614	1		1.211367409	1		1.376658689	1		1.186596669	1		Quantified
Q99JF8	PC4 and SFRS1-interacting protein	Psip1	FUNCTION: Transcriptional coactivator involv	PC4 and SFRS1-interacting	4.36	2	2	2	528	59.6604585	9.129394531	0.979335334	2	5.328675062	0.984350475	2	0.870220284	1.01428135	2	1.666499191	1.013829274	2	3.802995548	1.049119313	2	0.469475042	0.99860523	2	4.088076303	Quantified
Q99JI4	26S proteasome non-ATPase regulatory subunit 6	Psmd6	FUNCTION: Component of the 26S proteasor	26S proteasome non-ATPa	7.97	3	3	4	389	45.50722536	5.516113281	0.942337213	4	2.68334421	1.036864261	4	10.65830376	0.976370103	4	1.842176575	0.996435467	4	8.868898381	1.08073481	4	3.373497147	1.000902809	4	3.083048075	Quantified
Q9CX56	26S proteasome non-ATPase regulatory subunit 8	Psmd8	FUNCTION: Component of the 26S proteasor	26S proteasome non-ATPa	1.98	1	1	1	353	39.9049706	9.583496094	0.979580997	1		0.957892357	1		1.030091309	1		1.008237548	1		1.064800983	1		1.007334121	1		Quantified
P97371	Proteasome activator complex subunit 1	Psme1	FUNCTION: Implicated in immunoproteasom	Proteasome activator comp	15.66	3	3	3	249	28.65496069	5.973144531	0.964700422	3	1.80460605	0.978507043	3	7.742673637	0.998122477	3	11.2399888	1.027777858	3	4.551665222	0.942138356	3	0.676517896	1.001309166	3	9.521604437	Quantified
P35831	Tyrosine-protein phosphatase non-receptor type 12	Ptpn12	FUNCTION: Dephosphorylates a range of pro	Tyrosine-protein phosphata	1.03	1	1	1	775	86.47253392	5.985839844	0.930148497	1		0.787691589	1		0.963449706	1		0.857292364	1		1.482759944	1		1.225702129	1		Quantified
P46664	Adenylosuccinate synthetase isozyme 2	Adss2	FUNCTION: Plays an important role in the de	Adenylosuccinate syntheta	4.61	1	1	1	456	49.98958452	6.379394531	0.960663269	1		0.9119463	1		0.953718381	1		0.96855868	1		0.959777683	1		1.268437311	1		Quantified
Q3UQ28	Peroxidasin homolog	Pxdn	FUNCTION: Displays low peroxidase activity	Peroxidasin homolog OS=M	0.68	1	1	1	1475	164.9977378	7.137207031	0.994074471	1		0.985605245	1		1.009419566	1		0.980301965	1		0.9620761	1		1.075414705	1		Quantified
P70303	CTP synthase 2	Ctps2	FUNCTION: Catalyzes the ATP-dependent ar	CTP synthase 2 OS=Mus n	2.05	1	1	1	586	65.47265933	6.493652344	1.03485156	1		1.01137668	1		0.987067204	1		1.036964339	1		0.956930881	1		0.975058735	1		Quantified
O35551	Rab GTPase-binding effector protein 1	Rabep1	FUNCTION: Rab effector protein acting as lin	Rab GTPase-binding effect	1.04	1	1	2	862	99.46235666	5.008300781	0.97557277	2	5.315286494	1.00060203	2	7.080711776	0.980399321	2	6.657968615	0.96695648	2	8.088080423	0.938372389	2	1.185130734	1.134430546	2	2.226497033	Quantified
P46061	Ran GTPase-activating protein 1	Rangap1	FUNCTION: GTPase activator for RAN. Conv	Ran GTPase-activating pro	1.7	1	1	1	589	63.49137219	4.678222656	1.043932201	1		0.986750606	1		0.970116957	1		1.027671631	1		1.035069764	1		0.974597593	1		Quantified
P63321	Ras-related protein Ral-A	Rala	FUNCTION: Multifunctional GTPase involved	Ras-related protein Ral-A C	9.22	2	2	2	206	23.5379576	7.107910156	0.987944535	2	9.640356176	0.97789984	2	1.69755495	1.061721429	2	6.901756193	0.976687936	2	3.728442031	1.001085971	2	4.456968198	1.006872453	2	0.786591445	Quantified
Q99MI1	ELKS/Rab6-interacting/CAST family member 1	Erc1	FUNCTION: Regulatory subunit of the IKK co	ELKS/Rab6-interacting/CAS	0.8	1	1	1	1120	128.2516404	5.871582031	0.96888685	1		0.982776874	1		0.889305445	1		1.089378894	1		1.068568725	1		1.066145508	1		Quantified
Q8BX09	Retinoblastoma-binding protein 5	Rbbp5	FUNCTION: In embryonic stem (ES) cells, pla	Retinoblastoma-binding pro	1.12	1	1	1	538	59.06062702	5.097167969	1.05023687	1		1.030611321	1		0.963562325	1		1.003540381	1		1.021491635	1		0.959838691	1		Quantified
Q8BMG7	Rab3 GTPase-activating protein non-catalytic subuni	Rab3gap2	FUNCTION: Regulatory subunit of a GTPase	Rab3 GTPase-activating pr	0.66	1	1	1	1366	152.4380523	6.227050781	1.049124794	1		0.999492472	1		0.916183611	1		1.079261428	1		1.020799389	1		0.972391634	1		Quantified
B2RY56	RNA-binding protein 25	Rbm25	FUNCTION: RNA-binding protein that acts as	RNA-binding protein 25 OS	1.55	1	1	1	838	99.49119102	6.315917969	0.893553115	1		1.064717423	1		1.06743975	1		0.938789664	1		0.969274926	1		1.064953264	1		Quantified
Q05186	Reticulocalbin-1	Rcn1	FUNCTION: May regulate calcium-dependent	Reticulocalbin-1 OS=Mus n	9.85	3	3	4	325	38.08963388	4.843261719	1.025432698	4	5.372035818	0.969684026	4	3.555084786	0.875631419	4	5.621421706	1.037886096	4	8.951531862	1.063398558	4	7.672043738	1.00845084	4	0.522187207	Quantified
Q00915	Retinol-binding protein 1	Rbp1	FUNCTION: Cytoplasmic retinol-binding prote	Retinol-binding protein 1 OS	9.63	1	1	1	135	15.83584178	5.249511719	1.025688164	1		1.017112378	1		0.904029999	1		0.949238116	1		0.922767171	1		1.182953623	1		Quantified
Q00724	Retinol-binding protein 4	Rbp4	FUNCTION: Retinol-binding protein that medi	Retinol-binding protein 4 OS	3.48	1	1	2	201	23.19129036	5.985839844	1.0538426	2	5.644072083	1.062837226	2	4.231729725	0.987781227	2	0.977703281	0.930671238	2	2.434250691	1.012824286	2	1.702661173	0.971026924	2	1.457301652	Quantified
Q5FWK3	Rho GTPase-activating protein 1	Arhgap1	FUNCTION: GTPase activator for the Rho, Ra	Rho GTPase-activating pro	2.28	1	1	1	439	50.37908872	6.442871094	0.970866823	1		0.96980707	1		1.003458503	1		1.055411959	1		1.057052862	1		0.99006454	1		Quantified
Q8BWR8	Rhophilin-2	Rhpn2	FUNCTION: Binds specifically to GTP-Rho. M	Rhophilin-2 OS=Mus muscu	1.02	1	1	1	686	76.90825685	6.800292969	0.969487236	1		0.968051588	1		1.000080709	1		0.997222623	1		1.09918604	1		1.031390275	1		Quantified
P47963	60S ribosomal protein L13	Rpl13		60S ribosomal protein L13	9	2	2	2	211	24.29050586	11.54638672	0.988228887	2	0.081346503	1.01115873	2	9.381856573	1.020032636	2	7.924617345	1.002742281	2	3.628958783	1.003223592	2	11.87059719	0.98562341	2	9.02421925	Quantified
O09167	60S ribosomal protein L21	Rpl21	FUNCTION: Component of the large ribosom	60S ribosomal protein L21	5	1	1	1	160	18.55006206	10.49169922	1.011849542	1		0.937772528	1		1.002721366	1		1.037819165	1		1.057474317	1		1.001232529	1		Quantified
P47915	60S ribosomal protein L29	Rpl29	FUNCTION: Component of the large ribosom	60S ribosomal protein L29	6.88	1	1	1	160	17.5759257	11.83935547	0.807716439	1		0.903185562	1		0.906149704	1		1.179753304	1		1.091339372	1		1.197103773	1		Quantified
P27659	60S ribosomal protein L3	Rpl3	FUNCTION: The L3 protein is a component o	60S ribosomal protein L3 O	4.71	2	2	2	403	46.08079271	10.21337891	1.003498274	2	0.266863506	1.035179426	2	0.441828138	1.073932033	2	2.688382374	0.929929912	2	4.396360431	0.910033905	2	5.929750243	1.018622489	2	4.277212704	Quantified
Q9D8E6	60S ribosomal protein L4	Rpl4		60S ribosomal protein L4 O	2.86	1	1	1	419	47.12401139	11.00439453	1.010093609	1		0.983506689	1		0.987874756	1		1.026376227	1		0.997921319	1		1.016936	1		Quantified
P47962	60S ribosomal protein L5	Rpl5	FUNCTION: Component of the ribosome, a la	60S ribosomal protein L5 O	13.47	5	5	5	297	34.37870859	9.773925781	1.015632154	5	9.182020465	1.037331886	5	2.631416894	0.986161804	5	7.608497939	0.988701694	5	3.84353015	1.039616284	5	8.752129983	0.966178213	5	3.837821348	Quantified
P12970	60S ribosomal protein L7a	Rpl7a		60S ribosomal protein L7a	4.89	1	1	1	266	29.95798332	10.56494141	0.954750108	1		0.946527938	1		0.896069879	1		1.111412212	1		1.06489948	1		1.092942566	1		Quantified
P14148	60S ribosomal protein L7	Rpl7	FUNCTION: Component of the large ribosom	60S ribosomal protein L7 O	7.04	1	1	1	270	31.39975102	10.88720703	1.0842736	1		1.20823214	1		0.872729987	1		0.822032275	1		0.865318816	1		1.110782731	1		Quantified
Q9CWY8	Ribonuclease H2 subunit A	Rnaseh2a	FUNCTION: Catalytic subunit of RNase HII, a	Ribonuclease H2 subunit A	4.32	1	1	1	301	33.49169722	4.982910156																			Identified
Q7TPD3	Roundabout homolog 2	Robo2	FUNCTION: Receptor for SLIT2, and probabl	Roundabout homolog 2 OS	0.88	1	1	1	1470	161.0899989	6.328613281	0.962654843	1		1.002814143	1		1.072745149	1		1.005475631	1		1.089770466	1		0.914629193	1		Quantified
Q80UW8	DNA-directed RNA polymerases I, II, and III subunit F	Polr2e	FUNCTION: DNA-dependent RNA polymeras	DNA-directed RNA polymer	12.38	2	2	2	210	24.55471301	5.947753906	0.985781447	2	5.241326168	0.961658393	2	13.44273894	0.990992207	2	5.533117522	1.00068799	2	16.36341303	1.140198894	2	3.033343911	0.991244887	2	3.674247721	Quantified
O08740	DNA-directed RNA polymerase II subunit RPB11	Polr2j	FUNCTION: DNA-dependent RNA polymeras	DNA-directed RNA polymer	6.84	1	1	1	117	13.24299367	5.858886719	0.965986999	1		0.913876738	1		0.97217435	1		1.047955666	1		1.158499515	1		1.039183557	1		Quantified
P08775	DNA-directed RNA polymerase II subunit RPB1	Polr2a	FUNCTION: DNA-dependent RNA polymeras	DNA-directed RNA polymer	1.62	3	3	3	1970	217.0390662	7.371582031	0.929784157	3	1.435385834	0.974368382	3	7.24643923	1.05680257	3	11.31394433	0.978404383	3	3.159888311	1.078664101	3	8.12989991	0.937742305	3	8.640223374	Quantified
Q9CSH3	Exosome complex exonuclease RRP44	Dis3	FUNCTION: Putative catalytic component of t	Exosome complex exonucl	1.46	1	1	1	958	108.7691991	7.532714844	0.921502227	1		0.964374033	1		1.01432168	1		1.044538125	1		1.086389923	1		1.028688072	1		Quantified
Q99P72	Reticulon-4	Rtn4	FUNCTION: Required to induce the formation	Reticulon-4 OS=Mus muscu	1.38	1	1	1	1162	126.535055	4.538574219	0.888193062	1		1.070901791	1		0.865948618	1		0.996852456	1		1.157079194	1		1.120897966	1		Quantified
P57784	U2 small nuclear ribonucleoprotein A'	Snrpa1	FUNCTION: Involved in pre-mRNA splicing a	U2 small nuclear ribonucle	5.1	1	1	1	255	28.3401471	8.616699219	0.934137542	1		0.978853117	1		1.004596201	1		1.043575728	1		1.127574737	1		0.986803171	1		Quantified
Q8R4C2	RUN and FYVE domain-containing protein 2	Rufy2		RUN and FYVE domain-cor	1.16	1	1	1	606	70.04896038	5.833496094	0.97162109	1		0.996526797	1		0.984241554	1		1.047178218	1		0.999420246	1		1.024695234	1		Quantified
P56565	Protein S100-A1	S100a1	FUNCTION: Probably acts as a Ca(2+) signal	Protein S100-A1 OS=Mus r	7.45	1	1	2	94	10.49811665	4.500488281	0.986198028	2	6.662179216	0.855550749	2	2.715948103	0.976752009	2	2.347061633	1.049482722	2	1.415053501	1.236544363	2	0.009738412	1.025907814	2	8.131416536	Quantified
P27005	Protein S100-A8	S100a8	FUNCTION: S100A8 is a calcium- and zinc-b	Protein S100-A8 OS=Mus r	7.87	1	1	1	89	10.28806641	5.681152344	1.065084353	1		0.951199705	1		0.931736628	1		1.012473686	1		1.594745759	1		0.714551508	1		Quantified
Q9Z0L1	Sphingosine 1-phosphate receptor 4	S1pr4	FUNCTION: Receptor for the lysosphingolipic	Sphingosine 1-phosphate re	2.07	1	1	1	386	42.23527447	9.773925781	1.050971714	1		1.0090444	1		0.943870274	1		1.07326113	1		0.965383256	1		0.967835789	1		Quantified
Q9D0K2	Succinyl-CoA:3-ketoacid coenzyme A transferase 1,	Oxct1	FUNCTION: Key enzyme for ketone body cat	Succinyl-CoA:3-ketoacid co	2.12	1	1	1	520	55.95300003	8.528808594	1.000561361	1		1.013887633	1		0.972845146	1		0.964310293	1		0.987059953	1		1.080354104	1		Quantified
Q3UH53	Protein sidekick-1	Sdk1	FUNCTION: Adhesion molecule that promote	Protein sidekick-1 OS=Mus	0.32	1	1	1	2193	240.1562611	6.770996094	0.98547304	1		0.952055063	1		0.950785662	1		1.046200069	1		1.131254708	1		1.019195355	1		Quantified
Q9D1M0	Protein SEC13 homolog	Sec13	FUNCTION: Functions as a component of the	Protein SEC13 homolog OS	2.8	1	1	1	322	35.54307432	5.376464844	0.871818801	1		1.028281669	1		1.217174944	1		0.896431054	1		0.960888705	1		1.005724245	1		Quantified
O09126	Semaphorin-4D	Sema4d	FUNCTION: Cell surface receptor for PLXNB	Semaphorin-4D OS=Mus m	0.7	1	1	1	861	95.5793477	7.811035156	1.030007968	1		1.056410486	1		1.040993293	1		0.913266611	1		1.039018578	1		0.945964874	1		Quantified
Q76KF0	Semaphorin-6D	Sema6d	FUNCTION: Shows growth cone collapsing a	Semaphorin-6D OS=Mus m	0.75	1	1	1	1073	119.7391355	8.514160156	0.985310991	1		0.924742299	1		0.947033593	1		1.024886924	1		0.989210123	1		1.160965807	1		Quantified
Q62419	Endophilin-A2	Sh3gl1	FUNCTION: Implicated in endocytosis. May r	Endophilin-A2 OS=Mus mu	4.08	2	2	2	368	41.4922234	5.719238281	0.996711655	2	2.69698971	0.985763483	2	7.811525013	1.000684367	2	8.300066589	1.006195085	2	14.64916143	1.027944077	2	6.966629073	1.00683746	2	4.410316155	Quantified
Q91VW3	SH3 domain-binding glutamic acid-rich-like protein 3	Sh3bgrl3	FUNCTION: Could act as a modulator of glut	SH3 domain-binding glutam	10.75	1	1	1	93	10.47027558	5.135253906	0.955072269	1		0.961622897	1		1.130329326	1		1.018810602	1		1.092700087	1		0.887107365	1		Quantified
P46062	Signal-induced proliferation-associated protein 1	Sipa1	FUNCTION: GTPase activator for the nuclear	Signal-induced proliferation	2.03	2	2	2	1037	111.996895	6.354003906	1.002825553	2	4.363260262	0.970125549	2	7.377646527	1.060511778	2	0.51939754	0.949467133	2	4.540720877	1.07738622	2	8.77934048	0.983971231	2	1.452730846	Quantified
Q8CG47	Structural maintenance of chromosomes protein 4	Smc4	FUNCTION: Central component of the conde	Structural maintenance of c	1.09	1	1	1	1286	146.8033256	7.298339844	0.992798239	1		0.900700919	1		1.084947083	1		1.027550175	1		0.939253887	1		1.050014968	1		Quantified
O09044	Synaptosomal-associated protein 23	Snap23	FUNCTION: Essential component of the high	Synaptosomal-associated p	6.19	1	1	1	210	23.2463877	4.982910156	0.920747492	1		0.976921014	1		0.97660175	1		1.039313817	1		1.316463118	1		0.925581976	1		Quantified
Q62189	U1 small nuclear ribonucleoprotein A	Snrpa	FUNCTION: Component of the spliceosomal	U1 small nuclear ribonucle	5.23	1	2	2	287	31.81449165	9.803222656	1.019295876	1		0.992284858	1		1.137609839	1		0.934464526	1		1.007311054	1		0.915399246	1		Quantified
Q99L88	Beta-1-syntrophin	Sntb1	FUNCTION: Adapter protein that binds to and	Beta-1-syntrophin OS=Mus	3.35	1	1	1	537	58.04499993	8.309082031																			Identified
O70493	Sorting nexin-12	Snx12	FUNCTION: May be involved in several stage	Sorting nexin-12 OS=Mus r	6.67	1	1	1	165	19.10375064	7.342285156	0.974624754	1		0.990149454	1		1.002085166	1		0.984698674	1		1.067808683	1		1.03099066	1		Quantified
Q3UHD6	Sorting nexin-27	Snx27	FUNCTION: Involved in the retrograde transp	Sorting nexin-27 OS=Mus r	2.78	1	1	1	539	60.95030274	6.404785156	1.064953243	1		0.888205901	1		0.914517642	1		1.079052985	1		1.205284168	1		0.970005946	1		Quantified
Q9CY18	Sorting nexin-7	Snx7	FUNCTION: May be involved in several stage	Sorting nexin-7 OS=Mus m	2.84	1	1	1	387	44.97143342	5.071777344	1.089826151	1		0.96449116	1		0.887748049	1		0.994289799	1		1.015159446	1		1.091122838	1		Quantified
Q9EPT5	Solute carrier organic anion transporter family memb	Slco2a1	FUNCTION: May mediate the release of newl	Solute carrier organic anion	1.24	1	1	1	643	70.10115021	8.748535156	0.955021104	1		0.937750718	1		1.04045142	1		1.066645803	1		1.067013811	1		0.982436705	1		Quantified
Q6P069	Sorcin	Sri	FUNCTION: Calcium-binding protein that mod	Sorcin OS=Mus musculus	9.09	2	2	2	198	21.61329039	5.592285156	0.848378009	2	14.35919205	0.934219301	2	17.63353447	1.067293909	2	26.96369537	1.053270019	2	25.98016623	1.25703406	2	64.21860963	0.856898777	2	11.78792018	Quantified
Q91WP6	Serine protease inhibitor A3N	Serpina3n		Serine protease inhibitor A3	4.55	1	1	1	418	46.68828064	5.820800781	1.032460642	1		0.98683025	1		0.889520861	1		1.085273603	1		1.027338431	1		1.024052572	1		Quantified
Q8BH69	Selenide, water dikinase 1	Sephs1	FUNCTION: Synthesizes selenophosphate fr	Selenide, water dikinase 1	3.06	1	1	1	392	42.87872091	5.973144531	1.032427569	1		0.953346011	1		1.00164101	1		1.055965119	1		1.078830328	1		0.932411861	1		Quantified
O55201	Transcription elongation factor SPT5	Supt5h	FUNCTION: Component of the DRB sensitivit	Transcription elongation fac	0.83	1	1	1	1082	120.5893478	5.046386719	1.091639254	1		1.00665298	1		0.98135588	1		0.887656234	1		1.025163612	1		1.038985017	1		Quantified
P05480	Neuronal proto-oncogene tyrosine-protein kinase Src	Src	FUNCTION: Non-receptor protein tyrosine kin	Neuronal proto-oncogene ty	4.25	1	3	3	541	60.60648223	7.840332031	1.098385906	1		0.999983017	1		0.962881025	1		0.897690774	1		0.914415213	1		1.116673251	1		Quantified
O35326	Serine/arginine-rich splicing factor 5	Srsf5	FUNCTION: May be required for progression	Serine/arginine-rich splicing	2.6	1	1	1	269	30.8730733	11.56103516	1.045160455	1		1.017567291	1		0.978688541	1		1.021009463	1		0.965073682	1		0.978400021	1		Quantified
Q8BL97	Serine/arginine-rich splicing factor 7	Srsf7	FUNCTION: Required for pre-mRNA splicing.	Serine/arginine-rich splicing	7.87	1	1	1	267	30.79883662	11.89794922	0.928026619	1		1.043640325	1		1.153186237	1		0.933674169	1		0.985468729	1		0.950129926	1		Quantified
Q9D939	Sulfotransferase 1C2	Sult1c2	FUNCTION: Sulfotransferase that utilizes 3'-p	Sulfotransferase 1C2 OS=M	3.38	1	1	1	296	34.93045031	7.825683594	0.942851295	1		0.930726467	1		0.982219643	1		1.024362873	1		1.149179493	1		1.062979591	1		Quantified
Q9JM90	Signal-transducing adaptor protein 1	Stap1	FUNCTION: May function as an adapter mole	Signal-transducing adaptor	2.36	1	1	1	297	34.60600854	8.440917969	1.091427449	1		1.026590433	1		0.952929806	1		0.972265551	1		0.992882346	1		0.98253647	1		Quantified
P42227	Signal transducer and activator of transcription 3	Stat3	FUNCTION: Signal transducer and transcripti	Signal transducer and activ	2.47	2	2	2	770	87.99736486	6.303222656	1.018124257	2	1.169101231	1.018787026	2	1.646369474	1.005117962	2	5.635185374	1.006844817	2	2.203952713	0.96560246	2	6.700453505	0.987678239	2	0.913648726	Quantified
Q5DQR4	Syntaxin-binding protein 5-like	Stxbp5l	FUNCTION: Plays a role in vesicle trafficking	Syntaxin-binding protein 5-l	1.1	1	1	2	1185	131.7597426	6.858886719	1.017752403	2	3.048944506	1.049835749	2	0.552168238	1.000365602	2	13.23933674	0.887049636	2	14.70377534	1.078232995	2	10.37048593	1.003455257	2	7.528189262	Quantified
O55098	Serine/threonine-protein kinase 10	Stk10	FUNCTION: Serine/threonine-protein kinase i	Serine/threonine-protein kir	2.07	1	2	2	966	111.8360339	7.151855469	1.086525922	1		0.948172834	1		1.012106243	1		0.990078418	1		1.056967697	1		0.950187005	1		Quantified
Q91VJ4	Serine/threonine-protein kinase 38	Stk38	FUNCTION: Negative regulator of MAP3K1/2	Serine/threonine-protein kir	4.09	2	2	2	465	54.13941698	7.342285156	0.981555948	2	1.725045719	1.044243831	2	1.98022352	1.025970086	2	0.136695201	0.98405237	2	2.898265759	0.974154663	2	4.417234449	0.993145877	2	0.352565932	Quantified
Q9JI11	Serine/threonine-protein kinase 4	Stk4	FUNCTION: Stress-activated, pro-apoptotic k	Serine/threonine-protein kir	5.75	2	2	2	487	55.50578405	5.186035156	0.976355126	2	0.87805873	1.014667595	2	0.231460715	1.100680109	2	6.100607251	0.93211812	2	3.885361898	0.945340387	2	2.167948216	1.015466817	2	3.294547812	Quantified
Q8R3F9	Speckle targeted PIP5K1A-regulated poly(A) polyme	Tut1	FUNCTION: Poly(A) polymerase that creates	Speckle targeted PIP5K1A-	1.38	1	1	1	869	94.54373525	5.808105469																			Identified
Q9Z1Z2	Serine-threonine kinase receptor-associated protein	Strap	FUNCTION: The SMN complex plays a catal	Serine-threonine kinase rec	18.57	5	5	8	350	38.41798857	5.122558594	1.044240132	8	4.057001198	0.96802011	8	6.269842123	1.008293815	8	5.417498039	0.978302263	8	4.766195193	1.031391421	8	4.695840731	0.990073769	8	2.732734718	Quantified
Q64324	Syntaxin-binding protein 2	Stxbp2	FUNCTION: Involved in intracellular vesicle t	Syntaxin-binding protein 2	3.2	2	2	2	593	66.31549437	6.741699219	1.023425409	2	2.277610107	1.017578871	2	11.20512872	1.003444443	2	3.973921382	0.986954613	2	0.566320518	0.921279426	2	13.92127128	1.026929615	2	6.254252622	Quantified
Q8CGC7	Bifunctional glutamate/proline--tRNA ligase	Eprs1	FUNCTION: Multifunctional protein which is p	Bifunctional glutamate/proli	1.92	3	3	3	1512	169.9716116	7.664550781	0.991655489	3	8.621139983	0.985265107	3	1.096523178	0.932087157	3	6.336536618	1.070111821	3	9.963501626	1.078383787	3	6.490781674	0.962941674	3	6.248772545	Quantified
Q6P8M1	Putative deoxyribonuclease TATDN1	Tatdn1	FUNCTION: Putative deoxyribonuclease. {EC	Putative deoxyribonuclease	3.73	1	1	1	295	33.34365726	6.176269531	1.025493542	1		1.15847706	1		1.047912662	1		0.817289749	1		1.04837459	1		0.922453291	1		Quantified
Q8BHJ5	F-box-like/WD repeat-containing protein TBL1XR1	Tbl1xr1	FUNCTION: F-box-like protein involved in the	F-box-like/WD repeat-conta	2.53	1	1	1	514	55.62610375	5.630371094	1.0135421	1		1.044004875	1		0.949397443	1		1.01471885	1		1.03698157	1		0.978737532	1		Quantified
P10711	Transcription elongation factor A protein 1	Tcea1	FUNCTION: Necessary for efficient RNA poly	Transcription elongation fac	2.33	1	1	1	301	33.85896505	8.382324219	0.964626191	1		0.96596955	1		1.026526669	1		0.933696227	1		0.966339301	1		1.153400118	1		Quantified
P47226	Testin	Tes	FUNCTION: Scaffold protein that may play a	Testin OS=Mus musculus	3.31	1	1	1	423	47.95112211	8.309082031	0.935344927	1		1.051812004	1		1.04736634	1		0.879431563	1		1.057374532	1		1.066106574	1		Quantified
Q8BL74	General transcription factor 3C polypeptide 2	Gtf3c2	FUNCTION: Required for RNA polymerase III	General transcription factor	0.77	1	1	1	907	100.2111878	6.565917969	0.987482717	1		0.974614629	1		0.94833287	1		1.03062358	1		1.058681361	1		1.05450753	1		Quantified
Q04207	Transcription factor p65	Rela	FUNCTION: NF-kappa-B is a pleiotropic trans	Transcription factor p65 OS	1.46	1	1	1	549	60.17418591	5.757324219	1.028947463	1		1.091551091	1		1.015755212	1		1.001396422	1		0.802458562	1		0.989188197	1		Quantified
P04202	Transforming growth factor beta-1 proprotein	Tgfb1	FUNCTION: Transforming growth factor beta-	Transforming growth factor	2.56	1	1	1	390	44.28166958	8.616699219	0.896930965	1		0.970210373	1		0.914705794	1		1.112359681	1		1.061385449	1		1.107054845	1		Quantified
Q9ER58	Testican-2	Spock2	FUNCTION: May participate in diverse steps	Testican-2 OS=Mus muscu	2.6	1	1	1	423	46.83292944	4.868652344	0.98822926	1		1.08534971	1		1.026532782	1		0.931563187	1		1.07612263	1		0.933070551	1		Quantified
Q62318	Transcription intermediary factor 1-beta	Trim28	FUNCTION: Nuclear corepressor for KRAB d	Transcription intermediary f	4.68	4	4	5	834	88.79053805	5.770019531	0.979759554	5	4.601931633	0.99391481	5	5.838859611	1.052757161	5	6.327840516	0.972661825	5	4.265635788	1.032549862	5	3.039880683	0.997106805	5	3.59448264	Quantified
Q99JR5	Tubulointerstitial nephritis antigen-like	Tinagl1	FUNCTION: May be implicated in the adreno	Tubulointerstitial nephritis a	7.94	3	3	3	466	52.62982606	6.770996094	1.038034626	3	3.410307961	0.896952036	3	21.32900933	0.940008845	3	10.50318659	1.029531416	3	6.524825075	1.053185512	3	1.292747157	1.086824214	3	20.15581638	Quantified
Q5U405	Transmembrane protease serine 13	Tmprss13		Transmembrane protease s	1.47	1	1	1	543	59.76789659	9.012207031	0.92027456	1		0.923741327	1		0.996376864	1		1.062186838	1		1.078932972	1		1.081739842	1		Quantified
O88746	Target of Myb protein 1	Tom1	FUNCTION: May be involved in intracellular t	Target of Myb protein 1 OS	3.46	1	1	1	492	54.29121449	4.944824219	1.051985405	1		1.021582962	1		0.972041062	1		0.938973797	1		1.005816331	1		1.033701215	1		Quantified
Q9JLT2	Trehalase	Treh	FUNCTION: Intestinal trehalase is probably ir	Trehalase OS=Mus muscul	1.22	1	1	1	576	65.35978902	5.579589844	1.010328252	1		0.989253116	1		0.988614844	1		1.053374302	1		1.051776072	1		0.949551645	1		Quantified
Q8C0E3	E3 ubiquitin-protein ligase TRIM47	Trim47	FUNCTION: E3 ubiquitin-protein ligase that m	E3 ubiquitin-protein ligase T	1.72	1	1	1	641	69.86784018	6.404785156	1.033708121	1		0.951350098	1		0.97498009	1		1.008347458	1		0.955142125	1		1.086621659	1		Quantified

Q80XC2	tRNA (adenine(58)-N(1))-methyltransferase catalytic s	Trmt61a	FUNCTION: Catalytic subunit of tRNA (adenin	tRNA (adenine(58)-N(1))-me	5.86	1	1	1	290	31.61887729	6.785644531	1.005282647	1		0.9775707	1		0.941408896	1		1.078800818	1		1.022225078	1		1.013259143	1		Quantified
Q8R3G9	Tetraspanin-8	Tspan8		Tetraspanin-8 OS=Mus mus	5.96	1	1	1	235	25.56512971	5.160644531	1.014135959	1		0.995388913	1		1.162177679	1		0.927718337	1		0.959854211	1		0.924806138	1		Quantified
P07309	Transthyretin	Ttr	FUNCTION: Thyroid hormone-binding protein	Transthyretin OS=Mus mus	8.16	2	2	4	147	15.76598338	6.163574219	0.969096186	1		0.989093448	1		0.952541479	1		1.018127887	1		1.1388266	1		1.017493763	1		Quantified
P26369	Splicing factor U2AF 65 kDa subunit	U2af2	FUNCTION: Plays a role in pre-mRNA splicin	Splicing factor U2AF 65 kD	1.68	1	1	1	475	53.48322495	9.085449219																			Identified
Q91WM3	U3 small nucleolar RNA-interacting protein 2	Rrp9	FUNCTION: Component of a nucleolar small	U3 small nucleolar RNA-int	2.11	1	1	1	475	52.074786	7.884277344	1.14537881	1		0.690853566	1		1.088461344	1		0.983404558	1		1.221738698	1		0.993842372	1		Quantified
Q91YN5	UDP-N-acetylhexosamine pyrophosphorylase	Uap1	FUNCTION: Converts UTP and GlcNAc-1-P i	UDP-N-acetylhexosamine p	6.13	3	3	5	522	58.5715977	6.493652344	0.991735314	5	3.644065217	1.014651211	5	7.38646027	0.993681255	5	12.29326097	0.995112439	5	5.85404739	1.000812648	5	3.973767837	1.000465186	5	1.450194018	Quantified
E9Q735	Ubiquitin conjugation factor E4 A	Ube4a	FUNCTION: Ubiquitin-protein ligase that prob	Ubiquitin conjugation factor	0.68	1	1	1	1028	118.1227005	5.325683594	1.09933591	1		0.978035159	1		0.897669833	1		1.009195669	1		1.07053048	1		1.006430887	1		Quantified
Q9ES00	Ubiquitin conjugation factor E4 B	Ube4b	FUNCTION: Ubiquitin-protein ligase that prob	Ubiquitin conjugation factor	0.77	1	1	1	1173	133.2322408	6.074707031	1.005476631	1		0.966407576	1		1.09918975	1		0.971073169	1		0.983428446	1		0.979113058	1		Quantified
P21126	Ubiquitin-like protein 4A	Ubl4a	FUNCTION: As part of a cytosolic protein qua	Ubiquitin-like protein 4A OS	5.1	1	1	1	157	17.78956642	8.440917969	1.02832635	1		0.986876445	1		0.941439586	1		1.025516466	1		1.108672274	1		0.981347026	1		Quantified
P35123	Ubiquitin carboxyl-terminal hydrolase 4	Usp4	FUNCTION: Deubiquitinating enzyme that rer	Ubiquitin carboxyl-terminal	1.25	1	1	1	962	108.2739328	5.643066406	0.999072089	1		0.96902735	1		1.042040664	1		1.029354927	1		1.000522065	1		0.97816298	1		Quantified
Q6A4J8	Ubiquitin carboxyl-terminal hydrolase 7	Usp7	FUNCTION: Hydrolase that deubiquitinates ta	Ubiquitin carboxyl-terminal	2.36	3	3	4	1103	128.393321	5.503417969	0.990652863	4	1.584733907	0.974638215	4	2.932281921	1.031368105	4	4.842700882	0.957073549	4	7.481559031	1.088256435	4	8.644900898	0.941220923	4	1.579935691	Quantified
P58321	Ubiquitin carboxyl-terminal hydrolase isozyme L4	Uchl4	FUNCTION: Ubiquitin-protein hydrolase is inv	Ubiquitin carboxyl-terminal	3.86	1	1	1	233	26.43331808	5.097167969	0.959705662	1		0.937706221	1		0.905456084	1		1.182014774	1		1.158504236	1		0.958449873	1		Quantified
Q3U1V6	Ubiquitin-conjugating enzyme E2 variant 3	Uevld	FUNCTION: Possible negative regulator of pc	Ubiquitin-conjugating enzyr	1.91	1	1	1	471	51.64878861	7.283691406	0.961355615	1		1.043270836	1		0.958137784	1		0.993327798	1		1.009230276	1		1.062593125	1		Quantified
Q6P5E4	UDP-glucose:glycoprotein glucosyltransferase 1	Uggt1	FUNCTION: Recognizes glycoproteins with m	UDP-glucose:glycoprotein g	0.97	2	2	2	1551	176.3228342	5.617675781	1.081176494	2	8.260191604	0.926807591	2	7.54743047	1.012876253	2	0.387013829	0.975834843	2	2.698562426	0.969575112	2	2.737604381	1.042131926	2	2.147505161	Quantified
O70404	Vesicle-associated membrane protein 8	Vamp8	FUNCTION: SNAREs, soluble N-ethylmaleim	Vesicle-associated membra	7.92	1	1	1	101	11.44411992	8.191894531	0.991226469	1		0.951825708	1		0.989911586	1		1.030280131	1		1.039997344	1		1.040021854	1		Quantified
P35969	Vascular endothelial growth factor receptor 1	Flt1	FUNCTION: Tyrosine-protein kinase that acts	Vascular endothelial growth	1.88	1	1	1	1333	149.7811309	8.309082031	0.765822571	1		1.137021277	1		1.256305525	1		1.096739773	1		0.938711174	1		0.760435994	1		Quantified
Q8VDJ3	Vigilin	Hdlbp	FUNCTION: Appears to play a role in cell ste	Vigilin OS=Mus musculus (	2.52	4	4	5	1268	141.6552814	6.873535156	1.02511041	5	4.563275474	1.032204559	5	5.909486826	0.961213302	5	4.389621635	0.993941094	5	2.567174658	1.024664568	5	2.098500182	0.985320146	5	6.083441866	Quantified
Q9Z0K8	Pantetheinase	Vnn1	FUNCTION: Amidohydrolase that hydrolyzes	Pantetheinase OS=Mus mu	2.54	1	1	1	512	57.05429747	5.935058594	1.006190636	1		0.992253301	1		1.035477122	1		0.962529243	1		1.171120259	1		0.918243447	1		Quantified
P40336	Vacuolar protein sorting-associated protein 26A	Vps26a	FUNCTION: Acts as component of the retrom	Vacuolar protein sorting-as	9.48	2	2	3	327	38.08968445	6.565917969	0.963306088	3	3.333151782	1.076216519	3	2.453792284	0.97520632	3	1.504806445	0.99875945	3	2.261397003	1.042160536	3	5.365533787	0.986754048	3	1.813999013	Quantified
Q91XD6	Vacuolar protein-sorting-associated protein 36	Vps36	FUNCTION: Component of the ESCRT-II com	Vacuolar protein-sorting-as	8.29	3	3	3	386	43.70767625	7.151855469	1.046771194	3	4.332787527	0.933075497	3	1.76705522	0.943884919	3	3.722688502	1.08811813	3	1.542962811	1.122950062	3	3.323546384	0.98916024	3	0.747611278	Quantified
Q9CR26	Vacuolar protein sorting-associated protein VTA1 hor	Vta1	FUNCTION: Involved in the endosomal multiv	Vacuolar protein sorting-as	2.59	1	1	2	309	33.89190733	6.125488281	0.930095367	2	7.206146248	0.910559339	2	1.104477005	1.005629807	2	0.462208345	1.060719259	2	5.743477031	1.068340871	2	1.19703374	1.081317416	2	1.518018212	Quantified
A6PWY4	WD repeat-containing protein 76	Wdr76	FUNCTION: Specifically binds 5-hydroxymeth	WD repeat-containing prote	1.29	1	1	1	622	68.96443581	9.363769531	1.016500162	1		0.758130991	1		0.949851513	1		1.235948696	1		1.223840398	1		0.952635807	1		Quantified
Q9DBR1	5'-3' exoribonuclease 2	Xrn2	FUNCTION: Possesses 5'->3' exoribonucleas	5'-3' exoribonuclease 2 OS	0.74	1	1	1	951	108.6182592	7.591308594	1.015406213	1		1.033098521	1		1.033310747	1		0.95235012	1		0.990133018	1		0.98517224	1		Quantified
Q9EPK2	Protein XRP2	Rp2	FUNCTION: Acts as a GTPase-activating pro	Protein XRP2 OS=Mus mus	2.31	1	1	1	347	39.3511663	5.236816406	0.94480989	1		0.969403763	1		0.996430238	1		1.047815285	1		1.018461952	1		1.056663827	1		Quantified
Q04736	Tyrosine-protein kinase Yes	Yes1	FUNCTION: Non-receptor protein tyrosine kin	Tyrosine-protein kinase Yes	4.25	1	3	5	541	60.59146181	6.639160156	0.995281899	3	1.493901411	1.009549264	3	3.22756419	1.051280347	3	4.718693597	0.967424237	3	0.79359168	1.010250287	3	4.427008912	1.016655901	3	3.793566442	Quantified
P59326	YTH domain-containing family protein 1	Ythdf1	FUNCTION: Specifically recognizes and bind	YTH domain-containing fam	1.61	1	1	1	559	60.84099614	8.953613281	1.006467997	1		0.948956634	1		1.014346803	1		0.937271102	1		1.115274993	1		1.049204763	1		Quantified
Q0P678	Zinc finger CCCH domain-containing protein 18	Zc3h18		Zinc finger CCCH domain-c	1.37	1	1	1	948	105.63119	7.796386719	1.059772985	1		0.928198865	1		1.041076252	1		0.970778202	1		1.137172444	1		0.938686902	1		Quantified
A2A791	Zinc finger MYM-type protein 4	Zmym4	FUNCTION: Plays a role in the regulation of c	Zinc finger MYM-type prote	0.58	1	1	1	1549	172.3264595	6.800292969	1.04694624	1		0.957560895	1		0.94939993	1		1.059515032	1		0.94616827	1		1.048202278	1		Quantified
Q61116	Zinc finger protein 235	Znf235	FUNCTION: May be involved in transcriptiona	Zinc finger protein 235 OS=	1.24	1	1	1	645	72.96512128	8.602050781	0.870793765	1		0.850535899	1		0.89195162	1		1.213937753	1		1.321449792	1		1.032347023	1		Quantified
Q9Z0U1	Tight junction protein ZO-2	Tjp2	FUNCTION: Plays a role in tight junctions and	Tight junction protein ZO-2	0.69	1	1	1	1167	131.19999	6.785644531	0.929436363	1		0.96695698	1		1.07119808	1		0.987492211	1		0.911240819	1		1.11636002	1		Quantified
Q8R121	Protein Z-dependent protease inhibitor	Serpina10	FUNCTION: Inhibits activity of the coagulatio	Protein Z-dependent protea	1.79	1	1	1	448	51.76438146	5.668457031	1.014018331	1		1.114293684	1		1.044219088	1		0.928567456	1		0.922377964	1		0.949533961	1		Quantified
Q9CZN7	Serine hydroxymethyltransferase, mitochondrial	Shmt2	FUNCTION: Catalyzes the cleavage of serine	Serine hydroxymethyltransf	2.98	1	1	1	504	55.72360949	8.470214844																			Identified
Q91XZ4	Protocadherin beta-6	Pcdhb6	FUNCTION: Calcium-dependent cell-adhesio	Protocadherin beta-6 OS=M	1.3	1	1	1	772	84.2278045	4.792480469	1.023335766	1		0.983216408	1		0.988725832	1		1.042839641	1		0.981269463	1		0.995663458	1		Quantified
Q7TNC9	Inositol polyphosphate-5-phosphatase A	Inpp5a		Inositol polyphosphate-5-ph	2.18	1	1	1	412	47.59165164	6.917480469	1.07263485	1		1.05507014	1		1.072753776	1		0.883759761	1		0.954728908	1		0.947608382	1		Quantified
Q8BML9	Glutamine--tRNA ligase	Qars1	FUNCTION: Glutamine--tRNA ligase. Plays a	Glutamine--tRNA ligase OS	1.16	1	1	1	775	87.62087236	7.312988281	0.917253108	1		1.06120757	1		1.083101759	1		0.940016149	1		0.967875621	1		1.025854109	1		Quantified
E9Q4N7	AT-rich interactive domain-containing protein 1B	Arid1b	FUNCTION: Involved in transcriptional activat	AT-rich interactive domain-	0.31	1	1	1	2244	237.6447751	6.727050781	1.069512641	1		0.787343393	1		1.10355404	1		1.008755659	1		1.212922619	1		0.928661912	1		Quantified
E9Q5C9	Nucleolar and coiled-body phosphoprotein 1	Nolc1	FUNCTION: Nucleolar protein that acts as a r	Nucleolar and coiled-body p	1.28	1	1	1	702	73.65433649	9.363769531	0.93409959	1		1.022062413	1		1.15874566	1		1.023897746	1		0.922972412	1		0.906721024	1		Quantified
E9PVA8	eIF-2-alpha kinase activator GCN1	Gcn1	FUNCTION: Acts as a positive activator of the	eIF-2-alpha kinase activato	0.3	1	1	1	2671	292.8343131	7.356933594	1.054734833	1		0.907364976	1		0.976137425	1		0.953477372	1		1.109502767	1		1.075190792	1		Quantified
E9Q2M9	WD repeat- and FYVE domain-containing protein 4	Wdfy4	FUNCTION: Plays a critical role in the regulat	WD repeat- and FYVE dom	0.38	1	1	1	3183	354.1041708	6.341308594	1.051649584	1		1.005628313	1		0.917916476	1		1.016088611	1		1.009668598	1		1.032217532	1		Quantified

Header	Description
Accession	Identifier of the protein in the FASTA. database
Protein name	Shown annotation information of protein name in FASTA database.
Gene name	Shown annotation information of gene name in FASTA database.
Function	Protein function annotaion.
Description	Shown description of the protein in the database.
Coverage	The percentage of the protein sequence covered by identified peptides.
Unique Peptides	The number of peptide sequences unique to a protein group.
Peptides	The number of distinct peptide sequences in the protein group.
PSMs	Peptide spectrum matches:The total number of identified peptide sequences for the protein, including those redundantly identified.
AAs	The number of amino acids in the protein.
MW [kDa]	Calculated molecular weight of the protein.
calc. pI	Calculated protein isoelectric point.
Ratio (XXX/REF)	Corrected ratio of the signal intensity of the labeled channel of the sample to the reported ion peak signal value of the internal reference (REF).
Ratio Count	The number of peptide ratios that were actually used to calculate a particular protein ratio.
Ratio Variability [%]	The variability of peptide ratios that were actually used to calculate a particular protein ratio.
Identified or Quantified	Quantified :Proteins have intensities in more than half of the biological repeats in at least one group
