# Supplementary material for: Proteomic Profiling and Functional Analysis of B Cell-Derived Exosomes upon Pneumocystis Infection
Source: J Immunol Res. 2022 Apr 14;2022:5187166. doi: 10.1155/2022/5187166 (PMC9023222; doi:10.1155/2022/5187166)
Supplement: Supplementary Materials — Supplementary Table 1: initial culture cell number and protein amount of B cell exosomes used for mass spectrometry. Supplementary Table 2: all identified proteins of uninfected and Pneumocystis-infected B cell exosomes. Supplementary Table 3: SignalP and SecretomeP prediction results. Supplementary Table 4: differentially expressed proteins of B cell exosomes in response to PCP. Supplementary Table 5: quantitative information of peptides and proteins by PRM analysis. [file 5187166.f1.zip › Supplementary Table 3-SignalP and SecretomeP prediction results.pdf]

Supplementary Table 3：SignalP and SecretomeP prediction results

Accession	Protein Name	Gene Name	SP(Sec/SPI)	CS Position	SignalP	NN-score	Secretome P
Q5SX39	Myosin-4	Myh4	＜0.5		N	≤0.5	N
P43277	Histone H1.3	H1-3	＜0.5		N	≤0.5	N
O35744	Chitinase-like protein 3	Chil3	0.992	CS pos: 21-22. GSS-YQ. Pr: 0.8601	Y	0.648	N
P27661	Histone H2AX	H2ax	＜0.5		N	0.53	Y
Q9CQE8	RNA transcription, translation and transport fac	RTRAF	＜0.5		N	≤0.5	N
P43274	Histone H1.4	H1-4	＜0.5		N	≤0.5	N
P56716	Oxygen-regulated protein 1	Rp1	＜0.5		N	≤0.5	N
O54962	Barrier-to-autointegration factor	Banf1	＜0.5		N	0.534	Y
P0C0S6	Histone H2A.Z	H2az1	＜0.5		N	0.58	Y
P43276	Histone H1.5	H1-5	＜0.5		N	≤0.5	N
Q91Z83	Myosin-7	Myh7	＜0.5		N	≤0.5	N
Q6GSS7	Histone H2A type 2-A	Hist2h2aa1	＜0.5		N	≤0.5	N
P15864	Histone H1.2	H1-2	＜0.5		N	≤0.5	N
P68433	Histone H3.1	H3c1	＜0.5		N	0.681	Y
Q8CGP7	Histone H2A type 1-K	H2ac15	＜0.5		N	0.543	Y
Q3UPH1	Protein PRRC1	Prrc1	＜0.5		N	0.801	Y
P62806	Histone H4	H4c1	＜0.5		N	≤0.5	N
Q8CGP0	Histone H2B type 3-B	H2bu1	＜0.5		N	≤0.5	N
P10854	Histone H2B type 1-M	H2bc14	＜0.5		N	≤0.5	N
P10922	Histone H1.0	H1-0	＜0.5		N	≤0.5	N
P02301	Histone H3.3C	H3-5	＜0.5		N	0.709	Y
P70296	Phosphatidylethanolamine-binding protein 1	Pebp1	＜0.5		N	≤0.5	N
P19467	Mucin-13	Muc13	0.990	CS pos: 22-23. SQA-SS. Pr: 0.5767	Y	≤0.5	N
P16331	Phenylalanine-4-hydroxylase	Pah	＜0.5		N	≤0.5	N
Q61116	Zinc finger protein 235	Znf235	＜0.5		N	≤0.5	N
Q9CZT6	Protein CMSS1	Cmss1	＜0.5		N	0.673	Y
Q9Z277	Tyrosine-protein kinase BAZ1B	Baz1b	＜0.5		N	≤0.5	N
P47915	60S ribosomal protein L29	Rpl29	＜0.5		N	≤0.5	N
P20152	Vimentin	Vim	＜0.5		N	0.728	Y
Q60870	Receptor expression-enhancing protein 5	Reep5	＜0.5		N	≤0.5	N
Q8C2K1	Differentially expressed in FDCP 6	Def6	＜0.5		N	0.64	Y
Q8K012	Formin-binding protein 1-like	Fnbp1l	＜0.5		N	≤0.5	N
Q3SXB8	Collectin-11	Colec11	0.968	CS pos: 26-27. GCP-QQ. Pr: 0.3626	Y	0.741	N
P42586	Homeobox protein Nkx-2.2	Nkx2-2	＜0.5		N	0.943	Y
P48678	Prelamin-A/C	Lmna	＜0.5		N	≤0.5	N
P11911	B-cell antigen receptor complex-associated pr	Cd79a	0.997	CS pos: 28-29. CQA-LR. Pr: 0.9570	Y	0.641	N
P50396	Rab GDP dissociation inhibitor alpha	Gdi1	＜0.5		N	≤0.5	N
Q7TSJ6	Serine/threonine-protein kinase LATS2	Lats2	＜0.5		N	≤0.5	N
Q04750	DNA topoisomerase 1	Top1	＜0.5		N	≤0.5	N
P01749	Ig heavy chain V region 3	Ighv1-61	0.999	CS pos: 19-20. VHS-QV. Pr: 0.9228	Y	0.852	N
P00688	Pancreatic alpha-amylase	Amy2	0.999	CS pos: 15-16. CWA-QY. Pr: 0.9642	Y	0.786	N
O55143	Sarcoplasmic/endoplasmic reticulum calcium	Atp2a2	＜0.5		N	0.712	Y
Q9QZR9	Collagen alpha-4(IV) chain	Col4a4	0.845	CS pos: 32-33. GSG-KK. Pr: 0.6954	Y	≤0.5	N
Q921G7	Electron transfer flavoprotein-ubiquinone oxido	Etfdh	＜0.5		N	0.65	Y
P62309	Small nuclear ribonucleoprotein G	Snrpg	＜0.5		N	0.604	Y
Q3UQN2	F-BAR domain only protein 2	Fcho2	＜0.5		N	≤0.5	N
Q62418	Drebrin-like protein	Dbnl	＜0.5		N	0.744	Y
O89053	Coronin-1A	Coro1a	＜0.5		N	0.773	Y
Q9QYB5	Gamma-adducin	Add3	＜0.5		N	≤0.5	N
Q3TWW8	Serine/arginine-rich splicing factor 6	Srsf6	＜0.5		N	≤0.5	N
Q9Z130	Heterogeneous nuclear ribonucleoprotein D-lik	Hnrnpdl	＜0.5		N	≤0.5	N
P26039	Talin-1	Tln1	＜0.5		N	≤0.5	N
P07901	Heat shock protein HSP 90-alpha	Hsp90aa1	＜0.5		N	≤0.5	N
P11499	Heat shock protein HSP 90-beta	Hsp90ab1	＜0.5		N	≤0.5	N
Q8BTM8	Filamin-A	Flna	＜0.5		N	≤0.5	N
Q8VDD5	Myosin-9	Myh9	＜0.5		N	≤0.5	N
P58252	Elongation factor 2	Eef2	＜0.5		N	≤0.5	N
P52480	Pyruvate kinase PKM	Pkm	＜0.5		N	≤0.5	N
P17182	Alpha-enolase	Eno1	＜0.5		N	≤0.5	N
Q01853	Transitional endoplasmic reticulum ATPase	Vcp	＜0.5		N	≤0.5	N
P09405	Nucleolin	Ncl	＜0.5		N	≤0.5	N
P26041	Moesin	Msn	＜0.5		N	0.558	Y
P99024	Tubulin beta-5 chain	Tubb5	＜0.5		N	≤0.5	N

Q61292	Laminin subunit beta-2	Lamb2	0.929	CS pos: 37-38. AQA-PS. Pr: 0.3539	Y	≤0.5	N
Q62009	Periostin	Postn	0.999	CS pos: 23-24. ANA-NS. Pr: 0.9633	Y	≤0.5	N
Q02053	Ubiquitin-like modifier-activating enzyme 1	Uba1	< 0.5		N	0.551	Y
P40124	Adenylyl cyclase-associated protein 1	Cap1	< 0.5		N	≤0.5	N
P16858	Glyceraldehyde-3-phosphate dehydrogenase	Gapdh	< 0.5		N	0.541	Y
Q9JKF1	Ras GTPase-activating-like protein IQGAP1	Iqgap1	< 0.5		N	≤0.5	N
Q91ZX7	Prolow-density lipoprotein receptor-related pro	Lrp1	1.000	CS pos: 19-20. VSG-AT. Pr: 0.7616	Y	≤0.5	N
P17156	Heat shock-related 70 kDa protein 2	Hspa2	< 0.5		N	≤0.5	N
P40142	Transketolase	Tkt	< 0.5		N	≤0.5	N
P61979	Heterogeneous nuclear ribonucleoprotein K	Hnrnpk	< 0.5		N	≤0.5	N
O54724	Caveolae-associated protein 1	Cavin1	< 0.5		N	0.722	Y
P06151	L-lactate dehydrogenase A chain	Ldha	< 0.5		N	0.568	Y
O08553	Dihydropyrimidinase-related protein 2	Dpysl2	< 0.5		N	≤0.5	N
Q99JY9	Actin-related protein 3	Actr3	< 0.5		N	≤0.5	N
Q9DCD0	6-phosphogluconate dehydrogenase, decarbo:	Pgd	< 0.5		N	≤0.5	N
Q60710	Deoxynucleoside triphosphate triphosphohydr	Samhd1	< 0.5		N	≤0.5	N
P21619	Lamin-B2	Lmnb2	< 0.5		N	≤0.5	N
Q61316	Heat shock 70 kDa protein 4	Hspa4	< 0.5		N	≤0.5	N
P80314	T-complex protein 1 subunit beta	Cct2	< 0.5		N	≤0.5	N
P26040	Ezrin	Ezr	< 0.5		N	0.585	Y
Q8K4G1	Latent-transforming growth factor beta-binding	Ltbp4	0.999	CS pos: 26-27. ASG-SS. Pr: 0.3514	Y	≤0.5	N
P68254	14-3-3 protein theta	Ywhaq	< 0.5		N	≤0.5	N
Q9QZQ8	Core histone macro-H2A.1	Macroh2a1	< 0.5		N	≤0.5	N
P42932	T-complex protein 1 subunit theta	Cct8	< 0.5		N	0.539	Y
P47753	F-actin-capping protein subunit alpha-1	Capza1	< 0.5		N	≤0.5	N
Q9Z1Q5	Chloride intracellular channel protein 1	Clic1	< 0.5		N	≤0.5	N
Q9EQP2	EH domain-containing protein 4	Ehd4	< 0.5		N	≤0.5	N
Q61233	Plastin-2	Lcp1	< 0.5		N	0.51	Y
P61982	14-3-3 protein gamma	Ywhag	< 0.5		N	≤0.5	N
P0CG49	Polyubiquitin-B	Ubb	< 0.5		N	≤0.5	N
P07356	Annexin A2	Anxa2	< 0.5		N	0.739	Y
P47757	F-actin-capping protein subunit beta	Capzb	< 0.5		N	0.543	Y
P40936	Indolethylamine N-methyltransferase	Inmt	< 0.5		N	0.53	Y
Q6A028	Switch-associated protein 70	Swap70	< 0.5		N	≤0.5	N
Q8K0E8	Fibrinogen beta chain	Fgb	0.999	CS pos: 19-20. TQA-AD. Pr: 0.9071	Y	0.564	N
P09411	Phosphoglycerate kinase 1	Pgk1	< 0.5		N	≤0.5	N
Q71LX4	Talin-2	Tln2	< 0.5		N	≤0.5	N
P11983	T-complex protein 1 subunit alpha	Tcp1	< 0.5		N	≤0.5	N
O08638	Myosin-11	Myh11	< 0.5		N	≤0.5	N
P32261	Antithrombin-III	Serpinc1	0.988	CS pos: 34-35. CHG-NP. Pr: 0.7891	Y	0.577	N
P29351	Tyrosine-protein phosphatase non-receptor ty	Ptpn6	< 0.5		N	≤0.5	N
P80313	T-complex protein 1 subunit eta	Cct7	< 0.5		N	≤0.5	N
P14824	Annexin A6	Anxa6	< 0.5		N	≤0.5	N
Q9Z1Q9	Valine--tRNA ligase	Vars1	< 0.5		N	≤0.5	N
Q62465	Synaptic vesicle membrane protein VAT-1 hor	Vat1	< 0.5		N	0.559	Y
Q6URW6	Myosin-14	Myh14	< 0.5		N	≤0.5	N
P21981	Protein-glutamine gamma-glutamyltransferase	Tgm2	< 0.5		N	0.586	Y
P70168	Importin subunit beta-1	Kpnb1	< 0.5		N	0.592	Y
P28352	DNA-(apurinic or apyrimidinic site) endonuclea	Apex1	< 0.5		N	0.663	Y
P14152	Malate dehydrogenase, cytoplasmic	Mdh1	< 0.5		N	≤0.5	N
Q60668	Heterogeneous nuclear ribonucleoprotein D0	Hnrnpd	< 0.5		N	≤0.5	N
Q8BGQ7	Alanine--tRNA ligase, cytoplasmic	Aars1	< 0.5		N	≤0.5	N
Q99JI6	Ras-related protein Rap-1b	Rap1b	< 0.5		N	0.692	Y
P28656	Nucleosome assembly protein 1-like 1	Nap1l1	< 0.5		N	≤0.5	N
P30681	High mobility group protein B2	Hmgb2	< 0.5		N	≤0.5	N
P27773	Protein disulfide-isomerase A3	Pdia3	0.991	CS pos: 24-25. AAA-SD. Pr: 0.6527	Y	≤0.5	N
P08074	Carbonyl reductase [NADPH] 2	Cbr2	< 0.5		N	0.588	Y
Q9D8N0	Elongation factor 1-gamma	Eef1g	< 0.5		N	≤0.5	N
Q9QXS1	Plectin	Plec	< 0.5		N	≤0.5	N
Q91ZW3	SWI/SNF-related matrix-associated actin-depe	Smarca5	< 0.5		N	≤0.5	N
Q8CCK0	Core histone macro-H2A.2	Macroh2a2	< 0.5		N	≤0.5	N
Q06806	Tyrosine-protein kinase receptor Tie-1	Tie1	0.956	CS pos: 21-22. VGA-SV. Pr: 0.8193	Y	≤0.5	N
P17225	Polypyrimidine tract-binding protein 1	Ptbp1	< 0.5		N	≤0.5	N
Q9Z1N5	Spliceosome RNA helicase Ddx39b	Ddx39b	< 0.5		N	0.513	Y
P62874	Guanine nucleotide-binding protein G(I)/G(S)/(	Gnb1	< 0.5		N	≤0.5	N
P24452	Macrophage-capping protein	Capg	< 0.5		N	≤0.5	N

P46471	26S proteasome regulatory subunit 7	Psmc2	< 0.5		N	0.713	Y
P08003	Protein disulfide-isomerase A4	Pdia4	0.997	CS pos: 24-25. ASA-GD. Pr: 0.4930	Y	0.523	N
Q8C1B7	Septin-11	Septin11	< 0.5		N	≤0.5	N
Q78ZA7	Nucleosome assembly protein 1-like 4	Nap1l4	< 0.5		N	≤0.5	N
P80318	T-complex protein 1 subunit gamma	Cct3	< 0.5		N	≤0.5	N
Q9D554	Splicing factor 3A subunit 3	Sf3a3	< 0.5		N	≤0.5	N
Q93092	Transaldolase	Taldo1	< 0.5		N	≤0.5	N
Q8BK67	Protein RCC2	Rcc2	< 0.5		N	≤0.5	N
P68040	Receptor of activated protein C kinase 1	Rack1	< 0.5		N	≤0.5	N
O35375	Neuropilin-2	Nrp2	0.996	CS pos: 22-23. VRS-QQ. Pr: 0.9317	Y	0.513	N
P50580	Proliferation-associated protein 2G4	Pa2g4	< 0.5		N	≤0.5	N
O88543	COP9 signalosome complex subunit 3	Cops3	< 0.5		N	0.654	Y
P97321	Prolyl endopeptidase FAP	Fap	< 0.5		N	0.699	Y
Q8BG05	Heterogeneous nuclear ribonucleoprotein A3	Hnrnpa3	< 0.5		N	≤0.5	N
Q91WN1	DnaJ homolog subfamily C member 9	Dnajc9	< 0.5		N	0.81	Y
Q9WTI7	Unconventional myosin-Ic	Myo1c	< 0.5		N	≤0.5	N
P62880	Guanine nucleotide-binding protein G(I)/G(S)/(	Gnb2	< 0.5		N	≤0.5	N
Q78PY7	Staphylococcal nuclease domain-containing p	Snd1	< 0.5		N	≤0.5	N
Q9QZE5	Coatomer subunit gamma-1	Copg1	< 0.5		N	≤0.5	N
Q91WQ3	Tyrosine--tRNA ligase, cytoplasmic	Yars1	< 0.5		N	≤0.5	N
Q8BKG3	Inactive tyrosine-protein kinase 7	Ptk7	0.986	CS pos: 22-23. AQA-AI. Pr: 0.9356	Y	≤0.5	N
O70475	UDP-glucose 6-dehydrogenase	Ugdh	< 0.5		N	≤0.5	N
Q00519	Xanthine dehydrogenase/oxidase	Xdh	< 0.5		N	0.504	Y
P62814	V-type proton ATPase subunit B, brain isoform	Atp6v1b2	< 0.5		N	0.58	Y
Q9R0P3	S-formylglutathione hydrolase	Esd	< 0.5		N	0.595	Y
Q6P8X1	Sorting nexin-6	Snx6	< 0.5		N	≤0.5	N
P54822	Adenylosuccinate lyase	Adsl	< 0.5		N	0.54	Y
Q8R016	Bleomycin hydrolase	Blmh	< 0.5		N	0.526	Y
P35700	Peroxiredoxin-1	Prdx1	< 0.5		N	0.542	Y
O55131	Septin-7	Septin7	< 0.5		N	≤0.5	N
Q922R8	Protein disulfide-isomerase A6	Pdia6	0.981	CS pos: 19-20. VSG-LY. Pr: 0.3577	Y	0.684	N
P17751	Triosephosphate isomerase	Tpi1	< 0.5		N	≤0.5	N
P14211	Calreticulin	Calr	0.998	CS pos: 17-18. AAA-DP. Pr: 0.9185	Y	≤0.5	N
Q9WUM4	Coronin-1C	Coro1c	< 0.5		N	0.604	Y
Q8CHH9	Septin-8	Septin8	< 0.5		N	≤0.5	N
P06800	Receptor-type tyrosine-protein phosphatase C	Ptprc	0.893	CS pos: 25-26. VTG-QT. Pr: 0.4418	Y	≤0.5	N
Q3TBD2	Rho GTPase-activating protein 45	Arhgap45	< 0.5		N	≤0.5	N
Q99MN1	Lysine--tRNA ligase	Kars1	< 0.5		N	≤0.5	N
Q6P1F6	Serine/threonine-protein phosphatase 2A 55 k	Ppp2r2a	< 0.5		N	≤0.5	N
P84091	AP-2 complex subunit mu	Ap2m1	< 0.5		N	≤0.5	N
E9Q7G0	Nuclear mitotic apparatus protein 1	Numa1	< 0.5		N	≤0.5	N
Q9JK38	Glucosamine 6-phosphate N-acetyltransferase	Gnpnat1	< 0.5		N	0.586	Y
P97447	Four and a half LIM domains protein 1	Fhl1	< 0.5		N	≤0.5	N
Q9R062	Glycogenin-1	Gyg1	< 0.5		N	0.515	Y
Q9CQI6	Coactosin-like protein	Cotl1	< 0.5		N	0.737	Y
Q61656	Probable ATP-dependent RNA helicase DDX5	Ddx5	< 0.5		N	0.502	Y
Q9WTM5	RuvB-like 2	Ruvbl2	< 0.5		N	≤0.5	N
Q62469	Integrin alpha-2	Itga2	0.937	CS pos: 26-27. CLA-YN. Pr: 0.8671	Y	0.781	N
P17563	Methanethiol oxidase	Selenbp1	< 0.5		N	0.552	Y
P59999	Actin-related protein 2/3 complex subunit 4	Arpc4	< 0.5		N	0.646	Y
P26443	Glutamate dehydrogenase 1, mitochondrial	Glud1	< 0.5		N	≤0.5	N
Q80XI4	Phosphatidylinositol 5-phosphate 4-kinase typ	Pip4k2b	< 0.5		N	≤0.5	N
P62908	40S ribosomal protein S3	Rps3	< 0.5		N	≤0.5	N
Q8BV49	Pyrin and HIN domain-containing protein 1	Pyhin1	< 0.5		N	≤0.5	N
Q9R1T4	Septin-6	Septin6	< 0.5		N	≤0.5	N
P35396	Peroxisome proliferator-activated receptor delt	Ppard	< 0.5		N	≤0.5	N
Q8VE97	Serine/arginine-rich splicing factor 4	Srsf4	< 0.5		N	≤0.5	N
Q99K48	Non-POU domain-containing octamer-binding	Nono	< 0.5		N	≤0.5	N
Q00612	Glucose-6-phosphate 1-dehydrogenase X	G6pdx	< 0.5		N	≤0.5	N
Q3U1J4	DNA damage-binding protein 1	Ddb1	< 0.5		N	0.554	Y
Q8CIB5	Fermitin family homolog 2	Fermt2	< 0.5		N	≤0.5	N
Q6PDG5	SWI/SNF complex subunit SMARCC2	Smarcc2	< 0.5		N	≤0.5	N
P31230	Aminoacyl tRNA synthase complex-interacting	Aimp1	< 0.5		N	0.505	Y
Q9CR57	60S ribosomal protein L14	Rpl14	< 0.5		N	≤0.5	N
Q91Y47	Coagulation factor XI	F11	0.940	CS pos: 18-19. VSS-EC. Pr: 0.4317	Y	0.587	N
Q80TY0	Formin-binding protein 1	Fnbp1	< 0.5		N	≤0.5	N

P61226	Ras-related protein Rap-2b	Rap2b	< 0.5		N	0.775	Y
Q9JKX3	Transferrin receptor protein 2	Tfr2	< 0.5		N	0.668	Y
Q99J77	Sialic acid synthase	Nans	< 0.5		N	0.611	Y
Q99L45	Eukaryotic translation initiation factor 2 subuni	Eif2s2	< 0.5		N	≤0.5	N
Q9QYC0	Alpha-adducin	Add1	< 0.5		N	≤0.5	N
Q91WJ8	Far upstream element-binding protein 1	Fubp1	< 0.5		N	≤0.5	N
P63154	Crooked neck-like protein 1	Crnkl1	< 0.5		N	≤0.5	N
P62082	40S ribosomal protein S7	Rps7	< 0.5		N	0.905	Y
Q3TEA8	Heterochromatin protein 1-binding protein 3	Hp1bp3	< 0.5		N	≤0.5	N
Q3UPL0	Protein transport protein Sec31A	Sec31a	< 0.5		N	≤0.5	N
F6ZDS4	Nucleoprotein TPR	Tpr	< 0.5		N	≤0.5	N
P01837	Immunoglobulin kappa constant	Igkc	< 0.5		N	0.599	Y
P62071	Ras-related protein R-Ras2	Rras2	< 0.5		N	0.85	Y
Q04519	Sphingomyelin phosphodiesterase	Smpd1	< 0.5		N	0.669	Y
Q8BYW1	Rho GTPase-activating protein 25	Arhgap25	< 0.5		N	≤0.5	N
Q91VH2	Sorting nexin-9	Snx9	< 0.5		N	0.501	Y
O88545	COP9 signalosome complex subunit 6	Cops6	< 0.5		N	0.533	Y
Q8BGB5	LIM domain-containing protein 2	Limd2	< 0.5		N	0.707	Y
Q6P4T2	U5 small nuclear ribonucleoprotein 200 kDa h	Snrnp200	< 0.5		N	≤0.5	N
Q9Z1F9	SUMO-activating enzyme subunit 2	Uba2	< 0.5		N	0.533	Y
Q61147	Ceruloplasmin	Cp	0.994	CS pos: 19-20. ALA-RD. Pr: 0.8935	Y	0.632	N
Q8BRF7	Sec1 family domain-containing protein 1	Scfd1	< 0.5		N	≤0.5	N
P07310	Creatine kinase M-type	Ckm	< 0.5		N	≤0.5	N
P25444	40S ribosomal protein S2	Rps2	< 0.5		N	0.631	Y
P99029	Peroxiredoxin-5, mitochondrial	Prdx5	< 0.5		N	0.682	Y
Q9JLI8	Squamous cell carcinoma antigen recognized	Sart3	< 0.5		N	≤0.5	N
Q8CAY6	Acetyl-CoA acetyltransferase, cytosolic	Acat2	< 0.5		N	0.733	Y
P70441	Na(+)/H(+) exchange regulatory cofactor NHE-	Slc9a3r1	< 0.5		N	≤0.5	N
Q9R118	Serine protease HTRA1	Htra1	1.000	CS pos: 22-23. SLA-LP. Pr: 0.7355	Y	0.754	N
Q3THE2	Myosin regulatory light chain 12B	Myl12b	< 0.5		N	≤0.5	N
P80316	T-complex protein 1 subunit epsilon	Cct5	< 0.5		N	≤0.5	N
Q9D379	Epoxide hydrolase 1	Ephx1	< 0.5		N	0.564	Y
O70251	Elongation factor 1-beta	Eef1b	< 0.5		N	0.655	Y
Q9D0I9	Arginine--tRNA ligase, cytoplasmic	Rars1	< 0.5		N	≤0.5	N
Q62010	Oviduct-specific glycoprotein	Ovgp1	0.993	CS pos: 19-20. SDG-TA. Pr: 0.5917	Y	≤0.5	N
Q3UQ44	Ras GTPase-activating-like protein IQGAP2	Iqgap2	< 0.5		N	≤0.5	N
Q3UQU0	Bromodomain-containing protein 9	Brd9	< 0.5		N	≤0.5	N
P97461	40S ribosomal protein S5	Rps5	< 0.5		N	0.78	Y
Q8BH43	Wiskott-Aldrich syndrome protein family meml	Wasf2	< 0.5		N	≤0.5	N
Q9CQF9	Prenylcysteine oxidase	Pcyox1	0.985	CS pos: 28-29. ASA-EP. Pr: 0.6167	Y	0.687	N
Q9D3E6	Cohesin subunit SA-1	Stag1	< 0.5		N	≤0.5	N
Q80X19	Collagen alpha-1(XIV) chain	Col14a1	0.919	CS pos: 28-29. VRG-QV. Pr: 0.7398	Y	≤0.5	N
Q9QYJ0	DnaJ homolog subfamily A member 2	Dnaja2	< 0.5		N	≤0.5	N
P56480	ATP synthase subunit beta, mitochondrial	Atp5f1b	< 0.5		N	0.594	Y
Q9CQI7	U2 small nuclear ribonucleoprotein B''	Snrpb2	< 0.5		N	0.751	Y
Q9CR16	Peptidyl-prolyl cis-trans isomerase D	Ppid	< 0.5		N	≤0.5	N
O55128	Histone deacetylase complex subunit SAP18	Sap18	< 0.5		N	0.823	Y
Q80XA6	RalBP1-associated Eps domain-containing pro	Reps2	< 0.5		N	≤0.5	N
Q9WUA2	Phenylalanine--tRNA ligase beta subunit	Farsb	< 0.5		N	≤0.5	N
P35991	Tyrosine-protein kinase BTK	Btk	< 0.5		N	≤0.5	N
Q61136	Serine/threonine-protein kinase PRP4 homolo	Prpf4b	< 0.5		N	≤0.5	N
P35564	Calnexin	Canx	0.998	CS pos: 20-21. VEA-HD. Pr: 0.8158	Y	≤0.5	N
Q62376	U1 small nuclear ribonucleoprotein 70 kDa	Snrnp70	< 0.5		N	≤0.5	N
O08759	Ubiquitin-protein ligase E3A	Ube3a	< 0.5		N	≤0.5	N
P59708	Splicing factor 3B subunit 6	Sf3b6	< 0.5		N	≤0.5	N
P63028	Translationally-controlled tumor protein	Tpt1	< 0.5		N	0.527	Y
Q5SW19	Clustered mitochondria protein homolog	Cluh	< 0.5		N	≤0.5	N
P50518	V-type proton ATPase subunit E 1	Atp6v1e1	< 0.5		N	0.59	Y
P55302	Alpha-2-macroglobulin receptor-associated pro	Lrpap1	0.946	CS pos: 33-34. IAG-HG. Pr: 0.6243	Y	0.686	N
P97812	Indian hedgehog protein	Ihh	0.992	CS pos: 27-28. ARG-CG. Pr: 0.8656	Y	0.679	N
Q61830	Macrophage mannose receptor 1	Mrc1	0.980	CS pos: 17-18. VSV-QL. Pr: 0.3103	Y	0.597	N
P49182	Heparin cofactor 2	Serpind1	0.995	CS pos: 23-24. GLA-EQ. Pr: 0.6944	Y	0.535	N
Q8BM72	Heat shock 70 kDa protein 13	Hspa13	0.767	CS pos: 22-23. YLA-QQ. Pr: 0.3443	Y	0.661	N
P97823	Acyl-protein thioesterase 1	Lypla1	< 0.5		N	0.68	Y
Q8CDN6	Thioredoxin-like protein 1	Txnl1	< 0.5		N	≤0.5	N
Q9R1Q7	Proteolipid protein 2	Plp2	< 0.5		N	0.872	Y

Q3UW53	Protein Niban 1	Niban1	< 0.5		N	≤0.5	N
Q60520	Paired amphipathic helix protein Sin3a	Sin3a	< 0.5		N	≤0.5	N
Q9WTX5	S-phase kinase-associated protein 1	Skp1	< 0.5		N	0.649	Y
Q60953	Protein PML	Pml	< 0.5		N	≤0.5	N
Q7TSK7	ADAMTS-like protein 2	Adamtsl2	0.996	CS pos: 29-30. TEA-SD. Pr: 0.4793	Y	≤0.5	N
Q9EQK5	Major vault protein	Mvp	< 0.5		N	≤0.5	N
P12246	Serum amyloid P-component	Apcs	0.995	CS pos: 20-21. AFC-QT. Pr: 0.5415	Y	0.75	N
Q9DBA8	Probable imidazolonepropionase	Amdhd1	< 0.5		N	0.524	Y
P14131	40S ribosomal protein S16	Rps16	< 0.5		N	0.735	Y
Q61092	Laminin subunit gamma-2	Lamc2	0.999	CS pos: 21-22. AQA-TS. Pr: 0.8158	Y	≤0.5	N
Q9DBS1	Transmembrane protein 43	Tmem43	< 0.5		N	0.662	Y
P83940	Elongin-C	Eloc	< 0.5		N	≤0.5	N
P00329	Alcohol dehydrogenase 1	Adh1	< 0.5		N	0.662	Y
Q9JIF0	Protein arginine N-methyltransferase 1	Prmt1	< 0.5		N	≤0.5	N
Q99MK8	Beta-adrenergic receptor kinase 1	Grk2	< 0.5		N	≤0.5	N
Q8K354	Carbonyl reductase [NADPH] 3	Cbr3	< 0.5		N	0.526	Y
Q8CH18	Cell division cycle and apoptosis regulator pro	Ccar1	< 0.5		N	≤0.5	N
Q61419	Cytidine monophosphate-N-acetylneuraminic a	Cmah	< 0.5		N	≤0.5	N
P53996	Cellular nucleic acid-binding protein	Cnbp	< 0.5		N	0.773	Y
Q9CZ04	COP9 signalosome complex subunit 7a	Cops7a	< 0.5		N	≤0.5	N
Q99LI7	Cleavage stimulation factor subunit 3	Cstf3	< 0.5		N	≤0.5	N
P23116	Eukaryotic translation initiation factor 3 subuni	Eif3a	< 0.5		N	≤0.5	N
Q9DBZ5	Eukaryotic translation initiation factor 3 subuni	Eif3k	< 0.5		N	0.611	Y
Q8CFX1	GDH/6PGL endoplasmic bifunctional protein	H6pd	0.983	CS pos: 16-17. LQA-QE. Pr: 0.8834	Y	0.647	N
Q8VD04	GRIP1-associated protein 1	Gripap1	< 0.5		N	≤0.5	N
Q64471	Glutathione S-transferase theta-1	Gstt1	< 0.5		N	0.501	Y
Q9QUP5	Hyaluronan and proteoglycan link protein 1	Hapln1	0.999	CS pos: 15-16. CWA-DH. Pr: 0.8171	Y	0.729	N
P51125	Calpastatin	Cast	< 0.5		N	≤0.5	N
P11881	Inositol 1,4,5-trisphosphate receptor type 1	Itpr1	< 0.5		N	≤0.5	N
P32067	Lupus La protein homolog	Ssb	< 0.5		N	≤0.5	N
Q8CG19	Latent-transforming growth factor beta-binding	Ltbp1	0.970	CS pos: 23-24. AHG-RV. Pr: 0.8375	Y	≤0.5	N
Q3KNA1	Mas-related G-protein coupled receptor memb	Mrgprb2	< 0.5		N	≤0.5	N
Q99J25	rRNA methyltransferase 1, mitochondrial	Mrm1	< 0.5		N	≤0.5	N
Q9QY36	N-alpha-acetyltransferase 10	Naa10	< 0.5		N	0.773	Y
P70255	Nuclear factor 1 C-type	Nfic	< 0.5		N	≤0.5	N
Q91V88	Nephronectin	Npnt	0.996	CS pos: 19-20. VAA-DF. Pr: 0.9251	Y	≤0.5	N
Q8BHG1	Nardilysin	Nrdc	< 0.5		N	≤0.5	N
Q8C7K6	Prenylcysteine oxidase-like	Pcyox1l	0.991	CS pos: 20-21. AAA-GG. Pr: 0.4158	Y	0.648	N
Q61823	Programmed cell death protein 4	Pdcd4	< 0.5		N	≤0.5	N
Q8CG03	cGMP-specific 3',5'-cyclic phosphodiesterase	Pde5a	< 0.5		N	≤0.5	N
P61458	Pterin-4-alpha-carbinolamine dehydratase	Pcbd1	< 0.5		N	≤0.5	N
O08852	Polycystin-1	Pkd1	0.999	CS pos: 23-24. ALA-GD. Pr: 0.8944	Y	≤0.5	N
Q99MI1	ELKS/Rab6-interacting/CAST family member	Erc1	< 0.5		N	≤0.5	N
P12970	60S ribosomal protein L7a	Rpl7a	< 0.5		N	≤0.5	N
O08740	DNA-directed RNA polymerase II subunit RPB	Polr2j	< 0.5		N	≤0.5	N
Q3UH53	Protein sidekick-1	Sdk1	< 0.5		N	≤0.5	N
Q9D939	Sulfotransferase 1C2	Sult1c2	< 0.5		N	≤0.5	N
Q8BL74	General transcription factor 3C polypeptide 2	Gtf3c2	< 0.5		N	≤0.5	N
P04202	Transforming growth factor beta-1 proprotein	Tgfb1	0.961	CS pos: 29-30. AAG-LS. Pr: 0.6223	Y	0.884	N
Q5U405	Transmembrane protease serine 13	Tmprss13	< 0.5		N	0.852	Y
O70404	Vesicle-associated membrane protein 8	Vamp8	< 0.5		N	0.556	Y
Q9CR26	Vacuolar protein sorting-associated protein VT	Vta1	< 0.5		N	≤0.5	N
Q9DBR1	5'-3' exoribonuclease 2	Xrn2	< 0.5		N	≤0.5	N
Q9EPK2	Protein XRP2	Rp2	< 0.5		N	0.53	Y
Q8R121	Protein Z-dependent protease inhibitor	Serpina10	0.881	CS pos: 21-22. VTS-FN. Pr: 0.6406	Y	0.839	N
Q7TNC9	Inositol polyphosphate-5-phosphatase A	Inpp5a	< 0.5		N	≤0.5	N
P63260	Actin, cytoplasmic 2	Actg1	< 0.5		N	0.505	Y
P60710	Actin, cytoplasmic 1	Actb	< 0.5		N	≤0.5	N
P62737	Actin, aortic smooth muscle	Acta2	< 0.5		N	0.505	Y
P01027	Complement C3	C3	0.988	CS pos: 24-25. ALG-IP. Pr: 0.9422	Y	0.536	N
P13020	Gelsolin	Gsn	0.997	CS pos: 25-26. SHA-AT. Pr: 0.9307	Y	0.55	N
P16546	Spectrin alpha chain, non-erythrocytic 1	Sptan1	< 0.5		N	≤0.5	N
P11276	Fibronectin	Fn1	0.996	CS pos: 24-25. VRC-TE. Pr: 0.6827	Y	≤0.5	N
Q9R0G6	Cartilage oligomeric matrix protein	Comp	0.989	CS pos: 19-20. ATG-QG. Pr: 0.4787	Y	≤0.5	N
Q02566	Myosin-6	Myh6	< 0.5		N	≤0.5	N
Q61703	Inter-alpha-trypsin inhibitor heavy chain H2	Itih2	0.998	CS pos: 18-19. AQA-FE. Pr: 0.9801	Y	≤0.5	N

Q64727	Vinculin	Vcl	< 0.5		N	≤0.5	N
P57780	Alpha-actinin-4	Actn4	< 0.5		N	≤0.5	N
P63017	Heat shock cognate 71 kDa protein	Hspa8	< 0.5		N	≤0.5	N
P35441	Thrombospondin-1	Thbs1	0.931	CS pos: 18-19. CGS-NR. Pr: 0.7891	Y	≤0.5	N
Q62261	Spectrin beta chain, non-erythrocytic 1	Sptbn1	< 0.5		N	≤0.5	N
Q9Z1T2	Thrombospondin-4	Thbs4	0.972	CS pos: 26-27. TSA-QA. Pr: 0.6262	Y	≤0.5	N
P50247	Adenosylhomocysteinase	Ahcy	< 0.5		N	0.514	Y
Q08879	Fibulin-1	Fbln1	0.999	CS pos: 29-30. ANA-DI. Pr: 0.7393	Y	0.65	N
P68372	Tubulin beta-4B chain	Tubb4b	< 0.5		N	0.501	Y
Q61704	Inter-alpha-trypsin inhibitor heavy chain H3	Itih3	0.999	CS pos: 21-22. TSG-FP. Pr: 0.8892	Y	0.635	N
P68369	Tubulin alpha-1A chain	Tuba1a	< 0.5		N	≤0.5	N
P19221	Prothrombin	F2	0.900	CS pos: 24-25. VHS-QH. Pr: 0.8517	Y	0.737	N
Q00623	Apolipoprotein A-I	Apoa1	0.997	CS pos: 18-19. SQA-WH. Pr: 0.9763	Y	0.862	N
P14733	Lamin-B1	Lmnb1	< 0.5		N	≤0.5	N
P68368	Tubulin alpha-4A chain	Tuba4a	< 0.5		N	0.55	Y
Q6GQT1	Alpha-2-macroglobulin-P	A2m	0.993	CS pos: 30-31. TNA-SA. Pr: 0.7377	Y	0.52	N
Q9D0F9	Phosphoglucomutase-1	Pgm1	< 0.5		N	≤0.5	N
Q7TMM9	Tubulin beta-2A chain	Tubb2a	< 0.5		N	0.527	Y
O35490	Betaine--homocysteine S-methyltransferase 1	Bhmt	< 0.5		N	≤0.5	N
Q9D6F9	Tubulin beta-4A chain	Tubb4a	< 0.5		N	0.503	Y
P61161	Actin-related protein 2	Actr2	< 0.5		N	≤0.5	N
Q7TPR4	Alpha-actinin-1	Actn1	< 0.5		N	≤0.5	N
Q61696	Heat shock 70 kDa protein 1A	Hspa1a	< 0.5		N	≤0.5	N
P07724	Albumin	Alb	0.993	CS pos: 18-19. AFS-RG. Pr: 0.6691	Y	0.76	N
P63101	14-3-3 protein zeta/delta	Ywhaz	< 0.5		N	≤0.5	N
Q60605	Myosin light polypeptide 6	Myl6	< 0.5		N	≤0.5	N
P11031	Activated RNA polymerase II transcriptional co	Sub1	< 0.5		N	0.679	Y
O88783	Coagulation factor V	F5	0.911	CS pos: 22-23. GWG-SH. Pr: 0.3395	Y	≤0.5	N
P20029	Endoplasmic reticulum chaperone BiP	Hspa5	0.999	CS pos: 19-20. VRA-EE. Pr: 0.9793	Y	0.753	N
Q80X90	Filamin-B	Flnb	< 0.5		N	≤0.5	N
P26043	Radixin	Rdx	< 0.5		N	≤0.5	N
P82198	Transforming growth factor-beta-induced prote	Tgfbi	0.997	CS pos: 23-24. TLA-GP. Pr: 0.8285	Y	0.523	N
Q9JLJ2	4-trimethylaminobutyraldehyde dehydrogenase	Aldh9a1	< 0.5		N	≤0.5	N
P08113	Endoplasmin	Hsp90b1	0.990	CS pos: 21-22. VRA-DD. Pr: 0.9172	Y	≤0.5	N
P16627	Heat shock 70 kDa protein 1-like	Hspa1l	< 0.5		N	≤0.5	N
P12960	Contactin-1	Cntn1	0.992	CS pos: 20-21. CLG-DF. Pr: 0.8881	Y	0.625	N
P10126	Elongation factor 1-alpha 1	Eef1a1	< 0.5		N	≤0.5	N
Q64374	Regucalcin	Rgn	< 0.5		N	0.546	Y
Q61879	Myosin-10	Myh10	< 0.5		N	≤0.5	N
P05064	Fructose-bisphosphate aldolase A	Aldoa	< 0.5		N	≤0.5	N
Q9Z1R3	Apolipoprotein M	Apom	0.733	CS pos: 21-22. SMN-QC. Pr: 0.1527	Y	0.867	N
P18760	Cofilin-1	Cfl1	< 0.5		N	0.629	Y
P31001	Desmin	Des	< 0.5		N	0.684	Y
Q6ZQ38	Cullin-associated NEDD8-dissociated protein	Cand1	< 0.5		N	≤0.5	N
P11087	Collagen alpha-1(I) chain	Col1a1	0.998	CS pos: 22-23. THG-QE. Pr: 0.9642	Y	≤0.5	N
Q61838	Pregnancy zone protein	Pzp	0.997	CS pos: 24-25. ATT-AT. Pr: 0.3698	Y	0.592	N
Q07113	Cation-independent mannose-6-phosphate rec	Igf2r	1.000	CS pos: 35-36. AQA-QA. Pr: 0.6636	Y	≤0.5	N
P62962	Profilin-1	Pfn1	< 0.5		N	0.56	Y
Q9WVK4	EH domain-containing protein 1	Ehd1	< 0.5		N	≤0.5	N
P02468	Laminin subunit gamma-1	Lamc1	0.987	CS pos: 33-34. VRA-AM. Pr: 0.8587	Y	≤0.5	N
Q6PDM2	Serine/arginine-rich splicing factor 1	Srsf1	< 0.5		N	≤0.5	N
P16125	L-lactate dehydrogenase B chain	Ldhb	< 0.5		N	0.567	Y
P43275	Histone H1.1	H1-1	< 0.5		N	≤0.5	N
P01029	Complement C4-B	C4b	0.938	CS pos: 17-18. ASS-LQ. Pr: 0.4093	Y	≤0.5	N
P01942	Hemoglobin subunit alpha	Hba	< 0.5		N	≤0.5	N
Q60847	Collagen alpha-1(XII) chain	Col12a1	0.998	CS pos: 23-24. IEA-EV. Pr: 0.9664	Y	≤0.5	N
E9Q414	Apolipoprotein B-100	Apob	1.000	CS pos: 27-28. VWA-QD. Pr: 0.9941	Y	≤0.5	N
O88342	WD repeat-containing protein 1	Wdr1	< 0.5		N	0.518	Y
Q9CQV8	14-3-3 protein beta/alpha	Ywhab	< 0.5		N	≤0.5	N
Q9EQH3	Vacuolar protein sorting-associated protein 35	Vps35	< 0.5		N	≤0.5	N
Q9ET01	Glycogen phosphorylase, liver form	Pygl	< 0.5		N	≤0.5	N
P0DP26	Calmodulin-1	Calm1	< 0.5		N	0.676	Y
P13541	Myosin-3	Myh3	< 0.5		N	≤0.5	N
Q64487	Receptor-type tyrosine-protein phosphatase de	Ptprd	0.986	CS pos: 20-21. ACA-ET. Pr: 0.5781	Y	≤0.5	N
Q921I1	Serotransferrin	Tf	0.998	CS pos: 19-20. CLA-VP. Pr: 0.9391	Y	0.609	N
P08071	Lactotransferrin	Ltf	0.992	CS pos: 21-22. AKA-TT. Pr: 0.5769	Y	≤0.5	N

P28481	Collagen alpha-1(II) chain	Col2a1	0.999	CS pos: 25-26. CQG-QD. Pr: 0.9269	Y	≤0.5	N
Q60675	Laminin subunit alpha-2	Lama2	0.982	CS pos: 19-20. LEG-SQ. Pr: 0.4559	Y	≤0.5	N
P10630	Eukaryotic initiation factor 4A-II	Eif4a2	< 0.5		N	≤0.5	N
Q91V92	ATP-citrate synthase	Acly	< 0.5		N	0.532	Y
Q8R0Y6	Cytosolic 10-formyltetrahydrofolate dehydroge	Aldh1l1	< 0.5		N	≤0.5	N
P17742	Peptidyl-prolyl cis-trans isomerase A	Ppia	< 0.5		N	≤0.5	N
O88207	Collagen alpha-1(V) chain	Col5a1	0.993	CS pos: 36-37. SRA-AQ. Pr: 0.8862	Y	≤0.5	N
P58771	Tropomyosin alpha-1 chain	Tpm1	< 0.5		N	0.624	Y
P60843	Eukaryotic initiation factor 4A-I	Eif4a1	< 0.5		N	0.631	Y
P61205	ADP-ribosylation factor 3	Arf3	< 0.5		N	≤0.5	N
Q01149	Collagen alpha-2(I) chain	Col1a2	0.830	CS pos: 22-23. ATC-QY. Pr: 0.5899	Y	≤0.5	N
Q9ERK4	Exportin-2	Cse1l	< 0.5		N	≤0.5	N
P24549	Retinal dehydrogenase 1	Aldh1a1	< 0.5		N	0.534	Y
Q9CVB6	Actin-related protein 2/3 complex subunit 2	Arpc2	< 0.5		N	≤0.5	N
Q9WU78	Programmed cell death 6-interacting protein	Pdcd6ip	< 0.5		N	≤0.5	N
Q91Y97	Fructose-bisphosphate aldolase B	Aldob	< 0.5		N	≤0.5	N
Q61753	D-3-phosphoglycerate dehydrogenase	Phgdh	< 0.5		N	0.54	Y
Q61001	Laminin subunit alpha-5	Lama5	0.992	CS pos: 40-41. GEA-RT. Pr: 0.8941	Y	0.524	N
P62631	Elongation factor 1-alpha 2	Eef1a2	< 0.5		N	≤0.5	N
Q68FD5	Clathrin heavy chain 1	Cltc	< 0.5		N	≤0.5	N
Q9QUI0	Transforming protein RhoA	Rhoa	< 0.5		N	0.781	Y
Q9WVA4	Transgelin-2	Tagln2	< 0.5		N	0.79	Y
P02469	Laminin subunit beta-1	Lamb1	0.998	CS pos: 21-22. VCA-QE. Pr: 0.9512	Y	≤0.5	N
O70435	Proteasome subunit alpha type-3	Psma3	< 0.5		N	≤0.5	N
B0V2N1	Receptor-type tyrosine-protein phosphatase S	Ptprs	0.978	CS pos: 29-30. CLA-EE. Pr: 0.9511	Y	≤0.5	N
Q9R1P4	Proteasome subunit alpha type-1	Psma1	< 0.5		N	≤0.5	N
Q99JY3	GTPase IMAP family member 4	Gimap4	< 0.5		N	≤0.5	N
P68510	14-3-3 protein eta	Ywhah	< 0.5		N	≤0.5	N
Q05793	Basement membrane-specific heparan sulfate	Hspg2	0.941	CS pos: 25-26. THG-LR. Pr: 0.3611	Y	≤0.5	N
Q9Z2U1	Proteasome subunit alpha type-5	Psma5	< 0.5		N	≤0.5	N
Q61937	Nucleophosmin	Npm1	< 0.5		N	0.803	Y
Q9CWJ9	Bifunctional purine biosynthesis protein ATIC	Atic	< 0.5		N	0.614	Y
A2A8L5	Receptor-type tyrosine-protein phosphatase F	Ptprf	0.994	CS pos: 29-30. AHG-DS. Pr: 0.8216	Y	≤0.5	N
Q8VDN2	Sodium/potassium-transporting ATPase subur	Atp1a1	< 0.5		N	0.63	Y
P09041	Phosphoglycerate kinase 2	Pgk2	< 0.5		N	≤0.5	N
Q9Z204	Heterogeneous nuclear ribonucleoproteins C1	Hnrnpc	< 0.5		N	≤0.5	N
Q8BK62	Olfactomedin-like protein 3	Olfml3	0.992	CS pos: 21-22. LQG-QQ. Pr: 0.9443	Y	0.644	N
P62827	GTP-binding nuclear protein Ran	Ran	< 0.5		N	0.582	Y
Q60972	Histone-binding protein RBBP4	Rbbp4	< 0.5		N	0.636	Y
Q64449	C-type mannose receptor 2	Mrc2	0.603	CS pos: 30-31. GHP-AD. Pr: 0.2226	Y	0.51	N
A2AQ07	Tubulin beta-1 chain	Tubb1	< 0.5		N	0.546	Y
P21550	Beta-enolase	Eno3	< 0.5		N	≤0.5	N
P62960	Y-box-binding protein 1	Ybx1	< 0.5		N	0.73	Y
Q9ES30	Complement C1q tumor necrosis factor-relatec	C1qtnf3	0.999	CS pos: 22-23. CLC-QD. Pr: 0.9136	Y	0.714	N
Q9Z2U0	Proteasome subunit alpha type-7	Psma7	< 0.5		N	≤0.5	N
Q9R098	Hepatocyte growth factor activator	Hgfac	0.987	CS pos: 34-35. AQP-QA. Pr: 0.6064	Y	0.763	N
P62835	Ras-related protein Rap-1A	Rap1a	< 0.5		N	≤0.5	N
P29391	Ferritin light chain 1	Ftl1	< 0.5		N	≤0.5	N
Q04857	Collagen alpha-1(VI) chain	Col6a1	0.992	CS pos: 19-20. VAT-QD. Pr: 0.6045	Y	≤0.5	N
Q8BH64	EH domain-containing protein 2	Ehd2	< 0.5		N	≤0.5	N
P47754	F-actin-capping protein subunit alpha-2	Capza2	< 0.5		N	≤0.5	N
Q63918	Caveolae-associated protein 2	Cavin2	< 0.5		N	0.654	Y
O88844	Isocitrate dehydrogenase [NADP] cytoplasmic	Idh1	< 0.5		N	0.563	Y
Q2PZL6	Protocadherin Fat 4	Fat4	0.618	CS pos: 42-43. VQA-AE. Pr: 0.4547	Y	≤0.5	N
P45591	Cofilin-2	Cfl2	< 0.5		N	0.683	Y
P26638	Serine--tRNA ligase, cytoplasmic	Sars1	< 0.5		N	≤0.5	N
P28665	Murinoglobulin-1	Mug1	0.948	CS pos: 27-28. LNG-DS. Pr: 0.3292	Y	0.552	N
P20918	Plasminogen	Plg	0.993	CS pos: 19-20. GQG-DS. Pr: 0.9121	Y	≤0.5	N
Q9D0E1	Heterogeneous nuclear ribonucleoprotein M	Hnrnpm	< 0.5		N	≤0.5	N
P62141	Serine/threonine-protein phosphatase PP1-bet	Ppp1cb	< 0.5		N	0.604	Y
P62821	Ras-related protein Rab-1A	Rab1A	< 0.5		N	≤0.5	N
P07742	Ribonucleoside-diphosphate reductase large s	Rrm1	< 0.5		N	≤0.5	N
Q8BPB5	EGF-containing fibulin-like extracellular matrix	Efemp1	0.999	CS pos: 17-18. VKS-QY. Pr: 0.8227	Y	0.747	N
Q9CPW4	Actin-related protein 2/3 complex subunit 5	Arpc5	< 0.5		N	0.684	Y
O09173	Homogentisate 1,2-dioxygenase	Hgd	< 0.5		N	0.543	Y
Q8VHX6	Filamin-C	Flnc	< 0.5		N	≤0.5	N

Q8VCM7	Fibrinogen gamma chain	Fgg	0.999	CS pos: 25-26. GLA-YV. Pr: 0.9148	Y	0.654	N
P14869	60S acidic ribosomal protein P0	Rplp0	< 0.5		N	≤0.5	N
Q9Z1Z0	General vesicular transport factor p115	Uso1	< 0.5		N	≤0.5	N
Q62093	Serine/arginine-rich splicing factor 2	Srsf2	< 0.5		N	0.577	Y
P60766	Cell division control protein 42 homolog	Cdc42	< 0.5		N	0.747	Y
P10493	Nidogen-1	Nid1	0.917	CS pos: 28-29. GGC-LN. Pr: 0.6801	Y	0.676	N
Q3TXS7	26S proteasome non-ATPase regulatory subu	Psmd1	< 0.5		N	≤0.5	N
P16460	Argininosuccinate synthase	Ass1	< 0.5		N	≤0.5	N
Q9QUM9	Proteasome subunit alpha type-6	Psma6	< 0.5		N	≤0.5	N
P02535	Keratin, type I cytoskeletal 10	Krt10	< 0.5		N	≤0.5	N
P19157	Glutathione S-transferase P 1	Gstp1	< 0.5		N	≤0.5	N
O08710	Thyroglobulin	Tg	0.989	CS pos: 20-21. VAA-NI. Pr: 0.7661	Y	0.574	N
Q9CW03	Structural maintenance of chromosomes prote	Smc3	< 0.5		N	≤0.5	N
Q922U2	Keratin, type II cytoskeletal 5	Krt5	< 0.5		N	≤0.5	N
P50446	Keratin, type II cytoskeletal 6A	Krt6a	< 0.5		N	≤0.5	N
P62259	14-3-3 protein epsilon	Ywhae	< 0.5		N	≤0.5	N
Q8VEK3	Heterogeneous nuclear ribonucleoprotein U	Hnrnpu	< 0.5		N	≤0.5	N
Q61598	Rab GDP dissociation inhibitor beta	Gdi2	< 0.5		N	≤0.5	N
Q61176	Arginase-1	Arg1	< 0.5		N	≤0.5	N
P06684	Complement C5	C5	0.998	CS pos: 18-19. TWG-QE. Pr: 0.9879	Y	0.513	N
O35945	Aldehyde dehydrogenase, cytosolic 1	Aldh1a7	< 0.5		N	0.579	Y
P97333	Neuropilin-1	Nrp1	0.997	CS pos: 21-22. AGA-FR. Pr: 0.6902	Y	0.594	N
P53657	Pyruvate kinase PKLR	Pklr	< 0.5		N	≤0.5	N
O35286	Pre-mRNA-splicing factor ATP-dependent RN	Dhx15	< 0.5		N	≤0.5	N
Q8K1B8	Fermitin family homolog 3	Fermt3	< 0.5		N	≤0.5	N
P47856	Glutamine--fructose-6-phosphate aminotransfe	Gfpt1	< 0.5		N	≤0.5	N
P80317	T-complex protein 1 subunit zeta	Cct6a	< 0.5		N	≤0.5	N
Q8R555	Cartilage acidic protein 1	Crtac1	0.884	CS pos: 28-29. TGG-SQ. Pr: 0.3357	Y	0.705	N
Q91ZJ5	UTP--glucose-1-phosphate uridylyltransferase	Ugp2	< 0.5		N	≤0.5	N
Q60864	Stress-induced-phosphoprotein 1	Stip1	< 0.5		N	≤0.5	N
Q99K51	Plastin-3	Pls3	< 0.5		N	≤0.5	N
P49429	4-hydroxyphenylpyruvate dioxygenase	Hpd	< 0.5		N	≤0.5	N
Q9Z2X1	Heterogeneous nuclear ribonucleoprotein F	Hnrnpf	< 0.5		N	≤0.5	N
Q9QYB1	Chloride intracellular channel protein 4	Clic4	< 0.5		N	0.563	Y
Q7TQI3	Ubiquitin thioesterase OTUB1	Otub1	< 0.5		N	≤0.5	N
P97298	Pigment epithelium-derived factor	Serpinf1	0.995	CS pos: 19-20. GSS-QN. Pr: 0.6497	Y	0.806	N
P62137	Serine/threonine-protein phosphatase PP1-alp	Ppp1ca	< 0.5		N	0.572	Y
Q9D8U8	Sorting nexin-5	Snx5	< 0.5		N	≤0.5	N
Q921M7	CYFIP-related Rac1 interactor B	Cyrib	< 0.5		N	≤0.5	N
P98064	Mannan-binding lectin serine protease 1	Masp1	0.998	CS pos: 24-25. VSA-HT. Pr: 0.8855	Y	0.74	N
P24270	Catalase	Cat	< 0.5		N	≤0.5	N
P63087	Serine/threonine-protein phosphatase PP1-ga	Ppp1cc	< 0.5		N	≤0.5	N
P01899	H-2 class I histocompatibility antigen, D-B alp	H2-D1	0.997	CS pos: 24-25. TRA-GP. Pr: 0.9230	Y	0.513	N
P14685	26S proteasome non-ATPase regulatory subu	Psmd3	< 0.5		N	≤0.5	N
Q9R1P0	Proteasome subunit alpha type-4	Psma4	< 0.5		N	≤0.5	N
P49722	Proteasome subunit alpha type-2	Psma2	< 0.5		N	≤0.5	N
Q922B2	Aspartate--tRNA ligase, cytoplasmic	Dars1	< 0.5		N	≤0.5	N
P14206	40S ribosomal protein SA	Rpsa	< 0.5		N	0.62	Y
P58774	Tropomyosin beta chain	Tpm2	< 0.5		N	0.518	Y
Q76MZ3	Serine/threonine-protein phosphatase 2A 65 k	Ppp2r1a	< 0.5		N	0.562	Y
Q9CU62	Structural maintenance of chromosomes prote	Smc1a	< 0.5		N	≤0.5	N
P04186	Complement factor B	Cfb	0.999	CS pos: 22-23. VSA-TP. Pr: 0.9026	Y	≤0.5	N
P97798	Neogenin	Neo1	0.979	CS pos: 41-42. ASG-AA. Pr: 0.6226	Y	≤0.5	N
Q8VIJ6	Splicing factor, proline- and glutamine-rich	Sfpq	< 0.5		N	≤0.5	N
P62192	26S proteasome regulatory subunit 4	Psmc1	< 0.5		N	0.528	Y
Q91VC3	Eukaryotic initiation factor 4A-III	Eif4a3	< 0.5		N	0.634	Y
Q60841	Reelin	Reln	0.991	CS pos: 26-27. ARA-AT. Pr: 0.9068	Y	0.516	N
P21107	Tropomyosin alpha-3 chain	Tpm3	< 0.5		N	0.533	Y
P56395	Cytochrome b5	Cyb5a	< 0.5		N	0.69	Y
P45376	Aldo-keto reductase family 1 member B1	Akr1b1	< 0.5		N	≤0.5	N
P61750	ADP-ribosylation factor 4	Arf4	< 0.5		N	0.593	Y
Q9CQ65	S-methyl-5'-thioadenosine phosphorylase	Mtap	< 0.5		N	≤0.5	N
O55222	Integrin-linked protein kinase	Ilk	< 0.5		N	≤0.5	N
Q99L47	Hsc70-interacting protein	St13	< 0.5		N	0.755	Y
P39749	Flap endonuclease 1	Fen1	< 0.5		N	≤0.5	N
Q08024	Core-binding factor subunit beta	Cbfb	< 0.5		N	≤0.5	N

P21836	Acetylcholinesterase	Ache	0.985	CS pos: 31-32. ARA-EG. Pr: 0.7726	Y	0.701	N
Q8BH61	Coagulation factor XIII A chain	F13a1	< 0.5		N	≤0.5	N
Q91YR9	Prostaglandin reductase 1	Ptgr1	< 0.5		N	≤0.5	N
P09055	Integrin beta-1	Itgb1	0.997	CS pos: 20-21. VFG-QT. Pr: 0.8263	Y	≤0.5	N
P62196	26S proteasome regulatory subunit 8	Psmc5	< 0.5		N	≤0.5	N
Q9R0N0	Galactokinase	Galk1	< 0.5		N	0.751	Y
Q922D8	C-1-tetrahydrofolate synthase, cytoplasmic	Mthfd1	< 0.5		N	≤0.5	N
P63330	Serine/threonine-protein phosphatase 2A catal	Ppp2ca	< 0.5		N	≤0.5	N
Q61470	Leukocyte antigen CD37	Cd37	< 0.5		N	0.851	Y
P15530	B-cell antigen receptor complex-associated pr	Cd79b	0.996	CS pos: 28-29. VPA-MT. Pr: 0.6190	Y	≤0.5	N
O88662	Epithelial membrane protein 2	Emp2	0.547	CS pos: 17-18. STA-LL. Pr: 0.2859	Y	0.595	N
Q9QXY6	EH domain-containing protein 3	Ehd3	< 0.5		N	≤0.5	N
Q9D1A2	Cytosolic non-specific dipeptidase	Cndp2	< 0.5		N	≤0.5	N
Q9CZ44	NSFL1 cofactor p47	Nsfl1c	< 0.5		N	≤0.5	N
P04104	Keratin, type II cytoskeletal 1	Krt1	< 0.5		N	≤0.5	N
Q9Z2D6	Methyl-CpG-binding protein 2	Mecp2	< 0.5		N	0.512	Y
Q62188	Dihydropyrimidinase-related protein 3	Dpysl3	< 0.5		N	≤0.5	N
P63158	High mobility group protein B1	Hmgb1	< 0.5		N	≤0.5	N
Q99020	Heterogeneous nuclear ribonucleoprotein A/B	Hnrnpab	< 0.5		N	0.559	Y
Q9R182	Angiopoietin-related protein 3	Angptl3	0.976	CS pos: 16-17. VIA-SR. Pr: 0.4954	Y	0.515	N
Q9D1G1	Ras-related protein Rab-1B	Rab1b	< 0.5		N	≤0.5	N
P62317	Small nuclear ribonucleoprotein Sm D2	Snrpd2	< 0.5		N	0.914	Y
P02798	Metallothionein-2	Mt2	< 0.5		N	0.833	Y
P08226	Apolipoprotein E	Apoe	1.000	CS pos: 18-19. CLA-EG. Pr: 0.7035	Y	0.938	N
Q61035	Histidine--tRNA ligase, cytoplasmic	Hars1	< 0.5		N	≤0.5	N
Q9D6Z1	Nucleolar protein 56	Nop56	< 0.5		N	≤0.5	N
P46638	Ras-related protein Rab-11B	Rab11b	< 0.5		N	0.539	Y
P02088	Hemoglobin subunit beta-1	Hbb-b1	< 0.5		N	0.508	Y
P62715	Serine/threonine-protein phosphatase 2A catal	Ppp2cb	< 0.5		N	≤0.5	N
O09061	Proteasome subunit beta type-1	Psmb1	< 0.5		N	≤0.5	N
P28063	Proteasome subunit beta type-8	Psmb8	< 0.5		N	0.535	Y
P50516	V-type proton ATPase catalytic subunit A	Atp6v1a	< 0.5		N	≤0.5	N
Q01405	Protein transport protein Sec23A	Sec23a	< 0.5		N	0.606	Y
Q9D2M8	Ubiquitin-conjugating enzyme E2 variant 2	Ube2v2	< 0.5		N	0.878	Y
P60335	Poly(rC)-binding protein 1	Pcbp1	< 0.5		N	0.549	Y
Q501J6	Probable ATP-dependent RNA helicase DDX1	Ddx17	< 0.5		N	≤0.5	N
Q05895	Thrombospondin-3	Thbs3	0.984	CS pos: 22-23. CGS-QD. Pr: 0.5720	Y	≤0.5	N
O88544	COP9 signalosome complex subunit 4	Cops4	< 0.5		N	0.555	Y
Q61982	Neurogenic locus notch homolog protein 3	Notch3	0.994	CS pos: 39-40. GAA-AP. Pr: 0.6939	Y	≤0.5	N
P80315	T-complex protein 1 subunit delta	Cct4	< 0.5		N	≤0.5	N
Q08857	Platelet glycoprotein 4	Cd36	< 0.5		N	0.761	Y
P19253	60S ribosomal protein L13a	Rpl13a	< 0.5		N	0.566	Y
Q05144	Ras-related C3 botulinum toxin substrate 2	Rac2	< 0.5		N	0.693	Y
Q9R1P1	Proteasome subunit beta type-3	Psmb3	< 0.5		N	≤0.5	N
P46460	Vesicle-fusing ATPase	Nsf	< 0.5		N	≤0.5	N
P26231	Catenin alpha-1	Ctnna1	< 0.5		N	≤0.5	N
Q9JHU4	Cytoplasmic dynein 1 heavy chain 1	Dync1h1	< 0.5		N	≤0.5	N
P28271	Cytoplasmic aconitate hydratase	Aco1	< 0.5		N	≤0.5	N
Q61990	Poly(rC)-binding protein 2	Pcbp2	< 0.5		N	0.52	Y
P17710	Hexokinase-1	Hk1	< 0.5		N	≤0.5	N
P10639	Thioredoxin	Txn	< 0.5		N	0.698	Y
Q58A65	C-Jun-amino-terminal kinase-interacting protei	Spag9	< 0.5		N	≤0.5	N
Q8VC12	Urocanate hydratase	Uroc1	< 0.5		N	0.517	Y
Q91V41	Ras-related protein Rab-14	Rab14	< 0.5		N	≤0.5	N
P11152	Lipoprotein lipase	Lpl	0.972	CS pos: 27-28. VAA-AD. Pr: 0.3832	Y	0.741	N
Q99NB9	Splicing factor 3B subunit 1	Sf3b1	< 0.5		N	≤0.5	N
Q9EQU5	Protein SET	Set	< 0.5		N	≤0.5	N
P00493	Hypoxanthine-guanine phosphoribosyltransfera	Hprt1	< 0.5		N	0.766	Y
P42208	Septin-2	Septin2	< 0.5		N	≤0.5	N
Q91YI0	Argininosuccinate lyase	Asl	< 0.5		N	≤0.5	N
Q8CFG9	Complement C1r-B subcomponent	C1rb	0.996	CS pos: 16-17. VEG-SI. Pr: 0.9868	Y	0.716	N
Q9CQI3	Glia maturation factor beta	Gmfb	< 0.5		N	0.568	Y
P09103	Protein disulfide-isomerase	P4hb	0.996	CS pos: 19-20. VGA-DA. Pr: 0.7588	Y	0.731	N
Q6P1B1	Xaa-Pro aminopeptidase 1	Xpnpep1	< 0.5		N	0.594	Y
Q7TNV0	Protein DEK	Dek	< 0.5		N	0.618	Y
P84084	ADP-ribosylation factor 5	Arf5	< 0.5		N	0.65	Y

Q9WV32	Actin-related protein 2/3 complex subunit 1B	Arpc1b	< 0.5		N	≤0.5	N
P28828	Receptor-type tyrosine-protein phosphatase m	Ptprm	0.997	CS pos: 20-21. AAG-ET. Pr: 0.5807	Y	≤0.5	N
Q9WUA3	ATP-dependent 6-phosphofructokinase, platel	Pfkp	< 0.5		N	≤0.5	N
Q60973	Histone-binding protein RBBP7	Rbbp7	< 0.5		N	0.644	Y
Q8BH35	Complement component C8 beta chain	C8b	0.652	CS pos: 31-32. SRG-GK. Pr: 0.4538	Y	0.533	N
O55103	Periaxin	Prx	< 0.5		N	≤0.5	N
P63085	Mitogen-activated protein kinase 1	Mapk1	< 0.5		N	≤0.5	N
O08810	116 kDa U5 small nuclear ribonucleoprotein co	Eftud2	< 0.5		N	≤0.5	N
P29341	Polyadenylate-binding protein 1	Pabpc1	< 0.5		N	≤0.5	N
Q3TTY5	Keratin, type II cytoskeletal 2 epidermal	Krt2	< 0.5		N	≤0.5	N
Q8R001	Microtubule-associated protein RP/EB family r	Mapre2	< 0.5		N	≤0.5	N
P63323	40S ribosomal protein S12	Rps12	< 0.5		N	0.662	Y
P05063	Fructose-bisphosphate aldolase C	Aldoc	< 0.5		N	≤0.5	N
Q61699	Heat shock protein 105 kDa	Hsph1	< 0.5		N	≤0.5	N
P63005	Platelet-activating factor acetylhydrolase IB su	Pafah1b1	< 0.5		N	≤0.5	N
P30416	Peptidyl-prolyl cis-trans isomerase FKBP4	Fkbp4	< 0.5		N	≤0.5	N
P47738	Aldehyde dehydrogenase, mitochondrial	Aldh2	< 0.5		N	0.615	Y
Q8VED5	Keratin, type II cytoskeletal 79	Krt79	< 0.5		N	≤0.5	N
D3YXG0	Hemicentin-1	Hmcn1	0.663	CS pos: 21-22. SLA-GD. Pr: 0.5288	Y	≤0.5	N
Q6IRU2	Tropomyosin alpha-4 chain	Tpm4	< 0.5		N	≤0.5	N
P26350	Prothymosin alpha	Ptma	< 0.5		N	≤0.5	N
P14602	Heat shock protein beta-1	Hspb1	< 0.5		N	0.669	Y
Q6WVG3	BTB/POZ domain-containing protein KCTD12	Kctd12	< 0.5		N	0.781	Y
P42669	Transcriptional activator protein Pur-alpha	Pura	< 0.5		N	≤0.5	N
O88569	Heterogeneous nuclear ribonucleoproteins A2/	Hnrnpa2b1	< 0.5		N	≤0.5	N
Q9D0R2	Threonine--tRNA ligase 1, cytoplasmic	Tars1	< 0.5		N	≤0.5	N
P29699	Alpha-2-HS-glycoprotein	Ahsg	0.999	CS pos: 18-19. CQS-AP. Pr: 0.9517	Y	0.713	N
Q8VCC9	Spondin-1	Spon1	0.953	CS pos: 28-29. ALA-FS. Pr: 0.4289	Y	≤0.5	N
Q9CZU6	Citrate synthase, mitochondrial	Cs	< 0.5		N	0.611	Y
Q6PDN3	Myosin light chain kinase, smooth muscle	Mylk	< 0.5		N	≤0.5	N
Q61555	Fibrillin-2	Fbn2	0.966	CS pos: 28-29. TDG-QP. Pr: 0.7295	Y	≤0.5	N
Q61655	ATP-dependent RNA helicase DDX19A	Ddx19a	< 0.5		N	≤0.5	N
Q99KP6	Pre-mRNA-processing factor 19	Prpf19	< 0.5		N	≤0.5	N
P11103	Poly [ADP-ribose] polymerase 1	Parp1	< 0.5		N	≤0.5	N
P55264	Adenosine kinase	Adk	< 0.5		N	≤0.5	N
Q9JHH6	Carboxypeptidase B2	Cpb2	0.778	CS pos: 21-22. GFA-FQ. Pr: 0.6594	Y	0.707	N
Q11011	Puromycin-sensitive aminopeptidase	Npepps	< 0.5		N	≤0.5	N
Q9JLV5	Cullin-3	Cul3	< 0.5		N	≤0.5	N
P53994	Ras-related protein Rab-2A	Rab2a	< 0.5		N	≤0.5	N
Q9CS84	Neurexin-1	Nrxn1	0.993	CS pos: 25-26. CWA-EL. Pr: 0.6109	Y	≤0.5	N
Q8VED9	Galectin-related protein	Lgalsl	< 0.5		N	≤0.5	N
P12382	ATP-dependent 6-phosphofructokinase, liver t	Pfkl	< 0.5		N	≤0.5	N
Q99KI0	Aconitate hydratase, mitochondrial	Aco2	< 0.5		N	≤0.5	N
Q8R1B4	Eukaryotic translation initiation factor 3 subuni	Eif3c	< 0.5		N	≤0.5	N
Q8CI94	Glycogen phosphorylase, brain form	Pygb	< 0.5		N	≤0.5	N
Q9D662	Protein transport protein Sec23B	Sec23b	< 0.5		N	0.576	Y
P14438	H-2 class II histocompatibility antigen, A-U alp	H2-Aa	< 0.5		N	≤0.5	N
Q8BXK9	Chloride intracellular channel protein 5	Clic5	< 0.5		N	0.585	Y
G3X987	GTPase IMAP family member 9	Gimap9	< 0.5		N	≤0.5	N
O08539	Myc box-dependent-interacting protein 1	Bin1	< 0.5		N	≤0.5	N
Q8BFY9	Transportin-1	Tnpo1	< 0.5		N	≤0.5	N
P61164	Alpha-centractin	Actr1a	< 0.5		N	≤0.5	N
A6X935	Inter alpha-trypsin inhibitor, heavy chain 4	Itih4	0.960	CS pos: 28-29. TTA-EK. Pr: 0.6531	Y	0.611	N
Q8CIH5	1-phosphatidylinositol 4,5-bisphosphate phosp	Plcg2	< 0.5		N	≤0.5	N
P62320	Small nuclear ribonucleoprotein Sm D3	Snrpd3	< 0.5		N	0.763	Y
P61202	COP9 signalosome complex subunit 2	Cops2	< 0.5		N	≤0.5	N
O35344	Importin subunit alpha-4	Kpna3	< 0.5		N	0.597	Y
Q9EPL8	Importin-7	Ipo7	< 0.5		N	0.532	Y
Q7TMK9	Heterogeneous nuclear ribonucleoprotein Q	Syncrip	< 0.5		N	≤0.5	N
Q80YX1	Tenascin	Tnc	0.910	CS pos: 22-23. PEG-GV. Pr: 0.3640	Y	≤0.5	N
Q8R081	Heterogeneous nuclear ribonucleoprotein L	Hnrnpl	< 0.5		N	≤0.5	N
Q8BJY1	26S proteasome non-ATPase regulatory subui	Psmd5	< 0.5		N	0.563	Y
P01901	H-2 class I histocompatibility antigen, K-B alpl	H2-K1	0.994	CS pos: 21-22. TRA-GP. Pr: 0.8947	Y	≤0.5	N
P48758	Carbonyl reductase [NADPH] 1	Cbr1	< 0.5		N	0.598	Y
P54775	26S proteasome regulatory subunit 6B	Psmc4	< 0.5		N	0.519	Y
Q9CWK8	Sorting nexin-2	Snx2	< 0.5		N	0.534	Y

P35278	Ras-related protein Rab-5C	Rab5c	< 0.5		N	0.83	Y
P35235	Tyrosine-protein phosphatase non-receptor typ	Ptpn11	< 0.5		N	≤0.5	N
Q08943	FACT complex subunit SSRP1	Ssrp1	< 0.5		N	≤0.5	N
Q923D2	Flavin reductase (NADPH)	Blvrb	< 0.5		N	0.839	Y
P55258	Ras-related protein Rab-8A	Rab8a	< 0.5		N	0.612	Y
Q9CQC6	Basic leucine zipper and W2 domain-containin	Bzw1	< 0.5		N	≤0.5	N
Q9WU60	Attractin	Atrn	< 0.5		N	≤0.5	N
P09528	Ferritin heavy chain	Fth1	< 0.5		N	0.581	Y
Q3UV17	Keratin, type II cytoskeletal 2 oral	Krt76	< 0.5		N	≤0.5	N
P28474	Alcohol dehydrogenase class-3	Adh5	< 0.5		N	≤0.5	N
O54941	SWI/SNF-related matrix-associated actin-depe	Smarce1	< 0.5		N	0.572	Y
Q810U3	Neurofascin	Nfasc	0.993	CS pos: 24-25. GGA-IE. Pr: 0.8543	Y	≤0.5	N
O35350	Calpain-1 catalytic subunit	Capn1	< 0.5		N	0.566	Y
Q9Z2N8	Actin-like protein 6A	Actl6a	< 0.5		N	≤0.5	N
P18242	Cathepsin D	Ctsd	0.966	CS pos: 20-21. SFA-II. Pr: 0.8573	Y	0.751	N
Q8C650	Septin-10	Septin10	< 0.5		N	≤0.5	N
Q99KJ8	Dynactin subunit 2	Dctn2	< 0.5		N	≤0.5	N
O55029	Coatomer subunit beta'	Copb2	< 0.5		N	≤0.5	N
Q8R550	SH3 domain-containing kinase-binding protein	Sh3kbp1	< 0.5		N	≤0.5	N
Q921M3	Splicing factor 3B subunit 3	Sf3b3	< 0.5		N	0.65	Y
P28843	Dipeptidyl peptidase 4	Dpp4	< 0.5		N	0.687	Y
Q63844	Mitogen-activated protein kinase 3	Mapk3	< 0.5		N	0.532	Y
P09535	Insulin-like growth factor II	Igf2	0.977	CS pos: 24-25. CIA-AY. Pr: 0.5180	Y	0.885	N
P08752	Guanine nucleotide-binding protein G(i) subun	Gnai2	< 0.5		N	0.629	Y
P61028	Ras-related protein Rab-8B	Rab8b	< 0.5		N	0.726	Y
Q9DAK9	14 kDa phosphohistidine phosphatase	Phpt1	< 0.5		N	≤0.5	N
Q9QUR6	Prolyl endopeptidase	Prep	< 0.5		N	0.546	Y
Q8JZK9	Hydroxymethylglutaryl-CoA synthase, cytoplas	Hmgcs1	< 0.5		N	0.562	Y
P51150	Ras-related protein Rab-7a	Rab7a	< 0.5		N	≤0.5	N
P97384	Annexin A11	Anxa11	< 0.5		N	0.726	Y
Q9EQF5	Dihydropyrimidinase	Dpys	< 0.5		N	≤0.5	N
O35841	Apoptosis inhibitor 5	Api5	< 0.5		N	≤0.5	N
Q99MR6	Serrate RNA effector molecule homolog	Srrt	< 0.5		N	≤0.5	N
Q5SUR0	Phosphoribosylformylglycinamidine synthase	Pfas	< 0.5		N	≤0.5	N
P19973	Lymphocyte-specific protein 1	Lsp1	< 0.5		N	0.547	Y
P23198	Chromobox protein homolog 3	Cbx3	< 0.5		N	0.836	Y
P45700	Mannosyl-oligosaccharide 1,2-alpha-mannosic	Man1a1	< 0.5		N	≤0.5	N
P10107	Annexin A1	Anxa1	< 0.5		N	≤0.5	N
Q3UIA2	Rho GTPase-activating protein 17	Arhgap17	< 0.5		N	≤0.5	N
P12023	Amyloid-beta A4 protein	App	0.999	CS pos: 17-18. VRA-LE. Pr: 0.9819	Y	≤0.5	N
P70428	Exostosin-2	Ext2	< 0.5		N	0.692	Y
Q80SW1	S-adenosylhomocysteine hydrolase-like protei	Ahcyl1	< 0.5		N	≤0.5	N
O35643	AP-1 complex subunit beta-1	Ap1b1	< 0.5		N	≤0.5	N
Q9DBG3	AP-2 complex subunit beta	Ap2b1	< 0.5		N	0.677	Y
Q8K4Z5	Splicing factor 3A subunit 1	Sf3a1	< 0.5		N	≤0.5	N
P70695	Fructose-1,6-bisphosphatase isozyme 2	Fbp2	< 0.5		N	0.579	Y
P63038	60 kDa heat shock protein, mitochondrial	Hspd1	< 0.5		N	≤0.5	N
Q03265	ATP synthase subunit alpha, mitochondrial	Atp5f1a	< 0.5		N	0.557	Y
O70194	Eukaryotic translation initiation factor 3 subuni	Eif3d	< 0.5		N	≤0.5	N
Q62000	Mimecan	Ogn	0.976	CS pos: 19-20. TQQ-AP. Pr: 0.7092	Y	0.567	N
Q61554	Fibrillin-1	Fbn1	0.996	CS pos: 24-25. SHG-AD. Pr: 0.7884	Y	≤0.5	N
Q02819	Nucleobindin-1	Nucb1	0.993	CS pos: 25-26. VLA-VP. Pr: 0.6931	Y	≤0.5	N
Q91YM2	Rho GTPase-activating protein 35	Arhgap35	< 0.5		N	≤0.5	N
Q61301	Catenin alpha-2	Ctnna2	< 0.5		N	≤0.5	N
Q812C9	Retina-specific copper amine oxidase	Aoc2	< 0.5		N	0.769	Y
E9PV24	Fibrinogen alpha chain	Fga	0.995	CS pos: 19-20. VWT-TD. Pr: 0.8473	Y	≤0.5	N
Q61702	Inter-alpha-trypsin inhibitor heavy chain H1	Itih1	0.976	CS pos: 26-27. ALG-LA. Pr: 0.5603	Y	≤0.5	N
Q7TSV4	Phosphoglucomutase-2	Pgm2	< 0.5		N	≤0.5	N
P61027	Ras-related protein Rab-10	Rab10	< 0.5		N	0.548	Y
P32233	Developmentally-regulated GTP-binding protei	Drg1	< 0.5		N	≤0.5	N
Q7M6Y3	Phosphatidylinositol-binding clathrin assembly	Picalm	< 0.5		N	≤0.5	N
P63001	Ras-related C3 botulinum toxin substrate 1	Rac1	< 0.5		N	≤0.5	N
Q8BVE3	V-type proton ATPase subunit H	Atp6v1h	< 0.5		N	≤0.5	N
P25785	Metalloproteinase inhibitor 2	Timp2	0.999	CS pos: 26-27. ADA-CS. Pr: 0.9473	Y	0.854	N
Q9WVA3	Mitotic checkpoint protein BUB3	Bub3	< 0.5		N	0.57	Y
O35522	Proteasome subunit beta type-9	Psmb9	< 0.5		N	0.668	Y

P19324	Serpin H1	Serpinh1	0.997	CS pos: 17-18. ALA-AE. Pr: 0.8913	Y	0.841	N
P99026	Proteasome subunit beta type-4	Psmb4	< 0.5		N	0.587	Y
Q61550	Double-strand-break repair protein rad21 homo	Rad21	< 0.5		N	≤0.5	N
Q9JJN5	Carboxypeptidase N catalytic chain	Cpn1	0.599	CS pos: 20-21. VTP-VT. Pr: 0.3645	Y	≤0.5	N
P02463	Collagen alpha-1(IV) chain	Col4a1	1.000	CS pos: 25-26. SRA-AA. Pr: 0.6091	Y	≤0.5	N
Q920B9	FACT complex subunit SPT16	Supt16h	< 0.5		N	≤0.5	N
P12367	cAMP-dependent protein kinase type II-alpha r	Prkar2a	< 0.5		N	0.553	Y
O88456	Calpain small subunit 1	Capns1	< 0.5		N	0.864	Y
P18654	Ribosomal protein S6 kinase alpha-3	Rps6ka3	< 0.5		N	≤0.5	N
Q61207	Prosaposin	Psap	0.997	CS pos: 16-17. ALT-SP. Pr: 0.5495	Y	0.707	N
Q8CF98	Collectin-10	Colec10	0.991	CS pos: 27-28. SLG-LD. Pr: 0.8758	Y	0.797	N
Q9R1P3	Proteasome subunit beta type-2	Psmb2	< 0.5		N	≤0.5	N
O35639	Annexin A3	Anxa3	< 0.5		N	≤0.5	N
Q7TNC4	Putative RNA-binding protein Luc7-like 2	Luc7l2	< 0.5		N	≤0.5	N
Q9R1T2	SUMO-activating enzyme subunit 1	Sae1	< 0.5		N	0.576	Y
P35979	60S ribosomal protein L12	Rpl12	< 0.5		N	0.865	Y
Q6V4S5	Protein sidekick-2	Sdk2	0.993	CS pos: 24-25. AGA-QD. Pr: 0.5845	Y	≤0.5	N
Q9ES46	Beta-parvin	Parvb	< 0.5		N	0.624	Y
Q7TNG5	Echinoderm microtubule-associated protein-lik	Eml2	< 0.5		N	0.59	Y
Q64737	Trifunctional purine biosynthetic protein adeno	Gart	< 0.5		N	0.506	Y
Q8C0C7	Phenylalanine--tRNA ligase alpha subunit	Farsa	< 0.5		N	≤0.5	N
P97351	40S ribosomal protein S3a	Rps3a	< 0.5		N	0.679	Y
O88685	26S proteasome regulatory subunit 6A	Psmc3	< 0.5		N	≤0.5	N
P84089	Enhancer of rudimentary homolog	Erh	< 0.5		N	0.775	Y
Q6NZB0	DnaJ homolog subfamily C member 8	Dnajc8	< 0.5		N	0.668	Y
Q8VHY0	Chondroitin sulfate proteoglycan 4	Cspg4	0.846	CS pos: 29-30. TAP-AS. Pr: 0.2474	Y	0.64	N
O35864	COP9 signalosome complex subunit 5	Cops5	< 0.5		N	≤0.5	N
Q60692	Proteasome subunit beta type-6	Psmb6	< 0.5		N	0.512	Y
Q91ZR1	Ras-related protein Rab-4B	Rab4b	< 0.5		N	0.544	Y
P62334	26S proteasome regulatory subunit 10B	Psmc6	< 0.5		N	0.619	Y
P13864	DNA (cytosine-5)-methyltransferase 1	Dnmt1	< 0.5		N	≤0.5	N
O35516	Neurogenic locus notch homolog protein 2	Notch2	1.000	CS pos: 25-26. AHA-LQ. Pr: 0.9855	Y	0.642	N
Q9DCG6	Phenazine biosynthesis-like domain-containing	Pbld1	< 0.5		N	≤0.5	N
Q03350	Thrombospondin-2	Thbs2	0.996	CS pos: 18-19. ASA-GD. Pr: 0.9135	Y	≤0.5	N
P48774	Glutathione S-transferase Mu 5	Gstm5	< 0.5		N	≤0.5	N
P16301	Phosphatidylcholine-sterol acyltransferase	Lcat	0.757	CS pos: 24-25. ATP-FW. Pr: 0.5716	Y	0.748	N
P61021	Ras-related protein Rab-5B	Rab5b	< 0.5		N	0.676	Y
P99027	60S acidic ribosomal protein P2	Rplp2	0.515	CS pos: 16-17. GNS-SP. Pr: 0.3048	Y	≤0.5	N
P56959	RNA-binding protein FUS	Fus	< 0.5		N	≤0.5	N
P70349	Histidine triad nucleotide-binding protein 1	Hint1	< 0.5		N	0.795	Y
P51437	Cathelicidin antimicrobial peptide	Camp	0.869	CS pos: 27-28. GFS-QT. Pr: 0.6666	Y	≤0.5	N
P56399	Ubiquitin carboxyl-terminal hydrolase 5	Usp5	< 0.5		N	≤0.5	N
O08784	Treacle protein	Tcof1	< 0.5		N	≤0.5	N
Q9CZ30	Obg-like ATPase 1	Ola1	< 0.5		N	≤0.5	N
Q02013	Aquaporin-1	Aqp1	< 0.5		N	≤0.5	N
P49312	Heterogeneous nuclear ribonucleoprotein A1	Hnrnpa1	< 0.5		N	≤0.5	N
Q8QZY1	Eukaryotic translation initiation factor 3 subuni	Eif3l	< 0.5		N	≤0.5	N
Q9CZU3	Exosome RNA helicase MTR4	Mtrex	< 0.5		N	≤0.5	N
Q99LG2	Transportin-2	Tnpo2	< 0.5		N	0.535	Y
Q9D1D6	Collagen triple helix repeat-containing protein	Cthrc1	0.995	CS pos: 32-33. ISA-SE. Pr: 0.8121	Y	0.85	N
Q9DBT5	AMP deaminase 2	Ampd2	< 0.5		N	0.515	Y
Q64511	DNA topoisomerase 2-beta	Top2b	< 0.5		N	≤0.5	N
Q91WP0	Mannan-binding lectin serine protease 2	Masp2	0.976	CS pos: 19-20. LLG-SK. Pr: 0.6336	Y	0.79	N
P61327	Protein mago nashi homolog	Magoh	< 0.5		N	≤0.5	N
P70195	Proteasome subunit beta type-7	Psmb7	< 0.5		N	0.534	Y
P97310	DNA replication licensing factor MCM2	Mcm2	< 0.5		N	≤0.5	N
Q9Z2L7	Cytokine receptor-like factor 3	Crlf3	< 0.5		N	≤0.5	N
P62315	Small nuclear ribonucleoprotein Sm D1	Snrpd1	< 0.5		N	≤0.5	N
P60670	Nuclear protein localization protein 4 homolog	Nploc4	< 0.5		N	0.501	Y
Q9DBC7	cAMP-dependent protein kinase type I-alpha r	Prkar1a	< 0.5		N	≤0.5	N
Q2L4X1	Basic leucine zipper and W2 domain-containin	Bzw2	< 0.5		N	≤0.5	N
Q99JW4	LIM and senescent cell antigen-like-containing	Lims1	< 0.5		N	≤0.5	N
Q6PEB6	MOB-like protein phocein	Mob4	< 0.5		N	0.841	Y
Q769J6	A disintegrin and metalloproteinase with throm	Adamts13	< 0.5		N	0.509	Y
O54865	Guanylate cyclase soluble subunit beta-1	Gucy1b1	< 0.5		N	0.504	Y
P17427	AP-2 complex subunit alpha-2	Ap2a2	< 0.5		N	≤0.5	N

Q8BG32	26S proteasome non-ATPase regulatory subui	Psmd11	< 0.5		N	≤0.5	N
Q9WU84	Copper chaperone for superoxide dismutase	Ccs	< 0.5		N	0.586	Y
P02104	Hemoglobin subunit epsilon-Y2	Hbb-y	< 0.5		N	0.507	Y
O70318	Band 4.1-like protein 2	Epb41l2	< 0.5		N	≤0.5	N
P31938	Dual specificity mitogen-activated protein kina	Map2k1	< 0.5		N	≤0.5	N
Q8VDM4	26S proteasome non-ATPase regulatory subui	Psmd2	< 0.5		N	≤0.5	N
Q62356	Follistatin-related protein 1	Fstl1	1.000	CS pos: 18-19. VHG-EE. Pr: 0.9572	Y	0.507	N
Q02788	Collagen alpha-2(VI) chain	Col6a2	0.948	CS pos: 25-26. LHA-QQ. Pr: 0.6927	Y	≤0.5	N
P34022	Ran-specific GTPase-activating protein	Ranbp1	< 0.5		N	≤0.5	N
Q8BTZ7	Mannose-1-phosphate guanyltransferase beta	Gmppb	< 0.5		N	0.669	Y
Q9R0Q7	Prostaglandin E synthase 3	Ptges3	< 0.5		N	0.706	Y
Q9JKK7	Tropomodulin-2	Tmod2	< 0.5		N	0.522	Y
P08249	Malate dehydrogenase, mitochondrial	Mdh2	< 0.5		N	≤0.5	N
Q8VE37	Regulator of chromosome condensation	Rcc1	< 0.5		N	≤0.5	N
P70389	Insulin-like growth factor-binding protein comp	Igfals	0.992	CS pos: 27-28. LQG-TD. Pr: 0.3173	Y	0.702	N
O70172	Phosphatidylinositol 5-phosphate 4-kinase typ	Pip4k2a	< 0.5		N	≤0.5	N
P27612	Phospholipase A-2-activating protein	Plaa	< 0.5		N	0.555	Y
P15306	Thrombomodulin	Thbd	0.935	CS pos: 21-22. ALA-KL. Pr: 0.5013	Y	0.637	N
Q9WUM3	Coronin-1B	Coro1b	< 0.5		N	0.624	Y
Q9ERL7	Glia maturation factor gamma	Gmfg	< 0.5		N	0.783	Y
Q3U962	Collagen alpha-2(V) chain	Col5a2	0.992	CS pos: 26-27. IKA-QE. Pr: 0.8554	Y	≤0.5	N
Q3THS6	S-adenosylmethionine synthase isoform type-:	Mat2a	< 0.5		N	0.553	Y
O35381	Acidic leucine-rich nuclear phosphoprotein 32	Anp32a	< 0.5		N	≤0.5	N
Q9WVE8	Protein kinase C and casein kinase substrate	Pacsin2	< 0.5		N	0.608	Y
P62307	Small nuclear ribonucleoprotein F	Snrpf	< 0.5		N	≤0.5	N
P10649	Glutathione S-transferase Mu 1	Gstm1	< 0.5		N	0.505	Y
Q9CXW3	Calcyclin-binding protein	Cacybp	< 0.5		N	0.693	Y
P48036	Annexin A5	Anxa5	< 0.5		N	≤0.5	N
Q8VBW6	NEDD8-activating enzyme E1 regulatory subu	Nae1	< 0.5		N	0.569	Y
P08121	Collagen alpha-1(III) chain	Col3a1	0.996	CS pos: 23-24. ILA-QQ. Pr: 0.8746	Y	≤0.5	N
O54774	AP-3 complex subunit delta-1	Ap3d1	< 0.5		N	0.574	Y
P62242	40S ribosomal protein S8	Rps8	< 0.5		N	≤0.5	N
Q9D2V7	Coronin-7	Coro7	< 0.5		N	≤0.5	N
Q61543	Golgi apparatus protein 1	Glg1	0.962	CS pos: 27-28. AGA-QN. Pr: 0.5999	Y	≤0.5	N
Q3TIR3	Synembryn-A	Ric8a	< 0.5		N	≤0.5	N
Q922D4	Serine/threonine-protein phosphatase 6 regula	Ppp6r3	< 0.5		N	≤0.5	N
Q6PHN9	Ras-related protein Rab-35	Rab35	< 0.5		N	≤0.5	N
Q9EST5	Acidic leucine-rich nuclear phosphoprotein 32	Anp32b	< 0.5		N	≤0.5	N
P06745	Glucose-6-phosphate isomerase	Gpi	< 0.5		N	0.533	Y
Q99J45	Nuclear receptor-binding protein	Nrbp1	< 0.5		N	≤0.5	N
Q9QXZ0	Microtubule-actin cross-linking factor 1	Macf1	< 0.5		N	≤0.5	N
P13707	Glycerol-3-phosphate dehydrogenase [NAD(+)	Gpd1	< 0.5		N	0.506	Y
Q6DFW4	Nucleolar protein 58	Nop58	< 0.5		N	≤0.5	N
P13609	Serglycin	Srgn	0.995	CS pos: 25-26. VQG-YP. Pr: 0.9404	Y	≤0.5	N
Q9Z0Y1	Dynactin subunit 3	Dctn3	< 0.5		N	≤0.5	N
P32921	Tryptophan--tRNA ligase, cytoplasmic	Wars1	< 0.5		N	≤0.5	N
P50608	Fibromodulin	Fmod	0.999	CS pos: 18-19. SQG-QY. Pr: 0.9585	Y	0.858	N
Q8BPB0	MOB kinase activator 1B	Mob1b	< 0.5		N	0.647	Y
Q9D8W5	26S proteasome non-ATPase regulatory subui	Psmd12	< 0.5		N	≤0.5	N
Q6Q899	Antiviral innate immune response receptor RI(	Ddx58	< 0.5		N	≤0.5	N
Q64442	Sorbitol dehydrogenase	Sord	< 0.5		N	0.544	Y
Q3B7Z2	Oxysterol-binding protein 1	Osbp	< 0.5		N	≤0.5	N
Q9DB05	Alpha-soluble NSF attachment protein	Napa	< 0.5		N	0.504	Y
Q3ULJ0	Glycerol-3-phosphate dehydrogenase 1-like pr	Gpd1l	< 0.5		N	0.515	Y
P56371	Ras-related protein Rab-4A	Rab4a	< 0.5		N	≤0.5	N
P35279	Ras-related protein Rab-6A	Rab6a	< 0.5		N	0.703	Y
Q8CAA7	Glucose 1,6-bisphosphate synthase	Pgm2l1	< 0.5		N	≤0.5	N
Q9WTQ5	A-kinase anchor protein 12	Akap12	< 0.5		N	≤0.5	N
Q91WG8	Bifunctional UDP-N-acetylglucosamine 2-epim	Gne	< 0.5		N	≤0.5	N
Q8BVI4	Dihydropteridine reductase	Qdpr	< 0.5		N	≤0.5	N
P97429	Annexin A4	Anxa4	< 0.5		N	0.511	Y
Q61220	Protein kinase C-binding protein NELL2	Nell2	0.767	CS pos: 24-25. VWG-LG. Pr: 0.5257	Y	0.592	N
Q61171	Peroxiredoxin-2	Prdx2	< 0.5		N	0.562	Y
P39447	Tight junction protein ZO-1	Tjp1	< 0.5		N	≤0.5	N
O08692	Neutrophilic granule protein	Ngp	0.987	CS pos: 21-22. CEA-LR. Pr: 0.7842	Y	0.881	N
P70694	Estradiol 17 beta-dehydrogenase 5	Akr1c6	< 0.5		N	≤0.5	N

Q62151	Advanced glycosylation end product-specific r	Ager	0.993	CS pos: 23-24. AGG-QN. Pr: 0.6366	Y	≤0.5	N
Q99KD5	Protein unc-45 homolog A	Unc45a	< 0.5		N	≤0.5	N
Q9JMA1	Ubiquitin carboxyl-terminal hydrolase 14	Usp14	< 0.5		N	≤0.5	N
P84096	Rho-related GTP-binding protein RhoG	Rhog	< 0.5		N	0.793	Y
P61082	NEDD8-conjugating enzyme Ubc12	Ube2m	< 0.5		N	0.74	Y
Q9ES52	Phosphatidylinositol 3,4,5-trisphosphate 5-phc	Inpp5d	< 0.5		N	≤0.5	N
P68500	Contactin-5	Cntn5	< 0.5		N	≤0.5	N
Q9Z0E6	Guanylate-binding protein 2	Gbp2	< 0.5		N	≤0.5	N
D3Z7H8	Cartilage intermediate layer protein 2	Cilp2	0.997	CS pos: 20-21. AGA-RD. Pr: 0.8611	Y	≤0.5	N
P97326	Cadherin-6	Cdh6	0.725	CS pos: 21-22. TFS-NP. Pr: 0.5988	Y	≤0.5	N
Q60817	Nascent polypeptide-associated complex subu	Naca	< 0.5		N	≤0.5	N
Q61398	Procollagen C-endopeptidase enhancer 1	Pcolce	0.995	CS pos: 24-25. ARG-QT. Pr: 0.9682	Y	0.554	N
P58281	Dynamin-like 120 kDa protein, mitochondrial	Opa1	< 0.5		N	≤0.5	N
Q99PV0	Pre-mRNA-processing-splicing factor 8	Prpf8	< 0.5		N	≤0.5	N
O09110	Dual specificity mitogen-activated protein kina	Map2k3	< 0.5		N	≤0.5	N
P62270	40S ribosomal protein S18	Rps18	< 0.5		N	≤0.5	N
Q9DBH5	Vesicular integral-membrane protein VIP36	Lman2	0.893	CS pos: 46-47. VAA-DI. Pr: 0.8466	Y	≤0.5	N
Q91X52	L-xylulose reductase	Dcxr	< 0.5		N	0.578	Y
Q9DBE0	Cysteine sulfinic acid decarboxylase	Csad	< 0.5		N	≤0.5	N
O08788	Dynactin subunit 1	Dctn1	< 0.5		N	≤0.5	N
Q9CYL5	Golgi-associated plant pathogenesis-related p	Glipr2	< 0.5		N	0.632	Y
Q3UCV8	Ubiquitin thioesterase otulin	Otulin	< 0.5		N	≤0.5	N
Q91VR5	ATP-dependent RNA helicase DDX1	Ddx1	< 0.5		N	≤0.5	N
Q01705	Neurogenic locus notch homolog protein 1	Notch1	1.000	CS pos: 21-22. ARG-LR. Pr: 0.5956	Y	0.675	N
Q9D4H8	Cullin-2	Cul2	< 0.5		N	≤0.5	N
P61967	AP-1 complex subunit sigma-1A	Ap1s1	< 0.5		N	≤0.5	N
Q91VC4	Plasmalemma vesicle-associated protein	Plvap	< 0.5		N	≤0.5	N
Q8BRK9	Alpha-mannosidase 2x	Man2a2	< 0.5		N	0.595	Y
Q8K1I3	Secreted phosphoprotein 24	Spp2	0.911	CS pos: 23-24. ATG-FP. Pr: 0.8242	Y	0.87	N
P62754	40S ribosomal protein S6	Rps6	< 0.5		N	≤0.5	N
P08228	Superoxide dismutase [Cu-Zn]	Sod1	< 0.5		N	0.76	Y
P24369	Peptidyl-prolyl cis-trans isomerase B	Ppib	0.559	CS pos: 33-34. SVA-ND. Pr: 0.4548	Y	0.864	N
Q9QVP4	Myosin regulatory light chain 2, atrial isoform	Myl7	< 0.5		N	0.805	Y
Q7TMY8	E3 ubiquitin-protein ligase HUWE1	Huwe1	< 0.5		N	≤0.5	N
P34960	Macrophage metalloelastase	Mmp12	0.984	CS pos: 28-29. CGA-AP. Pr: 0.6960	Y	≤0.5	N
O88487	Cytoplasmic dynein 1 intermediate chain 2	Dync1i2	< 0.5		N	≤0.5	N
P08030	Adenine phosphoribosyltransferase	Aprt	< 0.5		N	≤0.5	N
Q99K85	Phosphoserine aminotransferase	Psat1	< 0.5		N	≤0.5	N
P61079	Ubiquitin-conjugating enzyme E2 D3	Ube2d3	< 0.5		N	0.876	Y
P14483	H-2 class II histocompatibility antigen, A beta (	H2-Ab1	0.995	CS pos: 27-28. TEG-GD. Pr: 0.8616	Y	≤0.5	N
P50431	Serine hydroxymethyltransferase, cytosolic	Shmt1	< 0.5		N	≤0.5	N
P51174	Long-chain specific acyl-CoA dehydrogenase,	Acadl	< 0.5		N	0.645	Y
Q9QVP9	Protein-tyrosine kinase 2-beta	Ptk2b	< 0.5		N	≤0.5	N
Q9Z2W0	Aspartyl aminopeptidase	Dnpep	< 0.5		N	≤0.5	N
Q61595	Kinectin	Ktn1	< 0.5		N	≤0.5	N
Q8C0E2	Vacuolar protein sorting-associated protein 26	Vps26b	< 0.5		N	≤0.5	N
Q62422	Osteoclast-stimulating factor 1	Ostf1	< 0.5		N	0.603	Y
O55234	Proteasome subunit beta type-5	Psmb5	< 0.5		N	0.753	Y
P42567	Epidermal growth factor receptor substrate 15	Eps15	< 0.5		N	≤0.5	N
Q9WV85	Nucleoside diphosphate kinase 3	Nme3	0.638	CS pos: 18-19. AYS-GV. Pr: 0.5538	Y	0.525	N
P54823	Probable ATP-dependent RNA helicase DDX6	Ddx6	< 0.5		N	≤0.5	N
P70460	Vasodilator-stimulated phosphoprotein	Vasp	< 0.5		N	0.74	Y
Q6ZWN5	40S ribosomal protein S9	Rps9	< 0.5		N	≤0.5	N
P05132	cAMP-dependent protein kinase catalytic subu	Prkaca	< 0.5		N	≤0.5	N
Q01730	Ras suppressor protein 1	Rsu1	< 0.5		N	0.52	Y
Q03249	Galactose-1-phosphate uridylyltransferase	Galt	< 0.5		N	0.681	Y
P10518	Delta-aminolevulinic acid dehydratase	Alad	< 0.5		N	0.529	Y
P15532	Nucleoside diphosphate kinase A	Nme1	< 0.5		N	≤0.5	N
Q8BHN3	Neutral alpha-glucosidase AB	Ganab	0.507	CS pos: 32-33. TLA-VD. Pr: 0.3745	Y	0.602	N
Q9QZH3	Peptidyl-prolyl cis-trans isomerase E	Ppie	< 0.5		N	≤0.5	N
Q91X83	S-adenosylmethionine synthase isoform type-	Mat1a	< 0.5		N	≤0.5	N
Q80UG5	Septin-9	Septin9	< 0.5		N	0.655	Y
Q07076	Annexin A7	Anxa7	< 0.5		N	0.819	Y
Q6P5F9	Exportin-1	Xpo1	< 0.5		N	0.615	Y
Q80XU3	Nuclear ubiquitous casein and cyclin-depende	Nucks1	< 0.5		N	≤0.5	N
Q9WTR5	Cadherin-13	Cdh13	0.997	CS pos: 22-23. TSA-DD. Pr: 0.8055	Y	0.617	N

P27601	Guanine nucleotide-binding protein subunit alp	Gna13	< 0.5		N	0.579	Y
Q922J3	CAP-Gly domain-containing linker protein 1	Clip1	< 0.5		N	≤0.5	N
A6H5Y3	Methionine synthase	Mtr	< 0.5		N	≤0.5	N
P24527	Leukotriene A-4 hydrolase	Lta4h	< 0.5		N	0.501	Y
Q9D1Q6	Endoplasmic reticulum resident protein 44	Erp44	0.834	CS pos: 29-30. ITA-EI. Pr: 0.6992	Y	≤0.5	N
Q6PDQ2	Chromodomain-helicase-DNA-binding protein	Chd4	< 0.5		N	≤0.5	N
Q9QUR8	Semaphorin-7A	Sema7a	0.755	CS pos: 44-45. ASA-QG. Pr: 0.2851	Y	≤0.5	N
Q6PE01	U5 small nuclear ribonucleoprotein 40 kDa pro	Snrnp40	< 0.5		N	0.64	Y
Q61081	Hsp90 co-chaperone Cdc37	Cdc37	< 0.5		N	≤0.5	N
Q9DAW9	Calponin-3	Cnn3	< 0.5		N	0.663	Y
Q9QZS0	Collagen alpha-3(IV) chain	Col4a3	1.000	CS pos: 28-29. VAS-KG. Pr: 0.8308	Y	≤0.5	N
Q9D1H7	Golgi to ER traffic protein 4 homolog	Get4	< 0.5		N	≤0.5	N
O55226	Chondroadherin	Chad	0.999	CS pos: 20-21. ALA-AC. Pr: 0.8552	Y	0.746	N
Q9JKR6	Hypoxia up-regulated protein 1	Hyou1	0.877	CS pos: 30-31. ALS-DT. Pr: 0.4695	Y	≤0.5	N
O88587	Catechol O-methyltransferase	Comt	< 0.5		N	0.941	Y
Q9JM99	Proteoglycan 4	Prg4	1.000	CS pos: 24-25. VSS-QD. Pr: 0.9404	Y	≤0.5	N
O35295	Transcriptional activator protein Pur-beta	Purb	< 0.5		N	≤0.5	N
Q3THK3	General transcription factor IIF subunit 1	Gtf2f1	< 0.5		N	0.55	Y
Q8CEF9	Transmembrane protein 132C	Tmem132c	0.941	CS pos: 31-32. TES-RG. Pr: 0.7396	Y	≤0.5	N
Q8VC30	Triokinase/FMN cyclase	Tkfc	< 0.5		N	≤0.5	N
P05202	Aspartate aminotransferase, mitochondrial	Got2	< 0.5		N	0.51	Y
B2RXS4	Plexin-B2	Plxnb2	0.895	CS pos: 19-20. GLS-LR. Pr: 0.5039	Y	0.601	N
P35293	Ras-related protein Rab-18	Rab18	< 0.5		N	≤0.5	N
Q9D154	Leukocyte elastase inhibitor A	Serpinb1a	< 0.5		N	≤0.5	N
Q61490	CD166 antigen	Alcam	0.861	CS pos: 27-28. GLG-WY. Pr: 0.7601	Y	≤0.5	N
P63328	Serine/threonine-protein phosphatase 2B catal	Ppp3ca	< 0.5		N	≤0.5	N
Q6DIB5	Multiple epidermal growth factor-like domains	Megf10	0.993	CS pos: 25-26. ASS-LN. Pr: 0.5242	Y	≤0.5	N
Q61792	LIM and SH3 domain protein 1	Lasp1	< 0.5		N	0.722	Y
Q8R059	UDP-glucose 4-epimerase	Gale	< 0.5		N	≤0.5	N
Q9WVJ2	26S proteasome non-ATPase regulatory subu	Psmd13	< 0.5		N	0.528	Y
Q9Z0N1	Eukaryotic translation initiation factor 2 subuni	Eif2s3x	< 0.5		N	≤0.5	N
P60882	Multiple epidermal growth factor-like domains	Megf8	0.981	CS pos: 27-28. VLA-GD. Pr: 0.7908	Y	≤0.5	N
Q9JM76	Actin-related protein 2/3 complex subunit 3	Arpc3	< 0.5		N	0.689	Y
Q62443	Neuronal pentraxin-1	Nptx1	0.990	CS pos: 22-23. SGA-QD. Pr: 0.8299	Y	0.672	N
Q61508	Extracellular matrix protein 1	Ecm1	0.968	CS pos: 19-20. ASA-AS. Pr: 0.3623	Y	0.587	N
Q9Z2D0	Myotubularin-related protein 9	Mtmr9	< 0.5		N	0.555	Y
Q8JZQ5	Amiloride-sensitive amine oxidase [copper-cor	Aoc1	0.984	CS pos: 22-23. ASA-VT. Pr: 0.7265	Y	0.659	N
Q9CPY7	Cytosol aminopeptidase	Lap3	< 0.5		N	≤0.5	N
Q60737	Casein kinase II subunit alpha	Csnk2a1	< 0.5		N	0.854	Y
O08583	THO complex subunit 4	Alyref	< 0.5		N	≤0.5	N
Q9CQD1	Ras-related protein Rab-5A	Rab5a	< 0.5		N	0.911	Y
Q9QUJ7	Long-chain-fatty-acid--CoA ligase 4	Acsl4	< 0.5		N	≤0.5	N
Q8C052	Microtubule-associated protein 1S	Map1s	< 0.5		N	≤0.5	N
P62281	40S ribosomal protein S11	Rps11	< 0.5		N	0.754	Y
Q99M28	RNA-binding protein with serine-rich domain 1	Rnps1	< 0.5		N	≤0.5	N
Q9DCW4	Electron transfer flavoprotein subunit beta	Etfb	< 0.5		N	≤0.5	N
P35585	AP-1 complex subunit mu-1	Ap1m1	< 0.5		N	0.657	Y
Q9JM14	5'(3')-deoxyribonucleotidase, cytosolic type	Nt5c	< 0.5		N	0.774	Y
P26262	Plasma kallikrein	Klkb1	0.640	CS pos: 23-24. CMT-QL. Pr: 0.1708	Y	0.535	N
Q60631	Growth factor receptor-bound protein 2	Grb2	< 0.5		N	≤0.5	N
O54988	STE20-like serine/threonine-protein kinase	Slk	< 0.5		N	≤0.5	N
P47955	60S acidic ribosomal protein P1	Rplp1	< 0.5		N	≤0.5	N
P19096	Fatty acid synthase	Fasn	< 0.5		N	≤0.5	N
P51885	Lumican	Lum	0.998	CS pos: 18-19. VSG-QY. Pr: 0.9668	Y	0.556	N
Q9CRA5	Golgi phosphoprotein 3	Golph3	< 0.5		N	0.801	Y
Q8BYA0	Tubulin-specific chaperone D	Tbcd	< 0.5		N	0.525	Y
P97927	Laminin subunit alpha-4	Lama4	0.987	CS pos: 24-25. SNA-AS. Pr: 0.6212	Y	≤0.5	N
P17095	High mobility group protein HMG-I/HMG-Y	Hmga1	< 0.5		N	≤0.5	N
Q6ZWX6	Eukaryotic translation initiation factor 2 subuni	Eif2s1	< 0.5		N	≤0.5	N
Q8CFI7	DNA-directed RNA polymerase II subunit RPB	Polr2b	< 0.5		N	≤0.5	N
P25911	Tyrosine-protein kinase Lyn	Lyn	< 0.5		N	≤0.5	N
Q3V3R4	Integrin alpha-1	Itga1	< 0.5		N	0.639	Y
Q61087	Laminin subunit beta-3	Lamb3	0.999	CS pos: 17-18. LCA-QQ. Pr: 0.2685	Y	≤0.5	N
Q9JIY5	Serine protease HTRA2, mitochondrial	Htra2	< 0.5		N	0.885	Y
Q8K2Q9	Shootin-1	Shtn1	< 0.5		N	0.572	Y
P61255	60S ribosomal protein L26	Rpl26	< 0.5		N	≤0.5	N

Q8K1M6	Dynamin-1-like protein	Dnm1l	< 0.5		N	≤0.5	N
O54754	Aldehyde oxidase 1	Aox1	< 0.5		N	≤0.5	N
P42225	Signal transducer and activator of transcription	Stat1	< 0.5		N	≤0.5	N
Q8R4Y4	Stabilin-1	Stab1	0.989	CS pos: 20-21. SDS-SF. Pr: 0.4268	Y	0.537	N
Q8C196	Carbamoyl-phosphate synthase [ammonia], m	Cps1	< 0.5		N	≤0.5	N
Q60854	Serpin B6	Serpinb6	< 0.5		N	≤0.5	N
Q99LX0	Parkinson disease protein 7 homolog	Park7	< 0.5		N	0.537	Y
P47911	60S ribosomal protein L6	Rpl6	< 0.5		N	≤0.5	N
Q8R0W0	Epiplakin	Eppk1	< 0.5		N	≤0.5	N
Q9Z0X1	Apoptosis-inducing factor 1, mitochondrial	Aifm1	< 0.5		N	≤0.5	N
Q64739	Collagen alpha-2(XI) chain	Col11a2	0.989	CS pos: 27-28. GWA-GA. Pr: 0.7647	Y	≤0.5	N
Q99LF4	RNA-splicing ligase RtcB homolog	Rtcb	< 0.5		N	0.528	Y
Q6IFX2	Keratin, type I cytoskeletal 42	Krt42	< 0.5		N	0.559	Y
Q9QZB7	Actin-related protein 10	Actr10	< 0.5		N	≤0.5	N
Q64433	10 kDa heat shock protein, mitochondrial	Hspe1	< 0.5		N	0.584	Y
P26928	Hepatocyte growth factor-like protein	Mst1	0.995	CS pos: 18-19. ALG-QR. Pr: 0.8524	Y	0.557	N
A2ASQ1	Agrin	Agrn	< 0.5		N	≤0.5	N
P28798	Progranulin	Grn	1.000	CS pos: 17-18. VAG-TQ. Pr: 0.7872	Y	0.651	N
P70122	Ribosome maturation protein SBDS	Sbds	< 0.5		N	0.606	Y
Q8CHR6	Dihydropyrimidine dehydrogenase [NADP(+)]	Dpyd	< 0.5		N	≤0.5	N
Q8R1F1	Protein Niban 2	Niban2	< 0.5		N	≤0.5	N
Q9CPR4	60S ribosomal protein L17	Rpl17	< 0.5		N	0.551	Y
P97352	Protein S100-A13	S100a13	< 0.5		N	0.773	Y
O70133	ATP-dependent RNA helicase A	Dhx9	< 0.5		N	≤0.5	N
Q8BWT1	3-ketoacyl-CoA thiolase, mitochondrial	Acaa2	< 0.5		N	0.502	Y
Q8BZM1	Glomulin	Glmn	< 0.5		N	≤0.5	N
Q99PL5	Ribosome-binding protein 1	Rrbp1	< 0.5		N	≤0.5	N
Q9CXY6	Interleukin enhancer-binding factor 2	Ilf2	< 0.5		N	≤0.5	N
P00375	Dihydrofolate reductase	Dhfr	< 0.5		N	≤0.5	N
Q8CIE6	Coatomer subunit alpha	Copa	< 0.5		N	≤0.5	N
P97807	Fumarate hydratase, mitochondrial	Fh	< 0.5		N	0.51	Y
Q6P9R2	Serine/threonine-protein kinase OSR1	Oxsr1	< 0.5		N	≤0.5	N
P22892	AP-1 complex subunit gamma-1	Ap1g1	< 0.5		N	≤0.5	N
P53026	60S ribosomal protein L10a	Rpl10a	< 0.5		N	0.618	Y
Q3U0S6	Ras-interacting protein 1	Rasip1	< 0.5		N	≤0.5	N
P35329	B-cell receptor CD22	Cd22	0.999	CS pos: 18-19. ASA-QY. Pr: 0.7892	Y	≤0.5	N
P63242	Eukaryotic translation initiation factor 5A-1	Eif5a	< 0.5		N	≤0.5	N
P61290	Proteasome activator complex subunit 3	Psme3	< 0.5		N	≤0.5	N
Q9EQS9	Immunoglobulin superfamily DCC subclass m	Igdcc4	0.978	CS pos: 22-23. ARG-EL. Pr: 0.8994	Y	0.713	N
Q6A0D4	Raftlin	Rftn1	< 0.5		N	≤0.5	N
P62748	Hippocalcin-like protein 1	Hpcal1	< 0.5		N	0.711	Y
Q8BWY3	Eukaryotic peptide chain release factor subuni	Etf1	< 0.5		N	≤0.5	N
P26645	Myristoylated alanine-rich C-kinase substrate	Marcks	< 0.5		N	≤0.5	N
Q8R4V5	Oncoprotein-induced transcript 3 protein	Oit3	0.972	CS pos: 19-20. VSP-AV. Pr: 0.5712	Y	0.686	N
P47791	Glutathione reductase, mitochondrial	Gsr	< 0.5		N	0.513	Y
Q9CQJ6	Density-regulated protein	Denr	< 0.5		N	0.802	Y
Q8BPU7	Engulfment and cell motility protein 1	Elmo1	< 0.5		N	≤0.5	N
Q6ZPJ3	(E3-independent) E2 ubiquitin-conjugating enz	Ube2o	< 0.5		N	≤0.5	N
Q9EQG9	Ceramide transfer protein	Cert1	< 0.5		N	≤0.5	N
O88998	Noelin	Olfm1	< 0.5		N	≤0.5	N
O88322	Nidogen-2	Nid2	0.993	CS pos: 30-31. VGA-LR. Pr: 0.9503	Y	0.712	N
Q569Z6	Thyroid hormone receptor-associated protein 3	Thrap3	< 0.5		N	≤0.5	N
Q8BWY9	Protein CIP2A	Cip2a	< 0.5		N	0.526	Y
Q8VH51	RNA-binding protein 39	Rbm39	< 0.5		N	≤0.5	N
Q8CJG0	Protein argonaute-2	Ago2	< 0.5		N	≤0.5	N
Q9JHK4	Geranylgeranyl transferase type-2 subunit alpl	Rabggta	< 0.5		N	≤0.5	N
Q91VI7	Ribonuclease inhibitor	Rnh1	< 0.5		N	0.526	Y
O08808	Protein diaphanous homolog 1	Diaph1	< 0.5		N	≤0.5	N
Q62348	Translin	Tsn	< 0.5		N	≤0.5	N
Q9QXX0	Protein jagged-1	Jag1	0.994	CS pos: 30-31. VCG-AS. Pr: 0.4631	Y	≤0.5	N
Q9ES89	Exostosin-like 2	Extl2	< 0.5		N	0.702	Y
Q8VCT3	Aminopeptidase B	Rnpep	< 0.5		N	0.575	Y
Q8BME9	Cerebellin-4	Cbln4	0.995	CS pos: 24-25. VWA-QN. Pr: 0.9718	Y	0.916	N
Q61553	Fascin	Fscn1	< 0.5		N	≤0.5	N
P62702	40S ribosomal protein S4, X isoform	Rps4x	< 0.5		N	0.605	Y
P70236	Dual specificity mitogen-activated protein kina	Map2k6	< 0.5		N	≤0.5	N

Q8R5A3	Amyloid beta A4 precursor protein-binding fam	Apbb1ip	< 0.5		N	≤0.5	N
Q9DC11	Plexin domain-containing protein 2	Plxdc2	0.594	CS pos: 30-31. AHG-EP. Pr: 0.4435	Y	≤0.5	N
Q01768	Nucleoside diphosphate kinase B	Nme2	< 0.5		N	≤0.5	N
P55284	Cadherin-5	Cdh5	0.979	CS pos: 24-25. AMA-GP. Pr: 0.8268	Y	≤0.5	N
P84104	Serine/arginine-rich splicing factor 3	Srsf3	< 0.5		N	≤0.5	N
Q91WC0	Actin-histidine N-methyltransferase	Setd3	< 0.5		N	≤0.5	N
Q9R269	Periplakin	Ppl	< 0.5		N	≤0.5	N
Q9DBJ1	Phosphoglycerate mutase 1	Pgam1	< 0.5		N	≤0.5	N
Q9CZJ2	Heat shock 70 kDa protein 12B	Hspa12b	< 0.5		N	≤0.5	N
O35737	Heterogeneous nuclear ribonucleoprotein H	Hnrnph1	< 0.5		N	≤0.5	N
P49817	Caveolin-1	Cav1	< 0.5		N	≤0.5	N
Q8BUK6	Protein Hook homolog 3	Hook3	< 0.5		N	≤0.5	N
Q60716	Prolyl 4-hydroxylase subunit alpha-2	P4ha2	0.958	CS pos: 23-24. VQA-EF. Pr: 0.8550	Y	0.793	N
Q9JIF7	Coatomer subunit beta	Copb1	< 0.5		N	≤0.5	N
P16110	Galectin-3	Lgals3	< 0.5		N	0.765	Y
P39054	Dynamin-2	Dnm2	< 0.5		N	≤0.5	N
O35955	Proteasome subunit beta type-10	Psmb10	< 0.5		N	0.504	Y
A3KGB4	TBC1 domain family member 8B	Tbc1d8b	< 0.5		N	≤0.5	N
P70336	Rho-associated protein kinase 2	Rock2	< 0.5		N	≤0.5	N
Q9DCN1	NAD-capped RNA hydrolase NUDT12	Nudt12	< 0.5		N	0.511	Y
Q8VD75	Huntingtin-interacting protein 1	Hip1	< 0.5		N	≤0.5	N
Q61107	Guanylate-binding protein 4	Gbp4	< 0.5		N	≤0.5	N
O08807	Peroxiredoxin-4	Prdx4	0.815	CS pos: 37-38. TES-LQ. Pr: 0.5588	Y	0.552	N
P35282	Ras-related protein Rab-21	Rab21	< 0.5		N	≤0.5	N
Q8R1K1	Ubiquitin-associated domain-containing protei	Ubac2	< 0.5		N	0.889	Y
Q91WG2	Rab GTPase-binding effector protein 2	Rabep2	< 0.5		N	≤0.5	N
Q3U0V1	Far upstream element-binding protein 2	Khsrp	< 0.5		N	≤0.5	N
Q8R326	Paraspeckle component 1	Pspc1	< 0.5		N	≤0.5	N
P33434	72 kDa type IV collagenase	Mmp2	0.980	CS pos: 29-30. AIA-AP. Pr: 0.9176	Y	0.589	N
Q6P9S7	Polypeptide N-acetylgalactosaminyltransferase	Galnt10	< 0.5		N	0.592	Y
Q99KN9	Clathrin interactor 1	Clint1	0.846	CS pos: 19-20. AFS-LC. Pr: 0.2428	Y	≤0.5	N
Q9R0P5	Destrin	Dstn	< 0.5		N	0.6	Y
Q8C5L6	Inositol polyphosphate 5-phosphatase K	Inpp5k	< 0.5		N	0.725	Y
P08556	GTPase NRas	Nras	< 0.5		N	0.68	Y
Q8BFQ4	WD repeat-containing protein 82	Wdr82	< 0.5		N	≤0.5	N
Q9D0T1	NHP2-like protein 1	Snu13	< 0.5		N	≤0.5	N
Q8R3C0	Mini-chromosome maintenance complex-bindi	Mcmbp	< 0.5		N	≤0.5	N
Q3TDD9	Protein phosphatase 1 regulatory subunit 21	Ppp1r21	< 0.5		N	≤0.5	N
Q8K2H4	Arf-GAP with coiled-coil, ANK repeat and PH c	Acap1	< 0.5		N	≤0.5	N
P70398	Probable ubiquitin carboxyl-terminal hydrolase	Usp9x	< 0.5		N	≤0.5	N
P54923	[Protein ADP-ribosylarginine] hydrolase	Adprh	< 0.5		N	0.565	Y
Q9EQQ9	Protein O-GlcNAcase	Oga	< 0.5		N	≤0.5	N
Q68FH4	N-acetylgalactosamine kinase	Galk2	< 0.5		N	0.552	Y
Q9QWL7	Keratin, type I cytoskeletal 17	Krt17	< 0.5		N	0.692	Y
Q8C115	Pleckstrin homology domain-containing family	Plekhh2	< 0.5		N	≤0.5	N
Q91YR7	Pre-mRNA-processing factor 6	Prpf6	< 0.5		N	≤0.5	N
Q3V4B5	COMM domain-containing protein 6	Commd6	< 0.5		N	0.711	Y
Q9JII6	Aldo-keto reductase family 1 member A1	Akr1a1	< 0.5		N	≤0.5	N
P40237	CD82 antigen	Cd82	< 0.5		N	0.832	Y
P63168	Dynein light chain 1, cytoplasmic	Dynll1	< 0.5		N	≤0.5	N
Q8K4K6	Pantothenate kinase 1	Pank1	< 0.5		N	≤0.5	N
Q99LD4	COP9 signalosome complex subunit 1	Gps1	< 0.5		N	≤0.5	N
Q9Z0P5	Twinfilin-2	Twf2	< 0.5		N	≤0.5	N
P28659	CUGBP Elav-like family member 1	Celf1	< 0.5		N	≤0.5	N
Q8BYC6	Serine/threonine-protein kinase TAO3	Taok3	< 0.5		N	≤0.5	N
P97464	Exostosin-1	Ext1	< 0.5		N	≤0.5	N
Q61103	Zinc finger protein ubi-d4	Dpf2	< 0.5		N	0.636	Y
Q91YE3	Egl nine homolog 1	Egln1	< 0.5		N	≤0.5	N
Q01320	DNA topoisomerase 2-alpha	Top2a	< 0.5		N	≤0.5	N
P62869	Elongin-B	Elob	< 0.5		N	0.578	Y
Q9D0W5	Peptidyl-prolyl cis-trans isomerase-like 1	Ppil1	< 0.5		N	≤0.5	N
Q8BTU1	Cilia- and flagella-associated protein 20	Cfap20	< 0.5		N	0.876	Y
Q01339	Beta-2-glycoprotein 1	Apoh	0.996	CS pos: 19-20. AIA-GR. Pr: 0.8475	Y	0.597	N
Q60963	Platelet-activating factor acetylhydrolase	Pla2g7	0.960	CS pos: 21-22. VHP-FH. Pr: 0.9001	Y	0.816	N
Q923G2	DNA-directed RNA polymerases I, II, and III su	Polr2h	< 0.5		N	≤0.5	N
P61971	Nuclear transport factor 2	Nutf2	< 0.5		N	0.625	Y

Q60715	Prolyl 4-hydroxylase subunit alpha-1	P4ha1	0.995	CS pos: 17-18. SLA-HP. Pr: 0.8912	Y	0.823	N
Q9DBD0	Inhibitor of carbonic anhydrase	Ica	0.998	CS pos: 19-20. CLA-LP. Pr: 0.8683	Y	0.626	N
Q9WVJ9	EGF-containing fibulin-like extracellular matrix	Efemp2	0.998	CS pos: 27-28. ASP-QD. Pr: 0.4407	Y	0.823	N
P62858	40S ribosomal protein S28	Rps28	< 0.5		N	0.522	Y
Q9Z2T6	Keratin, type II cuticular Hb5	Krt85	< 0.5		N	≤0.5	N
Q9R1C7	Pre-mRNA-processing factor 40 homolog A	Prpf40a	< 0.5		N	≤0.5	N
Q8BY87	Ubiquitin carboxyl-terminal hydrolase 47	Usp47	< 0.5		N	≤0.5	N
O55142	60S ribosomal protein L35a	Rpl35a	< 0.5		N	0.818	Y
P27046	Alpha-mannosidase 2	Man2a1	< 0.5		N	0.597	Y
Q8K2I3	Dimethylaniline monooxygenase [N-oxide-forn	Fmo2	< 0.5		N	0.582	Y
P01872	Immunoglobulin heavy constant mu	Ighm	< 0.5		N	0.604	Y
Q63810	Calcineurin subunit B type 1	Ppp3r1	< 0.5		N	0.548	Y
P62305	Small nuclear ribonucleoprotein E	Snrpe	< 0.5		N	0.802	Y
Q8VDM6	Heterogeneous nuclear ribonucleoprotein U-lik	Hnrnpul1	< 0.5		N	≤0.5	N
Q04592	Proprotein convertase subtilisin/kexin type 5	Pcsk5	0.585	CS pos: 34-35. CRT-RV. Pr: 0.4048	Y	≤0.5	N
Q91W10	Metal cation symporter ZIP8	Slc39a8	0.940	CS pos: 19-20. SLG-HP. Pr: 0.5385	Y	0.838	N
O88947	Coagulation factor X	F10	0.936	CS pos: 23-24. GKG-VF. Pr: 0.7951	Y	0.586	N
Q9WTL7	Acyl-protein thioesterase 2	Lypla2	< 0.5		N	0.659	Y
Q61161	Mitogen-activated protein kinase kinase kinase	Map4k2	< 0.5		N	≤0.5	N
O89026	Roundabout homolog 1	Robo1	0.718	CS pos: 19-20. CSG-SR. Pr: 0.3835	Y	≤0.5	N
Q8R574	Phosphoribosyl pyrophosphate synthase-asso	Prpsap2	< 0.5		N	≤0.5	N
Q6IQX7	Chondroitin sulfate synthase 2	Chpf	< 0.5		N	0.534	Y
P60122	RuvB-like 1	Ruvbl1	< 0.5		N	≤0.5	N
Q61545	RNA-binding protein EWS	Ewsr1	< 0.5		N	≤0.5	N
Q3UVV9	von Willebrand factor A domain-containing pro	Vwa3a	< 0.5		N	≤0.5	N
Q9QUN9	Dickkopf-related protein 3	Dkk3	0.992	CS pos: 21-22. TAP-AP. Pr: 0.4171	Y	0.858	N
Q91WT9	Cystathionine beta-synthase	Cbs	< 0.5		N	0.514	Y
A2AQ19	RNA polymerase-associated protein RTF1 hor	Rtf1	< 0.5		N	≤0.5	N
P21279	Guanine nucleotide-binding protein G(q) subur	Gnaq	< 0.5		N	≤0.5	N
Q99P58	Ras-related protein Rab-27B	Rab27b	< 0.5		N	0.591	Y
Q9EPK7	Exportin-7	Xpo7	< 0.5		N	0.565	Y
Q78PG9	Coiled-coil domain-containing protein 25	Ccdc25	< 0.5		N	0.766	Y
P25799	Nuclear factor NF-kappa-B p105 subunit	Nfkb1	< 0.5		N	≤0.5	N
Q02248	Catenin beta-1	Ctnnb1	< 0.5		N	0.614	Y
O35593	26S proteasome non-ATPase regulatory subu	Psmd14	< 0.5		N	0.777	Y
P54265	Myotonin-protein kinase	Dmpk	< 0.5		N	≤0.5	N
P86048	60S ribosomal protein L10-like	Rpl10l	< 0.5		N	0.662	Y
Q8C854	Myelin expression factor 2	Myef2	< 0.5		N	≤0.5	N
Q8BGC0	HIV Tat-specific factor 1 homolog	Htatsf1	< 0.5		N	≤0.5	N
A2AVA0	Sushi, von Willebrand factor type A, EGF and	Svep1	0.883	CS pos: 17-18. VSG-WT. Pr: 0.3298	Y	≤0.5	N
P97372	Proteasome activator complex subunit 2	Psme2	< 0.5		N	≤0.5	N
Q810U4	Neuronal cell adhesion molecule	Nrcam	0.979	CS pos: 29-30. ISA-LD. Pr: 0.9217	Y	0.571	N
Q9CYG7	Mitochondrial import receptor subunit TOM34	Tomm34	< 0.5		N	≤0.5	N
Q9DCL9	Multifunctional protein ADE2	Paics	< 0.5		N	≤0.5	N
P19437	B-lymphocyte antigen CD20	Ms4a1	< 0.5		N	≤0.5	N
Q9CZD3	Glycine--tRNA ligase	Gars1	< 0.5		N	≤0.5	N
Q8BND5	Sulfhydryl oxidase 1	Qsox1	0.992	CS pos: 32-33. AYA-AR. Pr: 0.9520	Y	0.57	N
Q9D1J3	SAP domain-containing ribonucleoprotein	Sarnp	< 0.5		N	≤0.5	N
P63037	DnaJ homolog subfamily A member 1	Dnaja1	< 0.5		N	≤0.5	N
P70232	Neural cell adhesion molecule L1-like protein	Chl1	0.988	CS pos: 25-26. SAA-EI. Pr: 0.6634	Y	≤0.5	N
Q8BY89	Choline transporter-like protein 2	Slc44a2	< 0.5		N	≤0.5	N
Q5XJY5	Coatomer subunit delta	Arcn1	< 0.5		N	≤0.5	N
Q8BWG8	Beta-arrestin-1	Arrb1	< 0.5		N	≤0.5	N
P97311	DNA replication licensing factor MCM6	Mcm6	< 0.5		N	≤0.5	N
Q61768	Kinesin-1 heavy chain	Kif5b	< 0.5		N	≤0.5	N
Q08481	Platelet endothelial cell adhesion molecule	Pecam1	0.991	CS pos: 17-18. LQA-EE. Pr: 0.8985	Y	≤0.5	N
P62918	60S ribosomal protein L8	Rpl8	< 0.5		N	0.636	Y
Q9R0E1	Multifunctional procollagen lysine hydroxylase	Plod3	0.996	CS pos: 27-28. TSA-SD. Pr: 0.4549	Y	0.63	N
Q9WUU7	Cathepsin Z	Ctsz	0.992	CS pos: 25-26. ARA-RL. Pr: 0.8809	Y	0.782	N
Q9DCT8	Cysteine-rich protein 2	Crip2	< 0.5		N	0.807	Y
Q8BRK8	5'-AMP-activated protein kinase catalytic subu	Prkaa2	< 0.5		N	≤0.5	N
Q8R2Y2	Cell surface glycoprotein MUC18	Mcam	0.981	CS pos: 23-24. AAG-VP. Pr: 0.6739	Y	≤0.5	N
Q8CB27	Ubiquitin thioesterase OTU1	Yod1	< 0.5		N	≤0.5	N
Q9JMH9	Unconventional myosin-XVIIIa	Myo18a	< 0.5		N	≤0.5	N
Q8K2V1	Serine/threonine-protein phosphatase 4 regula	Ppp4r1	< 0.5		N	≤0.5	N
P36993	Protein phosphatase 1B	Ppm1b	< 0.5		N	≤0.5	N

Q8VCT4	Carboxylesterase 1D	Ces1d	0.997	CS pos: 18-19. AWG-YP. Pr: 0.9180	Y	0.757	N
Q8CIN4	Serine/threonine-protein kinase PAK 2	Pak2	< 0.5		N	≤0.5	N
Q9ESX5	H/ACA ribonucleoprotein complex subunit DK(	Dkc1	< 0.5		N	≤0.5	N
Q9QXK3	Coatomer subunit gamma-2	Copg2	< 0.5		N	≤0.5	N
Q91WE2	PSME3-interacting protein	Psme3ip1	< 0.5		N	≤0.5	N
Q8VDF3	Death-associated protein kinase 2	Dapk2	< 0.5		N	0.531	Y
Q9Z1G3	V-type proton ATPase subunit C 1	Atp6v1c1	< 0.5		N	≤0.5	N
P36536	GTP-binding protein SAR1a	Sar1a	< 0.5		N	0.553	Y
Q9CSU0	Regulation of nuclear pre-mRNA domain-conta	Rprd1b	< 0.5		N	≤0.5	N
Q60790	Ras GTPase-activating protein 3	Rasa3	< 0.5		N	≤0.5	N
P31725	Protein S100-A9	S100a9	< 0.5		N	≤0.5	N
Q9D0F3	Protein ERGIC-53	Lman1	0.850	CS pos: 32-33. SDG-TG. Pr: 0.3827	Y	≤0.5	N
Q8QZY9	Splicing factor 3B subunit 4	Sf3b4	< 0.5		N	≤0.5	N
Q8BKC5	Importin-5	Ipo5	< 0.5		N	0.627	Y
P48193	Protein 4.1	Epb41	< 0.5		N	≤0.5	N
Q9QZ88	Vacuolar protein sorting-associated protein 29	Vps29	< 0.5		N	≤0.5	N
P46467	Vacuolar protein sorting-associated protein 4B	Vps4b	< 0.5		N	0.622	Y
P54116	Stomatin	Stom	< 0.5		N	≤0.5	N
P26516	26S proteasome non-ATPase regulatory subu	Psmd7	< 0.5		N	≤0.5	N
Q9Z1R2	Large proline-rich protein BAG6	Bag6	< 0.5		N	≤0.5	N
P97315	Cysteine and glycine-rich protein 1	Csrp1	< 0.5		N	≤0.5	N
P56528	ADP-ribosyl cyclase/cyclic ADP-ribose hydrola	Cd38	< 0.5		N	0.642	Y
Q3TCH7	Cullin-4A	Cul4a	< 0.5		N	≤0.5	N
Q9CZX8	40S ribosomal protein S19	Rps19	< 0.5		N	0.826	Y
Q6PD03	Serine/threonine-protein phosphatase 2A 56 k	Ppp2r5a	< 0.5		N	≤0.5	N
P59325	Eukaryotic translation initiation factor 5	Eif5	< 0.5		N	≤0.5	N
Q9JHJ0	Tropomodulin-3	Tmod3	< 0.5		N	0.617	Y
P49443	Protein phosphatase 1A	Ppm1a	< 0.5		N	0.517	Y
P16330	2',3'-cyclic-nucleotide 3'-phosphodiesterase	Cnp	< 0.5		N	0.526	Y
Q9WTZ2	Membrane-bound transcription factor site-1 pr	Mbtps1	0.982	CS pos: 22-23. HLG-DR. Pr: 0.3456	Y	≤0.5	N
O35685	Nuclear migration protein nudC	Nudc	< 0.5		N	≤0.5	N
Q9CPX6	Ubiquitin-like-conjugating enzyme ATG3	Atg3	< 0.5		N	0.528	Y
Q9Z1X4	Interleukin enhancer-binding factor 3	Ilf3	< 0.5		N	≤0.5	N
O89020	Afamin	Afm	0.996	CS pos: 21-22. SLA-LP. Pr: 0.7858	Y	0.626	N
Q99KB8	Hydroxyacylglutathione hydrolase, mitochondr	Hagh	< 0.5		N	0.797	Y
P27048	Small nuclear ribonucleoprotein-associated pr	Snrpb	< 0.5		N	≤0.5	N
Q9CXW4	60S ribosomal protein L11	Rpl11	< 0.5		N	≤0.5	N
Q60865	Caprin-1	Caprin1	< 0.5		N	≤0.5	N
Q3UDE2	Tubulin--tyrosine ligase-like protein 12	Ttll12	< 0.5		N	0.599	Y
Q61686	Chromobox protein homolog 5	Cbx5	< 0.5		N	0.745	Y
O08529	Calpain-2 catalytic subunit	Capn2	< 0.5		N	≤0.5	N
P30115	Glutathione S-transferase A3	Gsta3	< 0.5		N	≤0.5	N
Q9CQC9	GTP-binding protein SAR1b	Sar1b	< 0.5		N	0.527	Y
Q9EPX2	Papilin	Papln	0.999	CS pos: 20-21. SWA-RN. Pr: 0.9801	Y	≤0.5	N
Q8BU30	Isoleucine--tRNA ligase, cytoplasmic	Iars1	< 0.5		N	≤0.5	N
Q8BP47	Asparagine--tRNA ligase, cytoplasmic	NARS1	< 0.5		N	≤0.5	N
P52431	DNA polymerase delta catalytic subunit	Pold1	< 0.5		N	≤0.5	N
Q3TC72	Fumarylacetoacetate hydrolase domain-contai	Fahd2	< 0.5		N	0.723	Y
Q9CY64	Biliverdin reductase A	Blvra	< 0.5		N	0.623	Y
Q640N1	Adipocyte enhancer-binding protein 1	Aebp1	0.974	CS pos: 25-26. GNP-QT. Pr: 0.4557	Y	≤0.5	N
Q8VDG3	Poly(A)-specific ribonuclease PARN	Parn	< 0.5		N	≤0.5	N
Q80UM3	N-alpha-acetyltransferase 15, NatA auxiliary s	Naa15	< 0.5		N	≤0.5	N
Q9CQT1	Methylthioribose-1-phosphate isomerase	Mri1	< 0.5		N	≤0.5	N
Q9DB34	Charged multivesicular body protein 2a	Chmp2a	< 0.5		N	≤0.5	N
O88712	C-terminal-binding protein 1	Ctbp1	< 0.5		N	0.517	Y
Q8QZT1	Acetyl-CoA acetyltransferase, mitochondrial	Acat1	< 0.5		N	0.53	Y
Q8CIZ8	von Willebrand factor	Vwf	0.993	CS pos: 22-23. TLC-TE. Pr: 0.8736	Y	≤0.5	N
P13595	Neural cell adhesion molecule 1	Ncam1	0.947	CS pos: 19-20. AVS-LQ. Pr: 0.5985	Y	0.513	N
Q9DC48	Pre-mRNA-processing factor 17	Cdc40	< 0.5		N	≤0.5	N
P97290	Plasma protease C1 inhibitor	Serping1	0.995	CS pos: 22-23. AFS-DP. Pr: 0.8669	Y	0.725	N
Q9D1R9	60S ribosomal protein L34	Rpl34	< 0.5		N	0.829	Y
Q91YR1	Twinfilin-1	Twf1	< 0.5		N	0.679	Y
P70335	Rho-associated protein kinase 1	Rock1	< 0.5		N	≤0.5	N
P28661	Septin-4	Septin4	< 0.5		N	≤0.5	N
P35980	60S ribosomal protein L18	Rpl18	< 0.5		N	≤0.5	N
Q9DBF1	Alpha-aminoadipic semialdehyde dehydrogena	Aldh7a1	< 0.5		N	0.723	Y

P55288	Cadherin-11	Cdh11	0.854	CS pos: 24-25. AFA-LE. Pr: 0.5103	Y	≤0.5	N
P62331	ADP-ribosylation factor 6	Arf6	< 0.5		N	0.702	Y
Q05722	Collagen alpha-1(IX) chain	Col9a1	0.935	CS pos: 23-24. VSA-TL. Pr: 0.8065	Y	≤0.5	N
Q9CQM5	Thioredoxin domain-containing protein 17	Txndc17	< 0.5		N	≤0.5	N
P62996	Transformer-2 protein homolog beta	Tra2b	< 0.5		N	≤0.5	N
Q9JII5	DAZ-associated protein 1	Dazap1	< 0.5		N	≤0.5	N
P52840	Sulfotransferase 1A1	Sult1a1	< 0.5		N	0.548	Y
Q9Z2M7	Phosphomannomutase 2	Pmm2	< 0.5		N	0.63	Y
Q8VDP4	Cell cycle and apoptosis regulator protein 2	Ccar2	< 0.5		N	≤0.5	N
P70288	Histone deacetylase 2	Hdac2	< 0.5		N	≤0.5	N
Q8BHL4	Retinoic acid-induced protein 3	Gprc5a	< 0.5		N	≤0.5	N
Q8CC86	Nicotinate phosphoribosyltransferase	Naprt	< 0.5		N	≤0.5	N
Q8CI51	PDZ and LIM domain protein 5	Pdlim5	< 0.5		N	0.522	Y
Q922S4	cGMP-dependent 3',5'-cyclic phosphodiesteras	Pde2a	< 0.5		N	≤0.5	N
Q9DB16	Calcium-binding protein 39-like	Cab39l	< 0.5		N	≤0.5	N
Q8BLY2	Threonine--tRNA ligase 2, cytoplasmic	Tars3	< 0.5		N	≤0.5	N
Q61210	Rho guanine nucleotide exchange factor 1	Arhgef1	< 0.5		N	≤0.5	N
Q71KU9	Fibrinogen-like protein 1	Fgl1	0.991	CS pos: 22-23. GWA-LE. Pr: 0.9532	Y	0.6	N
P54071	Isocitrate dehydrogenase [NADP], mitochondri	Idh2	< 0.5		N	0.52	Y
Q9D358	Low molecular weight phosphotyrosine protein	Acp1	< 0.5		N	0.843	Y
P15327	Bisphosphoglycerate mutase	Bpgm	< 0.5		N	≤0.5	N
Q8K0T7	Protein unc-13 homolog C	Unc13c	< 0.5		N	≤0.5	N
Q9R1W5	Calcitonin gene-related peptide type 1 recepto	Calcrl	0.998	CS pos: 22-23. ISA-ES. Pr: 0.6440	Y	≤0.5	N
O89001	Carboxypeptidase D	Cpd	0.991	CS pos: 37-38. AQA-AH. Pr: 0.8803	Y	≤0.5	N
P47199	Quinone oxidoreductase	Cryz	< 0.5		N	≤0.5	N
Q9D0L8	mRNA cap guanine-N7 methyltransferase	Rnmt	< 0.5		N	≤0.5	N
Q9JHL1	Na(+)/H(+) exchange regulatory cofactor NHE-	Slc9a3r2	< 0.5		N	0.596	Y
O54818	Tumor protein D53	Tpd52l1	< 0.5		N	0.863	Y
Q9D020	Cytosolic 5'-nucleotidase 3A	Nt5c3a	< 0.5		N	0.554	Y
Q62283	Tetraspanin-7	Tspan7	< 0.5		N	0.779	Y
P42232	Signal transducer and activator of transcription	Stat5b	< 0.5		N	0.548	Y
Q8R5H1	Ubiquitin carboxyl-terminal hydrolase 15	Usp15	< 0.5		N	≤0.5	N
P20060	Beta-hexosaminidase subunit beta	Hexb	0.797	CS pos: 28-29. VAP-AR. Pr: 0.2749	Y	0.652	N
Q810B6	Rabankyrin-5	Ankfy1	< 0.5		N	≤0.5	N
Q03311	Cholinesterase	Bche	0.961	CS pos: 29-30. SHT-EE. Pr: 0.5016	Y	0.795	N
Q99KK7	Dipeptidyl peptidase 3	Dpp3	< 0.5		N	≤0.5	N
Q9JKY5	Huntingtin-interacting protein 1-related protein	Hip1r	< 0.5		N	≤0.5	N
Q99KQ4	Nicotinamide phosphoribosyltransferase	Nampt	< 0.5		N	≤0.5	N
O54931	A-kinase anchor protein 2	Akap2	< 0.5		N	≤0.5	N
P06801	NADP-dependent malic enzyme	Me1	< 0.5		N	≤0.5	N
Q99PT1	Rho GDP-dissociation inhibitor 1	Arhgdia	< 0.5		N	≤0.5	N
Q3UL36	Arginine and glutamate-rich protein 1	Arglu1	< 0.5		N	≤0.5	N
P70697	Uroporphyrinogen decarboxylase	Urod	< 0.5		N	0.547	Y
Q02257	Junction plakoglobin	Jup	< 0.5		N	≤0.5	N
Q64514	Tripeptidyl-peptidase 2	Tpp2	< 0.5		N	≤0.5	N
P28650	Adenylosuccinate synthetase isozyme 1	Adss1	< 0.5		N	≤0.5	N
Q61282	Aggrecan core protein	Acan	0.938	CS pos: 19-20. VIS-EE. Pr: 0.3714	Y	0.549	N
Q8VCA8	Secernin-2	Scrn2	< 0.5		N	≤0.5	N
Q9JIX8	Apoptotic chromatin condensation inducer in tl	Acin1	< 0.5		N	≤0.5	N
P38647	Stress-70 protein, mitochondrial	Hspa9	< 0.5		N	≤0.5	N
P31324	cAMP-dependent protein kinase type II-beta re	Prkar2b	< 0.5		N	0.59	Y
P51830	Adenylate cyclase type 9	Adcy9	< 0.5		N	0.63	Y
Q8C7R4	Ubiquitin-like modifier-activating enzyme 6	Uba6	< 0.5		N	0.516	Y
Q9DCH4	Eukaryotic translation initiation factor 3 subuni	Eif3f	< 0.5		N	0.846	Y
P28660	Nck-associated protein 1	Nckap1	< 0.5		N	0.542	Y
Q9DB29	Isoamyl acetate-hydrolyzing esterase 1 homol	Iah1	< 0.5		N	≤0.5	N
Q64373	Bcl-2-like protein 1	Bcl2l1	< 0.5		N	0.72	Y
Q61205	Platelet-activating factor acetylhydrolase IB su	Pafah1b3	< 0.5		N	≤0.5	N
Q8C1A5	Thimet oligopeptidase	Thop1	< 0.5		N	≤0.5	N
Q8BK63	Casein kinase I isoform alpha	Csnk1a1	< 0.5		N	≤0.5	N
Q3UKJ7	WD40 repeat-containing protein SMU1	Smu1	< 0.5		N	≤0.5	N
P61222	ATP-binding cassette sub-family E member 1	Abce1	< 0.5		N	≤0.5	N
O35900	U6 snRNA-associated Sm-like protein LSm2	Lsm2	< 0.5		N	0.597	Y
P61358	60S ribosomal protein L27	Rpl27	< 0.5		N	0.612	Y
P62900	60S ribosomal protein L31	Rpl31	< 0.5		N	≤0.5	N
O08599	Syntaxin-binding protein 1	Stxbp1	< 0.5		N	≤0.5	N

P62264	40S ribosomal protein S14	Rps14	＜0.5		N	0.691	Y
P67871	Casein kinase II subunit beta	Csnk2b	＜0.5		N	0.744	Y
P62301	40S ribosomal protein S13	Rps13	＜0.5		N	0.816	Y
Q9D0M5	Dynein light chain 2, cytoplasmic	Dynll2	＜0.5		N	≤0.5	N
Q9Z0H3	SWI/SNF-related matrix-associated actin-depe	Smarcb1	＜0.5		N	≤0.5	N
P60867	40S ribosomal protein S20	Rps20	＜0.5		N	0.769	Y
P62751	60S ribosomal protein L23a	Rpl23a	＜0.5		N	≤0.5	N
P62743	AP-2 complex subunit sigma	Ap2s1	＜0.5		N	≤0.5	N
Q6ZWU9	40S ribosomal protein S27	Rps27	＜0.5		N	≤0.5	N
P62889	60S ribosomal protein L30	Rpl30	＜0.5		N	≤0.5	N
P62878	E3 ubiquitin-protein ligase RBX1	Rbx1	＜0.5		N	0.576	Y
Q9CZM2	60S ribosomal protein L15	Rpl15	＜0.5		N	≤0.5	N
P62911	60S ribosomal protein L32	Rpl32	＜0.5		N	≤0.5	N
P62313	U6 snRNA-associated Sm-like protein LSm6	Lsm6	＜0.5		N	0.522	Y
P10852	4F2 cell-surface antigen heavy chain	Slc3a2	＜0.5		N	0.602	Y
O54950	5'-AMP-activated protein kinase subunit gamm	Prkag1	＜0.5		N	≤0.5	N
Q3THG9	Alanyl-tRNA editing protein Aarsd1	Aarsd1	＜0.5		N	≤0.5	N
Q6P542	ATP-binding cassette sub-family F member 1	Abcf1	＜0.5		N	≤0.5	N
Q8CBW3	Abl interactor 1	Abi1	＜0.5		N	≤0.5	N
Q9CQR4	Acyl-coenzyme A thioesterase 13	Acot13	＜0.5		N	≤0.5	N
Q60994	Adiponectin	Adipoq	0.999	CS pos: 17-18. SHA-ED. Pr: 0.9769	Y	0.879	N
Q9JKV1	Proteasomal ubiquitin receptor ADRM1	Adrm1	＜0.5		N	≤0.5	N
Q8BK64	Activator of 90 kDa heat shock protein ATPase	Ahsa1	＜0.5		N	≤0.5	N
D3YVF0	A-kinase anchor protein 5	Akap5	＜0.5		N	≤0.5	N
Q07456	Protein AMBP	Ambp	0.998	CS pos: 19-20. SRA-DP. Pr: 0.7501	Y	0.767	N
P97467	Peptidyl-glycine alpha-amidating monooxygen	Pam	0.988	CS pos: 24-25. CLA-FR. Pr: 0.9215	Y	≤0.5	N
O08739	AMP deaminase 3	Ampd3	＜0.5		N	≤0.5	N
P16406	Glutamyl aminopeptidase	Enpep	＜0.5		N	0.572	Y
B2RXR6	Serine/threonine-protein phosphatase 6 regula	Ankrd44	＜0.5		N	0.598	Y
Q9CZ52	Anthrax toxin receptor 1	Antxr1	0.957	CS pos: 27-28. GHG-GR. Pr: 0.6315	Y	≤0.5	N
Q9JME5	AP-3 complex subunit beta-2	Ap3b2	＜0.5		N	≤0.5	N
Q8R146	Acylamino-acid-releasing enzyme	Apeh	＜0.5		N	0.587	Y
Q9R0Q6	Actin-related protein 2/3 complex subunit 1A	Arpc1a	＜0.5		N	≤0.5	N
Q9D8S3	ADP-ribosylation factor GTPase-activating pro	Arfgap3	＜0.5		N	≤0.5	N
Q8CG72	ADP-ribose glycohydrolase ARH3	Adprs	＜0.5		N	0.556	Y
A2BH40	AT-rich interactive domain-containing protein 1	Arid1a	＜0.5		N	≤0.5	N
Q8CG76	Aflatoxin B1 aldehyde reductase member 2	Akr7a2	＜0.5		N	0.842	Y
Q8VEH3	ADP-ribosylation factor-like protein 8A	Arl8a	＜0.5		N	0.73	Y
P50428	Arylsulfatase A	Arsa	0.979	CS pos: 17-18. STA-SP. Pr: 0.7776	Y	0.876	N
Q3TYD4	Arylsulfatase G	Arsg	0.588	CS pos: 16-17. AFS-GF. Pr: 0.3011	Y	0.852	N
Q91WU5	Arsenite methyltransferase	As3mt	＜0.5		N	≤0.5	N
Q9DCQ2	Putative L-aspartate dehydrogenase	Aspdh	＜0.5		N	0.665	Y
Q99MQ4	Asporin	Aspn	0.971	CS pos: 15-16. CSA-KP. Pr: 0.7720	Y	0.572	N
Q80T21	ADAMTS-like protein 4	Adamtsl4	0.991	CS pos: 24-25. CQD-QE. Pr: 0.6407	Y	0.514	N
Q91YH5	Atlastin-3	Atl3	＜0.5		N	0.526	Y
Q9R001	A disintegrin and metalloproteinase with throm	Adamts5	0.994	CS pos: 23-24. SLA-AD. Pr: 0.6264	Y	≤0.5	N
P59017	Bcl-2-like protein 13	Bcl2l13	＜0.5		N	≤0.5	N
Q09200	Beta-1,4 N-acetylgalactosaminyltransferase 1	B4galnt1	0.728	CS pos: 25-26. LYS-ST. Pr: 0.1599	Y	0.71	N
P15535	Beta-1,4-galactosyltransferase 1	B4galt1	＜0.5		N	0.754	Y
P18572	Basigin	Bsg	0.988	CS pos: 21-22. ACA-AA. Pr: 0.3622	Y	≤0.5	N
Q07813	Apoptosis regulator BAX	Bax	＜0.5		N	0.741	Y
Q9R069	Basal cell adhesion molecule	Bcam	0.993	CS pos: 25-26. AQA-EL. Pr: 0.8178	Y	≤0.5	N
P98063	Bone morphogenetic protein 1	Bmp1	0.995	CS pos: 29-30. GRP-LD. Pr: 0.5822	Y	≤0.5	N
P97452	Ribosome biogenesis protein BOP1	Bop1	＜0.5		N	≤0.5	N
P46737	Lys-63-specific deubiquitinase BRCC36	Brcc3	＜0.5		N	≤0.5	N
Q8BRN9	Coiled-coil and C2 domain-containing protein 1	Cc2d1b	＜0.5		N	≤0.5	N
Q8BHG2	CXXC motif containing zinc binding protein	Czib	＜0.5		N	≤0.5	N
Q64444	Carbonic anhydrase 4	Ca4	0.993	CS pos: 15-16. VAP-ST. Pr: 0.3626	Y	0.53	N
O35887	Calumenin	Calu	0.981	CS pos: 19-20. ALS-KP. Pr: 0.6438	Y	0.756	N
P10605	Cathepsin B	Ctsb	0.999	CS pos: 17-18. TSA-HD. Pr: 0.9303	Y	0.852	N
P49935	Pro-cathepsin H	Ctsh	0.991	CS pos: 21-22. ATA-EL. Pr: 0.8040	Y	0.55	N
O70370	Cathepsin S	Ctss	0.830	CS pos: 22-23. SVA-ME. Pr: 0.4690	Y	0.528	N
Q8BVC4	Coiled-coil domain-containing protein 68	Ccdc68	＜0.5		N	0.606	Y
D3YZP9	Coiled-coil domain-containing protein 6	Ccdc6	＜0.5		N	≤0.5	N
Q64314	Hematopoietic progenitor cell antigen CD34	Cd34	0.837	CS pos: 34-35. TSA-TT. Pr: 0.6404	Y	≤0.5	N
P40240	CD9 antigen	Cd9	＜0.5		N	≤0.5	N

Q91WE6	Threonylcarbamoyladenosine tRNA methylthic	Cdkal1	< 0.5		N	≤0.5	N
P31809	Carcinoembryonic antigen-related cell adhesic	Ceacam1	0.924	CS pos: 34-35. TTA-EV. Pr: 0.4442	Y	≤0.5	N
Q9WUD1	STIP1 homology and U box-containing protein	Stub1	< 0.5		N	0.876	Y
Q9CR86	Calcium-regulated heat stable protein 1	Carhsp1	< 0.5		N	0.683	Y
A2AGT5	Cytoskeleton-associated protein 5	Ckap5	< 0.5		N	≤0.5	N
Q3U5Q7	UMP-CMP kinase 2, mitochondrial	Cmpk2	< 0.5		N	≤0.5	N
Q9DBC3	Cap-specific mRNA (nucleoside-2'-O-)-methylt	Cmtr1	< 0.5		N	≤0.5	N
Q9JJZ8	Cyclic nucleotide-gated cation channel alpha-:	Cnga3	< 0.5		N	0.567	Y
Q08093	Calponin-2	Cnn2	< 0.5		N	≤0.5	N
Q69Z26	Contactin-4	Cntn4	0.990	CS pos: 18-19. CLA-DD. Pr: 0.9552	Y	≤0.5	N
Q9DBL7	Bifunctional coenzyme A synthase	Coasy	< 0.5		N	0.682	Y
Q61245	Collagen alpha-1(XI) chain	Col11a1	0.521	CS pos: 34-35. VRG-AA. Pr: 0.4033	Y	≤0.5	N
Q8C166	Copine-1	Cpne1	< 0.5		N	0.642	Y
Q8BFS6	Serine/threonine-protein phosphatase CPPED	Cpped1	< 0.5		N	≤0.5	N
Q9EPU4	Cleavage and polyadenylation specificity facto	Cpsf1	< 0.5		N	≤0.5	N
Q64735	Complement component receptor 1-like protei	Cr1l	< 0.5		N	≤0.5	N
P23927	Alpha-crystallin B chain	Cryab	< 0.5		N	0.665	Y
P41241	Tyrosine-protein kinase CSK	Csk	< 0.5		N	≤0.5	N
Q62059	Versican core protein	Vcan	0.970	CS pos: 20-21. THA-LH. Pr: 0.6350	Y	≤0.5	N
Q9EPL2	Calsyntenin-1	Clstn1	0.975	CS pos: 28-29. VWA-AR. Pr: 0.9677	Y	≤0.5	N
Q7TSG2	RNA polymerase II subunit A C-terminal doma	Ctdp1	< 0.5		N	≤0.5	N
P30999	Catenin delta-1	Ctnnd1	< 0.5		N	≤0.5	N
Q9D5V5	Cullin-5	Cul5	< 0.5		N	≤0.5	N
Q9WTX6	Cullin-1	Cul1	< 0.5		N	≤0.5	N
Q7TMB8	Cytoplasmic FMR1-interacting protein 1	Cyfip1	< 0.5		N	≤0.5	N
O08967	Cytohesin-3	Cyth3	< 0.5		N	≤0.5	N
Q8CBY8	Dynactin subunit 4	Dctn4	< 0.5		N	≤0.5	N
Q99LD8	N(G),N(G)-dimethylarginine dimethylaminohyd	Ddah2	< 0.5		N	0.634	Y
Q62095	ATP-dependent RNA helicase DDX3Y	Ddx3y	< 0.5		N	≤0.5	N
Q810A7	ATP-dependent RNA helicase DDX42	Ddx42	< 0.5		N	≤0.5	N
O54786	DNA fragmentation factor subunit alpha	Dffa	< 0.5		N	0.686	Y
P49183	Deoxyribonuclease-1	Dnase1	0.706	CS pos: 22-23. AGT-LR. Pr: 0.3166	Y	0.654	N
Q9QYI3	DnaJ homolog subfamily C member 7	Dnajc7	< 0.5		N	≤0.5	N
Q8BUR4	Dedicator of cytokinesis protein 1	Dock1	< 0.5		N	≤0.5	N
Q8C147	Dedicator of cytokinesis protein 8	Dock8	< 0.5		N	≤0.5	N
O35098	Dihydropyrimidinase-related protein 4	Dpysl4	< 0.5		N	≤0.5	N
Q6NT99	Dual specificity protein phosphatase 23	Dusp23	< 0.5		N	0.673	Y
Q8BL66	Early endosome antigen 1	Eea1	< 0.5		N	≤0.5	N
P57776	Elongation factor 1-delta	Eef1d	< 0.5		N	0.729	Y
Q3UGC7	Eukaryotic translation initiation factor 3 subuni	Eif3j1	< 0.5		N	≤0.5	N
Q8JZQ9	Eukaryotic translation initiation factor 3 subuni	Eif3b	< 0.5		N	≤0.5	N
Q9Z1D1	Eukaryotic translation initiation factor 3 subuni	Eif3g	< 0.5		N	0.699	Y
Q9QZD9	Eukaryotic translation initiation factor 3 subuni	Eif3i	< 0.5		N	0.54	Y
Q05BC3	Echinoderm microtubule-associated protein-lik	Eml1	< 0.5		N	≤0.5	N
P59900	EMILIN-3	Emilin3	0.993	CS pos: 21-22. AQA-KG. Pr: 0.8177	Y	≤0.5	N
Q9R1E6	Ectonucleotide pyrophosphatase/phosphodiest	Enpp2	0.616	CS pos: 27-28. CLG-FT. Pr: 0.3400	Y	≤0.5	N
Q149F3	Eukaryotic peptide chain release factor GTP-b	Gspt2	< 0.5		N	≤0.5	N
Q9D172	Glutamine amidotransferase-like class 1 doma	Gatd3a	< 0.5		N	0.881	Y
Q8K0G8	Epithelial splicing regulatory protein 2	Esrp2	< 0.5		N	≤0.5	N
Q3U7R1	Extended synaptotagmin-1	Esyt1	< 0.5		N	≤0.5	N
Q8VBV3	Exosome complex component RRP4	Exosc2	< 0.5		N	≤0.5	N
Q3TCJ1	BRISC complex subunit Abraxas 2	Abraxas2	< 0.5		N	≤0.5	N
Q6DTY7	6-phosphofructo-2-kinase/fructose-2,6-bisphos	Pfkfb4	< 0.5		N	0.619	Y
Q9DCL2	Cytosolic iron-sulfur assembly component 2A	Ciao2a	< 0.5		N	0.626	Y
P23591	GDP-L-fucose synthase	Gfus	< 0.5		N	0.521	Y
Q91WF7	Polyphosphoinositide phosphatase	Fig4	< 0.5		N	≤0.5	N
Q62446	Peptidyl-prolyl cis-trans isomerase FKBP3	Fkbp3	< 0.5		N	0.714	Y
Q9JL26	Formin-like protein 1	Fmnl1	< 0.5		N	≤0.5	N
P50285	Dimethylaniline monooxygenase [N-oxide-forn	Fmo1	< 0.5		N	≤0.5	N
Q9WV91	Prostaglandin F2 receptor negative regulator	Ptgfrn	0.985	CS pos: 21-22. CRG-RV. Pr: 0.7689	Y	0.601	N
Q91XD4	Formimidoyltransferase-cyclodeaminase	Ftcd	< 0.5		N	0.515	Y
Q99LJ1	Tissue alpha-L-fucosidase	Fuca1	0.995	CS pos: 17-18. AVA-LA. Pr: 0.4277	Y	0.733	N
P97855	Ras GTPase-activating protein-binding protein	G3bp1	< 0.5		N	≤0.5	N
Q8BMP6	Golgi resident protein GCP60	Acbd3	< 0.5		N	≤0.5	N
Q61599	Rho GDP-dissociation inhibitor 2	Arhgdib	< 0.5		N	≤0.5	N
Q6P5E6	ADP-ribosylation factor-binding protein GGA2	Gga2	< 0.5		N	≤0.5	N

Q9CQM9	Glutaredoxin-3	Glrx3	< 0.5		N	0.602	Y
O08795	Glucosidase 2 subunit beta	Prkcsh	0.999	CS pos: 14-15. CWA-VE. Pr: 0.9414	Y	≤0.5	N
Q8K0C9	GDP-mannose 4,6 dehydratase	Gmds	< 0.5		N	≤0.5	N
Q922H4	Mannose-1-phosphate guanyltransferase alph	Gmppa	< 0.5		N	0.588	Y
Q99L27	GMP reductase 2	Gmpr2	< 0.5		N	0.522	Y
O70325	Phospholipid hydroperoxide glutathione peroxi	Gpx4	0.614	CS pos: 25-26. GLA-GT. Pr: 0.4068	Y	0.887	N
Q91Z53	Glyoxylate reductase/hydroxypyruvate reducta	Grhpr	< 0.5		N	≤0.5	N
Q9D8T2	Gasdermin-D	Gsdmd	< 0.5		N	≤0.5	N
P97494	Glutamate--cysteine ligase catalytic subunit	Gclc	< 0.5		N	≤0.5	N
P51855	Glutathione synthetase	Gss	< 0.5		N	0.512	Y
Q61133	Glutathione S-transferase theta-2	Gstt2	< 0.5		N	0.611	Y
P51859	Hepatoma-derived growth factor	Hdgf	< 0.5		N	0.537	Y
Q9R257	Heme-binding protein 1	Hebp1	< 0.5		N	≤0.5	N
P36552	Oxygen-dependent coproporphyrinogen-III oxi	Cpox	< 0.5		N	≤0.5	N
O54879	High mobility group protein B3	Hmgb3	< 0.5		N	≤0.5	N
P35492	Histidine ammonia-lyase	Hal	< 0.5		N	≤0.5	N
P34914	Bifunctional epoxide hydrolase 2	Ephx2	< 0.5		N	≤0.5	N
P13597	Intercellular adhesion molecule 1	Icam1	0.996	CS pos: 27-28. GDA-QV. Pr: 0.9331	Y	0.539	N
Q9JHR7	Insulin-degrading enzyme	Ide	< 0.5		N	≤0.5	N
Q60872	Eukaryotic translation initiation factor 1A	Eif1a	< 0.5		N	≤0.5	N
Q05D44	Eukaryotic translation initiation factor 5B	Eif5b	< 0.5		N	≤0.5	N
P63073	Eukaryotic translation initiation factor 4E	Eif4e	< 0.5		N	0.775	Y
Q62448	Eukaryotic translation initiation factor 4 gamma	Eif4g2	< 0.5		N	≤0.5	N
Q9WUK2	Eukaryotic translation initiation factor 4H	Eif4h	< 0.5		N	0.809	Y
Q61730	Interleukin-1 receptor accessory protein	Il1rap	0.976	CS pos: 20-21. SHA-SE. Pr: 0.9026	Y	≤0.5	N
P24547	Inosine-5'-monophosphate dehydrogenase 2	Impdh2	< 0.5		N	≤0.5	N
Q80V26	Golgi-resident adenosine 3',5'-bisphosphate 3'	Bpnt2	< 0.5		N	0.521	Y
Q9JHU9	Inositol-3-phosphate synthase 1	Isyna1	< 0.5		N	0.517	Y
Q91YE6	Importin-9	Ipo9	< 0.5		N	0.569	Y
Q64287	Interferon regulatory factor 4	Irf4	< 0.5		N	≤0.5	N
Q9CX00	IST1 homolog	Ist1	< 0.5		N	≤0.5	N
O88792	Junctional adhesion molecule A	F11r	0.968	CS pos: 26-27. VQG-KG. Pr: 0.7632	Y	≤0.5	N
Q9D3R6	Katanin p60 ATPase-containing subunit A-like	Katnal2	< 0.5		N	≤0.5	N
Q9DBP5	UMP-CMP kinase	Cmpk1	< 0.5		N	0.613	Y
B1AVY7	Kinesin-like protein KIF16B	Kif16b	< 0.5		N	≤0.5	N
Q8C078	Calcium/calmodulin-dependent protein kinase	Camkk2	< 0.5		N	≤0.5	N
P68404	Protein kinase C beta type	Prkcb	< 0.5		N	≤0.5	N
Q61789	Laminin subunit alpha-3	Lama3	0.993	CS pos: 31-32. SWS-VV. Pr: 0.7216	Y	≤0.5	N
O89112	Glutathione S-transferase LANCL1	Lancl1	< 0.5		N	0.517	Y
Q5SUF2	Luc7-like protein 3	Luc7l3	< 0.5		N	0.571	Y
Q07797	Galectin-3-binding protein	Lgals3bp	0.999	CS pos: 18-19. TQG-TE. Pr: 0.9788	Y	0.81	N
O89017	Legumain	Lgmn	0.997	CS pos: 17-18. AGA-VP. Pr: 0.7875	Y	0.703	N
Q8BFW7	Lipoma-preferred partner homolog	Lpp	< 0.5		N	0.561	Y
Q99N69	Leupaxin	Lpxn	< 0.5		N	0.565	Y
Q9ESE1	Lipopolysaccharide-responsive and beige-like	Lrba	< 0.5		N	0.615	Y
Q922Q8	Leucine-rich repeat-containing protein 59	Lrrc59	< 0.5		N	0.663	Y
Q3UZ39	Leucine-rich repeat flightless-interacting protei	Lrrfip1	< 0.5		N	≤0.5	N
Q9QXA5	U6 snRNA-associated Sm-like protein LSm4	Lsm4	< 0.5		N	0.61	Y
Q9CYI4	Putative RNA-binding protein Luc7-like 1	Luc7l	< 0.5		N	0.536	Y
A2AHG0	Leucine zipper putative tumor suppressor 3	Lzts3	< 0.5		N	≤0.5	N
O08663	Methionine aminopeptidase 2	Metap2	< 0.5		N	≤0.5	N
P27546	Microtubule-associated protein 4	Map4	< 0.5		N	≤0.5	N
Q61166	Microtubule-associated protein RP/EB family r	Mapre1	< 0.5		N	≤0.5	N
Q99LB6	Methionine adenosyltransferase 2 subunit beta	Mat2b	< 0.5		N	0.508	Y
O89029	Matrilin-4	Matn4	0.993	CS pos: 25-26. LQS-AG. Pr: 0.5764	Y	0.539	N
O35701	Matrilin-3	Matn3	0.973	CS pos: 27-28. SLA-AP. Pr: 0.7599	Y	0.622	N
Q7TPV4	Myb-binding protein 1A	Mybbp1a	< 0.5		N	≤0.5	N
P49717	DNA replication licensing factor MCM4	Mcm4	< 0.5		N	≤0.5	N
Q61881	DNA replication licensing factor MCM7	Mcm7	< 0.5		N	≤0.5	N
Q91VH6	Protein MEMO1	Memo1	< 0.5		N	0.73	Y
O88559	Menin	Men1	< 0.5		N	≤0.5	N
Q8VE43	Meteorin-like protein	Metrnl	0.997	CS pos: 45-46. ASA-QY. Pr: 0.9589	Y	0.883	N
P55002	Microfibrillar-associated protein 2	Mfap2	0.999	CS pos: 18-19. AQG-QY. Pr: 0.8479	Y	0.882	N
P34884	Macrophage migration inhibitory factor	Mif	< 0.5		N	0.707	Y
Q8VI63	MOB kinase activator 2	Mob2	< 0.5		N	0.513	Y
A2BDX3	Adenylyltransferase and sulfurtransferase MO	Mocs3	< 0.5		N	0.617	Y

P47809	Dual specificity mitogen-activated protein kina	Map2k4	< 0.5		N	0.582	Y
Q8VEM8	Phosphate carrier protein, mitochondrial	Slc25a3	< 0.5		N	≤0.5	N
Q9D6Y7	Mitochondrial peptide methionine sulfoxide rec	Msra	< 0.5		N	0.784	Y
Q9R190	Metastasis-associated protein MTA2	Mta2	< 0.5		N	≤0.5	N
P62774	Myotrophin	Mtpn	< 0.5		N	≤0.5	N
Q9DBV4	Matrix remodeling-associated protein 8	Mxra8	0.906	CS pos: 22-23. VLS-SG. Pr: 0.4909	Y	≤0.5	N
Q5SYD0	Unconventional myosin-Id	Myo1d	< 0.5		N	≤0.5	N
O70624	Myocilin	Myoc	0.984	CS pos: 20-21. ART-AQ. Pr: 0.5808	Y	≤0.5	N
Q9DBR7	Protein phosphatase 1 regulatory subunit 12A	Ppp1r12a	< 0.5		N	≤0.5	N
Q6PGB6	N-alpha-acetyltransferase 50	Naa50	< 0.5		N	0.501	Y
Q6ZQA0	Neurobeachin-like protein 2	Nbeal2	< 0.5		N	0.648	Y
Q91W39	Nuclear receptor coactivator 5	Ncoa5	< 0.5		N	≤0.5	N
Q9QYG0	Protein NDRG2	Ndrg2	< 0.5		N	≤0.5	N
Q3UHN9	Bifunctional heparan sulfate N-deacetylase/N-	Ndst1	< 0.5		N	0.585	Y
P29595	NEDD8	Nedd8	< 0.5		N	≤0.5	N
Q8BZW8	NHL repeat-containing protein 2	Nhlrc2	< 0.5		N	0.617	Y
Q9CRB2	H/ACA ribonucleoprotein complex subunit 2	Nhp2	< 0.5		N	≤0.5	N
Q8K2T1	NmrA-like family domain-containing protein 1	Nmral1	< 0.5		N	0.584	Y
Q3UFM5	Nucleolar MIF4G domain-containing protein 1	Nom1	< 0.5		N	≤0.5	N
Q7TNH6	Nephrocystin-3	Nphp3	< 0.5		N	≤0.5	N
Q8R480	Nuclear pore complex protein Nup85	Nup85	< 0.5		N	≤0.5	N
O35103	Osteomodulin	Omd	0.983	CS pos: 20-21. VYC-QY. Pr: 0.9211	Y	0.626	N
P47713	Cytosolic phospholipase A2	Pla2g4a	< 0.5		N	≤0.5	N
Q9CY58	Plasminogen activator inhibitor 1 RNA-binding	Serbp1	< 0.5		N	≤0.5	N
Q9D0B6	Protein PBDC1	Pbdc1	< 0.5		N	≤0.5	N
O55134	Protocadherin-12	Pcdh12	0.869	CS pos: 18-19. SYL-FI. Pr: 0.2933	Y	≤0.5	N
Q3UA06	Pachytene checkpoint protein 2 homolog	Trip13	< 0.5		N	0.513	Y
Q9QYX7	Protein piccolo	Pclo	< 0.5		N	≤0.5	N
P17918	Proliferating cell nuclear antigen	Pcna	< 0.5		N	0.562	Y
Q9D5U0	Lysophosphatidylcholine acyltransferase 2B	Lpcat2b	< 0.5		N	0.571	Y
Q922E4	Ethanolamine-phosphate cytidylyltransferase	Pcyt2	< 0.5		N	≤0.5	N
Q9Z0X4	cGMP-inhibited 3',5'-cyclic phosphodiesterase	Pde3a	< 0.5		N	≤0.5	N
P23440	Rod cGMP-specific 3',5'-cyclic phosphodiester	Pde6b	< 0.5		N	≤0.5	N
Q8CI19	Platelet-derived growth factor C	Pdgfc	0.974	CS pos: 14-15. ALA-GQ. Pr: 0.4863	Y	0.807	N
O70400	PDZ and LIM domain protein 1	Pdlim1	< 0.5		N	≤0.5	N
Q4VA53	Sister chromatid cohesion protein PDS5 homo	Pds5b	< 0.5		N	≤0.5	N
Q99K01	Pyridoxal-dependent decarboxylase domain-c	Pdxdc1	< 0.5		N	≤0.5	N
Q8K183	Pyridoxal kinase	Pdxk	< 0.5		N	0.656	Y
Q9QY39	PDZ domain-containing protein 4	Pdzd4	< 0.5		N	0.55	Y
Q11136	Xaa-Pro dipeptidase	Pepd	< 0.5		N	≤0.5	N
P61759	Prefoldin subunit 3	Vbp1	< 0.5		N	0.575	Y
P47857	ATP-dependent 6-phosphofructokinase, muscl	Pfkm	< 0.5		N	≤0.5	N
P28654	Decorin	Dcn	0.999	CS pos: 16-17. SWA-GP. Pr: 0.9586	Y	0.533	N
Q9DCE5	p21-activated protein kinase-interacting protei	Pak1ip1	< 0.5		N	≤0.5	N
Q62077	1-phosphatidylinositol 4,5-bisphosphate phosp	Plcg1	< 0.5		N	≤0.5	N
Q9JHK5	Pleckstrin	Plek	< 0.5		N	≤0.5	N
Q9R0E2	Procollagen-lysine,2-oxoglutarate 5-dioxygena	Plod1	0.998	CS pos: 18-19. VQA-KD. Pr: 0.9701	Y	0.571	N
P70208	Plexin-A3	Plxna3	0.981	CS pos: 19-20. CLG-SS. Pr: 0.6848	Y	≤0.5	N
Q9R0M4	Podocalyxin	Podxl	0.987	CS pos: 21-22. SHS-HN. Pr: 0.8822	Y	≤0.5	N
P97814	Proline-serine-threonine phosphatase-interacti	Pstpip1	< 0.5		N	≤0.5	N
Q60676	Serine/threonine-protein phosphatase 5	Ppp5c	< 0.5		N	≤0.5	N
O08709	Peroxiredoxin-6	Prdx6	< 0.5		N	≤0.5	N
Q08761	Vitamin K-dependent protein S	Pros1	0.895	CS pos: 24-25. SET-NF. Pr: 0.4986	Y	0.65	N
Q9DAW6	U4/U6 small nuclear ribonucleoprotein Prp4	Prpf4	< 0.5		N	≤0.5	N
Q99JF8	PC4 and SFRS1-interacting protein	Psip1	< 0.5		N	0.636	Y
Q99JI4	26S proteasome non-ATPase regulatory subu	Psmd6	< 0.5		N	≤0.5	N
Q9CX56	26S proteasome non-ATPase regulatory subu	Psmd8	< 0.5		N	≤0.5	N
P97371	Proteasome activator complex subunit 1	Psme1	< 0.5		N	≤0.5	N
P35831	Tyrosine-protein phosphatase non-receptor typ	Ptpn12	< 0.5		N	≤0.5	N
P46664	Adenylosuccinate synthetase isozyme 2	Adss2	< 0.5		N	≤0.5	N
Q3UQ28	Peroxidasin homolog	Pxdn	0.954	CS pos: 26-27. VVA-SK. Pr: 0.4729	Y	≤0.5	N
P70303	CTP synthase 2	Ctps2	< 0.5		N	≤0.5	N
O35551	Rab GTPase-binding effector protein 1	Rabep1	< 0.5		N	≤0.5	N
P46061	Ran GTPase-activating protein 1	Rangap1	< 0.5		N	≤0.5	N
P63321	Ras-related protein Ral-A	Rala	< 0.5		N	≤0.5	N
Q8BX09	Retinoblastoma-binding protein 5	Rbbp5	< 0.5		N	≤0.5	N

Q8BMG7	Rab3 GTPase-activating protein non-catalytic	Rab3gap2	< 0.5		N	≤0.5	N
B2RY56	RNA-binding protein 25	Rbm25	< 0.5		N	0.58	Y
Q05186	Reticulocalbin-1	Rcn1	0.967	CS pos: 23-24. LRA-KP. Pr: 0.6589	Y	0.854	N
Q00915	Retinol-binding protein 1	Rbp1	< 0.5		N	≤0.5	N
Q00724	Retinol-binding protein 4	Rbp4	0.996	CS pos: 18-19. GSA-ER. Pr: 0.8475	Y	0.553	N
Q5FWK3	Rho GTPase-activating protein 1	Arhgap1	< 0.5		N	0.806	Y
Q8BWR8	Rhophilin-2	Rhpn2	< 0.5		N	≤0.5	N
P47963	60S ribosomal protein L13	Rpl13	< 0.5		N	≤0.5	N
O09167	60S ribosomal protein L21	Rpl21	< 0.5		N	0.765	Y
P27659	60S ribosomal protein L3	Rpl3	< 0.5		N	0.537	Y
Q9D8E6	60S ribosomal protein L4	Rpl4	< 0.5		N	≤0.5	N
P47962	60S ribosomal protein L5	Rpl5	< 0.5		N	≤0.5	N
P14148	60S ribosomal protein L7	Rpl7	< 0.5		N	≤0.5	N
Q7TPD3	Roundabout homolog 2	Robo2	0.998	CS pos: 21-22. IDG-SR. Pr: 0.9111	Y	≤0.5	N
Q80UW8	DNA-directed RNA polymerases I, II, and III su	Polr2e	< 0.5		N	≤0.5	N
P08775	DNA-directed RNA polymerase II subunit RPB	Polr2a	< 0.5		N	≤0.5	N
Q9CSH3	Exosome complex exonuclease RRP44	Dis3	< 0.5		N	≤0.5	N
Q99P72	Reticulon-4	Rtn4	< 0.5		N	≤0.5	N
P57784	U2 small nuclear ribonucleoprotein A'	Snrpa1	< 0.5		N	0.856	Y
Q8R4C2	RUN and FYVE domain-containing protein 2	Rufy2	< 0.5		N	≤0.5	N
P56565	Protein S100-A1	S100a1	< 0.5		N	0.596	Y
P27005	Protein S100-A8	S100a8	< 0.5		N	≤0.5	N
Q9Z0L1	Sphingosine 1-phosphate receptor 4	S1pr4	< 0.5		N	≤0.5	N
Q9D0K2	Succinyl-CoA:3-ketoacid coenzyme A transfer	Oxct1	< 0.5		N	≤0.5	N
Q9D1M0	Protein SEC13 homolog	Sec13	< 0.5		N	0.644	Y
O09126	Semaphorin-4D	Sema4d	0.963	CS pos: 23-24. AVA-FA. Pr: 0.8031	Y	≤0.5	N
Q76KF0	Semaphorin-6D	Sema6d	0.994	CS pos: 20-21. LRA-VS. Pr: 0.5795	Y	≤0.5	N
Q62419	Endophilin-A2	Sh3gl1	< 0.5		N	0.877	Y
Q91VW3	SH3 domain-binding glutamic acid-rich-like pro	Sh3bgrl3	< 0.5		N	0.758	Y
P46062	Signal-induced proliferation-associated protein	Sipa1	< 0.5		N	≤0.5	N
Q8CG47	Structural maintenance of chromosomes prote	Smc4	< 0.5		N	≤0.5	N
O09044	Synaptosomal-associated protein 23	Snap23	< 0.5		N	0.758	Y
Q62189	U1 small nuclear ribonucleoprotein A	Snrpa	< 0.5		N	0.738	Y
O70493	Sorting nexin-12	Snx12	< 0.5		N	0.697	Y
Q3UHD6	Sorting nexin-27	Snx27	< 0.5		N	≤0.5	N
Q9CY18	Sorting nexin-7	Snx7	< 0.5		N	≤0.5	N
Q9EPT5	Solute carrier organic anion transporter family	Slco2a1	< 0.5		N	0.538	Y
Q6P069	Sorcin	Sri	< 0.5		N	0.928	Y
Q91WP6	Serine protease inhibitor A3N	Serpina3n	0.985	CS pos: 20-21. VLC-FP. Pr: 0.8322	Y	0.798	N
Q8BH69	Selenide, water dikinase 1	Sephs1	< 0.5		N	0.536	Y
O55201	Transcription elongation factor SPT5	Supt5h	< 0.5		N	≤0.5	N
P05480	Neuronal proto-oncogene tyrosine-protein kina	Src	< 0.5		N	≤0.5	N
O35326	Serine/arginine-rich splicing factor 5	Srsf5	< 0.5		N	≤0.5	N
Q8BL97	Serine/arginine-rich splicing factor 7	Srsf7	< 0.5		N	0.541	Y
Q9JM90	Signal-transducing adaptor protein 1	Stap1	< 0.5		N	0.745	Y
P42227	Signal transducer and activator of transcription	Stat3	< 0.5		N	0.521	Y
Q5DQR4	Syntaxin-binding protein 5-like	Stxbp5l	< 0.5		N	≤0.5	N
O55098	Serine/threonine-protein kinase 10	Stk10	< 0.5		N	0.658	Y
Q91VJ4	Serine/threonine-protein kinase 38	Stk38	< 0.5		N	≤0.5	N
Q9JI11	Serine/threonine-protein kinase 4	Stk4	< 0.5		N	≤0.5	N
Q9Z1Z2	Serine-threonine kinase receptor-associated p	Strap	< 0.5		N	≤0.5	N
Q64324	Syntaxin-binding protein 2	Stxbp2	< 0.5		N	≤0.5	N
Q8CGC7	Bifunctional glutamate/proline--tRNA ligase	Eprs1	< 0.5		N	≤0.5	N
Q6P8M1	Putative deoxyribonuclease TATDN1	Tatdn1	< 0.5		N	≤0.5	N
Q8BHJ5	F-box-like/WD repeat-containing protein TBL1	Tbl1xr1	< 0.5		N	0.593	Y
P10711	Transcription elongation factor A protein 1	Tcea1	< 0.5		N	≤0.5	N
P47226	Testin	Tes	< 0.5		N	≤0.5	N
Q04207	Transcription factor p65	Rela	< 0.5		N	≤0.5	N
Q9ER58	Testican-2	Spock2	0.998	CS pos: 24-25. AEG-DA. Pr: 0.4434	Y	≤0.5	N
Q62318	Transcription intermediary factor 1-beta	Trim28	< 0.5		N	≤0.5	N
Q99JR5	Tubulointerstitial nephritis antigen-like	Tinagl1	0.996	CS pos: 21-22. LEA-RR. Pr: 0.4314	Y	0.893	N
O88746	Target of Myb protein 1	Tom1	< 0.5		N	0.7	Y
Q9JLT2	Trehalase	Treh	0.985	CS pos: 20-21. QEA-LP. Pr: 0.4433	Y	0.651	N
Q8C0E3	E3 ubiquitin-protein ligase TRIM47	Trim47	< 0.5		N	≤0.5	N
Q80XC2	tRNA (adenine(58)-N(1))-methyltransferase ca	Trmt61a	< 0.5		N	0.757	Y
Q8R3G9	Tetraspanin-8	Tspan8	< 0.5		N	0.62	Y

P07309	Transthyretin	Ttr	0.999	CS pos: 20-21. SEA-GP. Pr: 0.7597	Y	0.711	N
Q91WM3	U3 small nucleolar RNA-interacting protein 2	Rrp9	< 0.5		N	≤0.5	N
Q91YN5	UDP-N-acetylhexosamine pyrophosphorylase	Uap1	< 0.5		N	0.51	Y
E9Q735	Ubiquitin conjugation factor E4 A	Ube4a	< 0.5		N	0.643	Y
Q9ES00	Ubiquitin conjugation factor E4 B	Ube4b	< 0.5		N	≤0.5	N
P21126	Ubiquitin-like protein 4A	Ubl4a	< 0.5		N	0.592	Y
P35123	Ubiquitin carboxyl-terminal hydrolase 4	Usp4	< 0.5		N	≤0.5	N
Q6A4J8	Ubiquitin carboxyl-terminal hydrolase 7	Usp7	< 0.5		N	≤0.5	N
P58321	Ubiquitin carboxyl-terminal hydrolase isozyme	Uchl4	< 0.5		N	0.583	Y
Q3U1V6	Ubiquitin-conjugating enzyme E2 variant 3	Uevld	< 0.5		N	0.521	Y
Q6P5E4	UDP-glucose:glycoprotein glucosyltransferase	Uggt1	0.624	CS pos: 42-43. AEA-NS. Pr: 0.4539	Y	≤0.5	N
P35969	Vascular endothelial growth factor receptor 1	Flt1	0.789	CS pos: 26-27. GSG-SK. Pr: 0.4938	Y	≤0.5	N
Q8VDJ3	Vigilin	Hdlbp	< 0.5		N	≤0.5	N
Q9Z0K8	Pantetheinase	Vnn1	0.861	CS pos: 23-24. ASS-LD. Pr: 0.4053	Y	0.824	N
P40336	Vacuolar protein sorting-associated protein 26	Vps26a	< 0.5		N	≤0.5	N
Q91XD6	Vacuolar protein-sorting-associated protein 36	Vps36	< 0.5		N	≤0.5	N
A6PWY4	WD repeat-containing protein 76	Wdr76	< 0.5		N	≤0.5	N
Q04736	Tyrosine-protein kinase Yes	Yes1	< 0.5		N	≤0.5	N
P59326	YTH domain-containing family protein 1	Ythdf1	< 0.5		N	≤0.5	N
Q0P678	Zinc finger CCCH domain-containing protein 1	Zc3h18	< 0.5		N	≤0.5	N
A2A791	Zinc finger MYM-type protein 4	Zmym4	< 0.5		N	≤0.5	N
Q9Z0U1	Tight junction protein ZO-2	Tjp2	< 0.5		N	≤0.5	N
Q91XZ4	Protocadherin beta-6	Pcdhb6	0.974	CS pos: 30-31. SEA-IR. Pr: 0.7444	Y	0.605	N
Q8BML9	Glutamine--tRNA ligase	Qars1	< 0.5		N	≤0.5	N
E9Q4N7	AT-rich interactive domain-containing protein 1	Arid1b	< 0.5		N	≤0.5	N
E9Q5C9	Nucleolar and coiled-body phosphoprotein 1	Nolc1	< 0.5		N	≤0.5	N
E9PVA8	eIF-2-alpha kinase activator GCN1	Gcn1	< 0.5		N	≤0.5	N
E9Q2M9	WD repeat- and FYVE domain-containing prot	Wdfy4	< 0.5		N	0.613	Y
Q99JX4	Eukaryotic translation initiation factor 3 subuni	Eif3m	< 0.5		N	≤0.5	N
Q9CPP0	Nucleoplasmin-3	Npm3	< 0.5		N	0.667	Y
O88796	Ribonuclease P protein subunit p30	Rpp30	< 0.5		N	≤0.5	N
Q6PER3	Microtubule-associated protein RP/EB family r	Mapre3	< 0.5		N	≤0.5	N
Q9CQR2	40S ribosomal protein S21	Rps21	< 0.5		N	0.728	Y
Q99PW4	EKC/KEOPS complex subunit Tp53rk	Tp53rk	< 0.5		N	≤0.5	N
P25206	DNA replication licensing factor MCM3	Mcm3	< 0.5		N	≤0.5	N
P21855	B-cell differentiation antigen CD72	Cd72	< 0.5		N	0.938	Y
Q61249	Immunoglobulin-binding protein 1	Igbp1	< 0.5		N	≤0.5	N
Q99MI6	GTPase IMAP family member 3	Gimap3	< 0.5		N	≤0.5	N
Q61781	Keratin, type I cytoskeletal 14	Krt14	< 0.5		N	≤0.5	N
Q9Z315	U4/U6.U5 tri-snRNP-associated protein 1	Sart1	< 0.5		N	≤0.5	N
Q9CS42	Ribose-phosphate pyrophosphokinase 2	Prps2	< 0.5		N	≤0.5	N
Q6ZWV7	60S ribosomal protein L35	Rpl35	< 0.5		N	≤0.5	N
Q9JMG1	Endothelial differentiation-related factor 1	Edf1	< 0.5		N	0.704	Y
P63280	SUMO-conjugating enzyme UBC9	Ube2i	< 0.5		N	0.749	Y
Q9JJW0	Peroxisomal membrane protein 4	Pxmp4	< 0.5		N	0.689	Y
Q8CCJ3	E3 UFM1-protein ligase 1	Ufl1	< 0.5		N	≤0.5	N
Q9JJQ0	GPI mannosyltransferase 3	Pigb	< 0.5		N	0.539	Y
P52432	DNA-directed RNA polymerases I and III subu	Polr1c	< 0.5		N	≤0.5	N
Q8BGE6	Cysteine protease ATG4B	Atg4b	< 0.5		N	≤0.5	N
Q9CWY8	Ribonuclease H2 subunit A	Rnaseh2a	< 0.5		N	0.536	Y
Q99L88	Beta-1-syntrophin	Sntb1	< 0.5		N	0.504	Y
Q8R3F9	Speckle targeted PIP5K1A-regulated poly(A) p	Tut1	< 0.5		N	≤0.5	N
P26369	Splicing factor U2AF 65 kDa subunit	U2af2	< 0.5		N	≤0.5	N
Q9CZN7	Serine hydroxymethyltransferase, mitochondri	Shmt2	< 0.5		N	0.548	Y
