# Supplementary material for: Proteomic Profiling and Functional Analysis of B Cell-Derived Exosomes upon Pneumocystis Infection
Source: J Immunol Res. 2022 Apr 14;2022:5187166. doi: 10.1155/2022/5187166 (PMC9023222; doi:10.1155/2022/5187166)
Supplement: Supplementary Materials — Supplementary Table 1: initial culture cell number and protein amount of B cell exosomes used for mass spectrometry. Supplementary Table 2: all identified proteins of uninfected and Pneumocystis-infected B cell exosomes. Supplementary Table 3: SignalP and SecretomeP prediction results. Supplementary Table 4: differentially expressed proteins of B cell exosomes in response to PCP. Supplementary Table 5: quantitative information of peptides and proteins by PRM analysis. [file 5187166.f1.zip › Supplementary Table 4-differentially expressed proteins of B-cell exosomes in response to PCP.pdf]

Supplementary Table 4：differentially expressed proteins of B-cell exosomes in response to PCP

Accession	Protein Name	Gene Name	Function	PCP-1/REF	PCP-2/REF	PCP-3/REF	average PCP	CON-1/REF	CON-2/REF	CON-3/REF	average CON	PCP/CON	t test p value	GO annotations
Q5SX39	Myosin-4	Myh4	FUNCTION: Muscle contr	1.657100194	1.746573863	1.282663218	1.562112425	0.600384258	0.618227523	0.52331262	0.580641467	2.690321849	0.002488473	GO:0006936(BP:muscle contraction);GO:001
P43277	Histone H1.3	H1f3	FUNCTION: Histone H1 p	1.151231366	1.858662882	1.381686795	1.463860348	0.611268356	0.615002113	0.753691078	0.659987182	2.218013299	0.019690798	GO:0030261(BP:chromosome condensation)
O35744	Chitinase-like protein 3	Chil3	FUNCTION: Lectin that bi	1.341700016	1.492073521	1.404494042	1.41275586	0.687836014	0.680807395	0.672621687	0.680421699	2.076294543	7.51541E-05	GO:0006032(BP:chitin catabolic process);GC
P27661	Histone H2AX	H2ax	FUNCTION: Variant histo	1.540539119	1.733396455	1.052429851	1.442121808	0.688867445	0.662678394	0.772144514	0.707896785	2.037192201	0.023248143	GO:0006974(BP:cellular response to DNA da
Q9CQE8	RNA transcription, translation and tr	RTRAF	FUNCTION: RNA-binding	1.284563198	1.935076175	1.103906807	1.44118206	0.701375017	0.778935649	0.666488553	0.71559974	2.01394995	0.046386153	GO:0006469(BP:negative regulation of protei
P43274	Histone H1.4	H1f4	FUNCTION: Histone H1 p	1.343234621	1.593107874	1.133766187	1.356702894	0.732364917	0.661970847	0.785782235	0.726706	1.866921278	0.010176574	GO:0030261(BP:chromosome condensation)
P56716	Oxygen-regulated protein 1	Rp1	FUNCTION: Microtubule-a	1.507320481	1.416880164	1.126857909	1.350352851	0.702471116	0.682232897	0.812862022	0.732522012	1.843429725	0.007102318	GO:0035082(BP:axoneme assembly);GO:007
O54962	Barrier-to-autointegration factor	Banf1	FUNCTION: Plays fundar	1.304927899	1.582616834	1.068660436	1.318735056	0.721193022	0.652517106	0.892188587	0.755299572	1.745976174	0.026774437	GO:0030261(BP:chromosome condensation)
P0C0S6	Histone H2A.Z	H2az1	FUNCTION: Variant histo	1.463547518	1.332312846	1.105575606	1.300478657	0.78842783	0.714009205	0.791060859	0.764499298	1.701085482	0.007581658	GO:0071392(BP:cellular response to estradic
P43276	Histone H1.5	H1f5	FUNCTION: Histone H1 p	1.279192964	1.452945464	1.176883905	1.303007445	0.769251474	0.72923691	0.856699663	0.785062682	1.659749564	0.004328963	GO:0030261(BP:chromosome condensation)
Q91Z83	Myosin-7	Myh7	FUNCTION: Myosins are	1.409173078	1.265596922	1.181881395	1.285550465	0.987973884	0.894506916	0.464852138	0.782444313	1.642992919	0.044655152	GO:0007512(BP:adult heart development);GC
Q6GSS7	Histone H2A type 2-A	h2ac18	FUNCTION: Core compo	1.315678666	1.524099728	1.030884659	1.290221018	0.757040766	0.796940707	0.872746573	0.808909349	1.595013112	0.03061486	GO:0006325(BP:chromatin organization);GO:
P15864	Histone H1.2	H1f2	FUNCTION: Histone H1 p	1.138421536	1.570831821	1.084040701	1.264431353	0.78692104	0.754840758	0.87079175	0.804184516	1.572314969	0.043416123	GO:0030261(BP:chromosome condensation)
P68433	Histone H3.1	H3c1	FUNCTION: Core compo	1.214058082	1.412340244	1.167015057	1.264471127	0.777517557	0.791979762	0.848366576	0.805954632	1.56891105	0.004227153	GO:0006335(BP:DNA replication-dependent n
Q8CGP7	Histone H2A type 1-K	H2ac15	FUNCTION: Core compo	1.271885843	1.522107977	1.016035498	1.270009773	0.721248528	0.778707582	0.936763606	0.812239906	1.563589481	0.045608714	GO:0006325(BP:chromatin organization);GO:
Q3UPH1	Protein PRRC1	Prrc1		1.211367409	1.376658689	1.186596669	1.258207589	0.833226894	0.827170556	0.786721614	0.815706354	1.54247614	0.001966972	GO:0034199(BP:activation of protein kinase A
P62806	Histone H4	H4c1	FUNCTION: Core compo	1.26775815	1.345100988	1.125838089	1.246232409	0.805807901	0.80787076	0.852406665	0.822028442	1.516045364	0.003009501	GO:0006335(BP:DNA replication-dependent n
Q8CGP0	Histone H2B type 3-B	H2bu1-ps	FUNCTION: Core compo	1.182769635	1.264111087	1.259604687	1.235495137	0.769579288	0.851285886	0.828888343	0.816584505	1.513003404	0.000309331	GO:0006334(BP:nucleosome assembly);GO:
P10854	Histone H2B type 1-M	H2bc14	FUNCTION: Core compo	1.159025161	1.236874803	1.264072807	1.219990924	0.778920648	0.893519586	0.809725094	0.827388443	1.474508055	0.001078887	GO:0006334(BP:nucleosome assembly);GO:
P10922	Histone H1.0	H1f0	FUNCTION: Histones H1	1.277831095	1.401730165	0.981099788	1.220220349	0.81788547	0.814430015	0.867136735	0.83315074	1.464585327	0.037177785	GO:0006342(BP:chromatin silencing);GO:003
P02301	Histone H3.3C	H3f3c	FUNCTION: Core compo	1.119923716	1.346806376	1.167216437	1.21131551	0.797609612	0.868115106	0.827130936	0.830951885	1.457744464	0.006179027	GO:0006334(BP:nucleosome assembly);GO:
P70296	Phosphatidylethanolamine-binding p	Pebp1	FUNCTION: Binds ATP, o	1.244968017	1.247482717	1.149500846	1.21398386	0.85272141	0.950961745	0.719388931	0.841024028	1.443459187	0.007440774	GO:0007568(BP:aging);GO:0042755(BP:eatin
P19467	Mucin-13	Muc13	FUNCTION: Epithelial an	1.230600309	1.237722951	1.146390785	1.204904682	0.84736789	0.839272246	0.853444656	0.846694931	1.42306826	0.000267987	GO:0030277(BP:maintenance of gastrointesti
P16331	Phenylalanine-4-hydroxylase	Pah	FUNCTION: Catalyzes th	1.085634777	1.250901431	1.207244638	1.181260282	0.826249811	0.895696984	0.826865756	0.849604183	1.390365425	0.003699487	GO:0006559(BP:L-phenylalanine catabolic pr
Q61116	Zinc finger protein 235	Znf235	FUNCTION: May be invol	1.213937753	1.321449792	1.032347023	1.189244856	0.870793765	0.850535899	0.89195162	0.871093761	1.365231745	0.020229673	GO:0006355(BP:regulation of transcription, D
Q9CZT6	Protein CMSS1	Cmss1		1.265751076	1.276804224	1.016817792	1.186457697	0.941033901	0.944648247	0.726974723	0.870885624	1.362357656	0.047065875	
Q9Z277	Tyrosine-protein kinase BAZ1B	Baz1b	FUNCTION: Atypical tyro	1.163912788	1.187210388	1.172802373	1.17464185	0.864605677	0.883930466	0.857611411	0.868715851	1.352158877	7.93234E-06	GO:0006974(BP:cellular response to DNA da
P47915	60S ribosomal protein L29	Rpl29	FUNCTION: Component o	1.179753304	1.091339372	1.197103773	1.156065483	0.807716439	0.903185562	0.906149704	0.872350568	1.325230389	0.003513417	GO:0008283(BP:cell proliferation);GO:00315
P20152	Vimentin	Vim	FUNCTION: Vimentins ar	1.231567807	1.233420532	1.015088769	1.160025703	0.936743826	0.864563301	0.830470547	0.877259225	1.322329443	0.023136754	GO:0014002(BP:astrocyte development);GO:
Q60870	Receptor expression-enhancing pro	Reep5	FUNCTION: May promote	1.155324864	1.141602746	1.165164134	1.154030581	0.931608459	0.985842409	0.729723016	0.88239127	1.307844514	0.025521542	GO:0071782(CC:endoplasmic reticulum tubul
Q8C2K1	Differentially expressed in FDCP 6	Def6	FUNCTION: Phosphatidy	1.075378871	1.292071496	1.124829159	1.164093175	0.906759762	0.877344448	0.891550455	0.891884888	1.305205627	0.014642142	GO:0005911(CC:cell-cell junction);GO:00057
Q8K012	Formin-binding protein 1-like	Fnbp1l	FUNCTION: Required to c	1.104250382	1.205900813	1.143294255	1.151148483	0.885499748	1.00004707	0.785950911	0.890499243	1.292700125	0.019088084	GO:0006914(BP:autophagy);GO:0060271(BF
Q3SXB8	Collectin-11	Colec11	FUNCTION: Lectin that pl	1.181933319	1.005021874	1.212731931	1.133229041	0.847584215	0.930324351	0.873165577	0.883691381	1.282381005	0.022620089	GO:0019730(BP:antimicrobial humoral respo
P42586	Homeobox protein Nkx-2.2	Nkx2-2	FUNCTION: Transcriptior	1.23774929	1.156021257	1.028606348	1.140792298	0.978017384	0.787504609	0.924094019	0.89653867	1.272440706	0.042526424	GO:0048708(BP:astrocyte differentiation);GO
P48678	Prelamin-A/C	Lmna	FUNCTION: Lamins are c	1.205561623	1.193447543	0.997691291	1.132233486	0.944613072	0.855788875	0.887696112	0.896032686	1.263607347	0.030743052	GO:0034613(BP:cellular protein localization);
P11911	B-cell antigen receptor complex-ass	Cd79a	FUNCTION: Required in c	1.161035376	1.230270422	1.021792602	1.137699466	0.987291699	0.893151581	0.828609477	0.903017586	1.259886279	0.037647484	GO:0002250(BP:adaptive immune response)
P50396	Rab GDP dissociation inhibitor alph	Gdi1	FUNCTION: Regulates th	1.160275181	1.019120728	1.092885305	1.090760405	0.890181539	0.828414114	0.898844075	0.872479909	1.250183979	0.009280531	GO:0050771(BP:negative regulation of axonc
Q7TSJ6	Serine/threonine-protein kinase LAT	Lats2	FUNCTION: Negative reg	1.113448715	1.13426852	1.13529059	1.127669275	0.899522784	0.943351359	0.874125	0.905666381	1.24512657	0.000490511	GO:0051301(BP:cell division);GO:0034613(B
Q04750	DNA topoisomerase 1	Top1	FUNCTION: Releases the	1.161415818	1.064191513	1.121425913	1.115677748	0.894275182	0.906491961	0.905922954	0.902230032	1.236577933	0.00169824	GO:0006338(BP:chromatin remodeling);GO:0
P01749	Ig heavy chain V region 3	Ighv1-61		1.185522834	1.145357322	1.043305685	1.124728614	0.947110049	0.913331334	0.869911839	0.910117741	1.235805615	0.010959051	GO:0050853(BP:B cell receptor signaling pat
P00688	Pancreatic alpha-amylase	Amy2		1.142971319	1.117127449	1.09889526	1.119664676	0.965791863	0.846259031	0.922117246	0.91138938	1.228525042	0.004990192	GO:0016052(BP:carbohydrate catabolic proc
O55143	Sarcoplasmic/endoplasmic reticulur	Atp2a2	FUNCTION: This magnes	1.056581587	1.104881743	1.193983886	1.118482405	1.019811511	0.910697743	0.81051349	0.913674248	1.224158837	0.047796067	GO:1990036(BP:calcium ion import into sarc
Q9QZR9	Collagen alpha-4(IV) chain	Col4a4	FUNCTION: Type IV colla	1.146904545	1.141134771	1.062125115	1.116721477	0.902045758	0.947357049	0.898553811	0.915985539	1.219147497	0.003126099	GO:0038063(BP:collagen-activated tyrosine k
Q921G7	Electron transfer flavoprotein-ubiqui	Etfdh	FUNCTION: Accepts elec	1.097639446	1.073406855	1.148240585	1.106428962	0.979598677	0.922110094	0.856272405	0.919327059	1.203520501	0.011111034	GO:0022900(BP:electron transport chain);GC
P62309	Small nuclear ribonucleoprotein G	Snrpg	FUNCTION: Plays role in	1.13322825	1.192652819	1.011368094	1.112416388	0.90747649	0.908442799	0.960983519	0.925634269	1.201788249	0.029296271	GO:0000398(BP:mRNA splicing, via spliceos
Q3UQN2	F-BAR domain only protein 2	Fcho2	FUNCTION: Functions in	1.112591555	1.079737377	1.110092889	1.100807273	0.899650036	0.926905529	0.925137817	0.917231127	1.200141644	0.000181938	GO:0048268(BP:clathrin coat assembly);GO:
Q62418	Drebrin-like protein	Dbnl	FUNCTION: Adapter prot	0.849201767	0.949381786	0.902871326	0.900484959	1.032198018	1.044784598	1.165216595	1.08073307	0.833216808	0.024645558	GO:0002250(BP:adaptive immune response)
O89053	Coronin-1A	Coro1a	FUNCTION: May be a cru	0.90831628	0.896142041	0.899363753	0.901274024	1.054981378	1.068936915	1.138537291	1.087485195	0.828769006	0.002040247	GO:0030036(BP:actin cytoskeleton organizat
Q9QYB5	Gamma-adducin	Add3	FUNCTION: Membrane-c	0.915704268	0.744102342	0.959971858	0.87325949	1.023375314	1.085281164	1.160303876	1.089653451	0.801410291	0.047974727	GO:0051017(BP:actin filament bundle assem

Q3TWW8	Serine/arginine-rich splicing factor 6	Srsf6	FUNCTION: Plays a role	0.870346946	0.851991667	0.896083685	0.872807433	1.089370554	1.151990898	1.072950991	1.104770814	0.790034839	0.001047045	GO:0000380(BP:alternative mRNA splicing, v
Q9Z130	Heterogeneous nuclear ribonucleop	Hnrnpdl	FUNCTION: Acts as a tra	0.77974398	0.784808321	0.726856797	0.763803033	1.130367691	1.17136568	1.283068518	1.194933963	0.639201041	0.00093845	GO:0016071(BP:mRNA metabolic process);G

Header	Description
Accession	Identifier of the protein in the FASTA. database
Protein name	Shown annotation information of protein name in FASTA database.
Gene name	Shown annotation information of gene name in FASTA database.
Function	Protein function annotaion.
Ratio (XXX/REF)	Corrected ratio of the signal intensity of the labeled channel of the sample to the reported ion peak signal value of the internal reference (REF).
Average Ratio (XXX/REF)	Average value of multiple ratios in the group
Ratio (PCP/CON)	Ratio of peptide abundance between two groups.
P value（t-test）	Statistical test of relative expression of proteins between two groups. P value (t-test) : each group of samples includes at least three or more biological repeats.
GO annotations	GO function annotation for the protein
