# Supplementary material for: Proteomic Profiling and Functional Analysis of B Cell-Derived Exosomes upon Pneumocystis Infection
Source: J Immunol Res. 2022 Apr 14;2022:5187166. doi: 10.1155/2022/5187166 (PMC9023222; doi:10.1155/2022/5187166)
Supplement: Supplementary Materials — Supplementary Table 1: initial culture cell number and protein amount of B cell exosomes used for mass spectrometry. Supplementary Table 2: all identified proteins of uninfected and Pneumocystis-infected B cell exosomes. Supplementary Table 3: SignalP and SecretomeP prediction results. Supplementary Table 4: differentially expressed proteins of B cell exosomes in response to PCP. Supplementary Table 5: quantitative information of peptides and proteins by PRM analysis. [file 5187166.f1.zip › Supplementary Table 5-quantitative information of peptides and proteins by PRM analysis.pdf]

Supplementary Table 5：quantitative information of peptides and proteins by PRM analysis

| | | | Normalized Peak Area | | | | | | Average Peptide Abundance | | Peptide Ratio | T test p value |
|---|---|---|---|---|---|---|---|---|---|---|---|---|
| **Peptide Sequence** | **Protein ID** | **Protein Description** | **PCP-1** | **PCP-2** | **PCP-3** | **CON-1** | **CON-2** | **CON-3** | **average_PCP** | **average_CON** | **Ratio_PCP/CON** | **TTEST_PCP/CON** |
| ATPEPSGTPSSDTVSR | O89053 | Coronin-1A | 1.151968778 | 1.332357415 | 2.602001364 | 2.235021558 | 2.269462798 | 3.067867268 | 1.695442519 | 2.524117208 | 0.671697223 | 0.193783072 |
| EPVITLEGHTK | O89053 | Coronin-1A | 0.282396097 | 0.248033755 | 0.296388022 | 0.530635665 | 0.34156253 | 0.615967164 | 0.275605958 | 0.49605512 | 0.555595432 | 0.055386725 |
| QVALWDTK | O89053 | Coronin-1A | 0.985809491 | 1.326201545 | 1.521906316 | 1.623070624 | 1.323673753 | 1.756669788 | 1.277972451 | 1.567804722 | 0.815134967 | 0.225196713 |
| ACEPGVDYVYK | P01027 | Complement C3 | 40.52804272 | 52.82334264 | 102.2888522 | 68.27273714 | 54.99198027 | 70.51613634 | 65.21341251 | 64.59361792 | 1.009595291 | 0.976149897 |
| DSITTWEILAVSLSDK | P01027 | Complement C3 | 7.635743175 | 0.18864455 | 0.550996249 | 1.549447602 | 2.167241214 | 2.464381593 | 2.791794658 | 2.060356803 | 1.355005431 | 0.779211887 |
| EVVADSVWVDVK | P01027 | Complement C3 | 30.82335213 | 11.97286865 | 10.67332731 | 24.32859168 | 37.90816815 | 19.38406532 | 17.8231827 | 27.20694172 | 0.655096882 | 0.333918227 |
| SSVAVPYVIVPLK | P01027 | Complement C3 | 66.10749508 | 140.8259892 | 191.1324913 | 123.9299301 | 127.9445562 | 133.2284926 | 132.6886585 | 128.3676596 | 1.033661117 | 0.911277035 |
| TIYTPGSTVLYR | P01027 | Complement C3 | 0.200724096 | 0.215312018 | 0.466227595 | 0.213351495 | 0.270857273 | 0.266243261 | 0.294087903 | 0.250150676 | 1.175643046 | 0.644247716 |
| EEAESTLQSFR | P20152 | Vimentin | 1.607872632 | 2.330265805 | 3.208771178 | 1.887867661 | 1.605800536 | 1.628150351 | 2.382303205 | 1.707272849 | 1.395385164 | 0.225612503 |
| ETNLESLPLVDTHSK | P20152 | Vimentin | 0.858432195 | 1.301458532 | 1.559633516 | 0.801701657 | 0.922388203 | 0.964251991 | 1.239841415 | 0.89611395 | 1.383575621 | 0.17777516 |
| ISLPLPTFSSLNLR | P20152 | Vimentin | 3.357198986 | 0.206729659 | 0.222299413 | 0.423364723 | 0.823159054 | 0.541933128 | 1.262076019 | 0.596152302 | 2.117036227 | 0.561913297 |
| YTVGGSETFDSLTDLVEHFK | P29351 | ine-protein phosphatase non-receptor t | 0.012760137 | 0.002182346 | 0.001866377 | 0.020684317 | 0.030966916 | 0.021519882 | 0.005602953 | 0.024390371 | 0.229719879 | 0.018142029 |
| ALAAAGYDVEK | P43277 | Histone H1.3 | 19.02271037 | 29.92231029 | 43.61074073 | 11.51249273 | 11.91527818 | 12.39742382 | 30.85192046 | 11.94173157 | 2.583538264 | 0.056580988 |
| ASGPPVSELITK | P43277 | Histone H1.3 | 36.83427351 | 41.49116261 | 64.86951449 | 16.16148248 | 16.37221105 | 25.81502411 | 47.7316502 | 19.44957255 | 2.454123353 | 0.037621158 |
| GTGASGSFK | P43277 | Histone H1.3 | 6.122383692 | 7.863377072 | 15.06960971 | 0.674575789 | 4.016087331 | 0.446156226 | 9.685123491 | 1.712273115 | 5.656295952 | 0.055069629 |
| AAYEAELGDAR | P48678 | Prelamin-A/C | 0.629417355 | 0.741154815 | 1.016899189 | 0.752533382 | 0.68909796 | 0.850978207 | 0.795823786 | 0.764203183 | 1.04137722 | 0.811893341 |
| SGAQASSTPLSPTR | P48678 | Prelamin-A/C | 1.041598104 | 0.822256911 | 1.126890211 | 1.028910842 | 0.688918414 | 0.878904825 | 0.996915075 | 0.865578027 | 1.151733344 | 0.381966206 |
| VAVEEVDEEGK | P48678 | Prelamin-A/C | 0.044115521 | 0.069633052 | 0.104567038 | 0.041237303 | 0.085739251 | 0.065156648 | 0.07277187 | 0.064044401 | 1.136272171 | 0.708530251 |
| DLEEATLQHEATAAALR | Q5SX39 | Myosin-4 | 0.163895568 | 0.29281024 | 0.316141626 | 0.175718356 | 0.188954323 | 0.073570966 | 0.257615811 | 0.146081215 | 1.763510872 | 0.135357118 |
| LASADIETYLLEK | Q5SX39 | Myosin-4 | 0.209824937 | 0.262281905 | 0.282276706 | 0.347153897 | 0.392553624 | 0.114705736 | 0.251461183 | 0.284804419 | 0.882925846 | 0.726139096 |
| LLDEEEATDNDLR | Q9WU78 | ogrammed cell death 6-interacting prote | 0.012815294 | 0.015123718 | 0.02685852 | 0.019920268 | 0.030649718 | 0.038790215 | 0.018265844 | 0.029786734 | 0.613220778 | 0.174316808 |
| DIPVPKPK | PRTC_Heavy_peptide | | 1 | 1 | 1 | 1 | 1 | 1 | | | | |

| | Relative Protein Abundance | | | | | | | Average Protein Abundance | | Protein Ratio | T test p value |
|---|---|---|---|---|---|---|---|---|---|---|---|
| **Protein Name** | **Protein Description** | **PCP-1** | **PCP-2** | **PCP-3** | **CON-1** | **CON-2** | **CON-3** | **average_PCP** | **average_CON** | **Ratio_PCP/CON** | **TTEST_PCP/WT** |
| O89053 | Coronin-1A | 0.806724789 | 0.968864238 | 1.473431901 | 1.462909282 | 1.31156636 | 1.813501407 | 1.083006976 | 1.529325683 | 0.708159804 | 0.148510505 |
| P01027 | Complement C3 | 29.05907144 | 41.2052314 | 61.02237892 | 43.65881161 | 44.65656061 | 45.17186383 | 43.76222726 | 44.49574535 | 0.983514871 | 0.94108454 |
| P20152 | Vimentin | 1.941167938 | 1.279484666 | 1.663568036 | 1.03764468 | 1.117115931 | 1.04477849 | 1.628073546 | 1.066513034 | 1.526538819 | 0.044028518 |
| P29351 | ine-protein phosphatase non-receptor t | 0.012760137 | 0.002182346 | 0.001866377 | 0.020684317 | 0.030966916 | 0.021519882 | 0.005602953 | 0.024390371 | 0.229719879 | 0.018142029 |
| P43277 | Histone H1.3 | 20.65978919 | 26.42561666 | 41.18328831 | 9.449517 | 10.76785885 | 12.88620139 | 29.42289805 | 11.03452575 | 2.666439748 | 0.041169611 |
| P48678 | Prelamin-A/C | 0.571710326 | 0.544348259 | 0.749452146 | 0.607560509 | 0.487918542 | 0.59834656 | 0.621836911 | 0.564608537 | 1.101359384 | 0.487456049 |
| Q5SX39 | Myosin-4 | 0.186860252 | 0.277546073 | 0.299209166 | 0.261436127 | 0.290753974 | 0.094138351 | 0.254538497 | 0.215442817 | 1.18146662 | 0.607499276 |
| Q9WU78 | ogrammed cell death 6-interacting prote | 0.012815294 | 0.015123718 | 0.02685852 | 0.019920268 | 0.030649718 | 0.038790215 | 0.018265844 | 0.029786734 | 0.613220778 | 0.174316808 |
| PRTC_Heavy_peptide | | 1 | 1 | 1 | 1 | 1 | 1 | | | | |
